# Fluid flow shear stress and tissue remodeling—an orthodontic perspective: evidence synthesis and differential gene expression network analysis

**DOI:** 10.3389/fbioe.2023.1256825

**Published:** 2023-09-18

**Authors:** Mustafa Nile, Matthias Folwaczny, Andrea Wichelhaus, Uwe Baumert, Mila Janjic Rankovic

**Affiliations:** ^1^ Department of Orthodontics and Dentofacial Orthopedics, LMU University Hospital, LMU Munich, Munich, Germany; ^2^ Department of Conservative Dentistry and Periodontology, LMU University Hospital, LMU Munich, Munich, Germany

**Keywords:** periodontal ligament cells, osteoblasts, osteocytes, mesenchymal stem cells, tissue remodeling, fluid flow shear stress, mechanobiology, *In vitro* models

## Abstract

**Introduction:** This study aimed to identify and analyze *in vitro* studies investigating the biological effect of fluid-flow shear stress (FSS) on cells found in the periodontal ligament and bone tissue.

**Method:** We followed the PRISMA guideline for systematic reviews. A PubMed search strategy was developed, studies were selected according to predefined eligibility criteria, and the risk of bias was assessed. Relevant data related to cell source, applied FSS, and locus-specific expression were extracted. Based on this evidence synthesis and, as an original part of this work, analysis of differential gene expression using over-representation and network-analysis was performed. Five relevant publicly available gene expression datasets were analyzed using gene set enrichment analysis (GSEA).

**Result:** A total of 6,974 articles were identified. Titles and abstracts were screened, and 218 articles were selected for full-text assessment. Finally, 120 articles were included in this study. Sample size determination and statistical analysis related to methodological quality and the ethical statement item in reporting quality were most frequently identified as high risk of bias. The analyzed studies mostly used custom-made fluid-flow apparatuses (61.7%). FSS was most frequently applied for 0.5 h, 1 h, or 2 h, whereas FSS magnitudes ranged from 6 to 20 dyn/cm^2^ depending on cell type and flow profile. Fluid-flow frequencies of 1 Hz in human cells and 1 and 5 Hz in mouse cells were mostly applied. FSS upregulated genes/metabolites responsible for tissue formation (AKT1, alkaline phosphatase, BGLAP, BMP2, Ca^2+^, COL1A1, CTNNB1, GJA1, MAPK1/MAPK3, PDPN, RUNX2, SPP1, TNFRSF11B, VEGFA, WNT3A) and inflammation (nitric oxide, PGE-2, PGI-2, PTGS1, PTGS2). Protein-protein interaction networks were constructed and analyzed using over-representation analysis and GSEA to identify shared signaling pathways.

**Conclusion:** To our knowledge, this is the first review giving a comprehensive overview and discussion of methodological technical details regarding fluid flow application in 2D cell culture *in vitro* experimental conditions. Therefore, it is not only providing valuable information about cellular molecular events and their quantitative and qualitative analysis, but also confirming the reproducibility of previously published results.

## 1 Introduction

The term “mechanotransduction” indicates processes through which cells sense mechanical stimuli and convert them into biological signals. Fluid flow shear stress (FSS) is a mechanobiological stimulus that is exerted by flowing fluids on cells within microporous tissues. Such stimulation, by virtue of altering the local interstitial and blood fluid flow, affects the behavior of cells by inducing biochemical and biophysical changes such as gene expression, activating signaling pathways and protein synthesis, which in turn would influence cell behavior such as proliferation, migration, differentiation, and apoptosis.

Interstitial fluid is considered a major component of the body mass and is commonly found in the extracellular matrix (Wittkowske et al., 2016). The term “interstitial fluid” is so-called due to plasma leakage from blood capillaries into the interstitial space, which is then returned to blood circulation by lymphatic drainage. Research has suggested that the main function of this fluid is to facilitate cell nutrition and waste removal (Cowin and Cardoso, 2015). Osmotic and hydrostatic pressure differences between blood capillaries, interstitial space, and lymphatics are the main driving forces behind this slow but constant interchange. Tensile stresses generated within the tissue matrix during function induce interstitial fluid flow changes, which elect signaling cascades by the surrounding mechanosensing cells (Tang et al., 2014; Jiang et al., 2016; Wittkowske et al., 2016; Li et al., 2019a).

**FIGURE 1:**
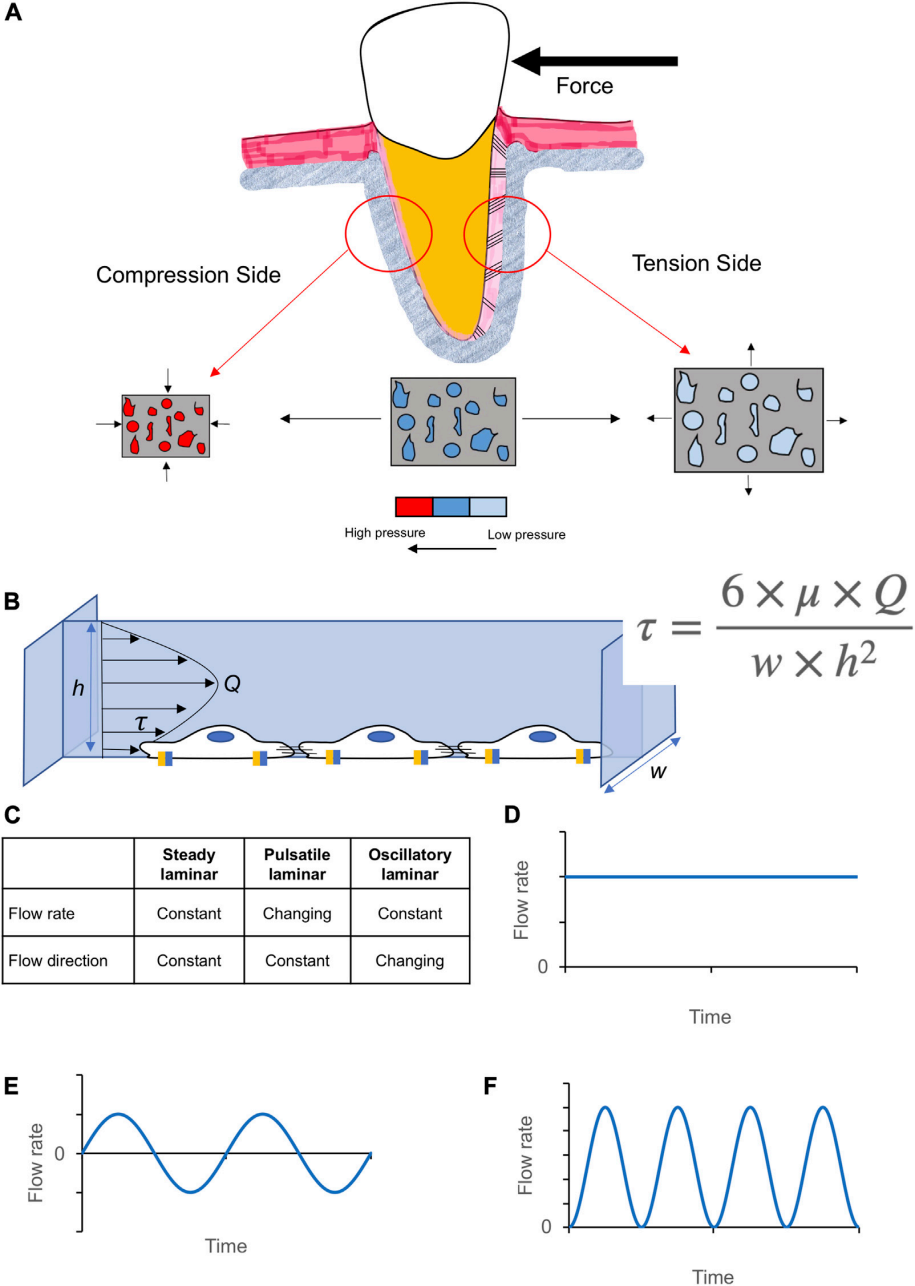
Generation of fluid flow in orthodontic tooth movement, its calculation, and modalities. **(A)** The effect of compression and stretching of a poro-elastic tissue on porosity size and pore pressure. **(B)** Illustration of fluid flow shear stress (τ) within a rectangular chamber (Q, flow rate; µ, viscosity of medium; height, h, and width, w, of the parallel flow chamber). Based on this equation, a change in the dimension of porosities influences the magnitude of FSS generated on cells. **(C)** Three common fluid flow patterns: steady laminar **(D)**, pulsatile laminar **(E)**, and oscillatory laminar flow **(F)**. The fluid flow profile of each is determined by its flow rate and flow direction.

Bone is a mechanosensing tissue and has a remarkable ability in sensing mechanical cues to maintain its structural integrity and functions. Mechanical stimulation perceived by skeletal cells, in particular interstitial FSS, guide bone remodeling, which is a physiological lifelong process. Another tissue known for its mechanosensing properties and closely related to bone remodeling is the periodontal ligament (PDL), a specialized tissue sandwiched between the tooth root and the alveolar tooth socket (Naveh et al., 2018). Earlier research on PDL mechanical properties highlighted the importance of circulatory pressure in maintaining the mechanical properties of the PDL (Bien, 1966). Recent research has suggested that fluid-filled PDL porosities and spaces give it a hydromechanical coupling property (Ashrafi et al., 2020), which is not only an important part of healthy masticatory function but may also be related to the tissue response during orthodontic treatment (Krishnan and Davidovitch, 2009). Orthodontic treatment is widely used in dentistry to correct dental malocclusion, improve function and dentofacial aesthetics. Moving a tooth using an orthodontic appliance is force- and time-dependent. When a constant force is applied to a tooth, several mechanical and biological changes occur within the periodontium around the tooth root. One of these mechanical changes is the disruption of interstitial fluid flow balance within and between the PDL and the bone. The disruption of fluid flow movement happens due to the stretching and compression of fluid-filled spaces and capillaries in the periodontal region, thus leading to differences in pore pressure (Ashrafi et al., 2020). Mathematical and *in silico* studies have suggested that porosity and permeability, properties of poro-elasticity, are influenced by pore pressure generated in tissues by mechanical loading (Weinbaum et al., 1994; Zeng et al., 1994; Chen et al., 1998; Ashrafi et al., 2020) (Figure 1A). Consequently, FSS is generated within porous tissues of the periodontium due to the flow of interstitial fluid along the pore pressure gradient (Wittkowske et al., 2016) (Figure 1B). The cellular changes that occur during orthodontic tooth movement (OTM) appear as bone resorption in direction of tooth movement at the compression side and as bone formation at the tension side, thus leading to tooth movement (Feller et al., 2015).

The PDL is a complex connective tissue composed of different cell types including but not limited to fibroblasts, stem cells, endothelial cells and macrophages (Marchesan et al., 2011). The presence of bone osteoblasts, osteocytes, and osteoclasts near the PDL makes these cells not less important in initiating the process of bone remodeling during OTM. It has been suggested that the loss of mechanical function is associated with the atrophy of PDL and extensive resorption of alveolar bone (Cohn, 1965), whereas excessive force leads to a loss of alveolar bone support (Nogueira et al., 2014). Such interrelated cellular adaptation to mechanical forces requires a biological system that senses these forces. There is increasing evidence that biological fluid flow mechanisms are involved in the adaptation of both soft and hard tissue (Buga et al., 1991; Klein-Nulend et al., 1996; Li et al., 2019a). The interlink between the PDL and bone through collagen fibers and blood supply allows the transmission of physical strain across the extracellular matrix of bone, thus resulting in a fluid movement within the bone tissue (McGarry et al., 2005; Feller et al., 2015; Wittkowske et al., 2016; Ashrafi et al., 2020). FSS generated by fluid movement in response to mechanical load is sensed by cells of the PDL and bone (van der Pauw et al., 2000; Wittkowske et al., 2016; Li et al., 2019a). Cell deformation caused by different mechanical stresses excites different cell signaling pathways responsible for cell remodeling due to cell deformation (Wittkowske et al., 2016). Anatomical wise, different cell signaling mechanisms are found in cells, at the level of the cell membrane, cell attachment and cell process. Moreover, mechanoreceptors such as Ruffini nerve endings may also be involved in periodontal tissue remodeling. Research has found that stretching and FSS activate different cell signaling pathways (McGarry et al., 2005): FSS is sensed first by cell membrane, while stretching is applied directly through cell attachment (Mullender et al., 2004).

To understand the biological effect of FSS on cell behavior, different *in vitro* setups have been utilized to determine the effect of shear stress on cells in a well-controlled cell culture environment. Since the magnitude of FSS is still debatable and may vary at different parts of the human body, it would be useful to study the different FSS profiles (steady laminar, pulsatile laminar and oscillatory laminar; Figures 1C–F) and magnitudes applied on similar cell types.

To get an overview on tissue remodeling, including the one during OTM, this article focused on the cellular behavior in response to FSS including cells from both bone and periodontal ligament tissue. Therefore, the aim of this review was to i) to provide overview of findings from 2D *in vitro* experiments utilizing FSS on bone and PDL cells, summarizing experimental settings (e.g., FSS parameters, *in vitro* apparatuses and methods used in determining FSS-related cell behavior) and gene and protein/metabolite expression data. ii) To elucidate the biological behavior of PDL and bone cells subjected to FSS, collected information was further used for enrichment analysis and protein-protein interaction (PPI) network construction and analysis. Additionally, the methodological and reporting quality of the included studies were assessed using an adapted risk of bias tool (Sun et al., 2021).

## 2 Materials and methods

A systematic identification of relevant original research was performed based on the guidelines of PRISMA 2020 (Moher et al., 2009; Page et al., 2021). Since only *in vitro* studies were regarded, a registration of the study protocol in the PROSPERO database was not possible.

### 2.1 Eligibility criteria

The inclusion criteria were formulated based on the P.I.C.O. model (Schardt et al., 2007).

1. P(atient)/cells used: foremost target were primary human cell types that are known to be involved in orthopedics/orthodontics-related bone remodeling including PDL cells, osteoblasts, osteocytes, and mesenchymal stem cells. Additionally, primary mouse osteoblasts, osteocytes and PDL cells, and the mouse osteocyte-like cell line MLO-Y4 were considered. This cell line derived from long bones of a transgenic mouse expressing the immortalizing T antigen and resembles a mature osteocyte phenotype (Kato et al., 1997). Though this cell line is known not to present the full primary osteocyte phenotype (Zhang et al., 2019), a huge amount of evidence comes from studies using this cell line (Robling and Bonewald, 2020).
2. I(ntervetion): *in vitro* fluid-flow shear stress (FSS) applied to cells in 2D culture; no limitations concerning flow characteristics.
3. C(ontrol): Cells not subjected to FSS.
4. O(utcome): FSS parameters (apparatus, type of flow, magnitude, duration, and frequency); reporting expression of genes, proteins and/or metabolites related to FSS using quantitative methods including but not limited to quantitative and semi-quantitative reverse-transcriptase polymerase chain reaction (RT-qPCR, sqPCR), ELISA, Western blotting (WB), radioimmunoassay (RIA) and immunofluorescence (IF).

The exclusion criteria were adopted as follows.

1. Review article/short communication/protocol study.
2. Primary cells independent of origin, not fulfilling the inclusion criteria.
3. *In vivo*, *in silico*, *ex vivo*, or *in situ* models.
4. 3D cell culture models.
5. No FSS-related reporting on gene or protein expression or experiment.
6. Unclear/unrelated *in vitro* fluid-flow model design.
7. Article not published in English.
8. Unhealthy/sick patient or controls.
9. Co-culture studies unless monoculture data is provided.

### 2.2 Search plan and article selection

The search strategy aimed to identify studies that used *in vitro* cell models simulating bone and periodontal ligament tissues remodeling under exposure against FSS. Specific keywords were used to establish a search strategy, taking into consideration the field of study, mechanical stimulation applied, cells responsible for remodeling and the setting of the study (Table 1). The search was completed on 19.07.2021. All results were imported to EndNote^®^ X9.3.1 (Clarivate Analytics, Philadelphia, Pennsylvania, United States).

**TABLE 1:** Search strategy design used for the systematic review. The search strategy was built according to four main variables based on the topic and the scope of the project: field, force, cell type, setting. Different key words were used under each main variable to increase the detection capability.

Field	Mechanical stimulation	Cells	Setting
orthodont* OR tooth movement OR ortho* OR Dent* OR Oral OR Mechanotransduction OR bone remodeling OR Stomatology OR mechanosens* OR calcium signaling OR osteogenesis OR orthodontic tooth movement OR osteoblastogenesis OR Periodont*	Extracellular Fluid flow OR interstitial fluid flow OR "Shear Strength" [MeSH] OR Stress, "Mechanical" [MeSH] OR fluid flow OR pulsatile flow OR flow chamber OR shear stress OR FSS OR fluid shear stress OR shear strength OR laminar shear stress OR pulsatile fluid flow	PDL OR hPDLCs OR hPDLFs OR hPDLs OR bone cells OR osteo* OR bone OR periodontal ligament OR fibroblast* OR MLO-Y4 OR human osteoblast-like cells OR human fetal osteoblast-like cells OR mesenchymal stem cells OR primary human osteoblasts OR progenitor cell* OR stem cell* OR human PDL-cells OR human PDL-fibroblasts OR human PDLFs OR human PDLs	cell* OR model OR in vitro

Search: ("bone remodeling"[tw] OR "calcium signaling"[tw] OR "orthodontic tooth movement"[tw] OR "tooth movement"[tw] OR dent* OR mechanosens*[tw] OR mechanotransduction[tw] OR oral OR ortho* OR orthodont* OR osteoblastogenesis OR osteogenesis[tw] OR periodont* OR stomatology) AND ("Extracellular Fluid flow"[tw] OR "flow chamber"[tw] OR "fluid flow"[tw] OR "fluid shear stress"[tw] OR "interstitial fluid flow"[tw] OR "laminar shear stress"[tw] OR "pulsatile flow"[tw] OR "pulsatile fluid flow"[tw] OR "Shear Strength"[MeSH] OR "shear stress"[tw] OR "Stress, Mechanical"[MeSH] OR FSS[tw]) AND ("bone cells"[tw] OR "human fetal osteoblast-like cells"[tw] OR "human osteoblast-like cells"[tw] OR "human PDL-cells"[tw] OR "human PDL-fibroblasts"[tw] OR "human PDLFs"[tw] OR "human PDLs"[tw] OR "mesenchymal stem cells"[tw] OR "MLO-Y4"[tw] OR "periodontal ligament"[tw] OR "primary human osteoblasts"[tw] OR "progenitor cell*"[tw] OR "stem cell*"[tw] OR bone[tw] OR fibroblast*[tw] OR hPDLCs[tw] OR hPDLFs[tw] OR hPDLs[tw] OR osteo*[tw] OR PDL[tw]) AND ("in vitro"[tw] OR cell*[tw] OR model[tw])

Filters: from 1000/1/1 ‐ 2021/7/19 Sort by: Publication Date

The studies were initially filtered by reading the abstract and the title according to the established eligibility criteria. Then, full-text reading was done for the remaining studies. After full-text reading, the articles not fulfilling the inclusion criteria were excluded (Supplementary Data Sheet S1), while the remaining were considered for data extraction (Supplementary Data Sheet S2). All steps were discussed between the authors (M.N., M.J.R., U.B.) till reaching a unified decision.

### 2.3 Risk of bias assessment

The risk of bias (RoB) of the included *in vitro* studies was evaluated using two separate assessment sheets specific for methodological or reporting RoB as previously published (Sun et al., 2021) (Supplementary Data Sheet S3). A score was applied to each criterion depending on its evaluation as previously published (Sun et al., 2021): “+” (low risk of bias), “–” (high risk of bias), “?” (incomplete/unclear risk of bias), and “n.a.” (not applicable). The data entry was carried out as previously reported (Sun et al., 2021), and for each cell group the RoB assessment was tabulated (Supplementary Data Sheet S3). All steps were discussed between the authors (M.N., M.J.R., U.B.) till reaching a unified decision.

### 2.4 Data extraction

For each cell-type, data extraction tables were designed to collect data related to the experimental design and FSS-related expression outcome (Supplementary Data Sheet S2). Therein, we extracted study data as originally reported related to (i) cells used (hPDLCs, hMSCs, hOst, hOcyt, mPDLCs, mOst, mOcyt) including cell-related information (origin of cells, age, number and sex of donor, health status, tooth type, isolation method, passages used, and cell density/confluency used); (ii) fluid flow type (steady laminar, pulsatile laminar, oscillatory laminar); (iii) apparatus used in each experiment; (iv) shear stress magnitudes/frequency/duration; (v) genes and analytes (official gene symbol if applicable); (vi) the methods used to measure their expression (RT-qPCR, sqPCR, ELISA, WB, RIA, EMSA, IF), and (vii) the measured FSS-related differential expression. All steps were discussed between the authors (M.N., M.J.R., U.B.) till reaching a unified decision. The pattern of gene expression was determined as minimum/maximum expression and reported as fold change, relative expression or as a calculated ratio. Gene, protein and/or metabolite regulation was calculated and reported as ratio in relation to the corresponding control when applicable.

#### 2.4.1 Information related to FSS

The type of fluid flow was defined as “steady laminar”, “pulsatile laminar” and “oscillatory laminar” based on the information given by the author of each study. The fluid flow character was confirmed by taking “flow rate” and “flow direction” into consideration, that was delivered by the motorized apparatuses to apply FSS to cells. The whole design of the fluid flow circuit such as, e.g., the utilization of the gravity force and the usage of axillary pulse dampers were taken into consideration. Characteristics of the fluid flow apparatuses and fluid flow circuits were extracted from the publication or references mentioned therein including manufacturer websites (Supplementary Data Sheet S2). Information was regarded as “not given” (n.g.) only after carefully following the previously mentioned steps. The fluid flow frequencies were extracted in Hertz (Hz) and FSS as dyn/cm^2^ or Pascal (Pa). Herein, FSS magnitudes are reported as dyn/cm^2^, which can be converted to Pascal according to the following relationship: 10 dyn/cm^2^ ≈ 1 Pa. FSS apparatuses identified in each study were grouped and quantified in tables based on cell type and fluid flow profiles (Supplementary Data Sheet S4). A quantitative summary of the most used fluid flow apparatuses and fluid flow profiles in each cell group was then provided (Tables 3, 4). Finally, the most frequently used FSS durations and magnitudes were quantified and summarized in tables using the relationship between dyn/cm^2^ and Pascal as mentioned (Supplementary Data Sheet S4).

#### 2.4.2 Information related to genes and proteins

All genes and molecules were extracted from the included studies without changing. The official name/symbol of the identified genes was determined using the HUGO Gene Nomenclature Committee (HGNC) database^1^ for human genes, the Mouse Genome Informatics (MGI)^2^ website for mouse genes, and the Gene Database at the National Center for Biotechnology Information^3^. Primer-BLAST^4^ was used to verify the specificity of PCR primers. Specificity of protein data, antibodies or ELISA was verified using information provided in the publication and/or suppliers mentioned therein. If possible, official gene symbols according to HGNC or MGI were used. If antibody specificity was not sufficient, their targets according to the given manufacturer were recorded (Sun et al., 2021).

Gene and protein expression-related data were either directly acquired from the text source or extracted from the graphs using Engauge Digitizer Software (version 12.1) (Mitchell et al., 2019). To provide a comprehensive comparative approach of gene/protein expression data between different studies, relevant data and ratios were calculated if they were not mentioned by the study-authors. “Fold changes” were extracted if they were calculated according to Livak and Schmittgen (2001) or their calculation was reported as being “delta delta Ct” (ΔΔCt). Additionally, the following terms were introduced: “relative gene expression” was entitled to percentages or gene expression ratios normalized to the control, and not calculated by ΔΔCt; “ratio-calc” indicated manual calculation by dividing intervention/control; “ratio” indicated ratios given by study-authors such as normalization to control in case of small molecules data or in case of gene expression ratios, e.g., the ratio of RANKL/OPG or Bcl-2/Bax.

#### 2.4.3 Gene and small molecules analysis

Using the data from Supplementary Data Sheet S2, genes/metabolites were considered for further analysis, which were extracted from at least three (≥3) studies (Supplementary Data Sheet S5). This threshold aimed to accommodate variations in study numbers per cell group and ensure representative data. Studies reporting similar genes/metabolites were categorized by fluid flow profile and regulation direction (up or down). Upregulation included “increase” or “increase with plateau” patterns; downregulation encompassed “decrease” or “decrease with plateau”. All other patterns were labeled as “other” regulation changes. Repeated studies on the same gene/metabolite were counted as one. The fraction/percentage of upregulation of the same gene/metabolite was calculated as part of the total (Supplementary Data Sheet S6).

### 2.5 Evidence-synthesis-based bioinformatical analysis of differential gene expression data

#### 2.5.1 Over-representation and network analysis

**FIGURE 2:**
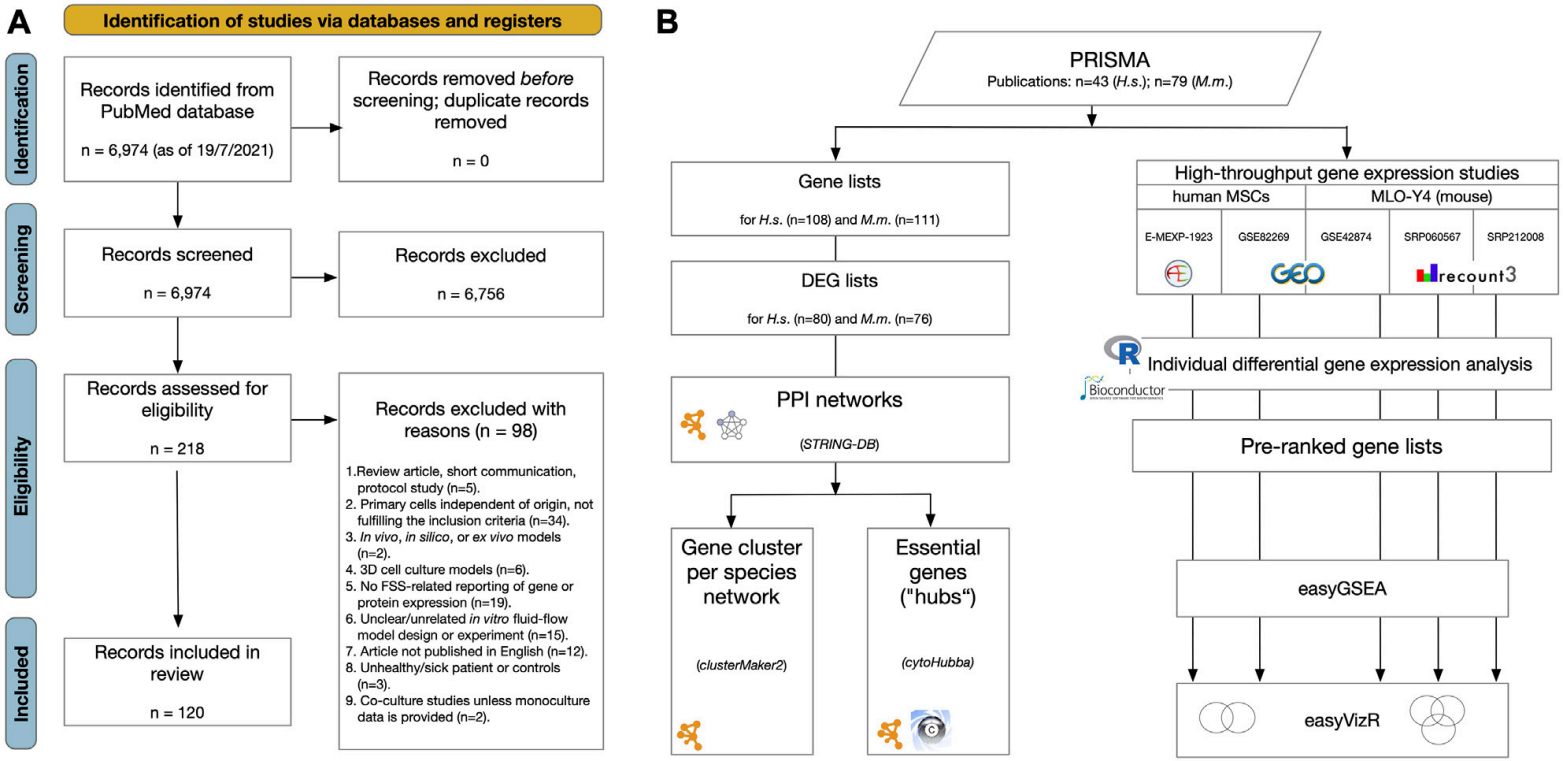
Workflows applied in this systematic review. **(A)** PRISMA2020 flow diagram for the whole process of study selection according to Moher et al. (2009) and Page et al. (2021). **(B)** Over representation and network analysis of gene lists derived from the review process. Gene lists were compiled, listing examined genes, proteins or metabolites showing differential gene expression after FSS application in primary human or mouse cell lines (Table 3, Supplementary Data Sheets S2, 6). From these, differential expressed gene (DEG) lists were generated according to the specified criteria. For both human and mouse DEG lists protein-protein interaction (PPI) networks were constructed using STRING-DB (Szklarczyk et al., 2023) (Figure 5). Pathway analysis was done using “GeneOntology/Biological Process” (Ashburner et al., 2000), KEGG (Kanehisa and Goto, 2000), and WikiPathways (Pico et al., 2008) (Supplementary Data Sheet S7). Subnetworks were identified in both PPI networks using the “*GLay community detection algorithm*” implemented in clusterMaker2 (Morris et al., 2011) (Table 6, Figure 5). Essential nodes, so-called “hub genes”, were detected using cytoHubba (Chin et al., 2014) (Figure 5, Supplementary Data Sheet S7). Altogether 5 high-throughput gene expression studies applying microarray or RNA-seq methodology were identified studying the effect of FSS on either human MSCs or the mouse osteocyte cell line MLO-Y4. Raw data were downloaded from the respective repositories and individually processed using bioinformatics workflows. Pre-ranked gene lists were generated, and gene set enrichment analysis (GSEA) was applied to each using easyGSEA (Cheng et al., 2021). Cell-type specific comparisons were done using easyVizR (Cheng et al., 2021) (Figure 6, Supplementary Data Sheet S8).

Based on the data extracted (Supplementary Data Sheet S2), two species-specific lists with differential expressed genes (DEGs) were compiled according to the previously defined criteria (Sun et al., 2021). The “human” list included DEGs from reports studying the effect of FSS on human cells (hMSCs, hOst, hPDLCs), and the “mouse” list from studies using mouse cells (mOst, mOcyt). Both DEG lists were used to generate protein-protein interaction (PPI) networks and for over-representation analysis (ORA) as described (Sun et al., 2021) (Figure 2B) with modifications described below.

Potential interactions between DEGs at protein level were analyzed using protein-protein interaction (PPI) networks, which were generated with the “*Search Tool for the Retrieval of Interacting Genes/Proteins*” database (STRING-DB) (v11.5)^5^ (Szklarczyk et al., 2019; Szklarczyk et al., 2023) using the stringApp plugin version 2.0.1 (Doncheva et al., 2019) with Cytoscape version 3.9.1 (Shannon et al., 2003; Su et al., 2014). Only high confidence interactions were included in the predicted networks, i.e., a minimum required combined score of 0.7 was applied. Cluster analysis and cluster visualization of both human and mouse PPI networks was done by applying the “*GLay Community Detection*” algorithm (Su et al., 2010) as implemented in the clusterMaker2 app version 2.3.4 (Morris et al., 2011) with default settings. Hub genes, i.e., essential genes in a network, were identified with cytoHubba version 0.1 (Chin et al., 2014) using default settings. For each node, a total score was calculated based on the eleven different local and global topological methods calculated by this plugin (Sun et al., 2021) (Supplementary Data Sheet S7). A node was considered a hub node if its total score was at least twofold higher than the mean total score of all nodes of that network (Sun et al., 2021). Networks were visualized with Cytoscape version 3.9.1.

Over-representation analysis was applied to the network with the stringApp plugin using the “*GeneOntology/Biological Process*” (Ashburner et al., 2000), WikiPathways (Pico et al., 2008; Martens et al., 2021), and the “*Kyoto Encyclopedia of Genes and Genomes*” (KEGG) (Kanehisa and Goto, 2000; Du et al., 2014) databases. As previously described, a cut-off ratio of ≥0.05 (i.e., at least 5% of the queried genes must be included in a specific database entry) was applied to increase the specificity of the enrichment terms (Sun et al., 2021). If available, the 15 most significant terms or pathways were reported. General pathways related to cancer and/or infectious diseases like “*Chagas disease*” (hsa05142) or “*Pertussis*” (hsa05133) were removed, and their accession numbers were reported. Gene set enrichment was applied to the complete networks, and to each identified cluster (Supplementary Data Sheet S7).

#### 2.5.2 High-throughput gene expression studies and pre-ranked gene set enrichment analysis (GSEA)

Though data extraction identified several high-throughput gene expression studies, comparable gene expression raw data (microarray or RNA-seq) from three of the identified studies only (Govey et al., 2014; Govey et al., 2015; Li et al., 2019b) was publicly available (Table 2). Data descriptions and the related publications were evaluated concerning experimental conditions (cell type characteristics, FSS parameters, controls) and the number of samples for each (FSS or corresponding control). Only studies and experimental conditions represented by at least two replicates each were considered. To identify additional data specific to our preselected cell types and in relation to FSS, hand-searching of Gene Expression Omnibus (GEO)^6^, ArrayExpress^7^, and the Sequence Read Archive^8^ was done. Two additional studies were identified: GSE18114 from GEO and E-MEXP-1923 from ArrayExpress (Glossop and Cartmell, 2009). Since no publication was identified related to the GSE18114 data, and the ArrayExpress dataset passed selection criteria, our reanalysis was finally based on five high-throughput gene expression studies (Table 2). In two microarray studies (GSE82269, E-MEXP-1923) FSS was applied to hMSCs (Glossop and Cartmell, 2009; Diaz et al., 2017), and in three studies FSS was applied to the mouse osteocytic-cell line MLO-Y4. The latter consists of one microarray study (GSE42874) (Govey et al., 2014) and two RNA-seq studies (SRP060567/GSE70667 and SRP212008) (Govey et al., 2015; Li et al., 2019b) (Figure 2B). To ease reanalysis, uniformly processed RNA-seq gene data from both studies were downloaded from the *recount3* repository^9^ (Wilks et al., 2021). The detailed workflow is outlined in Supplementary Data Sheet S8. Shortly, the raw data were downloaded from the specific repositories and analyzed separately with workflows specific to the platform used (microarray, RNA-seq) (Figure 2B). Pre-ranked gene lists were exported, and gene set enrichment analysis (GSEA) was applied using easyGSEA^10^ (Cheng et al., 2021) using the provided gene set definitions from KEGG, GO/Biological Process, and WikiPathways. For each cell type (hMSCs, mouse MLO-Y4), shared features were identified separately using easyVizR using default filtering settings (*p* < 0.05) (Figure 2B) (Cheng et al., 2021). All comparison results with FDR <0.1 in either of the easyGSEA results were retained. No further filtering was applied (Supplementary Data Sheet S8). As mentioned above, general pathways related to cancer and/or infectious diseases like “*C. disease*” (hsa05142) or “*Pertussis*” (hsa05133) were removed, and their accession numbers reported.

**TABLE 2:** Summary of the high-throughput gene expression studies analyzed with pre-ranked GSEA.

Study Id	References	Platform	Type	Cell type	FSS parameters	Samples
E-MEXP-1923 (AE)	Glossop and Cartmell (2009)	Affymetrix GeneChip Human Genome U133 Plus 2.0 [HG-U133_Plus_2]	Microarray (probe)	Human bone marrow MSCs from 1 female donor (23 years old; commercial source)	FSS: 1 dyn/cm^2^ for 1 h and 2 h post-FSS; Controls: sham treated	2 samples FSS, 2 samples control
GSE82269 (GEO)	Diaz et al. (2017)	Illumina HumanHT-12 V4.0 Expression BeadChip (GPL10558)	Microarray (BeadArray)	Human bone marrow MSCs from 3 donors (1 female, 20years; 2 male, 22 and 25 years; commercial source)	FSS: 15 dyn/cm^2^ for 6 h; Controls: sham treated	3 samples FSS, 3 samples control (batch correction: donor)
GSE42874 (GEO)	Govey et al. (2014)	Affymetrix Mouse Genome 430A 2.0 Array (GPL8321)	Microarray (probe)	Mouse MLO-Y4 osteocyte-like cell line	FSS: sinusoidally oscillating FF w/peak shear stress of 10 dyn/cm^2^ @ 1 Hz for 2 h and post-FSS incubation for 0/2/8/24 h; Controls: paired sham treated	3 samples FSS (2 h FSS and 2 h post-FSS), 3 samples control
GSE70667 (recount3: SRP060567)	Govey et al. (2015)	Illumina HiSeq 2500 (*Mus musculus*) (GPL17021)	RNA-seq	Mouse MLO-Y4 osteocyte-like cell line	FSS: sinusoidally oscillating FF w/peak shear stress of 10 dyn/cm^2^ @ 1 Hz for 2 h and post-FSS incubation for 2 h; Controls: paired sham treated	3 samples FSS, 3 samples control
SRP212008 (recount3)	Li et al. (2019b)	Illumina NextSeq 550	RNA-seq	Mouse MLO-Y4 osteocyte-like cell line	FSS: 15 dyn/cm^2^ oscillatory FSS @ 1 Hz for 2 h; Controls: sham treated	3 samples FSS, 3 samples control

GEO, Gene Expression Omnibus (https://www.ncbi.nlm.nih.gov/geo/; AE, ArrayExpress (https://www.ebi.ac.uk/biostudies/arrayexpress/); recount3 study explorer (https://jhubiostatistics.shinyapps.io/recount3-study-explorer/).

## 3 Results

### 3.1 Study selection

The study selection process was summarized in the PRISMA 2020 flow chart (Page et al., 2021) (Figure 2A). The finalized search strategy identified 6,974 unique studies. No articles were added using manual search of specific journals or reference chaining. After duplicate removal, title/abstract of all remaining records were screened, and 6,756 articles were excluded according to the predefined criteria. The remaining 218 reports were assessed for eligibility by full-text reading, and 98 articles were excluded in obedience to the pre-defined exclusion criteria (Figure 2A; Supplementary Data Sheet S1). Finally, 120 studies were included in this systematic review for data extraction and subsequent differential gene expression network analysis. No studies were identified related to mouse PDL cells.

### 3.2 Risk of bias assessment

**FIGURE 3:**
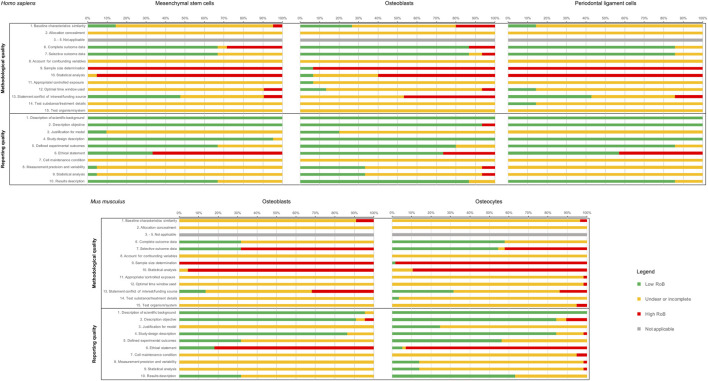
Summaries of the Risk of Bias (RoB) assessment of the included studies. In the first row, the RoB of the studies on human primary cells (MSCs, osteoblasts, and PDLCs), and in the second row the studies on primary mouse cells (osteoblasts, osteocytes) are shown. For each cell type, both RoB categories (reporting and methodological RoB) are given. RoB scoring was applied as follows: “+”, low risk of bias (LoB); “-”“, high risk of bias (HoB); “?”, not applicable or incomplete/unclear risk of bias.

The assessment of methodological quality was based on 15 predefined criteria (Supplementary Data Sheet S3). Overall, a large variability was observed between cell groups (Figure 3). Generally, a high risk of bias score was mostly assigned for “Statistical analysis” and “Sample size determination”. For both, primary mouse osteoblasts (mOst) and mouse osteocytes (mOcyt) a high risk of bias ratio in “Reporting bias: selective outcome data” was identified. Publications reporting on human primary osteoblasts (hOst) cell group showed an increased risk of bias ratio for the criterion “Statement conflict of interest/funding” compared to other cell groups. “Incomplete/unclear risk of bias” figure was most frequently identified for “Test organism/system”, “Test substance/treatment details”, “confounding bias” and “selection bias: allocation concealment”.

Concerning the reporting quality assessment (Figure 3), which was based on 10 predefined criteria (Supplementary Data Sheet S3), a high risk of bias figure was mostly assigned for the criterion “Ethical statement”, while incomplete/unclear risk of bias was assigned for “Cell maintenance conditions” in most of the studies. Due to the differences in the number of studies included in each cell group, a groupwise comparison was considered not conclusive.

### 3.3 Fluid flow characteristics

Fluid flow characteristics were defined based on fluid flow direction and rate as well as the fluid flow systems used to create the fluid movement in each study (Figure 1C, Table 3). Oscillatory laminar fluid flow was the most investigated fluid flow type in mOcyt- (21/58; 32.6%) and hMSCs-related studies (10/21; 47.6%). Both, steady laminar and pulsatile laminar fluid flow characters were equally investigated in mOst-related studies (9/22; 40.9%). Nevertheless, pulsatile laminar fluid flow was mainly used in hOst cell group (10/14; 71.4%). Steady laminar fluid flow was predominantly used in human periodontal ligament (hPDLCs) cell group (5/7; 71.4%), while pulsatile laminar fluid flow was used by the only identified study of human osteocytes (hOcyt).

**TABLE 3:** A numerical summary of fluid flow pattern in relation to each cell group type (Figures 1C–F; Supplementary Data Sheet S4).

Cell type (abbreviation)	No. of studies	Flow profiles				No. of flow profiles used
		Steady laminar	Pulsatile laminar	Oscillatory laminar	Unknown	
**Human MSCs (hMSCs)**	21	8	2	10	1	21
**Human osteoblasts (hOst)**	14	2	10	2	0	14
**Human osteocytes (hOcyt)**	1	0	1	0	0	1
**Human PDL (hPDLCs)**	7	5	2	0	0	7
**Mouse osteoblasts (mOst)**	22	9	9	4	0	22
**Mouse osteocytes (mOcyt)**	57	15	19	21	3	58
**Total**	122	39	43	37	4	123

### 3.4 Fluid flow apparatus for shear stress application

Fluid flow systems were analyzed for each cell group by taking into consideration the whole fluid-flow experimental setting including but not limited to the type of motor used, accessory fluid flow pulsation damper and the utilization of gravity forces. Afterwards, the FSS systems were compared with the fluid flow description by each author (Table 4).

**TABLE 4:** A numerical summary of fluid flow chamber type in relation to cell group type (for full details refer to Supplementary Data Sheet S4).

Chamber type	Mouse		Human				Total	Fluid flow type
	Osteocytes	Osteoblasts	MSCs	Osteoblasts	PDLCs	Osteocytes		
**Custom-made**	35	11	12	10	7	1	76	Steady laminar, oscillatory laminar, pulsatile laminar
**Rocking culture system**	1	1	1	1	0	0	4	Oscillatory laminar
**Streamer, Streamer Gold, FlexFlow (Flexcell Inc.)**	8	0	4	0	0	0	12	Steady laminar, oscillatory laminar, pulsatile laminar
**IBIDI**	2	2	2	0	0	0	6	Steady laminar, oscillatory laminar, pulsatile laminar
**Rotational orbital shaker**	1	0	1	1	0	0	3	Oscillatory laminar
**Cytodyne^a^**	2	4	0	2	0	0	8	Steady laminar, pulsatile laminar
**PeCon parallel plate (PeCon GmbH) ^a^**	1	0	0	0	0	0	1	Oscillatory laminar
**Focht Chamber System 2 (Bioptechs Inc.) ^a^**	1	0	0	0	0	0	1	Pulsatile
**Bioflux system (Fluxion Biosciences)**	0	1	0	0	0	0	1	Steady laminar
**Not given**	7	3	1	0	0	0	11	Laminar, oscillatory laminar, pulsatile laminar
Total	58	22	21	14	7	1	123	

^a^ No recent information available.

Different fluid flow systems were used to deliver steady laminar, pulsatile laminar and oscillatory laminar FSS on cells. Most of the identified fluid flow systems were custom-made (76/123; 61.7%) and were used to deliver either of the three fluid flow types. To deliver all previously mentioned fluid flow types, commercial systems from two companies were identified: Flexcell Inc (12/123; 9.7%) and ibidi (6/123; 4.8%). Flexcell Inc (Flexcell Corporation, Hillsborough, NC, United States) provides two fluid flow systems called FlexFlow™ and FlexCell Streamer^®^. FlexFlow™ consists of a single chamber and can be used either for tension or FSS applications, whereas the FlexCell Streamer^®^ is used to apply FSS to up to 6 culture slides in parallel. Both fluid flow variants provide FSS from 0–35 dyn/cm^2^. Ibidi GmbH (Gräfelfing, DE) provides a fluid flow system consisting of a specific pump system, a fluidic unit and channel slides called “µ-Slide”. In some identified studies, an ibidi channel slide was integrated into a custom-made fluid flow circuit driven by pumps from various manufacturers (e.g., Fu et al., 2008; Seref-Ferlengez et al., 2016; Lee et al., 2017). A “Cytodyne flow chamber” was used in 8/123 (6.5%) of the identified studies. We were not able to find any current information related to the manufacturer or this product.

Rocking culture systems (4/123; 3.2%) and rotational orbital shakers (3/123; 2.4%) were used to create an oscillatory fluid flow culturing environment. The PeCon parallel plate system (PeCon GmbH, Erbach, DE), the Focht Chamber System 2 (Bioptechs Inc., Butler, PA, United States) and the Bioflux system (Fluxion Biosciences, Oakland, CA, United States) are fluid flow chambers used for live imaging and were identified once each.

### 3.5 Fluid flow shear stress

A summary of the most frequently used FSS magnitudes and durations in hMSCs, hOst, hPDLCs, mOst and mOcyt is provided in Supplementary Data Sheet S4. Herein, we provide a short overview of the main findings. If several FSS durations were listed, the most frequently reported one is mentioned first and the following in descending order.

#### 3.5.1 Human cells

In the hMSCs cell group, the most frequently used FSS durations with a steady laminar fluid flow profile were 0.5, 6 and 24 h and the most frequently used FSS magnitude was 2 dyn/cm^2^ for 2 h. Nevertheless, the mostly used FSS duration in the hPDLCs cell group was 2 h and the mostly used FSS magnitude was 6 dyn/cm^2^ for 2 h or 4 h. For oscillatory fluid flow, the mostly applied FSS durations in the hMSCs cell group, were 2, 1 and 0.5 h and the mostly used FSS magnitudes were 20 dyn/cm^2^ for 0.05 h, 5 dyn/cm^2^ for 0.05 h, 10 dyn/cm^2^ for 0.05 and 2 h. For pulsatile laminar fluid flow, the most frequently used FSS duration in the hMSCs cell group was 1 h, and the most frequently used FSS magnitude was 6 dyn/cm^2^ for 1 h. Similarly, the mostly used FSS duration in the hOst cell group was 1 h, while the most frequently applied FSS magnitude/duration combination was 7 dyn/cm^2^ for 1 h.

#### 3.5.2 Mouse cells

In the mOcyt cell group, the most frequently used FSS durations using a steady laminar fluid flow profile were 2 and 0.5 h. Magnitudes of 16 dyn/cm^2^ (for 2, 0.5, 4, 1 and 24 h) and 10 dyn/cm^2^ (for 0.16 h and 2 h) were the ones most commonly applied. In contrast, the mostly used FSS durations for the same fluid flow profile in the mOst cell group were 0.5, 1, 0.16 h and the mostly used FSS magnitude was 12 dyn/cm^2^ for 1 h. For oscillatory fluid flow, the mostly used FSS durations in the mOcyt cell group were 1 and 2 h and the mostly used FSS magnitudes were 10 dyn/cm^2^ for 2 and 1 h and 20 dyn/cm^2^ for 1 and 0.15 h. For the same fluid flow profile, the mostly used FSS durations in the mOst cell group were 0.5 and 1 h. For pulsatile laminar fluid flow, the most frequently used FSS durations in the mOcyt cell group, were 1 and 0.5 h and the mostly used FSS magnitudes were 7 dyn/cm^2^ for 1 h, 10 dyn/cm^2^ for 0.5, 0.16 and 1 h and 4 dyn/cm^2^ for 0.5, 1 and 2 h. Similarly, the most frequently used FSS durations listed, were 1, 0.5 h in the mOst cell group. However, the mostly used FSS magnitude was 6 dyn/cm^2^ for 0.083, 0.5 and 1 h.

### 3.6 Fluid flow frequencies

The most frequently used fluid flow frequencies were 1 Hz in the hMSCs cell group and 5 Hz in hOst, hOcyt and hPDLCs cell groups. A frequency of 5 Hz was reportedly used two times more than 1 Hz in the mOst cell group, while equally used in the mOcyt cell group (Supplementary Data Sheet S4).

### 3.7 Analysis of gene, protein, or metabolite expression

**FIGURE 4:**
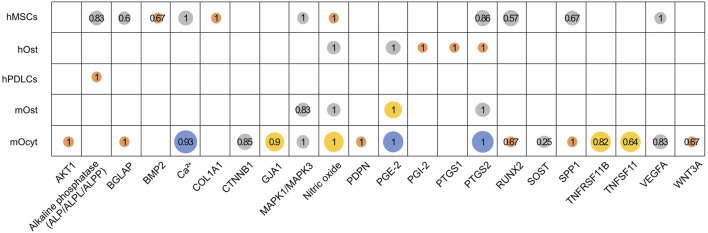
Proportion of studies reporting an upregulation of genes or metabolites in human and mouse cell groups. Altogether 22 genes or metabolites were identified, which were investigated by at least three studies in the mentioned cell groups (hMSCs, human MSCs; hOst, human osteoblasts; hPDLCs, human PDL cells; mOst, mouse osteoblasts; mOcyt, mouse osteocytes). Bubble size and its color represents the number of studies (● 3, ● 4–7, ● 8–11 or ● ≥ 12). The fraction of studies reporting an upregulation is given for each gene or metabolite. It was calculated as total number of studies reporting an upregulation divided by the total number of studies reporting the specific gene or metabolite (Supplementary Data Sheet S6).

Though species-specific data was extracted and analyzed, in the following sections gene symbols were written according to human gene nomenclature independent of the species originally reported in the relevant study (i.e., all uppercase). Information on the experimental conditions related to cell culture and FSS application and the differential expression of genes, proteins, or metabolites after FSS application was summarized in Supplementary Data Sheet S2. Due to the huge body of information, the focus in this section was placed on the most frequently studied genes, proteins, or metabolites (Supplementary Data Sheets S5, 6; Figure 4).

Expression of genes, proteins, or metabolites related to FSS application was extracted from the 120 included studies, of which 43 studies reported on human and 79 studies reported on mouse cell types, including two studies that investigated both species (Figure 2B). The human list contained 108 and the mouse list contained 111 (Table 5). Genes or proteins that were not clearly assigned to a specific gene symbol due to ambiguities in the reported PCR primers or antibodies used in WB or ELISA assays were included (e.g., “MAPK3/MAPK1”, “GSK3A/GSK3B”) (Table 5). Additionally, information on posttranslational modifications like phosphorylation or protein translocation between cytoplasm and nucleus were extracted (Supplementary Data Sheet S2), but not included in the following considerations.

**TABLE 5:** Genes, proteins, and metabolites analyzed in the included studies in primary human or mouse cell lines (Supplementary Data Sheet S2). Differential expressed genes (DEGs) used for the subsequent over-representation analysis (ORA) are given in bold (Figures 2B, 5; Supplementary Data Sheet S7).

Human	Mouse
**ADIPOQ**, AKT1, **ALPL**, **BAX**, **BCL2**, **BGLAP**, **BMP2**, **BMP7**, Calcium, CCL2 (?), **CCL3**, **CCL5**, **CD44**, **COL1A1**, CTNNB1, **CXCL8**, **CYP24A1**, **CYP27B1**, **DUSP6**, EGF, **EGR1**, **ENG**, **FGF2**, **FOS**, **FOSB**, **FOSL1**, **FOSL2**, **GADD45B**, **GLI1**, GSK3B, HGF, **HIF1A**, **HMOX1**, **IBSP**, **IER3**, **IGF1**, IGF2, **IGFBP1**, **IL1B**, **IL1RN**, **IL6**, **ITGB1**, **JUN**, **KDR**, **MAP3K8**, MAPK14/MAPK11/MAPK12, MAPK3/MAPK1, **MAPK8**, **MMP1**, **MMP2**, **MRTFA**, MTOR, **MYH11**, **MYH2**, **NANOG**, NFATC1, **NFKB1**, Nitric oxide, **NOS1**, **NOS2**, **NOS3**, **PDGFA**, PDGFB, **PDGFRA**, **PDGFRB**, **PECAM1**, PGE-2, PGF2α, PGI-2, PIK3CB, **PON1**, **POU5F1**, **PPARA**, PPP1R12A, **PTCH1**, **PTGES**, **PTGS1**, **PTGS2**, PTK2, ratio (BCL2/BAX), ratio (RANKL/OPG), RCAN1, **RHOA**, RPS6KB1, **RUNX2**, **S100A4**, **S100A7**, **S100A8**, **SOX2**, **SP7**, **SPP1**, SRC, **TAGLN**, **TGFB1**, TGFB3, **THY1**, **TIMP1**, **TIMP2**, TNAP, **TNFAIP6**, **TNFRSF11B**, **TNFSF11**, **TRPV1**, **TRPV4**, **VDR**, **VEGFA**, **VWF**, **WIF1**	6-keto-PGF1α, **ACTB**, AKT1, **ALPL**, **APC**, **AXIN1**, **BAD**, **BAX**, BCL2, **BGLAP**, **BMPR1A**, Calcium, cAMP, **CASP3**, CASP3/CASP7, CCL2, CCL3, CCL4, CCL5, **CCL7**, **CCN1**, **CCND1**, **CD44**, CSF2, **CTNNB1**, **CXCL1**, **CXCL2**, cyclic AMP, **DKK1**, **DKK2**, **DLX1**, **DLX5**, **DMP1**, EFNA2, EFNB2, EPHA2, EPHB4, **ERAL1**, **ESR1**, **FABP4**, FLT1, **FOS**, **FZD6**, **GJA1**, **GJC1**, GSK3A, GSK3A/GSK3B, GSK3B, **HGF**, **IGF1**, **IGF1R**, **IL11**, **IL6**, **ITGAV**, ITGB1, **ITGB3**, JAK2, **JUN**, KDR, **LEF1**, **LEPR**, **LIF**, LIMK2, **LPL**, **LRP5**, MAPK3/MAPK1, **MEPE**, **MKI67**, **MMP14**, **NCOA1**, Nitric oxide, Nos (unspecific), **NOS2**, **NPY**, NRP1, **PDPN**, PGE-2, PGF2α, **PHEX**, **PIEZO1**, **PIEZO2**, **PKD1**, **PKD2**, **PPARG**, **PTGER2**, **PTGS1**, **PTGS2**, PTHLH, ratio (BCL2/BAX), ratio (RANKL/OPG), **RUNX2**, SFRP1, **SFRP2**, **SFRP4**, **SOST**, **SP7**, **SPP1**, STAT3, **TGFB1**, **TJP1**, **TNF**, **TNFRSF11B**, **TNFSF11**, **TUBA1A**, **VCL**, **VEGFA**, **WNT1**, **WNT3A**, **WNT4**, **WNT5A**, ZIC1

A total of 22 genes or proteins and metabolites were investigated by at least three studies in relation to FSS in the five included cell groups (Figure 4, Supplementary Data Sheet S6). These include (gene/metabolite; cell type): alkaline phosphatase (ALP/ALPL/ALPP; hMSCs, hPDLCs); BGLAP (osteocalcin; hMSCs, mOcyt); BMP2 (bone morphogenetic protein 2; hMSCs); calcium (Ca^2+^; hMSCs, mOcyt); COL1A1 (collagen 1A1; hMSCs); MAPK1/MAPK3 (mitogen-activated protein kinase 1/3, aka ERK1/2; hMSCs, mOst, mOcyt); NO (nitric oxide; hMSCs, hOst, mOst, mOcyt); PGE-2 (prostaglandin E2; hOst, mOst, mOcyt); PGI-2 (prostacyclin/prostaglandin I2; hOst); PTGS1 (prostaglandin-endoperoxide synthase 1; hOst); PTGS2 (prostaglandin-endoperoxide synthase 2; hMSCs, hOst, mOst, mOcyt); RUNX2 (Runt-related transcription factor 2; hMSCs, mOcyt); SPP1 (secreted phosphoprotein 1; hMSCs, hOst, mOst, mOcyt); VEGFA (vascular endothelial growth factor A; hMSCs, mOcyt). Additionally, in mouse osteocytes the following loci were analyzed: AKT1 (AKT serine/threonine kinase 1), CTNNB1 (β1-catenin), GJA1 (gap junction protein α1), PDPN (podoplanin), SOST (sclerostin), TNFRSF11B (TNF receptor superfamily member 11b), TNFSF11 (tumor necrosis factor superfamily member 11), and WNT3A (Wnt family member 3A).

#### 3.7.1 Human cells

Four to seven studies reported an FSS-related increase of Ca^2+^, VEGFA and MAPK1/MAPK3 in the hMSCs cell group, and nitric oxide and PGE-2 in the hOst cell group. A complete upregulation has been reported of nitric oxide and COL1A1 in the hMSCs cell group, PGI-2, PTGS1 and PTGS2 in the hOst cell group and ALP/ALPL/ALPP in the hPDLCs cell group by three studies each. While the reported upregulation percentages were 83% for ALP, 86% for PTGS2, 67% for BMP2 and 67% for SPP1 in the hMSCs cell group, BGLAP and RUNX2 were the least upregulated genes in response to FSS at 57% and 60%, respectively.

#### 3.7.2 Mouse cells

At least twelve studies reported prevalent upregulation of Ca^2+^, PGE-2 and PTGS2 in the mOcyt cell group in response to FSS. More than three studies reported dominant upregulation in nitric oxide, PTGS2 and PGE-2 in the mOst cell group. FSS induced upregulation of nitric oxide, BGLAP, VEGFA, RUNX2, SPP1, MAPK1/MAPK3, AKT1, PDPN, CTNNB1, GJA1, TNFRSF11B and WNT3A by at least 65% in the mOcyt cell group. Nevertheless, TNFSF11 and SOST were the least expressed genes in the mOcyt cell group at 64% and 25%, respectively.

### 3.8 Over-representation and network analysis

Over-representation analysis (ORA) and consecutive network analysis were done as described (Figure 2B). ORA was applied to gene lists compiled from studies that applied FSS to human (hMSCs, hOst, hPDLCs) and mouse (mOst, mOcyt) cells. Altogether, 80 of 108 genes (∼74.1%) from the human and 76 of 111 genes (∼68.5%) from the mouse list (Table 5) were identified as differentially expressed genes (DEGs) as defined above.

#### 3.8.1 PPI networks

**FIGURE 5:**
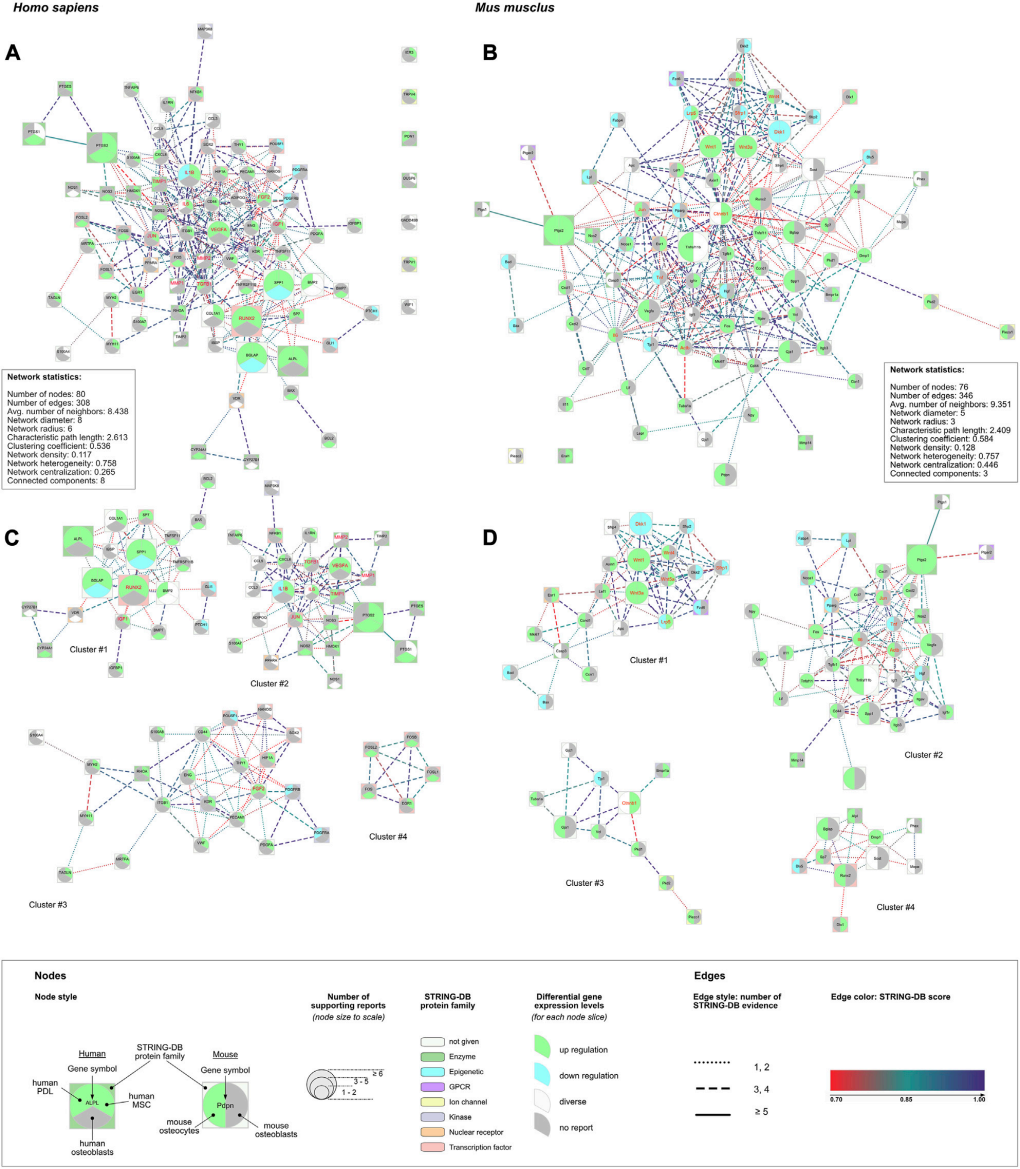
Human (left column) and mouse (right column) protein-protein interaction (PPI) networks were created using lists of DEGs (Table 5). **(A, B)** For each species PPI networks and their basic network statistics were created using the stringApp and Cytoscape. **(C, D)** Identified sub-networks identified from the complete networks shown above **(A, B)** (Table 6, Supplementary Data Sheet S7). The legend applies to all four networks: the node size corresponds to the number of reports identified for the corresponding underlying gene. The edges’ line style depicts the number of STRING sources for the given connection. Additionally, hub genes identified with cytoHubba (Chin et al., 2014) were colored red (Table 6). Pathway analysis was applied to each of the complete networks **(A, B)** and to each of the subnetworks **(C, D)** (Supplementary Data Sheet S7).

From both gene lists protein-protein interaction (PPI) networks were generated. The network statistics for both networks were calculated (Figure 5). The human network consisted of 73/80 DEGs; 7 DEGs were placed outside the PPI network (Table 6; Figure 5). The mouse PPI network included 74/76 DEGs; 2 DEGs were placed outside (Table 6; Figure 5). Most of the human DEGs were reported to be upregulated after FSS application in most of the cell types (e.g., VEGFA, PTGS2, ALPL, RUNX2, FGF2, TIMP1). Only five genes (PUSF1, PDGFRA, PDGFRB, PTCH1, GLI1) were reported to be downregulated. SPP1, BGLAP and IL1B were the only three extracted DEGs, that showed a cell-type specific, contrasting regulation pattern of either up- or downregulation. Overall, a similar pattern was observed in the network related to mouse DEGs. Most of the genetic loci were upregulated in either of both cell types; PTGS2, WNT1, WNT3A, and TNFSF11 were upregulated in both cell types. Contrasting regulation was found for LRP5 (mOst: upregulation; mOcyt: downregulation), whereas only DKK1 was downregulated in both mouse cell types after FSS application. Other genes (DKK2, FZD6, SFRP1, SFRP2, DLX5, HGF, TNF, TJP1, LPL, FABP4, BAD, BAX) were downregulated in either mOst or mOcyt.

**TABLE 6:** Differential expressed genes (DEGs) from the human and mouse gene lists, their contributions to the respective protein-protein interaction networks and the “*GLay community*” clusters. Identified hub genes were underlined. The corresponding networks and clusters were depicted in Figure 5.

Gene list	Genes (n) in STRING network	Cluster number	Number of genes (n) and genes in cluster		Cluster score
*H.s*	Genes in the *H.s*. PPI network (73): ADIPOQ, ALPL, BAX, BCL2, BGLAP, BMP2, BMP7, CCL3, CCL5, CD44, COL1A1, CXCL8, CYP24A1, CYP27B1, EGR1, ENG, FGF2, FOS, FOSB, FOSL1, FOSL2, GLI1, HIF1A, HMOX1, IBSP, IGF1, IGFBP1, IL1B, IL1RN, IL6, ITGB1, JUN, KDR, MAP3K8, MMP1, MMP2, MRTFA, MYH11, MYH2, NANOG, NFKB1, NOS1, NOS2, NOS3, PDGFA, PDGFRA, PDGFRB, PECAM1, POU5F1, PPARA, PTCH1, PTGES, PTGS1, PTGS2, RHOA, RUNX2, S100A4, S100A7, S100A8, SOX2, SP7, SPP1, TAGLN, TGFB1, THY1, TIMP1, TIMP2, TNFAIP6, TNFRSF11B, TNFSF11, VDR, VEGFA, VWF Genes outside the *H.s.* PPI network (7): DUSP6, GADD45B, IER3, PON1, TRPV1, TRPV4, WIF1 Hub genes (cut-off: SUMscore ≥4596.6; n = 11): VEGFA, IL6, FGF2, IL1B, MMP2, TGFB1, MMP1, IGF1, TIMP1, RUNX2, JUN	1	(20)	ALPL, BAX, BCL2, BGLAP, BMP2, BMP7, COL1A1, CYP24A1, CYP27B1, GLI1, IBSP, IGF1, IGFBP1, PTCH1, RUNX2, SP7, SPP1, TNFRSF11B, TNFSF11, VDR	0.191
		2	(26)	ADIPOQ, CCL3, CCL5, CXCL8, HMOX1, IL1B, IL1RN, IL6, JUN, MAP3K8, MMP1, MMP2, NFKB1, NOS1, NOS2, NOS3, PPARA, PTGES, PTGS1, PTGS2, S100A7, TGFB1, TIMP1, TIMP2, TNFAIP6, VEGFA	0.239
		3	(22)	CD44, ENG, FGF2, HIF1A, ITGB1, KDR, MRTFA, MYH11, MYH2, NANOG, PDGFA, PDGFRA, PDGFRB, PECAM1, POU5F1, RHOA, S100A4, S100A8, SOX2, TAGLN, THY1, VWF	0.211
		4	(5)	EGR1, FOS, FOSB, FOSL1, FOSL2	0.044
*M.m*	Genes in the *M.m*. PPI network (74): ACTB, ALPL, APC, AXIN1, BAD, BAX, BGLAP, BMPR1A, CASP3, CCL7, CCNA2, CCND1, CD44, CTNNB1, CXCL1, CXCL2, DKK1, DKK2, DLX1, DLX5, DMP1, ESR1, FABP4, FOS, FZD6, GJA1, GJC1, HGF, IGF1, IGF1R, IL11, IL6, ITGAV, ITGB3, JUN, LEF1, LEPR, LIF, LPL, LRP5, MEPE, MKI67, MMP14, NCOA1, NOS2, NPY, PDPN, PHEX, PIEZO1, PKD1, PKD2, PPARG, PTGER2, PTGS1, PTGS2, RUNX2, SFRP1, SFRP2, SFRP4, SOST, SP7, SPP1, TGFB1, TJP1, TNF, TNFRSF11B, TNFSF11, TUBA1A, VCL, VEGFA, WNT1, WNT3A, WNT4, WNT5A Genes outside the *M.m*. PPI network (2): ERAL1, PIEZO2 Hub genes (cut-off: SUMscore ≥12,622.9; n = 12): CTNNB1, WNT1, WNT3A, WNT5A, DKK1, WNT4, LRP5, SFRP1, IL6, TNF, ACTB, JUN	1	(21)	APC, Axin1, BAD, BAX, CASP3, CCN1, CCND1, DKK1, DKK2, ESR1, FZD6, LEF1, LRP5, MKI67, SFRP1, SFRP2, SFRP4, WNT1, WNT3A, WNT4, WNT5A	0.223
		2	(33)	ACTB, CCL7, CD44, CXCL1, CXCL2, FABP4, FOS, HGF, IGF1, IGF1R, IL11, IL6, ITGAV, ITGB3, JUN, LEPR, LIF, LPL, MMP14, NCOA1, NOS2, NPY, PDPN, PPARG, PTGER2, PTGS1, PTGS2, SPP1, TGFB1, TNF, TNFRSF11B, TNFSF11, VEGFA	0.25
		3	(10)	BMPR1A, CTNNB1, GJA1, GJC1, PIEZO1, PKD1, PKD2, TJP1, TUBA1A, VCL	0.06
		4	(10)	ALPL, BGLAP, DLX1, DLX5, DMP1, MEPE, PHEX, RUNX2, SOST, SP7	0.088

Eleven hub genes were identified in the human network, and twelve in the mouse network (Figure 5; Table 6, Supplementary Data Sheet S7). In each network, four highly connected gene clusters were identified (Figure 5; Table 6, Supplementary Data Sheet S7). The smallest one (cluster #4 from the human network) contained 5 genes from the human DEG list. Cluster #2 identified in the mouse network was the largest one, compromising 33 genes from the mouse DEG list.

#### 3.8.2 Over-representation analysis (ORA)

ORA was applied to identify biological processes and signaling pathways involved in regulation of gene expression after FSS application as described. For each database, the top 15 enriched terms from both DEG lists were ranked according to false discovery rate (FDR) and reported (Supplementary Data Sheet S7). ORA was also applied to all clusters separately using the same cut-offs (Supplementary Data Sheet S7).

*ORA of the human DEG list* (Supplementary Data Sheet S7, section 7.2). Most enriched terms were related to cytoskeleton/extracellular matrix reorganization (GO:0030335, GO0030198, hsa04510, WP306, WP3932) and ossification (GO:0001503, GO:0001649, GO:0030278, WP322, WP474, WP4808). Signaling pathways related to ERK1/ERK2 (GO:0070372, GO:0070374), MAPK (hsa04010), EGFR (hsa01521), Toll-like receptor-related signaling (hsa04620, WP75), and HIF-1 signaling (hsa04066) were also identified. Community detection revealed 4 clusters compromising altogether 73 DEGs. Cluster-specific ORA revealed that cluster #1 contained genes participating in ossification, or more generally in development/differentiation of mineralized tissue including bone and tooth. Being the largest cluster, cluster #2 contained genes relevant to the proliferation and migration of smooth muscle cells and other cell types, but also included pathways related to inflammation (i.e., rheumatoid arthritis, IL18, IL17 and IL1R signaling pathways), and genes from the TNF- and HIF1-signaling pathways. Though cluster #3 contained 22 DEGs, only 4 to 7 of these were assigned to enrichment terms. The terms with ≥5 DEGs assigned were “*cardiac progenitor differentiation*” (7 DEGs, WP2406), “*endothelial cell migration*” (5 DEGs; GO:0043542), and “*EGFR tyrosine kinase inhibitor resistance*” (5 DEGs; hsa01521 and WP4806). Since the smallest cluster (cluster #4) only contained 5 DEGs, a clear attribution to one of the three databases (GO/Biological Process, KEGG, WikiPathways) was not achieved. The 11 identified hub genes were mostly related to clusters #1 (IGF1, RUNX2) and #2 (IL1B, IL6, JUN, MMP1, MMP2, TGFB1, TIMP1, VEGFA). FGF2 was the only hub gene in cluster #3, and cluster #4 contained none.

*ORA of the mouse DEG list* (Supplementary Data Sheet S7, section 7.3). Enriched terms from GO/Biological Process were mostly related to organ or tissue development (GO:0048732, GO:2000027, GO:0050678, GO:0061138, GO:0060562, *etc.*), ossification (GO:0030278, GO:0001503) and WNT-related signaling (GO:0016055, GO:0060070). Ossification and WNT-related signaling were also identified with KEGG and WikiPathways. Additional pathways were identified related to HIPPO- (mmu04390) and TNF-signaling (mmu04668), and focal adhesion (WP85, mmu04510). Community detection revealed 4 clusters compromising altogether 74 DEGs. The first cluster contained 21 DEGs, that were mainly assigned to WNT and BMP signaling, but also to general developmental processes (i.e., embryonic or dermatome development) and HIPPO signaling. In all enrichment terms, at least one of the genes of the WNT family (WNT1, WNT3A, WNT4, WNT5A) was listed. Cluster #2, consisting of 33 DEGs, contained enrichment terms describing cell proliferation and/or migration of different cell types (i.e., bone, smooth muscle cells, leukocytes). Other enrichment terms were related to bone remodeling processes (bone remodeling, bone resorption, endochondral ossification, osteoclast signaling). Clusters #3 and #4 each contained 10 DEGs. With the DEGs contained in cluster #3 none of the enrichment terms satisfied the applied criteria (min of 4 DEGs from the list). In cluster #4, only the enrichment term “*biomineral tissue development*” (GO:0031214) satisfied the applied criteria. The 12 identified hub genes were mostly related to clusters #1 (DKK1, LRP5, SFRP1, WNT1, WNT3A, WNT4, WNT5A) and #2 (ACTB, IL6, JUN, TNF), whereas one hub gene was found in cluster #3 (CTNNB1).

### 3.9 High-throughput gene expression studies and gene set enrichment analysis (GSEA)

Five different human and mouse high-throughput gene expression studies were identified with raw data available in public repositories as described (Figure 2B). In two studies, FSS was applied to human bone marrow-derived mesenchymal stem cells (hMSCs). The effect of FSS on the mouse osteocyte cell line MLO-Y4 was analyzed in the other three studies. In this section, we will only focus on those clusters that were shared by either both (hMSC group) or at least two (MLO-Y4 group) GSEA results.

**FIGURE 6:**
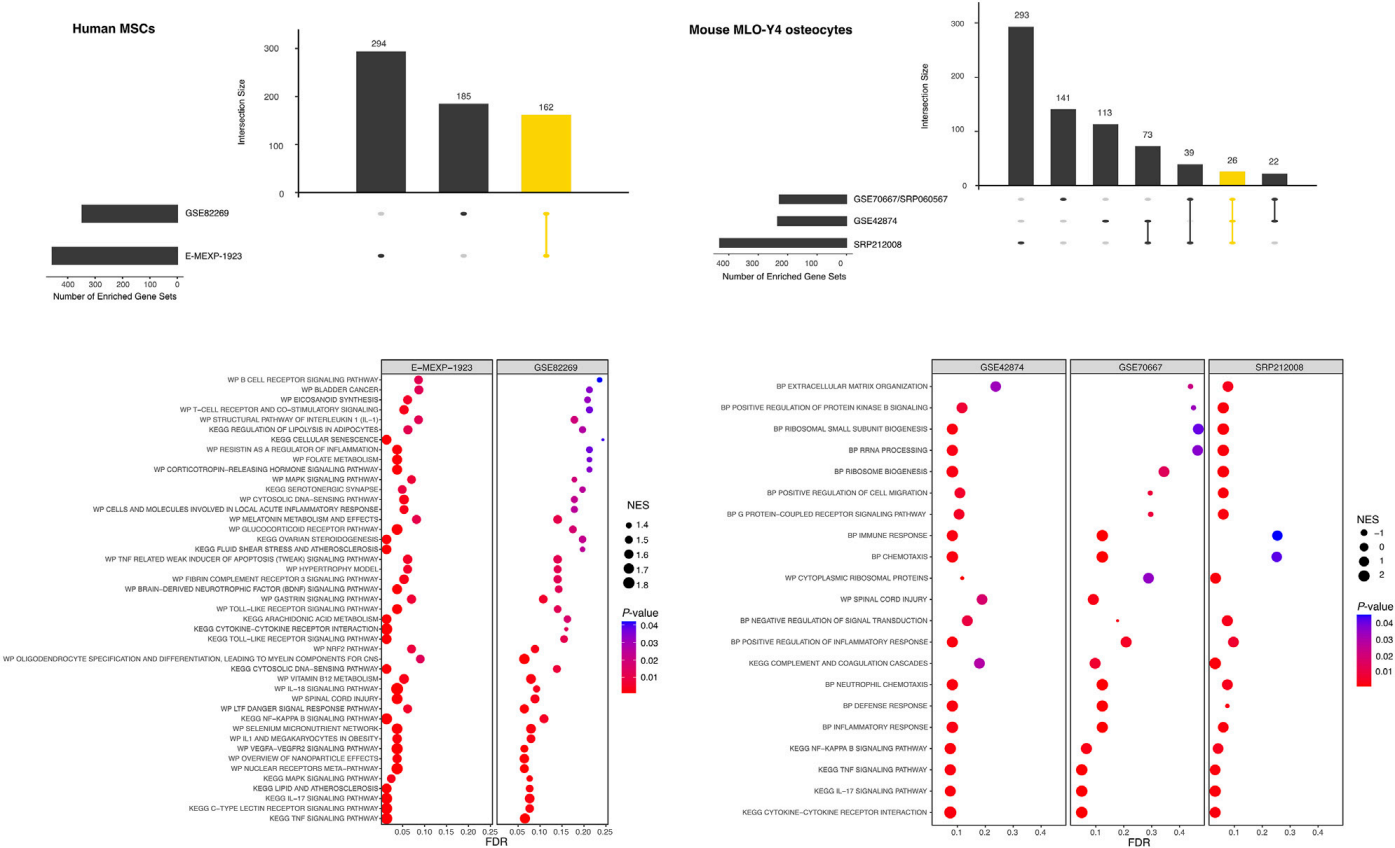
Pre-ranked gene set enrichment analysis (GSEA) of high-throughput gene expression studies investigating the effect of FSS on human mesenchymal stem cells (human MSCs, left column) or mouse MLO-Y4 osteocytes (right column). Differential gene expression was evaluated for each study separately. Ranked gene lists were generated and used for GSEA using easyGSEA; for each cell type the GSEA results were compared with easyVizR (Cheng et al., 2021) and the shared gene sets were further analyzed (yellow column in each of the upset-plots). The shared gene sets with *p* < 0.05, and FDR<0.1 in any of the individual studies were retained. The top 50 gene sets from hMSCs and all gene sets from mouse MLO-Y4 were visualized using dot plots. NES, normalized enrichment score; FDR, false discovery rate.

*Human mesenchymal stem cells (hMSCs)* (Figure 6; Supplementary Data Sheet S8, section 8.4). Both studies shared numerous pathways that were significantly enriched. These were related to inflammation (e.g., WP2865, WP4493, WP4481, HSA04657, WP4754, WP2637), but also to TNF- (HSA04668, WP 2036, HSA04668), MAPK- (WP382, HSA04010), and Toll-like receptor signaling (WP75, HSA04620). Several pathways related to metabolism (e.g., vitamin B12, WP1533; folate, WP176; arachidonic acid, HSA00590) were also significantly enriched, as well as the cytosolic DNA-sensing pathway (WP4655, HSA04623).

*Mouse osteocyte cell line MLO-Y4* (Figure 6; Supplementary Data Sheet S8, section 8.5). The easyVizR comparison of the results obtained from easyGSEA revealed, that at least two of the three studies shared pathway terms that were significantly enriched. Terms that were found to be shared between two studies were related to ribosomal RNA processing (GO:0006364) or more generally to ribosome biogenesis (GO:0042254, GO:0042274), but also to inflammatory response (GO:0006954, GO:0050729), or chemotaxis (GO:0006935, GO:0030593). Terms enriched in all three studies were related to the NF-KappaB- (MMU04064), TNF- (MMU04668), IL17-signaling (MMU04657) and cytokine-cytokine receptor interaction (MMU04060).

## 4 Discussion

The purpose of this systematic review was to identify studies that investigated the effect of fluid flow shear stress (FSS) on different cell types involved in bone remodeling, to analyze their molecular findings and to summarize commonly used fluid flow apparatuses, FSS magnitudes, durations and mechanobiological responses such as gene expression and metabolites release using protein-protein interaction (PPI) networks and enrichment analysis. Defined criteria were used to evaluate the risk of bias of each study using tailored clear definitions.

### 4.1 Commonly used apparatuses to apply FSS

Generally, a fluid flow device is considered a system that consists of a fluid-flow generating part (e.g., cone and plate, orbital shaker, rocking plate, peristaltic/gear/syringe/osmotic pump with or without gravity support), a fluid flow chamber, a pulse dampener, and other auxiliary parts such as bubble trap, digital bubble, or flow sensor. For *in vitro* fluid flow experiments, FSS apparatus selection may be influenced by the pattern of fluid flow, the fluid generating part, or the downstream analytics. For instance, different flow patterns may require different flow driving forces that necessitates a specific kind of pump or use a modified fluid flow system to convert one flow profile into another flow profile. The use of a pulse dampener will convert a pulsating fluid flow pattern into a steady laminar fluid flow pattern. Methods applied for downstream analysis may also play a role in selecting an appropriately sized fluid flow chamber for a given experiment. Chambers used in microscopy tend to be smaller and more delicate compared to larger parallel flow chambers or rocking and orbital shaking platforms. These provide a larger surface for cell monolayer culturing yet yield higher output of mechanically stimulated samples for further analysis. However, smaller parallel flow chambers are capable of exerting uniform and higher FSS magnitudes compared to rocking and orbital shaking platforms, which generate lower but non-uniform (variable) FSS magnitudes (Zhou et al., 2010).

Due to the complexity and unicity of each of the apparatus systems used in each experiment, our identification strategy depended firstly on the kind of flow chamber used. The other parts of the fluid flow apparatus controlling the fluid flow pattern such as the type of driving force (pump or/and gravity), the use of pulse dampener and the general design of the system as defined above were considered secondary.

The most frequently used fluid flow systems from the identified studies consisted of either custom-made or commercially available fluid flow chambers (Flexcell, PeCon, Fluxion Bioscience, Focht or Cytodyne). In both cases, a commercially available driving part (e.g., peristaltic/syringe pump) was used. According to our review, self-designed fluid flow chambers were the most frequently used experimental setup to deliver either of the three flow types (steady laminar, pulsatile laminar and oscillatory laminar fluid flow) independent of the cell type. The possible reason behind this might be that self-designed fluid flow chambers can be customized to serve the required experimental purpose such as geometry, cell culturing area, and/or fluid flow type (Sonam et al., 2016). For primary mouse and human osteoblasts, the Cytodyne fluid flow chamber was the second most frequently used to deliver steady laminar and pulsatile laminar FSS. Chambers from Flexcell Inc. Were the second most preferred for mOcyt and hMSCs to deliver all three fluid flow patterns. Moreover, the three fluid flow systems used for microscopy (Bioflux system, Focht Chamber System 2, and PeCon parallel plate system) were used to deliver either steady laminar, pulsatile laminar or oscillatory laminar fluid flow, respectively. Nevertheless, additional information regarding the Cytodyne fluid flow system and the PeCon parallel plate chamber were not identified.

Finally, the fluid flow profile applied should be defined more precisely. Using the term “*laminar*” to describe the fluid flow profile only can be confusing since three different fluid flow patterns (Figure 1; “*steady laminar*”, “*pulsatile laminar*” and “*oscillatory laminar*”) are considered in biomechanics. The term “*laminar*” describes that the flow is smooth with layers or lamina, and it can be adjusted to either steady or unsteady. The impact of the fluid flow patterns on cellular expression will be discussed in the next section.

### 4.2 FSS magnitudes and durations

Independent of the cell type, in any included study either of the three fluid flow types (Figure 1) was applied at different FSS magnitudes. This selection was based on the study’s aim and the required *in vitro* parameters like cell type, FSS magnitude and type of fluid flow to mimic an actual clinical or physiological situation, or to investigate the effect of FSS on gene expression. The application of these parameters in *vitro* simulations facilitates a more physiologically relevant *in vitro* setup and thus a robust analysis. In this systematic review, we found that the FSS parameters were chosen according to.

1) *Theoretical estimation of the physiological FSS in the canaliculi-lacunae network in bone tissue and blood vessels.* These studies have calculated physiological levels of fluid flow rate depending on the porous geometry of tissue, poro-elasticity and fluid properties using mathematical models (Table 7). Different levels of shear stress acting on the various cell types have been identified and were attributed to the differences in their micro-geometry (blood vessels, bones, muscles, ligament) (Table 7) and differences in the circulating fluids (e.g., blood *versus* serum) (Weinbaum et al., 1994; Zeng et al., 1994; Davies, 1995; Chen et al., 1998; Wittkowske et al., 2016). The average wall shear stress in large arteries with uniform geometry ranges between 20 and 40 dyn/cm^2^ (Davies, 1995). Other studies have estimated the interstitial FSS in the tunica media of an artery to ∼1 dyn/cm^2^ (Tada and Tarbell, 2000; Wang et al., 2007). On the bone level, a wall shear stress of 8–30 dyn/cm^2^ in lacunar-canalicular canals of a trabecular bone model was calculated (Weinbaum et al., 1994), whereas in the canaliculi of an osteon it ranged between 6 and 30 dyn/cm^2^ (Zeng et al., 1994). However, while these studies did not take into consideration other bone cell types such as osteoblasts and osteoclasts, giving that they occur at sites with larger porosities (surface of newly formed or removed bone), it was suggested that these cells may encounter shear stresses less than 8 dyn/cm^2^ (Wittkowske et al., 2016). Though fluid flow in tissues of defined geometry (e.g., blood vessels, bone lacunar canaliculi) are easier to predict mathematically, complex tissue geometry (e.g., ligaments, tendons) may require computational finite element analysis to estimate the true mechanical effect of fluid flow within these tissues.
2) *Finite element analysis (FEA) represents an important method to simulate mechanobiological interaction using 3D-models or pre-existing measurements to build accurate biological geometry.* Recently, a model of the lacunar-canalicular space from rat tibiae was obtained using confocal microscopy and 3D reconstruction (Verbruggen et al., 2014). An interstitial FSS of ∼110 dyn/cm2 in the osteocytes’ mechanical microenvironment after simulated vigorous physical activity was calculated using FEA. This approach has also been used to develop a model of fibrillar arrays in ligaments and tendons and a maximum FSS of 12.1 dyn/cm^2^ within these tissues was estimated (Chen et al., 1998) (Table 7).
3) *Ex vivo studies.* Based on an *ex vivo* model of murine tibia and mathematical modelling, it was shown that osteocytes elastically deform due to the application of a physiological shear stress of ∼50 dyn/cm^2^ (Price et al., 2011). Advances in microscopic life imaging enabled to determine the effect of FSS on osteocytes *ex vivo* (Verbruggen et al., 2015) or *in vivo* (Lewis et al., 2017; Lewis et al., 2021) in murine models. The bone was mechanically loaded using a 3-point-bending setup. Either strain distribution and deformation of osteocytes was determined (Verbruggen et al., 2015) or the increases in calcium signaling in osteocytes directly resulting from FSS (Lewis et al., 2017; Lewis et al., 2021) have been measured giving direct evidence of FSS effects on bone cell activity.
4) *In vitro studies* have been applied to study the sensitivity of different cell types including endothelial cells and osteocytes. Similar to endothelial cells *in vitro*, osteocytes respond to magnitudes of shear stress as low as 6 dyn/cm^2^ (Klein-Nulend et al., 1995b). This confirms the theoretical estimation of FSS in the canaliculi of osteons (Zeng et al., 1994). In addition, a positive correlation between FSS strength and bone cellular response has been suggested (Rath et al., 2010; Li et al., 2012).
5) *Fluid flow profiles* in human tissues have been an ongoing research topic due to their relationship to normal and abnormal physiological activity. Using a simple representation of the walking cyle, a reversed direction of fluid flow in bone canaliculi during relaxation and compression was predicted (Piekarski and Munro, 1977). These authors also highlighted the change in hydrostatic pressure within the lacunar-canalicular system during loading and unloading. This change of pattern within human tissues governs the fluid flow profile of fluid movement, e.g., a oscillatory fluid flow profile correlates with the normal cyclic activity of the skeletal system such as walking and running (Lu et al., 2012). In contrast, the change in human posture from sitting to standing would result in a physiological but unusual unidirectional steady fluid flow (Jacobs et al., 1998). In periodontal ligament (PDL), compressive force application leads to an increase of pore pressure and an outflow of interstitial fluid from the PDL porosities, whereas the inflow of interstitial fluid in tension areas resulted from a decrease in pore pressure (Ashrafi et al., 2020). Therefore, a change in interstitial flow direction in these tissues during cyclic loading and unloading is closely resembling the oscillatory fluid flow pattern.

**TABLE 7:** A summary of estimated fluid flow shear stress in different fluid contained porous tissues.

	Porous tissue					
	Blood vessels	Bone	Bone	Bone	Ligaments and tendon	Periodontal ligament (PDFL)
Cell type	Endothelial cells	Osteocytes (trabecular bone model lacunar canaliculi)	Osteocytes (osteon canaliculi model)	Osteoblasts	Fibroblasts	PDL cells
References	Davies (1995)	Weinbaum et al. (1994)	Zeng et al. (1994)	Wittkowske et al. (2016)	Chen et al. (1998)	Unknown
Wall shear stress	20–40 dyn/cm^2^	8–30 dyn/cm^2^	6–30 dyn/cm^2^	<8 dyn/cm^2^	12.1 dyn/cm^2^ (max. Shear stress)	Unknown
Reasoning	Blood is directly pumped by heart	The osteocytic processes are extended in tiny channels surrounded by calcified bone matrix (canalicular walls)	The osteocytic processes are extended in tiny channels surrounded by calcified bone matrix (canalicular walls)	These cells are located at sites of big porosities on the surface of newly formed bone	n.g	Unknown
Medium	Blood	Interstitial fluid	Interstitial fluid	Interstitial fluid	Interstitial fluid	Interstitial fluid
Type of flow	Big blood vessels: pulsatile laminar Small blood vessels: steady laminar	Constant loading: steady laminar Intermittent loading: oscillatory laminar	Constant loading: steady laminar Intermittent loading: oscillatory laminar	Constant loading: steady laminar Intermittent loading: oscillatory laminar	Constant loading: steady laminar Intermittent loading: oscillatory laminar	Constant loading: steady laminar Intermittent loading: oscillatory laminar
Note	Was estimated	Was estimated ** physiological frequency for loading and unloading in long bone that correspond with oscillatory fluid flow is 1–20 Hz Salvi et al. (2010)	Was estimated	Difficult to estimate the FSS values: Constant remodeling Unknown mechanical properties of soft osteoid	Was estimated	

Evidence on physiological fluid flow profiles within human tissue is important to understand the cellular biophysical environment. Oscillatory fluid flow patterns have been investigated by *in vitro* studies as being the most likely fluid flow profile experienced by bone cells *in vivo* (Jacobs et al., 1998; Ponik et al., 2007). Though several studies emphasized the importance of oscillatory flow in maintaining bone integrity (Genetos et al., 2007; Ponik et al., 2007; You et al., 2008; Li et al., 2012), there is also evidence, that the oscillatory fluid flow profile is less stimulatory to bone cells than steady laminar or pulsatile fluid flow profiles (Jacobs et al., 1998). Moreover, bone cell adaptation to common mechanical stress makes them more sensitive to excessive mechanical stress patterns (Turner et al., 2002; Lu et al., 2012). Thus, new FSS magnitudes or profiles are more stimulating to osteocytes than regular FSS magnitudes/profiles.

Herein, oscillatory, and pulsatile fluid flow were the most frequently applied fluid flow profiles in hOst, mOst, mOcyt, and hMSCs, whereas steady laminar fluid flow was mostly applied to hPDLCs. As pointed out, the selection of a fluid flow profile is dependent on the experimental setting, especially the presence of dynamic loading and unloading. In this context it should be mentioned, that the application of static loading to a tissue (e.g., the periodontal ligament), may result in a fluid flow profile that differs from applying cyclic loading and unloading commonly seen in daily human activity such as walking or food chewing (Ashrafi et al., 2020). Based on the in-/outflow of interstitial fluid during compression- or tension-related tissue deformation (Figure 1), it is suggested that the periodontium is subjected to a prominent one-directional dynamic fluid flow during the initial phase of orthodontic tooth movement (OTM) (Ashrafi et al., 2020), which becomes less dominant during the lag-phase of OTM (Reitan, 1960). The duration of the lag-phase is determined by the time required for periodontal remodeling and consequently determines the loading/unloading frequency on tissues, thus affecting fluid flow character (Reitan, 1960).

Independent of the studied cell type, the most frequently selected durations of FSS exposure were 0.5, 1 and 2 h. The main reasoning for this selection was based on the following criteria: (i) optimal time frame to determine cellular biological responses; (ii) technical issues. For instance, from a study on oscillatory fluid flow profile it was concluded that FSS durations of 1, 2, and 4 h resulted in an upregulation of PTGS2, TNFSF11, and TNFRSF11B gene expression favoring bone formation (Li et al., 2012). These authors also pointed out, that longer FSS applications than 4 h might require a different pH buffering system of the cell culture medium without negatively influencing the mechanical properties of the chamber. This and other drawbacks of parallel flow chambers (Huesa et al., 2010), like the formation of air bubbles that may impair the cellular *in vitro* environment (Anderson et al., 2006), or the negative impact of continuous cellular growth and matrix deposition on flow chamber geometry (Nauman et al., 2001) should be taken into consideration.

Intriguingly, the most used shear stress was 10 dyn/cm^2^ regardless of FSS duration and fluid flow profile, which corresponds with the physiological shear stresses (Weinbaum et al., 1994; Zeng et al., 1994). In addition, frequencies of 1 Hz and 5 Hz, which were commonly used in most cell groups, lie within the physiological range of bone loading and unloading caused by locomotion or maintaining posture (Weinbaum et al., 1994).

Finally, it is important to point out that the presence of endothelial cells, bone cells, mesenchymal stem cells and fibroblasts in proximity to each other may facilitate intercellular communication through body fluids including interstitial fluid (Cenaj et al., 2021).

### 4.3 Genes or metabolites reported by at least three studies

Herein, we identified genes and metabolites (AKT1, alkaline phosphatase, BGLAP, BMP2, Ca^2+^, COL1A1, CTNNB1, GJA1, MAPK1/MAPK3, nitric oxide, PDPN, PGE-2, PGI-2, PTGS1, PTGS2, RUNX2, SOST, SPP1, TNFRSF11B, TNFSF11, VEGFA, WNT3A), which were investigated by at least three studies on the effect of FSS on cells of human and mouse origin such as MSCs, PDLCs, and bone cells. These genes/metabolites were grouped based on their functional role during periodontal ligament remodeling as being responsible for tissue formation (AKT1, alkaline phosphatase, BGLAP, BMP2, Ca^2+^, COL1A1, CTNNB1, GJA1, MAPK1/MAPK3, PDPN, RUNX2, SPP1, TNFRSF11B, VEGFA, WNT3A), tissue degradation (SOST, TNFSF11) or inflammation (nitric oxide, PGE-2, PGI-2, PTGS1, PTGS2).

#### 4.3.1 Human cells

##### 4.3.1.1 Tissue formation

The periodontium has a complex ultrastructure including periodontal ligament and bone that is created by various cell types (Marchesan et al., 2011). Periodontal cells are involved in the perpetual remodeling of the periodontium thereby interacting with each other. This process is mediated and regulated by numerous genes/metabolites involved in cell differentiation, proliferation and migration, extracellular matrix formation and tissue maturation.

In addition to the regulatory rule of Ca^2+^ during inflammation and its multiple functions in various biological systems, several studies have highlighted the importance of intracellular Ca^2+^ release in hMSCs proliferation and osteogenic differentiation (Riddle et al., 2006; Hu et al., 2017). Our systematic review has identified six studies in which all of them reported an increase of intracellular calcium release by hMSCs after FSS application. This is supported by recent studies, which show direct evidence of FSS effects on bone cell activity, i.e., increased calcium signaling in osteocytes directly resulting from FSS (Lewis et al., 2017; Lewis et al., 2021).

The activity of alkaline phosphatase (ALPL) in mesenchymal cells has been suggested as an indicator of osteogenesis (Lim et al., 2013). Herein six studies were identified that determined the effect of FSS on ALPL using sqPCR, RT-qPCR or ELISA in hMSCs. A large proportion of studies (>80%) reporting upregulation or release of ALPL in hPDLCs and hMSCs suggest that FSS induces osteogenic differentiation in these cells (Lim et al., 2013; Lim et al., 2014; Qi and Zhang, 2014; Tang et al., 2014) (Figure 4; Supplementary Data Sheets S5, 6). In addition, FSS has been found to upregulate COL1A1 in hMSCs, which provides structural support and strength to various tissues and plays a crucial role in bone formation and integrity (Lim et al., 2013).

Several included studies reported FSS-dependent upregulation of VEGFA, MAPK1/MAPK3 and RUNX2. However, while both MAPK1/MAPK3 and RUNX2 are important regulators of osteoblastic proliferation and regulation (Riddle et al., 2006; Kuo et al., 2015), the number of studies reporting MAPK1/MAPK3 upregulation was higher than reports on RUNX2 upregulation in FSS-stimulated hMSCs. Nevertheless, the relevant studies investigating VEGFA have shown 100% upregulation in hMSCs in relation to FSS (Figure 4). This result was expected since VEGFA is not only involved in vascular development and angiogenesis but also in bone healing (Street et al., 2002). Noteworthy, hMSCs were sensitive to very low oscillatory FSS of 0.01 dyn/cm^2^ lasting for up to 3 weeks, which led to an upregulation of genes involved in tissue formation such as ALPL, COL1A1, BGLAP, SPP1, VEGFA, and RUNX2 (Lim et al., 2013).

##### 4.3.1.2 Inflammation

Inflammation has a significant effect on bone remodeling through bone resorption (Epsley et al., 2020). It is also considered as one of the earliest reactions to orthodontic mechanical force on the cellular level (Li et al., 2018). Several molecules and metabolites such as PGE-2 (a derivative of arachidonic acid and product of cyclooxygenase enzymes), nitric oxide (NO; product of nitric oxide synthases, NOS1-3) and Ca^2+^ (intracellular calcium ions) are produced within tissues and play a crucial role in modulating the inflammatory responses. FSS has been suggested to increase the level of NO and PGE-2 in various biological systems such as osteoblasts, osteocytes, and periodontal ligament cells (van der Pauw et al., 2000; Bakker et al., 2013a; Bakker et al., 2013b; van der Meijden et al., 2016; Deepak et al., 2017). The regulation of NO and PGE-2 was suggested to be dependent on Ca^2+^ signaling transduction (Ajubi et al., 1999). Independent on the human cell type, at least three relevant studies reported exclusive upregulation of NO, Ca^2+^, and PGE-2 in response to FSS. The mobilization of these metabolites by bone cells in response to FSS has been found to regulate tissue remodeling through an inflammatory pathway thereby acting as a second messenger (NO, Ca^2+^) or locally as a hormone-like substance (PGE-2) (Klein-Nulend et al., 1995a; Hung et al., 1995; Reich et al., 1997; McAllister and Frangos, 1999; You et al., 2001).

PTGS1 and PTGS2 are two isoforms of the prostaglandin-endoperoxide synthase (PTGS; aka cyclooxygenase, COX) that are upregulated during inflammation. Both genes are upregulated after FSS application in hOst (Joldersma et al., 2001; Bakker et al., 2003). However, the upregulation of PTGS2 has been found to be stronger and persisted up to 24 h compared PTGS1. Though we identified 3/3 (100%) studies reporting an FSS-dependent upregulation of PTGS1 and PTGS2, it has been suggested that PTGS2 is the mechanosensitive form of two PTGS isoforms that regulates the inflammatory response of bone to mechanical loading (Bakker et al., 2003).

#### 4.3.2 Mouse cells

##### 4.3.2.1 Tissue formation

Similarly, several studies on mouse bone cells have shown the FSS-dependent upregulation of genes and metabolites commonly expressed in hMSCs, hOst and hPDLCs. These include the upregulation of NO and BGLAP (100%) by mOst and mOcyt and Ca^2+^ (93%) by mOcyt in response to FSS.

Interestingly, the immortalized mouse osteocyte cell line MLO-Y4 was investigated in a larger number of included studies than primary human osteocytes. This could be related not only to its availability and reproducibility but also to the multiple roles of osteocytes in bone formation, maintenance, and remodeling. At least three of the studies have shown an upregulation (>80%) of AKT1, PDPN, CTNNB1, GJA1, TNRFSF11B, VEGFA and WNT3A expression after FSS (Figure 4). The expression of AKT1 has been considered essential for the functional opening of CX43 hemichannels and the release of central anabolic factors in response to FSS (Riquelme et al., 2021). It was also reported that PGE-2 is essential in promoting gap junction-mediated communication in osteocytes by controlling the up and downregulation of GJA1 (Xia et al., 2010).

TNRFS11B/TNRFS11 and the WNT/β-catenin signaling pathways have been considered to play a key role in regulating bone architecture and bone homeostasis (Bonewald and Johnson, 2008; Macsai et al., 2008; Baron and Kneissel, 2013; Liao et al., 2017; Yan et al., 2018). In addition, the upregulation of PDPN has been regarded as a reflection of the conversion of osteoblasts to osteoid cells or osteocytes (Zhang et al., 2006). Interestingly, mouse MLO-Y4 cells are sensitive even to very small pulsatile FSS of 2 dyn/cm^2^ (Huang et al., 2017). This FSS magnitude upregulated WNT3A expression and increased PGE-2 release (Huang et al., 2017).

##### 4.3.2.2 Tissue degradation

While an increasing number of reports have shown upregulation of the above-mentioned genes in mOcyt, only few studies have reported upregulation of genes that are responsible for bone resorption such as SOST1, TNFSF11 or TNFSF11B (Supplementary Data Sheets S5, 6). Only 25% (1/4) of studies reported a FSS-dependent upregulation of SOST1 in mOcyt leading to a downregulation of the Wnt/β-catenin pathway and thus inhibiting osteogenesis (Li et al., 2013). Variable expression of TNFSF11 or TNFSF11B was observed after application of different FSS profiles. High magnitudes of steady laminar FSS (16 dyn/cm^2^; 30 dyn/cm^2^) were shown to support the upregulation of TNFSF11 or TNFSF11B (Li et al., 2013) in comparison to low magnitudes (10 dyn/cm^2^) (Haugh et al., 2015; Yan et al., 2018). However, pulsatile fluid flow profiles at lower magnitudes (≈10 dyn/cm^2^) upregulated the expression of TNFSF11 or TNFSF11B (Kulkarni et al., 2010; Kulkarni et al., 2012; Bakker et al., 2013b; Liao et al., 2017). Interestingly, only 12.5% (1/8) of the studies using mouse osteocytes have shown an increase in the RANKL/OPG ratio and thus favoring bone resorption (Supplementary Data Sheets S5, 6).

##### 4.3.2.3 Inflammation

In addition to the inflammatory mediators mentioned above for human cells upregulation of PGE-2, PTGS2, and nitric oxide were found in the included studies. All inflammation-related genes and metabolites were solely upregulated by FSS independent of the specific mouse cell type.

### 4.4 General observations

The categorization of the genes/proteins/metabolites used herein (inflammation, tissue formation, tissue degradation) was used as a structural framework to improve data description and analysis thus enhancing the comprehensibility of the complex interactions between the different molecular pathways involved on various levels. Still, the results derived from studies on both human and mouse cells suggest dominant tissue formation character of FSS. However, direct comparison of their results was not feasible. This was primarily due to the heterogeneity which exist in different aspects within and between the studies (Sun et al., 2021), such as: 1) differences related to the cells used (e.g., cell density, cell isolation methods, passaging number, donor-related differences (sex, age, *etc.*), cell culturing materials and cell maintenance conditions); 2) differences related to the applied analytical techniques (quantification methods, reference gene selection, differences in expression reporting, and post-transcriptional modification); 3) differences in the experimental set-up like timings of sample collection, fluid flow profile used, duration of FSS application/post-FSS incubation, FSS magnitude and/or frequency, and data collection and analysis.

The effect of donor age and donor sex on cell physiology is well established. Due to the high heterogeneity of studies along with partially missing reports on donor age and/or sex, these variables were not considered for stratification in data analysis. Nevertheless, age-related changes relevant to FSS application have been identified in the literature and been summarized herein to emphasize their significance for further research. Aging is a physiological process which is inevitably bound to various cellular, mechanical, and spatial changes within tissues, including bone (Brodt et al., 1999). In osteocytes, aging is associated with a marked reduction of trabecular (Qiu et al., 2002) and cortical osteocyte density (Carter et al., 2013a; Carter et al., 2013b). Lacunar canalicular permeability and organization was also found to be negatively affected by aging, especially in females, leading to a disruption in the bone microarchitecture, reduced fluid flow and thus impairs cellular communication between osteocytes (Currey, 1964; Okada et al., 2002; Rodriguez-Florez et al., 2014; Ashique et al., 2017; Schurman et al., 2021).

Also, the periodontal ligament (PDL) undergoes characteristic age-related cellular, structural, and functional changes. As mentioned above, the PDL is located between the tooth and the alveolar bone and consists of cells of different tissue types embedded in a matrix, which is composed mainly of collagen type 1 and 2 (Karimbux et al., 1992; Marchesan et al., 2011). With increasing age a reduction in PDL thickness is commonly observed (Barczyk et al., 2013). Additionally, in aged populations collagen fibers are poorer organized (Cho and Garant, 1984; Kawada and Komatsu, 2000) and cellular collagen expression is reduced (Oehmke et al., 2004). Therefore, aging also affects the mechanical properties of the periodontal ligament, which dependent on collagen. Nevertheless, the periodontal ligament cells may have impaired function with aging. These altered functions are closely related to periodontal ligament fibroblast, which is suggested to produce more inflammatory mediators and yet is more prone to damage (Abiko et al., 1998). Therefore, it is suggested that age-related cellular changes in bone and periodontal ligament might impair the response to treatment at higher age.

The relationship between fluid flow and age were addressed in two *in vitro* studies (Joldersma et al., 2000; Klein-Nulend et al., 2002). It was found that apart from reduced proliferation capacity of aged cells, the mechanosensitivity of these cells to fluid flow remained unaffected. However, these *in vitro* studies did not take into consideration age-related micromechanical (structural) bone changes, which may influence the magnitude of fluid flow within bone and resulting shear stress acting on these cells.

Despite several limitations, we suggest that during orthodontic treatment, interstitial fluid flow occurs within the periodontium at stretching and compression areas around the tooth (Ashrafi et al., 2020). However, stronger bone deposition occurs in tissues subjected to stretching compared to compression (Li et al., 2018). While the vascular supply is reduced in the compression areas, we assume that the effect of interstitial fluid flow, which depends on the blood and lymphatic circulation, remains undisrupted on the tension side (Li et al., 2018). The fluid flow environment and the metabolic similarities between FSS-induced and stretched tissues during OTM remain to be further elucidated in the future.

### 4.5 Over-representation, gene set enrichment and network analysis

Due to the heterogeneity of the included studies, a direct comparison of FSS-dependent gene expression was not possible. In contrast to a recently published systematic review (Sun et al., 2021), five studies with publicly available high-throughput gene expression data (microarray, RNA-seq) were identified reporting FSS-dependent gene expression in hMSCs and mOcyt (MLO-Y4). Therefore, the previously published analytical workflow (Sun et al., 2021) was extended to gain additional insights into the molecular pathways regulated by FSS in OTM-related human and mouse cells. The bipartite workflow consisted of the following steps: (1) generation of protein-protein interaction (PPI) networks and application of over-representation analysis (ORA). (2) Re-analysis of five high-throughput gene expression studies. (3) To increase comparability within and between the workflow arms, identical databases were engaged for enrichment.

#### 4.5.1 PPI networks and over-representation analysis (ORA)

Species-specific lists of FSS-dependent differential expressed genes (DEGs) were extracted from all studies identified herein. Reports from high-throughput expression studies (microarray, RNA-seq, proteomics) were excluded, since only the most significantly up- and downregulated genes were reported, and different cut-offs were applied. Instead, gene expression data derived from RT-qPCR reanalysis was extracted and included. To increase specificity, only those genes showing clear FSS-dependent expression were included (Sun et al., 2021). Due to its methodological heterogeneity protein expression data were excluded (ELISA vs. WB quantification vs. enzyme activity; antibody specificity).

From the human gene list, seven genetic loci were not integrated into the network (IER3, TRPV1, TRPV4, PON1, DVSP6, GADD45B, WIF1), whereas only two genetic loci (ERAL1, PIEZO2) were not integrated into the mouse network. Lowering the STRING-DB cut-off score to 0.4 integrated the first 6 singletons (IER3, TRPV1, TRPV4, PON1, DVSP6, GADD45B) into the human network. All newly established edges originated from text-mining results, and only a few interactions were additionally supported by co-expression or other experimental data. Lowering the cut-off to 0.3 also integrated WIF1 into the network, also due to text-mining results. In the mouse network, decreasing the cut-off score to 0.4 established a PPI-interaction between PIEZO2 and PKD2 of the existing network (text-mining score: 0.45; co-expression score: 0.061). Lowering the cut-off to 0.2 did not integrate ERAL1 into the network but instead established an additional PPI-interaction between PIEZO2 and PKD1 of the existing network (text-mining score: 0.366, co-expression core: 0.062). Generally, a lower cut-off score might integrate singletons into an existing network, but at the same time it might increase the number of PPI interactions between other nodes of the existing network. Though not affecting enrichment directly, the reduction of the cut-off score to 0.3 will affect several network statistics by increasing the total number of edges and the average number of neighbors and decreasing the network diameter and the characteristic path length (not shown). This, in turn, might will affect the number of clusters and hub genes and thus downstream enrichment analysis. With a cut-off score of 0.7, 4 clusters were identified in each network. After application of a cut-off score of 0.3 to both PPI networks, the same clustering algorithms identified 3 clusters in the human and 2 clusters and 1 singleton (Eral1) in the mouse PPI networks (results not shown).

Generally, the findings from the two species-specific networks were consistent with the research topics covered in the included studies (Sun et al., 2021). Enrichment terms related to ossification and cytoskeleton/extracellular matrix reorganization were identified in both species. Since constraints were applied to increase specificity in reporting, the list of enrichment terms might suggest, that terms found in one PPI network were not identified in other networks or its clusters, e.g., BMP-related pathways and biological processes in the mouse PPI network, or ERK1/ERK2-related signaling in the human PPI network. Indeed, these terms were also identified in the other PPI network, but due to the applied selection criteria, these terms did not pass this cut-off. For example, ERK1/ERK2 signaling (GO:0070372) was represented by 18 genes from the human gene list covering 6.2% of the genes belonging to this biological process (FDR: 4.16E-14). ORA identified ERK1/ERK2-related terms also in the mouse list, but none was reported due to either criteria: “*Regulation of erk1 and erk2 cascade*” (GO:0070372; 11 genes, 3.5%, FDR: 3.88E-7), “*Positive regulation of erk1 and erk2 cascade*” (GO:0070374; 8 genes, 4.1%, FDR: 2.19E-6), and “*ERK1 and ERK2 cascade*” (GO:0070371; 3 genes, 9.7%, FDR 2.50E-3). The alike also applied to the “*Hippo signaling pathway*” (mmu04390) identified in the mouse PPI network (13 genes, 8.3%, FDR: 6.32E-13). Pathways related to Hippo signaling were also identified in cluster#3 of the human PPI network but failed to be reported due to the applied constraints: “*Hippo-Merlin signaling dysregulation*” (WP4541; 5 genes, 4.2%, FDR:1.92E-5) and “*H.ippo signaling regulation pathways*” (WP4540; 4 genes, 4.1%, FDR: 2.90E-4).

#### 4.5.2 Selection of high-throughput gene expression studies

The search strategy used herein identified several studies that applied high-throughput expression platforms to study FSS-dependent expression in one of the selected cell types: hMSCs were analyzed with antibody arrays (Lim et al., 2013) or microarrays (Kuo et al., 2015). Microarray platforms were also applied to study hPDLCs (Qi and Zhang, 2014) and mOsts (Lau et al., 2006). Mouse osteocytes were analyzed with microarrays (Govey et al., 2014; Kitase et al., 2014), RNA-seq (Govey et al., 2015; Li X. et al., 2019; Du et al., 2020) and proteomics (Govey et al., 2014). In line with ORA, we focused on gene expression platforms (microarray, RNA-seq) and identified three studies with publicly available raw data (Govey et al., 2014; Govey et al., 2015; Li et al., 2019b). By hand-searching of public repositories two additional studies with available raw data were identified (Glossop and Cartmell, 2009; Diaz et al., 2017).

#### 4.5.3 Pre-ranked GSEA

Several enriched pathways and biological processes were identified as shared in each species, indicating that despite differences in experimental conditions FSS-related gene expression might be similar. Nevertheless, differences were observed concerning enrichment statistics (enrichment score, associated *p* values) and genes belonging to the leading edges.

During the reanalysis of the data sets used for GSEA it became apparent, that each step in DEG analysis will influence the pre-ranked gene lists from each study and thus the subsequent GSEA. Originally, the workflow based on the “limma” Bioconductor package (Ritchie et al., 2015) was considered for both microarray and RNA-seq data. Finally, “limma” was chosen for the microarray and the “DESeq2” Bioconductor package (Love et al., 2014) for RNA-seq data analysis. Additionally, the inclusion of different microarray platforms required adaptations to the originally considered workflows (Love et al., 2015; Klaus and Reisenauer, 2016; Law et al., 2016). Additional steps during the workflow related to normalization, unsupervised filtering of non- or low-expressed probes/genes, model-selection (design-matrix, covariates, blocking factor) and the method to assess differential gene expression will influence the DEG results. Ranking of the gene lists will also influence the results of GSEA (Reimand et al., 2019). Usually, a score is used, that describes the level of differential expression for each gene, e.g., according to the *t-*statistics from the limma-workflow, according to log2FoldChange, or combinations of statistics and log2FoldChange (sign (log2FC)×-log10(*p*-value) or log2FC×-log10(*p*-value)).

The source of enrichment terms (pathways, disease state, transcription factor binding sites, miRNA or lncRNA interaction data, *etc.*) will also affect the outcome of an enrichment analysis, independent if ORA or GSEA is applied. Additionally, though sometimes named (nearly) identical, terms derived from different sources contain different gene sets. As such the KEGG “*Toll-like receptor signaling pathway*” (hsa04620) and WikiPathways’ pathway with the same name (WP75) share 99 genes out of 104 (KEGG) respectively 103 (WikiPathways). Therefore, the application of different enrichment sets and implementations for the actual enrichment is frequently advised in the literature (Reimand et al., 2019). Based on the experience gained during reanalysis, one bottleneck in the described workflow is the availability of resources to compare the data from different sources. For example, easyGSEA results were compared with the online companion program easyVizR (Cheng et al., 2021). For this comparison, information on the leading edge’ genes, the enrichment score, the *p-*value and the FDR or adjusted *p-*value were needed for each enrichment term from each of the studies. As such, a group-wise comparison of the pre-ranked GSEA results generated by STRING-DB (Szklarczyk et al., 2019; Szklarczyk et al., 2023) was not possible, since the unadjusted *p-*value was not given with the results from STRING-DB.

### 4.6 Strengths and limitations

Herein discussed evidence implicates that fluid flow is an important player in bone and periodontal tissue remodeling and especially in formation. To our knowledge, this is the first review giving a comprehensive overview and discussion of methodological technical details regarding fluid flow application in 2D cell culture *in vitro* experimental conditions. It is also providing valuable information about cellular molecular events and their quantitative and qualitative analysis.

Even though, the current analysis was done under an orthodontic point of view, specifically in terms of orthodontically induced bone remodeling, it must be clearly emphasized that most of the data considered herein have been reported from studies not exclusively addressing the orthodontic-related research questions issues. While this might be considered as limitation it is clearly indicating the need for more evidence on cells from the orofacial skeleton. Moreover, it is also providing a profound basis for these studies, concerning both molecular background and technical design.

Moreover, the considerable methodological and biological-related heterogeneity among studies should be considered when interpreting the current results. To increase the number of standardized and reproducible studies in the future there is a clear necessity for the development of an optimized fluid flow model, carefully adapted to the specific research requirements.

Even though this review tried to categorize all studies considering different fluid flow modes and cellular types, not all variables that affect bone remodeling were encompassed. This includes some donor-related characteristics that might affect bone remodeling, such as age and sex. Due to its complexity and importance for tissue remodeling, this topic has yet not sufficiently addressed and might therefore comprise an important subject for future work.

## Figures and Tables

**Table:** 

**AKT1**	AKT serine/threonine kinase 1
**ALP/ALPL/ALPP**	Alkaline phosphatase
**BGLAP**	Bone gamma-carboxyglutamate protein
**BMP2**	Bone morphogenetic protein 2
**Ca2+**	Calcium ion
**COL1A1**	Collagen type 1 alpha 1
**CTNNB1**	Catenin beta 1
**DEG/DEGs**	Differential expressed gene/genes
**ELISA**	Enzyme-linked Immunosorbent Assay
**FEA**	Finite element analysis
**FSS**	Fluid flow shear stress
**GEO**	Gene Expression Omnibus
**GJA1**	Gap junction protein alpha 1
**GO**	GeneOntology
**GSEA**	Gene Set Enrichment Analysis
**hMSCs**	Human mesenchymal stem cells
**hOcyt**	Human osteocytes
**hOst**	Human osteoblasts
**hPDLCs**	Human periodontal ligament cells
**IF**	mmunofluorescence
**KEGG**	Kyoto Encyclopedia of Genes and Genomes
**MAPK1**	Mitogen-Activated Protein Kinase 1
**MAPK3**	Mitogen-Activated Protein Kinase 3
**mOcyt**	Mouse osteocytes
**mOst**	Mouse osteoblasts
**NO**	Nitric oxide
**ORA**	Over Representation Analysis
**OTM**	Orthodontic tooth movement
**PDL**	Periodontal ligament
**PDLCs**	Periodontal ligament cells
**PDPN**	Podoplanin
**PGE-2**	Prostaglandin E2
**PTGS1**	Prostaglandin-endoperoxide synthase 1 (aka cyclooxygenase-1, Cox-1)
**PTGS2**	Prostaglandin-endoperoxide synthase 2 (aka cyclooxygenase-2, Cox-2)
**RoB**	Risk of bias
**RT-qPCR**	Reverse-transcriptase quantitative real-time polymerase chain reaction
**RUNX2**	Runt-related transcription factor 2
**SPP1**	Secreted phosphoprotein 1 (aka osteopontin, OPN)
**sqPCR**	Semiquantitative reverse-transcriptase polymerase chain reaction
**STRING**	Search Tool for Retrieval of Interacting Genes/Proteins
**TNFRSF11B**	TNF receptor superfamily member 11b (aka osteoprotegerin, OPG)
**TNFSF11**	TNF superfamily member 11 (aka Receptor Activator of NF-κB Ligand; RANKL)
**VEGFA**	Vascular endothelial growth factor A
**WB**	Western blotting
**WNT3A**	Wnt family member 3A

## Data Availability

The original contributions presented in the study are included in the article/Supplementary Material, further inquiries can be directed to the corresponding author.

## Footnotes

1
https://www.genenames.org/

2
https://www.informatics.jax.org/genes.shtml

3
https://www.ncbi.nlm.nih.gov/gene/

4
https://www.ncbi.nlm.nih.gov/tools/primer-blast/

6
https://www.ncbi.nlm.nih.gov/geo/

7
https://www.ebi.ac.uk/biostudies/arrayexpress

8
https://www.ncbi.nlm.nih.gov/sra/

9
https://jhubiostatistics.shinyapps.io/recount3-study-explorer/

10
https://tau.cmmt.ubc.ca/eVITTA/(easyGSEA, easyVizR)

## Supplementary material

SUPPLEMENTARY DATA SHEET S1 PRISMA 2020 exclusion criteria.

SUPPLEMENTARY DATA SHEET S2 Data extraction from studies on human and mouse cells; separately including references.

SUPPLEMENTARY DATA SHEET S3 Risk of Bias assessment; updated extraction sheets; RoB methodology and methodology results for human and mouse cells separately including references.

SUPPLEMENTARY DATA SHEET S4 Summary tables on FSS magnitude/duration and apparatuses.

SUPPLEMENTARY DATA SHEET S5 Analysis of commonly investigated genes and metabolite mentioned by at least three studies.

SUPPLEMENTARY DATA SHEET S6 Commonly investigated genes and metabolite mentioned by at least three studies.

SUPPLEMENTARY DATA SHEET S7 Detailed results from gene list analysis.

SUPPLEMENTARY DATA SHEET S8 Detailed results from gene set enrichment analysis of the high-throughput studies.
