# Supplementary material for: Fluid flow shear stress and tissue remodeling—an orthodontic perspective: evidence synthesis and differential gene expression network analysis
Source: Front Bioeng Biotechnol. 2023 Sep 18;11:1256825. doi: 10.3389/fbioe.2023.1256825 (PMC10545883; doi:10.3389/fbioe.2023.1256825)
Supplement: Supplementary file 1 [file DataSheet7.pdf]

Supplement 7

**"Fluid Flow Shear Stress and Tissue Remodeling – an Orthodontic Perspective: Evidence Synthesis and Differential Gene Expression Network analysis"**

# Results from the gene list analysis
# Over-representation analysis (ORA)

## Contents


**7 Introduction........................................................2**

*7.1 General information ........................................................2*

*7.2 References ........................................................2*

*7.3 STRING network from the Homo sapiens (H. s.) gene list ........................................................3*
7.3.1 Complete network ........................................................3
7.3.1.1 Characteristics of the complete network ........................................................3
7.3.1.2 Gene list enrichment analysis ........................................................4
7.3.1.3 Hub genes by cytoHubba ........................................................6
7.3.2 GLay community detection ........................................................8
7.3.2.1 GLay cluster #1 – Gene list enrichment ........................................................8
7.3.2.2 GLay cluster #2 – Gene list enrichment ........................................................9
7.3.2.3 GLay cluster #3 – Gene list enrichment ........................................................10
7.3.2.4 GLay cluster #4 – Gene list enrichment ........................................................10

*7.4 STRING network from the Mus musculus (M.m.) gene list ........................................................11*
7.4.1 Complete network ........................................................11
7.4.1.1 Characteristics of the complete network ........................................................11
7.4.1.2 Gene list enrichment ........................................................12
7.4.1.3 Hub genes by cytoHubba ........................................................14
7.4.2 GLay community clustering ........................................................16
7.4.2.1 GLay cluster #1 – Gene list enrichment ........................................................16
7.4.2.2 GLay cluster #2 – Gene list enrichment ........................................................17
7.4.2.3 GLay cluster #3 – Gene list enrichment ........................................................18
7.4.2.4 GLay cluster #4 – Gene list enrichment ........................................................18

# 7 Introduction

## 7.1 General information

For this systematic review, a previously published workflow (Sun et al. 2021) was adopted and applied as follows. Gene lists were generated from the extracted data (Supplement 3) and consisted of unambiguously identified genetic loci with reported gene expression after fluid flow application (differential expressed genes, DEGs). The gene lists were prepared for human and mouse cell types separately. Therefore, the human gene list consisted of DEGs identified in human primary periodontal ligament cells (hPDLCs), human primary osteoblasts (hOBs), and human mesenchymal stem cells (hMSCs). The mouse gene list consisted of DEGS identified in mouse primary osteoblasts (mOBs) and the mouse immortalized osteocyte cell line MLO-Y4.

The following workflow was applied to each gene list:

- STRING search within *Cytoscape* version 3.9.1 (Shannon et al. 2003) using *stringApp* version 2.0.1 (Doncheva et al. 2019) with the following settings: species specified as either "*Homo sapiens*" or "*Mus musculus*", confidence cutoff set to 0.7; no further restrictions were applied
- The *stringApp* was used for STRING enrichment of the complete network and the identified clusters using GeneOntology/Biological Process (Thomas 2017), KEGG Pathways (Du et al. 2014), and WikiPathways (Martens et al. 2021; Pico et al. 2008). To increase specificity in the pathways and ontology terms a cut-off of > 0.05 (i.e., 5 %) was applied meaning that at least 5% of the genes from the gene list must be covered by the individual term (Sun et al. 2021) with a minimum of 4 genes from the specific gene list. General pathways related to cancer and/or infectious diseases like "Chagas disease" (hsa05142) or "Pertussis" (hsa05133) were removed, and their accession numbers were reported. From each database, the top 15 according to false discovery rate (FDR) value were reported if available.
- Clustering was applied with the GLay community detection algorithm (Su et al. 2010) as implemented in *clusterMaker2* version 2.3.4 (Morris et al. 2011) using default values. Each cluster was extracted as a separate network and individually analyzed with STRING enrichment.
- Hub genes were identified with *cytoHubba* (version 0.1) (Chin et al. 2014) as described (Sun et al. 2021): the total score cut-off was set to ≥ 2× mean SUMtotal score.

## 7.2 References


Chin CH, Chen SH, Wu HH, Ho CW, Ko MT, Lin CY (2014). *cytoHubba*: identifying hub objects and sub-networks from complex interactome. BMC Syst Biol; 8(Suppl 4):S11.

Doncheva NT, Morris JH, Gorodkin J, Jensen LJ (2019). Cytoscape StringApp: Network Analysis and Visualization of Proteomics Data. Journal of Proteome Research; 18(2):623-632.

Du J, Yuan Z, Ma Z, Song J, Xie X, Chen Y (2014). KEGG-PATH: Kyoto encyclopedia of genes and genomes-based pathway analysis using a path analysis model. Mol Biosyst; 10(9):2441-7.

Martens M, Ammar A, Riutta A, Waagmeester A, Slenter DN, Hanspers K, R AM, Digles D, Lopes EN, Ehrhart F, Dupuis LJ, Winckers LA, Coort SL, Willighagen EL, Evelo CT, Pico AR, Kutmon M (2021). WikiPathways: connecting communities. Nucleic Acids Res; 49(D1):D613-D621.

Morris JH, Apeltsin L, Newman AM, Baumbach J, Wittkop T, Su G, Bader GD, Ferrin TE (2011). *clusterMaker*: a multi-algorithm clustering plugin for Cytoscape. BMC Bioinformatics; 12:436.

Pico AR, Kelder T, van Iersel MP, Hanspers K, Conklin BR, Evelo C (2008). WikiPathways: pathway editing for the people. PLoS Biol; 6(7):e184.

Shannon P, Markiel A, Ozier O, Baliga NS, Wang JT, Ramage D, Amin N, Schwikowski B, Ideker T (2003). Cytoscape: a software environment for integrated models of biomolecular interaction networks. Genome Res; 13(11):2498-504.

Su G, Kuchinsky A, Morris JH, States DJ, Meng F (2010). GLay: community structure analysis of biological networks. Bioinformatics; 26(24):3135-7.

Sun C, Janjic Rankovic M, Folwaczny M, Otto S, Wichelhaus A, Baumert U (2021). Effect of Tension on Human Periodontal Ligament Cells: Systematic Review and Network Analysis. Front Bioeng Biotechnol; 9:695053.

Thomas PD (2017). The Gene Ontology and the Meaning of Biological Function. Methods Mol Biol; 1446:15-24.

## 7.3 STRING network from the *Homo sapiens* (*H. s.*) gene list

### 7.3.1 Complete network

#### *7.3.1.1 Characteristics of the complete network*

- Gene list consisted of 80 genetic loci.
- Contains data from the hMSC, hPDLF and hOBs data extraction tables
- Network characteristics of the undirected STRING network:

| Parameter | Complete network | Connected network only |
|---|---|---|
| Number of nodes | 80 | 73 |
| Number of edges | 308 | 308 |
| Average number of neighbors | 8.438 | 8.438 |
| Network diameter | 8 | 6 |
| Network radius | 6 | 4 |
| Characteristic path length | 2.613 | 2.613 |
| Clustering coefficient | 0.536 | 0.536 |
| Network density | 0.117 | 0.117 |
| Network heterogeneity | 0.758 | 0.753 |
| Network centralization | 0.265 | 0.265 |
| Connected components | 8 | 1 |

- STRING enrichment with all 80 nodes and for each of the clusters was done. The cut-off of 5% and a minimum content of 4 entries from the gene list were applied. The results were sorted according to the false discovery rate (FDR) value and the top 15 enrichment terms from each database were assembled into the tables. Some general terms, mostly dealing with cancer or specific infectious diseases, were removed. The database entries ("Term name") were reported.
- Hub genes were identified by application of a cut-off, that was defined as ≥ 2× mean(SUMtotal).
- Clustering analysis applied with GLay community detection algorithm with default values detected 4 clusters with a minimum of 5 members with an average cluster size of 7.273 and an average modularity of 0.685.

Summary for the complete STRING network and the 4 clusters.

| Gene list | Genes (n) in STRING network | Cluster number | Number of genes (n) and genes in cluster | | Cluster score |
|---|---|---|---|---|---|
| *H.s.* | Genes in the *H.s.* PPI network (73): ADIPOQ, ALPL, BAX, BCL2, BGLAP, BMP2, BMP7, CCL3, CCL5, CD44, COL1A1, CXCL8, CYP24A1, CYP27B1, EGR1, ENG, FGF2, FOS, FOSB, FOSL1, FOSL2, GLI1, HIF1A, HMOX1, IBSP, IGF1, IGFBP1, IL1B, IL1RN, IL6, ITGB1, JUN, KDR, MAP3K8, MMP1, MMP2, MRTFA, MYH11, MYH2, NANOG, NFKB1, NOS1, NOS2, NOS3, PDGFA, PDGFRA, PDGFRB, PECAM1, POU5F1, PPARA, PTCH1, PTGES, PTGS1, PTGS2, RHOA, RUNX2, S100A4, S100A7, S100A8, SOX2, SP7, SPP1, TAGLN, TGFB1, THY1, TIMP1, TIMP2, TNFAIP6, TNFRSF11B, TNFSF11, VDR, VEGFA, VWF<br>Genes outside the H.s. PPI network (7): DUSP6, GADD45B, IER3, PON1, TRPV1, TRPV4, WIF1<br>Hub genes (cut-off: SUMscore ≥ 4596.61; n=11): VEGFA, IL6, FGF2, IL1B, MMP2, TGFB1, MMP1, IGF1, TIMP1, RUNX2, JUN | 1 | (20) | ALPL, BAX, BCL2, BGLAP, BMP2, BMP7, COL1A1, CYP24A1, CYP27B1, GLI1, IBSP, IGF1, IGFBP1, PTCH1, RUNX2, SP7, SPP1, TNFRSF11B, TNFSF11, VDR | 0.191 |
| | | 2 | (26) | ADIPOQ, CCL3, CCL5, CXCL8, HMOX1, IL1B, IL1RN, IL6, JUN, MAP3K8, MMP1, MMP2, NFKB1, NOS1, NOS2, NOS3, PPARA, PTGES, PTGS1, PTGS2, S100A7, TGFB1, TIMP1, TIMP2, TNFAIP6, VEGFA | 0.239 |
| | | 3 | (22) | CD44, ENG, FGF2, HIF1A, ITGB1, KDR, MRTFA, MYH11, MYH2, NANOG, PDGFA, PDGFRA, PDGFRB, PECAM1, POU5F1, RHOA, S100A4, S100A8, SOX2, TAGLN, THY1, VWF | 0.211 |
| | | 4 | (5) | EGR1, FOS, FOSB, FOSL1, FOSL2 | 0.044 |

### 7.3.1.2 Gene list enrichment analysis

| # Back-ground genes | # Genes | Fraction | Description | FDR value | Genes | p-value | Term name |
|---|---|---|---|---|---|---|---|
| **GO Biological Process** | | | | | | | |
| 522 | 30 | 0.057 | Positive regulation of cell migration | 4.54E-23 | HMOX1, TGFB1, COL1A1, GLI1, TNFAIP6, PDGFRA, PDGFRB, IL1B, KDR, FGF2, THY1, NOS3, IGF1, CXCL8, PDGFA, PTGS2, S100A7, JUN, BMP2, BMP7, ITGB1, BCL2, IL6, RHOA, TRPV4, HIF1A, PECAM1, CCL5, CCL3, VEGFA | 3.18E-26 | GO:0030335 |
| 265 | 21 | 0.079 | Ossification | 1.47E-18 | CYP24A1, MMP2, TGFB1, COL1A1, IBSP, CYP27B1, GLI1, IGF1, SOX2, PTGS2, BGLAP, RUNX2, ALPL, BMP2, SPP1, BMP7, BCL2, TNFSF11, RHOA, SP7, CCL3 | 5.95E-21 | GO:0001503 |
| 313 | 19 | 0.061 | Response to lipopolysaccharide | 8.22E-15 | TGFB1, NFKB1, CYP27B1, IL1B, NOS3, FOS, CXCL8, NOS2, PTGES, PTGS2, S100A7, S100A8, JUN, ALPL, IL6, RHOA, CCL5, CCL3, NOS1 | 5.82E-17 | GO:0032496 |
| 338 | 19 | 0.056 | Extracellular matrix organization | 2.92E-14 | TIMP1, MMP2, TGFB1, COL1A1, IBSP, PDGFRA, VWF, TIMP2, KDR, FGF2, TNFRSF11B, MMP1, PDGFA, ENG, SPP1, ITGB1, MYH11, CD44, PECAM1 | 2.23E-16 | GO:0030198 |
| 292 | 18 | 0.062 | Regulation of erk1 and erk2 cascade | 4.16E-14 | TGFB1, PDGFRA, PDGFRB, IL1B, KDR, FGF2, DUSP6, PDGFA, S100A7, JUN, BMP2, TNFSF11, ADIPOQ, CD44, TRPV4, CCL5, CCL3, VEGFA | 3.30E-16 | GO:0070372 |
| 315 | 18 | 0.057 | Angiogenesis | 1.36E-13 | HMOX1, MMP2, PDGFRA, PDGFRB, KDR, FGF2, THY1, NOS3, CXCL8, PDGFA, PTGS2, S100A7, JUN, ENG, ITGB1, HIF1A, PECAM1, VEGFA | 1.16E-15 | GO:0001525 |
| 328 | 18 | 0.055 | Response to steroid hormone | 2.52E-13 | TGFB1, COL1A1, FOSB, IL1RN, FOS, FOSL1, PTGS2, BGLAP, ENG, ALPL, SPP1, BMP7, BCL2, PPARA, IL6, ADIPOQ, RHOA, VDR | 2.26E-15 | GO:0048545 |
| 209 | 15 | 0.072 | Positive regulation of erk1 and erk2 cascade | 1.58E-12 | TGFB1, PDGFRA, PDGFRB, KDR, FGF2, PDGFA, S100A7, JUN, BMP2, TNFSF11, CD44, TRPV4, CCL5, CCL3, VEGFA | 1.54E-14 | GO:0070374 |
| 52 | 10 | 0.192 | Regulation of pri-mirna transcription by rna polymerase ii | 6.13E-12 | TGFB1, EGR1, POU5F1, FGF2, FOS, FOSL1, JUN, BMP2, PPARA, HIF1A | 6.35E-14 | GO:1902893 |
| 164 | 13 | 0.079 | Response to corticosteroid | 2.67E-11 | COL1A1, FOSB, IL1RN, FOS, FOSL1, PTGS2, BGLAP, ENG, ALPL, BCL2, IL6, ADIPOQ, RHOA | 3.08E-13 | GO:0031960 |
| 127 | 12 | 0.094 | Osteoblast differentiation | 3.32E-11 | CYP24A1, COL1A1, IBSP, GLI1, SOX2, BGLAP, RUNX2, ALPL, BMP2, SPP1, SP7, CCL3 | 3.88E-13 | GO:0001649 |
| 29 | 8 | 0.276 | Response to vitamin d | 1.48E-10 | CYP24A1, CYP27B1, PTGS2, BGLAP, ALPL, SPP1, BMP7, VDR | 1.85E-12 | GO:0033280 |
| 197 | 13 | 0.066 | Regulation of ossification | 2.12E-10 | TGFB1, CYP27B1, GLI1, IGF1, PTCH1, BGLAP, RUNX2, BMP2, BMP7, BCL2, IL6, HIF1A, CCL3 | 2.75E-12 | GO:0030278 |
| 160 | 12 | 0.075 | Response to nutrient | 3.70E-10 | HMOX1, CYP24A1, COL1A1, CYP27B1, TNFRSF11B, PTGS2, BGLAP, ALPL, SPP1, BMP7, ADIPOQ, VDR | 4.95E-12 | GO:0007584 |
| 120 | 11 | 0.092 | Odontogenesis | 3.96E-10 | COL1A1, PDGFRA, BAX, BGLAP, RUNX2, ALPL, BMP2, BMP7, TNFSF11, PPARA, RHOA | 5.37E-12 | GO:0042476 |
| **KEGG Pathways** (Excluded: hsa05142, hsa05133, hsa04932, hsa05140, hsa05152, hsa05130, hsa05167) | | | | | | | |
| 98 | 13 | 0.133 | AGE-RAGE signaling pathway in diabetic complications | 1.12E-13 | MMP2, TGFB1, COL1A1, NFKB1, EGR1, IL1B, BAX, NOS3, CXCL8, JUN, BCL2, IL6, VEGFA | 6.67E-16 | hsa04933 |
| 130 | 14 | 0.108 | Fluid shear stress and atherosclerosis | 1.12E-13 | HMOX1, MMP2, NFKB1, IL1B, KDR, NOS3, FOS, PDGFA, JUN, BCL2, RHOA, TRPV4, PECAM1, VEGFA | 6.77E-16 | hsa05418 |
| 92 | 12 | 0.130 | IL-17 signaling pathway | 9.47E-13 | NFKB1, FOSB, IL1B, FOS, CXCL8, FOSL1, MMP1, PTGS2, S100A7, S100A8, JUN, IL6 | 1.13E-14 | hsa04657 |
| 198 | 14 | 0.071 | Focal adhesion | 1.02E-11 | COL1A1, IBSP, PDGFRA, VWF, PDGFRB, KDR, IGF1, PDGFA, JUN, SPP1, ITGB1, BCL2, RHOA, VEGFA | 1.52E-13 | hsa04510 |
| 85 | 11 | 0.129 | Rheumatoid arthritis | 1.02E-11 | TGFB1, IL1B, FOS, CXCL8, MMP1, JUN, TNFSF11, IL6, CCL5, CCL3, VEGFA | 1.67E-13 | hsa05323 |
| 288 | 15 | 0.052 | MAPK signaling pathway | 6.08E-11 | GADD45B, TGFB1, NFKB1, PDGFRA, PDGFRB, MAP3K8, IL1B, KDR, FGF2, DUSP6, IGF1, FOS, PDGFA, JUN, VEGFA | 1.27E-12 | hsa04010 |
| 78 | 10 | 0.128 | EGFR tyrosine kinase inhibitor resistance | 9.14E-11 | PDGFRA, PDGFRB, KDR, FGF2, BAX, IGF1, PDGFA, BCL2, IL6, VEGFA | 2.45E-12 | hsa01521 |
| 196 | 13 | 0.066 | Proteoglycans in cancer | 9.14E-11 | MMP2, TGFB1, COL1A1, NANOG, KDR, FGF2, IGF1, PTCH1, ITGB1, CD44, RHOA, HIF1A, VEGFA | 2.59E-12 | hsa05205 |
| 128 | 11 | 0.086 | Relaxin signaling pathway | 3.14E-10 | MMP2, TGFB1, COL1A1, NFKB1, NOS3, FOS, MMP1, NOS2, JUN, NOS1, VEGFA | 1.03E-11 | hsa04926 |
| 101 | 10 | 0.099 | Toll-like receptor signaling pathway | 6.71E-10 | NFKB1, MAP3K8, IL1B, FOS, CXCL8, JUN, SPP1, IL6, CCL5, CCL3 | 2.60E-11 | hsa04620 |
| 103 | 10 | 0.097 | Parathyroid hormone synthesis, secretion and action | 7.46E-10 | CYP24A1, CYP27B1, EGR1, FOS, BGLAP, RUNX2, BCL2, TNFSF11, RHOA, VDR | 3.11E-11 | hsa04928 |
| 106 | 10 | 0.094 | HIF-1 signaling pathway | 9.05E-10 | HMOX1, TIMP1, NFKB1, NOS3, IGF1, NOS2, BCL2, IL6, HIF1A, VEGFA | 4.04E-11 | hsa04066 |
| 160 | 11 | 0.069 | MicroRNAs in cancer | 1.94E-09 | HMOX1, CYP24A1, NFKB1, PDGFRA, PDGFRB, PDGFA, PTGS2, BCL2, CD44, RHOA, VEGFA | 9.80E-11 | hsa05206 |
| 122 | 10 | 0.082 | Osteoclast differentiation | 2.74E-09 | TGFB1, NFKB1, FOSB, IL1B, FOSL2, TNFRSF11B, FOS, FOSL1, JUN, TNFSF11 | 1.47E-10 | hsa04380 |
| 112 | 8 | 0.071 | TNF signaling pathway | 3.83E-07 | NFKB1, MAP3K8, IL1B, FOS, PTGS2, JUN, IL6, CCL5 | 3.07E-08 | hsa04668 |
| **WikiPathways** (Excluded: WP3646, WP706, WP615, WP4707) | | | | | | | |
| 270 | 25 | 0.093 | IL-18 signaling pathway | 1.09E-23 | HMOX1, TIMP1, MMP2, COL1A1, NFKB1, IER3, IL1B, BAX, TNFRSF11B, FOS, CXCL8, MMP1, NOS2, PTGS2, JUN, RUNX2, BMP2, SPP1, BCL2, TNFSF11, IL6, ADIPOQ, CCL5, CCL3, VEGFA | 1.61E-26 | WP4754 |
| 63 | 14 | 0.222 | Lung fibrosis | 2.42E-17 | HMOX1, TIMP1, MMP2, TGFB1, IL1B, FGF2, IGF1, CXCL8, PDGFA, SPP1, BMP7, IL6, CCL5, CCL3 | 7.13E-20 | WP3624 |

| # Back-ground genes | # Genes | Fraction | Description | FDR value | Genes | p-value | Term name |
|---|---|---|---|---|---|---|---|
| 196 | 14 | 0.071 | Focal adhesion | 2.26E-11 | COL1A1, IBSP, PDGFRA, VWF, PDGFRB, KDR, IGF1, PDGFA, JUN, SPP1, ITGB1, BCL2, RHOA, VEGFA | 1.33E-13 | WP306 |
| 302 | 16 | 0.053 | Focal adhesion: PI3K-Akt-mTOR-signaling pathway | 2.26E-11 | COL1A1, IBSP, PDGFRA, VWF, PDGFRB, KDR, FGF2, NOS3, IGF1, NOS2, PDGFA, SPP1, ITGB1, HIF1A, NOS1, VEGFA | 1.61E-13 | WP3932 |
| 182 | 13 | 0.071 | Vitamin D receptor pathway | 1.04E-10 | CYP24A1, TGFB1, CYP27B1, TIMP2, IGFBP1, TNFRSF11B, BGLAP, S100A4, S100A8, SPP1, TNFSF11, HIF1A, VDR | 1.07E-12 | WP2877 |
| 14 | 7 | 0.500 | Osteoblast signaling | 1.19E-10 | COL1A1, IBSP, PDGFRA, PDGFRB, TNFRSF11B, BGLAP, TNFSF11 | 1.58E-12 | WP322 |
| 53 | 9 | 0.170 | Cardiac progenitor differentiation | 2.17E-10 | TGFB1, NANOG, PDGFRA, POU5F1, KDR, FGF2, THY1, IGF1, SOX2 | 3.30E-12 | WP2406 |
| 114 | 11 | 0.096 | Gastrin signaling pathway | 2.17E-10 | NFKB1, EGR1, FOS, CXCL8, PTGS2, JUN, BMP2, ITGB1, CD44, RHOA, VEGFA | 3.20E-12 | WP4659 |
| 83 | 10 | 0.120 | EGFR tyrosine kinase inhibitor resistance | 2.25E-10 | PDGFRA, PDGFRB, KDR, FGF2, BAX, IGF1, PDGFA, BCL2, IL6, VEGFA | 4.31E-12 | WP4806 |
| 35 | 8 | 0.229 | Photodynamic therapy-induced NF-kB survival signaling | 3.30E-10 | MMP2, NFKB1, IL1B, CXCL8, MMP1, PTGS2, IL6, VEGFA | 6.82E-12 | WP3617 |
| 19 | 7 | 0.368 | Overview of nanoparticle effects | 3.97E-10 | HMOX1, BAX, CXCL8, PTGS1, PTGS2, BCL2, IL6 | 8.77E-12 | WP3287 |
| 91 | 10 | 0.110 | Corticotropin-releasing hormone signaling pathway | 4.24E-10 | TGFB1, NFKB1, FOSB, FOSL2, NOS3, FOS, CXCL8, FOSL1, BCL2, RHOA | 9.99E-12 | WP2355 |
| 62 | 9 | 0.145 | Endochondral ossification | 4.69E-10 | TGFB1, FGF2, IGF1, PTCH1, RUNX2, ALPL, SPP1, BMP7, VEGFA | 1.18E-11 | WP474 |
| 62 | 9 | 0.145 | Endochondral ossification with skeletal dysplasias | 4.69E-10 | TGFB1, FGF2, IGF1, PTCH1, RUNX2, ALPL, SPP1, BMP7, VEGFA | 1.18E-11 | WP4808 |
| 103 | 10 | 0.097 | Toll-like receptor signaling pathway | 1.05E-09 | NFKB1, MAP3K8, IL1B, FOS, CXCL8, JUN, SPP1, IL6, CCL5, CCL3 | 3.11E-11 | WP75 |

### 7.3.1.3 Hub genes by cytoHubba

Top hub genes identified in the human STRING network derived from the human (n = 80) DEG list. Different score measures were calculated by cytoHubba (Chin et al. 2014). The cut-off was calculated (total score ≥ 4596.6) for this network (Sun et al. 2021). In this table, the genes were ordered in descending order according to the total score (above the dashed line). These hub genes were colored red in **Figure 5**.

| Gene symbol | Local-based methods | | | | Global-based methods | | | | | | | Total score |
|---|---|---|---|---|---|---|---|---|---|---|---|---|
| | MCC | DMNC | MNC | Degree | EPC | BottleNeck | EcCentricity | Closeness | Radiality | Betweenness | Stress | (SUMscore |
| VEGFA | 13848 | 0.43507 | 27 | 27 | 25.267 | 7 | 0.22813 | 47.75 | 4.84132 | 606.77843 | 3298 | 17892.30 |
| IL6 | 13384 | 0.38528 | 26 | 26 | 24.612 | 16 | 0.22813 | 46.41667 | 4.7526 | 529.21808 | 3438 | 17495.61 |
| FGF2 | 13014 | 0.41184 | 25 | 25 | 24.269 | 8 | 0.22813 | 45.5 | 4.72726 | 414.27583 | 2194 | 15755.41 |
| IL1B | 12324 | 0.44216 | 20 | 20 | 22.915 | 1 | 0.22813 | 43.41667 | 4.67656 | 265.59285 | 2026 | 14728.27 |
| MMP2 | 11570 | 0.52927 | 14 | 14 | 21.578 | 2 | 0.22813 | 38.75 | 4.47378 | 123.37719 | 842 | 12630.94 |
| TGFB1 | 11070 | 0.52077 | 15 | 15 | 21.686 | 5 | 0.22813 | 40.91667 | 4.62587 | 187.55267 | 1260 | 12620.53 |
| MMP1 | 11042 | 0.64479 | 11 | 11 | 19.381 | 2 | 0.22813 | 38.08333 | 4.49913 | 76.44875 | 454 | 11659.29 |
| IGF1 | 6021 | 0.42605 | 18 | 19 | 22.65 | 3 | 0.22813 | 42.16667 | 4.62587 | 366.18963 | 2086 | 8583.29 |
| TIMP1 | 6552 | 0.64479 | 11 | 11 | 19.579 | 1 | 0.22813 | 37.58333 | 4.47378 | 39.22588 | 408 | 7084.73 |
| RUNX2 | 2214 | 0.38589 | 16 | 18 | 19.324 | 7 | 0.22813 | 42.25 | 4.65122 | 926.30616 | 3724 | 6972.15 |
| JUN | 939 | 0.36854 | 19 | 20 | 21.127 | 6 | 0.22813 | 43.66667 | 4.71458 | 774.21035 | 2978 | 4806.32 |
| SPP1 | 2258 | 0.44871 | 16 | 16 | 20.191 | 12 | 0.22813 | 40.58333 | 4.58785 | 238.62252 | 1276 | 3882.66 |
| ITGB1 | 425 | 0.38056 | 15 | 16 | 19.675 | 3 | 0.22813 | 39.33333 | 4.47378 | 478.34297 | 2604 | 3605.43 |
| CD44 | 1444 | 0.41146 | 20 | 20 | 22.896 | 2 | 0.22813 | 42.25 | 4.60052 | 229.02873 | 1320 | 3105.41 |
| BGLAP | 2186 | 0.55995 | 11 | 11 | 16.026 | 4 | 0.22813 | 35.41667 | 4.28368 | 105.72414 | 664 | 3038.24 |
| BMP2 | 1501 | 0.4726 | 13 | 14 | 18.239 | 2 | 0.22813 | 38.58333 | 4.47378 | 200.99715 | 1012 | 2804.99 |
| CXCL8 | 1233 | 0.4726 | 13 | 14 | 19.065 | 2 | 0.22813 | 39.41667 | 4.52448 | 212.09527 | 1222 | 2759.80 |
| COL1A1 | 1514 | 0.44706 | 13 | 13 | 19.344 | 2 | 0.22813 | 37.83333 | 4.43576 | 119.10746 | 684 | 2407.40 |
| SP7 | 2160 | 0.69213 | 9 | 9 | 14.849 | 1 | 0.22813 | 33.25 | 4.16962 | 4.26216 | 44 | 2280.45 |
| HIF1A | 888 | 0.43064 | 15 | 15 | 21.237 | 4 | 0.22813 | 41 | 4.63854 | 168.50333 | 1024 | 2182.04 |
| IBSP | 1452 | 0.57279 | 9 | 9 | 16.012 | 1 | 0.22813 | 34.75 | 4.29635 | 50.66444 | 330 | 1907.52 |
| PTGS2 | 386 | 0.64146 | 8 | 10 | 15.327 | 3 | 0.22813 | 36.25 | 4.38507 | 288.83725 | 1038 | 1790.67 |
| RHOA | 76 | 0.4082 | 8 | 8 | 14.465 | 4 | 0.22813 | 35.33333 | 4.3724 | 267.23542 | 1344 | 1762.04 |
| FOS | 154 | 0.30655 | 13 | 13 | 16.652 | 3 | 0.22813 | 39.33333 | 4.5625 | 255.5747 | 1182 | 1681.66 |
| VDR | 4 | 0.30779 | 2 | 4 | 5.811 | 3 | 0.1825 | 27.93333 | 3.81476 | 280 | 1252 | 1583.05 |
| TNFSF11 | 738 | 0.45891 | 10 | 10 | 17.545 | 1 | 0.22813 | 37.5 | 4.49913 | 76.73088 | 684 | 1579.96 |
| NFKB1 | 25 | 0.56839 | 4 | 5 | 10.041 | 2 | 0.1825 | 30 | 3.94149 | 142 | 1034 | 1256.73 |
| KDR | 606 | 0.49759 | 12 | 12 | 18.515 | 1 | 0.22813 | 37.66667 | 4.44844 | 64.69924 | 442 | 1199.06 |
| NOS3 | 222 | 0.44117 | 11 | 11 | 16.31 | 2 | 0.22813 | 37.25 | 4.43576 | 130.88846 | 758 | 1193.55 |
| TNFRSF11B | 723 | 0.5854 | 7 | 8 | 14.494 | 1 | 0.22813 | 35.5 | 4.39774 | 40.14662 | 318 | 1152.35 |
| MYH2 | 5 | 0.30898 | 3 | 4 | 5.233 | 2 | 0.1825 | 26.8 | 3.65 | 147.03333 | 876 | 1073.21 |
| CCL5 | 272 | 0.47733 | 9 | 9 | 15.588 | 2 | 0.22813 | 36.25 | 4.41042 | 86.88346 | 586 | 1021.84 |
| PECAM1 | 648 | 0.57691 | 11 | 11 | 18.171 | 1 | 0.22813 | 36.5 | 4.38507 | 27.13656 | 192 | 950.00 |
| ENG | 606 | 0.54298 | 11 | 11 | 17.569 | 1 | 0.22813 | 36.66667 | 4.39774 | 30.01735 | 196 | 914.42 |
| ALPL | 720 | 0.71324 | 6 | 6 | 11.571 | 1 | 0.1825 | 30.7 | 4.03021 | 0 | 0 | 780.20 |
| MRTFA | 2 | 0 | 1 | 2 | 3.492 | 2 | 0.1825 | 25.63333 | 3.61198 | 88.29837 | 530 | 658.22 |
| HMOX1 | 252 | 0.52483 | 8 | 8 | 14.249 | 2 | 0.1825 | 33.98333 | 4.22031 | 40.73981 | 284 | 647.90 |
| BAX | 2 | 0 | 1 | 2 | 3.769 | 2 | 0.1825 | 26.76667 | 3.77674 | 142 | 442 | 625.49 |
| NOS2 | 248 | 0.49567 | 8 | 8 | 12.709 | 2 | 0.1825 | 33.65 | 4.19497 | 40.06345 | 264 | 621.30 |
| PDGFRB | 218 | 0.45891 | 10 | 10 | 18.032 | 3 | 0.22813 | 36.58333 | 4.41042 | 30.33452 | 258 | 589.05 |
| VWF | 265 | 0.62199 | 7 | 8 | 15.494 | 1 | 0.22813 | 35.16667 | 4.35972 | 23.00077 | 140 | 499.87 |
| MYH11 | 3 | 0.30779 | 2 | 3 | 3.591 | 1 | 0.1825 | 24.96667 | 3.53594 | 51.70163 | 326 | 419.29 |
| THY1 | 216 | 0.52506 | 9 | 9 | 15.914 | 1 | 0.22813 | 33.91667 | 4.22031 | 19.10858 | 84 | 392.91 |
| SOX2 | 150 | 0.54881 | 7 | 7 | 13.805 | 1 | 0.22813 | 33.5 | 4.23299 | 12.55508 | 76 | 305.87 |
| NANOG | 150 | 0.54881 | 7 | 7 | 13.682 | 1 | 0.22813 | 33.5 | 4.23299 | 12.55508 | 76 | 305.75 |
| PDGFRA | 144 | 0.61814 | 6 | 6 | 13.283 | 1 | 0.22813 | 33.91667 | 4.30903 | 4.74409 | 68 | 282.10 |
| POU5F1 | 168 | 0.5854 | 7 | 7 | 12.483 | 1 | 0.22813 | 32.5 | 4.15694 | 2.29372 | 16 | 251.25 |
| ADIPOQ | 25 | 0.56839 | 4 | 5 | 11.69 | 1 | 0.1825 | 32.73333 | 4.20764 | 27.11053 | 98 | 209.49 |
| CCL3 | 120 | 0.64826 | 5 | 5 | 11.671 | 1 | 0.1825 | 31.68333 | 4.11892 | 0 | 0 | 179.30 |
| FOSL2 | 120 | 0.64826 | 5 | 5 | 8.302 | 1 | 0.1825 | 29.23333 | 3.91615 | 0 | 0 | 173.28 |
| FOSL1 | 120 | 0.64826 | 5 | 5 | 7.74 | 1 | 0.1825 | 29.23333 | 3.91615 | 0 | 0 | 172.72 |
| EGR1 | 120 | 0.64826 | 5 | 5 | 7.652 | 1 | 0.1825 | 29.23333 | 3.91615 | 0 | 0 | 172.63 |
| FOSB | 120 | 0.64826 | 5 | 5 | 7.545 | 1 | 0.1825 | 29.23333 | 3.91615 | 0 | 0 | 172.53 |
| PDGFA | 32 | 0.42794 | 6 | 6 | 12.693 | 1 | 0.22813 | 32.25 | 4.1316 | 3.35105 | 22 | 120.08 |
| S100A8 | 3 | 0.30779 | 2 | 3 | 8.626 | 1 | 0.1825 | 30.01667 | 4.03021 | 6.96859 | 56 | 115.13 |
| GLI1 | 2 | 0 | 1 | 2 | 3.617 | 1 | 0.1825 | 26.76667 | 3.77674 | 11.13053 | 46 | 97.47 |
| PPARA | 3 | 0.30779 | 2 | 3 | 6.985 | 1 | 0.1825 | 28.56667 | 3.92882 | 4.33333 | 16 | 69.30 |
| BMP7 | 6 | 0.46346 | 3 | 3 | 7.933 | 1 | 0.1825 | 29.81667 | 4.01753 | 0 | 0 | 55.41 |
| IL1RN | 6 | 0.46346 | 3 | 3 | 8.655 | 1 | 0.1825 | 28.83333 | 3.90347 | 0 | 0 | 55.04 |
| NOS1 | 6 | 0.46346 | 3 | 3 | 6.327 | 1 | 0.1825 | 25.66667 | 3.59931 | 0 | 0 | 49.24 |
| PTCH1 | 2 | 0 | 1 | 2 | 3.676 | 1 | 0.1825 | 25.26667 | 3.59931 | 2.00362 | 8 | 48.73 |
| TIMP2 | 2 | 0.30779 | 2 | 2 | 6.04 | 1 | 0.1825 | 26.08333 | 3.70069 | 0 | 0 | 43.31 |
| TNFAIP6 | 2 | 0.30779 | 2 | 2 | 5.472 | 1 | 0.1825 | 25.66667 | 3.65 | 0 | 0 | 42.28 |
| PTGS1 | 2 | 0.30779 | 2 | 2 | 3.57 | 1 | 0.1825 | 24.25 | 3.49792 | 0 | 0 | 38.81 |
| PTGES | 2 | 0.30779 | 2 | 2 | 3.458 | 1 | 0.1825 | 24.25 | 3.49792 | 0 | 0 | 38.70 |
| S100A7 | 1 | 0 | 1 | 1 | 3.815 | 1 | 0.1825 | 26.65 | 3.81476 | 0 | 0 | 38.46 |
| IGFBP1 | 1 | 0 | 1 | 1 | 3.748 | 1 | 0.1825 | 25.98333 | 3.72604 | 0 | 0 | 37.64 |

| Gene symbol | Local-based methods | | | | Global-based methods | | | | | | | Total score |
|---|---|---|---|---|---|---|---|---|---|---|---|---|
| | MCC | DMNC | MNC | Degree | EPC | BottleNeck | EcCentricity | Closeness | Radiality | Betweenness | Stress | (SUMscore |
| TAGLN | 2 | 0 | 1 | 2 | 1.855 | 1 | 0.15208 | 19.98333 | 2.77552 | 2 | 2 | 34.77 |
| CYP27B1 | 2 | 0.30779 | 2 | 2 | 2.187 | 1 | 0.15208 | 20.48333 | 2.9276 | 0 | 0 | 33.06 |
| CYP24A1 | 2 | 0.30779 | 2 | 2 | 2.076 | 1 | 0.15208 | 20.48333 | 2.9276 | 0 | 0 | 32.95 |
| MAP3K8 | 1 | 0 | 1 | 1 | 2.519 | 1 | 0.15208 | 20.93333 | 3.04167 | 0 | 0 | 30.65 |
| BCL2 | 1 | 0 | 1 | 1 | 1.533 | 1 | 0.15208 | 19.56667 | 2.87691 | 0 | 0 | 28.13 |
| S100A4 | 1 | 0 | 1 | 1 | 1.636 | 1 | 0.15208 | 19.31667 | 2.75017 | 0 | 0 | 27.85 |
| DUSP6 | 0 | 0 | 0 | 0 | 1 | 0 | 0 | 0 | 0 | 0 | 0 | 1.00 |
| IER3 | 0 | 0 | 0 | 0 | 1 | 0 | 0 | 0 | 0 | 0 | 0 | 1.00 |
| TRPV1 | 0 | 0 | 0 | 0 | 1 | 0 | 0 | 0 | 0 | 0 | 0 | 1.00 |
| PON1 | 0 | 0 | 0 | 0 | 1 | 0 | 0 | 0 | 0 | 0 | 0 | 1.00 |
| TRPV4 | 0 | 0 | 0 | 0 | 1 | 0 | 0 | 0 | 0 | 0 | 0 | 1.00 |
| WIF1 | 0 | 0 | 0 | 0 | 1 | 0 | 0 | 0 | 0 | 0 | 0 | 1.00 |
| GADD45B | 0 | 0 | 0 | 0 | 1 | 0 | 0 | 0 | 0 | 0 | 0 | 1.00 |

## 7.3.2 GLay community detection

### *7.3.2.1 GLay cluster #1 – Gene list enrichment*

| # Back-ground genes | # Genes | Fraction | Description | FDR value | Genes | p-value | Term name |
|---|---|---|---|---|---|---|---|
| **GO Biological Process** | | | | | | | |
| 265 | 15 | 0.057 | Ossification | 2.73E-20 | CYP24A1, COL1A1, IBSP, CYP27B1, GLI1, IGF1, BGLAP, RUNX2, ALPL, BMP2, SPP1, BMP7, BCL2, TNFSF11, SP7 | 2.13E-24 | GO:0001503 |
| 127 | 10 | 0.079 | Osteoblast differentiation | 2.25E-13 | CYP24A1, COL1A1, IBSP, GLI1, BGLAP, RUNX2, ALPL, BMP2, SPP1, SP7 | 3.50E-17 | GO:0001649 |
| 29 | 7 | 0.241 | Response to vitamin d | 1.24E-11 | CYP24A1, CYP27B1, BGLAP, ALPL, SPP1, BMP7, VDR | 2.90E-15 | GO:0033280 |
| 84 | 8 | 0.095 | Response to vitamin | 6.71E-11 | CYP24A1, COL1A1, CYP27B1, BGLAP, ALPL, SPP1, BMP7, VDR | 2.09E-14 | GO:0033273 |
| 160 | 9 | 0.056 | Response to nutrient | 8.51E-11 | CYP24A1, COL1A1, CYP27B1, TNFRSF11B, BGLAP, ALPL, SPP1, BMP7, VDR | 3.31E-14 | GO:0007584 |
| 92 | 8 | 0.087 | Biomineral tissue development | 8.90E-11 | COL1A1, IBSP, CYP27B1, IGF1, BGLAP, ALPL, BMP2, SPP1 | 4.16E-14 | GO:0031214 |
| 120 | 8 | 0.067 | Odontogenesis | 3.67E-10 | COL1A1, BAX, BGLAP, RUNX2, ALPL, BMP2, BMP7, TNFSF11 | 3.14E-13 | GO:0042476 |
| 77 | 6 | 0.078 | Regulation of tissue remodeling | 8.80E-08 | BAX, TNFRSF11B, BGLAP, SPP1, TNFSF11, VDR | 1.78E-10 | GO:0034103 |
| 117 | 6 | 0.051 | Regulation of osteoblast differentiation | 6.98E-07 | GLI1, IGF1, PTCH1, RUNX2, BMP2, BMP7 | 1.96E-09 | GO:0045667 |
| 50 | 5 | 0.100 | Bone mineralization | 7.56E-07 | IBSP, CYP27B1, IGF1, BGLAP, BMP2 | 2.18E-09 | GO:0030282 |
| 24 | 4 | 0.167 | Cellular response to vitamin | 4.55E-06 | CYP24A1, COL1A1, BGLAP, VDR | 1.59E-08 | GO:0071295 |
| 33 | 4 | 0.121 | Positive regulation of tissue remodeling | 1.30E-05 | BAX, SPP1, TNFSF11, VDR | 5.11E-08 | GO:0034105 |
| 43 | 4 | 0.093 | Regulation of bone resorption | 3.20E-05 | TNFRSF11B, BGLAP, SPP1, TNFSF11 | 1.37E-07 | GO:0045124 |
| 60 | 4 | 0.067 | Positive regulation of osteoblast differentiation | 9.26E-05 | IGF1, RUNX2, BMP2, BMP7 | 4.83E-07 | GO:0045669 |
| 75 | 4 | 0.053 | Regulation of bone mineralization | 1.70E-04 | CYP27B1, BGLAP, BMP2, BMP7 | 1.13E-06 | GO:0030500 |
| **KEGG Pathways** | | | | | | | |
| 103 | 7 | 0.068 | Parathyroid hormone synthesis, secretion and action | 3.56E-09 | CYP24A1, CYP27B1, BGLAP, RUNX2, BCL2, TNFSF11, VDR | 1.06E-11 | hsa04928 |
| 62 | 4 | 0.065 | Basal cell carcinoma | 9.19E-05 | GLI1, BAX, PTCH1, BMP2 | 5.47E-07 | hsa05217 |
| **WikiPathways** | | | | | | | |
| 14 | 5 | 0.357 | Osteoblast signaling | 5.05E-09 | COL1A1, IBSP, TNFRSF11B, BGLAP, TNFSF11 | 7.44E-12 | WP322 |
| 62 | 6 | 0.097 | Endochondral ossification | 1.18E-08 | IGF1, PTCH1, RUNX2, ALPL, SPP1, BMP7 | 5.21E-11 | WP474 |
| 62 | 6 | 0.097 | Endochondral ossification with skeletal dysplasias | 1.18E-08 | IGF1, PTCH1, RUNX2, ALPL, SPP1, BMP7 | 5.21E-11 | WP4808 |
| 15 | 4 | 0.267 | OSX and miRNAs in tooth development | 3.43E-07 | RUNX2, ALPL, BMP7, SP7 | 3.03E-09 | WP3971 |
| 22 | 4 | 0.182 | FGF23 signaling in hypophosphatemic rickets and related disorders | 1.13E-06 | CYP24A1, CYP27B1, ALPL, SPP1 | 1.16E-08 | WP4790 |
| 33 | 4 | 0.121 | Type I collagen synthesis in the context of osteogenesis imperfecta | 4.33E-06 | COL1A1, TNFRSF11B, TNFSF11, SP7 | 5.11E-08 | WP4786 |
| 44 | 4 | 0.091 | Interleukin-11 signaling pathway | 1.13E-05 | IBSP, BGLAP, RUNX2, BCL2 | 1.49E-07 | WP2332 |
| 55 | 4 | 0.073 | Overlap between signal transduction pathways contributing to LMNA laminopathies | 2.14E-05 | TNFRSF11B, RUNX2, BMP2, SPP1 | 3.47E-07 | WP4879 |

### *7.3.2.2 GLay cluster #2 – Gene list enrichment*

| # Back-ground genes | # Genes | Fraction | Description | FDR value | Genes | p-value | Term name |
|---|---|---|---|---|---|---|---|
| **GO Biological Process** | | | | | | | |
| 136 | 8 | 0.059 | Regulation of smooth muscle cell proliferation | 3.40E-09 | HMOX1, MMP2, NOS3, PTGS2, JUN, IL6, ADIPOQ, CCL5 | 9.78E-12 | GO:0048660 |
| 144 | 8 | 0.056 | Positive regulation of small molecule metabolic process | 4.86E-09 | NFKB1, IL1B, NOS3, NOS2, PTGS2, PPARA, ADIPOQ, NOS1 | 1.51E-11 | GO:0062013 |
| 34 | 5 | 0.147 | Lipopolysaccharide-mediated signaling pathway | 2.42E-07 | TGFB1, IL1B, NOS3, CCL5, CCL3 | 1.53E-09 | GO:0031663 |
| 88 | 6 | 0.068 | Positive regulation of smooth muscle cell proliferation | 3.36E-07 | HMOX1, MMP2, PTGS2, JUN, IL6, CCL5 | 2.21E-09 | GO:0048661 |
| 98 | 6 | 0.061 | Positive regulation of leukocyte chemotaxis | 5.64E-07 | CXCL8, S100A7, IL6, CCL5, CCL3, VEGFA | 4.08E-09 | GO:0002690 |
| 49 | 5 | 0.102 | Negative regulation of blood pressure | 1.03E-06 | NOS3, NOS2, PPARA, ADIPOQ, NOS1 | 8.28E-09 | GO:0045776 |
| 56 | 5 | 0.089 | Positive regulation of blood vessel endothelial cell migration | 1.70E-06 | HMOX1, TGFB1, NOS3, PTGS2, VEGFA | 1.55E-08 | GO:0043536 |
| 23 | 4 | 0.174 | Positive regulation of cyclase activity | 4.23E-06 | TIMP2, NOS3, NOS2, NOS1 | 4.19E-08 | GO:0031281 |
| 24 | 4 | 0.167 | Positive regulation of lyase activity | 4.79E-06 | TIMP2, NOS3, NOS2, NOS1 | 4.88E-08 | GO:0051349 |
| 28 | 4 | 0.143 | Positive regulation of vascular endothelial growth factor production | 7.90E-06 | TGFB1, IL1B, PTGS2, IL6 | 8.54E-08 | GO:0010575 |
| 81 | 5 | 0.062 | Granulocyte chemotaxis | 8.13E-06 | IL1B, CXCL8, CCL5, CCL3, VEGFA | 8.86E-08 | GO:0071621 |
| 36 | 4 | 0.111 | Positive regulation of fatty acid metabolic process | 1.68E-05 | IL1B, PTGS2, PPARA, ADIPOQ | 2.16E-07 | GO:0045923 |
| 38 | 4 | 0.105 | Protein kinase b signaling | 1.99E-05 | TGFB1, IL1B, CCL5, CCL3 | 2.63E-07 | GO:0043491 |
| 52 | 4 | 0.077 | Regulation of vascular associated smooth muscle cell proliferation | 5.57E-05 | HMOX1, MMP2, JUN, ADIPOQ | 8.54E-07 | GO:1904705 |
| **KEGG Pathways** (Excluded: hsa05142, hsa05140, hsa05133, hsa05146, hsa05321, hsa05219, hsa05144, hsa05134, hsa04932) | | | | | | | |
| 85 | 9 | 0.106 | Rheumatoid arthritis | 8.93E-13 | TGFB1, IL1B, CXCL8, MMP1, JUN, IL6, CCL5, CCL3, VEGFA | 2.66E-15 | hsa05323 |
| 98 | 9 | 0.092 | AGE-RAGE signaling pathway in diabetic complications | 1.49E-12 | MMP2, TGFB1, NFKB1, IL1B, NOS3, CXCL8, JUN, IL6, VEGFA | 8.87E-15 | hsa04933 |
| 128 | 9 | 0.070 | Relaxin signaling pathway | 7.27E-12 | MMP2, TGFB1, NFKB1, NOS3, MMP1, NOS2, JUN, NOS1, VEGFA | 8.65E-14 | hsa04926 |
| 92 | 8 | 0.087 | IL-17 signaling pathway | 3.37E-11 | NFKB1, IL1B, CXCL8, MMP1, PTGS2, S100A7, JUN, IL6 | 5.02E-13 | hsa04657 |
| 101 | 8 | 0.079 | Toll-like receptor signaling pathway | 5.69E-11 | NFKB1, MAP3K8, IL1B, CXCL8, JUN, IL6, CCL5, CCL3 | 1.02E-12 | hsa04620 |
| 106 | 7 | 0.066 | HIF-1 signaling pathway | 3.95E-09 | HMOX1, TIMP1, NFKB1, NOS3, NOS2, IL6, VEGFA | 1.06E-10 | hsa04066 |
| 112 | 7 | 0.063 | TNF signaling pathway | 5.13E-09 | NFKB1, MAP3K8, IL1B, PTGS2, JUN, IL6, CCL5 | 1.53E-10 | hsa04668 |
| 130 | 7 | 0.054 | Fluid shear stress and atherosclerosis | 1.15E-08 | HMOX1, MMP2, NFKB1, IL1B, NOS3, JUN, VEGFA | 4.12E-10 | hsa05418 |
| 62 | 4 | 0.065 | Cytosolic DNA-sensing pathway | 2.15E-05 | NFKB1, IL1B, IL6, CCL5 | 1.66E-06 | hsa04623 |
| 67 | 4 | 0.060 | Epithelial cell signaling in Helicobacter pylori infection | 2.65E-05 | NFKB1, CXCL8, JUN, CCL5 | 2.23E-06 | hsa05120 |
| **WikiPathways** (Exluded: WP3646, WP5113, WP2431, WP5039, WP4891) | | | | | | | |
| 270 | 15 | 0.056 | IL-18 signaling pathway | 8.67E-19 | HMOX1, TIMP1, MMP2, NFKB1, IL1B, CXCL8, MMP1, NOS2, PTGS2, JUN, IL6, ADIPOQ, CCL5, CCL3, VEGFA | 1.28E-21 | WP4754 |
| 63 | 9 | 0.143 | Lung fibrosis | 4.89E-14 | HMOX1, TIMP1, MMP2, TGFB1, IL1B, CXCL8, IL6, CCL5, CCL3 | 2.16E-16 | WP3624 |
| 35 | 8 | 0.229 | Photodynamic therapy-induced NF-kB survival signaling | 6.94E-14 | MMP2, NFKB1, IL1B, CXCL8, MMP1, PTGS2, IL6, VEGFA | 4.09E-16 | WP3617 |
| 10 | 6 | 0.600 | Aspirin and miRNAs | 3.16E-12 | NFKB1, NOS3, PTGS1, PTGS2, PPARA, VEGFA | 2.33E-14 | WP4707 |
| 103 | 8 | 0.078 | Toll-like receptor signaling pathway | 1.33E-10 | NFKB1, MAP3K8, IL1B, CXCL8, JUN, IL6, CCL5, CCL3 | 1.18E-12 | WP75 |
| 32 | 6 | 0.188 | Signal transduction through IL1R | 6.67E-10 | TGFB1, NFKB1, IL1RN, IL1B, JUN, IL6 | 7.87E-12 | WP4496 |
| 139 | 8 | 0.058 | Regulation of toll-like receptor signaling pathway | 8.70E-10 | NFKB1, MAP3K8, IL1B, CXCL8, JUN, IL6, CCL5, CCL3 | 1.16E-11 | WP1449 |
| 155 | 8 | 0.052 | Nonalcoholic fatty liver disease | 1.80E-09 | TGFB1, NFKB1, IL1B, CXCL8, JUN, PPARA, IL6, ADIPOQ | 2.66E-11 | WP4396 |
| 19 | 5 | 0.263 | Overview of nanoparticle effects | 7.05E-09 | HMOX1, CXCL8, PTGS1, PTGS2, IL6 | 1.14E-10 | WP3287 |
| 89 | 6 | 0.067 | T-cell receptor (TCR) signaling pathway | 1.07E-07 | TGFB1, NFKB1, MAP3K8, IL1B, JUN, IL6 | 2.36E-09 | WP69 |
| 46 | 5 | 0.109 | NO/cGMP/PKG mediated neuroprotection | 2.57E-07 | NFKB1, IL1B, NOS3, NOS2, NOS1 | 6.17E-09 | WP4008 |
| 105 | 6 | 0.057 | Senescence and autophagy in cancer | 2.57E-07 | TGFB1, IL1B, CXCL8, JUN, IL6, CCL3 | 6.06E-09 | WP615 |
| 19 | 4 | 0.211 | LTF danger signal response pathway | 7.57E-07 | NFKB1, IL1B, CXCL8, IL6 | 2.12E-08 | WP4478 |
| 66 | 5 | 0.076 | AGE/RAGE pathway | 1.14E-06 | MMP2, NFKB1, NOS3, NOS2, JUN | 3.36E-08 | WP2324 |
| 24 | 4 | 0.167 | IL1 and megakaryocytes in obesity | 1.58E-06 | TIMP1, NFKB1, TIMP2, IL1B | 4.88E-08 | WP2865 |

## 7.3.2.3 GLay cluster #3 – Gene list enrichment

| # Back-ground genes | # Genes | Fraction | Description | FDR value | Genes | p-value | Term name |
|---|---|---|---|---|---|---|---|
| **GO Biological Process** | | | | | | | |
| 67 | 5 | 0.075 | Endothelial cell migration | 3.83E-06 | KDR, FGF2, ITGB1, RHOA, PECAM1 | 1.46E-08 | GO:0043542 |
| 56 | 4 | 0.071 | Leukocyte cell-cell adhesion | 7.71E-05 | S100A8, ITGB1, CD44, PECAM1 | 5.58E-07 | GO:0007159 |
| 62 | 4 | 0.065 | Myofibril assembly | 1.00E-04 | PDGFRA, PDGFRB, ITGB1, MYH11 | 8.21E-07 | GO:0030239 |
| 65 | 4 | 0.062 | Somatic stem cell population maintenance | 1.20E-04 | NANOG, POU5F1, FGF2, SOX2 | 9.83E-07 | GO:0035019 |
| 75 | 4 | 0.053 | Positive regulation of lipase activity | 1.80E-04 | PDGFRA, PDGFRB, FGF2, RHOA | 1.70E-06 | GO:0060193 |
| **KEGG Pathways** (Excluded: hsa05218) | | | | | | | |
| 78 | 5 | 0.064 | EGFR tyrosine kinase inhibitor resistance | 2.02E-06 | PDGFRA, PDGFRB, KDR, FGF2, PDGFA | 3.01E-08 | hsa01521 |
| **WikiPathways** | | | | | | | |
| 53 | 7 | 0.132 | Cardiac progenitor differentiation | 1.97E-10 | NANOG, PDGFRA, POU5F1, KDR, FGF2, THY1, SOX2 | 2.90E-13 | WP2406 |
| 24 | 4 | 0.167 | Angiogenesis | 4.07E-06 | PDGFRA, KDR, FGF2, HIF1A | 2.40E-08 | WP1539 |
| 83 | 5 | 0.060 | EGFR tyrosine kinase inhibitor resistance | 5.48E-06 | PDGFRA, PDGFRB, KDR, FGF2, PDGFA | 4.04E-08 | WP4806 |

## 7.3.2.4 GLay cluster #4 – Gene list enrichment

| # Back-ground genes | # Genes | Fraction | Category | Description | FDR value | Genes | p-value | Term name |
|---|---|---|---|---|---|---|---|---|
| 33 | 5 | 0.152 | STRING Clusters | Mixed, incl. bzip maf transcription factor, and bhlh transcription factor binding | 9.54E-11 | EGR1, FOSB, FOSL2, FOS, FOSL1 | 2.10E-14 | CL:6308 |
| 8 | 4 | 0.500 | STRING Clusters | Mixed, incl. ap-1 transcription factor, and arc, c-lobe | 6.13E-10 | EGR1, FOSB, FOSL2, FOS | 4.05E-13 | CL:6326 |
| 9 | 4 | 0.444 | InterPro Domains | AP-1 transcription factor | 3.96E-09 | FOSB, FOSL2, FOS, FOSL1 | 5.85E-13 | IPR000837 |
| 36 | 4 | 0.111 | Pfam | bZIP transcription factor | 1.86E-07 | FOSB, FOSL2, FOS, FOSL1 | 7.47E-11 | PF00170 |
| 33 | 4 | 0.121 | Pfam | bZIP Maf transcription factor | 1.86E-07 | FOSB, FOSL2, FOS, FOSL1 | 5.40E-11 | PF03131 |
| 44 | 4 | 0.091 | Pfam | Basic region leucine zipper | 1.86E-07 | FOSB, FOSL2, FOS, FOSL1 | 1.59E-10 | PF07716 |
| 39 | 4 | 0.103 | Reactome Pathways | NGF-stimulated transcription | 2.19E-07 | EGR1, FOSB, FOS, FOSL1 | 1.01E-10 | HSA-9031628 |
| 54 | 4 | 0.074 | InterPro Domains | Basic-leucine zipper domain | 1.17E-06 | FOSB, FOSL2, FOS, FOSL1 | 3.46E-10 | IPR004827 |
| 38 | 3 | 0.079 | SMART Domains | Basic region leucin zipper | 6.18E-05 | FOSL2, FOS, FOSL1 | 8.51E-08 | SM00338 |
| 20 | 3 | 0.150 | GO Biological Process | Response to corticosterone | 1.80E-04 | FOSB, FOS, FOSL1 | 1.42E-08 | GO:0051412 |
| 40 | 3 | 0.075 | GO Biological Process | Positive regulation of pri-mirna transcription by rna polymerase ii | 4.20E-04 | EGR1, FOS, FOSL1 | 9.85E-08 | GO:1902895 |
| 45 | 3 | 0.067 | GO Biological Process | Response to progesterone | 4.40E-04 | FOSB, FOS, FOSL1 | 1.38E-07 | GO:0032570 |
| 11 | 2 | 0.182 | GO Biological Process | Response to gravity | 0.0057 | FOS, FOSL1 | 4.07E-06 | GO:0009629 |

## 7.4 STRING network from the *Mus musculus* (*M.m.*) gene list

### 7.4.1 Complete network

#### *7.4.1.1 Characteristics of the complete network*

- Gene list of 76 genetic loci
- Contains data from the osteoblast and osteocyte data extraction tables
- Network characteristics of the undirected STRING network:

| Parameter | Complete network | Connected network only |
|---|---|---|
| Number of nodes | 76 | 74 |
| Number of edges | 346 | 346 |
| Average number of neighbors | 9.351 | 9.351 |
| Network diameter | 5 | 5 |
| Network radius | 3 | 3 |
| Characteristic path length | 2.409 | 2.409 |
| Clustering coefficient | 0.584 | 0.584 |
| Network density | 0.128 | 0.128 |
| Network heterogeneity | 0.757 | 0.757 |
| Network centralization | 0.446 | 0.446 |
| Connected components | 3 | 1 |

- STRING enrichment with complete network
- Hub genes were identified by application of a cut-off, that was defined as ≥ 2× mean(SUMtotal).
- Clustering analysis applied with GLay with default values generated 4 clusters with an average size of 12.667. The modularity was 0.621 with clusters ranging from 1 (unclustered genes) to 33.
- For each cluster STRING enrichment was applied.

| Gene list | Genes (n) in STRING network |
|---|---|
| *M.m.* | Genes in the *M.m.* PPI network (74): Actb, Alpl, Apc, Axin1, Bad, Bax, Bglap, Bmpr1a, Casp3, Ccl7, Ccna2, Ccnd1, Cd44, Ctnnb1, Cxcl1, Cxcl2, Dkk1, Dkk2, Dlx1, Dlx5, Dmp1, Esr1, Fabp4, Fos, Fzd6, Gja1, Gjc1, Hgf, Igf1, Igf1r, Il11, Il6, Itgav, Itgb3, Jun, Lef1, Lepr, Lif, Lpl, Lrp5, Mepe, Mki67, Mmp14, Ncoa1, Nos2, Npy, Pdpn, Phex, Piezo1, Pkd1, Pkd2, Pparg, Ptger2, Ptgs1, Ptgs2, Runx2, Sfrp1, Sfrp2, Sfrp4, Sost, Sp7, Spp1, Tgfb1, Tjp1, Tnf, Tnfrsf11b, Tnfsf11, Tuba1a, Vcl, Vegfa, Wnt1, Wnt3a, Wnt4, Wnt5a<br>Genes outside the *M.m.* PPI network (2): Eral1, Piezo2<br>Hub genes (cut-off: SUMscore ≥ 12622.9; n=12): Ctnnb1, Wnt1, Wnt3a, Wnt5a, Dkk1, Wnt4, Lrp5, Sfrp1, Il6, Tnf, Actb, Jun |

| Cluster number | Number of genes (n) and genes in cluster | | Cluster score |
|---|---|---|---|
| 1 | (21) | Apc, Axin1, Bad, Bax, Casp3, Ccn1, Ccnd1, Dkk1, Dkk2, Esr1, Fzd6, Lef1, Lrp5, Mki67, Sfrp1, Sfrp2, Sfrp4, Wnt1, Wnt3a, Wnt4, Wnt5a | 0.223 |
| 2 | (33) | Actb, Ccl7, Cd44, Cxcl1, Cxcl2, Fabp4, Fos, Hgf, Igf1, Igf1r, Il11, Il6, Itgav, Itgb3, Jun, Lepr, Lif, Lpl, Mmp14, Ncoa1, Nos2, Npy, Pdpn, Pparg, Ptger2, Ptgs1, Ptgs2, Spp1, Tgfb1, Tnf, Tnfrsf11b, Tnfsf11, Vegfa | 0.25 |
| 3 | (10) | Bmpr1a, Ctnnb1, Gja1, Gjc1, Piezo1, Pkd1, Pkd2, Tjp1, Tuba1a, Vcl | 0.06 |
| 4 | (10) | Alpl, Bglap, Dlx1, Dlx5, Dmp1, Mepe, Phex, Runx2, Sost, Sp7 | 0.088 |

## 7.4.1.2 Gene list enrichment

| # Back-ground genes | # Genes | Fraction | Description | FDR value | Genes | p-value | Term name |
|---|---|---|---|---|---|---|---|
| **GO Biological Process** | | | | | | | |
| 493 | 27 | 0.055 | Gland development | 2.05E-22 | Tgfb1, Cd44, Igf1r, Ctnnb1, Wnt3a, Tnfsf11, Wnt1, Tnf, Lrp5, Il6, Lef1, Hgf, Mki67, Sfrp1, Bmpr1a, Wnt4, Pkd1, Igf1, Gja1, Wnt5a, Esr1, Apc, Ncoa1, Pkd2, Ccnd1, Jun, Vegfa | 5.26E-25 | GO:0048732 |
| 210 | 20 | 0.095 | Regulation of animal organ morphogenesis | 1.84E-20 | Tgfb1, Ctnnb1, Wnt3a, Fzd6, Tnf, Dkk1, Sfrp2, Hgf, Bax, Sfrp1, Bmpr1a, Wnt4, Wnt5a, Lif, Dmp1, Esr1, Apc, Tnfrsf11b, Runx2, Vegfa | 6.14E-23 | GO:2000027 |
| 224 | 20 | 0.089 | Regulation of ossification | 5.46E-20 | Sost, Tgfb1, Ctnnb1, Tnf, Dkk1, Lrp5, Il6, Sfrp2, Sfrp1, Bmpr1a, Wnt4, Dlx5, Igf1, Gja1, Wnt5a, Mepe, Bglap, Apc, Runx2, Vegfa | 2.03E-22 | GO:0030278 |
| 243 | 20 | 0.082 | Wnt signaling pathway | 2.09E-19 | Sost, Sfrp4, Cd44, Ctnnb1, Wnt3a, Fzd6, Wnt1, Dkk1, Lrp5, Lef1, Sfrp2, Dkk2, Sfrp1, Wnt4, Pkd1, Wnt5a, Axin1, Apc, Pkd2, Ccnd1 | 9.23E-22 | GO:0016055 |
| 240 | 19 | 0.079 | Ossification | 3.96E-18 | Sost, Tgfb1, Wnt3a, Tnfsf11, Lrp5, Lef1, Alpl, Sfrp1, Ptgs2, Dlx5, Igf1, Gja1, Dmp1, Bglap, Sp7, Phex, Mmp14, Spp1, Runx2 | 2.17E-20 | GO:0001503 |
| 365 | 21 | 0.058 | Regulation of epithelial cell proliferation | 1.37E-17 | Pparg, Tgfb1, Ctnnb1, Wnt3a, Itgb3, Tnf, Bad, Il6, Sfrp2, Bax, Sfrp1, Bmpr1a, Dlx5, Igf1, Gja1, Wnt5a, Esr1, Apc, Ccnd1, Jun, Vegfa | 8.39E-20 | GO:0050678 |
| 195 | 17 | 0.087 | Morphogenesis of a branching epithelium | 7.9E-17 | Tgfb1, Cd44, Ctnnb1, Wnt1, Lrp5, Il6, Lef1, Sfrp2, Sfrp1, Wnt4, Pkd1, Igf1, Wnt5a, Esr1, Pkd2, Mmp14, Vegfa | 5.88E-19 | GO:0061138 |
| 367 | 20 | 0.054 | Epithelial tube morphogenesis | 2.32E-16 | Tgfb1, Cd44, Ctnnb1, Wnt3a, Fzd6, Wnt1, Lrp5, Lef1, Sfrp2, Sfrp1, Wnt4, Pkd1, Igf1, Gja1, Wnt5a, Esr1, Pkd2, Mmp14, Casp3, Vegfa | 1.97E-18 | GO:0060562 |
| 79 | 13 | 0.165 | Canonical wnt signaling pathway | 6.05E-16 | Sfrp4, Ctnnb1, Wnt3a, Fzd6, Wnt1, Lrp5, Lef1, Sfrp2, Sfrp1, Wnt4, Wnt5a, Axin1, Apc | 5.68E-18 | GO:0060070 |
| 149 | 15 | 0.101 | Regulation of peptidyl-serine phosphorylation | 1.11E-15 | Tgfb1, Cd44, Wnt3a, Tnf, Dkk1, Il6, Sfrp2, Hgf, Bax, Ptgs2, Wnt5a, Lif, Axin1, Il11, Vegfa | 1.12E-17 | GO:0033135 |
| 163 | 15 | 0.092 | Branching morphogenesis of an epithelial tube | 3.54E-15 | Tgfb1, Cd44, Ctnnb1, Wnt1, Lrp5, Lef1, Sfrp2, Wnt4, Pkd1, Igf1, Wnt5a, Esr1, Pkd2, Mmp14, Vegfa | 3.90E-17 | GO:0048754 |
| 207 | 16 | 0.077 | Positive regulation of epithelial cell proliferation | 3.78E-15 | Tgfb1, Ctnnb1, Wnt3a, Itgb3, Tnf, Bad, Il6, Sfrp1, Bmpr1a, Dlx5, Igf1, Wnt5a, Esr1, Ccnd1, Jun, Vegfa | 4.19E-17 | GO:0050679 |
| 222 | 16 | 0.072 | Response to mechanical stimulus | 1.02E-14 | Sost, Tgfb1, Ctnnb1, Fos, Tnf, Bad, Il6, Ptgs2, Piezo2, Pkd1, Igf1, Gja1, Pkd2, Mmp14, Piezo1, Jun | 1.18E-16 | GO:0009612 |
| 142 | 14 | 0.099 | Regulation of fat cell differentiation | 1.65E-14 | Pparg, Tgfb1, Wnt3a, Lpl, Wnt1, Tnf, Lrp5, Sfrp2, Sfrp1, Ptgs2, Igf1, Wnt5a, Axin1, Vegfa | 1.96E-16 | GO:0045598 |
| 113 | 13 | 0.115 | Regulation of morphogenesis of an epithelium | 3.18E-14 | Tgfb1, Ctnnb1, Fzd6, Tnf, Sfrp2, Hgf, Sfrp1, Wnt4, Gja1, Wnt5a, Lif, Esr1, Vegfa | 3.97E-16 | GO:1905330 |
| **KEGG Pathways** (Excluded: mmu05200, mmu05224, mmu05225, mmu05165, mmu05010, mmu05226, mmu05210, mmu05217, mmu05167, mmu04934, mmu05213, mmu05146, mmu05161) | | | | | | | |
| 157 | 18 | 0.115 | Wnt signaling pathway | 5.61E-20 | Sost, Sfrp4, Ctnnb1, Wnt3a, Fzd6, Wnt1, Dkk1, Lrp5, Lef1, Sfrp2, Dkk2, Sfrp1, Wnt4, Wnt5a, Axin1, Apc, Ccnd1, Jun | 5.07E-22 | mmu04310 |
| 199 | 19 | 0.095 | Proteoglycans in cancer | 6.6E-20 | Tgfb1, Cd44, Igf1r, Ctnnb1, Wnt3a, Itgb3, Fzd6, Wnt1, Tnf, Itgav, Hgf, Wnt4, Igf1, Wnt5a, Esr1, Casp3, Ccnd1, Actb, Vegfa | 7.96E-22 | mmu05205 |
| 137 | 13 | 0.095 | Signaling pathways regulating pluripotency of stem cells | 1.47E-13 | Igf1r, Ctnnb1, Wnt3a, Fzd6, Wnt1, Bmpr1a, Wnt4, Dlx5, Igf1, Wnt5a, Lif, Axin1, Apc | 3.99E-15 | mmu04550 |
| 156 | 13 | 0.083 | Hippo signaling pathway | 6.32E-13 | Tgfb1, Ctnnb1, Wnt3a, Fzd6, Wnt1, Lef1, Bmpr1a, Wnt4, Wnt5a, Axin1, Apc, Ccnd1, Actb | 1.90E-14 | mmu04390 |
| 196 | 13 | 0.066 | Focal adhesion | 7.58E-12 | Igf1r, Ctnnb1, Itgb3, Vcl, Bad, Itgav, Hgf, Igf1, Ccnd1, Actb, Jun, Spp1, Vegfa | 2.97E-13 | mmu04510 |
| 86 | 10 | 0.116 | Rheumatoid arthritis | 2.62E-11 | Tgfb1, Fos, Tnfsf11, Tnf, Il6, Cxcl1, Cxcl2, Il11, Jun, Vegfa | 1.10E-12 | mmu05323 |
| 112 | 10 | 0.089 | TNF signaling pathway | 2.78E-10 | Fos, Tnf, Il6, Cxcl1, Ptgs2, Lif, Cxcl2, Mmp14, Casp3, Jun | 1.26E-11 | mmu04668 |
| 90 | 9 | 0.100 | IL-17 signaling pathway | 1.08E-09 | Ccl7, Fos, Tnf, Il6, Cxcl1, Ptgs2, Cxcl2, Casp3, Jun | 5.52E-11 | mmu04657 |
| 119 | 9 | 0.076 | Thyroid hormone signaling pathway | 9.32E-09 | Ctnnb1, Itgb3, Bad, Itgav, Wnt4, Esr1, Ncoa1, Ccnd1, Actb | 5.62E-10 | mmu04919 |
| 91 | 8 | 0.088 | Endocrine resistance | 2.67E-08 | Igf1r, Fos, Bad, Bax, Igf1, Esr1, Ccnd1, Jun | 1.77E-09 | mmu01522 |
| 141 | 9 | 0.064 | Fluid shear stress and atherosclerosis | 3.32E-08 | Ctnnb1, Itgb3, Fos, Tnf, Itgav, Bmpr1a, Actb, Jun, Vegfa | 2.30E-09 | mmu05418 |
| 101 | 8 | 0.079 | AGE-RAGE signaling pathway in diabetic complications | 5.07E-08 | Tgfb1, Tnf, Il6, Bax, Casp3, Ccnd1, Jun, Vegfa | 3.82E-09 | mmu04933 |
| 156 | 9 | 0.058 | mTOR signaling pathway | 6.55E-08 | Igf1r, Wnt3a, Fzd6, Wnt1, Tnf, Lrp5, Wnt4, Igf1, Wnt5a | 5.33E-09 | mmu04150 |
| 121 | 8 | 0.066 | Osteoclast differentiation | 1.66E-07 | Pparg, Tgfb1, Itgb3, Fos, Tnfsf11, Tnf, Tnfrsf11b, Jun | 1.45E-08 | mmu04380 |
| 78 | 7 | 0.090 | EGFR tyrosine kinase inhibitor resistance | 1.88E-07 | Igf1r, Bad, Il6, Hgf, Bax, Igf1, Vegfa | 1.70E-08 | mmu01521 |
| **WikiPathways** (Excluded: WP3857) | | | | | | | |
| 106 | 16 | 0.151 | Wnt signaling pathway (NetPath) | 4.3E-19 | Ctnnb1, Wnt3a, Fzd6, Wnt1, Dkk1, Lrp5, Lef1, Sfrp2, Sfrp1, Wnt4, Wnt5a, Axin1, Apc, Ccnd1, Jun, Runx2 | 2.17E-21 | WP539 |
| 96 | 13 | 0.135 | Wnt signaling pathway and pluripotency | 5.63E-15 | Cd44, Ctnnb1, Wnt3a, Fzd6, Wnt1, Lrp5, Lef1, Wnt4, Wnt5a, Axin1, Apc, Ccnd1, Jun | 5.69E-17 | WP723 |
| 116 | 13 | 0.112 | ESC pluripotency pathways | 3.58E-14 | Ctnnb1, Wnt3a, Fos, Fzd6, Wnt1, Lrp5, Bmpr1a, Wnt4, Wnt5a, Lif, Axin1, Apc, Jun | 5.43E-16 | WP339 |
| 59 | 11 | 0.186 | Wnt signaling pathway | 3.58E-14 | Sfrp4, Ctnnb1, Wnt3a, Fzd6, Wnt1, Wnt4, Wnt5a, Axin1, Apc, Ccnd1, Jun | 6.80E-16 | WP403 |
| 287 | 16 | 0.056 | PluriNetWork: mechanisms associated with pluripotency | 2.11E-13 | Tgfb1, Cd44, Ctnnb1, Wnt3a, Fos, Dkk1, Lrp5, Lef1, Wnt5a, Lif, Axin1, Apc, Ncoa1, Casp3, Ccnd1, Spp1 | 5.33E-15 | WP1763 |
| 50 | 10 | 0.200 | TGF-beta signaling pathway | 2.68E-13 | Tgfb1, Ctnnb1, Fos, Wnt1, Tnf, Lef1, Lif, Jun, Spp1, Runx2 | 8.13E-15 | WP113 |
| 132 | 12 | 0.091 | Adipogenesis genes | 2.23E-12 | Pparg, Tgfb1, Sfrp4, Ctnnb1, Lpl, Wnt1, Tnf, Il6, Fabp4, Igf1, Lif, Ncoa1 | 7.88E-14 | WP447 |
| 99 | 10 | 0.101 | Spinal cord injury | 8.85E-11 | Tgfb1, Nos2, Fos, Tnf, Il6, Cxcl1, Gja1, Cxcl2, Casp3, Ccnd1 | 4.02E-12 | WP2432 |

| # Back-ground genes | # Genes | Fraction | Description | FDR value | Genes | p-value | Term name |
|---|---|---|---|---|---|---|---|
| 82 | 9 | 0.110 | MicroRNAs in cardiomyocyte hypertrophy | 5.06E-10 | Tgfb1, Igf1r, Ctnnb1, Wnt3a, Tnf, Lrp5, Igf1, Wnt5a, Lif | 2.55E-11 | WP1560 |
| 183 | 11 | 0.060 | Focal adhesion | 1.11E-09 | Itgb3, Vcl, Bad, Itgav, Hgf, Igf1, Ccnd1, Actb, Jun, Spp1, Vegfa | 6.18E-11 | WP85 |
| 62 | 8 | 0.129 | Endochondral ossification | 1.74E-09 | Tgfb1, Igf1r, Alpl, Bmpr1a, Igf1, Spp1, Runx2, Vegfa | 1.06E-10 | WP1270 |
| 72 | 8 | 0.111 | Primary focal segmental glomerulosclerosis (FSGS) | 4.81E-09 | Tgfb1, Ctnnb1, Itgb3, Vcl, Dkk1, Lrp5, Itgav, Mki67 | 3.16E-10 | WP2573 |
| 61 | 7 | 0.115 | Lung fibrosis | 4.93E-08 | Tgfb1, Tnf, Il6, Hgf, Igf1, Cxcl2, Spp1 | 3.49E-09 | WP3632 |
| 80 | 7 | 0.088 | Apoptosis | 2.63E-07 | Igf1r, Tnf, Bad, Bax, Igf1, Casp3, Jun | 2.00E-08 | WP1254 |
| 27 | 5 | 0.185 | Cytokines and inflammatory response | 9.70E-07 | Tgfb1, Tnf, Il6, Cxcl1, Il11 | 7.84E-08 | WP222 |
| 14 | 4 | 0.286 | Osteoclast signaling | 4.42E-06 | Itgb3, Tnfsf11, Tnfrsf11b, Spp1 | 3.79E-07 | WP454 |
| 51 | 5 | 0.098 | Id signaling pathway | 1.53E-05 | Igf1r, Ctnnb1, Ccna2, Igf1, Vegfa | 1.39E-06 | WP512 |
| 98 | 6 | 0.061 | IL-6 signaling pathway | 1.63E-05 | Fos, Bad, Il6, Ncoa1, Casp3, Jun | 1.56E-06 | WP387 |

## *7.4.1.3 Hub genes by cytoHubba*

Top hub genes identified in the human STRING network derived from the mouse (n = 76) DEG list. Different score measures were calculated by cytoHubba (Chin et al. 2014). The cut-off was calculated (total score ≥ 12622.9) for this network (Sun et al. 2021). In this table the genes were ordered in descending order according to the total score (above the dashed line). These hub genes were colored red in **Figure 5**.

| Gene | Local-based methods | | | | Global-based methods | | | | | | | Total score |
|---|---|---|---|---|---|---|---|---|---|---|---|---|
| symbol | MCC | DMNC | MNC | Degree | EPC | BottleNeck | EcCentricity | Closeness | Radiality | Betweenness | Stress | (SUMscore) |
| Ctnnb1 | 44794 | 0.359 | 41 | 41 | 31.360 | 32 | 0.325 | 56.833 | 4.508 | 1886.875 | 6374 | 53262.3 |
| Wnt1 | 37562 | 0.619 | 16 | 16 | 24.323 | 2 | 0.243 | 40.417 | 3.855 | 72.993 | 522 | 38260.5 |
| Wnt3a | 37584 | 0.637 | 16 | 16 | 23.618 | 1 | 0.243 | 40.083 | 3.828 | 52.399 | 426 | 38163.8 |
| Wnt5a | 36000 | 0.728 | 13 | 13 | 21.773 | 1 | 0.243 | 37.917 | 3.735 | 15.650 | 212 | 36319.0 |
| Dkk1 | 32088 | 0.619 | 16 | 16 | 24.371 | 1 | 0.243 | 40.417 | 3.855 | 65.943 | 530 | 32786.4 |
| Wnt4 | 30960 | 0.746 | 12 | 12 | 20.297 | 1 | 0.243 | 37.417 | 3.721 | 12.894 | 184 | 31244.3 |
| Lrp5 | 26904 | 0.641 | 15 | 15 | 24.022 | 1 | 0.243 | 39.917 | 3.841 | 52.169 | 476 | 27531.8 |
| Sfrp1 | 26040 | 0.717 | 12 | 12 | 21.264 | 1 | 0.243 | 37.917 | 3.761 | 21.613 | 264 | 26414.5 |
| Il6 | 15246 | 0.412 | 23 | 23 | 27.796 | 13 | 0.243 | 46.417 | 4.148 | 543.905 | 2292 | 18219.9 |
| Tnf | 15768 | 0.465 | 23 | 23 | 28.039 | 5 | 0.243 | 46.750 | 4.175 | 292.002 | 1602 | 17792.7 |
| Actb | 15372 | 0.509 | 21 | 21 | 26.991 | 2 | 0.243 | 45.583 | 4.135 | 277.308 | 1344 | 17114.8 |
| Jun | 12978 | 0.509 | 19 | 19 | 26.808 | 3 | 0.243 | 44.583 | 4.108 | 237.537 | 1350 | 14682.8 |
| Axin1 | 10826 | 0.611 | 11 | 11 | 21.869 | 1 | 0.243 | 37.417 | 3.748 | 17.387 | 118 | 11048.3 |
| Sfrp2 | 10080 | 0.787 | 8 | 8 | 16.885 | 1 | 0.243 | 35.417 | 3.668 | 8.102 | 148 | 10310.1 |
| Dkk2 | 10080 | 0.787 | 8 | 8 | 17.588 | 1 | 0.195 | 29.950 | 3.094 | 0.250 | 2 | 10150.9 |
| Vegfa | 8712 | 0.550 | 17 | 17 | 26.329 | 1 | 0.243 | 42.917 | 4.028 | 101.264 | 696 | 9618.3 |
| Tgfb1 | 8308 | 0.531 | 15 | 15 | 24.814 | 2 | 0.243 | 41.583 | 3.975 | 83.883 | 496 | 8991.0 |
| Pparg | 6832 | 0.429 | 17 | 17 | 26.004 | 2 | 0.243 | 43.417 | 4.068 | 317.338 | 1542 | 8801.5 |
| Casp3 | 6752 | 0.600 | 13 | 15 | 23.689 | 3 | 0.243 | 41.750 | 3.988 | 322.006 | 1120 | 8295.3 |
| Ptgs2 | 6128 | 0.585 | 12 | 14 | 22.847 | 3 | 0.243 | 41.083 | 3.961 | 339.244 | 1222 | 7787.0 |
| Fos | 5683 | 0.615 | 12 | 13 | 23.389 | 3 | 0.243 | 40.917 | 3.975 | 169.361 | 742 | 6691.5 |
| Lef1 | 5934 | 0.536 | 13 | 13 | 23.074 | 1 | 0.243 | 39.917 | 3.895 | 45.092 | 314 | 6387.8 |
| Fzd6 | 5040 | 0.768 | 7 | 7 | 16.720 | 1 | 0.243 | 34.750 | 3.641 | 0.000 | 0 | 5111.1 |
| Igf1 | 3680 | 0.507 | 18 | 18 | 26.559 | 1 | 0.243 | 43.583 | 4.055 | 153.702 | 930 | 4875.6 |
| Runx2 | 1287 | 0.411 | 20 | 21 | 27.084 | 3 | 0.243 | 44.750 | 4.068 | 414.938 | 1908 | 3730.5 |
| Sost | 1374 | 0.451 | 15 | 15 | 22.758 | 4 | 0.243 | 40.250 | 3.868 | 251.202 | 1096 | 2822.8 |
| Ccnd1 | 1298 | 0.481 | 15 | 15 | 25.092 | 1 | 0.243 | 41.917 | 4.001 | 149.745 | 694 | 2244.5 |
| Esr1 | 1242 | 0.549 | 13 | 13 | 23.034 | 1 | 0.243 | 40.750 | 3.961 | 55.897 | 372 | 1765.4 |
| Spp1 | 612 | 0.491 | 15 | 15 | 24.884 | 2 | 0.243 | 41.583 | 3.975 | 143.773 | 904 | 1762.9 |
| Cd44 | 320 | 0.539 | 10 | 12 | 21.015 | 4 | 0.243 | 39.750 | 3.908 | 305.362 | 1022 | 1738.8 |
| Tnfsf11 | 822 | 0.536 | 13 | 13 | 24.227 | 1 | 0.243 | 40.750 | 3.961 | 69.138 | 520 | 1507.9 |
| Bglap | 702 | 0.527 | 12 | 12 | 22.570 | 2 | 0.243 | 39.250 | 3.868 | 70.235 | 554 | 1418.7 |
| Sp7 | 654 | 0.543 | 11 | 11 | 20.060 | 1 | 0.243 | 38.583 | 3.841 | 57.557 | 450 | 1247.8 |
| Hgf | 864 | 0.641 | 8 | 8 | 19.628 | 1 | 0.243 | 37.083 | 3.801 | 5.677 | 40 | 988.1 |
| Apc | 726 | 0.525 | 8 | 8 | 17.949 | 1 | 0.243 | 36.417 | 3.748 | 29.920 | 138 | 969.8 |
| Sfrp4 | 840 | 0.695 | 7 | 7 | 16.569 | 1 | 0.243 | 35.417 | 3.695 | 2.011 | 16 | 929.6 |
| Pkd1 | 3 | 0.308 | 2 | 3 | 6.276 | 3 | 0.325 | 33.333 | 3.641 | 284.000 | 504 | 842.9 |
| Dmp1 | 156 | 0.379 | 10 | 10 | 17.588 | 1 | 0.195 | 34.867 | 3.521 | 96.914 | 484 | 814.5 |
| Tjp1 | 56 | 0.408 | 8 | 8 | 14.837 | 2 | 0.243 | 36.917 | 3.788 | 119.308 | 530 | 779.5 |
| Tnfrsf11b | 288 | 0.525 | 9 | 9 | 19.284 | 1 | 0.195 | 37.283 | 3.775 | 28.860 | 262 | 658.9 |
| Mki67 | 266 | 0.525 | 8 | 8 | 17.562 | 2 | 0.243 | 37.417 | 3.828 | 53.728 | 232 | 629.3 |
| Itgav | 210 | 0.479 | 10 | 10 | 20.186 | 1 | 0.243 | 36.333 | 3.681 | 36.518 | 224 | 552.4 |
| Itgb3 | 210 | 0.479 | 10 | 10 | 19.616 | 1 | 0.243 | 36.333 | 3.681 | 36.518 | 224 | 551.9 |
| Gja1 | 32 | 0.428 | 6 | 6 | 11.454 | 1 | 0.243 | 34.750 | 3.668 | 70.054 | 342 | 507.6 |
| Pkd2 | 2 | 0.000 | 1 | 2 | 1.885 | 2 | 0.243 | 23.667 | 2.708 | 144.000 | 254 | 433.5 |
| Cxcl1 | 264 | 0.622 | 7 | 7 | 16.563 | 1 | 0.195 | 33.533 | 3.508 | 5.463 | 42 | 380.9 |
| Vcl | 32 | 0.366 | 7 | 7 | 13.630 | 2 | 0.325 | 35.667 | 3.721 | 40.857 | 184 | 326.6 |
| Igf1r | 60 | 0.437 | 8 | 8 | 18.338 | 1 | 0.243 | 36.250 | 3.735 | 16.566 | 158 | 310.6 |
| Cxcl2 | 144 | 0.618 | 6 | 6 | 15.224 | 1 | 0.195 | 32.533 | 3.455 | 3.446 | 24 | 236.5 |
| Nos2 | 120 | 0.648 | 5 | 5 | 14.359 | 1 | 0.195 | 32.533 | 3.481 | 0.000 | 0 | 182.2 |
| Alpl | 120 | 0.648 | 5 | 5 | 13.440 | 1 | 0.195 | 30.950 | 3.335 | 0.000 | 0 | 179.6 |
| Lif | 6 | 0.284 | 4 | 4 | 8.373 | 1 | 0.195 | 30.950 | 3.375 | 8.750 | 54 | 120.9 |
| Lepr | 3 | 0.308 | 2 | 3 | 5.928 | 1 | 0.195 | 29.033 | 3.228 | 10.854 | 46 | 104.5 |
| Ncoa1 | 18 | 0.454 | 5 | 5 | 12.255 | 1 | 0.195 | 30.867 | 3.335 | 3.541 | 18 | 97.6 |
| Lpl | 8 | 0.379 | 4 | 4 | 9.369 | 1 | 0.195 | 29.700 | 3.268 | 4.764 | 24 | 88.7 |
| Ccl7 | 24 | 0.568 | 4 | 4 | 10.557 | 1 | 0.195 | 30.033 | 3.295 | 0.000 | 0 | 77.6 |
| Bmpr1a | 4 | 0.309 | 3 | 3 | 9.961 | 1 | 0.243 | 33.750 | 3.668 | 0.944 | 8 | 67.9 |
| Npy | 2 | 0.000 | 1 | 2 | 5.035 | 1 | 0.195 | 26.783 | 3.054 | 2.546 | 16 | 59.6 |
| Dlx5 | 6 | 0.463 | 3 | 3 | 9.784 | 1 | 0.195 | 28.200 | 3.134 | 0.000 | 0 | 54.8 |
| Tuba1a | 6 | 0.463 | 3 | 3 | 7.557 | 1 | 0.195 | 28.783 | 3.214 | 0.000 | 0 | 53.2 |
| Mepe | 6 | 0.463 | 3 | 3 | 7.060 | 1 | 0.195 | 27.117 | 2.988 | 0.000 | 0 | 50.8 |
| Phex | 6 | 0.463 | 3 | 3 | 6.357 | 1 | 0.195 | 27.117 | 2.988 | 0.000 | 0 | 50.1 |
| Il11 | 2 | 0.308 | 2 | 2 | 6.291 | 1 | 0.195 | 28.367 | 3.201 | 0.000 | 0 | 45.4 |
| Ccn1 | 2 | 0.308 | 2 | 2 | 6.144 | 1 | 0.195 | 27.367 | 3.121 | 0.000 | 0 | 44.1 |
| Fabp4 | 2 | 0.308 | 2 | 2 | 5.034 | 1 | 0.195 | 27.367 | 3.121 | 0.000 | 0 | 43.0 |
| Bax | 2 | 0.308 | 2 | 2 | 4.735 | 1 | 0.195 | 26.700 | 3.041 | 0.000 | 0 | 42.0 |
| Bad | 2 | 0.308 | 2 | 2 | 3.746 | 1 | 0.195 | 26.700 | 3.041 | 0.000 | 0 | 41.0 |
| Gjc1 | 2 | 0.308 | 2 | 2 | 4.254 | 1 | 0.195 | 24.867 | 2.841 | 0.000 | 0 | 39.5 |
| Dlx1 | 1 | 0.000 | 1 | 1 | 4.529 | 1 | 0.195 | 27.200 | 3.108 | 0.000 | 0 | 39.0 |
| Ptgs1 | 1 | 0.000 | 1 | 1 | 4.145 | 1 | 0.195 | 25.950 | 3.001 | 0.000 | 0 | 37.3 |
| Ptger2 | 1 | 0.000 | 1 | 1 | 4.089 | 1 | 0.195 | 25.950 | 3.001 | 0.000 | 0 | 37.2 |

| Gene symbol | Local-based methods | | | | Global-based methods | | | | | | | Total score (SUMscore) |
|---|---|---|---|---|---|---|---|---|---|---|---|---|
| | MCC | DMNC | MNC | Degree | EPC | BottleNeck | EcCentricity | Closeness | Radiality | Betweenness | Stress | |
| Mmp14 | 1 | 0.000 | 1 | 1 | 4.012 | 1 | 0.195 | 25.450 | 2.948 | 0.000 | 0 | 36.6 |
| Pdpn | 1 | 0.000 | 1 | 1 | 3.458 | 1 | 0.195 | 25.450 | 2.948 | 0.000 | 0 | 36.1 |
| Piezo1 | 1 | 0.000 | 1 | 1 | 1.312 | 1 | 0.195 | 18.017 | 1.747 | 0.000 | 0 | 25.3 |
| Piezo2 | 0 | 0.000 | 0 | 0 | 1.000 | 0 | 0.000 | 0.000 | 0.000 | 0.000 | 0 | 1.0 |
| Eral1 | 0 | 0.000 | 0 | 0 | 1.000 | 0 | 0.000 | 0.000 | 0.000 | 0.000 | 0 | 1.0 |

## 7.4.2 GLay community clustering

### *7.4.2.1 GLay cluster #1 – Gene list enrichment*

| # Back-ground genes | # Genes | Fraction | Description | FDR value | Genes | p-value | Term name |
|---|---|---|---|---|---|---|---|
| **GO Biological Process** | | | | | | | |
| 243 | 15 | 0.062 | Wnt signaling pathway | 4.54E-21 | Sfrp4, Wnt3a, Fzd6, Wnt1, Dkk1, Lrp5, Lef1, Sfrp2, Dkk2, Sfrp1, Wnt4, Wnt5a, Axin1, Apc, Ccnd1 | 3.52E-25 | GO:0016055 |
| 79 | 12 | 0.152 | Canonical wnt signaling pathway | 1.39E-20 | Sfrp4, Wnt3a, Fzd6, Wnt1, Lrp5, Lef1, Sfrp2, Sfrp1, Wnt4, Wnt5a, Axin1, Apc | 3.24E-24 | GO:0060070 |
| 126 | 9 | 0.071 | Negative regulation of canonical wnt signaling pathway | 3.26E-12 | Sfrp4, Fzd6, Dkk1, Sfrp2, Dkk2, Sfrp1, Wnt5a, Axin1, Apc | 2.53E-15 | GO:0090090 |
| 133 | 7 | 0.053 | Regulation of embryonic development | 6.03E-09 | Wnt3a, Wnt1, Dkk1, Sfrp2, Sfrp1, Wnt4, Wnt5a | 3.84E-11 | GO:0045995 |
| 4 | 4 | 1.000 | Regulation of dermatome development | 6.57E-09 | Wnt3a, Wnt1, Sfrp2, Wnt4 | 4.23E-11 | GO:0061183 |
| 71 | 6 | 0.085 | Somitogenesis | 1.03E-08 | Wnt3a, Dkk1, Lef1, Sfrp2, Sfrp1, Wnt5a | 7.70E-11 | GO:0001756 |
| 31 | 5 | 0.161 | Axis elongation | 2.02E-08 | Wnt3a, Sfrp2, Sfrp1, Wnt5a, Esr1 | 1.73E-10 | GO:0003401 |
| 31 | 5 | 0.161 | Non-canonical wnt signaling pathway | 2.02E-08 | Fzd6, Sfrp2, Sfrp1, Wnt4, Wnt5a | 1.73E-10 | GO:0035567 |
| 9 | 4 | 0.444 | Regulation of dopaminergic neuron differentiation | 4.34E-08 | Wnt3a, Dkk1, Sfrp2, Sfrp1 | 4.31E-10 | GO:1904338 |
| 98 | 6 | 0.061 | Regulation of bmp signaling pathway | 4.77E-08 | Sfrp4, Wnt1, Dkk1, Sfrp2, Sfrp1, Wnt5a | 4.85E-10 | GO:0030510 |
| 99 | 6 | 0.061 | Epithelial cell proliferation | 5.02E-08 | Bad, Bax, Mki67, Wnt5a, Esr1, Ccnd1 | 5.14E-10 | GO:0050673 |
| 105 | 6 | 0.057 | Regulation of fibroblast proliferation | 6.59E-08 | Wnt1, Ccna2, Bax, Sfrp1, Wnt5a, Esr1 | 7.21E-10 | GO:0048145 |
| 113 | 6 | 0.053 | Regulation of morphogenesis of an epithelium | 9.84E-08 | Fzd6, Sfrp2, Sfrp1, Wnt4, Wnt5a, Esr1 | 1.10E-09 | GO:1905330 |
| 53 | 5 | 0.094 | Negative regulation of bmp signaling pathway | 1.71E-07 | Wnt1, Dkk1, Sfrp2, Sfrp1, Wnt5a | 2.07E-09 | GO:0030514 |
| 62 | 5 | 0.081 | Stem cell proliferation | 3.29E-07 | Wnt3a, Wnt1, Lef1, Sfrp2, Wnt5a | 4.34E-09 | GO:0072089 |
| **KEGG Pathways** (Excluded: mmu05224, mmu05225, mmu05226, mmu05217, mmu04934, mmu05210, mmu05213) | | | | | | | |
| 157 | 15 | 0.096 | Wnt signaling pathway | 2.21E-25 | Sfrp4, Wnt3a, Fzd6, Wnt1, Dkk1, Lrp5, Lef1, Sfrp2, Dkk2, Sfrp1, Wnt4, Wnt5a, Axin1, Apc, Ccnd1 | 6.64E-28 | mmu04310 |
| 156 | 9 | 0.058 | Hippo signaling pathway | 5.88E-13 | Wnt3a, Fzd6, Wnt1, Lef1, Wnt4, Wnt5a, Axin1, Apc, Ccnd1 | 1.59E-14 | mmu04390 |
| 137 | 7 | 0.051 | Signaling pathways regulating pluripotency of stem cells | 1.11E-09 | Wnt3a, Fzd6, Wnt1, Wnt4, Wnt5a, Axin1, Apc | 4.68E-11 | mmu04550 |
| 95 | 6 | 0.063 | Melanogenesis | 8.98E-09 | Wnt3a, Fzd6, Wnt1, Lef1, Wnt4, Wnt5a | 4.06E-10 | mmu04916 |
| **WikiPathways** | | | | | | | |
| 106 | 13 | 0.123 | Wnt signaling pathway (NetPath) | 6.44E-23 | Wnt3a, Fzd6, Wnt1, Dkk1, Lrp5, Lef1, Sfrp2, Sfrp1, Wnt4, Wnt5a, Axin1, Apc, Ccnd1 | 3.25E-25 | WP539 |
| 96 | 10 | 0.104 | Wnt signaling pathway and pluripotency | 1.41E-16 | Wnt3a, Fzd6, Wnt1, Lrp5, Lef1, Wnt4, Wnt5a, Axin1, Apc, Ccnd1 | 1.43E-18 | WP723 |
| 59 | 9 | 0.153 | Wnt signaling pathway | 2.72E-16 | Sfrp4, Wnt3a, Fzd6, Wnt1, Wnt4, Wnt5a, Axin1, Apc, Ccnd1 | 4.12E-18 | WP403 |
| 41 | 8 | 0.195 | Wnt signaling in kidney disease | 3.19E-15 | Wnt3a, Fzd6, Wnt1, Lrp5, Wnt4, Wnt5a, Axin1, Apc | 6.44E-17 | WP3857 |
| 116 | 8 | 0.069 | ESC pluripotency pathways | 5.99E-12 | Wnt3a, Fzd6, Wnt1, Lrp5, Wnt4, Wnt5a, Axin1, Apc | 1.51E-13 | WP339 |

## 7.4.2.2 GLay cluster #2 – Gene list enrichment

| # Back-ground genes | # Genes | Fraction | Description | FDR value | Genes | p-value | Term name |
|---|---|---|---|---|---|---|---|
| **GO Biological Process** | | | | | | | |
| 62 | 8 | 0.129 | Regulation of bone remodeling | 3.34E-11 | Itgb3, Tnfsf11, Il6, Itgav, Lepr, Tnfrsf11b, Spp1, Vegfa | 8.80E-14 | GO:0046850 |
| 149 | 9 | 0.060 | Regulation of peptidyl-serine phosphorylation | 2.76E-10 | Tgfb1, Cd44, Tnf, Il6, Hgf, Ptgs2, Lif, Il11, Vegfa | 1.30E-12 | GO:0033135 |
| 52 | 7 | 0.135 | Regulation of bone resorption | 5.09E-10 | Itgb3, Tnfsf11, Il6, Itgav, Tnfrsf11b, Spp1, Vegfa | 2.72E-12 | GO:0045124 |
| 166 | 9 | 0.054 | Regulation of smooth muscle cell proliferation | 5.96E-10 | Pparg, Igf1r, Itgb3, Tnf, Il6, Ptgs2, Igf1, Jun, Vegfa | 3.28E-12 | GO:0048660 |
| 175 | 9 | 0.051 | Positive regulation of leukocyte differentiation | 8.54E-10 | Tgfb1, Itgb3, Fos, Tnfsf11, Tnf, Il6, Lif, Mmp14, Jun | 5.17E-12 | GO:1902107 |
| 109 | 8 | 0.073 | Positive regulation of smooth muscle cell proliferation | 9.76E-10 | Igf1r, Itgb3, Tnf, Il6, Ptgs2, Igf1, Jun, Vegfa | 6.06E-12 | GO:0048661 |
| 115 | 8 | 0.070 | Positive regulation of peptidyl-serine phosphorylation | 1.38E-09 | Tgfb1, Cd44, Tnf, Il6, Ptgs2, Lif, Il11, Vegfa | 9.10E-12 | GO:0033138 |
| 68 | 7 | 0.103 | Positive regulation of myeloid leukocyte differentiation | 2.21E-09 | Tgfb1, Itgb3, Fos, Tnfsf11, Tnf, Lif, Jun | 1.56E-11 | GO:0002763 |
| 90 | 7 | 0.078 | Negative regulation of cysteine-type endopeptidase activity involved in apoptotic process | 1.04E-08 | Cd44, Tnf, Il6, Hgf, Ptgs2, Igf1, Vegfa | 9.86E-11 | GO:0043154 |
| 115 | 7 | 0.061 | Positive regulation of protein kinase b signaling | 4.40E-08 | Tgfb1, Igf1r, Tnfsf11, Tnf, Il6, Igf1, Vegfa | 5.01E-10 | GO:0051897 |
| 118 | 7 | 0.059 | Tissue remodeling | 5.11E-08 | Tgfb1, Tnfsf11, Il6, Igf1, Lif, Mmp14, Vegfa | 5.94E-10 | GO:0048771 |
| 86 | 6 | 0.070 | Positive regulation of gliogenesis | 3.03E-07 | Pparg, Tgfb1, Tnf, Il6, Igf1, Lif | 4.49E-09 | GO:0014015 |
| 95 | 6 | 0.063 | Regulation of blood vessel endothelial cell migration | 5.09E-07 | Pparg, Tgfb1, Tnf, Ptgs2, Igf1, Vegfa | 7.90E-09 | GO:0043535 |
| 47 | 5 | 0.106 | Regulation of acute inflammatory response | 8.24E-07 | Pparg, Tnfsf11, Tnf, Il6, Ptgs2 | 1.34E-08 | GO:0002673 |
| 113 | 6 | 0.053 | Myeloid leukocyte migration | 1.23E-06 | Ccl7, Tnfsf11, Cxcl1, Cxcl2, Spp1, Vegfa | 2.12E-08 | GO:0097529 |
| **KEGG Pathways** (Excluded: mmu05140, mmu05414, mmu05142, mmu05146, mmu05133) | | | | | | | |
| 86 | 10 | 0.116 | Rheumatoid arthritis | 4.22E-14 | Tgfb1, Fos, Tnfsf11, Tnf, Il6, Cxcl1, Cxcl2, Il11, Jun, Vegfa | 1.27E-16 | mmu05323 |
| 112 | 9 | 0.080 | TNF signaling pathway | 1.26E-11 | Fos, Tnf, Il6, Cxcl1, Ptgs2, Lif, Cxcl2, Mmp14, Jun | 1.14E-13 | mmu04668 |
| 199 | 10 | 0.050 | Proteoglycans in cancer | 2.36E-11 | Tgfb1, Cd44, Igf1r, Itgb3, Tnf, Itgav, Hgf, Igf1, Actb, Vegfa | 3.56E-13 | mmu05205 |
| 90 | 8 | 0.089 | IL-17 signaling pathway | 7.89E-11 | Ccl7, Fos, Tnf, Il6, Cxcl1, Ptgs2, Cxcl2, Jun | 1.43E-12 | mmu04657 |
| 121 | 8 | 0.066 | Osteoclast differentiation | 6.35E-10 | Pparg, Tgfb1, Itgb3, Fos, Tnfsf11, Tnf, Tnfrsf11b, Jun | 1.34E-11 | mmu04380 |
| 88 | 7 | 0.080 | Hypertrophic cardiomyopathy | 3.13E-09 | Tgfb1, Itgb3, Tnf, Il6, Itgav, Igf1, Actb | 8.50E-11 | mmu05410 |
| 78 | 5 | 0.064 | EGFR tyrosine kinase inhibitor resistance | 2.19E-06 | Igf1r, Il6, Hgf, Igf1, Vegfa | 1.45E-07 | mmu01521 |
| 87 | 5 | 0.057 | Viral protein interaction with cytokine and cytokine receptor | 3.51E-06 | Ccl7, Tnf, Il6, Cxcl1, Cxcl2 | 2.43E-07 | mmu04061 |
| 91 | 5 | 0.055 | Hematopoietic cell lineage | 4.17E-06 | Cd44, Itgb3, Tnf, Il6, Il11 | 3.01E-07 | mmu04640 |
| 98 | 5 | 0.051 | Toll-like receptor signaling pathway | 5.69E-06 | Fos, Tnf, Il6, Jun, Spp1 | 4.28E-07 | mmu04620 |
| 99 | 5 | 0.051 | NF-kappa B signaling pathway | 5.74E-06 | Tnfsf11, Tnf, Cxcl1, Ptgs2, Cxcl2 | 4.50E-07 | mmu04064 |
| **WikiPathways** (Excluded: WP2432) | | | | | | | |
| 132 | 9 | 0.068 | Adipogenesis genes | 9.14E-11 | Pparg, Tgfb1, Lpl, Tnf, Il6, Fabp4, Igf1, Lif, Ncoa1 | 4.62E-13 | WP447 |
| 61 | 7 | 0.115 | Lung fibrosis | 7.59E-10 | Tgfb1, Tnf, Il6, Hgf, Igf1, Cxcl2, Spp1 | 7.67E-12 | WP3632 |
| 50 | 6 | 0.120 | TGF-beta signaling pathway | 1.22E-08 | Tgfb1, Fos, Tnf, Lif, Jun, Spp1 | 2.12E-10 | WP113 |
| 27 | 5 | 0.185 | Cytokines and inflammatory response | 3.51E-08 | Tgfb1, Tnf, Il6, Cxcl1, Il11 | 1.06E-09 | WP222 |
| 14 | 4 | 0.286 | Osteoclast signaling | 3.52E-07 | Itgb3, Tnfsf11, Tnfrsf11b, Spp1 | 1.25E-08 | WP454 |
| 62 | 5 | 0.081 | Endochondral ossification | 1.08E-06 | Tgfb1, Igf1r, Igf1, Spp1, Vegfa | 4.91E-08 | WP1270 |
| 82 | 5 | 0.061 | MicroRNAs in cardiomyocyte hypertrophy | 3.64E-06 | Tgfb1, Igf1r, Tnf, Igf1, Lif | 1.84E-07 | WP1560 |

### 7.4.2.3 GLay cluster #3 – Gene list enrichment

| # Back-ground genes | # Genes | Fraction | Description | FDR value | Genes | p-value | Term name |
|---|---|---|---|---|---|---|---|
| GO Biological Process | | | | | | | |
| | | | None satisfying applied criteria. | | | | |
| KEGG Pathways | | | | | | | |
| | | | None satisfying applied criteria. | | | | |
| WikiPathways | | | | | | | |
| | | | None satisfying applied criteria. | | | | |

### 7.4.2.4 GLay cluster #4 – Gene list enrichment

| # Back-ground genes | # Genes | Fraction | Description | FDR value | Genes | p-value | Term name |
|---|---|---|---|---|---|---|---|
| GO Biological Process | | | | | | | |
| 92 | 5 | 0.054 | Biomineral tissue development | 2.37E-06 | Alpl, Mepe, Dmp1, Bglap, Phex | 3.67E-10 | GO:0031214 |
| KEGG Pathways | | | | | | | |
| | | | None satisfying applied criteria. | | | | |
| WikiPathways | | | | | | | |
| | | | None satisfying applied criteria. | | | | |
