# Supplementary material for: Fluid flow shear stress and tissue remodeling—an orthodontic perspective: evidence synthesis and differential gene expression network analysis
Source: Front Bioeng Biotechnol. 2023 Sep 18;11:1256825. doi: 10.3389/fbioe.2023.1256825 (PMC10545883; doi:10.3389/fbioe.2023.1256825)
Supplement: Supplementary file 2 [file DataSheet2.pdf]

Supplement 2

"Fluid Flow Shear Stress and Tissue Remodeling – an Orthodontic Perspective: Evidence Synthesis and Differential Gene Expression Network analysis"

Results from the Data Extraction

Table of contents

2.1 HUMAN MESENCHYMAL STEM CELLS 2

2.2 HUMAN OSTEOBLASTS 14

2.3 HUMAN OSTEOCYTES 19

2.4 HUMAN PERIODONTAL LIGAMENT CELLS 20

2.5 MOUSE OSTEOBLASTS 24

2.6 MOUSE OSTEOCYTES 30

2.1 Human mesenchymal stem cells

Reference	Gene or analyte[a]	Official gene symbol or abbreviation[b]	Cell type (age/number and sex of donor (health status), tooth type, isolation method, passages used, cell density/confluency)[a,c]	Flow type (steady laminar, pulsatile laminar, or oscillatory laminar)[a,d]	FSS duration and frequency (Hz)[a]	FSS magnitude[a]	FSS apparatus[a]	Gene expression: increase, decrease, no change (method w/ reference gene); methods: (RT-qPCR, sqPCR)[f]	Gene expression: when it reaches peak and peak's magnitude (fold change; relative gene expression; times or ratio; unclear = ?)[f]	Protein/metabolite expression: increase, decrease, no change (method w/reference); methods: ELISA, WB, RIA, EMSA, IF[f]	Protein/metabolite expression: when it reaches peak and peak's magnitude (times or ratio; unclear = ?)[f]	Remarks
Riddle et al. (2007)	Ca^{2+}	Calcium	Human BMSCs (Cambrex Biosciences) (18y/ 1M (n.g.), n.g., n.g., P3-8, 130000 cells or 80000 cells/ n.g.)	Oscillatory laminar	180s/1Hz	5dyn/cm^2,<br>10dyn/cm^2,<br>20dyn/cm^2	Custom-made			Fluctuated increase (Fura-2 microscopy)		
Riddle et al. (2007)	Calcineurin	RCAN1	Human BMSCs (Cambrex Biosciences) (18y/ 1M (n.g.), n.g., n.g., P3-8, 130000 cells or 80000 cells/ n.g.)	Oscillatory laminar	5min/1Hz	5dyn/cm^2,<br>10dyn/cm^2,<br>20dyn/cm^2	Custom-made			Increase (colorimetric assay)	5min: 789.3 (pmol P/mg protein); 789.3/309.3 = 2.55 (ratio-calc)†	
Yourek et al. (2010)	ALP	ALP (unspecific)	Bone marrow hMSCs (20-26y/7M and 1F (Healthy), n.g., Ficoll paque, P3-5, 7.6–8.6×10^3 cells per cm^2/ n.g.) ("hMSC")	Steady laminar	24h: immediately after FSS and 3d, 7d, 8d, 11d post-FSS/n.g.	9dyn/cm^2	Custom-made			cellular ALP @ 7d post-FSS: increase (activity)<br>cellular ALP @ 8d post-FSS: decrease (activity)<br>cellular ALP @ 11d post-FSS: decrease (activity)	hMSCs cellular ALP @ 7d: 14.5mU/ng; 14.5/14.2 = 1.02 (ratio-calc)†	
Yourek et al. (2010)	BMP-2	BMP2	Bone marrow hMSCs (20-26y/7M and 1F (Healthy), n.g., Ficoll paque, P3-5, 7.6–8.6×10^3 cells per cm^2/ n.g.) ("hMSC")	Steady laminar	24h (after 3d or 7d pre-culture in basal medium)/n.g.	4dyn/cm^2,<br>15dyn/cm^2,<br>22dyn/cm^2	Custom-made	4dyn/cm^2: increase (RT-qPCR, GAPDH)<br>15dyn/cm^2: increase (RT-qPCR, GAPDH)<br>22dyn/cm^2: increase (RT-qPCR, GAPDH)	4dyn/cm^2 @ 7d pre-FSS: 5.3 (FC)†<br>15dyn/cm^2 @ 7d pre-FSS: 7.0 (FC)†<br>22dyn/cm^2 @ 7d pre-FSS: 7.2 (FC)†			
Yourek et al. (2010)	BSP	IBSP	Bone marrow hMSCs (20-26y/7M and 1F (Healthy), n.g., Ficoll paque, P3-5, 7.6–8.6×10^3 cells per cm^2/ n.g.) ("hMSC")	Steady laminar	24h (after 3d or 7d pre-culture in basal medium)/n.g.	4dyn/cm^2,<br>15dyn/cm^2,<br>22dyn/cm^2	Custom-made	4dyn/cm^2: increase (RT-qPCR, GAPDH)<br>15dyn/cm^2: increase (RT-qPCR, GAPDH)<br>22dyn/cm^2: increase (RT-qPCR, GAPDH)	4dyn/cm^2 @ 7d pre-FSS: 5.3 (FC)†<br>15dyn/cm^2 @ 7d pre-FSS: 10.1 (FC)†<br>22dyn/cm^2 @ 7d pre-FSS: 8.9 (FC)†			
Yourek et al. (2010)	OP	SPP1	Bone marrow hMSCs (20-26y/7M and 1F (Healthy), n.g., Ficoll paque, P3-5, 7.6–8.6×10^3 cells per cm^2/ n.g.) ("hMSC")	Steady laminar	24h (after 3d or 7d pre-culture in basal medium)/n.g.	4dyn/cm^2,<br>15dyn/cm^2,<br>22dyn/cm^2	Custom-made	4dyn/cm^2: increase (RT-qPCR, GAPDH)<br>15dyn/cm^2: increase (RT-qPCR, GAPDH)<br>22dyn/cm^2: increase (RT-qPCR, GAPDH)	4dyn/cm^2 @ 7d pre-FSS: 8.3 (FC)†<br>15dyn/cm^2 @ 7d pre-FSS: 9.6(FC)†<br>22dyn/cm^2 @ 7d pre-FSS: 9.5(FC)†			
Kraft et al. (2011)	COX-1	PTGS1	Human Dental Pulp Cells (21y/ 1M (healthy), M, dig., P5, cell 4×10^5 cells/ n.g.)	Pulsatile laminar	1h FSS (0h, 1h, 3h post-FSS incubation)/5Hz	0.6Pa, a pulse amplitude of 0.3Pa and a peak shear stress rate of 8.4Pa/s	Custom-made	Post-FSS incubation: no change (RT-qPCR, GUS)	No change			
Kraft et al. (2011)	COX-2	PTGS2	Human Dental Pulp Cells (21y/ 1M (healthy), M, dig., P5, cell 4×10^5 cells/ n.g.)	Pulsatile laminar	1h FSS (0h, 1h, 3h post-FSS incubation)/5Hz	0.6Pa, a pulse amplitude of 0.3Pa and a peak shear stress rate of 8.4Pa/s	Custom-made	Post-FSS incubation: increase (RT-qPCR, GUS)	3h post-FSS: 5.0141 (FC)†			
Kraft et al. (2011)	NO	Nitric oxide	Human Dental Pulp Cells (21y/ 1M (healthy), M, dig., P5, cell 4×10^5 cells/ n.g.)	Pulsatile laminar	5min, 10min, 15min, 30min, 60min FSS/5Hz	0.6Pa, a pulse amplitude of 0.3Pa and a peak shear stress rate of 8.4Pa/s	Custom-made			Increase (Griess, NO_2^-)	60min FSS: 3.7313 (ratio)†	

[a] Entry provided as reported in the given study.
[b] Human genes were confirmed with the HUGO Gene Nomenclature Committee (HGNC; URL: https://www.genenames.org); mouse genes were confirmed with the Mouse Genome Informatics (MGI; URL: https://www.informatics.jax.org/genes.shtml) after checking the specificity of primers with Primer-BLAST.
[c] Sex of donors: "M" – male, "F" – female; Tooth type: "PM" – premolar, "M" – molar; dig. Indicate isolation by cell digestion; Exp. indicate isolation by cell explant; Cell density: given in cells/cm^2 if not otherwise mentioned.
[d] Flow type deduced from the description of the FSS apparatus given by the authors.
[f] RT-qPCR (reverse-transcriptase quantitative polymerase chain reaction); sqPCR (semi-quantitative polymerase chain reaction); ELISA (enzyme-linked immuno absorbent assay); WB (western blotting); RIA (radioimmunoassay); EMSA (electromobility shift assay); IF (immunofluorescence)

rel.: indicate relative gene expression. Is entitled to percentages or gene expression ratios normalized to control, and not calculated by ΔΔCT.
FC: indicate fold change. When Author mentions the use of ΔΔCT or the method according to Livak & Schmittken (2004) in calculating FC.
n.g.: not given. For information not given by study-authors.
† Information derived from figures using Engauge Digitizer.
* Indicate manual calculations by measuring the graphs, without using the Engauge Digitizer.
ratio-calc: indicate manual calculation by dividing intervention/control = result (ratio-calc)
ratio: indicate ratios given by study-authors such as normalization to control in case of small molecules data or in case of gene expression ratios, e.g. ratio of RANKL/OPG or Bcl-2/Bax.

Reference	Gene or analyte[a]	Official gene symbol or abbreviation[b]	Cell type (age/number and sex of donor (health status), tooth type, isolation method, passages used, cell density/confluency)[a,c]	Flow type (steady laminar, pulsatile laminar, or oscillatory laminar)[a,d]	FSS duration and frequency (Hz)[a]	FSS magnitude[a]	FSS apparatus[a]	Gene expression: increase, decrease, no change (method w/ reference gene); methods: (RT-qPCR, sqPCR)[f]	Gene expression: when it reaches peak and peak's magnitude (fold change; relative gene expression; times or ratio; unclear = ?)[f]	Protein/metabolite expression: increase, decrease, no change (method w/reference); methods: ELISA, WB, RIA, EMSA, IF[f]	Protein/metabolite expression: when it reaches peak and peak's magnitude (times or ratio; unclear = ?)[f]	Remarks
Kraft et al. (2011)	NO	Nitric oxide	Human Dental Pulp Cells (21y/ 1M (healthy), M, dig., P5, cell 4×10^5 cells/ n.g.)	Pulsatile laminar	1h FSS (1h, 3h post-FSS incubation)/5Hz	0.6Pa, a pulse amplitude of 0.3Pa and a peak shear stress rate of 8.4Pa/s	Custom-made			Decrease then increase (Griess, NO_2^-)	1h post-FSS: 0.78195 (ratio)† 3h post-FSS: 1.53383 (ratio)†	
Kraft et al. (2011)	PGE2	PGE2	Human Dental Pulp Cells (21y/ 1M (healthy), M, dig., P5, cell 4×10^5 cells/ n.g.)	Pulsatile laminar	5min, 10min, 15min, 30min, 60min FSS/5Hz	0.6Pa, a pulse amplitude of 0.3Pa and a peak shear stress rate of 8.4Pa/s	Custom-made			Temporary decrease followed by temporary increase then increase with plateau (ELISA)	5min FSS: 0.84 (ratio)† 10min FSS:1.2 (ratio) † 30min FSS: 1.31 (ratio) † 60min FSS: 1.32 (ratio)†	
Kraft et al. (2011)	PGE2	PGE2	Human Dental Pulp Cells (21y/ 1M (healthy), M, dig., P5, cell 4×10^5 cells/ n.g.)	Pulsatile laminar	1h FSS (1h, 3h post-FSS incubation)/5Hz	0.6Pa, a pulse amplitude of 0.3Pa and a peak shear stress rate of 8.4Pa/s	Custom-made			Increase (ELISA)	1h post-FSS: 5.7556 (ratio)†	
Sonam et al. (2016)	ALPL	ALPL	hMSC (Lonza) (n.g./ n.g. (n.g.), n.g., n.g., P1-5, 1000 cells per cm^2 / n.g.)	Steady laminar	48h/n.g.	1Pa	Custom-made (planar topography)	Increase (RT-qPCR; GAPDH)	48h: 3.5 (rel.)†	Increase (IF)	48h: 2.7 (ratio)†	
Sonam et al. (2016)	CD44	CD44	hMSC (Lonza) (n.g./ n.g. (n.g.), n.g., n.g., P1-5, 1000 cells per cm^2 / n.g.)	Steady laminar	48h/n.g.	1Pa	Custom-made (planar topography)	Increase (RT-qPCR; GAPDH)	48h: 0.7 (rel.)†	Increase (IF)	48h: 0.6 (ratio)†	
Sonam et al. (2016)	ENG	ENG	hMSC (Lonza) (n.g./ n.g. (n.g.), n.g., n.g., P1-5, 1000 cells per cm^2 / n.g.)	Steady laminar	48h/n.g.	1Pa	Custom-made (planar topography)	Increase (RT-qPCR; GAPDH)	48h: 0.7 (rel.)†	Increase (IF)	48h: 0.8 (ratio)†	
Sonam et al. (2016)	non-muscle myosin II A	MYH2	hMSC (Lonza) (n.g./ n.g. (n.g.), n.g., n.g., P1-5, 1000 cells per cm^2 / n.g.)	Steady laminar	48h/n.g.	1Pa	Custom-made (planar topography)	Increase (RT-qPCR; GAPDH)	48h: 3.3 (rel.)†	Increase (IF)	48h: 2.5 (ratio)†	
Sonam et al. (2016)	OCN	BGLAP	hMSC (Lonza) (n.g./ n.g. (n.g.), n.g., n.g., P1-5, 1000 cells per cm^2 / n.g.)	Steady laminar	48h/n.g.	1Pa	Custom-made (planar topography)	Increase (RT-qPCR; GAPDH)	48h: 3.1 (rel.)†	Increase (IF)	48h: 2.6 (ratio)†	
Sonam et al. (2016)	OPN	SPP1	hMSC (Lonza) (n.g./ n.g. (n.g.), n.g., n.g., P1-5, 1000 cells per cm^2 / n.g.)	Steady laminar	48h/n.g.	1Pa	Custom-made (planar topography)	Increase (RT-qPCR; GAPDH)	48h: 3.4 (rel.)†	Increase (IF)	48h: 2.4 (ratio)†	
Sonam et al. (2016)	Rho A	RHOA	hMSC (Lonza) (n.g./ n.g. (n.g.), n.g., n.g., P1-5, 1000 cells per cm^2 / n.g.)	Steady laminar	48h/n.g.	1Pa	Custom-made (planar topography)	Increase (RT-qPCR; GAPDH)	48h: 2.5 (rel.)†	Increase (IF)	48h: 3 (ratio)†	
Sonam et al. (2016)	RUNX2	RUNX2	hMSC (Lonza) (n.g./ n.g. (n.g.), n.g., n.g., P1-5, 1000 cells per cm^2 / n.g.)	Steady laminar	48h/n.g.	1Pa	Custom-made (planar topography)	Increase (RT-qPCR; GAPDH)	48h: 2.9 (rel.)†	Increase (IF)	48h: 3.1 (ratio)†	
Sonam et al. (2016)	THY1	THY1	hMSC (Lonza) (n.g./ n.g. (n.g.), n.g., n.g., P1-5, 1000 cells per cm^2 / n.g.)	Steady laminar	48h/n.g.	1Pa	Custom-made (planar topography)	Increase (RT-qPCR; GAPDH)	48h: 0.8 (rel.)†	Increase (IF)	48h: 0.8 (ratio)†	
Hu et al. (2017)	Adiponectin	ADIPOQ	Bone marrow cells from posterior iliac crests (MSCs) (n.g./ n.g. (healthy), n.g., n.g., P3-5, n.g./ n.g.)	Steady laminar	1h, 3h, 6h, 12h, 24h, 72h/n.g.	12dyn/cm^2	FlexFlo™ chamber (FlexCell Int.)	Temporary decrease, temporary increase then decrease (RT-qPCR; GAPDH)	3h: 1.0/1.1 = 0.9 (ratio-calc)† 12h: 1.0/0.9 = 1.1 (ratio-calc)† 72h: 0.7/1.1 = 0.6 (ratio-calc)†			
Hu et al. (2017)	Ca^{2+}	Calcium	Bone marrow cells from posterior iliac crests (MSCs) (n.g./ n.g. (healthy), n.g., n.g., P3-5, n.g./ n.g.)	Steady laminar	120s FSS/n.g.	12dyn/cm^2	FlexFlo™ chamber (FlexCell Int.)			Fluctuated increase (fura-2 microscopy)		
Hu et al. (2017)	Calcineurin	RCAN1	Bone marrow cells from posterior iliac crests (MSCs) (n.g./ n.g. (healthy), n.g., n.g., P3-5, n.g./ n.g.)	Steady laminar	1h, 3h, 6h, 12h, 24h, 72h/n.g.	12dyn/cm^2	FlexFlo™ chamber (FlexCell Int.)			Increase (WB, GAPDH)	No quantitative information given.	

[a] Entry provided as reported in the given study.
[b] Human genes were confirmed with the HUGO Gene Nomenclature Committee (HGNC; URL: https://www.genenames.org); mouse genes were confirmed with the Mouse Genome Informatics (MGI; URL: https://www.informatics.jax.org/genes.shtml) after checking the specificity of primers with Primer-BLAST.
[c] Sex of donors: "M" – male, "F" – female; Tooth type: "PM" – premolar, "M" – molar; dig. Indicate isolation by cell digestion; Exp. indicate isolation by cell explant; Cell density: given in cells/cm^2 if not otherwise mentioned.
[d] Flow type deduced from the description of the FSS apparatus given by the authors.
f RT-qPCR (reverse-transcriptase quantitative polymerase chain reaction); sqPCR (semi-quantitative polymerase chain reaction); ELISA (enzyme-linked immuno absorbent assay); WB (western blotting); RIA (radioimmunoassay); EMSA (electromobility shift assay); IF (immunofluorescence)

rel.: indicate relative gene expression. Is entitled to percentages or gene expression ratios normalized to control, and not calculated by ΔΔCT.
FC: indicate fold change. When Author mentions the use of ΔΔCT or the method according to Livak & Schmittken (2004) in calculating FC.
n.g.: not given. For information not given by study-authors.
† Information derived from figures using Engauge Digitizer.
* Indicate manual calculations by measuring the graphs, without using the Engauge Digitizer.
ratio-calc: indicate manual calculation by dividing intervention/control = result (ratio-calc)
ratio: indicate ratios given by study-authors such as normalization to control in case of small molecules data or in case of gene expression ratios, e.g. ratio of RANKL/OPG or Bcl-2/Bax.

Reference	Gene or analyte[a]	Official gene symbol or abbreviation[b]	Cell type (age/number and sex of donor (health status), tooth type, isolation method, passages used, cell density/confluency)[a,c]	Flow type (steady laminar, pulsatile laminar, or oscillatory laminar)[a,d]	FSS duration and frequency (Hz)[a]	FSS magnitude[a]	FSS apparatus[a]	Gene expression: increase, decrease, no change (method w/ reference gene); methods: (RT-qPCR, sqPCR)[f]	Gene expression: when it reaches peak and peak's magnitude (fold change; relative gene expression; times or ratio; unclear = ?)[f]	Protein/metabolite expression: increase, decrease, no change (method w/reference); methods: ELISA, WB, RIA, EMSA, IF[f]	Protein/metabolite expression: when it reaches peak and peak's magnitude (times or ratio; unclear = ?)[f]	Remarks
Hu et al. (2017)	NFATc1	NFATC1	Bone marrow cells from posterior iliac crests (MSCs) (n.g./ n.g. (healthy), n.g., n.g., P3-5, n.g./ n.g.)	Steady laminar	1h, 3h, 6h, 12h, 24h, 72h/n.g.	12dyn/cm²	FlexFlo™ chamber (FlexCell Int.)			Increase (WB, GAPDH)	No quantitative information given.	
Hu et al. (2017)	Ocn	BGLAP	Bone marrow cells from posterior iliac crests (MSCs) (n.g./ n.g. (healthy), n.g., n.g., P3-5, n.g./ n.g.)	Steady laminar	1h, 3h, 6h, 12h, 24h, 72h/n.g.	12dyn/cm²	FlexFlo™ chamber (FlexCell Int.)	Increase, temporary decrease then Increase (RT-qPCR; GAPDH)	1h: 1.2/1 = 1.2 (ratio-calc)†<br>3h: 1.1/1.2 = 0.91 (ratio-calc)†<br>6h: 1.3/1.2 = 1.1 (ratio-calc)†			
Hu et al. (2017)	Osx	SP7	Bone marrow cells from posterior iliac crests (MSCs) (n.g./ n.g. (healthy), n.g., n.g., P3-5, n.g./ n.g.)	Steady laminar	1h, 3h, 6h, 12h, 24h, 72h/n.g.	12dyn/cm²	FlexFlo™ chamber (FlexCell Int.)	Increase (RT-qPCR; GAPDH)	72h: 2.9/1.2 = 2.4 (ratio-calc)†			
Hu et al. (2017)	Trpv1	TRPV1	Bone marrow cells from posterior iliac crests (MSCs) (n.g./ n.g. (healthy), n.g., n.g., P3-5, n.g./ n.g.)	Steady laminar	1h, 3h, 6h, 12h, 24h, 72h/n.g.	12dyn/cm²	FlexFlo™ chamber (FlexCell Int.)	Increase (RT-qPCR; GAPDH)	3h: 1.1/0.9 = 1.2 (ratio-calc)†	Increase (WB, GAPDH)	3h: 1.1 (ratio-calc)*	
Hu et al. (2017)	Trpv4	TRPV4	Bone marrow cells from posterior iliac crests (MSCs) (n.g./ n.g. (healthy), n.g., n.g., P3-5, n.g./ n.g.)	Steady laminar	1h, 3h, 6h, 12h, 24h, 72h/n.g.	12dyn/cm²	FlexFlo™ chamber (FlexCell Int.)	Increase followed by plateau (RT-qPCR; GAPDH)	3h: 3.1/1.1 = 2.8 (ratio-calc) †<br>12h: 3.5/1.2 = 2.9 (ratio-calc)†	Increase (WB, GAPDH)	3h: 3.2 (ratio-calc)*	
Li et al. (2004)	ALP	ALP (unspecific)	MSCs (BioWhittaker) (n.g./ n.g. (healthy), n.g., Ficoll paque, P2-8, n.g./ n.g.)	Oscillatory laminar	2h FSS (3d post FSS incubation) /1Hz	10dyn/cm²	Custom-made			Decrease (colorimetric assay)	3d post-FSS: 0.02µg/mL; 0.02/0.03 = 0.66 (ratio-calc)†	ALP activity with p-nitrophenol substrate; unspecific!
Li et al. (2004)	Ca^{2+}	Calcium	MSCs (BioWhittaker) (n.g./ n.g. (healthy), n.g., Ficoll paque, P2-8, n.g./ n.g.)	Oscillatory laminar	2h FSS (24h post-FSS incubation)/1Hz	10dyn/cm²	Custom-made			Fluctuated increase (fura-2 microscopy)		
Li et al. (2004)	Cbfa1	RUNX2	MSCs (BioWhittaker) (n.g./ n.g. (healthy), n.g., Ficoll paque, P2-8, n.g./ n.g.)	Oscillatory laminar	2h FSS (24h post-FSS)/1Hz	10dyn/cm²	Custom-made	Decrease (RT-qPCR, GAPDH)	24h post-FSS: 0.9 (rel.)†			
Li et al. (2004)	Col-1	COL1A1	MSCs (BioWhittaker) (n.g./ n.g. (healthy), n.g., Ficoll paque, P2-8, n.g./ n.g.)	Oscillatory laminar	2h FSS (24h post-FSS incubation)/1Hz	10dyn/cm²	Custom-made	Increase (RT-qPCR, GAPDH)	24h post-FSS: 1.1 (rel.)†			
Li et al. (2004)	OP	SPP1	MSCs (BioWhittaker) (n.g./ n.g. (healthy), n.g., Ficoll paque, P2-8, n.g./ n.g.)	Oscillatory laminar	2h FSS (24h post-FSS incubation)/1Hz	10dyn/cm²	Custom-made	Increase (RT-qPCR, GAPDH)	24h post-FSS: 1.6 (rel.)†			
Li et al. (2004)	OSTC	BGALP	MSCs (BioWhittaker) (n.g./ n.g. (healthy), n.g., Ficoll paque, P2-8, n.g./ n.g.)	Oscillatory laminar	2h FSS (24h post-FSS incubation)/1Hz	10dyn/cm²	Custom-made	Increase (RT-qPCR, GAPDH)	24h post-FSS: 1.4 (rel.)†			verified; Primer-BLAST (primer) and BLAST (probe)
Yuan et al. (2012)	p-ERK1/2	MAPK3; MAPK1	hMSCs (Lonza) (n.g./ n.g. (n.g.), n.g., n.g., P4-8, n.g./ n.g.)	Steady laminar	10min, 30min, 60min, 120min/n.g.	0.2Pa	Custom-made			Increase (WB, β-actin)	0.2Pa @ 60min: 1.3/0.2 = 6.5 (ratio-calc)*	
Yuan et al. (2012)	p-ERK1/2	MAPK3; MAPK1	hMSCs (Lonza) (n.g./ n.g. (n.g.), n.g., n.g., P4-8, n.g./ n.g.)	Steady laminar	10min, 30min, 60min, 120min/n.g.	2Pa	Custom-made			Temporary decrease (WB, β-actin)	10min: 0.7/1.1 = 0.64 (ratio-calc)*<br>30min: 0.75/1.1 = 0.68 (ratio-calc)*	
Yuan et al. (2012)	p-JNK	MAPK8	hMSCs (Lonza) (n.g./ n.g. (n.g.), n.g., n.g., P4-8, n.g./ n.g.)	Steady laminar	10min, 30min, 60min, 120min/n.g.	0.2Pa	Custom-made			Increase (WB, β-actin)	30min: 1.7/0.4 = 4.25 (ratio-calc)*	
Yuan et al. (2012)	p-JNK	MAPK8	hMSCs (Lonza) (n.g./ n.g. (n.g.), n.g., n.g., P4-8, n.g./ n.g.)	Steady laminar	10min, 30min, 60min, 120min/n.g.	2Pa	Custom-made			Increase (WB, β-actin)	120min: 1.3/0.9 = 1.44 (ratio-calc)*	
Yuan et al. (2012)	p-p38 MAPK	MAPK14; MAPK11; MAPK12	hMSCs (Lonza) (n.g./ n.g. (n.g.), n.g., n.g., P4-8, n.g./ n.g.)	Steady laminar	10min, 30min, 60min, 120min/n.g.	0.2Pa	Custom-made			Increase with plateau (WB, β-actin)	30min: 0.6/0.2 = 3 (ratio-calc)*<br>120min: 0.5/0.2 = 2.5 (ratio-calc)*	
Yuan et al. (2012)	p-p38 MAPK	MAPK14; MAPK11; MAPK12	hMSCs (Lonza) (n.g./ n.g. (n.g.), n.g., n.g., P4-8, n.g./ n.g.)	Steady laminar	10min, 30min, 60min, 120min/n.g.	2Pa	Custom-made			Increase (WB, β-actin)	p-p38 MAPK: 120min: 0.9/0.7 = 1.29 (ratio-calc)*	

[a] Entry provided as reported in the given study.
[b] Human genes were confirmed with the HUGO Gene Nomenclature Committee (HGNC; URL: https://www.genenames.org); mouse genes were confirmed with the Mouse Genome Informatics (MGI; URL: https://www.informatics.jax.org/genes.shtml) after checking the specificity of primers with Primer-BLAST.
[c] Sex of donors: "M" – male, "F" – female; Tooth type: "PM" – premolar, "M" – molar; dig. Indicate isolation by cell digestion; Exp. indicate isolation by cell explant; Cell density: given in cells/cm² if not otherwise mentioned.
[d] Flow type deduced from the description of the FSS apparatus given by the authors.
[f] RT-qPCR (reverse-transcriptase quantitative polymerase chain reaction); sqPCR (semi-quantitative polymerase chain reaction); ELISA (enzyme-linked immuno absorbent assay); WB (western blotting); RIA (radioimmunoassay); EMSA (electromobility shift assay); IF (immunofluorescence)

rel.: indicate relative gene expression. Is entitled to percentages or gene expression ratios normalized to control, and not calculated by ΔΔCT.
FC: indicate fold change. When Author mentions the use of ΔΔCT or the method according to Livak & Schmittken (2004) in calculating FC.
n.g.: not given. For information not given by study-authors.
† Information derived from figures using Engauge Digitizer.
* Indicate manual calculations by measuring the graphs, without using the Engauge Digitizer.
ratio-calc: indicate manual calculation by dividing intervention/control = result (ratio-calc)
ratio: indicate ratios given by study-authors such as normalization to control in case of small molecules data or in case of gene expression ratios, e.g. ratio of RANKL/OPG or Bcl-2/Bax.

Reference	Gene or analyte[a]	Official gene symbol or abbreviation [b]	Cell type (age/number and sex of donor (health status), tooth type, isolation method, passages used, cell density/confluency)[a,c]	Flow type (steady laminar, pulsatile laminar, or oscillatory laminar)[a,d]	FSS duration and frequency (Hz)[a]	FSS magnitude[a]	FSS apparatus[a]	Gene expression: increase, decrease, no change (method w/ reference gene); methods: (RT-qPCR, sqPCR)[f]	Gene expression: when it reaches peak and peak's magnitude (fold change; relative gene expression; times or ratio; unclear = ?)[f]	Protein/metabolite expression: increase, decrease, no change (method w/reference); methods: ELISA, WB, RIA, EMSA, IF[f]	Protein/metabolite expression: when it reaches peak and peak's magnitude (times or ratio; unclear = ?)[f]	Remarks
Yuan et al. (2012)	p38 MAPK	MAPK14; MAPK11; MAPK12	hMSCs (Lonza) (n.g./ n.g. (n.g.), n.g., n.g., P4-8, n.g./ n.g.)	Steady laminar	10min, 30min, 60min, 120min/n.g.	0.2Pa	Custom-made			Increase with plateau (WB, β-actin)	10min: 0.8/0.65 = 1.2 (ratio-calc)*<br>120min: 0.76/0.65 = 1.16 (ratio-calc)*	
Yuan et al. (2012)	p38 MAPK	MAPK14; MAPK11; MAPK12	hMSCs (Lonza) (n.g./ n.g. (n.g.), n.g., n.g., P4-8, n.g./n.g.)	Steady laminar	10min, 30min, 60min, 120min/n.g.	2Pa	Custom-made			No change (WB, β-actin)	No change	
Yuan et al. (2012)	total-ERK1/2	MAPK3; MAPK1	hMSCs (Lonza) (n.g./ n.g. (n.g.), n.g., n.g., P4-8, n.g./n.g.)	Steady laminar	10min, 30min, 60min, 120min/n.g.	0.2Pa	Custom-made			Temporary decrease (WB, β-actin)	0.2Pa @ 30min: 1.1/1.35 = 0.81 (ratio-calc)*	
Yuan et al. (2012)	total-ERK1/2	MAPK3; MAPK1	hMSCs (Lonza) (n.g./ n.g. (n.g.), n.g., n.g., P4-8, n.g./n.g.)	Steady laminar	10min, 30min, 60min, 120min/n.g.	2Pa	Custom-made			No change (WB, β-actin)	No change	
Yuan et al. (2012)	total-JNK	MAPK8	hMSCs (Lonza) (n.g./ n.g. (n.g.), n.g., n.g., P4-8, n.g./ n.g.)	Steady laminar	10min, 30min, 60min, 120min/n.g.	0.2Pa	Custom-made			Temporary decrease then increase (WB, β-actin)	10min: 0.6/0.7 = 0.85 (ratio-calc)*<br>30min: 0.65/0.7 = 0.9 (ratio-calc)*<br>120min: 0.85/0.7 = 1.2 (ratio-calc)*	
Yuan et al. (2012)	total-JNK	MAPK8	hMSCs (Lonza) (n.g./ n.g. (n.g.), n.g., n.g., P4-8, n.g./ n.g.)	Steady laminar	10min, 30min, 60min, 120min/n.g.	2Pa	Custom-made			Temporary decrease (WB, β-actin)	30min: 1.1/1.5 = 0.7 (ratio-calc)*	
Kim et al. (2011)	CD31	PECAM1	MSCs (PT-2501; Lonza Walkersville, Inc., Walkersville, MD, USA) (n.g./ n.g. (n.g.), n.g., n.g., P5, 2.5×10^6 cells per cm^2/ n.g.)	Steady laminar	1d (24h)/n.g.	2.5dyn/cm^2, 10dyn/cm^2	Custom-made	2.5dyn/cm^2: increase (sqPCR; GAPDH)<br>10dyn/cm^2: increase (sqPCR; GAPDH)	2.5dyn/cm^2: 0.5/0.01 = 50 (ratio-calc)†<br>10dyn/cm^2: 0.1/0.01 = 10 (ratio-calc)†			
Kim et al. (2011)	Flk-1/KDR	KDR	MSCs (PT-2501; Lonza Walkersville, Inc., Walkersville, MD, USA) (n.g./ n.g. (n.g.), n.g., n.g., P5, 2.5×10^6 cells per cm^2/ n.g.)	Steady laminar	1d (24h)/n.g.	2.5dyn/cm^2, 10dyn/cm^2	Custom-made	2.5dyn/cm^2: increase (sqPCR; GAPDH)<br>10dyn/cm^2: increase (sqPCR; GAPDH)	2.5dyn/cm^2: 0.06/0.001 = 60 (ratio-calc)†<br>10dyn/cm^2: 0.05/0.001 = 50 (ratio-calc)†			
Kim et al. (2011)	MHC (myosine heavy chain C)	MYH11	MSCs (PT-2501; Lonza Walkersville, Inc., Walkersville, MD, USA) (n.g./ n.g. (n.g.), n.g., n.g., P5, 2.5×10^6 cells per cm^2/ n.g.)	Steady laminar	1d (24h)/n.g.	2.5dyn/cm^2, 10dyn/cm^2	Custom-made	2.5dyn/cm^2: increase (sqPCR; GAPDH)<br>10dyn/cm^2: increase (sqPCR; GAPDH)	2.5dyn/cm^2: 0.3/ 0.2 = 1.5 (ratio-calc)†<br>10dyn/cm^2: 0.5/0.2 = 2.5 (ratio-calc)†			Specificy verified by Primer-BLAST
Kim et al. (2011)	Myocardin	MRTFA	MSCs (PT-2501; Lonza Walkersville, Inc., Walkersville, MD, USA) (n.g./ n.g. (n.g.), n.g., n.g., P5, 2.5×10^6 cells per cm^2/ n.g.)	Steady laminar	1d (24h)/n.g.	2.5dyn/cm^2, 10dyn/cm^2	Custom-made	2.5dyn/cm^2: increase (sqPCR; GAPDH)<br>10dyn/cm^2: increase (sqPCR; GAPDH)	2.5dyn/cm^2: 0.3/0.1 = 3 (ratio-calc)†<br>10dyn/cm^2: 0.8/0.1 = 8 (ratio-calc)†			
Kim et al. (2011)	SM-22alpha	TAGLN	MSCs (PT-2501; Lonza Walkersville, Inc., Walkersville, MD, USA) (n.g./ n.g. (n.g.), n.g., n.g., P5, 2.5×10^6 cells per cm^2/ n.g.)	Steady laminar	1d (24h)/n.g.	2.5dyn/cm^2, 10dyn/cm^2	Custom-made	2.5dyn/cm^2: increase (sqPCR; GAPDH)<br>10dyn/cm^2: increase (sqPCR; GAPDH)	2.5dyn/cm^2: 0.4/0.3 = 1.3 (ratio-calc)†<br>10dyn/cm^2: 0.8/0.3 = 2.7 (ratio-calc)†			
Kim et al. (2011)	vWF	VWF	MSCs (PT-2501; Lonza Walkersville, Inc., Walkersville, MD, USA) (n.g./ n.g. (n.g.), n.g., n.g., P5, 2.5×10^6 cells per cm^2/ n.g.)	Steady laminar	1d (24h)/n.g.	2.5dyn/cm^2, 10dyn/cm^2	Custom-made	2.5dyn/cm^2: increase (sqPCR; GAPDH)<br>10dyn/cm^2: increase (sqPCR; GAPDH)	2.5dyn/cm^2: 0.04/0.007 = 5.7 (ratio-calc)†<br>10dyn/cm^2: 0.03/0.008 = 3.8 (ratio-calc)†			
Kraft et al. (2010)	COX1	PTGS1	human dental pulp-derived mesenchymal stem cells (PDSCs) (20y / 1M (healthy), M, dig., P2-11 and P4-23, 4×10^5 cells/ n.g.) "mature"	Pulsating laminar	1h FSS (0min, 5min, 10min, 15min, 30min, 60min, 3h post-FSS incubation)/5Hz	0.6Pa, a pulse amplitude of 0.3Pa and a peak shear stress rate of 8.4Pa/s.	Custom-made	No change (RT-qPCR, GUS)	No change			"mature" phenotype

[a] Entry provided as reported in the given study.
[b] Human genes were confirmed with the HUGO Gene Nomenclature Committee (HGNC; URL: https://www.genenames.org); mouse genes were confirmed with the Mouse Genome Informatics (MGI; URL: https://www.informatics.jax.org/genes.shtml) after checking the specificity of primers with Primer-BLAST.
[c] Sex of donors: "M" – male, "F" – female; Tooth type: "PM" – premolar, "M" – molar; dig. Indicate isolation by cell digestion; Exp. indicate isolation by cell explant; Cell density: given in cells/cm^2 if not otherwise mentioned.
[d] Flow type deduced from the description of the FSS apparatus given by the authors.
[f] RT-qPCR (reverse-transcriptase quantitative polymerase chain reaction); sqPCR (semi-quantitative polymerase chain reaction); ELISA (enzyme-linked immuno absorbent assay); WB (western blotting); RIA (radioimmunoassay); EMSA (electromobility shift assay); IF (immunofluorescence)

rel.: indicate relative gene expression. Is entitled to percentages or gene expression ratios normalized to control, and not calculated by ΔΔCT.
FC: indicate fold change. When Author mentions the use of ΔΔCT or the method according to Livak & Schmittken (2004) in calculating FC.
n.g.: not given. For information not given by study-authors.
† Information derived from figures using Engauge Digitizer.
* Indicate manual calculations by measuring the graphs, without using the Engauge Digitizer.
ratio-calc: indicate manual calculation by dividing intervention/control = result (ratio-calc)
ratio: indicate ratios given by study-authors such as normalization to control in case of small molecules data or in case of gene expression ratios, e.g. ratio of RANKL/OPG or Bcl-2/Bax.

Reference	Gene or analyte[a]	Official gene symbol or abbreviation [b]	Cell type (age/number and sex of donor (health status), tooth type, isolation method, passages used, cell density/confluency)[a,c]	Flow type (steady laminar, pulsatile laminar, or oscillatory laminar)[a,d]	FSS duration and frequency (Hz)[a]	FSS magnitude[a]	FSS apparatus[a]	Gene expression: increase, decrease, no change (method w/ reference gene); methods: (RT-qPCR, sqPCR)[f]	Gene expression: when it reaches peak and peak's magnitude (fold change; relative gene expression; times or ratio; unclear = ?)[f]	Protein/metabolite expression: increase, decrease, no change (method w/reference); methods: ELISA, WB, RIA, EMSA, IF[f]	Protein/metabolite expression: when it reaches peak and peak's magnitude (times or ratio; unclear = ?)[f]	Remarks
Kraft et al. (2010)	COX1	PTGS1	human dental pulp-derived mesenchymal stem cells (PDSCs) (21y / 1F (healthy), M, dig., P2-11 and P4-23, 4×10^5 cells/ n.g.) "immature"	Pulsating laminar	1h FSS (0min, 5min, 10min, 15min, 30min, 60min, 3h post-FSS incubation)/5Hz	0.6Pa, a pulse amplitude of 0.3Pa and a peak shear stress rate of 8.4Pa/s.	Custom-made	Increase then decrease (RT-qPCR, GUS)	0h post-FSS: 1.6 (FC)†<br>3h post-FSS: 0.97 (FC)†			"immature" phenotype
Kraft et al. (2010)	COX2	PTGS2	human dental pulp-derived mesenchymal stem cells (PDSCs) (20y / 1M (healthy), M, dig., P2-11 and P4-23, 4×10^5 cells/ n.g.) "mature"	Pulsating laminar	1h FSS (0min, 5min, 10min, 15min, 30min, 60min, 3h post-FSS incubation)/5Hz	0.6Pa, a pulse amplitude of 0.3Pa and a peak shear stress rate of 8.4Pa/s.	Custom-made	Increase (RT-qPCR, GUS)	3h post-FSS: 2.96 (FC)†			"mature" phenotype
Kraft et al. (2010)	COX2	PTGS2	human dental pulp-derived mesenchymal stem cells (PDSCs) (21y / 1F (healthy), M, dig., P2-11 and P4-23, 4×10^5 cells/ n.g.) "immature"	Pulsating laminar	1h FSS (0min, 5min, 10min, 15min, 30min, 60min, 3h post-FSS incubation)/5Hz	0.6Pa, a pulse amplitude of 0.3Pa and a peak shear stress rate of 8.4Pa/s.	Custom-made	Increase (RT-qPCR, GUS)	1h post-FSS: 4.9 (FC)†			"immature" phenotype
Kraft et al. (2010)	NO	Nitric oxide	human dental pulp-derived mesenchymal stem cells (PDSCs) (20y / 1M (healthy), M, dig., P2-11 and P4-23, 4×10^5 cells/n.g.) "mature"	Pulsating laminar	1h FSS (0min, 5min, 10min, 15min, 30min, 60min, 3h post-FSS incubation)/5Hz	0.6Pa, a pulse amplitude of 0.3Pa and a peak shear stress rate of 8.4Pa/s.	Custom-made			0h post-FSS: increase (Griess, NO_2^-)<br>Post-FSS: decrease then increase (Griess, NO_2^-)	0h post-FSS: 17.1nmol; 17.1/4.5 = 3.8 (ratio-calc)†<br>1h post-FSS: 1.8nmol; 1.8/2.3 = 0.78 (ratio-calc)†<br>3h post-FSS: 5.4nmol; 5.4/3.5 = 1.54 (ratio-calc)†	"mature" phenotype;
Kraft et al. (2010)	NO	Nitric oxide	human dental pulp-derived mesenchymal stem cells (PDSCs) (21y / 1F (healthy), M, dig., P2-11 and P4-23, 4×10^5 cells/n.g.) "immature"	Pulsating laminar	1h FSS (0min, 5min, 10min, 15min, 30min, 60min, 3h post-FSS incubation)/5Hz	0.6Pa, a pulse amplitude of 0.3Pa and a peak shear stress rate of 8.4Pa/s.	Custom-made			0h post-FSS: increase (Griess, NO_2^-)<br>Post-FSS: decrease then increase (Griess, NO_2^-)	0h post-FSS: 12.2nmol; 12.2/2.3 = 5.31 (ratio-calc)†<br>1h post-FSS: 1.8nmol; 1.8/2.4 = 0.75 (ratio-calc)†<br>3h post-FSS: 2.9nmol; 2.9/2.5 = 1.16 (ratio-calc)†	"immature" phenotype
Kraft et al. (2010)	PGE2	PGE2	human dental pulp-derived mesenchymal stem cells (PDSCs) (20y / 1M (healthy), M, dig., P2-11 and P4-23, 4×10^5 cells/n.g.) "mature"	Pulsating laminar	1h FSS (0min, 5min, 10min, 15min, 30min, 60min, 3h post-FSS incubation)/5Hz	0.6Pa, a pulse amplitude of 0.3Pa and a peak shear stress rate of 8.4Pa/s	Custom-made			0h post-FSS: increase (ELISA)<br>Post-FSS: increase (ELISA)	0h post-FSS: 1.7 ng; 1.7/0.8 = 2.13 (ratio-calc)†<br>1h post-FSS: 15.2 ng; 15.2/2.7 = 5.63 (ratio-calc)†	"mature" phenotype
Kraft et al. (2010)	PGE2	PGE2	human dental pulp-derived mesenchymal stem cells (PDSCs) (21y / 1F (healthy), M, dig., P2-11 and P4-23, 4×10^5 cells/n.g.) "immature"	Pulsating laminar	1h FSS (0min, 5min, 10min, 15min, 30min, 60min, 3h post-FSS incubation)/5Hz	0.6Pa, a pulse amplitude of 0.3Pa and a peak shear stress rate of 8.4Pa/s.	Custom-made			0h post FSS: increase (ELISA)<br>1h post-FSS: increase (ELISA)	0h post-FSS: 2.3 ng; 2.3/1.1 = 2.09 (ratio-calc)†<br>1h post-FSS: 5.6 ng; 5.6/0.9 = 6.22 (ratio-calc)†	"immature" phenotype
Charoenpong et al. (2019)	IL1β	IL1B	Human Pulpal Stem Cells (HDPCs) (n.g./ n.g. (n.g.), M, Ficoll paque, P3-6, n.g./ n.g.)	Steady laminar	2h, 6h, 16h/n.g.	0.1Pa, 0.2Pa	Custom-made	Decrease then increase (RT-qPCR, GAPDH)	0.2Pa @ 2h: 0.9 (rel.)†<br>0.2Pa @ 6h: 1.4 (rel.)†			Angular fluid flow; rotating cone
Charoenpong et al. (2019)	IL6	IL6	Human Pulpal Stem Cells (HDPCs) (n.g./ n.g. (n.g.), M, Ficoll paque, P3-6, n.g./ n.g.)	Steady laminar	2h, 6h, 16h/n.g.	0.1Pa, 0.2Pa	Custom-made	Increase (RT-qPCR, GAPDH)	0.2Pa @ 6h: 1.9555 (rel.)†			
Charoenpong et al. (2019)	S100A4	S100A4	Human Pulpal Stem Cells (HDPCs) (n.g./ n.g. (n.g.), M, Ficoll paque, P3-6, n.g./ n.g.)	Steady laminar	2h, 6h, 16h/n.g.	0.1Pa, 0.2Pa	Custom-made	0.1Pa: Increase, decrease then baseline (RT-qPCR, GAPDH)<br>0.2Pa: decrease then increase (RT-qPCR, GAPDH)	0.1Pa @ 2h: 1.1 (rel.)†<br>0.1Pa @ 6h: 0.7 (rel.)†<br>0.1Pa @ 16h: 1 (rel.)†<br>0.2Pa @ 6h: 0.8 (rel.)†<br>0.2Pa @ 16h: 1 (rel.)†			
Charoenpong et al. (2019)	S100A7	S100A7	Human Pulpal Stem Cells (HDPCs) (n.g./ n.g. (n.g.), M, Ficoll paque, P3-6, n.g./ n.g.)	Steady laminar	2h, 6h, 16h/n.g.	0.1Pa, 0.2Pa	Custom-made	0.1Pa: increase (RT-qPCR, GAPDH)<br>0.2Pa: increase (RT-qPCR, GAPDH)	0.1Pa @ 16h: 2.5 (rel.)†<br>0.2Pa @ 16h: 5.3 (rel.)†	0.1Pa: increase (ELISA)<br>0.2Pa: increase (ELISA)	0.1Pa @ 16h: 112.7pg/mL; 112.7/32.7 = 3.45 (ratio-calc)†<br>0.2Pa @ 16h: 469pg/mL; 469/32.7 = 14.34 (ratio-calc)†	

[a] Entry provided as reported in the given study.
[b] Human genes were confirmed with the HUGO Gene Nomenclature Committee (HGNC; URL: https://www.genenames.org); mouse genes were confirmed with the Mouse Genome Informatics (MGI; URL: https://www.informatics.jax.org/genes.shtml) after checking the specificity of primers with Primer-BLAST.
[c] Sex of donors: "M" – male, "F" – female; Tooth type: "PM" – premolar, "M" – molar; dig. Indicate isolation by cell digestion; Exp. indicate isolation by cell explant; Cell density: given in cells/cm² if not otherwise mentioned.
[d] Flow type deduced from the description of the FSS apparatus given by the authors.
[f] RT-qPCR (reverse-transcriptase quantitative polymerase chain reaction); sqPCR (semi-quantitative polymerase chain reaction); ELISA (enzyme-linked immuno absorbent assay); WB (western blotting); RIA (radioimmunoassay); EMSA (electromobility shift assay); IF (immunofluorescence)

rel.: indicate relative gene expression. Is entitled to percentages or gene expression ratios normalized to control, and not calculated by ΔΔCT.
FC: indicate fold change. When Author mentions the use of ΔΔCT or the method according to Livak & Schmittken (2004) in calculating FC.
n.g.: not given. For information not given by study-authors.
† Information derived from figures using Engauge Digitizer.
* Indicate manual calculations by measuring the graphs, without using the Engauge Digitizer.
ratio-calc: indicate manual calculation by dividing intervention/control = result (ratio-calc)
ratio: indicate ratios given by study-authors such as normalization to control in case of small molecules data or in case of gene expression ratios, e.g. ratio of RANKL/OPG or Bcl-2/Bax.

Reference	Gene or analyte[a]	Official gene symbol or abbreviation[b]	Cell type (age/number and sex of donor (health status), tooth type, isolation method, passages used, cell density/confluency)[a,c]	Flow type (steady laminar, pulsatile laminar, or oscillatory laminar)[a,d]	FSS duration and frequency (Hz)[a]	FSS magnitude[a]	FSS apparatus[a]	Gene expression: increase, decrease, no change (method w/ reference gene); methods: (RT-qPCR, sqPCR)[f]	Gene expression: when it reaches peak and peak's magnitude (fold change; relative gene expression; times or ratio; unclear = ?)[f]	Protein/metabolite expression: increase, decrease, no change (method w/reference); methods: ELISA, WB, RIA, EMSA, IF[f]	Protein/metabolite expression: when it reaches peak and peak's magnitude (times or ratio; unclear = ?)[f]	Remarks
Charoenpong et al. (2019)	S100A8	S100A8	Human Pulpal Stem Cells (HDPCs) (n.g./ n.g. (n.g.), M, Ficoll paque, P3-6, n.g./ n.g.)	Steady laminar	2h, 6h, 16h/n.g.	0.1Pa, 0.2Pa	Custom-made	0.1Pa @ 16h: increase (RT-qPCR, GAPDH)<br>0.2 Pa @ 16h: increase (RT-qPCR, GAPDH)	0.1Pa @ 2h: 1 (rel.)†<br>0.1Pa @ 16h: 7.5 (rel.)†<br>0.2Pa @ 2h: 1 (rel.)†<br>0.2Pa @ 16h: 7.1 (rel.)†			
Charoenpong et al. (2019)	VEGF	VEGFA	Human Pulpal Stem Cells (HDPCs) (n.g./ n.g. (n.g.), M, Ficoll paque, P3-6, n.g./ n.g.)	Steady laminar	2h, 6h, 16h/n.g.	0.1Pa, 0.2Pa	Custom-made	Increase (RT-qPCR, GAPDH)	0.2 Pa @ 2h: 1.5 (rel.)†			
Glossop and Cartmell (2009)	DUSP6	DUSP6	Primary human MSCs, bone marrow (Lonza) (23y/1F (healthy), n.g., n.g., P5, 2x10^5/ n.g.)	Pulsatile laminar (setup not described, but peristaltic pump was used)	1h FSS (sampling 0h, 1h, 2h, 24h post-FSS)/n.g.	1dyn/cm^2, 5dyn/cm^2, 10dyn/cm^2	Streamer (Flexcell Int.)	1dyn/cm^2: increase (RT-qPCR, 18S)<br>5dyn/cm^2: increase then decrease (RT-qPCR, 18S)<br>10dyn/cm^2: temporary increase then base level, temporary increase then base level (RT-qPCR, 18S)	1dyn/cm^2 @ 2h post-FSS: 25.1 (FC)†<br>5dyn/cm^2 @ 0h post-FSS: 7.9 (FC)†<br>5dyn/cm^2 @ 24h post-FSS: -2 (FC)†<br>10dyn/cm^2 @ 0h post-FSS: 6.5 (FC)†<br>10dyn/cm^2 @ 1h post-FSS: 1 (FC)†<br>10dyn/cm^2 @ 2h post-FSS: 2.5 (FC)†<br>10dyn/cm^2 @ 24h post-FSS: 1 (FC)†			
Glossop and Cartmell (2009)	GADD45B	GADD45B	Primary human MSCs, bone marrow (Lonza) (23y/1F (healthy), n.g., n.g., P5, 2×10^5/ n.g.)	Pulsatile laminar (setup not described, but peristaltic pump was used)	1h FSS (sampling 0h, 1h, 2h, 24h post-FSS FSS)/n.g.	1dyn/cm^2, 5dyn/cm^2, 10dyn/cm^2	Streamer (Flexcell Int.)	1dyn/cm^2: increase (RT-qPCR, 18S)<br>5dyn/cm^2: increase then decrease (RT-qPCR, 18S)<br>10dyn/cm^2: increase, temporary decrease then increase (RT-qPCR, 18S)	1dyn/cm^2 @ 2h post-FSS: 8 (FC)†<br>5dyn/cm^2 @ 0h post-FSS: 16.9 (FC)†<br>5dyn/cm^2 @ 24h post-FSS: -1.1 (FC)†<br>10dyn/cm^2 @ 1h post-FSS: 5.1 (FC)†<br>10dyn/cm^2 @ 2h post-FSS: -1 (FC)†<br>10dyn/cm^2 @ 24h post-FSS: 1.9 (FC)†			
Glossop and Cartmell (2009)	IL1B	IL1B	Primary human MSCs, bone marrow (Lonza) (23y/1F (healthy), n.g., n.g., P5, 2×10^5/ n.g.)	Pulsatile laminar (setup not described, but peristaltic pump was used)	1h FSS (sampling 0h, 1h, 2h, 24h post-FSS FSS)/n.g.	1dyn/cm^2, 5dyn/cm^2, 10dyn/cm^2	Streamer (Flexcell Int.)	1dyn/cm^2: increase (RT-qPCR, 18S)<br>5dyn/cm^2: increase (RT-qPCR, 18S)<br>10dyn/cm^2: increase (RT-qPCR, 18S)	1dyn/cm^2 @ 2h post-FSS: 153.6 (FC)†<br>5dyn/cm^2 @ 2h post-FSS: 24.6 (FC)†<br>10dyn/cm^2 @ 2h post-FSS: 46.4 (FC)†			
Glossop and Cartmell (2009)	JUN	JUN	Primary human MSCs, bone marrow (Lonza) (23y/1F (healthy), n.g., n.g., P5, 2×10^5/ n.g.)	Pulsatile laminar (setup not described, but peristaltic pump was used)	10min FSS (sampling 0h, 1h, 2h, 24h post-FSS)/n.g.	1dyn/cm^2, 5dyn/cm^2, 10dyn/cm^2	Streamer (Flexcell Int.)	1dyn/cm^2: increase (RT-qPCR, 18S)	24h post-FSS: 9.1 (FC)†			
Glossop and Cartmell (2009)	MAP3K8	MAP3K8	Primary human MSCs, bone marrow (Lonza) (23y/1F (healthy), n.g., n.g., P5, 2×10^5/ n.g.)	Pulsatile laminar (setup not described, but peristaltic pump was used)	1h FSS (sampling 0h, 1h, 2h, 24h post-FSS)/n.g.	1dyn/cm^2, 5dyn/cm^2, 10dyn/cm^2	Streamer (Flexcell Int.)	1dyn/cm^2: increase (RT-qPCR, 18S)<br>5dyn/cm^2: increase then decrease (RT-qPCR, 18S)<br>10dyn/cm^2: increase (RT-qPCR, 18S)	1dyn/cm^2 @ 2h post-FSS: 38.3 (FC)†<br>5dyn/cm^2 @ 2h post-FSS: 12.4 (FC)†<br>5dyn/cm^2 @ 24h post-FSS: -2.5 (FC)†<br>10dyn/cm^2 @ 2h post-FSS: 7.7 (FC)†			

[a] Entry provided as reported in the given study.
[b] Human genes were confirmed with the HUGO Gene Nomenclature Committee (HGNC; URL: https://www.genenames.org); mouse genes were confirmed with the Mouse Genome Informatics (MGI; URL: https://www.informatics.jax.org/genes.shtml) after checking the specificity of primers with Primer-BLAST.
[c] Sex of donors: "M" – male, "F" – female; Tooth type: "PM" – premolar, "M" – molar; dig. Indicate isolation by cell digestion; Exp. indicate isolation by cell explant; Cell density: given in cells/cm^2 if not otherwise mentioned.
[d] Flow type deduced from the description of the FSS apparatus given by the authors.
[f] RT-qPCR (reverse-transcriptase quantitative polymerase chain reaction); sqPCR (semi-quantitative polymerase chain reaction); ELISA (enzyme-linked immuno absorbent assay); WB (western blotting); RIA (radioimmunoassay); EMSA (electromobility shift assay); IF (immunofluorescence)

rel.: indicate relative gene expression. Is entitled to percentages or gene expression ratios normalized to control, and not calculated by ΔΔCT.
FC: indicate fold change. When Author mentions the use of ΔΔCT or the method according to Livak & Schmittken (2004) in calculating FC.
n.g.: not given. For information not given by study-authors.
† Information derived from figures using Engauge Digitizer.
* Indicate manual calculations by measuring the graphs, without using the Engauge Digitizer.
ratio-calc: indicate manual calculation by dividing intervention/control = result (ratio-calc)
ratio: indicate ratios given by study-authors such as normalization to control in case of small molecules data or in case of gene expression ratios, e.g. ratio of RANKL/OPG or Bcl-2/Bax.

Reference	Gene or analyte[a]	Official gene symbol or abbreviation[b]	Cell type (age/number and sex of donor (health status), tooth type, isolation method, passages used, cell density/confluency)[a,c]	Flow type (steady laminar, pulsatile laminar, or oscillatory laminar)[a,d]	FSS duration and frequency (Hz)[a]	FSS magnitude[a]	FSS apparatus[a]	Gene expression: increase, decrease, no change (method w/ reference gene); methods: (RT-qPCR, sqPCR)[f]	Gene expression: when it reaches peak and peak's magnitude (fold change; relative gene expression; times or ratio; unclear = ?)[f]	Protein/metabolite expression: increase, decrease, no change (method w/reference); methods: ELISA, WB, RIA, EMSA, IF[f]	Protein/metabolite expression: when it reaches peak and peak's magnitude (times or ratio; unclear = ?)[f]	Remarks
Glossop and Cartmell (2009)	NFKB1	NFKB1	Primary human MSCs, bone marrow (Lonza) (23y/1F (healthy), n.g., n.g., P5, 2x10^5/ n.g.)	Pulsatile laminar (setup not described, but peristaltic pump was used)	1h FSS (sampling 0h, 1h, 2h, 24h post-FSS)/n.g.	1dyn/cm^2, 5dyn/cm^2, 10dyn/cm^2	Streamer (Flexcell Int.)	1dyn/cm^2: increase (RT-qPCR, 18S)	24h post-FSS: 6.1 (FC)†			
Glossop and Cartmell (2009)	PDGFA	PDGFA	Primary human MSCs, bone marrow (Lonza) (23y/1F (healthy), n.g., n.g., P5, 2×10^5/ n.g.)	Pulsatile laminar (setup not described, but peristaltic pump was used)	1h FSS (sampling 0h, 1h, 2h, 24h post-FSS)/n.g.	1dyn/cm^2, 5dyn/cm^2, 10dyn/cm^2	Streamer (Flexcell Int.)	1dyn/cm^2: increase (RT-qPCR, 18S)	24h post-FSS: 18.7 (FC)†			might be correct; RT-qPCR with TaqMan probes w/o reference.
Lim et al. (2013)	ALP	ALPL	hABMSCs (n.g./ n.g. (n.g.), n.g., n.g., P3-5, n.g./ n.g.)	Oscillatory laminar	5, 10, 30, 60, and 120min/d for 2w or 3w / n.g.	0.01-0.0205dyn/cm^2 (max. 0.00205Pa)	Rocking culture system (Vision Ltd.)	2w: increase with plateau (5-30min/d) then maximum increase at 60min (sqPCR, GAPDH)<br>3w: increase (sqPCR, GAPDH)	5min/d for 2w: 2.5 (rel.)†<br>10min/d for 2w: 2.5 (rel.)†<br>30min/d for 2w: 2.4 (rel.)†<br>60min/d for 2w: 5.9 (rel.)†<br>60min/d for 3w: 2.6 (rel.)†	2w: increase (colorimetric assay)	30min/d for 2w: 7.2ng/h/protein; 7.2/5.3 = 1.4 (ratio-calc)†	Genbank: BC090861
Lim et al. (2013)	ANTIBODY ARRAY		hABMSCs (n.g./ n.g. (n.g.), n.g., n.g., P3-5, n.g./ n.g.)	Oscillatory laminar	5, 10, 30, 60, and 120min/d for 2w or 3w / n.g.	0.01-0.0205dyn/cm^2 (max. 0.00205Pa)	Rocking culture system (Vision Ltd.)			RayBio™ Human Growth Factor Antibody Array (RayBiotech, Inc.)		
Lim et al. (2013)	COL-I	COL1A1	hABMSCs (n.g./ n.g. (n.g.), n.g., n.g., P3-5, n.g./ n.g.)	Oscillatory laminar	5, 10, 30, 60, and 120min/d for 2w or 3w / n.g.	0.01-0.0205dyn/cm^2 (max. 0.00205Pa)	Rocking culture system (Vision Ltd.)	2w: increase followed by plateau (sqPCR, GAPDH)<br>3w: increase followed by plateau (sqPCR, GAPDH)	30min/d for 2w: 1.55 (rel.) †<br>120min/d for 2w: 1.6 (rel.)†<br>30min/d for 3w: 1.21 (rel.) †<br>120min/d for 3w: 1.3 (rel.)†			
Lim et al. (2013)	EGF	EGF	hABMSCs (n.g./ n.g. (n.g.), n.g., n.g., P3-5, n.g./ n.g.)	Oscillatory laminar	5, 10, 30, 60, and 120min/d / n.g.	0.01-0.0205dyn/cm^2 (max. 0.00205Pa)	Rocking culture system (Vision Ltd.)			Increase (antibody array)	Time not mentioned, only fold change	p.142/a
Lim et al. (2013)	HGF	HGF	hABMSCs (n.g./ n.g. (n.g.), n.g., n.g., P3-5, n.g./ n.g.)	Oscillatory laminar	5, 10, 30, 60, and 120min/d / n.g.	0.01-0.0205dyn/cm^2 (max. 0.00205Pa)	Rocking culture system (Vision Ltd.)			Increase (antibody array)	Time not mentioned	p.142/b
Lim et al. (2013)	IBSP	IBSP	hABMSCs (n.g./ n.g. (n.g.), n.g., n.g., P3-5, n.g./ n.g.)	Oscillatory laminar	5, 10, 30, 60, and 120min/d for 2w or 3w / n.g.	0.01-0.0205dyn/cm^2 (max. 0.00205Pa)	Rocking culture system (Vision Ltd.)	2w: temporary increase then base level, temporary increase then base level (sqPCR, GAPDH)<br>3w: temporary decrease followed by temporary increase then decrease with plateau (sqPCR, GAPDH)	5min/d for 2w: 2.1 (rel.)†<br>10min/d for 2w: base level<br>60min/d for 2w: 4.4 (rel.)†<br>120min/d for 2w: base level<br>5min/d for 3w: 0.7 (rel.)†<br>10min/d for 3w: 1.1 (rel.)†<br>30min/d for 3w: 0.81 (rel.)†<br>60min/d for 3w: 0.8 (rel.)†<br>120min/d for 3w: 0.82 (rel.)†			
Lim et al. (2013)	IGF-1	IGF1	hABMSCs (n.g./ n.g. (n.g.), n.g., n.g., P3-5, n.g./ n.g.)	Oscillatory laminar	5, 10, 30, 60, and 120min/d / n.g.	0.01-0.0205dyn/cm^2 (max. 0.00205Pa)	Rocking culture system (Vision Ltd.)			Increase (antibody array)	Time not mentioned	p.142/d
Lim et al. (2013)	IGF-II	IGF2	hABMSCs (n.g./ n.g. (n.g.), n.g., n.g., P3-5, n.g./ n.g.)	Oscillatory laminar	5, 10, 30, 60, and 120min/d / n.g.	0.01-0.0205dyn/cm^2 (max. 0.00205Pa)	Rocking culture system (Vision Ltd.)			Increase (antibody array)	Time not mentioned	p.142/e
Lim et al. (2013)	OCN	BGLAP	hABMSCs (n.g./ n.g. (n.g.), n.g., n.g., P3-5, n.g./ n.g.)	Oscillatory laminar	5, 10, 30, 60, and 120min/d for 2w or 3w /n.g.	0.01-0.0205dyn/cm^2 (max. 0.00205Pa)	Rocking culture system (Vision Ltd.)	2w: increase (sqPCR, GAPDH)<br>3w: increase (sqPCR, GAPDH)	10min/d for 2w: 4.9 (rel.)†<br>60min/d for 3w: 1.7 (rel.)†			
Lim et al. (2013)	OPN	SPP1	hABMSCs (n.g./ n.g. (n.g.), n.g., n.g., P3-5, n.g./ n.g.)	Oscillatory laminar	5, 10, 30, 60, and 120min/d for 2w or 3w/n.g.	0.01-0.0205dyn/cm^2 (max. 0.00205Pa)	Rocking culture system (Vision Ltd.)	2w: increase (sqPCR, GAPDH)<br>3w: increase followed by plateau (sqPCR, GAPDH)	30min/d for 2w: 6.0 (rel.)†<br>30min/d for 3w: 1.26 (rel.)†<br>600min/d for 3w: 1.22 (rel.)†<br>120min/d for 3w: 1.2 (rel.)†			
Lim et al. (2013)	PDGF-AA	PDGFA	hABMSCs (n.g./ n.g. (n.g.), n.g., n.g., P3-5, n.g./ n.g.)	Oscillatory laminar	5, 10, 30, 60, and 120min/d / n.g.	0.01-0.0205dyn/cm^2 (max. 0.00205Pa)	Rocking culture system (Vision Ltd.)			Increase (antibody array)	Time not mentioned	p.142/g

[a] Entry provided as reported in the given study.
[b] Human genes were confirmed with the HUGO Gene Nomenclature Committee (HGNC; URL: https://www.genenames.org); mouse genes were confirmed with the Mouse Genome Informatics (MGI; URL: https://www.informatics.jax.org/genes.shtml) after checking the specificity of primers with Primer-BLAST.
[c] Sex of donors: "M" – male, "F" – female; Tooth type: "PM" – premolar, "M" – molar; dig. Indicate isolation by cell digestion; Exp. indicate isolation by cell explant; Cell density: given in cells/cm^2 if not otherwise mentioned.
[d] Flow type deduced from the description of the FSS apparatus given by the authors.
f RT-qPCR (reverse-transcriptase quantitative polymerase chain reaction); sqPCR (semi-quantitative polymerase chain reaction); ELISA (enzyme-linked immuno absorbent assay); WB (western blotting); RIA (radioimmunoassay); EMSA (electromobility shift assay); IF (immunofluorescence)

rel.: indicate relative gene expression. Is entitled to percentages or gene expression ratios normalized to control, and not calculated by ΔΔCT.
FC: indicate fold change. When Author mentions the use of ΔΔCT or the method according to Livak & Schmittken (2004) in calculating FC.
n.g.: not given. For information not given by study-authors.
† Information derived from figures using Engauge Digitizer.
* Indicate manual calculations by measuring the graphs, without using the Engauge Digitizer.
ratio-calc: indicate manual calculation by dividing intervention/control = result (ratio-calc)
ratio: indicate ratios given by study-authors such as normalization to control in case of small molecules data or in case of gene expression ratios, e.g. ratio of RANKL/OPG or Bcl-2/Bax.

Reference	Gene or analyte[a]	Official gene symbol or abbreviation[b]	Cell type (age/number and sex of donor (health status), tooth type, isolation method, passages used, cell density/confluency)[a,c]	Flow type (steady laminar, pulsatile laminar, or oscillatory laminar)[a,d]	FSS duration and frequency (Hz)[a]	FSS magnitude[a]	FSS apparatus[a]	Gene expression: increase, decrease, no change (method w/ reference gene); methods: (RT-qPCR, sqPCR)[f]	Gene expression: when it reaches peak and peak's magnitude (fold change; relative gene expression; times or ratio; unclear = ?)[f]	Protein/metabolite expression: increase, decrease, no change (method w/reference); methods: ELISA, WB, RIA, EMSA, IF[f]	Protein/metabolite expression: when it reaches peak and peak's magnitude (times or ratio; unclear = ?)[f]	Remarks
Lim et al. (2013)	PDGF-BB	PDGFB	hABMSCs (n.g./ n.g. (n.g.), n.g., n.g., P3-5, n.g./ n.g.)	Oscillatory laminar	5, 10, 30, 60, and 120min/d / n.g.	0.01-0.0205dyn/cm² (max. 0.00205Pa)	Rocking culture system (Vision Ltd.)			Increase (antibody array)	Time not mentioned	p.142/h
Lim et al. (2013)	RUNX2	RUNX2	hABMSCs (n.g./ n.g. (n.g.), n.g., n.g., P3-5, n.g./ n.g.)	Oscillatory laminar	5, 10, 30, 60, and 120min/d for 2w or 3w / n.g.	0.01-0.0205dyn/cm² (max. 0.00205Pa)	Rocking culture system (Vision Ltd.)	2w: increase followed by plateau (sqPCR, GAPDH)<br>3w: increase then Decrease (sqPCR, GAPDH)	5min/d for 2w: 2.7 (rel.)†<br>10min/d for 2w: 3 (rel.)†<br>30min/d for 2w: 3.1 (rel.)†<br>5min/d for 3w: 1.3 (rel.)†<br>120min/d for 3w: 0.7 (rel.)†			
Lim et al. (2013)	TGF-b3	TGFB3	hABMSCs (n.g./ n.g. (n.g.), n.g., n.g., P3-5, n.g./ n.g.)	Oscillatory laminar	5, 10, 30, 60, and 120min/d / n.g.	0.01-0.0205dyn/cm² (max. 0.00205Pa)	Rocking culture system (Vision Ltd.)			Increase (antibody array)	Time not mentioned	p.142/f
Lim et al. (2013)	VEGF	VEGFA	hABMSCs (n.g./ n.g. (n.g.), n.g., n.g., P3-5, n.g./ n.g.)	Oscillatory laminar	5, 10, 30, 60, and 120min/d / n.g.	0.01-0.0205dyn/cm² (max. 0.00205Pa)	Rocking culture system (Vision Ltd.)			Increase with plateau (ELISA)	5min/d for 24h: 2845.3 pg/mL; 2845.3/2380.6 = 1.2 (ratio-calc)†<br>10min/d for 24h: 2689 pg/mL; 2689/2380.6 =1.12 (ratio-calc)†<br>30min/d for 24h: 2845.3 pg/mL; 2845.3/2380.6 = 1.2 (ratio-calc)†<br>60min/d for 24h: 2700 pg/mL; 2700/2380.6 = 1.13 (ratio-calc) †	
Lim et al. (2013)	VEGF	VEGFA	hABMSCs (n.g./ n.g. (n.g.), n.g., n.g., P3-5, n.g./ n.g.)	Oscillatory laminar	5, 10, 30, 60, and 120min/d / n.g.	0.01-0.0205dyn/cm² (max. 0.00205Pa)	Rocking culture system (Vision Ltd.)			Increase (antibody array)	Time not mentioned	p.142/c
Salvi et al. (2010)	Ca^{2+}	Calcium	Human mesenchymal stem cells (hMSCs, Cambrex PT-2501) (n.g./ n.g.(n.g.), n.g., n.g., n.g., 4×10³ cells per cm²/ n.g.)	Oscillatory laminar	180s/1Hz	5dyn/cm², 10dyn/cm², 20dyn/cm²	n.g.			Fluctuated increase (Fluorescence microscopy, Fura Red-AM dye)		
Celil Aydemir et al. (2010)	Cox2	PTGS2	Human mesenchymal stem cells (hMSC) (Cambrex Inc., Walkersville, MD) (n.g./ n.g. (n.g.), n.g., n.g., n.g., n.g./ n.g.)	Oscillatory laminar	15min/1Hz	16dyn/cm² (1.6Pa)	Flexcell Inc.	Increase (RT-qPCR, GAPDH)	15min: 2.8493 (rel.)†	Increase (WB, GAPDH)	15min: 145/79 = 1.84 (ratio-calc)†	See Celil Aydemir et al. (2007): similar system.
Celil Aydemir et al. (2010)	NFAT2	NFATC1	Human mesenchymal stem cells (hMSC) (Cambrex Inc., Walkersville, MD) (n.g./ n.g. (n.g.), n.g., n.g., n.g., n.g./ n.g.)	Oscillatory laminar	15min/1Hz	16dyn/cm² (1.6Pa)	Flexcell Inc.			Increase (colorimetric assay)	15min: 2.2/0.9 = 2.44 (ratio-calc)†	
Celil Aydemir et al. (2007)	Cox2	PTGS2	hMSCs were purchased from Cambrex (East Rutherford, NJ, USA) (n.g./ n.g. (n.g.), n.g., n.g., n.g., n.g./n.g.)	Oscillatory laminar	15min/1Hz	5–10dyn/cm²	Flexcell Inc.	Increase (RT-qPCR, GAPDH)	15min: 2.3 (rel.)			
Hoey et al. (2012)	BMP2	BMP2	MSCs harvested from human bone marrow (Lonza, Walkersville, MD, USA) (n.g./ n.g. (n.g.), n.g., n.g., P4, n.g./ n.g.)	Oscillatory laminar	2h (sampling 30min, 2h, 24h, 48h post-FSS)/n.g.	1.0Pa (28ml/min)	Custom-made	Increase followed by decrease then baseline (RT-qPCR; GAPDH)	2h post-FSS: 4.6/0.9 = 5.1 (ratio-calc)†<br>24h post-FSS: 0.2/0.3= 0.67 (ratio-calc)†<br>48h post-FSS: 0.35/0.35 = 1 (ratio-calc)†			
Hoey et al. (2012)	COX2	PTGS2	MSCs harvested from human bone marrow (Lonza, Walkersville, MD, USA) (n.g./ n.g. (n.g.), n.g., n.g., P4, n.g./ n.g.)	Oscillatory laminar	2h (sampling 30min, 2h, 24h, 48h post-FSS)/n.g.	1.0Pa (28ml/min)	Custom-made	Increase followed by decrease then baseline (RT-qPCR; GAPDH)	30min post-FSS: 1.7/0.17 = 10 (ratio-calc)†<br>24h post-FSS: 1 (ratio-calc)†<br>48h post-FSS: 1 (ratio-calc)†			
Hoey et al. (2012)	GLI1	GLI1	MSCs harvested from human bone marrow (Lonza, Walkersville, MD, USA) (n.g./ n.g. (n.g.), n.g., n.g., P4, n.g./ n.g.)	Oscillatory laminar	2h (sampling 30min, 2h, 24h, 48h post-FSS)/n.g.	1.0Pa (28ml/min)	Custom-made	Decrease (RT-qPCR; GAPDH)	2h post-FSS: 0.7/1.1 = 0.64 (ratio-calc)†			

[a] Entry provided as reported in the given study.
[b] Human genes were confirmed with the HUGO Gene Nomenclature Committee (HGNC; URL: https://www.genenames.org); mouse genes were confirmed with the Mouse Genome Informatics (MGI; URL: https://www.informatics.jax.org/genes.shtml) after checking the specificity of primers with Primer-BLAST.
[c] Sex of donors: "M" – male, "F" – female; Tooth type: "PM" – premolar, "M" – molar; dig. Indicate isolation by cell digestion; Exp. indicate isolation by cell explant; Cell density: given in cells/cm² if not otherwise mentioned.
[d] Flow type deduced from the description of the FSS apparatus given by the authors.
[f] RT-qPCR (reverse-transcriptase quantitative polymerase chain reaction); sqPCR (semi-quantitative polymerase chain reaction); ELISA (enzyme-linked immuno absorbent assay); WB (western blotting); RIA (radioimmunoassay); EMSA (electromobility shift assay); IF (immunofluorescence)

rel.: indicate relative gene expression. Is entitled to percentages or gene expression ratios normalized to control, and not calculated by ΔΔCT.
FC: indicate fold change. When Author mentions the use of ΔΔCT or the method according to Livak & Schmittken (2004) in calculating FC.
n.g.: not given. For information not given by study-authors.
† Information derived from figures using Engauge Digitizer.
* Indicate manual calculations by measuring the graphs, without using the Engauge Digitizer.
ratio-calc: indicate manual calculation by dividing intervention/control = result (ratio-calc)
ratio: indicate ratios given by study-authors such as normalization to control in case of small molecules data or in case of gene expression ratios, e.g. ratio of RANKL/OPG or Bcl-2/Bax.

Reference	Gene or analyte[a]	Official gene symbol or abbreviation[b]	Cell type (age/number and sex of donor (health status), tooth type, isolation method, passages used, cell density/confluency)[a,c]	Flow type (steady laminar, pulsatile laminar, or oscillatory laminar)[a,d]	FSS duration and frequency (Hz)[a]	FSS magnitude[a]	FSS apparatus[a]	Gene expression: increase, decrease, no change (method w/ reference gene); methods: (RT-qPCR, sqPCR)[f]	Gene expression: when it reaches peak and peak's magnitude (fold change; relative gene expression; times or ratio; unclear = ?)[f]	Protein/metabolite expression: increase, decrease, no change (method w/reference); methods: ELISA, WB, RIA, EMSA, IF[f]	Protein/metabolite expression: when it reaches peak and peak's magnitude (times or ratio; unclear = ?)[f]	Remarks
Hoey et al. (2012)	OPN	SPP1	MSCs harvested from human bone marrow (Lonza, Walkersville, MD, USA) (n.g./ n.g. (n.g.), n.g., n.g., P4, n.g./ n.g.)	Oscillatory laminar	2h (sampling 30min, 2h, 24h, 48h post-FSS)/n.g.	1.0Pa (28ml/min)	Custom-made	Increase then baseline (RT-qPCR; GAPDH)	30min post-FSS: 0.4/0.3 = 1.3 (ratio-calc)†<br>24h post-FSS: 1 (ratio-calc)†			
Hoey et al. (2012)	PTCH1	PTCH1	MSCs harvested from human bone marrow (Lonza, Walkersville, MD, USA) (n.g./ n.g. (n.g.), n.g., n.g., P4, n.g./ n.g.)	Oscillatory laminar	2h (sampling 30min, 2h, 24h, 48h post-FSS)/n.g.	1.0Pa (28ml/min)	Custom-made	Decrease (RT-qPCR; GAPDH)	2h post-FSS: 0.6/0.9 = 0.67 (ratio-calc)†			
Hoey et al. (2012)	RUNX2	RUNX2	MSCs harvested from human bone marrow (Lonza, Walkersville, MD, USA) (n.g./ n.g. (n.g.), n.g., n.g., P4, n.g./ n.g.)	Oscillatory laminar	2h (sampling 30min, 2h, 24h, 48h post-FSS)/n.g.	1.0Pa (28ml/min)	Custom-made	Decrease then increase with plateau (RT-qPCR; GAPDH)	2h post-FSS: 0.3/0.4 = 0.75 (ratio--calc)†<br>24h post-FSS: 0.8/0.7 = 1.14 (ratio-calc)†			
Kuo et al. (2015)	ARRAY		human bone marrow-derived MSCs (Lonza) (21y/1F (n.g.), n.g., n.g., P4-5, 6,000 cells per cm/ n.g.)	Oscillatory laminar	1h 0.5h,1h, 2h, 4h, 24h /1Hz (60/min)	0.5d±4dyn/cm²	Custom-made	Gene profiling was performed in cells under OS for 0, 0.5, 1, 2, 4, and 24 hours using an Affymetrix U133A 2.0 array (Affymetrix, Santa Clara, CA).				
Kuo et al. (2015)	NANOG	NANOG	human bone marrow-derived MSCs (Lonza) (21y/1F (n.g.), n.g., n.g., P4-5, 6,000 cells per cm/ n.g.)	Oscillatory laminar	1h 0.5h,1h, 2h, 4h, 24h /1Hz (60/min)	0.5d±4dyn/cm²	Custom-made	Temporary increase followed by decrease then increase (RT-qPCR, GAPDH)	0.5h: 1.7(rel.)†<br>1h: 0.8 (rel.)†<br>24h: 1.1 (rel.)†	0.5h: increase (WB, β-actin)<br>4h: decrease (WB, β-actin)	No quantitative information given.	
Kuo et al. (2015)	Oct4	POU5F1	human bone marrow-derived MSCs (Lonza) (21y/1F (n.g.), n.g., n.g., P4-5, 6,000 cells per cm/ n.g.)	Oscillatory laminar	1h 0.5h,1h, 2h, 4h, 24h /1Hz (60/min)	0.5d±4dyn/cm²	Custom-made	Decrease with plateau (RT-qPCR, GAPDH)	0.5-1h: 0.6 (rel.)†<br>2h: 0.66 (rel.)†			
Kuo et al. (2015)	p-MYPT1	PPP1R12A	human bone marrow-derived MSCs (Lonza) (21y/1F (n.g.), n.g., n.g., P4-5, 6,000 cells per cm/ n.g.)	Oscillatory laminar	1h 0.5h,1h, 2h, 4h, 24h /1Hz (60/min)	0.5d±4dyn/cm²	Custom-made			0.5h: decrease (WB, β-actin)	No quantitative information given.	
Kuo et al. (2015)	p-β-catenin	CTNNB1	human bone marrow-derived MSCs (Lonza) (21y/1F (n.g.), n.g., n.g., P4-5, 6,000 cells per cm/ n.g.)	Oscillatory laminar	1h 0.5h,1h, 2h, 4h, 24h /1Hz (60/min)	0.5d±4dyn/cm²	Custom-made			0.5h: increase (WB, β-actin)	No quantitative information given.	
Kuo et al. (2015)	PPAR	PPARA	human bone marrow-derived MSCs (Lonza) (21y/1F (n.g.), n.g., n.g., P4-5, 6,000 cells per cm/ n.g.)	Oscillatory laminar	1h 0.5h,1h, 2h, 4h, 24h /1Hz (60/min)	0.5d±4dyn/cm²	Custom-made	Temporary decrease then increase (RT-qPCR; GAPDH)	0.5h: 0.8 (rel.)†<br>24h: 3.6 (rel.)†			
Kuo et al. (2015)	RUNX2	RUNX2	human bone marrow-derived MSCs (Lonza) (21y/1F (n.g.), n.g., n.g., P4-5, 6,000 cells per cm/ n.g.)	Oscillatory laminar	1h 0.5h,1h, 2h, 4h, 24h /1Hz (60/min)	0.5d±4dyn/cm²	Custom-made	Increase (RT-qPCR; GAPDH)	2h: 3 (rel.)†			
Kuo et al. (2015)	SOX2	SOX2	human bone marrow-derived MSCs (Lonza) (21y/1F (n.g.), n.g., n.g., P4-5, 6,000 cells per cm/ n.g.)	Oscillatory laminar	1h 0.5h,1h, 2h, 4h, 24h /1Hz (60/min)	0.5d±4dyn/cm²	Custom-made	Temporary increase then decrease with plateau, followed by a baseline level then decrease (RT-qPCR, GAPDH)	0.5h: 1.4 (rel.)†<br>1-2h: 0.8 (rel.)†<br>4h: 1 (rel.) †<br>24h: 0.8 (rel.) †			
Kuo et al. (2015)	WIF	WIF1	human bone marrow-derived MSCs (Lonza) (21y/1F (n.g.), n.g., n.g., P4-5, 6,000 cells per cm/ n.g.)	Oscillatory laminar	1h 0.5h,1h, 2h, 4h, 24h /1Hz (60/min)	0.5d±4dyn/cm²	Custom-made	Temporary Increase then baseline (RT-qPCR; GAPDH)	0.5h: 1.4 (rel.)†<br>1-24h: 1 (rel.)†			
Riddle et al. (2006)	Ca^{2+}	Calcium	hMSCs (Cambrex Biosciences) (18y/ 1M (n.g.), n.g., n.g., n.g., n.g./ n.g.)	Oscillatory laminar	180s/1Hz	20dyn/cm²	Custom-made			Fluctuated increase (fura-2 microscopy)		
Riddle et al. (2006)	p-ERK1/2	MAPK3; MAPK1	hMSCs (Cambrex Biosciences) (18y/ 1M (n.g.), n.g., n.g., n.g., n.g./ n.g.)	Oscillatory laminar	15-120min/1Hz	20dyn/cm²	Custom-made			1min, 5min, 15min, 30min, 60min: increase (WB, total ERK)	No quantitative information given.	

[a] Entry provided as reported in the given study.
[b] Human genes were confirmed with the HUGO Gene Nomenclature Committee (HGNC; URL: https://www.genenames.org); mouse genes were confirmed with the Mouse Genome Informatics (MGI; URL: https://www.informatics.jax.org/genes.shtml) after checking the specificity of primers with Primer-BLAST.
[c] Sex of donors: "M" – male, "F" – female; Tooth type: "PM" – premolar, "M" – molar; dig. Indicate isolation by cell digestion; Exp. indicate isolation by cell explant; Cell density: given in cells/cm² if not otherwise mentioned.
[d] Flow type deduced from the description of the FSS apparatus given by the authors.
f RT-qPCR (reverse-transcriptase quantitative polymerase chain reaction); sqPCR (semi-quantitative polymerase chain reaction); ELISA (enzyme-linked immuno absorbent assay); WB (western blotting); RIA (radioimmunoassay); EMSA (electromobility shift assay); IF (immunofluorescence)

rel.: indicate relative gene expression. Is entitled to percentages or gene expression ratios normalized to control, and not calculated by ΔΔCT.
FC: indicate fold change. When Author mentions the use of ΔΔCT or the method according to Livak & Schmittken (2004) in calculating FC.
n.g.: not given. For information not given by study-authors.
† Information derived from figures using Engauge Digitizer.
* Indicate manual calculations by measuring the graphs, without using the Engauge Digitizer.
ratio-calc: indicate manual calculation by dividing intervention/control = result (ratio-calc)
ratio: indicate ratios given by study-authors such as normalization to control in case of small molecules data or in case of gene expression ratios, e.g. ratio of RANKL/OPG or Bcl-2/Bax.

Reference	Gene or analyte[a]	Official gene symbol or abbreviation[b]	Cell type (age/number and sex of donor (health status), tooth type, isolation method, passages used, cell density/confluency)[a,c]	Flow type (steady laminar, pulsatile laminar, or oscillatory laminar)[a,d]	FSS duration and frequency (Hz)[a]	FSS magnitude[a]	FSS apparatus[a]	Gene expression: increase, decrease, no change (method w/ reference gene); methods: (RT-qPCR, sqPCR)[f]	Gene expression: when it reaches peak and peak's magnitude (fold change; relative gene expression; times or ratio; unclear = ?)[f]	Protein/metabolite expression: increase, decrease, no change (method w/reference); methods: ELISA, WB, RIA, EMSA, IF[f]	Protein/metabolite expression: when it reaches peak and peak's magnitude (times or ratio; unclear = ?)[f]	Remarks
Lim et al. (2014)	ALP	ALP (unspecific)	hABMSCs (n.g./ n.g. (n.g.), n.g., n.g., n.g., 10^4 cells per cm^2/ n.g.)	Oscillatory laminar	10d @ 10, 30, 60, 120, 180min/d / n.g.	0.86–1.51dyn/cm^2	Rotational orbital shaker			Increase with plateau (activity)	30min/d: 6.1ng/h/protein; 6.1/4.7 = 1.3 (ratio-calc)† 60min/d: 5.5ng/h/protein; 5.5/4.7 = 1.17 (ratio-calc)† 120min/d: 5.6ng/h/protein; 5.6/4.7 = 1.19 (ratio-calc)†	ALP mentioned in footnote of Table 1; ALP activity shown in figure 6, but methodology not specified.
Lim et al. (2014)	BMP2	BMP2	hABMSCs (n.g./ n.g. (n.g.), n.g., n.g., n.g., 10^4 cells per cm^2/ n.g.)	Oscillatory laminar	10d @ 10, 30, 60, 120, 180min/d / n.g.	0.86–1.51dyn/cm^2	Rotational orbital shaker			Increase (ELISA)	120min/d: 2718.5pg/mL; 2718.5/1688.5 = 1.61 (ratio-calc)†	
Lim et al. (2014)	COL-1	COL1A1	hABMSCs (n.g./ n.g. (n.g.), n.g., n.g., n.g., 10^4 cells per cm^2/ n.g.)	Oscillatory laminar	10d @ 10, 30, 60, 120, 180min/d / n.g.	0.86–1.51dyn/cm^2	Rotational orbital shaker	Decrease then increase (sqPCR, GAPDH)	10min/d: 0.6 (rel.)† 180min/d: 1.3 (rel.)†			
Lim et al. (2014)	OCN	BGLAP	hABMSCs (n.g./ n.g. (n.g.), n.g., n.g., n.g., 10^4 cells per cm^2/ n.g.)	Oscillatory laminar	10d @ 10, 30, 60, 120, 180min/d / n.g.	0.86–1.51dyn/cm^2	Rotational orbital shaker	Temporary decrease then increase (sqPCR, GAPDH)	10min/d: 1.0 (rel.)† 30min/d: 1.9 (rel.)†			
Lim et al. (2014)	OPN	SPP1	hABMSCs (n.g./ n.g. (n.g.), n.g., n.g., n.g., 10^4 cells per cm^2/ n.g.)	Oscillatory laminar	10d @ 10, 30, 60, 120, 180min/d / n.g.	0.86–1.51dyn/cm^2	Rotational orbital shaker	Increase, decrease then increase (sqPCR, GAPDH)	30min/d: 2.3 (rel.)† 60min/d: 0.8 (rel.)† 180min/d: 1.9 (rel.)†			
Lim et al. (2014)	RUNX2	RUNX2	hABMSCs (n.g./ n.g. (n.g.), n.g., n.g., n.g., 10^4 cells per cm^2/ n.g.)	Oscillatory laminar	10d @ 10, 30, 60, 120, 180min/d / n.g.	0.86–1.51dyn/cm^2	Rotational orbital shaker	Decrease with plateau then increase (sqPCR, GAPDH)	30min/d: 0.6 (rel.)† 180min/d: 1.3 (rel.)†			
Lim et al. (2014)	VEGF	VEGFA	hABMSCs (n.g./ n.g. (n.g.), n.g., n.g., n.g., 10^4 cells per cm^2/ n.g.)	Oscillatory laminar	10d @ 10, 30, 60, 120, 180min/d / n.g.	0.86–1.51dyn/cm^2	Rotational orbital shaker			Increase (ELISA)	60min/d: 4193.2pg/mL; 4193.2/2537.2 = 1.65 (ratio-calc)†	
Becquart et al. (2016)	ALP	ALPL	Human MSCs (n.g./ 1 and 6 n.g. (n.g.), n.g., Ficoll paque, P2, cell n.g./ n.g.)	Steady laminar	30min (sampling 0h, 1h, 6h post-FSS) / 2.8Hz	0.7Pa	µ-Slide w/ Ibidi pump system (Ibidi, DE)	Increase (RT-qPCR, 18S)	1h post-FSS: 2.1 (FC)†			
Becquart et al. (2016)	EGR1	EGR1	Human MSCs (n.g./ 1 and 6 n.g. (n.g.), n.g., Ficoll paque, P2, cell n.g./ n.g.)	Steady laminar	30min (sampling 0h, 1h, 6h post-FSS) / 2.8Hz	0.7Pa	µ-Slide w/ Ibidi pump system (Ibidi, DE)	Increase (RT-qPCR, 18S)	0h post-FSS: 14.6 (FC)†			
Becquart et al. (2016)	FGF2	FGF2	Human MSCs (n.g./ 1 and 6 n.g. (n.g.), n.g., Ficoll paque, P2, cell n.g./ n.g.)	Steady laminar	30min (sampling 0h, 1h, 6h post-FSS) / 2.8Hz	0.7Pa	µ-Slide w/ Ibidi pump system (Ibidi, DE)	Increase (RT-qPCR, 18S)	1h post-FSS: 16.5 (FC)†			
Becquart et al. (2016)	HIF1A	HIF1A	Human MSCs (n.g./ 1 and 6 n.g. (n.g.), n.g., Ficoll paque, P2, cell n.g./ n.g.)	Steady laminar	30min (sampling 0h, 1h, 6h post-FSS) / 2.8Hz	0.7Pa	µ-Slide w/ Ibidi pump system (Ibidi, DE)	Increase (RT-qPCR, 18S)	0h post-FSS: 2.1 (FC)† 1h post-FSS: 2.2 (FC)†			
Becquart et al. (2016)	IER3	IER3	Human MSCs (n.g./ 1 and 6 n.g. (n.g.), n.g., Ficoll paque, P2, cell n.g./ n.g.)	Steady laminar	30min (sampling 0h, 1h, 6h post-FSS) / 2.8Hz	0.7Pa	µ-Slide w/ Ibidi pump system (Ibidi, DE)	Increase (RT-qPCR, 18S)	1h post-FSS: 17.6 (FC)†			
Becquart et al. (2016)	IGF1	IGF1	Human MSCs (n.g./ 1 and 6 n.g. (n.g.), n.g., Ficoll paque, P2, cell n.g./ n.g.)	Steady laminar	30min (sampling 0h, 1h, 6h post-FSS) / 2.8Hz	0.7Pa	µ-Slide w/ Ibidi pump system (Ibidi, DE)	Increase (RT-qPCR, 18S)	1h post-FSS: 17 (FC)†			
Becquart et al. (2016)	IGFBP1	IGFBP1	Human MSCs (n.g./ 1 and 6 n.g. (n.g.), n.g., Ficoll paque, P2, cell n.g./ n.g.)	Steady laminar	30min (sampling 0h, 1h, 6h post-FSS) / 2.8Hz	0.7Pa	µ-Slide w/ Ibidi pump system (Ibidi, DE)	Increase (RT-qPCR, 18S)	1h post-FSS: 38.7 (FC)†			
Becquart et al. (2016)	ITGB1	ITGB1	Human MSCs (n.g./ 1 and 6 n.g. (n.g.), n.g., Ficoll paque, P2, cell n.g./ n.g.)	Steady laminar	30min (sampling 0h, 1h, 6h post-FSS) / 2.8Hz	0.7Pa	µ-Slide w/ Ibidi pump system (Ibidi, DE)	Increase (RT-qPCR, 18S)	1h post-FSS: 7.9 (FC)†			
Becquart et al. (2016)	NO	Nitric oxide	Human MSCs (n.g./ 1 and 6 n.g. (n.g.), n.g., Ficoll paque, P2, cell n.g./ n.g.)	Steady laminar	30min (sampling 0h, 1h, 6h post-FSS) / 2.8Hz	0.1Pa, 0.7Pa, 2.1Pa, 4.2Pa	µ-Slide w/ Ibidi pump system (Ibidi, DE)			Increase (Griess, NO_2^- + colorimetric assay)	0.7Pa @ 0h post-FSS: 24.5µM; 24.5/1.6 = 15.31 (ratio-calc)†	
Becquart et al. (2016)	p-ERK1/2	MAPK3; MAPK1	Human MSCs (n.g./ 1 and 6 n.g. (n.g.), n.g., Ficoll paque, P2, cell n.g./ n.g.)	Steady laminar	30min (sampling 0h, 1h, 6h post-FSS) / 2.8Hz	0.7Pa	µ-Slide w/ Ibidi pump system (Ibidi, DE)			Increase (WB, β-tubulin)	0.7Pa @ 0h post-FSS: 9.5 (ratio)†	
Becquart et al. (2016)	PTGES	PTGES	Human MSCs (n.g./ 1 and 6 n.g. (n.g.), n.g., Ficoll paque, P2, cell n.g./ n.g.)	Steady laminar	30min (sampling 0h, 1h, 6h post-FSS) / 2.8Hz	0.7Pa	µ-Slide w/ Ibidi pump system (Ibidi, DE)	Increase (RT-qPCR, 18S)	0h post-FSS: 1.5 (FC)†			

[a] Entry provided as reported in the given study.
[b] Human genes were confirmed with the HUGO Gene Nomenclature Committee (HGNC; URL: https://www.genenames.org); mouse genes were confirmed with the Mouse Genome Informatics (MGI; URL: https://www.informatics.jax.org/genes.shtml) after checking the specificity of primers with Primer-BLAST.
[c] Sex of donors: "M" – male, "F" – female; Tooth type: "PM" – premolar, "M" – molar; dig. Indicate isolation by cell digestion; Exp. indicate isolation by cell explant; Cell density: given in cells/cm^2 if not otherwise mentioned.
[d] Flow type deduced from the description of the FSS apparatus given by the authors.
[f] RT-qPCR (reverse-transcriptase quantitative polymerase chain reaction); sqPCR (semi-quantitative polymerase chain reaction); ELISA (enzyme-linked immuno absorbent assay); WB (western blotting); RIA (radioimmunoassay); EMSA (electromobility shift assay); IF (immunofluorescence)

rel.: indicate relative gene expression. Is entitled to percentages or gene expression ratios normalized to control, and not calculated by ΔΔCT.
FC: indicate fold change. When Author mentions the use of ΔΔCT or the method according to Livak & Schmittken (2004) in calculating FC.
n.g.: not given. For information not given by study-authors.
† Information derived from figures using Engauge Digitizer.
* Indicate manual calculations by measuring the graphs, without using the Engauge Digitizer.
ratio-calc: indicate manual calculation by dividing intervention/control = result (ratio-calc)
ratio: indicate ratios given by study-authors such as normalization to control in case of small molecules data or in case of gene expression ratios, e.g. ratio of RANKL/OPG or Bcl-2/Bax.

Reference	Gene or analyte[a]	Official gene symbol or abbreviation[b]	Cell type (age/number and sex of donor (health status), tooth type, isolation method, passages used, cell density/confluency)[a,c]	Flow type (steady laminar, pulsatile laminar, or oscillatory laminar)[a,d]	FSS duration and frequency (Hz)[a]	FSS magnitude[a]	FSS apparatus[a]	Gene expression: increase, decrease, no change (method w/ reference gene); methods: (RT-qPCR, sqPCR)[f]	Gene expression: when it reaches peak and peak's magnitude (fold change; relative gene expression; times or ratio; unclear = ?)[f]	Protein/metabolite expression: increase, decrease, no change (method w/reference); methods: ELISA, WB, RIA, EMSA, IF[f]	Protein/metabolite expression: when it reaches peak and peak's magnitude (times or ratio; unclear = ?)[f]	Remarks
Becquart et al. (2016)	PTGS2	PTGS2	Human MSCs (n.g./ 1 and 6 n.g. (n.g.), n.g., Ficoll paque, P2, cell n.g./ n.g.)	Steady laminar	30min (sampling 0h, 1h, 6h post-FSS) / 2.8Hz	0.7Pa	μ-Slide w/ Ibidi pump system (Ibidi, DE)	Increase (RT-qPCR, 18S)	1h post-FSS: 56.7 (FC)†			
Becquart et al. (2016)	RUNX2	RUNX2	Human MSCs (n.g./ 1 and 6 n.g. (n.g.), n.g., Ficoll paque, P2, cell n.g./ n.g.)	Steady laminar	30min (sampling 0h, 1h, 6h post-FSS) / 2.8Hz	0.7Pa	μ-Slide w/ Ibidi pump system (Ibidi, DE)	Increase (RT-qPCR, 18S)	0h post-FSS: 1.9 (FC)†			
Becquart et al. (2016)	VEGFA	VEGFA	Human MSCs (n.g./ 1 and 6 n.g. (n.g.), n.g., Ficoll paque, P2, cell n.g./ n.g.)	Steady laminar	30min (sampling 0h, 1h, 6h post-FSS) / 2.8Hz	0.7Pa	μ-Slide w/ Ibidi pump system (Ibidi, DE)	Increase (RT-qPCR, 18S)	1h post-FSS: 14 (FC)†			
Lee et al. (2017)	AKT; p-Akt	AKT1	Bone marrow MSCs (n.g./ n.g. (n.g.), n.g., Ficoll paque, P1, 3×10^6 cells per ml / 80%)	Steady laminar	5min, 1h, 3h / n.g.	$15dyn/cm^2$	μ-slide $VI^{0.4}$ (Ibidi, DE) w/ fibronectin coating; 12-roller peristaltic pump (REGLO analog MS4/12, Ismatec)			p-AKT: 5min: increase (WB, AKT)	p-AKT/AKT @ 5min: 7.0 (ratio)†	
Lee et al. (2017)	Ca^{2+}	Calcium	Bone marrow MSCs (n.g./ n.g. (n.g.), n.g., Ficoll paque, P1, 3×10^6 cells per ml / 80%)	Steady laminar	70s/n.g.	$15dyn/cm^2$	μ-slide $VI^{0.4}$ (Ibidi, DE) w/ fibronectin coating; 12-roller peristaltic pump (REGLO analog MS4/12, Ismatec)			Fluctuated increase (Fluo-4 AM microscopy)		
Lee et al. (2017)	COX2	PTGS2	Bone marrow MSCs (n.g./ n.g. (n.g.), n.g., Ficoll paque, P1, 3×10^6 cells per ml / 80%)	Steady laminar	5min, 6h / n.g.	$15dyn/cm^2$	μ-slide $VI^{0.4}$ (Ibidi, DE) w/ fibronectin coating; 12-roller peristaltic pump (REGLO analog MS4/12, Ismatec)			Decrease then increase (WB, β-actin)	5min: 0.9 (ratio)†<br>6h: 2.4 (ratio)†	
Lee et al. (2017)	ERK; p-ERK	MAPK3; MAPK1	Bone marrow MSCs (n.g./ n.g. (n.g.), n.g., Ficoll paque, P1, 3×10^6 cells per ml / 80%)	Steady laminar	5min, 1h, 3h / n.g.	$15dyn/cm^2$	μ-slide $VI^{0.4}$ (Ibidi, DE) w/ fibronectin coating; 12-roller peristaltic pump (REGLO analog MS4/12, Ismatec)			p-ERK @ 5min: increase (WB, ERK)	p-ERK/ERK @ 5 min: 3.4 (ratio)†	
Lee et al. (2017)	FAK; p-FAK	PTK2	Bone marrow MSCs (n.g./ n.g. (n.g.), n.g., Ficoll paque, P1, 3×10^6 cells per ml / 80%)	Steady laminar	5min, 30min, 6h / n.g.	$15dyn/cm^2$	μ-slide $VI^{0.4}$ (Ibidi, DE) w/ fibronectin coating; 12-roller peristaltic pump (REGLO analog MS4/12, Ismatec)			p-FAK @ 5min: decrease (WB, FAK)	p-FAK/FAK @ 5min: 0.9 (ratio)†	
Lee et al. (2017)	HMOX1	HMOX1	Bone marrow MSCs (n.g./ n.g. (n.g.), n.g., Ficoll paque, P1, 3×10^6 cells per ml / 80%)	Steady laminar	WB: 5min, 6h / n.g.; RT-qPCR: 3h, 6h / n.g.	$15dyn/cm^2$	μ-slide $VI^{0.4}$ (Ibidi, DE) w/ fibronectin coating; 12-roller peristaltic pump (REGLO analog MS4/12, Ismatec)	Increase (RT-qPCR, GAPDH)	6h: 6.3 (FC)†	5min-6h: no change (WB, β-actin)	No change	
Lee et al. (2017)	IL1RN	IL1RN	Bone marrow MSCs (n.g./ n.g. (n.g.), n.g., Ficoll paque, P1, 3×10^6 cells per ml / 80%)	Steady laminar	5min, 6h / n.g.	$15dyn/cm^2$	μ-slide $VI^{0.4}$ (Ibidi, DE) w/ fibronectin coating; 12-roller peristaltic pump (REGLO analog MS4/12, Ismatec)	Increase (RT-qPCR, GAPDH)	6h: 9.8 (FC)†	6h: decrease (WB, β-actin)	6h: 0.8 (ratio)*	

[a] Entry provided as reported in the given study.
[b] Human genes were confirmed with the HUGO Gene Nomenclature Committee (HGNC; URL: https://www.genenames.org); mouse genes were confirmed with the Mouse Genome Informatics (MGI; URL: https://www.informatics.jax.org/genes.shtml) after checking the specificity of primers with Primer-BLAST.
[c] Sex of donors: "M" – male, "F" – female; Tooth type: "PM" – premolar, "M" – molar; dig. Indicate isolation by cell digestion; Exp. indicate isolation by cell explant; Cell density: given in cells/cm^2 if not otherwise mentioned.
[d] Flow type deduced from the description of the FSS apparatus given by the authors.
[f] RT-qPCR (reverse-transcriptase quantitative polymerase chain reaction); sqPCR (semi-quantitative polymerase chain reaction); ELISA (enzyme-linked immuno absorbent assay); WB (western blotting); RIA (radioimmunoassay); EMSA (electromobility shift assay); IF (immunofluorescence)

rel.: indicate relative gene expression. Is entitled to percentages or gene expression ratios normalized to control, and not calculated by ΔΔCT.
FC: indicate fold change. When Author mentions the use of ΔΔCT or the method according to Livak & Schmittken (2004) in calculating FC.
n.g.: not given. For information not given by study-authors.
† Information derived from figures using Engauge Digitizer.
* Indicate manual calculations by measuring the graphs, without using the Engauge Digitizer.
ratio-calc: indicate manual calculation by dividing intervention/control = result (ratio-calc)
ratio: indicate ratios given by study-authors such as normalization to control in case of small molecules data or in case of gene expression ratios, e.g. ratio of RANKL/OPG or Bcl-2/Bax.

Reference	Gene or analyte[a]	Official gene symbol or abbreviation[b]	Cell type (age/number and sex of donor (health status), tooth type, isolation method, passages used, cell density/confluency)[a,c]	Flow type (steady laminar, pulsatile laminar, or oscillatory laminar)[a,d]	FSS duration and frequency (Hz)[a]	FSS magnitude[a]	FSS apparatus[a]	Gene expression: increase, decrease, no change (method w/ reference gene); methods: (RT-qPCR, sqPCR)[f]	Gene expression: when it reaches peak and peak's magnitude (fold change; relative gene expression; times or ratio; unclear = ?)[f]	Protein/metabolite expression: increase, decrease, no change (method w/reference); methods: ELISA, WB, RIA, EMSA, IF[f]	Protein/metabolite expression: when it reaches peak and peak's magnitude (times or ratio; unclear = ?)[f]	Remarks
Lee et al. (2017)	PTGS2	PTGS2	Bone marrow MSCs (n.g./ n.g. (n.g.), n.g., Ficoll paque, P1, 3×10^6 cells per ml / 80%)	Steady laminar	3h, 6h / n.g.	15dyn/cm^2	μ-slide VI$^{0.4}$ (Ibidi, DE) w/ fibronectin coating; 12-roller peristaltic pump (REGLO analog MS4/12, Ismatec)	Increase (RT-qPCR, GAPDH)	6h: 5.3 (FC)†			
Lee et al. (2017)	TNFAIP6	TNFAIP6	Bone marrow MSCs (n.g./ n.g. (n.g.), n.g., Ficoll paque, P1, 3×10^6 cells per ml / 80%)	Steady laminar	3h, 6h / n.g.	15dyn/cm^2	μ-slide VI$^{0.4}$ (Ibidi, DE) w/ fibronectin coating; 12-roller peristaltic pump (REGLO analog MS4/12, Ismatec)	Increase (RT-qPCR, GAPDH)	6h: 2.5 (FC)†	6h: no change (WB, β-actin)	No change	

References

Becquart P, Cruel M, Hoc T, Sudre L, Pernelle K, Bizios R, Logeart-Avramoglou D, Petite H, Bensidhoum M (2016). Human mesenchymal stem cell responses to hydrostatic pressure and shear stress. Eur Cell Mater; 31:160-73.

Celil Aydemir AB, Lee S, Won Kim D, Gardner TR, Prince D, Mok Ahn J, Lee FY (2007). Nuclear factor of activated T cell mediates proinflammatory gene expression in response to mechanotransduction. Ann N Y Acad Sci; 1117:138-42.

Celil Aydemir AB, Minematsu H, Gardner TR, Kim KO, Ahn JM, Lee FY (2010). Nuclear factor of activated T cells mediates fluid shear stress- and tensile strain-induced Cox2 in human and murine bone cells. Bone; 46(1):167-75.

Charoenpong H, Osathanon T, Pavasant P, Limjeerajarus N, Keawprachum B, Limjeerajarus CN, Cheewinthamrongrod V, Palaga T, Lertchirakarn V, Ritprajak P (2019). Mechanical stress induced S100A7 expression in human dental pulp cells to augment osteoclast differentiation. Oral Dis; 25(3):812-821.

Glossop JR, Cartmell SH (2009). Effect of fluid flow-induced shear stress on human mesenchymal stem cells: differential gene expression of IL1B and MAP3K8 in MAPK signaling. Gene Expression Patterns; 9(5):381-8.

Hoey DA, Tormey S, Ramcharan S, O'Brien FJ, Jacobs CR (2012). Primary cilia-mediated mechanotransduction in human mesenchymal stem cells. Stem Cells; 30(11):2561-70.

Hu K, Sun H, Gui B, Sui C (2017). TRPV4 functions in flow shear stress induced early osteogenic differentiation of human bone marrow mesenchymal stem cells. Biomed Pharmacother; 91:841-848.

Kim DH, Heo SJ, Kim SH, Shin JW, Park SH, Shin JW (2011). Shear stress magnitude is critical in regulating the differentiation of mesenchymal stem cells even with endothelial growth medium. Biotechnol Lett; 33(12):2351-9.

Kraft DC, Bindslev DA, Melsen B, Abdallah BM, Kassem M, Klein-Nulend J (2010). Mechanosensitivity of dental pulp stem cells is related to their osteogenic maturity. Eur J Oral Sci; 118(1):29-38.

Kraft DC, Bindslev DA, Melsen B, Klein-Nulend J (2011). Human dental pulp cells exhibit bone cell-like responsiveness to fluid shear stress. Cytotherapy; 13(2):214-26.

Kuo YC, Chang TH, Hsu WT, Zhou J, Lee HH, Hui-Chun Ho J, Chien S, Lee OK (2015). Oscillatory shear stress mediates directional reorganization of actin cytoskeleton and alters differentiation propensity of mesenchymal stem cells. Stem Cells; 33(2):429-42.

Lee HJ, Diaz MF, Ewere A, Olson SD, Cox CS, Jr., Wenzel PL (2017). Focal adhesion kinase signaling regulates anti-inflammatory function of bone marrow mesenchymal stromal cells induced by biomechanical force. Cell Signal; 38:1-9.

Li YJ, Batra NN, You L, Meier SC, Coe IA, Yellowley CE, Jacobs CR (2004). Oscillatory fluid flow affects human marrow stromal cell proliferation and differentiation. J Orthop Res; 22(6):1283-9.

Lim KT, Kim J, Seonwoo H, Chang JU, Choi H, Hexiu J, Cho WJ, Choung PH, Chung JH (2013). Enhanced osteogenesis of human alveolar bone-derived mesenchymal stem cells for tooth tissue engineering using fluid shear stress in a rocking culture method. Tissue Eng Part C Methods; 19(2):128-45.

Lim KT, Hexiu J, Kim J, Seonwoo H, Choung PH, Chung JH (2014). Synergistic effects of orbital shear stress on in vitro growth and osteogenic differentiation of human alveolar bone-derived mesenchymal stem cells. Biomed Res Int; 2014:316803.

Riddle RC, Taylor AF, Genetos DC, Donahue HJ (2006). MAP kinase and calcium signaling mediate fluid flow-induced human mesenchymal stem cell proliferation. Am J Physiol Cell Physiol; 290(3):C776-84.

Riddle RC, Taylor AF, Rogers JR, Donahue HJ (2007). ATP release mediates fluid flow-induced proliferation of human bone marrow stromal cells. J Bone Miner Res; 22(4):589-600.

Salvi JD, Lim JY, Donahue HJ (2010). Increased mechanosensitivity of cells cultured on nanotopographies. J Biomech; 43(15):3058-62.

Sonam S, Sathe SR, Yim EK, Sheetz MP, Lim CT (2016). Cell contractility arising from topography and shear flow determines human mesenchymal stem cell fate. Scientific Reports; 6:20415.

Yourek G, McCormick SM, Mao JJ, Reilly GC (2010). Shear stress induces osteogenic differentiation of human mesenchymal stem cells. Regen Med; 5(5):713-24.

Yuan L, Sakamoto N, Song G, Sato M (2012). Migration of human mesenchymal stem cells under low shear stress mediated by mitogen-activated protein kinase signaling. Stem Cells Dev; 21(13):2520-30.

[a] Entry provided as reported in the given study.
[b] Human genes were confirmed with the HUGO Gene Nomenclature Committee (HGNC; URL: https://www.genenames.org); mouse genes were confirmed with the Mouse Genome Informatics (MGI; URL: https://www.informatics.jax.org/genes.shtml) after checking the specificity of primers with Primer-BLAST.
[c] Sex of donors: "M" – male, "F" – female; Tooth type: "PM" – premolar, "M" – molar; dig. Indicate isolation by cell digestion; Exp. indicate isolation by cell explant; Cell density: given in cells/cm^2 if not otherwise mentioned.
[d] Flow type deduced from the description of the FSS apparatus given by the authors.
f RT-qPCR (reverse-transcriptase quantitative polymerase chain reaction); sqPCR (semi-quantitative polymerase chain reaction); ELISA (enzyme-linked immuno absorbent assay); WB (western blotting); RIA (radioimmunoassay); EMSA (electromobility shift assay); IF (immunofluorescence)

rel.: indicate relative gene expression. Is entitled to percentages or gene expression ratios normalized to control, and not calculated by ΔΔCT.
FC: indicate fold change. When Author mentions the use of ΔΔCT or the method according to Livak & Schmittken (2004) in calculating FC.
n.g.: not given. For information not given by study-authors.
† Information derived from figures using Engauge Digitizer.
* Indicate manual calculations by measuring the graphs, without using the Engauge Digitizer.
ratio-calc: indicate manual calculation by dividing intervention/control = result (ratio-calc)
ratio: indicate ratios given by study-authors such as normalization to control in case of small molecules data or in case of gene expression ratios, e.g. ratio of RANKL/OPG or Bcl-2/Bax.

2.2 Human osteoblasts

Reference	Gene or analyte[a]	Official gene symbol or abbreviation[b]	Cell Type ((age and sex)/number and sex of donor (health status), tooth type, isolation method, passages used, cell density / confluency)[a,c]	Flow type (steady laminar, pulsatile laminar, or oscillatory laminar)[a,d]	FSS-duration and frequency[a]	FSS magnitude[a]	FSS apparatus[a]	Gene expression: Increase, decrease, no change (method w/ reference gene); methods: RT-qPCR, sqPCR[f]	Gene expression: when it reaches peak and peak's magnitude (fold change; relative gene expression; times or ratio; unclear = ?)[f]	Protein expression: Increase, decrease, no change (method w/ reference); methods: ELISA, WB, RIA, EMSA, IF[f]	Protein expression: When it reaches peak and peak's magnitude (times or ratio; unclear = ?)[f]	Remarks
Joldersma et al. (2001)	COX-1	PTGS1	Transiliac bone biopsies ((56-75y females)/7F(Healthy), n.g., dig., n.g., 25×10^3 cells per cm^2/ n.g.)	Pulsatile laminar (roller pump)	1h @ 5Hz	0.6±0.3Pa, peak stress rate 8.4Pa/s	Custom-made	Increase (sqPCR, GAPDH)	1h: 2.9 (rel.)†			
Joldersma et al. (2001)	COX-2	PTGS2	Transiliac bone biopsies ((56-75y females)/7F(Healthy), n.g., dig., n.g., 25×10^3 cells per cm^2/ n.g.)	Pulsatile laminar (roller pump)	1h @ 5Hz	0.6±0.3Pa, peak stress rate 8.4Pa/s	Custom-made	Increase (sqPCR, GAPDH)	1h: 1.3 (rel.)†			
Joldersma et al. (2001)	PGE_2	PGE2	Transiliac bone biopsies ((56-75y females)/7F(Healthy), n.g., dig., n.g., 25×10^3 cells per cm^2/ n.g.)	Pulsatile laminar (roller pump)	1h @ 5Hz	0.6±0.3Pa, peak stress rate 8.4Pa/s	Custom-made			Mean increase (EIA)	1h: 1504.1pg/µgDNA; 1504.1/491.7 = 3 (ratio-calc)†	
Joldersma et al. (2001)	$PGF_{2\alpha}$	PGF2alpha	Transiliac bone biopsies ((56-75y females)/7F(Healthy), n.g., dig., n.g., 25×10^3 cells per cm^2/ n.g.)	Pulsatile laminar (roller pump)	1h @ 5Hz	0.6±0.3Pa, peak stress rate 8.4Pa/s	Custom-made			Mean increase (RIA)	1h: 196.99pg/µgDNA; 196.99/163.5 = 1.2 (ratio-calc)†	
Joldersma et al. (2001)	PGI_2	PGI2	Transiliac bone biopsies ((56-75y females)/7F(Healthy), n.g., dig., n.g., 25×10^3 cells per cm^2/ n.g.)	Pulsatile laminar (roller pump)	1h @ 5Hz	0.6±0.3Pa, peak stress rate 8.4Pa/s	Custom-made			Mean increase (EIA)	1h: 320.2pg/µgDNA; 320.2/155.3 = 2 (ratio-calc)†	
Klein-Nulend et al. (2002)	PGE2	PGE2	Iliac crest bone biopsies ((7-85y males and females)/22M(healthy) and 17F(healthy), n.g., dig., P2, 5×10^5 cells per cm^2/ n.g.)	Pulsating laminar	1h (sampling 0h, 1h, 24h post-PFF) @ 5Hz	Mean SS 0.7±0.3Pa, peak stress rate of 9.5Pa/s	Custom-made			Increase (EIA)	0h post-PFF: 178.4ng/mg protein; 178.4/70.9 = 2.5 (ratio-calc)† 1h post-PFF: 24.2/16.4 = 1.4 (ratio-calc) 24h post-PFF: 106.8/54.4 =1.9 (ratio-calc)	
Klein-Nulend et al. (2002)	PGI2	PGI2	Iliac crest bone biopsies ((7-85y males and females)/22M(healthy) and 17F(healthy), n.g., dig., P2, 5×10^5 cells per cm^2/ n.g.)	Pulsating laminar	1h (sampling 0h, 1h, 24h post-PFF) @ 5Hz	Mean SS 0.7±0.3Pa, peak stress rate of 9.5Pa/s	Custom-made			Increase (EIA)	0h post-PFF: 10.6ng/mg protein; 10.6/6.5 1.6 (ratio-calc)† 1h post-PFF: 0.8/0.6 = 1.3 (ratio-calc) 24h post-PFF: 2.9/1.7 = 1.7 (ratio-calc)	
Bakker et al. (2004)	Bax	BAX	Human trabecular bone samples (60–84y female)/7F(healthy), n.g, dig., n.g., 5×10^5 cells per cm^2/n.g.)	Pulsatile laminar	1h @ (3Hz, 5Hz, 9Hz)	0.4±0.1Pa mean SS @ 3Hz; 0.6±0.3Pa mean SS @ 5Hz; 1.2±0.4Pa mean SS @ 9Hz	Custom-made	Increase (RT-qPCR, PBGD)	1h @ 0.6-1.2Pa: 1.2432 (rel.)†			
Bakker et al. (2004)	Bcl-2	BCL2	Human trabecular bone samples (60–84y female)/7F(healthy), n.g, dig., n.g., 5×10^5 cells per cm^2/n.g.)	Pulsatile laminar	1h @ (3Hz, 5Hz, 9Hz)	0.4±0.1Pa mean SS @ 3Hz; 0.6±0.3Pa mean SS @ 5Hz; 1.2±0.4Pa mean SS @ 9Hz	Custom-made	Increase (RT-qPCR, PBGD)	1h @ 0.4Pa: 1.6 (rel.)† 1h @ 0.6Pa: 4.6 (rel.)† 1h @ 1.2Pa: 5.1 (rel.)†			
Bakker et al. (2004)	Bcl-2 / Bax	ratio (BCL2/BAX)	Human trabecular bone samples (60–84y female)/7F(healthy), n.g, dig., n.g., 5×10^5 cells per cm^2/n.g.)	Pulsatile laminar	1h @ (3Hz, 5Hz, 9Hz)	0.4±0.1Pa mean SS @ 3Hz; 0.6±0.3Pa mean SS @ 5Hz; 1.2±0.4Pa mean SS @ 9Hz	Custom-made	Increase (RT-qPCR, PBGD)	1h @ 0.4Pa: 1.2 (ratio)† 1h @ 0.6Pa: 3.6 (ratio)† 1h @ 1.2Pa: 4.7 (ratio)†			
McGarry et al. (2005)	Collagen type1	COL1A1	Human bone fragments ((14–16y males and 11y female)/2M (n.g) + 1F (n.g), n.g., exp., P (2+), 5×10^5 cells per cm^2 / n.g.)	Pulsatile laminar	1h @ 5Hz	0.6±0.3Pa	Custom-made			Increase (WB, n.g.)	1h+24h post-PFF: 0.6 (ratio)	

[a] Entry provided as reported in the given study.
[b] Human genes were confirmed with the HUGO Gene Nomenclature Committee (HGNC; URL: https://www.genenames.org); mouse genes were confirmed with the Mouse Genome Informatics (MGI; URL: https://www.informatics.jax.org/genes.shtml) after checking the specificity of primers with Primer-BLAST.
[c] Sex of donors: "M" – male, "F" – female; Tooth type: "PM" – premolar, "M" – molar; dig. Indicate isolation by cell digestion; Exp. indicate isolation by cell explant; Cell density: given in cells/cm^2 if not otherwise mentioned.
[d] Flow type deduced from the description of the FSS apparatus given by the authors.
f RT-qPCR (reverse-transcriptase quantitative polymerase chain reaction); sqPCR (semi-quantitative polymerase chain reaction); ELISA (enzyme-linked immuno absorbent assay); WB (western blotting); RIA (radioimmunoassay); EMSA (electromobility shift assay); IF (immunofluorescence)
rel.: indicate relative gene expression. Is entitled to percentages or gene expression ratios normalized to control, and not calculated by ΔΔCT.
FC: indicate fold change. When Author mentions the use of ΔΔCT or the method according to Livak & Schmittken (2004) in calculating FC.
n.g.: not given. For information not given by study-authors.
† Information derived from figures using Engauge Digitizer.
* Indicate manual calculations by measuring the graphs, without using the Engauge Digitizer.
ratio-calc: indicate manual calculation by dividing intervention/control = result (ratio-calc)
ratio: indicate ratios given by study-authors such as normalization to control in case of small molecules data or in case of gene expression ratios, e.g. ratio of RANKL/OPG or Bcl-2/Bax.

Reference	Gene or analyte[a]	Official gene symbol or abbreviation[b]	Cell Type ((age and sex)/number and sex of donor (health status), tooth type, isolation method, passages used, cell density / confluency)[a,c]	Flow type (steady laminar, pulsatile laminar, or oscillatory laminar)[a,d]	FSS-duration and frequency[a]	FSS magnitude[a]	FSS apparatus[a]	Gene expression: Increase, decrease, no change (method w/ reference gene); methods: RT-qPCR, sqPCR[f]	Gene expression: when it reaches peak and peak's magnitude (fold change; relative gene expression; times or ratio; unclear = ?)[f]	Protein expression: Increase, decrease, no change (method w/ reference); methods: ELISA, WB, RIA, EMSA, IF[f]	Protein expression: When it reaches peak and peak's magnitude (times or ratio; unclear = ?)[f]	Remarks
McGarry et al. (2005)	NO	Nitric oxide	Human bone fragments ((14–16y males and 11y female)/2M (n.g) + 1F (n.g), n.g., exp., P (2+), 5×10^5 cells per cm^2 / n.g.)	Pulsatile laminar	1h @ 5Hz	0.6±0.3Pa	Custom-made			Increase (Griess, NO2-)	1h: 7.1 (ratio)	
McGarry et al. (2005)	PGE2	PGE2	Human bone fragments ((14–16y males and 11y female)/2M (n.g) + 1F (n.g), n.g., exp., P (2+), 5×10^5 cells per cm^2 / n.g.)	Pulsatile laminar	1h @ 5Hz	0.6±0.3Pa	Custom-made			Increase (ELISA)	1h: 3.3 (ratio)	
Sterck et al. (1998)	NO	Nitric oxide	Human transiliac bone biopsies ((7–77y males and females)/10M (Healthy)+7F(Healthy), n.g., dig., n.g., 25×10^3 cells per cm^2/ n.g.)	Pulsatile laminar	1h (sampling 0h, 24h post-PFF) @ 5Hz	SS of 0.7±0.03Pa, peak stress rate of 12.2Pa/s	Custom-made			Increase (Griess, NO_2^-)	0h post-PFF: 2.3 (ratio)<br>24h post-PFF: 1.01 (ratio-calc)	
Sterck et al. (1998)	PGE2	PGE2	Human transiliac bone biopsies ((7–77y males and females)/10M (Healthy)+7F(Healthy), n.g., dig., n.g., 25×10^3 cells per cm^2/ n.g.)	Pulsatile laminar	1h (sampling 0h, 24h post-PFF) @ 5Hz	SS of 0.7±0.03Pa, peak stress rate of 12.2Pa/s	Custom-made			Increase (ELISA)	0h post-PFF: 1.8 (ratio)<br>24h post-PFF: 1.9 (ratio)	
Sterck et al. (1998)	TGF-Beta1	TGFB1	Human transiliac bone biopsies ((7–77y males and females)/10M (Healthy)+7F(Healthy), n.g., dig., n.g., 25×10^3 cells per cm^2/ n.g.)	Pulsatile laminar	1h (sampling 0h, 24h post-PFF) @ 5Hz	SS of 0.7±0.03Pa, peak stress rate of 12.2Pa/s	Custom-made			Increase (ELISA)	0h post-PFF: 0.7 (ratio)<br>24h post-PFF: 1.5 (ratio-calc)	
Rangaswami et al. (2009)	c-fos	FOS	Human trabecular bone ((n.g.)/ n.g.(n.g.), n.g,, dig., P5, 1.3×10^5 or 5×10^5 cells per cm^2/ ~40%)	Steady laminar	20min/n.g.	12dyn/cm^2	Cytodyne parallel flow chamber	Increase (sqPCR, GAPDH)	No quantitative information given.			
Rangaswami et al. (2009)	fosB, ΔfosB	FOSB	Human trabecular bone ((n.g.)/ n.g.(n.g.), n.g,, dig., P5, 1.3×10^5 or 5×10^5 cells per cm^2/ ~40%)	Steady laminar	20min/n.g.	12dyn/cm^2	Cytodyne parallel flow chamber	Increase (sqPCR, GAPDH)	No quantitative information given.			
Rangaswami et al. (2009)	fra-1	FOSL1	Human trabecular bone ((n.g.)/ n.g.(n.g.), n.g,, dig., P5, 1.3×10^5 or 5×10^5 cells per cm^2/ ~40%)	Steady laminar	20min/n.g.	12dyn/cm^2	Cytodyne parallel flow chamber	Increase (sqPCR, GAPDH)	No quantitative information given.			
Rangaswami et al. (2009)	fra-2	FOSL2	Human trabecular bone ((n.g.)/ n.g.(n.g.), n.g,, dig., P5, 1.3×10^5 or 5×10^5 cells per cm^2/ ~40%)	Steady laminar	20min/n.g.	12dyn/cm^2	Cytodyne parallel flow chamber	Increase (sqPCR, GAPDH)	No quantitative information given.			
Rangaswami et al. (2012)	FAK	PTK2	Human trabecular bone fragments ((n.g)/ n.g. (n.g.), n.g, exp., P1-3, 1.3×10^5 or 5×10^5 cells per cm^2/ ~40%)	Steady laminar	5min, 10min/n.g.	12dyn/cm^2	Cytodyne parallel flow chamber			5min: decrease (WB, n.g.)	No quantitative information given.	
Rangaswami et al. (2012)	p-GSK3B / GSK3B	GSK3B	Human trabecular bone fragments ((n.g)/ n.g. (n.g.), n.g, exp., P1-3, 1.3×10^5 or 5×10^5 cells per cm^2/ ~40%)	Steady laminar	5min, 10min/n.g.	12dyn/cm^2	Cytodyne parallel flow chamber			5min: increase (WB, GSK3B)	No quantitative information given.	
Rangaswami et al. (2012)	p(S^{473})-Akt / Akt	AKT1	Human trabecular bone fragments ((n.g)/ n.g. (n.g.), n.g, exp., P1-3, 1.3×10^5 or 5×10^5 cells per cm^2/ ~40%)	Steady laminar	5min, 10min/n.g.	12dyn/cm^2	Cytodyne parallel flow chamber			5min: increase (WB, AKT)	p-Akt/Akt @ 5min: 5.6 (ratio)†	
Rangaswami et al. (2012)	p(Y^{397})-FAK	PTK2	Human trabecular bone fragments ((n.g)/ n.g. (n.g.), n.g, exp., P1-3, 1.3×10^5 or 5×10^5 cells per cm^2/ ~40%)	Steady laminar	5min, 10min/n.g.	12dyn/cm^2	Cytodyne parallel flow chamber			5min: increase (WB, FAK)	No quantitative information given.	
Rangaswami et al. (2012)	p(Y^{418})-Src	SRC	Human trabecular bone fragments ((n.g)/ n.g. (n.g.), n.g, exp., P1-3, 1.3×10^5 or 5×10^5 cells per cm^2/ ~40%)	Steady laminar	5min, 10min/n.g.	12dyn/cm^2	Cytodyne parallel flow chamber			5min: increase (WB, Src)	No quantitative information given.	
Rangaswami et al. (2012)	p(Y^{576})-FAK	PTK2	Human trabecular bone fragments ((n.g)/ n.g. (n.g.), n.g, exp., P1-3, 1.3×10^5 or 5×10^5 cells per cm^2/ ~40%)	Steady laminar	5min, 10min/n.g.	12dyn/cm^2	Cytodyne parallel flow chamber			5min: increase (WB, FAK)	No quantitative information given.	
Rangaswami et al. (2012)	Src	SRC	Human trabecular bone fragments ((n.g)/ n.g. (n.g.), n.g, exp., P1-3, 1.3×10^5 or 5×10^5 cells per cm^2/ ~40%)	Steady laminar	5min, 10min/n.g.	12dyn/cm^2	Cytodyne parallel flow chamber			5min: decrease (WB, n.g.)	No quantitative information given.	

[a] Entry provided as reported in the given study.
[b] Human genes were confirmed with the HUGO Gene Nomenclature Committee (HGNC; URL: https://www.genenames.org); mouse genes were confirmed with the Mouse Genome Informatics (MGI; URL: https://www.informatics.jax.org/genes.shtml) after checking the specificity of primers with Primer-BLAST.
[c] Sex of donors: "M" – male, "F" – female; Tooth type: "PM" – premolar, "M" – molar; dig. Indicate isolation by cell digestion; Exp. indicate isolation by cell explant; Cell density: given in cells/cm^2 if not otherwise mentioned.
[d] Flow type deduced from the description of the FSS apparatus given by the authors.
f RT-qPCR (reverse-transcriptase quantitative polymerase chain reaction); sqPCR (semi-quantitative polymerase chain reaction); ELISA (enzyme-linked immuno absorbent assay); WB (western blotting); RIA (radioimmunoassay); EMSA (electromobility shift assay); IF (immunofluorescence)

rel.: indicate relative gene expression. Is entitled to percentages or gene expression ratios normalized to control, and not calculated by ΔΔCT.
FC: indicate fold change. When Author mentions the use of ΔΔCT or the method according to Livak & Schmittken (2004) in calculating FC.
n.g.: not given. For information not given by study-authors.
† Information derived from figures using Engauge Digitizer.
* Indicate manual calculations by measuring the graphs, without using the Engauge Digitizer.
ratio-calc: indicate manual calculation by dividing intervention/control = result (ratio-calc)
ratio: indicate ratios given by study-authors such as normalization to control in case of small molecules data or in case of gene expression ratios, e.g. ratio of RANKL/OPG or Bcl-2/Bax.

Reference	Gene or analyte[a]	Official gene symbol or abbreviation[b]	Cell Type ((age and sex)/number and sex of donor (health status), tooth type, isolation method, passages used, cell density / confluency)[a,c]	Flow type (steady laminar, pulsatile laminar, or oscillatory laminar)[a,d]	FSS-duration and frequency[a]	FSS magnitude[a]	FSS apparatus[a]	Gene expression: Increase, decrease, no change (method w/ reference gene); methods: RT-qPCR, sqPCR[f]	Gene expression: when it reaches peak and peak's magnitude (fold change; relative gene expression; times or ratio; unclear = ?)[f]	Protein expression: Increase, decrease, no change (method w/ reference); methods: ELISA, WB, RIA, EMSA, IF[f]	Protein expression: When it reaches peak and peak's magnitude (times or ratio; unclear = ?)[f]	Remarks
van der Meijden et al. (2016)	CYP24	CYP24A1	Human trabecular bone (maxilla/mandible) samples ((35.1± 5.7y males and females)/2M(healthy), 9F(Healthy), n.g., exp., n.g., 5×10^5 cells per cm^2/ n.g.)	Pulsatile laminar	1h (sampling 0h, 3h post-PFF) / 5Hz	0.7Pa	Custom-made	Increase (RT-qPCR, TBP)	3h post-PFF: 4.3 (FC)†			
van der Meijden et al. (2016)	CYP27B1	CYP27B1	Human trabecular bone (maxilla/mandible) samples ((35.1± 5.7y males and females)/2M(healthy), 9F(Healthy), n.g., exp., n.g., 5×10^5 cells per cm^2/ n.g.)	Pulsatile laminar	1h (sampling 0h, 3h post-PFF) / 5Hz	0.7Pa	Custom-made	Decrease then increase (RT-qPCR, TBP)	0h post-PFF: 0.9 (FC)†<br>3h post-PFF: 2.1 (FC)†			
van der Meijden et al. (2016)	NO	Nitric oxide	Human trabecular bone (maxilla/mandible) samples ((35.1± 5.7y males and females)/2M(healthy), 9F(Healthy), n.g., exp., n.g., 5×10^5 cells per cm^2/ n.g.)	Pulsatile laminar	1h (sampling 0h, 3h post-PFF) / 5Hz	0.7Pa	Custom-made			Increase (Griess, NO_2^-)	0h post-PFF: 34.3nmol; 34.3/2.3 = 14.9 (ratio-calc)†	
van der Meijden et al. (2016)	OPG	TNFRSF11B	Human trabecular bone (maxilla/mandible) samples ((35.1± 5.7y males and females)/2M(healthy), 9F(Healthy), n.g., exp., n.g., 5×10^5 cells per cm^2/ n.g.)	Pulsatile laminar	1h (sampling 0h, 3h post-PFF) / 5Hz	0.7Pa	Custom-made	Increase (RT-qPCR, TBP)	3h post-PFF: 1.2 (FC)†			
van der Meijden et al. (2016)	Osteocalcin	BGLAP	Human trabecular bone (maxilla/mandible) samples ((35.1± 5.7y males and females)/2M(healthy), 9F(Healthy), n.g., exp., n.g., 5×10^5 cells per cm^2/ n.g.)	Pulsatile laminar	1h (sampling 0h, 3h post-PFF) / 5Hz	0.7Pa	Custom-made	Decrease (RT-qPCR, TBP)	3h post-PFF: 0.9 (FC)†			
van der Meijden et al. (2016)	Osteopontin	SPP1	Human trabecular bone (maxilla/mandible) samples ((35.1± 5.7y males and females)/2M(healthy), 9F(Healthy), n.g., exp., n.g., 5×10^5 cells per cm^2/ n.g.)	Pulsatile laminar	1h (sampling 0h, 3h post-PFF) / 5Hz	0.7Pa	Custom-made	Decrease (RT-qPCR, TBP)	3h post-PFF: 0.9 (FC)†			
van der Meijden et al. (2016)	RANKL	TNFSF11	Human trabecular bone (maxilla/mandible) samples ((35.1± 5.7y males and females)/2M(healthy), 9F(Healthy), n.g., exp., n.g., 5×10^5 cells per cm^2/ n.g.)	Pulsatile laminar	1h (sampling 0h, 3h post-PFF) / 5Hz	0.7Pa	Custom-made	Increase (RT-qPCR, TBP)	3h post-PFF: 4.3 (FC)†			
van der Meijden et al. (2016)	RANKL / OPG	ratio (RANKL/OPG)	Human trabecular bone (maxilla/mandible) samples ((35.1± 5.7y males and females)/2M(healthy), 9F(Healthy), n.g., exp., n.g., 5×10^5 cells per cm^2/ n.g.)	Pulsatile laminar	1h (sampling 0h, 3h post-PFF) / 5Hz	0.7Pa	Custom-made	Increase (RT-qPCR, TBP)	3h post-PFF: 7.5 (ratio)†			
van der Meijden et al. (2016)	VDR	VDR	Human trabecular bone (maxilla/mandible) samples ((35.1± 5.7y males and females)/2M(healthy), 9F(Healthy), n.g., exp., n.g., 5×10^5 cells per cm^2/ n.g.)	Pulsatile laminar	1h (sampling 0h, 3h post-PFF) / 5Hz	0.7Pa	Custom-made	Increase then decrease (RT-qPCR, TBP)	0h post-PFF: 1.1 (FC)†<br>3h post-PFF: 0.6 (FC)†			
Santos et al. (2011)	BMP2	BMP2	Human trabecular bone samples ((18-84y males and females)/2M(n.g.) and 7F(n.g.), n.g, dig., P2, 5×10^3 cells per cm^2/ n.g.)	Pulsatile laminar	1h (sampling 1-24h post-PFF) / n.g.	0.7±0.3Pa	Custom-made	Increase then decrease with plateau (RT-qPCR, GAPDH)	0h post-PFF: 1.3 (rel.)†<br>1h post-PFF: 0.55 (rel.)†<br>3h post-PFF: 0.56(rel.)†	Increase (ELISA)	24h post-PFF: 169.2 pM; 169.2/145 = 1.2 (ratio-calc)†	
Santos et al. (2011)	BMP7	BMP7	Human trabecular bone samples ((18-84y males and females)/2M(n.g.) and 7F(n.g.), n.g, dig., P2, 5×10^3 cells per cm^2/ n.g.)	Pulsatile laminar	1h (sampling 1-24h post-PFF) / n.g.	0.7±0.3Pa	Custom-made	Increase (RT-qPCR, GAPDH)	3h post-PFF: 5.6 (rel.)†	Increase with plateau (ELISA)	6h post-PFF: 31.9pM; 31.9/13.6 = 2.3 (ratio-calc)†<br>24h post-PFF: 30pM; 30/12 = 2.5 (ratio-calc)†	
Santos et al. (2011)	NO	Nitric oxide	Human trabecular bone samples ((18-84y males and females)/2M(n.g.) and 7F(n.g.), n.g, dig., P2, 5×10^3 cells per cm^2/ n.g.)	Pulsatile laminar	5min / n.g.	0.7±0.3Pa	Custom-made			Increase (Griess, NO_2^-)	16.8 nmol; 16.8/5.1 = 3.2 (ratio-calc)†	

[a] Entry provided as reported in the given study.
[b] Human genes were confirmed with the HUGO Gene Nomenclature Committee (HGNC; URL: https://www.genenames.org); mouse genes were confirmed with the Mouse Genome Informatics (MGI; URL: https://www.informatics.jax.org/genes.shtml) after checking the specificity of primers with Primer-BLAST.
[c] Sex of donors: "M" – male, "F" – female; Tooth type: "PM" – premolar, "M" – molar; dig. Indicate isolation by cell digestion; Exp. indicate isolation by cell explant; Cell density: given in cells/cm^2 if not otherwise mentioned.
[d] Flow type deduced from the description of the FSS apparatus given by the authors.
f RT-qPCR (reverse-transcriptase quantitative polymerase chain reaction); sqPCR (semi-quantitative polymerase chain reaction); ELISA (enzyme-linked immuno absorbent assay); WB (western blotting); RIA (radioimmunoassay); EMSA (electromobility shift assay); IF (immunofluorescence)

rel.: indicate relative gene expression. Is entitled to percentages or gene expression ratios normalized to control, and not calculated by ΔΔCT.
FC: indicate fold change. When Author mentions the use of ΔΔCT or the method according to Livak & Schmittken (2004) in calculating FC.
n.g.: not given. For information not given by study-authors.
† Information derived from figures using Engauge Digitizer.
* Indicate manual calculations by measuring the graphs, without using the Engauge Digitizer.
ratio-calc: indicate manual calculation by dividing intervention/control = result (ratio-calc)
ratio: indicate ratios given by study-authors such as normalization to control in case of small molecules data or in case of gene expression ratios, e.g. ratio of RANKL/OPG or Bcl-2/Bax.

Reference	Gene or analyte[a]	Official gene symbol or abbreviation[b]	Cell Type ((age and sex)/number and sex of donor (health status), tooth type, isolation method, passages used, cell density / confluency)[a,c]	Flow type (steady laminar, pulsatile laminar, or oscillatory laminar)[a,d]	FSS-duration and frequency[a]	FSS magnitude[a]	FSS apparatus[a]	Gene expression: Increase, decrease, no change (method w/ reference gene); methods: RT-qPCR, sqPCR[f]	Gene expression: when it reaches peak and peak's magnitude (fold change; relative gene expression; times or ratio; unclear = ?)[f]	Protein expression: Increase, decrease, no change (method w/ reference); methods: ELISA, WB, RIA, EMSA, IF[f]	Protein expression: When it reaches peak and peak's magnitude (times or ratio; unclear = ?)[f]	Remarks
Aisha et al. (2015)	Alkaline phosphatase	ALP (unspecific)	NHOst cells ((n.g.) /n.g. (n.g.), n.g., n.g., n.g., 2.5×10^5 cells per cm^2/ n.g.)	Oscillatory laminar	72h / n.g.	250 RPM	Orbital Shaker			Increase (activity)	72h: 0.0034U/mL/µmol; 0.0034/0.0022 = 1.55 (ratio-calc)†	ALP activity determined using p-nitrophenol substrate.
Aisha et al. (2015)	OCN	BGLAP	NHOst cells ((n.g.) /n.g. (n.g.), n.g., n.g., n.g., 2.5×10^5 cells per cm^2/ n.g.)	Oscillatory laminar	72h / n.g.	250 RPM	Orbital Shaker			Decrease (ELISA)	72h: 4.2ng/mL; 4.2/4.4 = 0.95 (ratio-calc)†	
Aisha et al. (2015)	OPG	TNFRSF11B	NHOst cells ((n.g.) /n.g. (n.g.), n.g., n.g., n.g., 2.5×10^5 cells per cm^2/ n.g.)	Oscillatory laminar	72h / n.g.	250 RPM	Orbital Shaker			Increase (ELISA)	72h: 803.3pg/mL; 803.29/151.3 = 5.31 (ratio-calc)†	
Aisha et al. (2015)	RANKL	TNFSF11	NHOst cells ((n.g.) /n.g. (n.g.), n.g., n.g., n.g., 2.5×10^5 cells per cm^2/ n.g.)	Oscillatory laminar	72h / n.g.	250 RPM	Orbital Shaker			Decrease (ELISA)	72h: 135.6pg/mL; 135.6/364.6 = 0.37 (ratio-calc)†	
Aisha et al. (2015)	RANKL / OPG	ratio (RANKL/OPG)	NHOst cells ((n.g.) /n.g. (n.g.), n.g., n.g., n.g., 2.5×10^5 cells per cm^2/ n.g.)	Oscillatory laminar	72h / n.g.	250 RPM	Orbital Shaker			Decrease (ELISA)	72h: 0.16 (ratio)†	
Klein-Nulend et al. (1998)	ecNOS (endothelial NOS)	NOS3	Human Transiliac bone biopsies ((7-90y, n.g.)/18, n.g. (healthy), n.g., dig., n.g., 5×10^5 cells per cm^2/ n.g.)	Pulsatile laminar	1h (sampling 0h, 1h, 24h post-PFF) @ 5Hz	0.7±0.03Pa (12.2Pa/s)	Custom-made	Increase (sqPCR, β-actin)	0h post-PFF: 142.6% (rel.)† 1h post-PFF: 180.5% (rel.)†			Figure 5b
Klein-Nulend et al. (1998)	NO	Nitric oxide	Human Transiliac bone biopsies ((7-90y, n.g.)/18, n.g. (healthy), n.g., dig., n.g., 5×10^5 cells per cm^2/ n.g.)	Pulsatile laminar	0min, 4min, 8min, 15min, 30min, 60min @ 5Hz	0.7±0.03Pa (12.2Pa/s)	Custom-made			Increase (Griess, NO_2^-)	30min: 423.8nmol/mg protein; 423.8/63.8 = 6.6 (ratio-calc)†	
Klein-Nulend et al. (1998)	NO	Nitric oxide	Human Transiliac bone biopsies ((7-90y, n.g.)/18, n.g. (healthy), n.g., dig., n.g., 5×10^5 cells per cm^2/ n.g.)	Pulsatile laminar	1h (sampling 0h, 1h, 24h post-PFF) @ 5Hz	0.7±0.03Pa (12.2Pa/s)	Custom-made			Increase (Griess, NO_2^-)	0h post-PFF: 734.8nmol/mg protein; 734.8/323.1 = 2.3 (ratio-calc)† 1h post-PFF: 70.9nmol/mg protein; 70.9/62.4 = 1.14 (ratio-calc)† 24h post-PFF: 358.6nmol/mg protein; 358.6/319.9 = 1.12 (ratio-calc)†	
Bakker et al. (2003)	Cox1	PTGS1	Primary human bone cells ((54–84y females)/ 9F (healthy), n.g., dig., n.g., 5×10^5 cells per slide / n.g.)	Pulsatile laminar	1h (sampling 0h, 24h post-PFF) @ 5Hz	0.6±0.3Pa mean SS	Custom-made	Mean increase (sqPCR, GAPDH)	0h post-PFF: 1.3 (rel.)†			
Bakker et al. (2003)	COX2	PTGS2	Primary human bone cells ((54–84y females)/ 9F (healthy), n.g., dig., n.g., 5×10^5 cells per slide / n.g.)	Pulsatile laminar	1h (sampling 0h, 24h post-PFF) @ 5Hz	0.6±0.3Pa mean SS	Custom-made	Mean increase (RT-qPCR, PBGD)	0h post-PFF: 3.8 (rel.)† 24h post-PFF: 4.97 (rel.)†			
Bakker et al. (2003)	PGE2	PGE2	Primary human bone cells ((54–84y females)/ 9F (healthy), n.g., dig., n.g., 5×10^5 cells per slide / n.g.)	Pulsatile laminar	1h (sampling 0h, 24h post-PFF) @ 5Hz	0.6±0.3Pa mean SS	Custom-made			Increase (ELISA)	0h post-PFF: 2.3 ng/5×10^5 cells; 2.3/1.2 = 2.76 (ratio-calc)† 24h post-PFF: 0.96 ng/5×10^5 cells; 0.96/0.6 = 1.6 (ratio-calc)†	
Joldersma et al. (2000)	COX-1	PTGS1	Human transiliac bone biopsies ((56-80y females)/9F(Healthy), n.g., dig., n.g., 25×10^3 cells per cm^2/ n.g.)	Pulsatile laminar	1h @ 5Hz	FSS of 0.7±0.02Pa, peak stress rate of 12.2Pa/s	Custom-made	Increase (sqPCR, GAPDH)	1.3 (rel.)†			Data of 9 individuals also reported.
Joldersma et al. (2000)	COX-2	PTGS2	Human transiliac bone biopsies ((56-80y females)/9F(Healthy), n.g., dig., n.g., 25×10^3 cells per cm^2/ n.g.)	Pulsatile laminar	1h @ 5Hz	FSS of 0.7±0.02Pa, peak stress rate of 12.2Pa/s	Custom-made	Increase (sqPCR, GAPDH)	2.9 (rel.)†			Data of 9 individuals also reported.
Joldersma et al. (2000)	PGE_2	PGE2	Human transiliac bone biopsies ((56-80y females)/9F(Healthy), n.g., dig., n.g., 25×10^3 cells per cm^2/ n.g.)	Pulsatile laminar	1h @ 5Hz	FSS of 0.7±0.02Pa, peak stress rate of 12.2Pa/s	Custom-made			Mean increase (EIA)	3897.4 pg/µgDNA; 3897.4/1107.7 = 3.52 (ratio-calc)†	Data of 9 individuals also reported.
Joldersma et al. (2000)	$PGF_{2\alpha}$	PGF2alpha	Human transiliac bone biopsies ((56-80y females)/9F(Healthy), n.g., dig., n.g., 25×10^3 cells per cm^2/ n.g.)	Pulsatile laminar	1h @ 5Hz	FSS of 0.7±0.02Pa, peak stress rate of 12.2Pa/s	Custom-made			Mean increase (RIA)	532.5pg/µgDNA; 532.5/245 = 2.17 (ratio-calc)†	Data of 9 individuals also reported.
Joldersma et al. (2000)	PGI_2	PGI2	Human transiliac bone biopsies ((56-80y females)/9F(Healthy), n.g., dig., n.g., 25×10^3 cells per cm^2/ n.g.)	Pulsatile laminar	1h @ 5Hz	FSS of 0.7±0.02Pa, peak stress rate of 12.2Pa/s	Custom-made			Mean increase (EIA)	1110.2pg/µgDNA; 1110.2/302 = 3.68 (ratio-calc)†	Data of 9 individuals also reported.

[a] Entry provided as reported in the given study.
[b] Human genes were confirmed with the HUGO Gene Nomenclature Committee (HGNC; URL: https://www.genenames.org); mouse genes were confirmed with the Mouse Genome Informatics (MGI; URL: https://www.informatics.jax.org/genes.shtml) after checking the specificity of primers with Primer-BLAST.
[c] Sex of donors: "M" – male, "F" – female; Tooth type: "PM" – premolar, "M" – molar; dig. Indicate isolation by cell digestion; Exp. indicate isolation by cell explant; Cell density: given in cells/cm^2 if not otherwise mentioned.
[d] Flow type deduced from the description of the FSS apparatus given by the authors.
f RT-qPCR (reverse-transcriptase quantitative polymerase chain reaction); sqPCR (semi-quantitative polymerase chain reaction); ELISA (enzyme-linked immuno absorbent assay); WB (western blotting); RIA (radioimmunoassay); EMSA (electromobility shift assay); IF (immunofluorescence)

rel.: indicate relative gene expression. Is entitled to percentages or gene expression ratios normalized to control, and not calculated by ΔΔCT.
FC: indicate fold change. When Author mentions the use of ΔΔCT or the method according to Livak & Schmittken (2004) in calculating FC.
n.g.: not given. For information not given by study-authors.
† Information derived from figures using Engauge Digitizer.
* Indicate manual calculations by measuring the graphs, without using the Engauge Digitizer.
ratio-calc: indicate manual calculation by dividing intervention/control = result (ratio-calc)
ratio: indicate ratios given by study-authors such as normalization to control in case of small molecules data or in case of gene expression ratios, e.g. ratio of RANKL/OPG or Bcl-2/Bax.

Reference	Gene or analyte[a]	Official gene symbol or abbreviation[b]	Cell Type ((age and sex)/number and sex of donor (health status), tooth type, isolation method, passages used, cell density / confluency)[a,c]	Flow type (steady laminar, pulsatile laminar, or oscillatory laminar)[a,d]	FSS-duration and frequency[a]	FSS magnitude[a]	FSS apparatus[a]	Gene expression: Increase, decrease, no change (method w/ reference gene); methods: RT-qPCR, sqPCR[f]	Gene expression: when it reaches peak and peak's magnitude (fold change; relative gene expression; times or ratio; unclear = ?)[f]	Protein expression: Increase, decrease, no change (method w/ reference); methods: ELISA, WB, RIA, EMSA, IF[f]	Protein expression: When it reaches peak and peak's magnitude (times or ratio; unclear = ?)[f]	Remarks
Ehnert et al. (2017)	ALP	ALP (unspecific)	Human high tibial osteotomy/phOBs cells ((65.4±12.0y males and females)/11M (n.g) and 21F (n.g), n.g., dig., P3-4, 15×10³ cells per cm²/ n.g.)	(Oscillatory laminar)	7d with 2h/d / n.g.	rocking speed: 25rpm or 50rpm	A rocking shaker			Mean increase (activity)	2h/d for 7d @ 25rpm: 134.7% (rel.)	ALP activity determined using p-nitrophenol substrate.

References

Aisha MD, Nor-Ashikin MN, Sharaniza AB, Nawawi H, Froemming GR (2015). Orbital fluid shear stress promotes osteoblast metabolism, proliferation and alkaline phosphates activity in vitro. Exp Cell Res; 337(1):87-93.

Bakker A, Klein-Nulend J, Burger E (2004). Shear stress inhibits while disuse promotes osteocyte apoptosis. Biochem Biophys Res Commun; 320(4):1163-8.

Bakker AD, Klein-Nulend J, Burger EH (2003). Mechanotransduction in bone cells proceeds via activation of COX-2, but not COX-1. Biochem Biophys Res Commun; 305(3):677-83.

Ehnert S, Sreekumar V, Aspera-Werz RH, Sajadian SO, Wintermeyer E, Sandmann GH, Bahrs C, Hengstler JG, Godoy P, Nussler AK (2017). TGF-β(1) impairs mechanosensation of human osteoblasts via HDAC6-mediated shortening and distortion of primary cilia. J Mol Med (Berl); 95(6):653-663.

Joldersma M, Burger EH, Semeins CM, Klein-Nulend J (2000). Mechanical stress induces COX-2 mRNA expression in bone cells from elderly women. J Biomech; 33(1):53-61.

Joldersma M, Klein-Nulend J, Oleksik AM, Heyligers IC, Burger EH (2001). Estrogen enhances mechanical stress-induced prostaglandin production by bone cells from elderly women. Am J Physiol Endocrinol Metab; 280(3):E436-42.

Klein-Nulend J, Helfrich MH, Sterck JG, MacPherson H, Joldersma M, Ralston SH, Semeins CM, Burger EH (1998). Nitric oxide response to shear stress by human bone cell cultures is endothelial nitric oxide synthase dependent. Biochem Biophys Res Commun; 250(1):108-14.

Klein-Nulend J, Sterck JG, Semeins CM, Lips P, Joldersma M, Baart JA, Burger EH (2002). Donor age and mechanosensitivity of human bone cells. Osteoporos Int; 13(2):137-46.

McGarry JG, Klein-Nulend J, Mullender MG, Prendergast PJ (2005). A comparison of strain and fluid shear stress in stimulating bone cell responses--a computational and experimental study. FASEB J; 19(3):482-4.

Rangaswami H, Marathe N, Zhuang S, Chen Y, Yeh JC, Frangos JA, Boss GR, Pilz RB (2009). Type II cGMP-dependent protein kinase mediates osteoblast mechanotransduction. J Biol Chem; 284(22):14796-808.

Rangaswami H, Schwappacher R, Tran T, Chan GC, Zhuang S, Boss GR, Pilz RB (2012). Protein kinase G and focal adhesion kinase converge on Src/Akt/β-catenin signaling module in osteoblast mechanotransduction. J Biol Chem; 287(25):21509-19.

Santos A, Bakker AD, Willems HM, Bravenboer N, Bronckers AL, Klein-Nulend J (2011). Mechanical loading stimulates BMP7, but not BMP2, production by osteocytes. Calcif Tissue Int; 89(4):318-26.

Sterck JG, Klein-Nulend J, Lips P, Burger EH (1998). Response of normal and osteoporotic human bone cells to mechanical stress in vitro. Am J Physiol; 274(6):E1113-20.

van der Meijden K, Bakker AD, van Essen HW, Heijboer AC, Schulten EA, Lips P, Bravenboer N (2016). Mechanical loading and the synthesis of 1,25(OH)2D in primary human osteoblasts. J Steroid Biochem Mol Biol; 156:32-9.

[a] Entry provided as reported in the given study.
[b] Human genes were confirmed with the HUGO Gene Nomenclature Committee (HGNC; URL: https://www.genenames.org); mouse genes were confirmed with the Mouse Genome Informatics (MGI; URL: https://www.informatics.jax.org/genes.shtml) after checking the specificity of primers with Primer-BLAST.
[c] Sex of donors: "M" – male, "F" – female; Tooth type: "PM" – premolar, "M" – molar; dig. Indicate isolation by cell digestion; Exp. indicate isolation by cell explant; Cell density: given in cells/cm² if not otherwise mentioned.
[d] Flow type deduced from the description of the FSS apparatus given by the authors.
f RT-qPCR (reverse-transcriptase quantitative polymerase chain reaction); sqPCR (semi-quantitative polymerase chain reaction); ELISA (enzyme-linked immuno absorbent assay); WB (western blotting); RIA (radioimmunoassay); EMSA (electromobility shift assay); IF (immunofluorescence)

rel.: indicate relative gene expression. Is entitled to percentages or gene expression ratios normalized to control, and not calculated by ΔΔCT.
FC: indicate fold change. When Author mentions the use of ΔΔCT or the method according to Livak & Schmittken (2004) in calculating FC.
n.g.: not given. For information not given by study-authors.
† Information derived from figures using Engauge Digitizer.
* Indicate manual calculations by measuring the graphs, without using the Engauge Digitizer.
ratio-calc: indicate manual calculation by dividing intervention/control = result (ratio-calc)
ratio: indicate ratios given by study-authors such as normalization to control in case of small molecules data or in case of gene expression ratios, e.g. ratio of RANKL/OPG or Bcl-2/Bax.

2.3 Human osteocytes

Reference	Gene or analyte[a]	Official gene symbol or abbreviation[b]	Cell type (age/number and sex of donor (health status), tooth type, isolation method, passages used, cell density/confluency)[a,c]	Flow type (steady laminar, pulsatile laminar, or oscillatory laminar)[a,d]	FSS-duration and frequency[a]	FSS-magnitude[a]	FSS-apparatus[a]	Gene expression: Increase, decrease, no change (method w/ reference gene); Methods: (RT-qPCR, sqPCR)[f]	Gene expression: when it reaches peak and peak's magnitude (fold change; relative gene expression; times or ratio; unclear = ?)[f]	Protein expression: Increase, decrease, no change (method w/reference); Methods: ELISA, WB, RIA, EMSA, IF[f]	Protein expression: When it reaches peak and peak's magnitude (times or ratio; unclear = ?)[f]	Remarks
Pathak et al. (2015)	COX2	PTGS2	Trabecular bone samples (human Primary bone osteocytes) (38y (male), 73y (male) and 55y (Female)/ 2M and 1F (healthy), n.g., dig., P1-3, 5×10⁴ cells/ n.g.)	Pulsating laminar	60min (sampling 1h post-PFF) / 5Hz	Mean ± amplitude: 0.7±0.7Pa	Custom-made	Increase (RT-qPCR, YWHAZ)	1h post-PFF: 1.9/0.28 = 6.78 (ratio-calc)†			
Pathak et al. (2015)	CYR61	CCN1	Trabecular bone samples (human Primary bone osteocytes) (38y (male), 73y (male) and 55y (Female)/ 2M and 1F (healthy), n.g., dig., P1-3, 5×10⁴ cells/ n.g.)	Pulsating laminar	60min (sampling 1h post-PFF) / 5Hz	Mean ± amplitude: 0.7±0.7Pa	Custom-made	Increase (RT-qPCR, YWHAZ)	1h post-PFF: 0.13/0.05 = 2.6 (ratio-calc)†			
Pathak et al. (2015)	IL-6	IL6	Trabecular bone samples (human Primary bone osteocytes) (38y (male), 73y (male) and 55y (Female)/ 2M and 1F (healthy), n.g., dig., P1-3, 5×10⁴ cells/ n.g.)	Pulsating laminar	60min (sampling 1h post-PFF) / 5Hz	Mean ± amplitude: 0.7±0.7Pa	Custom-made	Increase (RT-qPCR, YWHAZ)	1h post-PFF: 0.79/0.07 = 11.3 (ratio-calc)†			
Pathak et al. (2015)	MEPE	MEPE	Trabecular bone samples (human Primary bone osteocytes) (38y (male), 73y (male) and 55y (Female)/ 2M and 1F (healthy), n.g., dig., P1-3, 5×10⁴ cells/ n.g.)	Pulsating laminar	60min (sampling 1h post-PFF) / 5Hz	Mean ± amplitude: 0.7±0.7Pa	Custom-made	Increase (RT-qPCR, YWHAZ)	1h post-PFF: 1.3/0.93 = 1.4 (ratio-calc)†			
Pathak et al. (2015)	NO	Nitric oxide	Trabecular bone samples (human Primary bone osteocytes) (38y (male), 73y (male) and 55y (Female)/ 2M and 1F (healthy), n.g., dig., P1-3, 5×10⁴ cells/ n.g.)	Pulsating laminar	5min, 60min / 5Hz	Mean ± amplitude: 0.7±0.7Pa	Custom-made			Increase (Griess, NO_2^-)	5min: 3.2 (nmol/5×10⁴); 3.2/1.2 = 2.67 (ratio-calc)† 60min: 6.2 (nmol/5×10⁴); 6.2/1.7 = 3.6 (ratio-calc)†	
Pathak et al. (2015)	OPG	TNFRSF11B	Trabecular bone samples (human Primary bone osteocytes) (38y (male), 73y (male) and 55y (Female)/ 2M and 1F (healthy), n.g., dig., P1-3, 5×10⁴ cells/ n.g.)	Pulsating laminar	60min (sampling 1h post-PFF) / 5Hz	Mean ± amplitude: 0.7±0.7Pa	Custom-made	Increase (RT-qPCR, YWHAZ)	1h post-PFF: 1.7/1.12 = 1.5 (ratio-calc)†			
Pathak et al. (2015)	PGE2	PGE2	Trabecular bone samples (human Primary bone osteocytes) (38y (male), 73y (male) and 55y (Female)/ 2M and 1F (healthy), n.g., dig., P1-3, 5×10⁴ cells/ n.g.)	Pulsating laminar	5min, 60min / 5Hz	Mean ± amplitude: 0.7±0.7Pa	Custom-made			5min: Decrease (ELISA) 60min: Increase (ELISA)	5min: 183 (pg/5×10⁴); 183/254.7 = 0.7 (ratio-calc)† 60min: 473.4 (pg/5×10⁴); 473.4/189.2 = 2.5 (ratio-calc)†	
Pathak et al. (2015)	RANKL	TNFSF11	Trabecular bone samples (human Primary bone osteocytes) (38y (male), 73y (male) and 55y (Female)/ 2M and 1F (healthy), n.g., dig., P1-3, 5×10⁴ cells/ n.g.)	Pulsating laminar	60min (sampling 1h post-PFF) / 5Hz	Mean ± amplitude: 0.7±0.7Pa	Custom-made	Increase (RT-qPCR, YWHAZ)	1h post-PFF: 1.0/0.54 = 1.9 (ratio-calc)†			
Pathak et al. (2015)	RANKL / OPG	ratio (RANKL/OPG)	Trabecular bone samples (human Primary bone osteocytes) (38y (male), 73y (male) and 55y (Female)/ 2M and 1F (healthy), n.g., dig., P1-3, 5×10⁴ cells/ n.g.)	Pulsating laminar	60min (sampling 1h post-PFF) / 5Hz	Mean ± amplitude: 0.7±0.7Pa	Custom-made	Increase (RT-qPCR, YWHAZ)	1h post-PFF: 0.76/0.58 = 1.3 (ratio-calc)†			
Pathak et al. (2015)	SOST	SOST	Trabecular bone samples (Human Primary bone osteocytes) (38y (male), 73y (male) and 55y (Female)/ 2M and 1F (healthy), n.g., dig., P1-3, 5×10⁴ cells/n.g.)	Pulsating laminar	60min (sampling 1h post-PFF) / 5Hz	Mean ± amplitude: 0.7±0.7Pa	Custom-made	Increase (RT-qPCR, YWHAZ)	1h post-PFF: 0.04/0.04 = 1 (ratio-calc)†			

Reference

Pathak JL, Bravenboer N, Luyten FP, Verschueren P, Lems WF, Klein-Nulend J, Bakker AD (2015). Mechanical loading reduces inflammation-induced human osteocyte-to-osteoclast communication. Calcif Tissue Int; 97(2):169-78.

[a] Entry provided as reported in the given study.
[b] Human genes were confirmed with the HUGO Gene Nomenclature Committee (HGNC; URL: https://www.genenames.org); mouse genes were confirmed with the Mouse Genome Informatics (MGI; URL: https://www.informatics.jax.org/genes.shtml) after checking the specificity of primers with Primer-BLAST.
[c] Sex of donors: "M" – male, "F" – female; Tooth type: "PM" – premolar, "M" – molar; dig. Indicate isolation by cell digestion; Exp. indicate isolation by cell explant; Cell density: given in cells/cm² if not otherwise mentioned.
[d] Flow type deduced from the description of the FSS apparatus given by the authors.
[f] RT-qPCR (reverse-transcriptase quantitative polymerase chain reaction); sqPCR (semi-quantitative polymerase chain reaction); ELISA (enzyme-linked immuno absorbent assay); WB (western blotting); RIA (radioimmunoassay); EMSA (electromobility shift assay); IF (immunofluorescence)

rel.: indicate relative gene expression. Is entitled to percentages or gene expression ratios normalized to control, and not calculated by ΔΔCT.
FC: indicate fold change. When Author mentions the use of ΔΔCT or the method according to Livak & Schmittken (2004) in calculating FC.
n.g.: not given. For information not given by study-authors.
† Information derived from figures using Engauge Digitizer.
* Indicate manual calculations by measuring the graphs, without using the Engauge Digitizer.
ratio-calc: indicate manual calculation by dividing intervention/control = result (ratio-calc)
ratio: indicate ratios given by study-authors such as normalization to control in case of small molecules data or in case of gene expression ratios, e.g. ratio of RANKL/OPG or Bcl-2/Bax.

2.4 Human periodontal ligament cells

Reference	Gene or analyte[a]	Official gene symbol or abbreviation[b]	Cell Type (age/sex of donor, health status, tooth type, isolation method, passages used, cell density/confluency)[a,c]	Flow type (steady laminar, pulsatile laminar, or oscillatory laminar)[a,d]	FSS duration and frequency[a]	FSS magnitude[a]	FSS-apparatus[a]	Gene expression: Increase, decrease, no change (method w/ reference gene); Methods: (RT-qPCR, sqPCR)[f]	Gene expression: when it reaches peak and peak's magnitude (fold change; relative gene expression; times or ratio; unclear = ?)[f]	Protein expression: Increase, decrease, no change (method w/reference); Methods: ELISA, WB, RIA, EMSA, IF[f]	Protein expression: When it reaches peak and peak's magnitude (times or ratio; unclear = ?)[f]	Remarks
Zheng et al. (2016)	hALP	ALPL	hPDLCs (12–28y/n.g., Healthy, M, dig., P3–6, n.g./ 90%)	Steady laminar	2h, 4h, 8h, 12h/n.g.	6dyn/cm²	Custom-made	Increase (sqPCR, GAPDH)	8h: 1.6/0.56 = 2.9 (ratio-calc)†			Verified by Primer-BLAST.
Zheng et al. (2016)	hBMP-2	BMP2	hPDLCs (12–28y/n.g., Healthy, M, dig., P3–6, n.g./ 90%)	Steady laminar	2h, 4h, 8h, 12h/n.g.	6dyn/cm²	Custom-made	Increase (sqPCR, GAPDH)	8h: 1.9/0.05 = 38 (ratio-calc)†			
Zheng et al. (2016)	hFGF-2	FGF2	hPDLCs (12–28y/n.g., Healthy, M, dig., P3–6, n.g./ 90%)	Steady laminar	2h, 4h, 8h, 12h/n.g.	6dyn/cm²	Custom-made	Increase followed by plateau (sqPCR, GAPDH)	2h: 0.86/0.12 = 7.2 (ratio-calc)†<br>4h: 0.84/0.12 = 7 (ratio-calc)†<br>8h: 0.75/0.12 = 6.25 (ratio-calc)†	Increase with plateau followed by decrease (ELISA)	2h: 89ng/mL; 89/46.2 = 1.9 (ratio-calc)†<br>4h: 92.4ng/mL; 92.4/46.2 = 2 (ratio-calc)†<br>12h: 5.3ng/mL; 5.3/46.2 = 0.11 (ratio-calc)†	
Zheng et al. (2016)	hIL-6	IL6	hPDLCs (12–28y/n.g., Healthy, M, dig., P3–6, n.g./ 90%)	Steady laminar	2h, 4h, 8h, 12h/n.g.	6dyn/cm²	Custom-made	Increase with plateau followed by decrease (sqPCR, GAPDH)	2h: 1.6/0.25 = 6.4 (ratio-calc)†<br>4h: 1.5/0.25 = 6 (ratio-calc)†<br>8h: 1.4/0.25 = 5.6 (ratio-calc) †<br>12h: 0.19/0.25 = 0.76 (ratio-calc)†			
Zheng et al. (2016)	hOPN	SPP1	hPDLCs (12–28y/n.g., Healthy, M, dig., P3–6, n.g./ 90%)	Steady laminar	2h, 4h, 8h, 12h/n.g.	6dyn/cm²	Custom-made	Increase (sqPCR, GAPDH)	8h: 1.5/0.01 = 150 (ratio-calc)†	Temporary increase, decrease, then Increase (ELISA)	2h: 1.76ng/mL; 1.76/1.6 = 1.1 (ratio-calc)†<br>8h: 0.9ng/mL; 0.9/1.6 = 0.6 (ratio-calc)†<br>12h: 2.72ng/mL; 2.72 /1.6 = 1.7 (ratio-calc)†	
Zheng et al. (2016)	hTGF-β	TGFB1	hPDLCs (12–28y/n.g., Healthy, M, dig., P3–6, n.g./ 90%)	Steady laminar	2h, 4h, 8h, 12h/n.g.	6dyn/cm²	Custom-made	Increase followed by plateau (sqPCR, GAPDH)	8h: 0.84/0.47 = 1.8 (ratio-calc)†<br>12h: 0.79/0.47 = 1.6 (ratio-calc)†			
Zheng et al. (2016)	hVEGF	VEGFA	hPDLCs (12–28y/n.g., Healthy, M, dig., P3–6, n.g./ 90%)	Steady laminar	2h, 4h, 8h, 12h/n.g.	6dyn/cm²	Custom-made	Increase (sqPCR, GAPDH)	8h: 0.9/0.01 = 90 (ratio-calc)†			
Maeda et al. (2007)	IL-1Beta	IL1B	hPDLCs (n.g./n.g., n.g., teeth (not given which one), exp., p3-4, n.g./ n.g.)	Pulsatile laminar	30min (sampling 1.5h, 2.5h, 3.5h, 4.5h, 6.5h post-PFF) @ 5Hz	0.6±0.3Pa	Custom-made	Decrease (RT-qPCR, GAPDH)	Cell line1 @ 6.5h Post-PFF: 0.069 (rel.)†<br>Cell line2 @ 4.5h Post-PFF: 0.076 (rel.)†			"Cell line 1":<br>"Cell line 2":<br>Cell lines difference was not clarified
Maeda et al. (2007)	IL-8	CXCL8	hPDLCs (n.g./n.g., n.g., teeth (not given which one), exp., p3-4, n.g./ n.g.)	Pulsatile laminar	30min (sampling 1.5h, 2.5h, 3.5h, 4.5h, 6.5h 24h, 48h, post-PFF) @ 5Hz	0.6±0.3Pa	Custom-made	Increase (RT-qPCR, GAPDH)	Cell line1 @ 3.5h Post-PFF: 37.3 (rel.)†<br>Cell line2 @ 3.5h Post-PFF: 23.4 (rel.)†	Increase (ELISA)	Cell line1 @ 24h Post-PFF: 67 pg/ml; 67/30.1 = 2.2 (ratio-calc)†<br>Cell line2 @ 48h Post-PFF: 86.1 pg/ml; 86.1/54.5 = 1.5 (ratio-calc)†	"Cell line 1":<br>"Cell line 2":<br>Cell lines difference was not clarified
Maeda et al. (2007)	MCP-I	CCL2 (unclear)	hPDLCs (n.g./n.g., n.g., teeth (not given which one), exp., p3-4, n.g./ n.g.)	Pulsatile laminar	30min (sampling 1.5h, 2.5h, 3.5h, 4.5h, 6.5h post-PFF) @ 5Hz	0.6±0.3Pa	Custom-made	Undetectable (RT-qPCR, GAPDH)	Undetectable			According to PCR primers CCL3; according to gene name CCL2!
Maeda et al. (2007)	MIP-1(alpha)	CCL3	hPDLCs (n.g./n.g., n.g., teeth (not given which one), exp., p3-4, n.g./ n.g.)	Pulsatile laminar	30min (sampling 1.5h, 2.5h, 3.5h, 4.5h, 6.5h post-PFF) @ 5Hz	0.6±0.3Pa	Custom-made	Cell line1: decrease (RT-qPCR, GAPDH)<br>Cell line2: Temporary decrease then increase with plateau followed by decrease then increase (qPCR, GAPDH)	Cell line1 @ 4.5h Post-PFF: 0.16 (rel.)†<br>Cell line2 @ 1.5h Post-PFF: 0.69 (rel.)†<br>Cell line2 @ 2.5h Post-PFF: 1.6 (rel.)†<br>Cell line2 @ 3.5h Post-PFF: 1.61 (rel.)†<br>Cell line2 @ 4.5h Post-PFF: 0.98 (rel.)†<br>Cell line2 @ 6.5h Post-PFF: 1.3 (rel.)†			"Cell line 1": ?<br>"Cell line 2": ?; cell line difference not specified;<br>Verified with Primer-BLAST.

[a] Entry provided as reported in the given study.
[b] Human genes were confirmed with the HUGO Gene Nomenclature Committee (HGNC; URL: https://www.genenames.org); mouse genes were confirmed with the Mouse Genome Informatics (MGI; URL: https://www.informatics.jax.org/genes.shtml) after checking the specificity of primers with Primer-BLAST.
[c] Sex of donors: "M" – male, "F" – female; Tooth type: "PM" – premolar, "M" – molar; dig. Indicate isolation by cell digestion; Exp. indicate isolation by cell explant; Cell density: given in cells/cm² if not otherwise mentioned.
[d] Flow type deduced from the description of the FSS apparatus given by the authors.
f RT-qPCR (reverse-transcriptase quantitative polymerase chain reaction); sqPCR (semi-quantitative polymerase chain reaction); ELISA (enzyme-linked immuno absorbent assay); WB (western blotting); RIA (radioimmunoassay); EMSA (electromobility shift assay); IF (immunofluorescence)

rel.: indicate relative gene expression. Is entitled to percentages or gene expression ratios normalized to control, and not calculated by ΔΔCT.
FC: indicate fold change. When Author mentions the use of ΔΔCT or the method according to Livak & Schmittken (2004) in calculating FC.
n.g.: not given. For information not given by study-authors.
† Information derived from figures using Engauge Digitizer.
* Indicate manual calculations by measuring the graphs, without using the Engauge Digitizer.
ratio-calc: indicate manual calculation by dividing intervention/control = result (ratio-calc)
ratio: indicate ratios given by study-authors such as normalization to control in case of small molecules data or in case of gene expression ratios, e.g. ratio of RANKL/OPG or Bcl-2/Bax.

Reference	Gene or analyte[a]	Official gene symbol or abbreviation[b]	Cell Type (age/sex of donor, health status, tooth type, isolation method, passages used, cell density/confluency)[a,c]	Flow type (steady laminar, pulsatile laminar, or oscillatory laminar)[a,d]	FSS duration and frequency[a]	FSS magnitude[a]	FSS-apparatus[a]	Gene expression: Increase, decrease, no change (method w/ reference gene); Methods: (RT-qPCR, sqPCR)[f]	Gene expression: when it reaches peak and peak's magnitude (fold change; relative gene expression; times or ratio; unclear = ?)[f]	Protein expression: Increase, decrease, no change (method w/reference); Methods: ELISA, WB, RIA, EMSA, IF[f]	Protein expression: When it reaches peak and peak's magnitude (times or ratio; unclear = ?)[f]	Remarks
Maeda et al. (2007)	RANTES	CCL5	hPDLCs (n.g./n.g., n.g., teeth (not given which one), exp., p3-4, n.g./ n.g.)	Pulsatile laminar	30min (sampling 1.5h, 2.5h, 3.5h, 4.5h, 6.5h post-PFF) @ 5Hz	0.6±0.3Pa	Custom-made	Cell line1: decrease with plateau then increase<br>Cell line 2: Increase (RT-qPCR, GAPDH)	Cell line1 @ 1.5h Post-PFF: 0.42 (rel.)†<br>Cell line1 @ 2.5h Post-PFF: 0.5 (rel.)†<br>Cell line1 @ 3.5h Post-PFF: 0.51 (rel.)†<br>Cell line1 @ 6.5h Post-PFF: 1.5 (rel.)†<br>Cell line2 @ 6.5h Post-PFF: 1.6 (rel.)†			"Cell line 1": ?<br>"Cell line 2": ?; cell line difference not specified
Zheng et al. (2019)	hMMP-2	MMP2	hPDLCs (12–28y/n.g., Healthy, M, dig., P3–6, n.g./ 80-90%)	Steady laminar	2h, 4h / n.g.	6dyn/cm²	Custom-made	Decrease (sqPCR, GAPDH)	4h: 0.20/0.26 = 0.76 (ratio-calc)*	Decrease (ELISA)	4h: 0.4ng/ml; 0.4/1.3 = 0.31 (ratio-calc)†	
Zheng et al. (2019)	hPDGFR-alpha	PDGFRA	hPDLCs (12–28y/n.g., Healthy, M, dig., P3–6, n.g./ 80-90%)	Steady laminar	2h, 4h / n.g.	6dyn/cm²	Custom-made	Decrease (sqPCR, GAPDH)	4h: 0.59 (rel.)†	Temporary increase then decrease (ELISA)	2h: 0.76pg/ml; 0.76/0.7 = 1.1 (ratio-calc)†<br>4h: 0.15pg/ml; 0.15/0.7 = 0.2 (ratio-calc)†	
Zheng et al. (2019)	hPDGFR-beta	PDGFRB	hPDLCs (12–28y/n.g., Healthy, M, dig., P3–6, n.g./ 80-90%)	Steady laminar	2h, 4h / n.g.	6dyn/cm²	Custom-made	Decrease (sqPCR, GAPDH)	4h: 0.48 (rel.)†	Decrease (ELISA)	4h: 0.16pg/ml; 0.16/0.5 = 0.32 (ratio-calc)†	
Qi and Zhang (2014)	Akt1	p-AKT1	immortalized hPDLCs ("I-PDL"; <25y/n.g., n.g., n.g., exp., n.g., n.g./70-80%)	Steady laminar	6h/n.g.	3dyn/cm², 6dyn/cm², 9dyn/cm², 12dyn/cm², 15dyn/cm²	Custom-made			shear stress (n.g.): increase (WB, β-actin)	No quantitative information given.	
Qi and Zhang (2014)	ALP	ALPL	immortalized hPDLCs ("I-PDL"; <25y/n.g., n.g., n.g., exp., n.g., n.g./70-80%)	Steady laminar	6h/n.g.	3dyn/cm², 6dyn/cm², 9dyn/cm², 12dyn/cm², 15dyn/cm²	Custom-made	Increase (RT-qPCR, GAPDH)	3dyn: 1.7 (FC)†<br>6dyn: 2.3 (FC)†<br>9dyn: 3.4 (FC)†<br>12dyn: 4 (FC)†<br>15dyn: 5.3 (FC)†	15dyn: increase (WB, β-actin)	No quantitative information given.	Verified by Primer-BLAST
Qi and Zhang (2014)	ARRAY		immortalized hPDLCs ("I-PDL"; <25y/n.g., n.g., n.g., exp., n.g., n.g./70-80%)	Steady laminar	6h/n.g.	3dyn/cm², 6dyn/cm², 9dyn/cm², 12dyn/cm², 15dyn/cm²	Custom-made	miRNA microarray (Agilent Whole Human Genome Array)				
Qi and Zhang (2014)	miRNA ARRAY		immortalized hPDLCs ("I-PDL"; <25y/n.g., n.g., n.g., exp., n.g., n.g./70-80%)	Steady laminar	6h/n.g.	3dyn/cm², 6dyn/cm², 9dyn/cm², 12dyn/cm², 15dyn/cm²	Custom-made	Agilent Whole Human Genome Array	no published datasets			
Qi and Zhang (2014)	mTOR	MTOR	immortalized hPDLCs ("I-PDL"; <25y/n.g., n.g., n.g., exp., n.g., n.g./70-80%)	Steady laminar	6h/n.g.	3dyn/cm², 6dyn/cm², 9dyn/cm², 12dyn/cm², 15dyn/cm²	Custom-made			shear stress(n.g.): increase (WB, β-actin)	No quantitative information given.	
Qi and Zhang (2014)	OCN	BGLAP	immortalized hPDLCs ("I-PDL"; <25y/n.g., n.g., n.g., exp., n.g., n.g./70-80%)	Steady laminar	6h/n.g.	3dyn/cm², 6dyn/cm², 9dyn/cm², 12dyn/cm², 15dyn/cm²	Custom-made	Increase (RT-qPCR, GAPDH)	3dyn: 1.5 (FC)†<br>6dyn: 2.4 (FC)†<br>9dyn: 2.7 (FC)†<br>12dyn: 3 (FC)†<br>15dyn: 3.1 (FC)†	15dyn: increase (WB, β-actin)	No quantitative information given.	
Qi and Zhang (2014)	OPN	SPP1	immortalized hPDLCs ("I-PDL"; <25y/n.g., n.g., n.g., exp., n.g., n.g./70-80%)	Steady laminar	6h/n.g.	3dyn/cm², 6dyn/cm², 9dyn/cm², 12dyn/cm², 15dyn/cm²	Custom-made	Increase (RT-qPCR, GAPDH)	3dyn: 1.6(FC)†<br>6dyn: 1.7 (FC)†<br>9dyn: 2 (FC)†<br>12dyn: 2.26(FC)†<br>15dyn: 2.8 (FC)†	15dyn: increase (WB, β-actin)	No quantitative information given.	
Qi and Zhang (2014)	p-Akt1	AKT1	immortalized hPDLCs ("I-PDL"; <25y/n.g., n.g., n.g., exp., n.g., n.g./70-80%)	Steady laminar	6h/n.g.	3dyn/cm², 6dyn/cm², 9dyn/cm², 12dyn/cm², 15dyn/cm²	Custom-made			shear stress (n.g.): increase (WB, Akt1)	No quantitative information given.	

[a] Entry provided as reported in the given study.
[b] Human genes were confirmed with the HUGO Gene Nomenclature Committee (HGNC; URL: https://www.genenames.org); mouse genes were confirmed with the Mouse Genome Informatics (MGI; URL: https://www.informatics.jax.org/genes.shtml) after checking the specificity of primers with Primer-BLAST.
[c] Sex of donors: "M" – male, "F" – female; Tooth type: "PM" – premolar, "M" – molar; dig. Indicate isolation by cell digestion; Exp. indicate isolation by cell explant; Cell density: given in cells/cm² if not otherwise mentioned.
[d] Flow type deduced from the description of the FSS apparatus given by the authors.
[f] RT-qPCR (reverse-transcriptase quantitative polymerase chain reaction); sqPCR (semi-quantitative polymerase chain reaction); ELISA (enzyme-linked immuno absorbent assay); WB (western blotting); RIA (radioimmunoassay); EMSA (electromobility shift assay); IF (immunofluorescence)

rel.: indicate relative gene expression. Is entitled to percentages or gene expression ratios normalized to control, and not calculated by ΔΔCT.
FC: indicate fold change. When Author mentions the use of ΔΔCT or the method according to Livak & Schmittken (2004) in calculating FC.
n.g.: not given. For information not given by study-authors.
† Information derived from figures using Engauge Digitizer.
* Indicate manual calculations by measuring the graphs, without using the Engauge Digitizer.
ratio-calc: indicate manual calculation by dividing intervention/control = result (ratio-calc)
ratio: indicate ratios given by study-authors such as normalization to control in case of small molecules data or in case of gene expression ratios, e.g. ratio of RANKL/OPG or Bcl-2/Bax.

Reference	Gene or analyte[a]	Official gene symbol or abbreviation[b]	Cell Type (age/sex of donor, health status, tooth type, isolation method, passages used, cell density/confluency)[a,c]	Flow type (steady laminar, pulsatile laminar, or oscillatory laminar)[a,d]	FSS duration and frequency[a]	FSS magnitude[a]	FSS-apparatus[a]	Gene expression: Increase, decrease, no change (method w/ reference gene); Methods: (RT-qPCR, sqPCR)[f]	Gene expression: when it reaches peak and peak's magnitude (fold change; relative gene expression; times or ratio; unclear = ?)[f]	Protein expression: Increase, decrease, no change (method w/reference); Methods: ELISA, WB, RIA, EMSA, IF[f]	Protein expression: When it reaches peak and peak's magnitude (times or ratio; unclear = ?)[f]	Remarks
Qi and Zhang (2014)	p-mTOR	MTOR	immortalized hPDLCs ("I-PDL"; <25y/n.g., n.g., n.g., exp., n.g., n.g./70-80%)	Steady laminar	6h/n.g.	3dyn/cm², 6dyn/cm², 9dyn/cm², 12dyn/cm², 15dyn/cm²	Custom-made			shear stress(n.g.): increase (WB, mTOR)	No quantitative information given.	
Qi and Zhang (2014)	p-p70S6K	RPS6KB1	immortalized hPDLCs ("I-PDL"; <25y/n.g., n.g., n.g., exp., n.g., n.g./70-80%)	Steady laminar	6h/n.g.	3dyn/cm², 6dyn/cm², 9dyn/cm², 12dyn/cm², 15dyn/cm²	Custom-made			Shear stress (n.g.): increase (WB, p70S6K)	No quantitative information given.	
Qi and Zhang (2014)	p-PI3K	PIK3CB	immortalized hPDLCs ("I-PDL"; <25y/n.g., n.g., n.g., exp., n.g., n.g./70-80%)	Steady laminar	6h/n.g.	3dyn/cm², 6dyn/cm², 9dyn/cm², 12dyn/cm², 15dyn/cm²	Custom-made			Shear stress (n.g.): increase (WB, PI3K)	No quantitative information given.	
Qi and Zhang (2014)	p70S6K (p70 S6 kinase)	RPS6KB1	immortalized hPDLCs ("I-PDL"; <25y/n.g., n.g., n.g., exp., n.g., n.g./70-80%)	Steady laminar	6h/n.g.	3dyn/cm², 6dyn/cm², 9dyn/cm², 12dyn/cm², 15dyn/cm²	Custom-made			Shear stress (n.g.): increase (WB, β-actin)	No quantitative information given.	A mitogen activated Ser/Thr protein kinase that is required for cell growth and G1 cell cycle progression.
Qi and Zhang (2014)	PI3K	PIK3CB	immortalized hPDLCs ("I-PDL"; <25y/n.g., n.g., n.g., exp., n.g., n.g./70-80%)	Steady laminar	6h/n.g.	3dyn/cm², 6dyn/cm², 9dyn/cm², 12dyn/cm², 15dyn/cm²	Custom-made			Shear stress (n.g.): increase (WB, β-actin)	No quantitative information given.	
Qi and Zhang (2014)	PON	PON1	immortalized hPDLCs ("I-PDL"; <25y/n.g., n.g., n.g., exp., n.g., n.g./70-80%)	Steady laminar	6h/n.g.	3dyn/cm², 6dyn/cm², 9dyn/cm², 12dyn/cm², 15dyn/cm²	Custom-made	Increase (RT-qPCR, GAPDH)	3dyn: 1.8 (FC)† 6dyn: 2.7 (FC)† 9dyn: 3.3 (FC)† 12dyn: 3.7 (FC)† 15dyn: 4.1 (FC)†	15dyn: increase (WB, β-actin)	No quantitative information given.	
Tang et al. (2014)	ALP	ALPL	hPDLCs (11–28y/3M+3F, healthy, PM, exp., P3–6, 1×10⁵ cells per cm²/ 70-80%)	Steady laminar	2h (sampling 0h, 6h, 12h, 24h, 48h post-FSS) / n.g.	12dyn/cm²	Custom-made	Temporary decrease followed by increase then decrease (RT-qPCR, GAPDH)	6h post-FSS: 0.8 (FC)† 12h post-FSS: 1.6 (FC)† 48h post-FSS: 0.95 (FC)†	Increase (ELISA)	24h post-FSS: 0.030U/mg; 0.030/0.019 = 1.6 (ratio-calc)†	ALP activity with p-nitrophenol substrate.
Tang et al. (2014)	BMP2	BMP2	hPDLCs (11–28y/3M+3F, healthy, PM, exp., P3–6, 1×10⁵ cells per cm²/ 70-80%)	Steady laminar	2h (sampling 0h, 6h, 12h, 24h, 48h post-FSS) / n.g.	12dyn/cm²	Custom-made	Increase then baseline (RT-qPCR, GAPDH)	6h post-FSS: 11.6 (FC)† 48h post-FSS: baseline (FC)†			
Tang et al. (2014)	COL-I	COL1A1	hPDLCs (11–28y/3M+3F, healthy, PM, exp., P3–6, 1×10⁵ cells per cm²/ 70-80%)	Steady laminar	2h (sampling 0h, 6h, 12h, 24h, 48h post-FSS) / n.g.	12dyn/cm²	Custom-made	Increase then decrease (RT-qPCR, GAPDH)	6h post-FSS: 2 (FC)† 48h post-FSS: 0.9 (FC)†			
Tang et al. (2014)	p-ERK1/2 / ERK1/2	MAPK3; MAPK1	hPDLCs (11–28y/3M+3F, healthy, PM, exp., P3–6, 1×10⁵ cells per cm²/ 70-80%)	Steady laminar	2h (sampling 0min, 15min, 30min, 60min post-FSS) / n.g.	12dyn/cm²	Custom-made			Decrease then Increase (WB, ERK1/2)	15min post-FSS: 0.8 (densitometric ratio)* 30min post-FSS: 1.4 (densitometric ratio)*	
Tang et al. (2014)	p-p38 / p38	MAPK14; MAPK11; MAPK12	hPDLCs (11–28y/3M+3F, healthy, PM, exp., P3–6, 1×10⁵ cells per cm²/ 70-80%)	Steady laminar	2h (sampling 0min, 15min, 30min, 60min post-FSS) / n.g.	12dyn/cm²	Custom-made			Increase (WB, p38)	15min post-FSS: 1.7 (densitometric ratio)*	
Tang et al. (2014)	RUNX2	RUNX2	hPDLCs (11–28y/3M+3F, healthy, PM, exp., P3–6, 1×10⁵ cells per cm²/ 70-80%)	Steady laminar	2h (sampling 0h, 6h, 12h, 24h, 48h post-FSS) / n.g.	12dyn/cm²	Custom-made	Increase (RT-qPCR, GAPDH)	12h post-FSS: 1.8 (FC)†			
Tang et al. (2014)	SP7	SP7	hPDLCs (11–28y/3M+3F, healthy, PM, exp., P3–6, 1×10⁵ cells per cm²/ 70-80%)	Steady laminar	2h (sampling 0h, 6h, 12h, 24h, 48h post-FSS) / n.g.	12dyn/cm²	Custom-made	Increase (RT-qPCR, GAPDH)	6h post-FSS: 3.1 (FC)†			
van der Pauw et al. (2000)	NO	Nitric oxide	hPDLF (12-40y/n.g., healthy. PM+M, exp., P3, n.g./ n.g.)	Pulsatile laminar	5min, 10min, 30min, 60min @ 5Hz	0.7±0.02Pa (estimated peak stress: 12.2Pa/s)	Custom-made			Increase (Griess, NO_2^-)	5min: 10.4 ± 24.6 (ratio) 10min: 10.9 ± 22.1 (ratio) 30min: 8.9 ± 16.2 (ratio) 60min: 11.2 ± 24.1 (ratio)	

[a] Entry provided as reported in the given study.
[b] Human genes were confirmed with the HUGO Gene Nomenclature Committee (HGNC; URL: https://www.genenames.org); mouse genes were confirmed with the Mouse Genome Informatics (MGI; URL: https://www.informatics.jax.org/genes.shtml) after checking the specificity of primers with Primer-BLAST.
[c] Sex of donors: "M" – male, "F" – female; Tooth type: "PM" – premolar, "M" – molar; dig. Indicate isolation by cell digestion; Exp. indicate isolation by cell explant; Cell density: given in cells/cm² if not otherwise mentioned.
[d] Flow type deduced from the description of the FSS apparatus given by the authors.
f RT-qPCR (reverse-transcriptase quantitative polymerase chain reaction); sqPCR (semi-quantitative polymerase chain reaction); ELISA (enzyme-linked immuno absorbent assay); WB (western blotting); RIA (radioimmunoassay); EMSA (electromobility shift assay); IF (immunofluorescence)

rel.: indicate relative gene expression. Is entitled to percentages or gene expression ratios normalized to control, and not calculated by ΔΔCT.
FC: indicate fold change. When Author mentions the use of ΔΔCT or the method according to Livak & Schmittken (2004) in calculating FC.
n.g.: not given. For information not given by study-authors.
† Information derived from figures using Engauge Digitizer.
* Indicate manual calculations by measuring the graphs, without using the Engauge Digitizer.
ratio-calc: indicate manual calculation by dividing intervention/control = result (ratio-calc)
ratio: indicate ratios given by study-authors such as normalization to control in case of small molecules data or in case of gene expression ratios, e.g. ratio of RANKL/OPG or Bcl-2/Bax.

Reference	Gene or analyte[a]	Official gene symbol or abbreviation[b]	Cell Type (age/sex of donor, health status, tooth type, isolation method, passages used, cell density/confluency)[a,c]	Flow type (steady laminar, pulsatile laminar, or oscillatory laminar)[a,d]	FSS duration and frequency[a]	FSS magnitude[a]	FSS-apparatus[a]	Gene expression: Increase, decrease, no change (method w/ reference gene); Methods: (RT-qPCR, sqPCR)[f]	Gene expression: when it reaches peak and peak's magnitude (fold change; relative gene expression; times or ratio; unclear = ?)[f]	Protein expression: Increase, decrease, no change (method w/reference); Methods: ELISA, WB, RIA, EMSA, IF[f]	Protein expression: When it reaches peak and peak's magnitude (times or ratio; unclear = ?)[f]	Remarks
van der Pauw et al. (2000)	PGE2	PGE2	hPDLF (12-40y/n.g., healthy. PM+M, exp., P3, n.g./ n.g.)	Pulsatile laminar	1h @ 5Hz	0.7±0.02Pa (estimated peak stress: 12.2Pa/s)	Custom-made			Increase (ELISA)	Cannot be calculated	
van der Pauw et al. (2000)	TNAP	ALP	hPDLF (12-40y/n.g., healthy. PM+M, exp., P3, n.g./ n.g.)	Pulsatile laminar	1h @ 5Hz	0.7±0.02Pa (estimated peak stress: 12.2Pa/s)	Custom-made			Decrease (ELISA)	Cannot be calculated	
Zheng et al. (2012)	MMP-1	MMP1	hPDLCs (12-28y/n.g., healthy, M, dig., P3-6, n.g./ 90%)	Steady laminar	2h, 4h, 8h, 12h/n.g.	6dyn/cm2, 9dyn/cm2, 12dyn/cm2	Custom-made	6dyn: Temporary decrease followed by Increase then decrease (RT-qPCR, GAPDH)<br>9dyn: Temporary decrease then Increase (RT-qPCR, GAPDH)<br>12dyn: increase followed by plateau (RT-qPCR, GAPDH)	6dyn @ 2h: 0.92 (rel.)†<br>6dyn @ 4h: 1.8 (rel.)†<br>6dyn @ 12h: 0.9 (rel.)†<br>9dyn @ 2h: 0.4 (rel.)†<br>9dyn @ 4h: 6.2 (rel.)†<br>12dyn @ 8h: 5.8 (rel.)†<br>12dyn @ 12h: 5.7 (rel.) †	Increase (ELISA)	6dyn @ 8h: 1490pg/ml*	
Zheng et al. (2012)	MMP-2	MMP2	hPDLCs (12-28y/n.g., healthy, M, dig., P3-6, n.g./ 90%)	Steady laminar	2h, 4h, 8h, 12h/n.g.	6dyn/cm2, 9dyn/cm2, 12dyn/cm2	Custom-made	6dyn: Temporary increase followed by temporary decrease then increase (RT-qPCR, GAPDH)<br>9dyn: decrease with plateau, Increase then decrease (RT-qPCR, GAPDH)<br>12dyn: increase (RT-qPCR, GAPDH)	6dyn @ 2h: 1.08 (rel.)†<br>6dyn @ 4h: 0.6 (rel.)†<br>6dyn @ 12h: 3.5 (rel.)†<br>9dyn @ 2-4h: 0.7 (rel.)†<br>9dyn @ 8h: 1.17 (rel.)†<br>9dyn @ 12h: 0.68 (rel.)†<br>12dyn @ 12h: 1.9 (rel.)†	Increase (ELISA)	6dyn @ 12h:13 ng/ml*	
Zheng et al. (2012)	p-ERK	MAPK3; MAPK1	hPDLCs (12-28y/n.g., healthy, M, dig., P3-6, n.g./ 90%)	Steady laminar	2h, 4h, 8h, 12h/n.g.	6dyn/cm2, 9dyn/cm2, 12dyn/cm2	Custom-made			Increase (WB, GAPDH)	5min (WB): 1.5 (ratio)*	
Zheng et al. (2012)	p-JNK	MAPK8	hPDLCs (12-28y/n.g., healthy, M, dig., P3-6, n.g./ 90%)	Steady laminar	2h, 4h, 8h, 12h/n.g.	6dyn/cm2, 9dyn/cm2, 12dyn/cm2	Custom-made			Increase (WB, GAPDH)	5min (WB):1.4 (ratio)*	
Zheng et al. (2012)	p-p38	MAPK14; MAPK11; MAPK12	hPDLCs (12-28y/n.g., healthy, M, dig., P3-6, n.g./ 90%)	Steady laminar	2h, 4h, 8h, 12h/n.g.	6dyn/cm2, 9dyn/cm2, 12dyn/cm2	Custom-made			Increase (WB, GAPDH)	10min (WB): 8 (ratio)*	
Zheng et al. (2012)	TIMP-1	TIMP1	hPDLCs (12-28y/n.g., healthy, M, dig., P3-6, n.g./ 90%)	Steady laminar	2h, 4h, 8h, 12h/n.g.	6dyn/cm2, 9dyn/cm2, 12dyn/cm2	Custom-made	6dyn: increase (RT-qPCR, GAPDH)<br>9dyn: Increase (RT-qPCR, GAPDH)<br>12dyn: increase (RT-qPCR, GAPDH)	6dyn @ 8h: 3.6 (rel.)†<br>9dyn @ 8h: 2.0 (rel.)†<br>12dyn @ 8h: 2.7 (rel.)†			
Zheng et al. (2012)	TIMP-2	TIMP2	hPDLCs (12-28y/n.g., healthy, M, dig., P3-6, n.g./ 90%)	Steady laminar	2h, 4h, 8h, 12h/n.g.	6dyn/cm2, 9dyn/cm2, 12dyn/cm2	Custom-made	6dyn: increase then decrease with plateau (RT-qPCR, GAPDH)<br>9dyn: decrease with plateau, increase then decrease (RT-qPCR, GAPDH)<br>12dyn: Temporary decrease, temporary increase then decrease (RT-qPCR, GAPDH)	6dyn @ 2h: 1.1 (rel.)†<br>6dyn @ 8h: 0.5 (rel.)†<br>6dyn @ 12h: 0.52 (rel.)†<br>9dyn @ 2-4h: 0.8 (rel.)†<br>9dyn @ 8h: 1.17 (rel.)†<br>9dyn @12h: 0.47 (rel.)†<br>12dyn @ 2h: 0.6 (rel.)†<br>12dyn @ 4h: 1.15 (rel.)†<br>12dyn @ 8h: 0.4 (rel.)†			

References

Maeda A, Soejima K, Bandow K, Kuroe K, Kakimoto K, Miyawaki S, Okamoto A, Matsuguchi T (2007). Force-induced IL-8 from periodontal ligament cells requires IL-1beta. J Dent Res; 86(7):629-34.

Qi L, Zhang Y (2014). The microRNA 132 regulates fluid shear stress-induced differentiation in periodontal ligament cells through mTOR signaling pathway. Cell Physiol Biochem; 33(2):433-45.

Tang M, Peng Z, Mai Z, Chen L, Mao Q, Chen Z, Chen Q, Liu L, Wang Y, Ai H (2014). Fluid shear stress stimulates osteogenic differentiation of human periodontal ligament cells via the extracellular signal-regulated kinase 1/2 and p38 mitogen-activated protein kinase signaling pathways. J Periodontol; 85(12):1806-13.

van der Pauw MT, Klein-Nulend J, van den Bos T, Burger EH, Everts V, Beertsen W (2000). Response of periodontal ligament fibroblasts and gingival fibroblasts to pulsating fluid flow: nitric oxide and prostaglandin E_2 release and expression of tissue non-specific alkaline phosphatase activity. J Periodontal Res; 35(6):335-43.

Zheng L, Huang Y, Song W, Gong X, Liu M, Jia X, Zhou G, Chen L, Li A, Fan Y (2012). Fluid shear stress regulates metalloproteinase-1 and 2 in human periodontal ligament cells: involvement of extracellular signal-regulated kinase (ERK) and P38 signaling pathways. J Biomech; 45(14):2368-75.

Zheng L, Chen L, Chen Y, Gui J, Li Q, Huang Y, Liu M, Jia X, Song W, Ji J, Gong X, Shi R, Fan Y (2016). The effects of fluid shear stress on proliferation and osteogenesis of human periodontal ligament cells. J Biomech; 49(4):572-9.

Zheng L, Shi Q, Na J, Liu N, Guo Y, Fan Y (2019). Platelet-Derived Growth Factor Receptor-α and β are Involved in Fluid Shear Stress Regulated Cell Migration in Human Periodontal Ligament Cells. Cell Mol Bioeng; 12(1):85-97.

[a] Entry provided as reported in the given study.
[b] Human genes were confirmed with the HUGO Gene Nomenclature Committee (HGNC; URL: https://www.genenames.org); mouse genes were confirmed with the Mouse Genome Informatics (MGI; URL: https://www.informatics.jax.org/genes.shtml) after checking the specificity of primers with Primer-BLAST.
[c] Sex of donors: "M" – male, "F" – female; Tooth type: "PM" – premolar, "M" – molar; dig. Indicate isolation by cell digestion; Exp. indicate isolation by cell explant; Cell density: given in cells/cm² if not otherwise mentioned.
[d] Flow type deduced from the description of the FSS apparatus given by the authors.
[f] RT-qPCR (reverse-transcriptase quantitative polymerase chain reaction); sqPCR (semi-quantitative polymerase chain reaction); ELISA (enzyme-linked immuno absorbent assay); WB (western blotting); RIA (radioimmunoassay); EMSA (electromobility shift assay); IF (immunofluorescence)

rel.: indicate relative gene expression. Is entitled to percentages or gene expression ratios normalized to control, and not calculated by ΔΔCT.
FC: indicate fold change. When Author mentions the use of ΔΔCT or the method according to Livak & Schmittken (2004) in calculating FC.
n.g.: not given. For information not given by study-authors.
† Information derived from figures using Engauge Digitizer.
* Indicate manual calculations by measuring the graphs, without using the Engauge Digitizer.
ratio-calc: indicate manual calculation by dividing intervention/control = result (ratio-calc)
ratio: indicate ratios given by study-authors such as normalization to control in case of small molecules data or in case of gene expression ratios, e.g. ratio of RANKL/OPG or Bcl-2/Bax.

2.5 Mouse osteoblasts

Reference	Gene or analyte[a]	Official gene symbol or abbreviation[b]	Cell Type (age/ number and sex of donor (health status), tooth type, isolation method, passages used, cell density/confluency)[a,c]	Flow type (steady laminar, pulsatile laminar, or oscillatory laminar)[a,d]	FSS duration and frequency[a]	FSS magnitude[a]	FSS apparatus[a]	Gene expression: Increase, decrease, no change (method w/ reference gene); methods: RT-qPCR, sqPCR[f]	Gene expression: when it reaches peak and peak's magnitude (fold change; relative gene expression; times or ratio; unclear = ?)[f]	Protein expression: Increase, decrease, no change (method w/reference); methods: ELISA, WB, RIA, EMSA, IF[f]	Protein expression: When it reaches peak and peak's magnitude (times or ratio; unclear = ?)[f]	Remarks
Lau et al. (2006)	ALP	Alp (unclear)	Primary mouse calvarial osteoblasts (8-week-old / n.g. (C57BL/6 = "B6"), n.g., dig., P 3–6, 50,000 cells / ~80%)	Steady laminar	30min (sampling 48h post-FSS) /n.g.	20dyn/cm^2	Cytodyne flow chamber			Increase (activity)	170/100 = 1.7 (ratio-calc)*	cellular ALP activity with p-nitrophenol substrate
Lau et al. (2006)	ARRAY		Primary mouse calvarial osteoblasts (8-week-old / n.g. (C57BL/6 = "B6"), n.g., dig., P 3–6, 50,000 cells / ~80%)	Steady laminar	30min (sampling 4h post-FSS) / n.g.	20dyn/cm^2	Cytodyne flow chamber	Data not published				
Lau et al. (2006)	Axin	Axin1	Primary mouse calvarial osteoblasts (8-week-old / n.g. (C57BL/6 = "B6"), n.g., dig., P 3–6, 50,000 cells / ~80%)	Steady laminar	30min (sampling 4h post-FSS) / n.g.	20dyn/cm^2	Cytodyne flow chamber	Increase (RT-qPCR, β-actin)	4h post-FSS: 3.48±0.82 (rel.)			
Lau et al. (2006)	Bmpr1	Bmpr1a	Primary mouse calvarial osteoblasts (8-week-old / n.g. (C57BL/6 = "B6"), n.g., dig., P 3–6, 50,000 cells / ~80%)	Steady laminar	30min (sampling 4h post-FSS) / n.g.	20dyn/cm^2	Cytodyne flow chamber	Increase (RT-qPCR, β-actin)	4h post-FSS: 3.64±0.41 (rel.)			Verified with Primer-BLAST
Lau et al. (2006)	Cox-2	Ptgs2	Primary mouse calvarial osteoblasts (8-week-old / n.g. (C57BL/6 = "B6"), n.g., dig., P 3–6, 50,000 cells / ~80%)	Steady laminar	30min/n.g.	20dyn/cm^2	Cytodyne flow chamber			increase (WB, actin)	350/100 = 3.5 (ratio-calc)*	
Lau et al. (2006)	Ctnnb1	Ctnnb1	Primary mouse calvarial osteoblasts (8-week-old / n.g. (C57BL/6 = "B6"), n.g., dig., P 3–6, 50,000 cells / ~80%)	Steady laminar	30min (sampling 4h post-FSS) / n.g.	20dyn/cm^2	Cytodyne flow chamber	Increase (RT-qPCR, β-actin)	4h post-FSS: 3.36±0.59 (rel.)			
Lau et al. (2006)	Dlx1	Dlx1	Primary mouse calvarial osteoblasts (8-week-old / n.g. (C57BL/6 = "B6"), n.g., dig., P 3–6, 50,000 cells / ~80%)	Steady laminar	30min (sampling 4h post-FSS) / n.g.	20dyn/cm^2	Cytodyne flow chamber	Increase (RT-qPCR, β-actin)	4h post-FSS: 1.70±0.38 (rel.)			
Lau et al. (2006)	Esr1	Esr1	Primary mouse calvarial osteoblasts (8-week-old / n.g. (C57BL/6 = "B6"), n.g., dig., P 3–6, 50,000 cells / ~80%)	Steady laminar	30min (sampling 4h post-FSS) / n.g.	20dyn/cm^2	Cytodyne flow chamber	Increase (RT-qPCR, β-actin)	4h post-FSS: 2.24±0.57 (rel.)			
Lau et al. (2006)	Igf1r	Igf1r	Primary mouse calvarial osteoblasts (8-week-old / n.g. (C57BL/6 = "B6"), n.g., dig., P 3–6, 50,000 cells / ~80%)	Steady laminar	30min (sampling 4h post-FSS) / n.g.	20dyn/cm^2	Cytodyne flow chamber	Increase (RT-qPCR, β-actin)	4h post-FSS: 2.28±0.39 (rel.)			
Lau et al. (2006)	Integrin B1	Itgb1	Primary mouse calvarial osteoblasts (8-week-old / n.g. (C57BL/6 = "B6"), n.g., dig., P 3–6, 50,000 cells / ~80%)	Steady laminar	30min (sampling 10min post FSS/n.g.	20dyn/cm^2	Cytodyne flow chamber			increase (WB, actin)	210/100 = 2.1 (ratio-calc)*	
Lau et al. (2006)	Lef1	Lef1	Primary mouse calvarial osteoblasts (8-week-old / n.g. (C57BL/6 = "B6"), n.g., dig., P 3–6, 50,000 cells / ~80%)	Steady laminar	30min (sampling 4h post-FSS) / n.g.	20dyn/cm^2	Cytodyne flow chamber	Increase (RT-qPCR, β-actin)	4h post-FSS: 2.79±0.49 (rel.)			
Lau et al. (2006)	Lrp5	Lrp5	Primary mouse calvarial osteoblasts (8-week-old / n.g. (C57BL/6 = "B6"), n.g., dig., P 3–6, 50,000 cells / ~80%)	Steady laminar	30min (sampling 4h post-FSS) / n.g.	20dyn/cm^2	Cytodyne flow chamber	Increase (RT-qPCR, β-actin)	4h post-FSS: 3.23±1.42 (rel.)			
Lau et al. (2006)	Ncoa1	Ncoa1	Primary mouse calvarial osteoblasts (8-week-old / n.g. (C57BL/6 = "B6"), n.g., dig., P 3–6, 50,000 cells / ~80%)	Steady laminar	30min (sampling 4h post-FSS) / n.g.	20dyn/cm^2	Cytodyne flow chamber	Increase (RT-qPCR, β-actin)	4h post-FSS: 2.76±0.81 (rel.)			
Lau et al. (2006)	p-Erk1/2 / total-Erk1/2	Mapk3; Mapk1	Primary mouse calvarial osteoblasts (8-week-old / n.g. (C57BL/6 = "B6"), n.g., dig., P 3–6, 50,000 cells / ~80%)	Steady laminar	30min/n.g.	20dyn/cm^2	Cytodyne flow chamber			increase (WB, ERK1/2)	320/100 = 3.2 (ratio-calc)*	
Lau et al. (2006)	Tgfb1	Tgfb1	Primary mouse calvarial osteoblasts (8-week-old / n.g. (C57BL/6 = "B6"), n.g., dig., P 3–6, 50,000 cells / ~80%)	Steady laminar	30min (sampling 4h post-FSS) / n.g.	20dyn/cm^2	Cytodyne flow chamber	Increase (RT-qPCR, β-actin)	4h post-FSS incubation: 2.44±0.71 (rel.)			
Lau et al. (2006)	Wnt1	Wnt1	Primary mouse calvarial osteoblasts (8-week-old / n.g. (C57BL/6 = "B6"), n.g., dig., P 3–6, 50,000 cells / ~80%)	Steady laminar	30min (sampling 4h post-FSS) / n.g.	20dyn/cm^2	Cytodyne flow chamber	Increase (RT-qPCR, β-actin)	4h post-FSS: 2.32±0.15 (rel.)			
Lau et al. (2006)	Wnt3a	Wnt3a	Primary mouse calvarial osteoblasts (8-week-old / n.g. (C57BL/6 = "B6"), n.g., dig., P 3–6, 50,000 cells / ~80%)	Steady laminar	30min (sampling 4h post-FSS) / n.g.	20dyn/cm^2	Cytodyne flow chamber	Increase (RT-qPCR, β-actin)	4h post-FSS: 3.28±0.86 (rel.)			

[a] Entry provided as reported in the given study.
[b] Human genes were confirmed with the HUGO Gene Nomenclature Committee (HGNC; URL: https://www.genenames.org); mouse genes were confirmed with the Mouse Genome Informatics (MGI; URL: https://www.informatics.jax.org/genes.shtml) after checking the specificity of primers with Primer-BLAST.
[c] Sex of donors: "M" – male, "F" – female; Tooth type: "PM" – premolar, "M" – molar; dig. Indicate isolation by cell digestion; Exp. indicate isolation by cell explant; Cell density: given in cells/cm^2 if not otherwise mentioned.
[d] Flow type deduced from the description of the FSS apparatus given by the authors.
[f] RT-qPCR (reverse-transcriptase quantitative polymerase chain reaction); sqPCR (semi-quantitative polymerase chain reaction); ELISA (enzyme-linked immuno absorbent assay); WB (western blotting); RIA (radioimmunoassay); EMSA (electromobility shift assay); IF (immunofluorescence)

rel.: indicate relative gene expression. Is entitled to percentages or gene expression ratios normalized to control, and not calculated by ΔΔCT.
FC: indicate fold change. When Author mentions the use of ΔΔCT or the method according to Livak & Schmittken (2004) in calculating FC.
n.g.: not given. For information not given by study-authors.
† Information derived from figures using Engauge Digitizer.
* Indicate manual calculations by measuring the graphs, without using the Engauge Digitizer.
ratio-calc: indicate manual calculation by dividing intervention/control = result (ratio-calc)
ratio: indicate ratios given by study-authors such as normalization to control in case of small molecules data or in case of gene expression ratios, e.g. ratio of RANKL/OPG or Bcl-2/Bax.

Reference	Gene or analyte[a]	Official gene symbol or abbreviation[b]	Cell Type (age/ number and sex of donor (health status), tooth type, isolation method, passages used, cell density/confluency)[a,c]	Flow type (steady laminar, pulsatile laminar, or oscillatory laminar)[a,d]	FSS duration and frequency[a]	FSS magnitude[a]	FSS apparatus[a]	Gene expression: Increase, decrease, no change (method w/ reference gene); methods: RT-qPCR, sqPCR[f]	Gene expression: when it reaches peak and peak's magnitude (fold change; relative gene expression; times or ratio; unclear = ?)[f]	Protein expression: Increase, decrease, no change (method w/reference); methods: ELISA, WB, RIA, EMSA, IF[f]	Protein expression: When it reaches peak and peak's magnitude (times or ratio; unclear = ?)[f]	Remarks
Lau et al. (2006)	Wnt5a	Wnt5a	Primary mouse calvarial osteoblasts (8-week-old / n.g. (C57BL/6 = "B6"), n.g., dig., P 3–6, 50,000 cells / ~80%)	Steady laminar	30min (sampling 4h post-FSS) / n.g.	20dyn/cm^2	Cytodyne flow chamber	Increase (RT-qPCR, β-actin)	4h post-FSS: 1.85±0.02 (rel.)			
Lau et al. (2006)	β-catenin	Ctnnb1	Primary mouse calvarial osteoblasts (8-week-old / n.g. (C57BL/6 = "B6"), n.g., dig., P 3–6, 50,000 cells / ~80%)	Steady laminar	30min/n.g.	20dyn/cm^2	Cytodyne flow chamber			increase (WB, actin)	200/100 = 2 (ratio-calc)*	
Suzuki et al. (2013)	Ca^{++}	Calcium	Primary mouse osteoblasts (n.g./ n.g. (WT C57BL/6 mice), n.g., dig., n.g., 1×10^6 cells per mL/ n.g.)	Steady laminar	10min/n.g.	5dyn/cm^2	BioFlux system (FlexCell)			Fluctuated increase (Fluo-4AM microscopy)		Using "constant" flow.
Yang et al. (2015)	c-Fos	Fos	Primary mouse osteoblasts (2-3 day/ n.g. (BALB/c neonatal mice), n.g., dig., n.g., 1.5×10^5 per slide/ n.g.)	Steady laminar	60min, 90min / n.g.	≈12dyn/cm^2	Custom-made			Increase (WB, GAPDH)	60min: 0.5/0.4 = 1.25 (ratio-calc)†	
Yang et al. (2015)	COX-2	Ptgs2	Primary mouse osteoblasts (2-3 day/ n.g. (BALB/c neonatal mice), n.g., dig., n.g., 1.5×10^5 per slide/ n.g.)	Steady laminar	60min, 90min / n.g.	≈12dyn/cm^2	Custom-made			Increase (WB, GAPDH)	90min: 0.9/0.8 = 1.12 (ratio-calc)†	
Yang et al. (2010)	Cyclin D1	Ccnd1	Calvarial osteoblasts (day 3–5 after birth/ 10 to 12 (C57BL/6J), n.g., dig., P2-4, 2×10^5/ n.g.)	Oscillatory laminar	30min, 1h, 2h / 1Hz	12dyn/cm^2	Custom-made			Increase (WB, GAPDH)	2h: 0.23/0.09 = 2.56 (ratio-calc)*	
Yang et al. (2010)	p-Akt / Akt	Akt1	Calvarial osteoblasts (day 3–5 after birth/ 10 to 12 (C57BL/6J), n.g., dig., P2-4, 2×10^5/ n.g.)	Oscillatory laminar	30min, 1h, 2h / 1Hz	12dyn/cm^2	Custom-made			Increase (WB, Akt)	30min: 0.58/0.21 = 2.76 (ratio-calc)*	
Yang et al. (2010)	p-Erk1/2 / Erk1/2	Mapk3; Mapk1	Calvarial osteoblasts (day 3–5 after birth/ 10 to 12 (C57BL/6J), n.g., dig., P2-4, 2×10^5/ n.g.)	Oscillatory laminar	5min, 15min, 30min, 60min / 1Hz	12dyn/cm^2	Custom-made			Increase then decrease (WB, total-Erk1/2)	15min: 0.81/0.4 = 2.03 (ratio-calc)*<br>5min: 0.95/0.4 = 2.37 (ratio-calc)*	
Yang et al. (2010)	p-GSK3β / GSK3β	Gsk3b	Calvarial osteoblasts (day 3–5 after birth/ 10 to 12 (C57BL/6J), n.g., dig., P2-4, 2×10^5/ n.g.)	Oscillatory laminar	30min, 1h, 2h / 1Hz	12dyn/cm^2	Custom-made			Increase (WB, total-Gsk3b)	60min: 1/0.87 = 1.14 (ratio-calc)*	
Yang et al. (2010)	Vinculin	Vcl	Calvarial osteoblasts (day 3–5 after birth/ 10 to 12 (C57BL/6J), n.g., dig., P2-4, 2×10^5/ n.g.)	Oscillatory laminar	30min, 1h, 2h / 1Hz	12dyn/cm^2	Custom-made			4h: decrease (WB, GAPDH)	No quantitative information given.	
Fu et al. (2008)	LIMK2	Limk2	Primary osteoblasts (2–3 days old/ n.g. (BALB/c mice), n.g., dig., P2, 4 $x10^4$ cells per slide/ 50%)	Steady laminar	1h / n.g.	12dyn/cm^2	µ-Slides (Ibidi) w/ BT00-100/YZ1515 peristaltic pump (LongerPump Inc., China)			Increase (IF staining, FITC-phalloidine and DAPI)	40.8/22 = 1.8 (ratio-calc)*	"Negative control siRNA that had no specific silencing effect was also synthesized by RiboBio"<br>R1: negative control exposed to FSS<br>R3: negative control Not exposed to FSS
Li et al. (2005)	PGE2	PGE2	Calvarial osteoblasts (3–5-day-old/ n.g. (C57BL/6), n.g., dig., n.g., 2000 cells per cm^2/ 90%)	Steady laminar	30min, 15–60min / n.g.	12dyn/cm^2	n.g.			Increase (ELISA)	60min: 37.4ng/mg; 37.4/5.1 = 7.3 (ratio-calc)†	
Bakker et al. (2003a)	NO	Nitric oxide	Primary mouse bone cells (n.g./ n.g. (Swiss albino mice), n.g., dig., n.g., 15–30×10^4/ n.g.)	Pulsating laminar	5min, 30 min / 5Hz	0.6Pa (w/ 0.3Pa pulse amplitude, 8.4Pa/s peak stress rate)	Custom-made			Increase (Griess, NO_2^-)	5min: 110.4nmol/mg; 110.4/61.5 = 1.8 (ratio-calc)†	
Bakker et al. (2003a)	PGE2	PGE2	Primary mouse bone cells (n.g./ n.g. (Swiss albino mice), n.g., dig., n.g., 15–30×10^4/ n.g.)	Pulsating laminar	5min, 30 min / 5Hz	0.6Pa (w/ 0.3Pa pulse amplitude, 8.4Pa/s peak stress rate)	Custom-made			Increase (ELISA)	30min: 52.6ng/mg; 52.6/30.1 = 1.74 (ratio-calc)†	
Rangaswami et al. (2012)	p-Akt / Akt	Akt1	Murine primary osteoblasts (5 -7day-old/ n.g. ($Prkg2^{+/+}$mice), n.g., exp., n.g., n.g./ n.g.)	Steady laminar	5min, 10min / n.g.	12dyn/cm^2	Cytodyne parallel plate flow chamber			5min: increase (WB, Akt)	No quantitative information given.	seems to be knock-out only mice (Figure2, C); also used human osteoblasts

[a] Entry provided as reported in the given study.
[b] Human genes were confirmed with the HUGO Gene Nomenclature Committee (HGNC; URL: https://www.genenames.org); mouse genes were confirmed with the Mouse Genome Informatics (MGI; URL: https://www.informatics.jax.org/genes.shtml) after checking the specificity of primers with Primer-BLAST.
[c] Sex of donors: "M" – male, "F" – female; Tooth type: "PM" – premolar, "M" – molar; dig. Indicate isolation by cell digestion; Exp. indicate isolation by cell explant; Cell density: given in cells/cm^2 if not otherwise mentioned.
[d] Flow type deduced from the description of the FSS apparatus given by the authors.
f RT-qPCR (reverse-transcriptase quantitative polymerase chain reaction); sqPCR (semi-quantitative polymerase chain reaction); ELISA (enzyme-linked immuno absorbent assay); WB (western blotting); RIA (radioimmunoassay); EMSA (electromobility shift assay); IF (immunofluorescence)

rel.: indicate relative gene expression. Is entitled to percentages or gene expression ratios normalized to control, and not calculated by ΔΔCT.
FC: indicate fold change. When Author mentions the use of ΔΔCT or the method according to Livak & Schmittken (2004) in calculating FC.
n.g.: not given. For information not given by study-authors.
† Information derived from figures using Engauge Digitizer.
* Indicate manual calculations by measuring the graphs, without using the Engauge Digitizer.
ratio-calc: indicate manual calculation by dividing intervention/control = result (ratio-calc)
ratio: indicate ratios given by study-authors such as normalization to control in case of small molecules data or in case of gene expression ratios, e.g. ratio of RANKL/OPG or Bcl-2/Bax.

Reference	Gene or analyte[a]	Official gene symbol or abbreviation[b]	Cell Type (age/ number and sex of donor (health status), tooth type, isolation method, passages used, cell density/confluency)[a,c]	Flow type (steady laminar, pulsatile laminar, or oscillatory laminar)[a,d]	FSS duration and frequency[a]	FSS magnitude[a]	FSS apparatus[a]	Gene expression: Increase, decrease, no change (method w/ reference gene); methods: RT-qPCR, sqPCR[f]	Gene expression: when it reaches peak and peak's magnitude (fold change; relative gene expression; times or ratio; unclear = ?)[f]	Protein expression: Increase, decrease, no change (method w/reference); methods: ELISA, WB, RIA, EMSA, IF[f]	Protein expression: When it reaches peak and peak's magnitude (times or ratio; unclear = ?)[f]	Remarks
Bakker et al. (2013)	NO	Nitric oxide	Mouse long-bone osteoblasts (8 and 28 weeks / n.g./M (C57BL/6J), n.g., dig., P3, 3×10^5 cells per slide/ n.g.)	Pulsatile laminar	0, 5, 10, 15, 20, 25, 30min / 5Hz	0.7±0.3Pa	Custom-made			Increase with plateau (Griess, NO_2^-)	5min: 12.69nmole/3×10^5; 12.6/3.5 = 3.6<br>30min: 14.9nmole/3×10^5; 14.9/5.2 = 2.9 (ratio-calc)†	
Bakker et al. (2013)	NO	Nitric oxide	Mouse calvaria-derived osteoblasts (n.g./ n.g.(C57BL/6J), n.g., dig., P3, 5×10^4 cells per mL/ n.g.)	Pulsatile laminar	30min / 5Hz	0.7±0.3Pa	μ-Slide IV (Ibidi), collagen coated			Increase (DAR4 M-AM)	1.1/0.2 = 5.5 (production rate)	Huesa, Helfrich, Aspden (2010) Parallel-plate fluid flow systems for bone cell stimulation. J Biomech 43:1182–1189
Thi et al. (2012)	PGE2	PGE2	Primary mouse osteoblasts (n.g./ n.g. (embryonic (E19–20) wild type), n.g., dig., n.g., 2×10^4 cells per cm^2/ n.g.)	Pulsatile laminar	1h / 1Hz	10dyn/cm^2	Cytodyne (La Jolla, CA)			Increase (ELISA)	1h: 1.7pg/ug; 1.7/ 0.7 = 2.4 (ratio-calc)†	
Mehrotra et al. (2006)	cAMP	cyclic_AMP	Primary osteoblastic calvariae cells (n.g./ n.g. (n.g.), n.g., dig., n.g., n.g./ n.g.)	Steady laminar	5min, 15min, 30min, 60min / n.g.	10dyn/cm^2	Custom-made			Increase (EIA)	5min: 1171.93 (pmol/mg); 1171.93/329.82 = 3.55 (ratio-calc)†	cAMP EIA assay (Cayman)
Mehrotra et al. (2006)	COX2	Ptgs2	Primary osteoblastic calvariae cells (n.g./ n.g. (n.g.), n.g., dig., n.g., n.g./ n.g.)	Steady laminar	1h (sampling 0h, 4h post FSS) / n.g.	10dyn/cm^2	Custom-made	4h post FSS: increase (Northern blot, GAPDH)	No quantitative information given.			
Mehrotra et al. (2006)	OPG	Tnfrsf11b	Primary osteoblastic calvariae cells (n.g./ n.g. (n.g.), n.g., dig., n.g., n.g./ n.g.)	Steady laminar	1h (sampling 0h, 2h, 4h, 8h post FSS) / n.g.	10dyn/cm^2	Custom-made	Increase (RT-qPCR, GAPDH)<br>Increase then decrease (Northern blot, GAPDH)	0h post-FSS incubation: 2.7±0.3 (FC)†<br>Northern blot: 2h post-FSS incubation: 1.1 (ratio)<br>8h post-FSS incubation: 0.5 (ratio)			
Mehrotra et al. (2006)	p-Erk1/2	Mapk3; Mapk1	Primary osteoblastic calvariae cells (n.g./ n.g. (n.g.), n.g., dig., n.g., n.g./ n.g.)	Steady laminar	0min, 5min, 15min, 30min, 60 / n.g.	10dyn/cm^2	Custom-made			5min FSS: increase (WB, ERK1/2)	No quantitative information is given	
Mehrotra et al. (2006)	RANKL	Tnfsf11	Primary osteoblastic calvariae cells (n.g./ n.g. (n.g.), n.g., dig., n.g., n.g./ n.g.)	Steady laminar	1h (sampling 0h, 2h, 4h, 8h post FSS) / n.g.	10dyn/cm^2	Custom-made	Increase (RT-qPCR, 18S)<br>Increase (Northern blot, GAPDH)	RT-qPCR: 2h post-FSS incubation: 22.8±4.9 (FC)<br>Northern blot: 8h post-FSS incubation: 8 (ratio)	2h post-FSS culture: increase (WB, actin)	No quantitative information is given	
Mehrotra et al. (2006)	RANKL / OPG	ratio (RANKL/OPG)	Primary osteoblastic calvariae cells (n.g./ n.g. (n.g.), n.g., dig., n.g., n.g./ n.g.)	Steady laminar	1h (sampling 0h, 2h, 4h post FSS) / n.g.	10dyn/cm^2	Custom-made	Increase (RT-qPCR, GAPDH)	4h post-FSS incubation: 30 (ratio)			
Bakker et al. (2001)	NO	Nitric oxide	Primary Mouse long bone cells (n.g./ n.g. (pregnant Swiss albino mice), n.g., dig., n.g., 5×10^5 cells per glass slide/ n.g.)	Pulsatile laminar	15min @ 5Hz	Mean SS: 0.64Pa<br>Peak SS: 8.40Pa	Custom-made			Increase (Griess, NO_2^-)	15min @ 5Hz(0.64Pa): 13.0nM/5×10^5 cells; 13.0/0.4 = 32.5 (ratio-calc)†	
Bakker et al. (2001)	PGE2	PGE2	Primary Mouse long bone cells (n.g./ n.g. (pregnant Swiss albino mice), n.g., dig., n.g., 5×10^5 cells per glass slide/ n.g.)	Pulsatile laminar	15min @ 9Hz	Mean SS:1.20 pa<br>Peak SS: 20.90Pa	Custom-made			Increase (ELISA)	15min @ 9Hz(1.20Pa): 8.7ng/5×10^5cells; 8.7/3.1 = 2.8 (ratio-calc)†	
Soejima et al. (2001)	NO	Nitric oxide	Primary mouse long bone cells (adult/ n.g. (Swiss albino mice), n.g., dig., n.g., 5×10^5 cells per slide/ n.g.)	Pulsatile laminar	30min @ 5Hz	FSS of 0.6±0.3Pa, estimated peak stress rate of 8.5Pa/s	Custom-made			Adult mouse long bone cells: increase (Griess, NO_2^-)	30min: 10.7nM/10^5 cells; 10.7/2.6 = 4.11 (ratio-calc)†	
Soejima et al. (2001)	NO	Nitric oxide	Primary mouse calvarial cells (adult/ n.g. (Swiss albino mice), n.g., dig., n.g., 5×10^5 cells per slide/ n.g)	Pulsatile laminar	30min @ 5Hz	FSS of 0.6±0.3Pa, estimated peak stress rate of 8.5Pa/s	Custom-made			Adult mouse calavarial cells: increase followed by plateau (Griess, NO_2^-)	5min: 6nM/10^5 cells; 6/1 = 6 (ratio-calc)*<br>20-30 min: 4.8nM/10^5 cells; 4.8/1.5 = 3.2 (ratio-calc)†	
Soejima et al. (2001)	NO	Nitric oxide	Primary neonatal mouse calvarial cells (3-4days/ n.g., n.g., dig., n.g., 5×10^5 cells per slide/ n.g)	Pulsatile laminar	30min @ 5Hz	FSS of 0.6±0.3Pa, estimated peak stress rate of 8.5Pa/s	Custom-made			Neonatal mouse calavarial cells: increase (Griess, NO_2^-)	15min: 19.4nM/10^5 cells; 19.4/9.9 = 1.95 (ratio-calc)†	

[a] Entry provided as reported in the given study.
[b] Human genes were confirmed with the HUGO Gene Nomenclature Committee (HGNC; URL: https://www.genenames.org); mouse genes were confirmed with the Mouse Genome Informatics (MGI; URL: https://www.informatics.jax.org/genes.shtml) after checking the specificity of primers with Primer-BLAST.
[c] Sex of donors: "M" – male, "F" – female; Tooth type: "PM" – premolar, "M" – molar; dig. Indicate isolation by cell digestion; Exp. indicate isolation by cell explant; Cell density: given in cells/cm^2 if not otherwise mentioned.
[d] Flow type deduced from the description of the FSS apparatus given by the authors.
[f] RT-qPCR (reverse-transcriptase quantitative polymerase chain reaction); sqPCR (semi-quantitative polymerase chain reaction); ELISA (enzyme-linked immuno absorbent assay); WB (western blotting); RIA (radioimmunoassay); EMSA (electromobility shift assay); IF (immunofluorescence)

rel.: indicate relative gene expression. Is entitled to percentages or gene expression ratios normalized to control, and not calculated by ΔΔCT.
FC: indicate fold change. When Author mentions the use of ΔΔCT or the method according to Livak & Schmittken (2004) in calculating FC.
n.g.: not given. For information not given by study-authors.
† Information derived from figures using Engauge Digitizer.
* Indicate manual calculations by measuring the graphs, without using the Engauge Digitizer.
ratio-calc: indicate manual calculation by dividing intervention/control = result (ratio-calc)
ratio: indicate ratios given by study-authors such as normalization to control in case of small molecules data or in case of gene expression ratios, e.g. ratio of RANKL/OPG or Bcl-2/Bax.

Reference	Gene or analyte[a]	Official gene symbol or abbreviation[b]	Cell Type (age/ number and sex of donor (health status), tooth type, isolation method, passages used, cell density/confluency)[a,c]	Flow type (steady laminar, pulsatile laminar, or oscillatory laminar)[a,d]	FSS duration and frequency[a]	FSS magnitude[a]	FSS apparatus[a]	Gene expression: Increase, decrease, no change (method w/ reference gene); methods: RT-qPCR, sqPCR[f]	Gene expression: when it reaches peak and peak's magnitude (fold change; relative gene expression; times or ratio; unclear = ?)[f]	Protein expression: Increase, decrease, no change (method w/reference); methods: ELISA, WB, RIA, EMSA, IF[f]	Protein expression: When it reaches peak and peak's magnitude (times or ratio; unclear = ?)[f]	Remarks
Klein-Nulend et al. (1997)	6-keto-PGF$_{1alpha}$	6-keto-PGF1alpha	Primary mouse calvarial cells (3- to 4-day-old/ n.g. (n.g.), n.g., dig., n.g., 5×10^5 cells per slide/ n.g.)	Pulsatile laminar	1h (sampling 1h post FSS) @ 5Hz	FSS: 0.7±0.03Pa, Peak stress rate: 12Pa/s	Custom-made			Increase (ELISA)	1h post FSS: 660.5 pg; 660.5/74.1 = 8.9 (ratio-calc)†	stable metabolite of PGI2
Klein-Nulend et al. (1997)	PGE2	PGE2	Primary mouse calvarial cells (3- to 4-day-old/ n.g. (n.g.), n.g., dig., n.g., 5×10^5 cells per slide/ n.g.)	Pulsatile laminar	1h (sampling 1h post FSS) @ 5Hz	FSS: 0.7±0.03Pa, Peak stress rate: 12Pa/s	Custom-made			Increase (ELISA)	1h post FSS: 553 pg; 553/111.8 = 4.9 (ratio-calc)†	
Klein-Nulend et al. (1997)	PGF$_{2alpha}$	PGF2alpha	Primary mouse calvarial cells (3- to 4-day-old/ n.g. (n.g.), n.g., dig., n.g., 5×10^5 cells per slide/ n.g.)	Pulsatile laminar	1h (sampling 1h post FSS) @ 5Hz	FSS: 0.7±0.03Pa, Peak stress rate: 12Pa/s	Custom-made			Increase (ELISA)	1h post FSS: 73.2 pg; 73.2/45.8 = 1.6 (ratio-calc)†	
Klein-Nulend et al. (1997)	PGHS-1	Ptgs1	Primary mouse calvarial cells (3- to 4-day-old/ n.g. (n.g.), n.g., dig., n.g., 5×10^5 cells per slide/ n.g.)	Pulsatile laminar	1h (sampling 0h, 1h post FSS) @ 5Hz	FSS: 0.7±0.03Pa, Peak stress rate: 12Pa/s	Custom-made	0h post FSS: no change (Northern blot, GAPDH) 1h post FSS: Increase (Northern blot, GAPDH)	1h post FSS: 200/75 = 2.7 (ratio-calc)*			
Klein-Nulend et al. (1997)	PGHS-2 (prostaglandin G/H synthase 2)	Ptgs2	Primary mouse calvarial cells (3- to 4-day-old/ n.g. (n.g.), n.g., dig., n.g., 5×10^5 cells per slide/ n.g.)	Pulsatile laminar	1h (sampling 0h, 1h post FSS) @ 5Hz	FSS: 0.7±0.03Pa, Peak stress rate: 12Pa/s	Custom-made	0h post FSS: Increase (Northern blot, GAPDH) 1h post FSS: Increase (Northern blot, GAPDH)	0h post FSS: 700/100 = 7 (ratio-calc)* 1h post FSS: 1450/150 = 9.7 (ratio-calc)*	Increase (ELISA)	1h post FSS: 31.8 (nM PEG_2); 31.8/ 13.9 = 2.3 (ratio-calc)†	
Klein-Nulend et al. (1996)	6-keto-PGF$_{1alpha}$	6-keto-PGF1alpha	Primary mouse calvarial bone cells (3-4 day old / n.g. (n.g.), n.g., dig., n.g., 25×10^3 cells per slide / n.g.)	Pulsatile laminar	1h (sampling 1h post FSS) / 5Hz	FSS: 0.5±0.02Pa, and the peak stress rate 0.4Pa/s	Custom-made			Increase (ELISA)	1h post FSS: 17.6nM; 17.6/10.9 = 1.6 (ratio-calc)†	stable metabolite of PGI2
Klein-Nulend et al. (1996)	PGE2	PGE2	Primary mouse calvarial bone cells (3-4 day old / n.g. (n.g.), n.g., dig., n.g., 25×10^3 cells per slide / n.g.)	Pulsatile laminar	1h (sampling 1h post FSS) / 5Hz	FSS: 0.5±0.02Pa, and the peak stress rate 0.4Pa/s	Custom-made			Increase (ELISA)	1h post FSS: 30.4nM; 30.4/10.8 = 2.8 (ratio-calc)†	
Klein-Nulend et al. (1996)	PGF$_{2\alpha}$	PGF2alpha	Primary mouse calvarial bone cells (3-4 day old / n.g. (n.g.), n.g., dig., n.g., 25×10^3 cells per slide / n.g.)	Pulsatile laminar	1h (sampling 1h post FSS) / 5Hz	FSS: 0.5±0.02Pa, and the peak stress rate 0.4Pa/s	Custom-made			Increase (ELISA)	1h post FSS: 1.7nM; 1.7/0.6 = 2.8 (ratio-calc)†	
Klein-Nulend et al. (1996)	TGF-beta	Tgfb1	Primary mouse calvarial bone cells (3-4 day old / n.g. (n.g.), n.g., dig., n.g., 25×10^3 cells per slide / n.g.)	Pulsatile laminar	1h (sampling 24h post FSS) / 5Hz	FSS: 0.5±0.02Pa, and the peak stress rate 0.4Pa/s	Custom-made			Increase (Colorimetric assay/radiometric assay)	24h post FSS:1.6ng/µg DNA; 1.6/4.6 = 0.34 (ratio-calc)*	
Bakker et al. (2003b)	PGE2	PGE2	Mouse long bone cells (n.g./ n.g. (adult Swiss albino mice), n.g., dig., n.g., 5×10^5 cells per slide/ n.g.)	Pulsatile laminar	1h (sampling 0h, 24h post FSS) / 5Hz	Mean SS of 0.6Pa, pulse amplitude of 0.3Pa	Custom-made			Increase (EIA)	0h post FSS: 8.96ng/5×10^5 cells; 8.96/5.9 = 1.5 (ratio-calc)† 24h post FSS: 3.8ng/5×10^5 cells; 3.8/1.9 = 2 (ratio-calc)†	
Kapur et al. (2010)	c-fos	Fos	Primary calvariae osteoblasts (8,12 and 10-week-old / n.g. (C57BL/6), n.g. dig., P3-6, 50000 cells/ ~80%)	Steady laminar	30min (sampling 4h post FSS) / n.g.	20dyn/cm^2	Cytodyne flow chamber	Increase (RT-qPCR, β-actin)	4h post FSS: 3.06±0.68 (FC)			
Kapur et al. (2010)	Ctnnb1	Ctnnb1	Primary calvariae osteoblasts (8,12 and 10-week-old / n.g. (C57BL/6), n.g. dig., P3-6, 50000 cells/ ~80%)	Steady laminar	30min (sampling 4h post FSS) / n.g.	20dyn/cm^2	Cytodyne flow chamber	Increase (RT-qPCR, β-actin)	4h post FSS: 2.62 ± 0.54 (FC)			
Kapur et al. (2010)	Dlx1	Dlx1	Primary calvariae osteoblasts (8,12 and 10-week-old / n.g. (C57BL/6), n.g. dig., P3-6, 50000 cells/ ~80%)	Steady laminar	30min (sampling 4h post FSS) / n.g.	20dyn/cm^2	Cytodyne flow chamber	Increase (RT-qPCR, β-actin)	4h post FSS: 1.68 ± 0.48 (FC)			
Kapur et al. (2010)	Era	Eral1	Primary calvariae osteoblasts (8,12 and 10-week-old / n.g. (C57BL/6), n.g. dig., P3-6, 50000 cells/ ~80%)	Steady laminar	30min (sampling 4h post FSS) / n.g.	20dyn/cm^2	Cytodyne flow chamber	Increase (RT-qPCR, β-actin)	4h post FSS: 2.40±0.75 (FC)			One delta ct ; Table (1)
Kapur et al. (2010)	Igf1r	Igf1r	Primary calvariae osteoblasts (8,12 and 10-week-old / n.g. (C57BL/6), n.g. dig., P3-6, 50000 cells/ ~80%)	Steady laminar	30min (sampling 4h post FSS) / n.g.	20dyn/cm^2	Cytodyne flow chamber	Increase (RT-qPCR, β-actin)	4h post FSS: 3.30±0.36 (FC)			"B6" should be C57BL/6; "C3H": seems, that some substrains are available!
Kapur et al. (2010)	Lepr	Lepr	Primary calvariae osteoblasts (8,12 and 10-week-old / n.g. (C57BL/6), n.g. dig., P3-6, 50000 cells/ ~80%)	Steady Laminar	30min (sampling 0h, 24h post FSS) / n.g.	20dyn/cm^2	Cytodyne flow chamber	Increase (RT-qPCR, β-actin)	0h post FSS: 1.4 (FC)†	Increase (EIA)	24h post FSS: 5.8pg/mg; 5.8/2.3 = 2.5 (ratio-calc)†	Figure 8, A

[a] Entry provided as reported in the given study.
[b] Human genes were confirmed with the HUGO Gene Nomenclature Committee (HGNC; URL: https://www.genenames.org); mouse genes were confirmed with the Mouse Genome Informatics (MGI; URL: https://www.informatics.jax.org/genes.shtml) after checking the specificity of primers with Primer-BLAST.
[c] Sex of donors: "M" – male, "F" – female; Tooth type: "PM" – premolar, "M" – molar; dig. Indicate isolation by cell digestion; Exp. indicate isolation by cell explant; Cell density: given in cells/cm^2 if not otherwise mentioned.
[d] Flow type deduced from the description of the FSS apparatus given by the authors.
f RT-qPCR (reverse-transcriptase quantitative polymerase chain reaction); sqPCR (semi-quantitative polymerase chain reaction); ELISA (enzyme-linked immuno absorbent assay); WB (western blotting); RIA (radioimmunoassay); EMSA (electromobility shift assay); IF (immunofluorescence)

rel.: indicate relative gene expression. Is entitled to percentages or gene expression ratios normalized to control, and not calculated by ΔΔCT.
FC: indicate fold change. When Author mentions the use of ΔΔCT or the method according to Livak & Schmittken (2004) in calculating FC.
n.g.: not given. For information not given by study-authors.
† Information derived from figures using Engauge Digitizer.
* Indicate manual calculations by measuring the graphs, without using the Engauge Digitizer.
ratio-calc: indicate manual calculation by dividing intervention/control = result (ratio-calc)
ratio: indicate ratios given by study-authors such as normalization to control in case of small molecules data or in case of gene expression ratios, e.g. ratio of RANKL/OPG or Bcl-2/Bax.

Reference	Gene or analyte[a]	Official gene symbol or abbreviation[b]	Cell Type (age/ number and sex of donor (health status), tooth type, isolation method, passages used, cell density/confluency)[a,c]	Flow type (steady laminar, pulsatile laminar, or oscillatory laminar)[a,d]	FSS duration and frequency[a]	FSS magnitude[a]	FSS apparatus[a]	Gene expression: Increase, decrease, no change (method w/ reference gene); methods: RT-qPCR, sqPCR[f]	Gene expression: when it reaches peak and peak's magnitude (fold change; relative gene expression; times or ratio; unclear = ?)[f]	Protein expression: Increase, decrease, no change (method w/reference); methods: ELISA, WB, RIA, EMSA, IF[f]	Protein expression: When it reaches peak and peak's magnitude (times or ratio; unclear = ?)[f]	Remarks
Kapur et al. (2010)	Ncoa1	Ncoa1	Primary calvariae osteoblasts (8,12 and 10-week-old / n.g. (C57BL/6), n.g. dig., P3-6, 50000 cells/ ~80%)	Steady laminar	30min (sampling 4h post FSS) / n.g.	20dyn/cm^2	Cytodyne flow chamber	Increase (RT-qPCR, β-actin)	4h post FSS: 2.89±0.11 (FC)			
Kapur et al. (2010)	p-Erk1/2	Mapk3; Mapk1	Primary calvariae osteoblasts (8,12 and 10-week-old / n.g. (C57BL/6), n.g. dig., P3-6, 50000 cells/~80%)	Steady laminar	30min/n.g.	20dyn/cm^2	Cytodyne flow chamber			30min: increase (WB, ERK1/2)	300/100= 3 (ratio-calc)*	Figure2B
Kapur et al. (2010)	p(Y)-JAK2	Jak2	Primary calvariae osteoblasts (8,12 and 10-week-old / n.g. (C57BL/6), n.g. dig., P3-6, 50000 cells/~80%)	Steady Laminar	30min/n.g.	20dyn/cm^2	Cytodyne flow chamber			increase (WB, Total JAK2)	61%*	
Kapur et al. (2010)	p(Y)-STAT3	Stat3	Primary calvariae osteoblasts (8,12 and 10-week-old / n.g. (C57BL/6), n.g. dig., P3-6, 50000 cells/~80%)	Steady Laminar	30min/n.g.	20dyn/cm^2	Cytodyne flow chamber			increase (WB, Total STAT3)	67%*	
Kapur et al. (2010)	Wnt1	Wnt1	Primary calvariae osteoblasts (8,12 and 10-week-old / n.g. (C57BL/6), n.g. dig., P3-6, 50000 cells/~80%)	Steady laminar	30min (sampling 4h post FSS) / n.g.	20dyn/cm^2	Cytodyne flow chamber	Increase (RT-qPCR, β-actin)	4h post FSS: 2.71±0.74 (FC)			
Kapur et al. (2010)	Wnt3a	Wnt3a	Primary calvariae osteoblasts (8,12 and 10-week-old / n.g. (C57BL/6), n.g. dig., P3-6, 50000 cells/~80%)	Steady laminar	30min (sampling 4h post FSS) / n.g.	20dyn/cm^2	Cytodyne flow chamber	Increase (RT-qPCR, β-actin)	4h post FSS: 2.56±0.75 (FC)			
Xing et al. (2014)	p-Erk1/2	Mapk3; Mapk1	Primary osteoblastic cells (6 to 12 weeks/ n.g. (n.g.), n.g., dig., n.g., n.g./ n.g.)	Oscillatory laminar	5min, 15min @1Hz	10dyn/cm^2	n.g.			5min: increase (WB, total ERK)	No quantitative information given.	
Castillo et al. (2014)	Cox-2	Ptgs2	Primary mouse calvaria cells (3- to 5-day-old/ n.g. (WT), n.g., dig., P4-6, 2,500 per cm^2/ 80%)	Oscillatory laminar	4h (sampling 1h post FSS) @1Hz	15dyn/cm^2	Custom-made			Increase (WB, vinculin)	1h post FSS: 1.2/0.53 = 2.3 (ratio-calc)†	
Castillo et al. (2014)	p(Y)-ERK1/2; ERK1/2	Mapk3; Mapk1	Primary mouse calvaria cells (3- to 5-day-old/ n.g. (WT), n.g., dig., P4-6, 2500 per cm^2/ 80%)	Oscillatory laminar	30min @1Hz	15dyn/cm^2	Custom-made			Increase (WB, total ERK)	30 min: 4.5/1.1 = 4 (ratio-calc)†	BERKO: β-estrogen receptor knockout
Castillo et al. (2014)	PGE2	PGE2	Primary mouse calvaria cells (3- to 5-day-old/ n.g. (WT), n.g., dig., P4-6, 2500 per cm^2/ 80%)	Oscillatory laminar	4h (sampling 1h post FSS) @1Hz	15dyn/cm^2	Custom-made			Increase (ELISA)	1h post FSS: 24.7ng/mg; 24.7/7.1 = 3.5 (ratio-calc)†	
Kido et al. (2009)	Dkk1	Dkk1	Murine primary osteoblasts (mPOBs) (n.g./ n.g. (n.g.), n.g., dig., n.g., 1×10^5 cells per mL/ 70-80%)	Oscillatory laminar	0.5h, 2h, 3h, 6h / n.g.	100–120rpm, 200rpm, 2Pa	horizontal shaking apparatus	Decrease (RT-qPCR, GAPDH)	n.g.: 56.4/99.7 = 0.57 (ratio-calc)†			
Kido et al. (2009)	Dkk2	Dkk2	Murine primary osteoblasts (mPOBs) (n.g./ n.g. (n.g.), n.g., dig., n.g., 1×10^5 cells per mL/ 70-80%)	Oscillatory laminar	0.5h, 2h, 3h, 6h / n.g.	100–120rpm, 200rpm, 2Pa	horizontal shaking apparatus	Decrease (RT-qPCR, GAPDH)	n.g.: 33.1/99.7 = 0.33 (ratio-calc)†			
Kido et al. (2009)	IL-11	Il11	Murine primary osteoblasts (mPOBs) (n.g./ n.g. (n.g.), n.g., dig., n.g., 1×10^5 cells per mL/ 70-80%)	Oscillatory laminar	0.5h, 2h, 3h, 6h / n.g.	100–120rpm, 200rpm, 2Pa	horizontal shaking apparatus	Increase (RT-qPCR, GAPDH)	2h: 8.3 (rel.)†	24h: increase (WB, n.g.)	No quantitative information given.	
Kido et al. (2009)	sFRP1 (secreted frizzle-related protein 1)	Sfrp1	Murine primary osteoblasts (mPOBs) (n.g./ n.g. (n.g.), n.g., dig., n.g., 1×10^5 cells per mL/ 70-80%)	Oscillatory laminar	0.5h, 2h, 3h, 6h / n.g.	100–120rpm, 200rpm, 2Pa	horizontal shaking apparatus	Decrease (RT-qPCR, GAPDH)	n.g.: 93.4/99.7 = 0.9 (ratio-calc)†			
Kido et al. (2009)	sFRP2	Sfrp2	Murine primary osteoblasts (mPOBs) (n.g./ n.g. (n.g.), n.g., dig., n.g., 1×10^5 cells per mL/ 70-80%)	Oscillatory laminar	0.5h, 2h, 3h, 6h / n.g.	100–120rpm, 200rpm, 2Pa	horizontal shaking apparatus	Decrease (RT-qPCR, GAPDH)	n.g.: 94.9/ 99.2 = 0.95 (ratio-calc)†			
Callewaert et al. (2010)	COX2	Ptgs2	Primary mouse bone cells (n.g./ n.g. (WT), n.g., dig., n.g., 2 x10^5 cells per slide/ n.g.)	Pulsating laminar	1h @ 5Hz	Mean SS of 0.6Pa, Pulse amplitude of 0.3Pa	n.g.	Increase (RT-qPCR, HPRT)	1h: 3.14±1.72/0.42±0.17 = 7.5 (ratio-calc)			No reference for chamber used given.
Callewaert et al. (2010)	NO	Nitric oxide	Primary mouse bone cells (n.g./ n.g. (WT), n.g., dig., n.g., 2 x10^5 cells per slide/ n.g.)	Pulsating laminar	10min @ 5Hz	Mean SS of 0.6Pa, Pulse amplitude of 0.3Pa	n.g.			Increase (Griess, NO_2^-)	10 min: 0.77pmol/ng DNA; 0.77/0.26 = 2.96 (ratio-calc)†	No reference for chamber used given.

[a] Entry provided as reported in the given study.
[b] Human genes were confirmed with the HUGO Gene Nomenclature Committee (HGNC; URL: https://www.genenames.org); mouse genes were confirmed with the Mouse Genome Informatics (MGI; URL: https://www.informatics.jax.org/genes.shtml) after checking the specificity of primers with Primer-BLAST.
[c] Sex of donors: "M" – male, "F" – female; Tooth type: "PM" – premolar, "M" – molar; dig. Indicate isolation by cell digestion; Exp. indicate isolation by cell explant; Cell density: given in cells/cm^2 if not otherwise mentioned.
[d] Flow type deduced from the description of the FSS apparatus given by the authors.
[f] RT-qPCR (reverse-transcriptase quantitative polymerase chain reaction); sqPCR (semi-quantitative polymerase chain reaction); ELISA (enzyme-linked immuno absorbent assay); WB (western blotting); RIA (radioimmunoassay); EMSA (electromobility shift assay); IF (immunofluorescence)

rel.: indicate relative gene expression. Is entitled to percentages or gene expression ratios normalized to control, and not calculated by ΔΔCT.
FC: indicate fold change. When Author mentions the use of ΔΔCT or the method according to Livak & Schmittken (2004) in calculating FC.
n.g.: not given. For information not given by study-authors.
† Information derived from figures using Engauge Digitizer.
* Indicate manual calculations by measuring the graphs, without using the Engauge Digitizer.
ratio-calc: indicate manual calculation by dividing intervention/control = result (ratio-calc)
ratio: indicate ratios given by study-authors such as normalization to control in case of small molecules data or in case of gene expression ratios, e.g. ratio of RANKL/OPG or Bcl-2/Bax.

Reference	Gene or analyte[a]	Official gene symbol or abbreviation[b]	Cell Type (age/ number and sex of donor (health status), tooth type, isolation method, passages used, cell density/confluency)[a,c]	Flow type (steady laminar, pulsatile laminar, or oscillatory laminar)[a,d]	FSS duration and frequency[a]	FSS magnitude[a]	FSS apparatus[a]	Gene expression: Increase, decrease, no change (method w/ reference gene); methods: RT-qPCR, sqPCR[f]	Gene expression: when it reaches peak and peak's magnitude (fold change; relative gene expression; times or ratio; unclear = ?)[f]	Protein expression: Increase, decrease, no change (method w/reference); methods: ELISA, WB, RIA, EMSA, IF[f]	Protein expression: When it reaches peak and peak's magnitude (times or ratio; unclear = ?)[f]	Remarks
Igwe et al. (2009)	Dmp1	Dmp1	Primary mouse calvarial osteoblastic cells (7-day-old / n.g. (n.g.), n.g., dig., n.g., 100000 cells per slide / n.g.)	Steady laminar	30min @ 5Hz	10dyn/cm^2	Custom-made	Increase (RT-qPCR, 18S)	30min: 2.74 (rel.)†			
Igwe et al. (2009)	NPY	Npy	Primary mouse calvarial osteoblastic cells (7-day-old / n.g. (n.g.), n.g., dig., n.g., 100000 cells per slide / n.g.)	Steady laminar	30min @ 5Hz	10dyn/cm^2	Custom-made	Decrease (RT-qPCR, 18S)	30min: 0.59 (rel.)†			

References

Bakker AD, Soejima K, Klein-Nulend J, Burger EH (2001). The production of nitric oxide and prostaglandin E(2) by primary bone cells is shear stress dependent. J Biomech; 34(5):671-7.

Bakker AD, Joldersma M, Klein-Nulend J, Burger EH (2003a). Interactive effects of PTH and mechanical stress on nitric oxide and PGE2 production by primary mouse osteoblastic cells. Am J Physiol Endocrinol Metab; 285(3):E608-13.

Bakker AD, Klein-Nulend J, Burger EH (2003b). Mechanotransduction in bone cells proceeds via activation of COX-2, but not COX-1. Biochem Biophys Res Commun; 305(3):677-83.

Bakker AD, Huesa C, Hughes A, Aspden RM, van't Hof RJ, Klein-Nulend J, Helfrich MH (2013). Endothelial nitric oxide synthase is not essential for nitric oxide production by osteoblasts subjected to fluid shear stress in vitro. Calcif Tissue Int; 92(3):228-39.

Callewaert F, Bakker A, Schrooten J, Van Meerbeek B, Verhoeven G, Boonen S, Vanderschueren D (2010). Androgen receptor disruption increases the osteogenic response to mechanical loading in male mice. J Bone Miner Res; 25(1):124-31.

Castillo AB, Triplett JW, Pavalko FM, Turner CH (2014). Estrogen receptor-β regulates mechanical signaling in primary osteoblasts. Am J Physiol Endocrinol Metab; 306(8):E937-44.

Fu Q, Wu C, Shen Y, Zheng S, Chen R (2008). Effect of LIMK2 RNAi on reorganization of the actin cytoskeleton in osteoblasts induced by fluid shear stress. J Biomech; 41(15):3225-8.

Igwe JC, Jiang X, Paic F, Ma L, Adams DJ, Baldock PA, Pilbeam CC, Kalajzic I (2009). Neuropeptide Y is expressed by osteocytes and can inhibit osteoblastic activity. J Cell Biochem; 108(3):621-30.

Kapur S, Amoui M, Kesavan C, Wang X, Mohan S, Baylink DJ, Lau KH (2010). Leptin receptor (Lepr) is a negative modulator of bone mechanosensitivity and genetic variations in Lepr may contribute to the differential osteogenic response to mechanical stimulation in the C57BL/6J and C3H/HeJ pair of mouse strains. J Biol Chem; 285(48):37607-18.

Kido S, Kuriwaka-Kido R, Imamura T, Ito Y, Inoue D, Matsumoto T (2009). Mechanical stress induces Interleukin-11 expression to stimulate osteoblast differentiation. Bone; 45(6):1125-32.

Klein-Nulend J, Semeins CM, Burger EH (1996). Prostaglandin mediated modulation of transforming growth factor-beta metabolism in primary mouse osteoblastic cells in vitro. J Cell Physiol; 168(1):1-7.

Klein-Nulend J, Burger EH, Semeins CM, Raisz LG, Pilbeam CC (1997). Pulsating fluid flow stimulates prostaglandin release and inducible prostaglandin G/H synthase mRNA expression in primary mouse bone cells. J Bone Miner Res; 12(1):45-51.

Lau KH, Kapur S, Kesavan C, Baylink DJ (2006). Up-regulation of the Wnt, estrogen receptor, insulin-like growth factor-I, and bone morphogenetic protein pathways in C57BL/6J osteoblasts as opposed to C3H/HeJ osteoblasts in part contributes to the differential anabolic response to fluid shear. J Biol Chem; 281(14):9576-88.

Li J, Liu D, Ke HZ, Duncan RL, Turner CH (2005). The P2X7 nucleotide receptor mediates skeletal mechanotransduction. J Biol Chem; 280(52):42952-9.

Mehrotra M, Saegusa M, Wadhwa S, Voznesensky O, Peterson D, Pilbeam C (2006). Fluid flow induces Rankl expression in primary murine calvarial osteoblasts. J Cell Biochem; 98(5):1271-83.

Rangaswami H, Schwappacher R, Tran T, Chan GC, Zhuang S, Boss GR, Pilz RB (2012). Protein kinase G and focal adhesion kinase converge on Src/Akt/β-catenin signaling module in osteoblast mechanotransduction. J Biol Chem; 287(25):21509-19.

Soejima K, Klein-Nulend J, Semeins CM, Burger EH (2001). Different responsiveness of cells from adult and neonatal mouse bone to mechanical and biochemical challenge. J Cell Physiol; 186(3):366-70.

Suzuki T, Notomi T, Miyajima D, Mizoguchi F, Hayata T, Nakamoto T, Hanyu R, Kamolratanakul P, Mizuno A, Suzuki M, Ezura Y, Izumi Y, Noda M (2013). Osteoblastic differentiation enhances expression of TRPV4 that is required for calcium oscillation induced by mechanical force. Bone; 54(1):172-8.

Thi MM, Islam S, Suadicani SO, Spray DC (2012). Connexin43 and pannexin1 channels in osteoblasts: who is the "hemichannel"? J Membr Biol; 245(7):401-9.

Xing Y, Gu Y, Bresnahan JJ, Paul EM, Donahue HJ, You J (2014). The roles of P2Y2 purinergic receptors in osteoblasts and mechanotransduction. PLoS One; 9(9):e108417.

Yang Z, Bidwell JP, Young SR, Gerard-O'Riley R, Wang H, Pavalko FM (2010). Nmp4/CIZ inhibits mechanically induced beta-catenin signaling activity in osteoblasts. J Cell Physiol; 223(2):435-41.

Yang Z, Tan S, Shen Y, Chen R, Wu C, Xu Y, Song Z, Fu Q (2015). Inhibition of FSS-induced actin cytoskeleton reorganization by silencing LIMK2 gene increases the mechanosensitivity of primary osteoblasts. Bone; 74:182-90.

[a] Entry provided as reported in the given study.
[b] Human genes were confirmed with the HUGO Gene Nomenclature Committee (HGNC; URL: https://www.genenames.org); mouse genes were confirmed with the Mouse Genome Informatics (MGI; URL: https://www.informatics.jax.org/genes.shtml) after checking the specificity of primers with Primer-BLAST.
[c] Sex of donors: "M" – male, "F" – female; Tooth type: "PM" – premolar, "M" – molar; dig. Indicate isolation by cell digestion; Exp. indicate isolation by cell explant; Cell density: given in cells/cm^2 if not otherwise mentioned.
[d] Flow type deduced from the description of the FSS apparatus given by the authors.
f RT-qPCR (reverse-transcriptase quantitative polymerase chain reaction); sqPCR (semi-quantitative polymerase chain reaction); ELISA (enzyme-linked immuno absorbent assay); WB (western blotting); RIA (radioimmunoassay); EMSA (electromobility shift assay); IF (immunofluorescence)

rel.: indicate relative gene expression. Is entitled to percentages or gene expression ratios normalized to control, and not calculated by ΔΔCT.
FC: indicate fold change. When Author mentions the use of ΔΔCT or the method according to Livak & Schmittken (2004) in calculating FC.
n.g.: not given. For information not given by study-authors.
† Information derived from figures using Engauge Digitizer.
* Indicate manual calculations by measuring the graphs, without using the Engauge Digitizer.
ratio-calc: indicate manual calculation by dividing intervention/control = result (ratio-calc)
ratio: indicate ratios given by study-authors such as normalization to control in case of small molecules data or in case of gene expression ratios, e.g. ratio of RANKL/OPG or Bcl-2/Bax.

2.6 Mouse osteocytes

Reference	Gene or analyte[a]	Official gene symbol or abbreviation[b]	Cell type (age/ number and sex of donor (health status), tooth type, isolation method, passages used, cell density/confluency)[a,c]	Flow type (Steady laminar, Pulsatile laminar, or Oscillatory laminar)[a,d]	FSS-duration and frequency[a]	FSS-magnitude[a]	FSS apparatus[a]	Gene expression: Increase, decrease, no change (method w/ reference gene); methods: RT-qPCR, sqPCR, northern hybridization[f]	Gene expression: when it reaches peak and peak's magnitude (fold change; relative gene expression; times or ratio; unclear = ?)[f]	Protein expression: Increase, decrease, no change (method w/reference); methods: ELISA, WB, RIA, EMSA, IF[f]	Protein expression: When it reaches peak and peak's magnitude (times or ratio; unclear = ?)[f]	Remarks
Santos et al. (2010)	NO	Nitric oxide	MLO-Y4 (n.g./ n.g. (n.g.), n.g., n.g., n.g., 2.75×10^5 cells per slide/ n.g.)	Pulsatile laminar	0min, 5min, 10min, 15min@ 5Hz	0.7±0.3Pa	Custom-made			Increase (Griess, NO_2^-)	Problem in control in the figure	Contradicting reporting between figure (2a) and text.
Santos et al. (2010)	β-catenin	Ctnnb1	MLO-Y4 (n.g./ n.g. (n.g.), n.g., n.g., n.g., 2.75×10^5 cells per slide/ n.g.)	Pulsatile laminar	30min @ 5Hz	0.7±0.3Pa	Custom-made			Increase (ELISA)	1194.8pg/mL; 1194.8/712.8 = 1.7 (ratio-calc)†	
Geoghegan et al. (2019)	Cox-2	Ptgs2	MLO-Y4 (n.g./ n.g. (n.g.), n.g., n.g., n.g., 200000 cells per slide/ n.g.)	Oscillatory laminar	1h @ 0.5Hz	1Pa	Custom-made	Increase (RT-qPCR, Rpl13a)	2.7 (rel.)†			
Geoghegan et al. (2019)	OPG	Tnfrsf11b	MLO-Y4 (n.g./ n.g. (n.g.), n.g., n.g., n.g., 200000 cells per slide/ n.g.)	Oscillatory laminar	1h @ 0.5Hz	1Pa	Custom-made	Increase (RT-qPCR, Rpl13a)	1.6 (rel.)†			
Geoghegan et al. (2019)	Rankl	Tnfsf11	MLO-Y4 (n.g./ n.g. (n.g.), n.g., n.g., n.g., 200000 cells per slide/ n.g.)	Oscillatory laminar	1h @ 0.5Hz	1Pa	Custom-made	Decrease (RT-qPCR, Rpl13a)	0.88 (rel.)†			
Geoghegan et al. (2019)	RANKL / OPG	ratio (RANKL/OPG)	MLO-Y4 (n.g./ n.g. (n.g.), n.g., n.g., n.g., 200000 cells per slide/ n.g.)	Oscillatory laminar	1h @ 0.5Hz	1Pa	Custom-made	Decrease (RT-qPCR, Rpl13a)	0.50 (ratio)†			
Lu et al. (2012b)	Ca^{2+}	Calcium	MLO-Y4 (n.g./ n.g. (n.g.), n.g., n.g., n.g., n.g./ 70-80%)	Steady laminar	9min (sampling at 60s 120s, 240s, 360s, 480s, 600s during FSS) @ 1Hz	20dyn/cm^2	Custom-made			Fluctuated increase (Fura-2 AM microscopy)	steady; laminar (80s): 4.9 ([Ca^{2+}] intensity of baseline ratio))†	
Lu et al. (2012b)	Ca^{2+}	Calcium	MLO-Y4 (n.g./ n.g. (n.g.), n.g., n.g., n.g., n.g./ 70-80%)	Oscillatory laminar	9min (sampling at 60s, 120s, 240s, 360s, 480s, 600s during FSS) @ 1Hz	20dyn/cm^2	Custom-made			Fluctuated increase (Fura-2 AM microscopy)	oscillatory laminar (87s): 2.65 ([Ca^{2+}] intensity of baseline ratio)†; Note: comparison between steady and oscillatory fluid flow	
Zhang et al. (2015)	Ca^{2+}	Calcium	MLO-Y4 (n.g./ n.g. (n.g.), n.g., n.g., n.g., 100000 and 150000 cells per slide/ 70-80%)	Oscillatory laminar	3min-2h @ 1Hz	1Pa	Custom-made			Fluctuated increase (Fluorescence microscopy, Fura-2 AM)		
Zhang et al. (2015)	COX-2	Ptgs2	MLO-Y4 (n.g./ n.g. (n.g.), n.g., n.g., n.g., 100000 and 150000 cells per slide/ 70-80%)	Oscillatory laminar	3min-2h @ 1Hz	1Pa	Custom-made	Increase (RT-qPCR, 18S)	2h: 3.9 (rel.)†			
Zhang et al. (2015)	PGE2	PGE2	MLO-Y4 (n.g./ n.g. (n.g.), n.g., n.g., n.g., 100000 and 150000 cells per slide/ 70-80%)	Oscillatory laminar	3min-2h @ 1Hz	1Pa	Custom-made			Increase (ELISA)†	2h: 4.2 (ratio)†	Note: interesting figure combining release and mRNA expression
Zhang et al. (2015)	RANKL / OPG	ratio (RANKL/OPG)	MLO-Y4 (n.g./ n.g. (n.g.), n.g., n.g., n.g., 100000 and 150000 cells per slide/ 70-80%)	Oscillatory laminar	3min-2h @ 1Hz	1Pa	Custom-made	Decrease (RT-qPCR, 18S)	2h: 0.69/1.3 = 0.5 (ratio-calc)†			
Govey et al. (2015)	Cxcl1	Cxcl1	MLO-Y4 (n.g./ n.g. (n.g.), n.g., n.g., n.g., 1.35×10^4/ ~60%)	Oscillatory laminar	2h @ 1Hz (2h, 8h, 24h post FSS incubation)	1Pa (10dyn/cm^2)	n.g.	Increase (RT-qPCR, β-actin)	2h post FSS: 7.60 (rel.)			
Govey et al. (2015)	Cxcl2	Cxcl2	MLO-Y4 (n.g./ n.g. (n.g.), n.g., n.g., n.g., 1.35×10^4/ ~60%)	Oscillatory laminar	2h @ 1Hz (2h, 8h, 24h post FSS incubation)	1Pa (10dyn/cm^2)	n.g.	Increase (RT-qPCR, β-actin)	2h post FSS: 8.55 (rel.)			
Govey et al. (2015)	RNA-Seq		MLO-Y4 (n.g./ n.g. (n.g.), n.g., n.g., n.g., 1.35×10^4/ ~60%)	Oscillatory laminar	2h @ 1Hz (2h, 8h, 24h post FSS incubation)	1Pa (10dyn/cm^2)	n.g.	RNAseq: Illumina sequencing w/ HiSeq 2500	GEO:GSE70667			Includes reanalysis of microarray data published in Govey et al 2014!
Kalogeropoulos et al. (2010)	Zic1	Zic1	MLO-Y4 (n.g./ n.g. (n.g.), n.g., n.g., n.g., n.g./ n.g.)	Oscillatory laminar	(1h, 6h, 12h)/n.g.	7.5dyn/cm^2	Orbital shaker			12h: increase (WB, GAPDH)	No quantitative information given.	Fluorescence microscopy: increase fluorescence intensity (especially 12h)
Bakker et al. (2014)	Cox2	Ptgs2	MLO-Y4 (n.g./ n.g. (n.g.), n.g., n.g., n.g., n.g./ n.g.)	Pulsatile laminar	60min / 5Hz	0.7±0.3Pa	Custom-made	Increase (RT-qPCR, GAPDH)	0.1/0.04 = 2.5 (ratio-calc)†			

[a] Entry provided as reported in the given study.
[b] Human genes were confirmed with the HUGO Gene Nomenclature Committee (HGNC; URL: https://www.genenames.org); mouse genes were confirmed with the Mouse Genome Informatics (MGI; URL: https://www.informatics.jax.org/genes.shtml) after checking the specificity of primers with Primer-BLAST.
[c] Sex of donors: "M" – male, "F" – female; Tooth type: "PM" – premolar, "M" – molar; dig. Indicate isolation by cell digestion; Exp. indicate isolation by cell explant; Cell density: given in cells/cm^2 if not otherwise mentioned.
[d] Flow type deduced from the description of the FSS apparatus given by the authors.
f RT-qPCR (reverse-transcriptase quantitative polymerase chain reaction); sqPCR (semi-quantitative polymerase chain reaction); ELISA (enzyme-linked immuno absorbent assay); WB (western blotting); RIA (radioimmunoassay); EMSA (electromobility shift assay); IF (immunofluorescence)

rel.: indicate relative gene expression. Is entitled to percentages or gene expression ratios normalized to control, and not calculated by ΔΔCT.
FC: indicate fold change. When Author mentions the use of ΔΔCT or the method according to Livak & Schmittken (2004) in calculating FC.
n.g.: not given. For information not given by study-authors.
† Information derived from figures using Engauge Digitizer.
* Indicate manual calculations by measuring the graphs, without using the Engauge Digitizer.
ratio-calc: indicate manual calculation by dividing intervention/control = result (ratio-calc)
ratio: indicate ratios given by study-authors such as normalization to control in case of small molecules data or in case of gene expression ratios, e.g. ratio of RANKL/OPG or Bcl-2/Bax.

Reference	Gene or analyte[a]	Official gene symbol or abbreviation[b]	Cell type (age/ number and sex of donor (health status), tooth type, isolation method, passages used, cell density/confluency)[a,c]	Flow type (Steady laminar, Pulsatile laminar, or Oscillatory laminar)[a,d]	FSS-duration and frequency[a]	FSS-magnitude[a]	FSS apparatus[a]	Gene expression: Increase, decrease, no change (method w/ reference gene); methods: RT-qPCR, sqPCR, northern hybridization[f]	Gene expression: when it reaches peak and peak's magnitude (fold change; relative gene expression; times or ratio; unclear = ?)[f]	Protein expression: Increase, decrease, no change (method w/reference); methods: ELISA, WB, RIA, EMSA, IF[f]	Protein expression: When it reaches peak and peak's magnitude (times or ratio; unclear = ?)[f]	Remarks
Bakker et al. (2014)	IL-6	Il6	MLO-Y4 (n.g./ n.g. (n.g.), n.g., n.g., n.g., n.g./ n.g.)	Pulsatile laminar	60min @ 5H	0.7±0.3Pa	Custom-made	Increase (RT-qPCR, GAPDH)	60min: 242.9/62.6 = 3.9 (ratio-calc)† (Increase by 3.8-fold compared to control)	Increase (ELISA)	60min: 0.17ng/2×10^5 cells; 0.17/0.08 = 2.1 (ratio-calc)† (increase by 2-fold compared to control)	
Bakker et al. (2014)	NO	Nitric oxide	MLO-Y4 (n.g./ n.g. (n.g.), n.g., n.g., n.g., n.g./ n.g.)	Pulsatile laminar	60min (sampling 5min and 60min during FSS) / 5Hz	0.7±0.3Pa	Custom-made			Increase (Griess, NO_2^-)	5min: 1.8-fold increase 60min: 4.5-fold increase	
Xia et al. (2010)	Cx43	Gja1	MLO-Y4 (n.g./ n.g. (n.g.), n.g., n.g., n.g., n.g./ n.g.)	Steady laminar	0h, 0.5h, 2h, 8h, 16h / n.g.	16dyn/cm^2	Custom-made			Increase (WB, β-actin)	16h: 2.6 (ratio)†	
Xia et al. (2010)	p-GSK3α/β	Gsk3a; Gsk3b	MLO-Y4 (n.g./ n.g. (n.g.), n.g., n.g., n.g., n.g./ n.g.)	Steady laminar	0h, 0.5h, 2h, 8h, 16h / n.g.	16dyn/cm^2	Custom-made			Increase (WB, GSK3β)	0.5h: 2.6 (ratio)†	
Xia et al. (2010)	p(S^{473})-Akt	Akt1	MLO-Y4 (n.g./ n.g. (n.g.), n.g., n.g., n.g., n.g./ n.g.)	Steady laminar	0h, 0.5h, 2h, 8h, 16h / n.g.	16dyn/cm^2	Custom-made			Increase (WB, AKT)	0.5h: 16.9 (ratio)†	
Xia et al. (2010)	p(T^{308})-Akt	Akt1	MLO-Y4 (n.g./ n.g. (n.g.), n.g., n.g., n.g., n.g./ n.g.)	Steady laminar	0h, 0.5h, 2h, 8h, 16h / n.g.	16dyn/cm^2	Custom-made			Increase (WB, AKT)	0.5h: 15.4 (ratio)†	
Xia et al. (2010)	β-catenin	Ctnnb1	MLO-Y4 (n.g./ n.g. (n.g.), n.g., n.g., n.g., n.g./ n.g.)	Steady laminar	0h, 0.5h, 2h, 8h, 16h / n.g.	16dyn/cm^2	Custom-made			Increase (WB, β-actin)	0.5h: 2.3 (ratio)†	
Xia et al. (2010)	β-catenin (active)	Ctnnb1	MLO-Y4 (n.g./ n.g. (n.g.), n.g., n.g., n.g., n.g./ n.g.)	Steady laminar	0h, 0.5h, 2h, 8h, 16h / n.g.	16dyn/cm^2	Custom-made			Increase (WB, β-actin)	0.5h: 2.8 (ratio)†	
Thi et al. (2010)	NRP-1	Nrp1	MLO-Y4 (n.g./ n.g. (n.g.), n.g., n.g., n.g., n.g./ n.g.)	Pulsatile laminar	5h @ 1Hz	5dyn/cm^2	Cytodyne parallel plate flow chamber			Increase (WB, GAPDH)	No quantitative information given.	
Thi et al. (2010)	VEGF	Vegfa	MLO-Y4 (n.g./ n.g. (n.g.), n.g., n.g., n.g., n.g./ n.g.)	Pulsatile laminar	5h @ 1Hz	5dyn/cm^2	Cytodyne parallel plate flow chamber			Increase (ELISA); Increase (WB, GAPDH)	ELISA: 20.4pg/µg protein; 20.4/1.1 = 18.5 (ratio/calc)† WB: no quantitative information given	
Thi et al. (2010)	VEGFR-1	Flt1	MLO-Y4 (n.g./ n.g. (n.g.), n.g., n.g., n.g., n.g./ n.g.)	Pulsatile laminar	5h @ 1Hz	5dyn/cm^2	Cytodyne parallel plate flow chamber			Increase (WB, GAPDH)	No quantitative information given.	
Thi et al. (2010)	VEGFR-2	Kdr	MLO-Y4 (n.g./ n.g. (n.g.), n.g., n.g., n.g., n.g./ n.g.)	Pulsatile laminar	5h @ 1Hz	5dyn/cm^2	Cytodyne parallel plate flow chamber			Increase (WB, GAPDH)	No quantitative information given.	
Maycas et al. (2017)	ERK1/2 (nuclear)	Mapk3; Mapk1	MLO-Y4 (n.g./ n.g. (n.g.), n.g., n.g., n.g., 2×10^4 cells per cm^2/ n.g.)	Pulsatile laminar	10min (sampling 18h post FSS) / 8Hz	10dyn/cm^2	Flexcell® Streamer®			Increase (WB, Lamin B1)	1.7 (ratio)†	
Maycas et al. (2017)	GM-CSF	Csf2	MLO-Y4 (n.g./ n.g. (n.g.), n.g., n.g., n.g., 2×10^4 cells per cm^2/ n.g.)	Pulsatile laminar	10min (sampling 18h post FSS) / 8Hz	10dyn/cm^2	Flexcell® Streamer®			Decrease (ELISA)	683.8pg/mL; 683.8/3525.6 = 0.19 (ratio-calc)†	
Maycas et al. (2017)	IL-6	Il6	MLO-Y4 (n.g./ n.g. (n.g.), n.g., n.g., n.g., 2×10^4 cells per cm^2/ n.g.)	Pulsatile laminar	10min (sampling 18h post FSS) / 8Hz	10dyn/cm^2	Flexcell® Streamer®			Decrease (ELISA)	543pg/mL; 543/3457 = 0.16 (ratio-calc)†	
Maycas et al. (2017)	MCP-1	Ccl2	MLO-Y4 (n.g./ n.g. (n.g.), n.g., n.g., n.g., 2×10^4 cells per cm^2/ n.g.)	Pulsatile laminar	10min (sampling 18h post FSS) / 8Hz	10dyn/cm^2	Flexcell® Streamer®			Decrease (ELISA)	7.58E+05pg/mL; 7.58E+05/2.08E+06 = 0.36 (ratio-calc)†	
Maycas et al. (2017)	MIP-1β	Ccl4	MLO-Y4 (n.g./ n.g. (n.g.), n.g., n.g., n.g., 2×10^4 cells per cm^2/ n.g.)	Pulsatile laminar	10min (sampling 18h post FSS) / 8Hz	10dyn/cm^2	Flexcell® Streamer®			Decrease (ELISA)	52.6pg/mL; 52.6/98.6 = 0.53 (ratio-calc)†	
Maycas et al. (2017)	MIP1α	Ccl3	MLO-Y4 (n.g./ n.g. (n.g.), n.g., n.g., n.g., 2×10^4 cells per cm^2/ n.g.)	Pulsatile laminar	10min (sampling 18h post FSS) / 8Hz	10dyn/cm^2	Flexcell® Streamer®			Decrease (ELISA)	65.4pg/mL; 65.4/93.2 = 0.7 (ratio-calc)†	
Maycas et al. (2017)	p(S^9)-GSK3β / GSK3B	Gsk3b	MLO-Y4 (n.g./ n.g. (n.g.), n.g., n.g., n.g., 2×10^4 cells per cm^2/ n.g.)	Pulsatile laminar	10min (sampling 18h post FSS) / 8Hz	10dyn/cm^2	Flexcell® Streamer®			Increase (WB, GSK3B)	1.0/0.028 = 35.7 (ratio-calc)†	
Maycas et al. (2017)	p(Thr^{202}/Tyr^{204})-ERK1/2	Mapk3; Mapk1	MLO-Y4 (n.g./ n.g. (n.g.), n.g., n.g., n.g., 2×10^4 cells per cm^2/ n.g.)	Pulsatile laminar	10min (sampling 18h post FSS) / 8Hz	10dyn/cm^2	Flexcell® Streamer®			Increase (WB, ERK)	2.2 (ratio)†	

[a] Entry provided as reported in the given study.
[b] Human genes were confirmed with the HUGO Gene Nomenclature Committee (HGNC; URL: https://www.genenames.org); mouse genes were confirmed with the Mouse Genome Informatics (MGI; URL: https://www.informatics.jax.org/genes.shtml) after checking the specificity of primers with Primer-BLAST.
[c] Sex of donors: "M" – male, "F" – female; Tooth type: "PM" – premolar, "M" – molar; dig. Indicate isolation by cell digestion; Exp. indicate isolation by cell explant; Cell density: given in cells/cm^2 if not otherwise mentioned.
[d] Flow type deduced from the description of the FSS apparatus given by the authors.
[f] RT-qPCR (reverse-transcriptase quantitative polymerase chain reaction); sqPCR (semi-quantitative polymerase chain reaction); ELISA (enzyme-linked immuno absorbent assay); WB (western blotting); RIA (radioimmunoassay); EMSA (electromobility shift assay); IF (immunofluorescence)

rel.: indicate relative gene expression. Is entitled to percentages or gene expression ratios normalized to control, and not calculated by ΔΔCT.
FC: indicate fold change. When Author mentions the use of ΔΔCT or the method according to Livak & Schmittken (2004) in calculating FC.
n.g.: not given. For information not given by study-authors.
† Information derived from figures using Engauge Digitizer.
* Indicate manual calculations by measuring the graphs, without using the Engauge Digitizer.
ratio-calc: indicate manual calculation by dividing intervention/control = result (ratio-calc)
ratio: indicate ratios given by study-authors such as normalization to control in case of small molecules data or in case of gene expression ratios, e.g. ratio of RANKL/OPG or Bcl-2/Bax.

Reference	Gene or analyte[a]	Official gene symbol or abbreviation[b]	Cell type (age/ number and sex of donor (health status), tooth type, isolation method, passages used, cell density/confluency)[a,c]	Flow type (Steady laminar, Pulsatile laminar, or Oscillatory laminar)[a,d]	FSS-duration and frequency[a]	FSS-magnitude[a]	FSS apparatus[a]	Gene expression: Increase, decrease, no change (method w/ reference gene); methods: RT-qPCR, sqPCR, northern hybridization[f]	Gene expression: when it reaches peak and peak's magnitude (fold change; relative gene expression; times or ratio; unclear = ?)[f]	Protein expression: Increase, decrease, no change (method w/reference); methods: ELISA, WB, RIA, EMSA, IF[f]	Protein expression: When it reaches peak and peak's magnitude (times or ratio; unclear = ?)[f]	Remarks
Maycas et al. (2017)	RANKL	Tnfsf11	MLO-Y4 (n.g./ n.g. (n.g.), n.g., n.g., n.g., 2×10^4 cells per cm^2/ n.g.)	Pulsatile laminar	10min (sampling 18h post FSS) / 8Hz	10dyn/cm^2	Flexcell® Streamer®			Decrease (ELISA)	6819.1pg/mL; 6819.1/8793 = 0.77 (ratio-calc)†	
Maycas et al. (2017)	RANTES	Ccl5	MLO-Y4 (n.g./ n.g. (n.g.), n.g., n.g., n.g., 2×10^4 cells per cm^2/n.g.)	Pulsatile laminar	10min (sampling 18h post FSS) / 8Hz	10dyn/cm^2	Flexcell® Streamer®			Decrease (ELISA)	309.7pg/mL; 309.7/777.7 = 0.39 (ratio-calc)†	
Maycas et al. (2017)	VEGF	Vegfa	MLO-Y4 (n.g./ n.g. (n.g.), n.g., n.g., n.g., 2×10^4 cells per cm^2/n.g.)	Pulsatile laminar	10min (sampling 18h post FSS) / 8Hz	10dyn/cm^2	Flexcell® Streamer®			Decrease (ELISA)	635.6pg/mL; 635.6/3156.8 = 0.2 (ratio-calc)†	
Maycas et al. (2017)	β-catenin (nuclear)	Ctnnb1	MLO-Y4 (n.g./ n.g. (n.g.), n.g., n.g., n.g., 2×10^4 cells per cm^2/n.g.)	Pulsatile laminar	10min (sampling 18h post FSS) / 8Hz	10dyn/cm^2	Flexcell® Streamer®			Increase (WB, Lamin B1)	1.8 (ratio)†	
Maycas et al. (2017)	β-catenin (total)	Ctnnb1	MLO-Y4 (n.g./ n.g. (n.g.), n.g., n.g., n.g., 2×10^4 cells per cm^2/n.g.)	Pulsatile laminar	10min (sampling 18h post FSS) / 8Hz	10dyn/cm^2	Flexcell® Streamer®			Increase (WB, α-tubulin)	4.7 (ratio)†	
Wang et al. (2019)	Ca^{2+}	Calcium	MLO-Y4 (n.g./ n.g. (n.g.), n.g., n.g., n.g., n.g./n.g.)	Oscillatory laminar	9min (sampling at 60s, 120s, 240s, 360s, 480s, 600s during FSS) @ 0.25Hz	2Pa	Custom-made			Fluctuated increase and decrease (fluorescence microscopy, Suramin)	High density:<br>Increase @ 119s: 1.77 ([Ca^{2+}] fold of baseline ratio)*<br>Decrease @ 238s: 0.95 ([Ca^{2+}] fold of baseline ratio)*	
Wang et al. (2019)	Ca^{2+}	Calcium	MLO-Y4 (n.g./ n.g. (n.g.), n.g., n.g., n.g., n.g./n.g.)	Oscillatory laminar	9min (sampling at 60s, 120s, 240s, 360s, 480s, 600s during FSS) @ 0.25Hz	2Pa	Custom-made			Fluctuated increase and decrease (fluorescence microscopy, Suramin)	Low density:<br>Increase @ 70s: 1.65 ([Ca^{2+}] fold of baseline ratio)*<br>Decrease @ 355s: 0.89 ([Ca^{2+}] fold of baseline ratio) *	
Shah et al. (2017)	Ca^{2+}	Calcium	MLO-Y4 (n.g./ n.g. (n.g.), n.g., n.g., n.g., 15×10^3 or 25×10^3 cells per slide/n.g.)	n.g.	20s, 30min, 24h/n.g.	16dyn/cm^2	n.g.			Increase (intracellular calcium Fluorescence microscopy, Fluo-4AM)		
Shah et al. (2017)	CX43	Gja1	MLO-Y4 (n.g./ n.g. (n.g.), n.g., n.g., n.g., 15×10^3 or 25×10^3 cells per slide/n.g.)	n.g.	20s, 30min, 24h/n.g.	16dyn/cm^2	n.g.	30min: increase (RT-qPCR, GAPDH)<br>24h: increase (RT-qPCR, GAPDH)	30min: 1.8 (FC)†<br>24h: 2.2 (FC)†			
Shah et al. (2017)	Dkk-1	Dkk1	MLO-Y4 (n.g./ n.g. (n.g.), n.g., n.g., n.g., 15×10^3 or 25×10^3 cells per slide/n.g.)	n.g.	20s, 30min, 24h/n.g.	16dyn/cm^2	n.g.	30min: decrease (RT-qPCR, GAPDH)<br>24h: decrease (RT-qPCR, GAPDH)	30min: 0.9 (FC)†<br>24h: 0.76 (FC)†			
Shah et al. (2017)	Gp38	Pdpn	MLO-Y4 (n.g./ n.g. (n.g.), n.g., n.g., n.g., 15×10^3 or 25×10^3 cells per slide/n.g.)	n.g.	20s, 30min, 24h/n.g.	16dyn/cm^2	n.g.	30min: increase (RT-qPCR, GAPDH)<br>24h: increase (RT-qPCR, GAPDH)	30min: 4.5 (FC)†<br>24h: 3.7 (FC)†			
Shah et al. (2017)	RANKL	Tnfsf11	MLO-Y4 (n.g./ n.g. (n.g.), n.g., n.g., n.g., 15×10^3 or 25×10^3 cells per slide/n.g.)	n.g.	20s, 30min, 24h/n.g.	16dyn/cm^2	n.g.	30min: increase (RT-qPCR, GAPDH)<br>24h: increase (RT-qPCR, GAPDH)	30min: 6.6 (FC)†<br>24h: 2.3 (FC)†			
Thi et al. (2003)	Cx43	Gja1	MLO-Y4 (n.g./ n.g. (n.g.), n.g., n.g., n.g., n.g./n.g.)	Steady laminar	1h, 2h, or 3 h / n.g.	5dyn/cm^2, 20dyn/cm^2	Parallel-plate flow chamber (Cytodyne, La Jolla, CA)	5dyn/cm^2 @ 3h: increase (Northern blot/18S)<br>20dyn/cm^2 @ 1h: increase (Northern, 18S)	5dyn/cm^2 @ 3h: 1.5 (rel.)*<br>20dyn/cm^2 @ 1h: 1.1 (rel.)*			

[a] Entry provided as reported in the given study.
[b] Human genes were confirmed with the HUGO Gene Nomenclature Committee (HGNC; URL: https://www.genenames.org); mouse genes were confirmed with the Mouse Genome Informatics (MGI; URL: https://www.informatics.jax.org/genes.shtml) after checking the specificity of primers with Primer-BLAST.
[c] Sex of donors: "M" – male, "F" – female; Tooth type: "PM" – premolar, "M" – molar; dig. Indicate isolation by cell digestion; Exp. indicate isolation by cell explant; Cell density: given in cells/cm^2 if not otherwise mentioned.
[d] Flow type deduced from the description of the FSS apparatus given by the authors.
[f] RT-qPCR (reverse-transcriptase quantitative polymerase chain reaction); sqPCR (semi-quantitative polymerase chain reaction); ELISA (enzyme-linked immuno absorbent assay); WB (western blotting); RIA (radioimmunoassay); EMSA (electromobility shift assay); IF (immunofluorescence)

rel.: indicate relative gene expression. Is entitled to percentages or gene expression ratios normalized to control, and not calculated by ΔΔCT.
FC: indicate fold change. When Author mentions the use of ΔΔCT or the method according to Livak & Schmittken (2004) in calculating FC.
n.g.: not given. For information not given by study-authors.
† Information derived from figures using Engauge Digitizer.
* Indicate manual calculations by measuring the graphs, without using the Engauge Digitizer.
ratio-calc: indicate manual calculation by dividing intervention/control = result (ratio-calc)
ratio: indicate ratios given by study-authors such as normalization to control in case of small molecules data or in case of gene expression ratios, e.g. ratio of RANKL/OPG or Bcl-2/Bax.

Reference	Gene or analyte[a]	Official gene symbol or abbreviation[b]	Cell type (age/ number and sex of donor (health status), tooth type, isolation method, passages used, cell density/confluency)[a,c]	Flow type (Steady laminar, Pulsatile laminar, or Oscillatory laminar)[a,d]	FSS-duration and frequency[a]	FSS-magnitude[a]	FSS apparatus[a]	Gene expression: Increase, decrease, no change (method w/ reference gene); methods: RT-qPCR, sqPCR, northern hybridization[f]	Gene expression: when it reaches peak and peak's magnitude (fold change; relative gene expression; times or ratio; unclear = ?)[f]	Protein expression: Increase, decrease, no change (method w/reference); methods: ELISA, WB, RIA, EMSA, IF[f]	Protein expression: When it reaches peak and peak's magnitude (times or ratio; unclear = ?)[f]	Remarks
Thi et al. (2003)	Cx45	Gjc1	MLO-Y4 (n.g./ n.g. (n.g.), n.g., n.g., n.g., n.g./n.g.)	Steady laminar	1h, 2h, or 3 h / n.g.	5dyn/cm^2, 20dyn/cm^2	Parallel-plate flow chamber (Cytodyne, La Jolla, CA)	5dyn/cm^2 @ 1-3h no change (Northern blot/18S) 20dyn/cm^2 @ 3h: increase (Northern, 18S)	20dyn/cm^2 @ 3h: 1.4 (rel.)*	cytosolic protein: increase (WB, GAPDH)	5dyn/cm^2 @ 1h: 0.7 (ratio)* 20dyn/cm^2 @ 3h: 1.6 (ratio)*	
Thi et al. (2003)	ZO-1	Tjp1	MLO-Y4 (n.g./ n.g. (n.g.), n.g., n.g., n.g., n.g./n.g.)	Steady laminar	1h, 2h, or 3 h / n.g.	5dyn/cm^2, 20dyn/cm^2	Parallel-plate flow chamber (Cytodyne, La Jolla, CA)	Decrease (sqPCR, 18S)	5dyn/cm^2 @ 3h: 0.11 (rel.)† 20dyn/cm^2 @ 3h: 0.026 (rel.)†	Membrane bound: decrease (WB, β-actin)	5dyn/cm^2 @ 1h: 0.5 (ratio)* 20dyn/cm^2 @ 3h: 0.5 (ratio)*	
Reilly et al. (2003)	Ca^{2+}	Calcium	MLO-Y4 (n.g./ n.g. (n.g.), n.g., n.g., n.g., 200000 cell per slide/ 80%)	Oscillatory laminar	1h/n.g.	2Pa, 1Pa	Custom-made			Fluctuated increase (fluorescence microscopy, Fura-2 AM)		
Reilly et al. (2003)	PGE2	PGE2	MLO-Y4 (n.g./ n.g. (n.g.), n.g., n.g., n.g., 200000 cell per slide/ 80%)	Oscillatory laminar	1h/n.g.	2Pa, 1Pa	Custom-made			Increase (ELISA)	4.4 (PGE2/µg DNA); 4.4 (ratio)†	
Lu et al. (2012a)	Ca^{2+}	Calcium	MLO-Y4 (n.g./ n.g. (n.g.), n.g., n.g., n.g., 1.0×10^4 cell per cm^2 slide area/ n.g.)	Steady laminar (magnetic gear pump)	9 min /n.g.	0.5Pa, 1Pa, 2Pa, 4Pa	Custom-made			Fluctuated increase (fluorescence microscopy, Fura-2 AM)		
Xu et al. (2012)	alpha tubulin	Tuba1a	MLO-Y4 (n.g./ n.g. (n.g.), n.g., n.g., n.g., 2.2×10^6 per slide/ n.g.)	Oscillatory laminar	2h @ 1Hz	1Pa (10dyn/cm^2)	Custom-made	Increase (RT-qPCR, 18S)	2h: 4.05(FC)†	Increase (WB, GAPDH)	2h: 1.7 (ratio)†	
Xu et al. (2012)	beta-actin	Actb	MLO-Y4 (n.g./ n.g. (n.g.), n.g., n.g., n.g., 2.2×10^6 per slide/ n.g.)	Oscillatory laminar	2h @ 1Hz	1Pa (10dyn/cm^2)	Custom-made	Increase (RT-qPCR, 18S)	2h: 1.98 (FC)†	Increase (WB, GAPDH)	2h: 1.3 (ratio)†	
Xu et al. (2012)	CD44	Cd44	MLO-Y4 (n.g./ n.g. (n.g.), n.g., n.g., n.g., 2.2×10^6 per slide/ n.g.)	Oscillatory laminar	2h @ 1Hz	1Pa (10dyn/cm^2)	Custom-made	Increase (RT-qPCR, 18S)	2h: 2.0(FC)†			
Xu et al. (2012)	Cox-2	Ptgs2	MLO-Y4 (n.g./ n.g. (n.g.), n.g., n.g., n.g., 2.2×10^6 per slide/ n.g.)	Oscillatory laminar	2h @ 1Hz	1Pa (10dyn/cm^2)	Custom-made	Increase (RT-qPCR, 18S)	2h: 4.05 (FC)†			
Xu et al. (2012)	E11 (gp38)	Pdpn	MLO-Y4 (n.g./ n.g. (n.g.), n.g., n.g., n.g., 2.2×10^6 per slide/ n.g.)	Oscillatory laminar	2h @ 1Hz	1Pa (10dyn/cm^2)	Custom-made	Increase (RT-qPCR, 18S)	2h: 7.6 (FC)†			Primer-BLAST approved
Xu et al. (2012)	Integrin alpha v	Itgav	MLO-Y4 (n.g./ n.g. (n.g.), n.g., n.g., n.g., 2.2×10^6 per slide/ n.g.)	Oscillatory laminar	2h @ 1Hz	1Pa (10dyn/cm^2)	Custom-made	Increase (RT-qPCR, 18S)	2h: 2.60 (FC)†			
Xu et al. (2012)	Integrin beta3	Itgb3	MLO-Y4 (n.g./ n.g. (n.g.), n.g., n.g., n.g., 2.2×10^6 per slide/ n.g.)	Oscillatory laminar	2h @ 1Hz	1Pa (10dyn/cm^2)	Custom-made	Increase (RT-qPCR, 18S)	2h: 13.64 (FC)†			
Xu et al. (2012)	OPN	Spp1	MLO-Y4 (n.g./ n.g. (n.g.), n.g., n.g., n.g., 2.2×10^6 per slide/ n.g.)	Oscillatory laminar	2h @ 1Hz	1Pa (10dyn/cm^2)	Custom-made	Increase (RT-qPCR, 18S)	2h: 2.65 (FC)†			
Xu et al. (2012)	Runx2	Runx2	MLO-Y4 (n.g./ n.g. (n.g.), n.g., n.g., n.g., 2.2×10^6 per slide/ n.g.)	Oscillatory laminar	2h @ 1Hz	1Pa (10dyn/cm^2)	Custom-made	Increase (RT-qPCR, 18S)	2h: 6.9(FC)†			
Xu et al. (2012)	vinculin	Vcl	MLO-Y4 (n.g./ n.g. (n.g.), n.g., n.g., n.g., 2.2×10^6 per slide/ n.g.)	Oscillatory laminar	2h @ 1Hz	1Pa (10dyn/cm^2)	Custom-made	Increase (RT-qPCR, 18S)	2h: 2.59 (FC)†	Increase (WB, GAPDH)	2h: 1.7 (ratio)†	
Jing et al. (2013)	Ca++	Calcium	MLO-Y4 (n.g./ n.g. (n.g.), n.g., n.g., n.g., n.g./n.g.)	Steady laminar (magnetic gear pump)	10min period/n.g.	0.5Pa, 1Pa, 2Pa, 4Pa	n.g.			Fluctuated increase (fluorescence microscopy, Fura-2 AM)		
Govey et al. (2014)	ARRAY / LC-MS		MLO-Y4 (n.g./ n.g. (n.g.), n.g., n.g., n.g., 1.35×10^4 cells per cm^2/ 60%)	Oscillatory laminar	2h @ 1Hz (2h, 8h, or 24 h post FSS)	1Pa (10dyn/cm2)	Custom-made	Mouse Genome 430A 2.0 GeneChips (Affymetrix)	GEO:GSE42874		Proteomics data submitted to http://omics.pnl.gov	Re-analysed together with RNAseq data in Govey et al 2015.
Kulkarni et al. (2012b)	c-fos	Fos	MLO-Y4 (n.g./ n.g. (n.g.), n.g., n.g., n.g., 2×10^5/ n.g.)	Pulsatile laminar	1h @ 5Hz	0.7Pa (pulse amplitude of 0.3Pa, peak stress rate of 8.4Pa/s)	Custom-made	Increase (RT-qPCR, GAPDH)	3.5/2.5 = 1.4 (ratio-calc)†			
Kulkarni et al. (2012b)	c-jun	Jun	MLO-Y4 (n.g./ n.g. (n.g.), n.g., n.g., n.g., 2×10^5/ n.g.)	Pulsatile laminar	1h @ 5Hz	0.7Pa (pulse amplitude of 0.3Pa, peak stress rate of 8.4Pa/s)	Custom-made	Increase (RT-qPCR, GAPDH)	5.1/4.5 = 1.13 (ratio-calc)†			

[a] Entry provided as reported in the given study.
[b] Human genes were confirmed with the HUGO Gene Nomenclature Committee (HGNC; URL: https://www.genenames.org); mouse genes were confirmed with the Mouse Genome Informatics (MGI; URL: https://www.informatics.jax.org/genes.shtml) after checking the specificity of primers with Primer-BLAST.
[c] Sex of donors: "M" – male, "F" – female; Tooth type: "PM" – premolar, "M" – molar; dig. Indicate isolation by cell digestion; Exp. indicate isolation by cell explant; Cell density: given in cells/cm^2 if not otherwise mentioned.
[d] Flow type deduced from the description of the FSS apparatus given by the authors.
f RT-qPCR (reverse-transcriptase quantitative polymerase chain reaction); sqPCR (semi-quantitative polymerase chain reaction); ELISA (enzyme-linked immuno absorbent assay); WB (western blotting); RIA (radioimmunoassay); EMSA (electromobility shift assay); IF (immunofluorescence)

rel.: indicate relative gene expression. Is entitled to percentages or gene expression ratios normalized to control, and not calculated by ΔΔCT.
FC: indicate fold change. When Author mentions the use of ΔΔCT or the method according to Livak & Schmittken (2004) in calculating FC.
n.g.: not given. For information not given by study-authors.
† Information derived from figures using Engauge Digitizer.
* Indicate manual calculations by measuring the graphs, without using the Engauge Digitizer.
ratio-calc: indicate manual calculation by dividing intervention/control = result (ratio-calc)
ratio: indicate ratios given by study-authors such as normalization to control in case of small molecules data or in case of gene expression ratios, e.g. ratio of RANKL/OPG or Bcl-2/Bax.

Reference	Gene or analyte[a]	Official gene symbol or abbreviation[b]	Cell type (age/ number and sex of donor (health status), tooth type, isolation method, passages used, cell density/confluency)[a,c]	Flow type (Steady laminar, Pulsatile laminar, or Oscillatory laminar)[a,d]	FSS-duration and frequency[a]	FSS-magnitude[a]	FSS apparatus[a]	Gene expression: Increase, decrease, no change (method w/ reference gene); methods: RT-qPCR, sqPCR, northern hybridization[f]	Gene expression: when it reaches peak and peak's magnitude (fold change; relative gene expression; times or ratio; unclear = ?)[f]	Protein expression: Increase, decrease, no change (method w/reference); methods: ELISA, WB, RIA, EMSA, IF[f]	Protein expression: When it reaches peak and peak's magnitude (times or ratio; unclear = ?)[f]	Remarks
Kulkarni et al. (2012b)	MT1-MMP	Mmp14	MLO-Y4 (n.g./ n.g. (n.g.), n.g., n.g., n.g., 2×10^5/ n.g.)	Pulsatile laminar	1h @ 5Hz	0.7Pa (pulse amplitude of 0.3Pa, peak stress rate of 8.4Pa/s)	Custom-made	Decrease (RT-qPCR, GAPDH)	8.4/18.2 = 0.46 (ratio-calc)†	Decrease (WB, β-actin)	No quantitative information given.	
Kulkarni et al. (2012b)	NO	Nitric oxide	MLO-Y4 (n.g./ n.g. (n.g.), n.g., n.g., n.g., 2×10^5/ n.g.)	Pulsatile laminar	1h @ 5Hz, (samples taken at 5min, 15min, 60min during FSS)	0.7Pa (pulse amplitude of 0.3Pa, peak stress rate of 8.4Pa/s)	Custom-made			Increase (Griess, NO_2^-)	5min: 3.65 (ratio)†	
Genetos et al. (2007)	PGE2	PGE2	MLO-Y4 (n.g./ n.g. (n.g.), n.g., n.g., n.g., 900 cells per cm^2 / n.g.)	Oscillatory laminar	30min @1Hz	20dyn/cm2	Custom-made			Increase (ELISA)	2.2/ 0.93 = 2.4 (ratio-calc)†	
Litzenberger et al. (2010)	Ca^{2+}	Calcium	MLO-Y4 parental cells (n.g./ n.g. (n.g.), n.g., n.g., n.g., n.g./ n.g.)	Oscillatory laminar	2h @1Hz	1Pa	Custom-made			Increase (Florescence microscopy Fura-2 AM)	Increase: 68.95 (precent of cells exhibiting Ca^{2+} flux)†	MLO-Y4 "parental" cells was extracted as mentioned by Author
Litzenberger et al. (2010)	COX-2	Ptgs2	MLO-Y4 parental cells (n.g./ n.g. (n.g.), n.g., n.g., n.g., n.g./ n.g.)	Oscillatory laminar	2h @1Hz	1Pa	Custom-made	Increase (RT-qPCR, 18S)	6.1 (rel.)†			
Litzenberger et al. (2010)	PGE2	PGE2	MLO-Y4 parental cells (n.g./ n.g. (n.g.), n.g., n.g., n.g., n.g./ n.g.)	Oscillatory laminar	2h @1Hz	1Pa	Custom-made			Increase (ELISA)	1.8 (ratio)†	
Litzenberger et al. (2010)	RANKL / OPG	ratio (RANKL/OPG)	MLO-Y4 parental cells (n.g./ n.g. (n.g.), n.g., n.g., n.g., n.g./ n.g.)	Oscillatory laminar	2h @1Hz	1Pa	Custom-made	Decrease (RT-qPCR, 18S)	0.59 (ratio)†			
Li et al. (2012)	COX-2	Ptgs2	MLO-Y4 (n.g./ n.g. (n.g.), n.g., n.g., n.g., n.g./ 70%)	Oscillatory laminar	1h, 2h, 4h @ 0.5Hz, 1Hz, 2Hz, 5Hz	0.5Pa, 1Pa, 2Pa, 5Pa	Custom-made	Duration-related: increase (RT-qPCR, 18S)	2Hz, 0.5Pa@2h: 1.287(rel.)†<br>2Hz, 1.0Pa@2h: 1.5 (rel.)†<br>2Hz, 2.0Pa@2h: 2 (rel.)†<br>2Hz, 5.0Pa@2h: 2.9 (rel.)†<br>0.5Hz, 0.5Pa@4h: 2.7 (rel.)†<br>0.5Hz, 1.0Pa@4h: 2.6 (rel.)†<br>0.5Hz, 2.0Pa@4h: 3.9 (rel.)†<br>0.5Hz, 5.0Pa@4h: 4.4 (rel.)†			
Li et al. (2012)	RANKL / OPG	ratio (RANKL/OPG)	MLO-Y4 (n.g./ n.g. (n.g.), n.g., n.g., n.g., n.g./ 70%)	Oscillatory laminar	1h, 2h, 4h @ 0.5Hz, 1Hz, 2Hz, 5Hz	0.5Pa, 1Pa, 2Pa, 5Pa	Custom-made	Duration-related: decrease (RT-qPCR, 18S)	2Hz, 0.5Pa@2h: 0.42 (ratio)†<br>2Hz, 1.0Pa@1h: 0.85 (ratio)†<br>2Hz, 2.0Pa@2h: 0.46 (ratio)†<br>2Hz, 5.0Pa@1h: 0.44 (ratio)†<br>0.5Hz, 0.5Pa@2h: 1.1 (ratio)†<br>0.5Hz, 1.0Pa@2h: 0.7 (ratio)†<br>0.5Hz, 2.0Pa@2h: 0.3 (ratio)†<br>0.5Hz, 5.0Pa@4h: 0.58 (ratio)†			
Kamel et al. (2010)	PGE2	PGE2	MLO-Y4 (n.g./ n.g. (n.g.), n.g., n.g., P27, 5×10^5 cells per slide/ 70%)	Pulsatile laminar	2h @ 0.5Hz	(2, 4, 8, 16, 24 ±0.6) dyn/cm2	Flexcell® Streamer® Shear Stress Device			Increase (ELISA)	24dyn/cm^2 @120min: 1704.9pg/mL †	

[a] Entry provided as reported in the given study.
[b] Human genes were confirmed with the HUGO Gene Nomenclature Committee (HGNC; URL: https://www.genenames.org); mouse genes were confirmed with the Mouse Genome Informatics (MGI; URL: https://www.informatics.jax.org/genes.shtml) after checking the specificity of primers with Primer-BLAST.
[c] Sex of donors: "M" – male, "F" – female; Tooth type: "PM" – premolar, "M" – molar; dig. Indicate isolation by cell digestion; Exp. indicate isolation by cell explant; Cell density: given in cells/cm^2 if not otherwise mentioned.
[d] Flow type deduced from the description of the FSS apparatus given by the authors.
f RT-qPCR (reverse-transcriptase quantitative polymerase chain reaction); sqPCR (semi-quantitative polymerase chain reaction); ELISA (enzyme-linked immuno absorbent assay); WB (western blotting); RIA (radioimmunoassay); EMSA (electromobility shift assay); IF (immunofluorescence)

rel.: indicate relative gene expression. Is entitled to percentages or gene expression ratios normalized to control, and not calculated by ΔΔCT.
FC: indicate fold change. When Author mentions the use of ΔΔCT or the method according to Livak & Schmittken (2004) in calculating FC.
n.g.: not given. For information not given by study-authors.
† Information derived from figures using Engauge Digitizer.
* Indicate manual calculations by measuring the graphs, without using the Engauge Digitizer.
ratio-calc: indicate manual calculation by dividing intervention/control = result (ratio-calc)
ratio: indicate ratios given by study-authors such as normalization to control in case of small molecules data or in case of gene expression ratios, e.g. ratio of RANKL/OPG or Bcl-2/Bax.

Reference	Gene or analyte[a]	Official gene symbol or abbreviation[b]	Cell type (age/ number and sex of donor (health status), tooth type, isolation method, passages used, cell density/confluency)[a,c]	Flow type (Steady laminar, Pulsatile laminar, or Oscillatory laminar)[a,d]	FSS-duration and frequency[a]	FSS-magnitude[a]	FSS apparatus[a]	Gene expression: Increase, decrease, no change (method w/ reference gene); methods: RT-qPCR, sqPCR, northern hybridization[f]	Gene expression: when it reaches peak and peak's magnitude (fold change; relative gene expression; times or ratio; unclear = ?)[f]	Protein expression: Increase, decrease, no change (method w/reference); methods: ELISA, WB, RIA, EMSA, IF[f]	Protein expression: When it reaches peak and peak's magnitude (times or ratio; unclear = ?)[f]	Remarks
Kamel et al. (2010)	Ptgs2	Ptgs2	MLO-Y4 (n.g./ n.g. (n.g.), n.g., n.g., P27, 5×10^5 cells per slide/ 70%)	Pulsatile laminar	2h @ 0.5Hz	(2, 4, 8, 16, 24 ±0.6) dyn/cm2	Flexcell® Streamer® Shear Stress Device	Increase (RT-qPCR, GAPDH)	16dyn/cm^2: 5.4 (FC)†			
Rath et al. (2010)	Ca^{2+}	Calcium	MLO-Y4 (n.g./ n.g. (n.g.), n.g., n.g., n.g., n.g./ 70–80%)	Pulsatile laminar	n.g./n.g.	2dyn/cm^2, 8dyn/cm^2, 16dyn/cm^2	Parallel plate, live-cell micro-observation chamber (Focht Chamber System 2, Bioptechs Inc., Butler, PA, USA)			Fluorescence microscopy (Fluo-4 AM): increase with increasing shear stress.		
Rath et al. (2010)	NO	Nitric oxide	MLO-Y4 (n.g./ n.g. (n.g.), n.g., n.g., n.g., n.g./ 70–80%)	Pulsatile laminar	n.g./n.g.	2dyn/cm^2, 8dyn/cm^2, 16dyn/cm^2	Parallel plate, live-cell micro-observation chamber (Focht Chamber System 2, Bioptechs Inc., Butler, PA, USA)			DAR-4M fluorescence: increase		
Zhang et al. (2006)	E11 (gp38)	Pdpn	MLO-Y4 (n.g./ n.g. (n.g.), n.g., n.g., n.g., 4×10^5/ n.g.)	Steady laminar	2h (2h and 24h post FSS incubation) / n.g.	4dyn/cm^2, 16dyn/cm^2	Streamer Gold chamber (Flexcell International Corp., Hillsborough, NC)	Increase (Northern blot, GAPDH)	4dyn/cm^2 @ 2h post FSS: 4.3463/2.7408 = 1.6 (ratio-calc)† 16dyn/cm^2 @ 2h post FSS: 5.0/2.87 = 1.7 (ratio-calc)†			
Bakker et al. (2009)	Ca+	Calcium	MLO-Y4 (n.g./ n.g. (n.g.), n.g., n.g., P30-35, 2×10^4 cells per cm^2/ n.g.)	Pulsatile laminar	30min @ 5Hz	0.7±0.3Pa	Custom-made			Fluorescence microscopy (Fluo-4/AM)	Fluctuated increase	
Bakker et al. (2009)	NO	Nitric oxide	MLO-Y4 (n.g./ n.g. (n.g.), n.g., n.g., P30-35, 2×10^4 cells per cm^2/ n.g.)	Pulsatile laminar	30min @ 5Hz	0.7±0.3Pa	Custom-made			Increase (Griess, NO_2^-)	30 min: 16 nmol/µg DNA; 16/2.8 = 5.7 (ratio-calc)†	
Li et al. (2013)	bcl-2 / bax	ratio (Bcl-2/Bax)	MLO-Y4 (n.g./ n.g. (n.g.), n.g., n.g., n.g., n.g./ 75–85%)	Steady laminar (magnetic gear pump)	0h, 0.5h, 1h, 2h, 4h, 8h, 12h, 24h / n.g.	16dyn/cm^2, 30dyn/cm^2	Custom-made parallel-plate flow chamber	16dyn/cm^2: increase (RT-qPCR, GAPDH) 30dyn/cm^2: decrease (RT-qPCR, GAPDH)	16dyn/cm^2 @ 8h: 3.1 (ratio)† 30dyn/cm^2 @ 8h: 0.4 (ratio)†			16dyn/cm^2 (physiological levels), 30dyn/cm^2 (high levels)
Li et al. (2013)	Caspase-3	Casp3	MLO-Y4 (n.g./ n.g. (n.g.), n.g., n.g., n.g., n.g./ 75–85%)	Steady laminar (magnetic gear pump)	0h, 0.5h, 1h, 2h, 4h, 8h, 12h, 24h / n.g.	16dyn/cm^2, 30dyn/cm^2	Custom-made parallel-plate flow chamber	16dyn/cm^2: increase then decrease (RT-qPCR, GAPDH) 30dyn/cm^2: increase (RT-qPCR, GAPDH)	16dyn/cm^2 @ 0.5h: 8.2 (rel.)† 16dyn/cm^2 @ 24h: 0.12 (rel.)† 30dyn/cm^2 @ 24h: 9.8 (rel.)†	16dyn/cm^2: decrease (WB, GAPDH) 30dyn/cm^2: increase (WB, GAPDH)	16dyn/cm^2 @ 24h: 0.2/ 0.42 = 0.5 (ratio-calc)* 30dyn/cm^2 @24h: 0.87/0.25 = 3.48 (ratio-calc)†	16dyn/cm^2 (physiological levels), 30dyn/cm^2 (high levels)
Li et al. (2013)	Cx43	Gja1	MLO-Y4 (n.g./ n.g. (n.g.), n.g., n.g., n.g., n.g./ 75–85%)	Steady laminar (magnetic gear pump)	0h, 0.5h, 1h, 2h, 4h, 8h, 12h, 24h / n.g.	16dyn/cm^2, 30dyn/cm^2	Custom-made parallel-plate flow chamber	16dyn/cm^2: increase (RT-qPCR, GAPDH) 30dyn/cm^2: decrease then increase (RT-qPCR, GAPDH)	16dyn/cm^2 @ 24h: 5.8 (rel.)† 30dyn/cm^2 @ 2h: 0.22 (rel.)† 30dyn/cm^2 @ 24h: 1.4 (rel.)†	16dyn/cm^2: Total and membrane Cxc43: increase (WB, GAPDH) 30dyn/cm^2: Total and membrane Cxc43: decrease (WB, GAPDH)	16dyn/cm^2: Total 24h:0.69/0.37 = 1.9 (ratio-calc)* 16dyn/cm^2: Membrane 24h:0.6/0.24 = 2.5 (ratio-calc)* 30dyn/cm^2: Total 24h:0.32/0.6 = 0.53 (ratio-calc)† 16dyn/cm^2: Membrane 24h:0.30/0.46 = 0.65 (ratio-calc)†	16dyn/cm^2 (physiological levels), 30dyn/cm^2 (high levels) Membrane and Total Cx43 were extracted as per Author
Li et al. (2013)	OPG	Tnfrsf11b	MLO-Y4 (n.g./ n.g. (n.g.), n.g., n.g., n.g., n.g./ 75–85%)	Steady laminar (magnetic gear pump)	0h, 0.5h, 1h, 2h, 4h, 8h, 12h, 24h / n.g.	16dyn/cm^2, 30dyn/cm^2	Custom-made parallel-plate flow chamber	16dyn/cm^2: increase (RT-qPCR, GAPDH) 30dyn/cm^2: decrease with plateau (RT-qPCR, GAPDH)	16dyn/cm^2 @ 24h: 3.6 (rel.)† 30dyn/cm^2 @ 2h: 0.4 (rel.)† 30dyn/cm^2 @ 8h: 0.5 (rel.)† 30dyn/cm^2 @ 24h: 0.4 (rel.)†			16dyn/cm^2 (physiological levels), 30dyn/cm^2 (high levels)

[a] Entry provided as reported in the given study.
[b] Human genes were confirmed with the HUGO Gene Nomenclature Committee (HGNC; URL: https://www.genenames.org); mouse genes were confirmed with the Mouse Genome Informatics (MGI; URL: https://www.informatics.jax.org/genes.shtml) after checking the specificity of primers with Primer-BLAST.
[c] Sex of donors: "M" – male, "F" – female; Tooth type: "PM" – premolar, "M" – molar; dig. Indicate isolation by cell digestion; Exp. indicate isolation by cell explant; Cell density: given in cells/cm^2 if not otherwise mentioned.
[d] Flow type deduced from the description of the FSS apparatus given by the authors.
f RT-qPCR (reverse-transcriptase quantitative polymerase chain reaction); sqPCR (semi-quantitative polymerase chain reaction); ELISA (enzyme-linked immuno absorbent assay); WB (western blotting); RIA (radioimmunoassay); EMSA (electromobility shift assay); IF (immunofluorescence)

rel.: indicate relative gene expression. Is entitled to percentages or gene expression ratios normalized to control, and not calculated by ΔΔCT.
FC: indicate fold change. When Author mentions the use of ΔΔCT or the method according to Livak & Schmittken (2004) in calculating FC.
n.g.: not given. For information not given by study-authors.
† Information derived from figures using Engauge Digitizer.
* Indicate manual calculations by measuring the graphs, without using the Engauge Digitizer.
ratio-calc: indicate manual calculation by dividing intervention/control = result (ratio-calc)
ratio: indicate ratios given by study-authors such as normalization to control in case of small molecules data or in case of gene expression ratios, e.g. ratio of RANKL/OPG or Bcl-2/Bax.

Reference	Gene or analyte[a]	Official gene symbol or abbreviation[b]	Cell type (age/ number and sex of donor (health status), tooth type, isolation method, passages used, cell density/confluency)[a,c]	Flow type (Steady laminar, Pulsatile laminar, or Oscillatory laminar)[a,d]	FSS-duration and frequency[a]	FSS-magnitude[a]	FSS apparatus[a]	Gene expression: Increase, decrease, no change (method w/ reference gene); methods: RT-qPCR, sqPCR, northern hybridization[f]	Gene expression: when it reaches peak and peak's magnitude (fold change; relative gene expression; times or ratio; unclear = ?)[f]	Protein expression: Increase, decrease, no change (method w/reference); methods: ELISA, WB, RIA, EMSA, IF[f]	Protein expression: When it reaches peak and peak's magnitude (times or ratio; unclear = ?)[f]	Remarks
Li et al. (2013)	RANKL	Tnfsf11	MLO-Y4 (n.g./ n.g. (n.g.), n.g., n.g., n.g., n.g./ 75–85%)	Steady laminar (magnetic gear pump)	0h, 0.5h, 1h, 2h, 4h, 8h, 12h, 24h / n.g.	16dyn/cm^2, 30dyn/cm^2	Custom-made parallel-plate flow chamber	16dyn/cm^2: increase (RT-qPCR, GAPDH)<br>30dyn/cm^2: increase (RT-qPCR, GAPDH)	16dyn/cm^2 @ 0.5h: 7.2 (rel.)†<br>30dyn/cm^2 @ 24h: 6.8 (rel.)†	16dyn/cm^2: Increase then decrease (WB, GAPDH)<br>30dyn/cm^2: increase (WB, GAPDH)	16dyn/cm^2 @ 2h: 0.6/0.36 = 1.7 (ratio-calc)*<br>24h: 0.25/0.36 = 0.7 (ratio-calc)*<br>30dyn/cm^2 @24h: 0.91/0.46 = 1.97 (ratio-calc)†	16dyn/cm^2 (physiological levels), 30dyn/cm^2 (high levels)
Li et al. (2013)	Sost	Sost	MLO-Y4 (n.g./ n.g. (n.g.), n.g., n.g., n.g., n.g./ 75–85%)	Steady laminar (magnetic gear pump)	0h, 0.5h, 1h, 2h, 4h, 8h, 12h, 24h / n.g.	16dyn/cm^2, 30dyn/cm^2	Custom-made parallel-plate flow chamber	16dyn/cm^2: decrease with plateau (RT-qPCR, GAPDH)<br>30dyn/cm^2: increase then baseline (RT-qPCR, GAPDH)	16dyn/cm^2 @ 2h: 0.5 (rel.)†<br>16dyn/cm^2 @ 8h: 0.5 (rel.)†<br>16dyn/cm^2 @ 24h: 0.1 (rel.)†<br>30dyn/cm^2 @ 2h: 1.8 (rel.)†<br>30dyn/cm^2 @ 24h: 1 (rel.)†	16dyn/cm^2: 24h: decrease (WB, GAPDH)<br>30dyn/cm^2: increase (WB, GAPDH)	16dyn/cm^2 @ 24h:0.27/0.55 = 0.49 (ratio-calc)*<br>30dyn/cm^2 @24h: 0.44/0.34 = 1.3 (ratio-calc)†	16dyn/cm^2 (physiological levels), 30dyn/cm^2 (high levels)
Middleton et al. (2018)	Ca+	Calcium	MLO-Y4 (n.g./ n.g. (n.g.), n.g., n.g., P40, 1×10^6 cells per mL / 80%)	Steady laminar	2h/n.g.	1Pa	Custom-made, Steady fluid flow microfluidics syringe			Increase (Fluorescence microscopy, Fura-2 AM)		
Cheng et al. (2001)	Cx43	Gja1	MLO-Y4 (n.g./ n.g. (n.g.), n.g., n.g., n.g., n.g./ 75–85%)	Steady laminar	2h (Sampling 0h, 0.5h, 2h, 4h, and 24h post FSS) / 5Hz	16dyn/cm^2	Custom-made			Increase then decrease (WB, n.g.)	4h post FSS: 44.6/29.7 = 1.5 (ratio-calc)†<br>24h post FSS: 6.5/16.5 = 0.4 (ratio-calc)†	
Huang et al. (2017)	Wnt1	Wnt1	Primary mouse osteocytes (5 months/ n.g./M (C57BL/6 mice), n.g., n.g., n.g., n.g./ n.g.)	n.g.	2h (sampling at 0h and 24h post FSS) / n.g.	2dyn/cm^2	n.g.	Increase (RT-qPCR, GAPDH)	0h post FSS: 2 (rel.)†<br>24h post FSS: 1.1 (rel.)†			
Huang et al. (2017)	WNT3a	Wnt3a	Primary mouse osteocytes (5 months/ n.g./M (C57BL/6 mice), n.g., n.g., n.g., n.g./ n.g.)	n.g.	2h (sampling at 0h and 24h post FSS) / n.g.	2dyn/cm^2	n.g.	0h post FSS: increase<br>24h post FSS: decrease (RT-qPCR, GAPDH)	0h post FSS: 2 (rel.)†<br>24h post FSS: 0.92 (rel.)†			
Huang et al. (2017)	Wnt4	Wnt4	Primary mouse osteocytes (5 months/ n.g./M (C57BL/6 mice), n.g., n.g., n.g., n.g./ n.g.)	n.g.	2h (sampling at 0h and 24h post FSS) / n.g.	2dyn/cm^2	n.g.	0h post FSS: decrease<br>24h post FSS: increase (RT-qPCR, GAPDH)	0h post FSS: 0.97 (rel.)†<br>24h post FSS: 1.1 (rel.)†			
Maycas et al. (2015)	p-ERK1/2	Mapk3; Mapk1	MLO-Y4 (n.g./ n.g. (n.g.), n.g., n.g., n.g., n.g./ n.g.)	Pulsatile laminar	10min @ 8Hz	10dyn/cm^2	Pulsatile laminar (Flexcell Streamer)			Increase (WB, ERK)	2.3 (ratio)†	
Maycas et al. (2015)	PTHrP	Pthlh	MLO-Y4 (n.g./ n.g. (n.g.), n.g., n.g., n.g., n.g./ n.g.)	Pulsatile laminar	1h, 6h, 24h @ 8Hz	10dyn/cm^2	Pulsatile laminar (Flexcell Streamer)			Total cell extract: increase with plateau (WB, β-actin)<br>Cell supernatant: increase (WB, B-actin)	total cell extract @ 6h: 5.6 (ratio)†<br>total cell extract @ 24h: 5.4 (ratio)†<br>cell supernatant @ 24h: 2.6 (ratio)†	Cell supernatant WB was extracted as mentioned by Author
Maycas et al. (2015)	β-catenin	Ctnnb1	MLO-Y4 (n.g./ n.g. (n.g.), n.g., n.g., n.g., n.g./ n.g.)	Pulsatile laminar	10min @ 8Hz	10dyn/cm^2	Pulsatile laminar (Flexcell Streamer)			Nuclear β-catenin: increase (WB, lamin B1)<br>Total β-catenin: increase (WB, α-tubulin)	Nuclear β-catenin: 1.8 (ratio)†<br>Total β-catenin: 4.6 (ratio)†	
Haugh et al. (2015)	COX-2	Ptgs2	MLO-Y4 (n.g./ n.g. (n.g.), n.g., n.g., n.g., 200000 cells per slide/ n.g.)	Oscillatory laminar	1h @ 1Hz	1Pa	Custom-made	Increase (RT-qPCR, GAPDH)	6.22 (FC)†			
Haugh et al. (2015)	OPG	Tnfrsf11b	MLO-Y4 (n.g./ n.g. (n.g.), n.g., n.g., n.g., 200000 cells per slide/ n.g.)	Oscillatory laminar	1h @ 1Hz	1Pa	Custom-made	Decrease (RT-qPCR, GAPDH)	0.9 (FC)†			
Haugh et al. (2015)	PGE2	PGE2	MLO-Y4 (n.g./ n.g. (n.g.), n.g., n.g., n.g., 200000 cells per slide/ n.g.)	Oscillatory laminar	1h @ 1Hz	1Pa	Custom-made			Increase (EIA)	10.2pg/ngDNA; 10.2/3.9 = 2.6 (ratio-calc)†	

[a] Entry provided as reported in the given study.
[b] Human genes were confirmed with the HUGO Gene Nomenclature Committee (HGNC; URL: https://www.genenames.org); mouse genes were confirmed with the Mouse Genome Informatics (MGI; URL: https://www.informatics.jax.org/genes.shtml) after checking the specificity of primers with Primer-BLAST.
[c] Sex of donors: "M" – male, "F" – female; Tooth type: "PM" – premolar, "M" – molar; dig. Indicate isolation by cell digestion; Exp. indicate isolation by cell explant; Cell density: given in cells/cm^2 if not otherwise mentioned.
[d] Flow type deduced from the description of the FSS apparatus given by the authors.
[f] RT-qPCR (reverse-transcriptase quantitative polymerase chain reaction); sqPCR (semi-quantitative polymerase chain reaction); ELISA (enzyme-linked immuno absorbent assay); WB (western blotting); RIA (radioimmunoassay); EMSA (electromobility shift assay); IF (immunofluorescence)

rel.: indicate relative gene expression. Is entitled to percentages or gene expression ratios normalized to control, and not calculated by ΔΔCT.
FC: indicate fold change. When Author mentions the use of ΔΔCT or the method according to Livak & Schmittken (2004) in calculating FC.
n.g.: not given. For information not given by study-authors.
† Information derived from figures using Engauge Digitizer.
* Indicate manual calculations by measuring the graphs, without using the Engauge Digitizer.
ratio-calc: indicate manual calculation by dividing intervention/control = result (ratio-calc)
ratio: indicate ratios given by study-authors such as normalization to control in case of small molecules data or in case of gene expression ratios, e.g. ratio of RANKL/OPG or Bcl-2/Bax.

Reference	Gene or analyte[a]	Official gene symbol or abbreviation[b]	Cell type (age/ number and sex of donor (health status), tooth type, isolation method, passages used, cell density/confluency)[a,c]	Flow type (Steady laminar, Pulsatile laminar, or Oscillatory laminar)[a,d]	FSS-duration and frequency[a]	FSS-magnitude[a]	FSS apparatus[a]	Gene expression: Increase, decrease, no change (method w/ reference gene); methods: RT-qPCR, sqPCR, northern hybridization[f]	Gene expression: when it reaches peak and peak's magnitude (fold change; relative gene expression; times or ratio; unclear = ?)[f]	Protein expression: Increase, decrease, no change (method w/reference); methods: ELISA, WB, RIA, EMSA, IF[f]	Protein expression: When it reaches peak and peak's magnitude (times or ratio; unclear = ?)[f]	Remarks
Haugh et al. (2015)	RANKL	Tnfsf11	MLO-Y4 (n.g./ n.g. (n.g.), n.g., n.g., n.g., 200000 cells per slide/ n.g.)	Oscillatory laminar	1h @ 1Hz	1Pa	Custom-made	Decrease (RT-qPCR, GAPDH)	0.8 (FC)†			
Kulkarni et al. (2012a)	CYR61	Ccn1	MLO-Y4 (n.g./ n.g. (n.g.), n.g., n.g., P30-31, 2×10⁴ cells per cm²/ n.g.)	Pulsatile laminar	1h @ 5Hz	Mean FSS: 0.7Pa, pulse amplitude of 0.3Pa, peak SS: 8.4Pa/s	Custom-made	Increase (RT-qPCR, GAPDH)	1854.6/ 1150.3 = 1.6 (ratio-calc)†			TaqMan (reference n.g.)
Kulkarni et al. (2012a)	OPG	Tnfrsf11b	MLO-Y4 (n.g./ n.g. (n.g.), n.g., n.g., P30-31, 2×10⁴ cells per cm²/ n.g.)	Pulsatile laminar	1h @ 5Hz	Mean FSS: 0.7Pa, pulse amplitude of 0.3Pa, peak SS: 8.4Pa/s	Custom-made	Increase (RT-qPCR, GAPDH)	26.1/18 = 1.45 (ratio-calc)†			
Kulkarni et al. (2012a)	RANKL	Tnfsf11	MLO-Y4 (n.g./ n.g. (n.g.), n.g., n.g., P30-31, 2×10⁴ cells per cm²/ n.g.)	Pulsatile laminar	1h @ 5Hz	Mean FSS: 0.7Pa, pulse amplitude of 0.3Pa, peak SS: 8.4Pa/s	Custom-made	Increase (RT-qPCR, GAPDH)	33.2/10 = 3.3 (ratio-calc)†			
Juffer et al. (2012)	HGF	Hgf	MLO-Y4 (n.g./ n.g. (n.g.), n.g., n.g., n.g., 3x10⁵ cells per glass slide / n.g.)	Pulsatile laminar	1h (sampling 1h, 3h, 6h, 24h post FSS) / 5Hz	22Pa/s; 44Pa/s	Custom-made	22Pa/s: decrease with plateau (RT-qPCR, GAPDH)	0-6h post FSS: ≈0.6 (rel.)†	Increase (ELISA)	24h post-FF: 4.6ng;4.6/2 = 2.3 (ratio-calc)†	22Pa/s ("low shear stress"); 44Pa/s ("high shear stress")
Juffer et al. (2012)	IGF-I Ea isoform	Igf1	MLO-Y4 (n.g./ n.g. (n.g.), n.g., n.g., n.g., 3x10⁵ cells per glass slide / n.g.)	Pulsatile laminar	1h (sampling 1h, 3h, 6h, 24h post FSS) / 5Hz	22Pa/s; 44Pa/s	Custom-made	22Pa/s: temporary increase then temporary decrease then increase with plateau<br>44Pa/s: increase (RT-qPCR, GAPDH)	22Pa/s @ 0h post FSS: 1.1 (rel.)†<br>22Pa/s @ 1h post FSS: 0.8 (rel.)†<br>22Pa/s @ 3h post FSS: 1.5 (rel.)†<br>44Pa/s @ 0h post FSS: 3.2 (rel.)†			22Pa/s ("low shear stress"); 44Pa/s ("high shear stress")
Juffer et al. (2012)	MGF (mechano growth factor)	Igf1	MLO-Y4 (n.g./ n.g. (n.g.), n.g., n.g., n.g., 3x10⁵ cells per glass slide / n.g.)	Pulsatile laminar	1h (sampling 1h, 3h, 6h, 24h post FSS) / 5Hz	22Pa/s; 44Pa/s	Custom-made	22Pa/s: decrease then post-FF increase with plateau<br>44Pa/s: increase (RT-qPCR, GAPDH)	22 Pa/s @ 0h post FSS: 0.7 (rel.)†<br>22 Pa/s @ 3h post FSS: 1.2 (rel.)†<br>22 Pa/s @ 6h post FSS: 1.3 (rel.)†<br>22 Pa/s @ 24h post FSS: 1.2 (rel.)†<br>44 Pa/s @ 6h post FSS: 1.9 (rel.)†			22Pa/s ("low shear stress"); 44Pa/s ("high shear stress"); Primer-BLAST: Igf1 (Mgf is a fragment of Igf1)
Juffer et al. (2012)	NO	Nitric oxide	MLO-Y4 (n.g./ n.g. (n.g.), n.g., n.g., n.g., 3x10⁵ cells per glass slide / n.g.)	Pulsatile laminar	15min / 5Hz	22Pa/s; 44Pa/s	Custom-made			Increase (Griess, NO_2^-)	15min: 8.6nmole; 8.6/2.3 = 3.7 (ratio-calc)†	22Pa/s ("low shear stress"); 44Pa/s ("high shear stress")
Juffer et al. (2012)	VEGF	Vegfa	MLO-Y4 (n.g./ n.g. (n.g.), n.g., n.g., n.g., 3x10⁵ cells per glass slide / n.g.)	Pulsatile laminar	1h (sampling 1h, 3h, 6h, 24h post FSS) / 5Hz	22Pa/s; 44Pa/s	Custom-made	22Pa/s: increase followed by plateau (RT-qPCR, GAPDH)	1h post FSS: 2.4 (rel.)†<br>6-24h post FSS: ≈1.6 (rel.)	Increase (ELISA)	24h post FSS: 1.6ng; 1.6/0.8 = 2 (ratio-calc)†	22Pa/s ("low shear stress"); 44Pa/s ("high shear stress")
Santos et al. (2009)	APC	Apc	MLO-Y4 (n.g./ n.g. (n.g.), n.g., n.g., n.g., 2×10⁵ cells per slide/ n.g.)	Pulsatile laminar	1h (1h, 3h post-PFF incubation) / 5Hz	mean FSS: 0.7Pa, pulse amplitude of 0.3Pa	Custom-made	Temporary decrease then increase (RT-qPCR, GAPDH)	0.5h post-PFF: 0.5 (rel.)†<br>1h post-PFF: 1.5 (rel.)†			
Santos et al. (2009)	c-jun	Jun	MLO-Y4 (n.g./ n.g. (n.g.), n.g., n.g., n.g., 2×10⁵ cells per slide/ n.g.)	Pulsatile laminar	1h (1h, 3h post-PFF incubation) / 5Hz	mean FSS: 0.7Pa, pulse amplitude of 0.3Pa	Custom-made	Increase (RT-qPCR, GAPDH)	3h post-PFF: 1.3 (rel.)†			
Santos et al. (2009)	CCND1	Ccnd1	MLO-Y4 (n.g./ n.g. (n.g.), n.g., n.g., n.g., 2×10⁵ cells per slide/ n.g.)	Pulsatile laminar	1h (1h, 3h post-PFF incubation) / 5Hz	mean FSS: 0.7Pa, pulse amplitude of 0.3Pa	Custom-made	Increase (RT-qPCR, GAPDH)	3h post-PFF: 2.0 (rel.)†			
Santos et al. (2009)	CD44	Cd44	MLO-Y4 (n.g./ n.g. (n.g.), n.g., n.g., n.g., 2×10⁵ cells per slide/ n.g.)	Pulsatile laminar	1h (1h, 3h post-PFF incubation) / 5Hz	mean FSS: 0.7Pa, pulse amplitude of 0.3Pa	Custom-made	Increase (RT-qPCR, GAPDH)	3h post-PFF: 1.7 (rel.)†			

[a] Entry provided as reported in the given study.
[b] Human genes were confirmed with the HUGO Gene Nomenclature Committee (HGNC; URL: https://www.genenames.org); mouse genes were confirmed with the Mouse Genome Informatics (MGI; URL: https://www.informatics.jax.org/genes.shtml) after checking the specificity of primers with Primer-BLAST.
[c] Sex of donors: "M" – male, "F" – female; Tooth type: "PM" – premolar, "M" – molar; dig. Indicate isolation by cell digestion; Exp. indicate isolation by cell explant; Cell density: given in cells/cm² if not otherwise mentioned.
[d] Flow type deduced from the description of the FSS apparatus given by the authors.
[f] RT-qPCR (reverse-transcriptase quantitative polymerase chain reaction); sqPCR (semi-quantitative polymerase chain reaction); ELISA (enzyme-linked immuno absorbent assay); WB (western blotting); RIA (radioimmunoassay); EMSA (electromobility shift assay); IF (immunofluorescence)

rel.: indicate relative gene expression. Is entitled to percentages or gene expression ratios normalized to control, and not calculated by ΔΔCT.
FC: indicate fold change. When Author mentions the use of ΔΔCT or the method according to Livak & Schmittken (2004) in calculating FC.
n.g.: not given. For information not given by study-authors.
† Information derived from figures using Engauge Digitizer.
* Indicate manual calculations by measuring the graphs, without using the Engauge Digitizer.
ratio-calc: indicate manual calculation by dividing intervention/control = result (ratio-calc)
ratio: indicate ratios given by study-authors such as normalization to control in case of small molecules data or in case of gene expression ratios, e.g. ratio of RANKL/OPG or Bcl-2/Bax.

Reference	Gene or analyte[a]	Official gene symbol or abbreviation[b]	Cell type (age/ number and sex of donor (health status), tooth type, isolation method, passages used, cell density/confluency)[a,c]	Flow type (Steady laminar, Pulsatile laminar, or Oscillatory laminar)[a,d]	FSS-duration and frequency[a]	FSS-magnitude[a]	FSS apparatus[a]	Gene expression: Increase, decrease, no change (method w/ reference gene); methods: RT-qPCR, sqPCR, northern hybridization[f]	Gene expression: when it reaches peak and peak's magnitude (fold change; relative gene expression; times or ratio; unclear = ?)[f]	Protein expression: Increase, decrease, no change (method w/reference); methods: ELISA, WB, RIA, EMSA, IF[f]	Protein expression: When it reaches peak and peak's magnitude (times or ratio; unclear = ?)[f]	Remarks
Santos et al. (2009)	Fzd6	Fzd6	MLO-Y4 (n.g./ n.g. (n.g.), n.g., n.g., n.g., 2×10^{5} cells per slide/ n.g.)	Pulsatile laminar	1h (1h, 3h post-PFF incubation) / 5Hz	mean FSS: 0.7Pa, pulse amplitude of 0.3Pa	Custom-made	Decrease (RT-qPCR, GAPDH)	1h post-PFF: 0.8 (rel.)†			
Santos et al. (2009)	Gja1 (connexin 43)	Gja1	MLO-Y4 (n.g./ n.g. (n.g.), n.g., n.g., n.g., 2×10^{5} cells per slide/ n.g.)	Pulsatile laminar	1h (1h, 3h post-PFF incubation) / 5Hz	mean FSS: 0.7Pa, pulse amplitude of 0.3Pa	Custom-made	Increase (RT-qPCR, GAPDH)	1h post-PFF: 1.5 (rel.)†			
Santos et al. (2009)	LRP5	Lrp5	MLO-Y4 (n.g./ n.g. (n.g.), n.g., n.g., n.g., 2×10^{5} cells per slide/ n.g.)	Pulsatile laminar	1h (1h, 3h post-PFF incubation) / 5Hz	mean FSS: 0.7Pa, pulse amplitude of 0.3Pa	Custom-made	Decrease (RT-qPCR, GAPDH)	3h post-PFF: 0.3 (rel.)†			
Santos et al. (2009)	NO	Nitric oxide	MLO-Y4 (n.g./ n.g. (n.g.), n.g., n.g., n.g., 2×10^{5} cells per slide/ n.g.)	Pulsatile laminar	1h / 5Hz	mean FSS: 0.7Pa, pulse amplitude of 0.3Pa	Custom-made			Increase (Griess, NO_2^-)	178.6nmol; 178.6/30.2 = 5.9 (ratio-calc)†	
Santos et al. (2009)	sFRP4	Sfrp4	MLO-Y4 (n.g./ n.g. (n.g.), n.g., n.g., n.g., 2×10^{5} cells per slide/ n.g.)	Pulsatile laminar	1h (1h, 3h post-PFF incubation) / 5Hz	mean FSS: 0.7Pa, pulse amplitude of 0.3Pa	Custom-made	Decrease with plateau then increase (RT-qPCR, GAPDH)	0.5h post-PFF: 0.65 (rel.)†<br>1h post-PFF: 0.5 (rel.)†<br>3h post-PFF: 1.4 (rel.)†			
Santos et al. (2009)	Wnt3a	Wnt3a	MLO-Y4 (n.g./ n.g. (n.g.), n.g., n.g., n.g., 2×10^{5} cells per slide/ n.g.)	Pulsatile laminar	1h (1h, 3h post-PFF incubation) / 5Hz	mean FSS: 0.7Pa, pulse amplitude of 0.3Pa	Custom-made	Decrease then increase (RT-qPCR, GAPDH)	0.5h post-PFF: 0.5 (rel.)†<br>1h post-PFF: 1.7 (rel.)†			
Santos et al. (2009)	β-catenin	Ctnnb1	MLO-Y4 (n.g./ n.g. (n.g.), n.g., n.g., n.g., 2×10^{5} cells per slide/ n.g.)	Pulsatile laminar	1h (1h, 3h post-PFF incubation) / 5Hz	mean FSS: 0.7Pa, pulse amplitude of 0.3Pa	Custom-made	Temporary decrease then increase (RT-qPCR, GAPDH)	0.5h post-PFF: 0.9 (rel.)†<br>1h post-PFF: 1.3 (rel.)†			
Xu et al. (2014)	Cox-2	Ptgs2	MLO-Y4 (n.g./ n.g. (n.g.), n.g., n.g., n.g., 2.2×10^{6} per slide/ n.g.)	Oscillatory laminar	2h @ 1Hz	1Pa (10dyn/cm^2)	Custom-made	Increase (RT-qPCR, 18S)	2h: 1.74(FC)†			
Xu et al. (2014)	iNOS	Nos2	MLO-Y4 (n.g./ n.g. (n.g.), n.g., n.g., n.g., 2.2×10^{6} per slide/ n.g.)	Oscillatory laminar	2h @ 1Hz	1Pa (10dyn/cm^2)	Custom-made	Increase (RT-qPCR, 18S)	2h: 4.94 (FC)†			
Xu et al. (2014)	NO	Nitric oxide	MLO-Y4 (n.g./ n.g. (n.g.), n.g., n.g., n.g., 2.2×10^{6} per slide/ n.g.)	Oscillatory laminar	2h @ 1Hz	1Pa (10dyn/cm^2)	Custom-made			Increase (Griess, NO_2^-)	11.8µM;11.8/4.9 = 2.4 (ratio-calc)†	
Xu et al. (2014)	PC1	Pkd1	MLO-Y4 (n.g./ n.g. (n.g.), n.g., n.g., n.g., 2.2×10^{6} per slide/ n.g.)	Oscillatory laminar	2h @ 1Hz	1Pa (10dyn/cm^2)	Custom-made	Increase (RT-qPCR, 18S)	2h: 5 (FC)†			
Xu et al. (2014)	PC2	Pkd2	MLO-Y4 (n.g./ n.g. (n.g.), n.g., n.g., n.g., 2.2×10^{6} per slide/ n.g.)	Oscillatory laminar	2h @ 1Hz	1Pa (10dyn/cm^2)	Custom-made	Increase (RT-qPCR, 18S)	2h: 10.6 (FC)†			
Xu et al. (2014)	PGE2	PGE2	MLO-Y4 (n.g./ n.g. (n.g.), n.g., n.g., n.g., 2.2×10^{6} per slide/ n.g.)	Oscillatory laminar	2h @ 1Hz	1Pa (10dyn/cm^2)	Custom-made			Increase (EIA)	178.62µg/mL;178.62/30.2 = 5.9 (ratio-calc)†	
Fahlgren et al. (2018)	Caspase 3/7 activity	Casp3; Casp7	MLO-Y4 (n.g./ n.g. (n.g.), n.g., n.g., P31–33, 1.3×10^{3} cells/cm^2 per slide/ n.g.)	Pulsatile Laminar (SP: square wave; PL: sinusoidal wave)	60min / (1Hz, 5Hz)	2.9±2.9Pa @ 1Hz square wave (SP); 0.7±0.7Pa @ 5Hz, sinusoidal wave (PL); Unloaded	Custom-made			Increase (luminescence assay)	SP/unloading: 1.2RLU/ngDNA; 1.2/0.95 = 1.3 (ratio-calc)†<br>PL/unloading: 0.99RLU/ngDNA; 0.99/0.95 = 1.04 (ratio-calc)†<br>SP/PL: 1.2RLU/ngDNA; 1.2/0.99 = 1.2 (ratio-calc)†	SP (supraphysiological loading), PL (physiological loading); Caspase-Glo 3/7 assay (Promega)
Fahlgren et al. (2018)	COX-2	Ptgs2	MLO-Y4 (n.g./ n.g. (n.g.), n.g., n.g., P31–33, 1.3×10^{3} cells/cm^2 per slide/ n.g.)	Pulsatile Laminar (SP: square wave; PL: sinusoidal wave)	60min / (1Hz, 5Hz)	2.9±2.9Pa @ 1Hz square wave (SP); 0.7±0.7Pa @ 5Hz, sinusoidal wave (PL); Unloaded	Custom-made	SP/PL: decrease<br>SP/unloading: no change<br>PL/unloading: increase (RT-qPCR, S18)	SP/PL: 0.81/0.99 = 0.81 (ratio-calc)<br>SP/unloading: 0.81/0.81 = 1 (ratio-calc)†<br>PL/unloading: 0.99/0.81 = 1.2 (ratio-calc)†			SP (supraphysiological loading), PL (physiological loading)

[a] Entry provided as reported in the given study.
[b] Human genes were confirmed with the HUGO Gene Nomenclature Committee (HGNC; URL: https://www.genenames.org); mouse genes were confirmed with the Mouse Genome Informatics (MGI; URL: https://www.informatics.jax.org/genes.shtml) after checking the specificity of primers with Primer-BLAST.
[c] Sex of donors: "M" – male, "F" – female; Tooth type: "PM" – premolar, "M" – molar; dig. Indicate isolation by cell digestion; Exp. indicate isolation by cell explant; Cell density: given in cells/cm^2 if not otherwise mentioned.
[d] Flow type deduced from the description of the FSS apparatus given by the authors.
f RT-qPCR (reverse-transcriptase quantitative polymerase chain reaction); sqPCR (semi-quantitative polymerase chain reaction); ELISA (enzyme-linked immuno absorbent assay); WB (western blotting); RIA (radioimmunoassay); EMSA (electromobility shift assay); IF (immunofluorescence)

rel.: indicate relative gene expression. Is entitled to percentages or gene expression ratios normalized to control, and not calculated by ΔΔCT.
FC: indicate fold change. When Author mentions the use of ΔΔCT or the method according to Livak & Schmittken (2004) in calculating FC.
n.g.: not given. For information not given by study-authors.
† Information derived from figures using Engauge Digitizer.
* Indicate manual calculations by measuring the graphs, without using the Engauge Digitizer.
ratio-calc: indicate manual calculation by dividing intervention/control = result (ratio-calc)
ratio: indicate ratios given by study-authors such as normalization to control in case of small molecules data or in case of gene expression ratios, e.g. ratio of RANKL/OPG or Bcl-2/Bax.

Reference	Gene or analyte[a]	Official gene symbol or abbreviation[b]	Cell type (age/ number and sex of donor (health status), tooth type, isolation method, passages used, cell density/confluency)[a,c]	Flow type (Steady laminar, Pulsatile laminar, or Oscillatory laminar)[a,d]	FSS-duration and frequency[a]	FSS-magnitude[a]	FSS apparatus[a]	Gene expression: Increase, decrease, no change (method w/ reference gene); methods: RT-qPCR, sqPCR, northern hybridization[f]	Gene expression: when it reaches peak and peak's magnitude (fold change; relative gene expression; times or ratio; unclear = ?)[f]	Protein expression: Increase, decrease, no change (method w/reference); methods: ELISA, WB, RIA, EMSA, IF[f]	Protein expression: When it reaches peak and peak's magnitude (times or ratio; unclear = ?)[f]	Remarks
Fahlgren et al. (2018)	Ki-67	Mki67	MLO-Y4 (n.g./ n.g. (n.g.), n.g., n.g., P31–33, 1.3×10^3 cells/cm^2 per slide/ n.g.)	Pulsatile Laminar (SP: square wave; PL: sinusoidal wave)	60min / (1Hz, 5Hz)	2.9±2.9Pa @ 1Hz square wave (SP); 0.7±0.7Pa @ 5Hz, sinusoidal wave (PL); Unloaded	Custom-made	Decrease (RT-qPCR, S18)	SP/PL: 0.89/0.99 = 0.89 (ratio-calc)†<br>SP/unloading: 0.89/1.028 = 0.86 (ratio-calc)†<br>PL/unloading: 0.99/1.028 = 0.96 (ratio-calc)†			SP (supraphysiological loading), PL (physiological loading)
Fahlgren et al. (2018)	NO	Nitric oxide	MLO-Y4 (n.g./ n.g. (n.g.), n.g., n.g., P31–33, 1.3×10^3 cells/cm^2 per slide/ n.g.)	Pulsatile Laminar (SP: square wave; PL: sinusoidal wave)	60min / (1Hz, 5Hz)	2.9±2.9Pa @ 1Hz square wave (SP); 0.7±0.7Pa @ 5Hz, sinusoidal wave (PL); Unloaded	Custom-made			Increase (Griess, NO_2^-)	5 min post-FF:<br>SP/PL: 2.7-fold<br>SP/unloading: 33-fold<br>15min post-FF:<br>SP/PL: 2.9-fold<br>SP/unloading: 43-fold<br>60 min post-FF:<br>SP/PL: 2.3-fold<br>SP/unloading: 58-fold	SP (supraphysiological loading), PL (physiological loading)
Fahlgren et al. (2018)	OPG	Tnfrsf11b	MLO-Y4 (n.g./ n.g. (n.g.), n.g., n.g., P31–33, 1.3×10^3 cells/cm^2 per slide/ n.g.)	Pulsatile Laminar (SP: square wave; PL: sinusoidal wave)	60min / (1Hz, 5Hz)	2.9±2.9Pa @ 1Hz square wave (SP); 0.7±0.7Pa @ 5Hz, sinusoidal wave (PL); Unloaded	Custom-made	SP/PL: decrease<br>SP/unloading increase<br>PL/unloading: increase (RT-qPCR, S18)	SP/PL: 0.89/1.00 = 0.89 (ratio-calc)†<br>SP/unloading: 0.89/0.8 = 1.1 (ratio-calc)†<br>PL/unloading: 1.00/0.8 = 1.25 (ratio-calc)†	ELISA:<br>SP/PL: increase<br>SP/unloading increase<br>PL/unloading: decrease	SP/PL: 7.128pg/mL; 7.128/0.957 = 7.4 (ratio-calc)†<br>SP/unloading: 7.1pg/mL; 7.128/1.915 = 3.7 (ratio-calcL)†<br>PL/unloading: 0.957pg/mL; 0.957/1.915 = 0.5 (ratio-calc)†	SP (supraphysiological loading), PL (physiological loading)
Fahlgren et al. (2018)	PGE2	PGE2	MLO-Y4 (n.g./ n.g. (n.g.), n.g., n.g., P31–33, 1.3×10^3 cells/cm^2 per slide/ n.g.)	Pulsatile Laminar (SP: square wave; PL: sinusoidal wave)	60min / (1Hz, 5Hz)	2.9±2.9Pa @ 1Hz square wave (SP); 0.7±0.7Pa @ 5Hz, sinusoidal wave (PL); Unloaded	Custom-made			SP/PL: decrease<br>SP/unloading: increase<br>PL/unloading: increase (ELISA)	SP/PL: 0.88/0.99 = 0.88 (ratio-calc)†<br>SP/unloading: 0.88/0.78 = 1.1 (ratio-calc)†<br>PL/unloading: 0.99/0.78 = 1.3 (ratio-calc)†	SP (supraphysiological loading), PL (physiological loading)
Fahlgren et al. (2018)	RANKL	Tnfsf11	MLO-Y4 (n.g./ n.g. (n.g.), n.g., n.g., P31–33, 1.3×10^3 cells/cm^2 per slide/ n.g.)	Pulsatile Laminar (SP: square wave; PL: sinusoidal wave)	60min / (1Hz, 5Hz)	2.9±2.9Pa @ 1Hz square wave (SP); 0.7±0.7Pa @ 5Hz, sinusoidal wave (PL); Unloaded	Custom-made	SP/PL: decrease<br>SP/unloading decrease<br>PL/unloading: decrease (RT-qPCR, S18)	SP/PL: 0.75/ 0.99 = 0.75 (ratio-calc)†<br>SP/unloading: 0.75/ 1.08 = 0.69 (ratio-calc)†<br>PL/unloading: 0.99/1.08 = 0.92 (ratio-calc)†	SP/PL: increase<br>SP/unloading decrease<br>PL/unloading: decrease (ELISA)	SP/PL: 1.05pg/mL; 1.05/0.99 = 1.1 (ratio-clac)†<br>SP/unloading: 1.05pg/mL; 1.05/1.05 = 1 (ratio-calc)†<br>PL/unloading: 0.99pg/mL; 0.99/1.05 = 0.94 (ratio-calc)†	SP (supraphysiological loading), PL (physiological loading)<br>SP/PL: increase<br>SP/unloading increase
Fahlgren et al. (2018)	RANKL / OPG	ratio (RANKL/OPG)	MLO-Y4 (n.g./ n.g. (n.g.), n.g., n.g., P31–33, 1.3×10^3 cells/cm^2 per slide/ n.g.)	Pulsatile Laminar (SP: square wave; PL: sinusoidal wave)	60min / (1Hz, 5Hz)	2.9±2.9Pa @ 1Hz square wave (SP); 0.7±0.7Pa @ 5Hz, sinusoidal wave (PL); Unloaded	Custom-made			SP/PL: decrease (ELISA)<br>SP/unloading: decrease (ELISA)<br>PL/unloading: increase (ELISA)	SP/PL: 0.15/1.0 = 0.15 (ratio-calc)†<br>SP/unloading: 0.15/0.54 = 0.3 (ratio-calc)†<br>PL/unloading: 1.0/0.54 = 1.85 (ratio-calc)†	SP (supraphysiological loading), PL (physiological loading)
Kitase et al. (2010)	p-GSK3a / GSK3a	Gsk3a	MLO-Y4 (n.g./ n.g. (n.g.), n.g., n.g., n.g., 3×10^4 cells per cm^2/ 80%)	Steady laminar	2h/n.g.	16dyn/cm^2	n.g.			Increase (WB, GSK3a)	45min: 4.5 (ratio)†	
Kitase et al. (2010)	p-GSK3b / GSK3b	Gsk3b	MLO-Y4 (n.g./ n.g. (n.g.), n.g., n.g., n.g., 3×10^4 cells per cm^2/ 80%)	Steady laminar	2h/n.g.	16dyn/cm^2	n.g.			Increase (WB, GSK3b)	45min: 3.7 (ratio)†	
Chen et al. (2015)	Dlx5	Dlx5	MLO-Y4 (n.g./ n.g. (n.g.), n.g., n.g., n.g., 4000 cells per cm^2/ 80%)	Oscillatory laminar	24h @ 0.5Hz	Amplitude of 1.5cm	Rocking platform	Decrease (RT-qPCR, GAPDH)	152/330.7 = 0.45 (ratio-calc)†			
Chen et al. (2015)	FABP4	Fabp4	MLO-Y4 (n.g./ n.g. (n.g.), n.g., n.g., n.g., 4000 cells per cm^2/ 80%)	Oscillatory laminar	24h @ 0.5Hz	Amplitude of 1.5cm	Rocking platform	Decrease (RT-qPCR, GAPDH)	0.1/0.31 = 0.32 (ratio-calc)†			

[a] Entry provided as reported in the given study.
[b] Human genes were confirmed with the HUGO Gene Nomenclature Committee (HGNC; URL: https://www.genenames.org); mouse genes were confirmed with the Mouse Genome Informatics (MGI; URL: https://www.informatics.jax.org/genes.shtml) after checking the specificity of primers with Primer-BLAST.
[c] Sex of donors: "M" – male, "F" – female; Tooth type: "PM" – premolar, "M" – molar; dig. Indicate isolation by cell digestion; Exp. indicate isolation by cell explant; Cell density: given in cells/cm^2 if not otherwise mentioned.
[d] Flow type deduced from the description of the FSS apparatus given by the authors.
f RT-qPCR (reverse-transcriptase quantitative polymerase chain reaction); sqPCR (semi-quantitative polymerase chain reaction); ELISA (enzyme-linked immuno absorbent assay); WB (western blotting); RIA (radioimmunoassay); EMSA (electromobility shift assay); IF (immunofluorescence)

rel.: indicate relative gene expression. Is entitled to percentages or gene expression ratios normalized to control, and not calculated by ΔΔCT.
FC: indicate fold change. When Author mentions the use of ΔΔCT or the method according to Livak & Schmittken (2004) in calculating FC.
n.g.: not given. For information not given by study-authors.
† Information derived from figures using Engauge Digitizer.
* Indicate manual calculations by measuring the graphs, without using the Engauge Digitizer.
ratio-calc: indicate manual calculation by dividing intervention/control = result (ratio-calc)
ratio: indicate ratios given by study-authors such as normalization to control in case of small molecules data or in case of gene expression ratios, e.g. ratio of RANKL/OPG or Bcl-2/Bax.

Reference	Gene or analyte[a]	Official gene symbol or abbreviation[b]	Cell type (age/ number and sex of donor (health status), tooth type, isolation method, passages used, cell density/confluency)[a,c]	Flow type (Steady laminar, Pulsatile laminar, or Oscillatory laminar)[a,d]	FSS-duration and frequency[a]	FSS-magnitude[a]	FSS apparatus[a]	Gene expression: Increase, decrease, no change (method w/ reference gene); methods: RT-qPCR, sqPCR, northern hybridization[f]	Gene expression: when it reaches peak and peak's magnitude (fold change; relative gene expression; times or ratio; unclear = ?)[f]	Protein expression: Increase, decrease, no change (method w/reference); methods: ELISA, WB, RIA, EMSA, IF[f]	Protein expression: When it reaches peak and peak's magnitude (times or ratio; unclear = ?)[f]	Remarks
Chen et al. (2015)	LPL	Lpl	MLO-Y4 (n.g./ n.g. (n.g.), n.g., n.g., n.g., 4000 cells per cm^2/ 80%)	Oscillatory laminar	24h @ 0.5Hz	Amplitude of 1.5cm	Rocking platform	Decrease (RT-qPCR, GAPDH)	0.05/0.18 = 0.3 (ratio-calc)†			
Chen et al. (2015)	OCN	Bglap	MLO-Y4 (n.g./ n.g. (n.g.), n.g., n.g., n.g., 4000 cells per cm^2/ 80%)	Oscillatory laminar	24h @ 0.5Hz	Amplitude of 1.5cm	Rocking platform	Increase (RT-qPCR, GAPDH)	0.8/0.03 = 26.6 (ratio-calc)†			
Chen et al. (2015)	OPN	Spp1	MLO-Y4 (n.g./ n.g. (n.g.), n.g., n.g., n.g., 4000 cells per cm^2/ 80%)	Oscillatory laminar	24h @ 0.5Hz	Amplitude of 1.5cm	Rocking platform	Increase (RT-qPCR, GAPDH)	6.3/2.3 = 2.7(ratio-calc)†			
Chen et al. (2015)	Osx (osterix)	Sp7	MLO-Y4 (n.g./ n.g. (n.g.), n.g., n.g., n.g., 4000 cells per cm^2/ 80%)	Oscillatory laminar	24h @ 0.5Hz	Amplitude of 1.5cm	Rocking platform	Increase (RT-qPCR, GAPDH)	0.6/0.19 = 3 (ratio-calc)†			
Chen et al. (2015)	PPARγ	Pparg	MLO-Y4 (n.g./ n.g. (n.g.), n.g., n.g., n.g., 4000 cells per cm^2/ 80%)	Oscillatory laminar	24h @ 0.5Hz	Amplitude of 1.5cm	Rocking platform	Decrease (RT-qPCR, GAPDH)	0.18/0.25 = 0.72 (ratio-calc)†			
Chen et al. (2015)	Runx2	Runx2	MLO-Y4 (n.g./ n.g. (n.g.), n.g., n.g., n.g., 4000 cells per cm^2/ 80%)	Oscillatory laminar	24h @ 0.5Hz	Amplitude of 1.5cm	Rocking platform	Decrease (RT-qPCR, GAPDH)	0.04/0.19 = 0.21 (ratio-calc)†			
Li et al. (2019)	ARRAY		MLO-Y4 (n.g./ n.g. (n.g.), n.g., n.g., n.g., n.g./ n.g.)	Oscillatory laminar	2h @ 1Hz	15 dyn/cm^2	ibidi pump system (ibidi, Germany)		PRJNA551282			
Li et al. (2019)	Cyr61	Ccn1	MLO-Y4 (n.g./ n.g. (n.g.), n.g., n.g., n.g., n.g./ n.g.)	Oscillatory laminar	2h @ 1Hz	15 dyn/cm^2	ibidi pump system (ibidi, Germany)	Increase (RT-qPCR, ribosomal protein S2)	125/25 = 5 (ratio-calc)*			TaqMan (reference approved)
Li et al. (2019)	Piezo1	Piezo1	MLO-Y4 (n.g./ n.g. (n.g.), n.g., n.g., n.g., n.g./ n.g.)	Oscillatory laminar	2h @ 1Hz	15 dyn/cm^2	ibidi pump system (ibidi, Germany)	Increase (RT-qPCR, ribosomal protein S2)	5.8/2.5 = 2.32 (ratio-calc)*			
Li et al. (2019)	Piezo2	Piezo2	MLO-Y4 (n.g./ n.g. (n.g.), n.g., n.g., n.g., n.g./ n.g.)	Oscillatory laminar	2h @ 1Hz	15 dyn/cm^2	ibidi pump system (ibidi, Germany)	No change (RT-qPCR, ribosomal protein S2)				
Li et al. (2019)	Ptgs2	Ptgs2	MLO-Y4 (n.g./ n.g. (n.g.), n.g., n.g., n.g., n.g./ n.g.)	Oscillatory laminar	2h @ 1Hz	15 dyn/cm^2	ibidi pump system (ibidi, Germany)	Increase (RT-qPCR, ribosomal protein S2)	15/2 = 7.5 (ratio-calc)*			
Li et al. (2019)	Tnfrsf11b	Tnfrsf11b	MLO-Y4 (n.g./ n.g. (n.g.), n.g., n.g., n.g., n.g./ n.g.)	Oscillatory laminar	2h @ 1Hz	15 dyn/cm^2	ibidi pump system (ibidi, Germany)	Increase (RT-qPCR, ribosomal protein S2)	0.3/0.12 = 2.5 (ratio-calc)*			
Li et al. (2019)	Wnt1	Wnt1	MLO-Y4 (n.g./ n.g. (n.g.), n.g., n.g., n.g., n.g./ n.g.)	Oscillatory laminar	2h @ 1Hz	15 dyn/cm^2	ibidi pump system (ibidi, Germany)	Increase (RT-qPCR, ribosomal protein S2)	0.08/0.03 = 2.6 (ratio-calc)*			
Alford et al. (2003)	Cx43	Gja1	MLOY-4 (n.g./ n.g. (n.g.), n.g., n.g., n.g., 250000 cells per slide/ n.g.)	Oscillatory laminar	1h @ 5Hz	±10dyn/cm^2	Custom-made			Increase (WB, n.g.)	2.4 (ratio)†	
Kitase et al. (2014)	ARRAY		MLO-Y4 (n.g./ n.g. (n.g.), n.g., n.g., n.g., n.g./ n.g.)	n.g.	2h/n.g.	16dyn/cm^2	n.g.					
Kitase et al. (2014)	CCL7	Ccl7	MLO-Y4 (n.g./ n.g. (n.g.), n.g., n.g., n.g., n.g./ n.g.)	n.g.	2h (sampling 24h Post FSS) /n.g.	16dyn/cm^2	n.g.	Increase (Northern blot, GAPDH)	24h post FSS: 8/0.4 = 20 (ratio-calc)†			
Ren et al. (2013)	Cx43	Gja1	MLO-Y4 (n.g./ n.g. (n.g.), n.g., n.g., n.g., 8×10^4 cells/ n.g.)	Oscillatory laminar	2h @ 1Hz (2h post-FF incubation)	12dyn	Parallel plate flow chamber (Pecon, Germany)			Increase (WB, actin)	1.2 (ratio)†	
Ren et al. (2013)	p-ERK 1/2	Mapk3; Mapk1	MLO-Y4 (n.g./ n.g. (n.g.), n.g., n.g., n.g., 8×10^4 cells/ n.g.)	Oscillatory laminar	2h @ 1Hz (2h post-FF incubation)	12dyn	Parallel plate flow chamber (Pecon, Germany)			Increase (WB, total ERK 1/2)	1.6 (ratio)†	
González et al. (2017)	OPG	Tnfrsf11b	MLO-Y4 (n.g./ n.g. (n.g.), n.g., n.g., n.g., n.g. n.g./n.g.)	Pulsatile laminar	30min/n.g.	10dyn/cm^2 (8.5cm/s)	FlexCell Streamer	Increase (RT-qPCR, 18S)	11.5/3.3 = 3.5 (ratio-calc)†			
González et al. (2017)	RunX2	Runx2	MLO-Y4 (n.g./ n.g. (n.g.), n.g., n.g., n.g., n.g. n.g./n.g.)	Pulsatile laminar	30min/n.g.	10dyn/cm^2 (8.5cm/s)	FlexCell Streamer	Increase (RT-qPCR, 18S)	9.3/4 = 2.3 (ratio-calc)†			
González et al. (2017)	VEGF	Vegfa	MLO-Y4 (n.g./ n.g. (n.g.), n.g., n.g., n.g., n.g. n.g./n.g.)	Pulsatile laminar	30min/n.g.	10dyn/cm^2 (8.5cm/s)	FlexCell Streamer	Increase (RT-qPCR, 18S)	2.96/1.4 = 2.1 (ratio-calc)†			
Bakker et al. (2013)	Cox2	Ptgs2	MLO-Y4 (n.g./ n.g. (n.g.), n.g., n.g., P30-38, 2×10^4 cells per cm^2/ n.g.)	Pulsatile laminar	60min @ 5Hz	0.7±0.3Pa	Custom-made	Increase (RT-qPCR, GAPDH)	2.49 (rel.)†			
Bakker et al. (2013)	NO	Nitrix oxide	MLO-Y4 (n.g./ n.g. (n.g.), n.g., n.g., P30-38, 2×10^4 cells per cm^2/ n.g.)	Pulsatile laminar	60min @ 5Hz	0.7±0.3Pa	Custom-made			Increase (Griess, NO_2^-)	17.5nmol/3×10^5 cells; 17.5/6.1 = 2.9 (ratio-calc)†	
Bakker et al. (2013)	Opg	Tnfrsf11b	MLO-Y4 (n.g./ n.g. (n.g.), n.g., n.g., P30-38, 2×10^4 cells per cm^2/ n.g.)	Pulsatile laminar	60min @ 5Hz	0.7±0.3Pa	Custom-made	Increase (RT-qPCR, GAPDH)	1.99 (rel.)†			

[a] Entry provided as reported in the given study.
[b] Human genes were confirmed with the HUGO Gene Nomenclature Committee (HGNC; URL: https://www.genenames.org); mouse genes were confirmed with the Mouse Genome Informatics (MGI; URL: https://www.informatics.jax.org/genes.shtml) after checking the specificity of primers with Primer-BLAST.
[c] Sex of donors: "M" – male, "F" – female; Tooth type: "PM" – premolar, "M" – molar; dig. Indicate isolation by cell digestion; Exp. indicate isolation by cell explant; Cell density: given in cells/cm^2 if not otherwise mentioned.
[d] Flow type deduced from the description of the FSS apparatus given by the authors.
[f] RT-qPCR (reverse-transcriptase quantitative polymerase chain reaction); sqPCR (semi-quantitative polymerase chain reaction); ELISA (enzyme-linked immuno absorbent assay); WB (western blotting); RIA (radioimmunoassay); EMSA (electromobility shift assay); IF (immunofluorescence)

rel.: indicate relative gene expression. Is entitled to percentages or gene expression ratios normalized to control, and not calculated by ΔΔCT.
FC: indicate fold change. When Author mentions the use of ΔΔCT or the method according to Livak & Schmittken (2004) in calculating FC.
n.g.: not given. For information not given by study-authors.
† Information derived from figures using Engauge Digitizer.
* Indicate manual calculations by measuring the graphs, without using the Engauge Digitizer.
ratio-calc: indicate manual calculation by dividing intervention/control = result (ratio-calc)
ratio: indicate ratios given by study-authors such as normalization to control in case of small molecules data or in case of gene expression ratios, e.g. ratio of RANKL/OPG or Bcl-2/Bax.

Reference	Gene or analyte[a]	Official gene symbol or abbreviation[b]	Cell type (age/ number and sex of donor (health status), tooth type, isolation method, passages used, cell density/confluency)[a,c]	Flow type (Steady laminar, Pulsatile laminar, or Oscillatory laminar)[a,d]	FSS-duration and frequency[a]	FSS-magnitude[a]	FSS apparatus[a]	Gene expression: Increase, decrease, no change (method w/ reference gene); methods: RT-qPCR, sqPCR, northern hybridization[f]	Gene expression: when it reaches peak and peak's magnitude (fold change; relative gene expression; times or ratio; unclear = ?)[f]	Protein expression: Increase, decrease, no change (method w/reference); methods: ELISA, WB, RIA, EMSA, IF[f]	Protein expression: When it reaches peak and peak's magnitude (times or ratio; unclear = ?)[f]	Remarks
Bakker et al. (2013)	PGE2	PGE2	MLO-Y4 (n.g./ n.g. (n.g.), n.g., n.g., P30-38, 2×10⁴ cells per cm²/ n.g.)	Pulsatile laminar	60min (sampling 0min, 10 min, 20min, 30min, 40min, 50min, 60min) / 5Hz	0.7±0.3Pa	Custom-made			Increase with plateau (ELISA)	5min: 512.8ng/3×10⁵ cells; 512.8/356 = 1.4 (ratio-calc)† 60min: 520.8ng/3×10⁵ cells; 520.8/428 = 1.2 (ratio-calc)†	
Bakker et al. (2013)	Rankl	Tnfsf11	MLO-Y4 (n.g./ n.g. (n.g.), n.g., n.g., P30-38, 2×10⁴ cells per cm²/ n.g.)	Pulsatile laminar	60min @ 5Hz	0.7±0.3Pa	Custom-made	Increase (RT-qPCR, GAPDH)	1.93 (rel.)†			
Kulkarni et al. (2010)	MEPE	Mepe	MLO-Y4 (n.g./ n.g. (n.g.), n.g., n.g., P30-31, 1×10³ cells per cm²/ n.g.)	Pulsatile laminar	1h (sampling 0h, 1h, 6h post FSS) / 5Hz	Mean shear stress: 0.7Pa, pulse amplitude of 0.3Pa, peak SS of 8.4Pa/s	Custom-made	Increase followed by decrease then increase (RT-qPCR, GAPDH)	0h post FSS: 0.1/0.04 = 2.5 (ratio-calc)† 1h post FSS: 0.049/0.06 = 0.8 (ratio-calc)† 6h post FSS: 0.06/0.03 = 2 (ratio-calc)†			
Kulkarni et al. (2010)	OPG	Tnfrsf11b	MLO-Y4 (n.g./ n.g. (n.g.), n.g., n.g., P30-31, 1×10³ cells per cm²/ n.g.)	Pulsatile laminar	1h (sampling 0h, 1h, 6h post FSS) / 5Hz	Mean shear stress: 0.7Pa, pulse amplitude of 0.3Pa, peak SS of 8.4Pa/s	Custom-made	Increase (RT-qPCR, GAPDH)	0h post FSS: 44.97/20.7 = 2.2 (ratio-calc)†			
Kulkarni et al. (2010)	PHEX	Phex	MLO-Y4 (n.g./ n.g. (n.g.), n.g., n.g., P30-31, 1×10³ cells per cm²/ n.g.)	Pulsatile laminar	1h (sampling 0h, 1h, 6h post FSS) / 5Hz	Mean shear stress: 0.7Pa, pulse amplitude of 0.3Pa, peak SS of 8.4Pa/s	Custom-made	Increase followed by decrease (RT-qPCR, GAPDH)	0h post FSS: 0.2/0.12 = 1.7 (ratio-calc)† 6h post FSS: 0.1/0.11 = 0.9 (ratio-calc)†			
Kulkarni et al. (2010)	RANKL	Tnfsf11	MLO-Y4 (n.g./ n.g. (n.g.), n.g., n.g., P30-31, 1×10³ cells per cm²/ n.g.)	Pulsatile laminar	1h (sampling 0h, 1h, 6h post FSS) / 5Hz	Mean shear stress: 0.7Pa, pulse amplitude of 0.3Pa, peak SS of 8.4Pa/s	Custom-made	Increase (RT-qPCR, GAPDH)	0h post FSS: 38.9/20.8 = 1.9 (ratio-calc)†			
Kulkarni et al. (2010)	RANKL / OPG	ratio (RANKL/OPG)	MLO-Y4 (n.g./ n.g. (n.g.), n.g., n.g., P30-31, 1×10³ cells per cm²/ n.g.)	Pulsatile laminar	1h (sampling 0h, 1h, 6h post FSS) / 5Hz	Mean shear stress: 0.7Pa, pulse amplitude of 0.3Pa, peak SS of 8.4Pa/s	Custom-made	Decrease (RT-qPCR, GAPDH)	0h post FSS: 1.1/1.4 = 0.8 (ratio-calc)†			
Liao et al. (2017)	EphA2	Epha2	MLO-Y4 (n.g./ n.g. (n.g.), n.g., n.g., n.g., 200000 cells per slide/ 80-90%)	Pulsatile laminar	2h @ 5Hz	0.7±0.3Pa @ 5Hz L-PFF: 4dyn/cm² H-PFF: 16dyn/cm²	Streamer® Shear Stress Device (Flexcell)			Decrease (WB, GAPDH)	No quantitative information given.	L-PFF / H-PFF => reference (Kamel et al. 2010) in this publication
Liao et al. (2017)	EphB4	Ephb4	MLO-Y4 (n.g./ n.g. (n.g.), n.g., n.g., n.g., 200000 cells per slide/ 80-90%)	Pulsatile laminar	2h @ 5Hz	0.7±0.3Pa @ 5Hz L-PFF: 4dyn/cm² H-PFF: 16dyn/cm²	Streamer® Shear Stress Device (Flexcell)			Increase (WB, GAPDH)	No quantitative information given.	
Liao et al. (2017)	EphrinA2	Efna2	MLO-Y4 (n.g./ n.g. (n.g.), n.g., n.g., n.g., 200000 cells per slide/ 80-90%)	Pulsatile laminar	2h @ 5Hz	0.7±0.3Pa @ 5Hz L-PFF: 4dyn/cm² H-PFF: 16dyn/cm²	Streamer® Shear Stress Device (Flexcell)			Decrease (WB, GAPDH)	No quantitative information given.	approved (Primer-BLAST)
Liao et al. (2017)	EphrinB2	Efnb2	MLO-Y4 (n.g./ n.g. (n.g.), n.g., n.g., n.g., 200000 cells per slide/ 80-90%)	Pulsatile laminar	2h @ 5Hz	0.7±0.3Pa @ 5Hz L-PFF: 4dyn/cm² H-PFF: 16dyn/cm²	Streamer® Shear Stress Device (Flexcell)			Increase (WB, GAPDH)	No quantitative information given.	approved (Primer-BLAST)
Liao et al. (2017)	OPG	Tnfrsf11b	MLO-Y4 (n.g./ n.g. (n.g.), n.g., n.g., n.g., 200000 cells per slide/ 80-90%)	Pulsatile laminar	2h @ 5Hz	0.7±0.3Pa @ 5Hz L-PFF: 4dyn/cm² H-PFF: 16dyn/cm²	Streamer® Shear Stress Device (Flexcell)	Increase (RT-qPCR, GAPDH)	L-PFF: 1.6 (rel.)† H-PFF: 1.4 (rel.)†	Increase (ELISA)	L-PFF: 4 (ratio)† H-PFF: 3.0 (ratio)†	
Liao et al. (2017)	p-ERK1/2	Mapk3; Mapk1	MLO-Y4 (n.g./ n.g. (n.g.), n.g., n.g., n.g., 200000 cells per slide/ 80-90%)	Pulsatile laminar	2h @ 5Hz	0.7±0.3Pa @ 5Hz L-PFF: 4dyn/cm² H-PFF: 16dyn/cm²	Streamer® Shear Stress Device (Flexcell)			Increase (WB, GAPDH)	No quantitative information given.	
Liao et al. (2017)	p-STAT3	Stat3	MLO-Y4 (n.g./ n.g. (n.g.), n.g., n.g., n.g., 200000 cells per slide/ 80-90%)	Pulsatile laminar	2h @ 5Hz	0.7±0.3Pa @ 5Hz L-PFF: 4dyn/cm² H-PFF: 16dyn/cm²	Streamer® Shear Stress Device (Flexcell)			Increase (WB, GAPDH)	No quantitative information given.	

[a] Entry provided as reported in the given study.
[b] Human genes were confirmed with the HUGO Gene Nomenclature Committee (HGNC; URL: https://www.genenames.org); mouse genes were confirmed with the Mouse Genome Informatics (MGI; URL: https://www.informatics.jax.org/genes.shtml) after checking the specificity of primers with Primer-BLAST.
[c] Sex of donors: "M" – male, "F" – female; Tooth type: "PM" – premolar, "M" – molar; dig. Indicate isolation by cell digestion; Exp. indicate isolation by cell explant; Cell density: given in cells/cm² if not otherwise mentioned.
[d] Flow type deduced from the description of the FSS apparatus given by the authors.
[f] RT-qPCR (reverse-transcriptase quantitative polymerase chain reaction); sqPCR (semi-quantitative polymerase chain reaction); ELISA (enzyme-linked immuno absorbent assay); WB (western blotting); RIA (radioimmunoassay); EMSA (electromobility shift assay); IF (immunofluorescence)

rel.: indicate relative gene expression. Is entitled to percentages or gene expression ratios normalized to control, and not calculated by ΔΔCT.
FC: indicate fold change. When Author mentions the use of ΔΔCT or the method according to Livak & Schmittken (2004) in calculating FC.
n.g.: not given. For information not given by study-authors.
† Information derived from figures using Engauge Digitizer.
* Indicate manual calculations by measuring the graphs, without using the Engauge Digitizer.
ratio-calc: indicate manual calculation by dividing intervention/control = result (ratio-calc)
ratio: indicate ratios given by study-authors such as normalization to control in case of small molecules data or in case of gene expression ratios, e.g. ratio of RANKL/OPG or Bcl-2/Bax.

Reference	Gene or analyte[a]	Official gene symbol or abbreviation[b]	Cell type (age/ number and sex of donor (health status), tooth type, isolation method, passages used, cell density/confluency)[a,c]	Flow type (Steady laminar, Pulsatile laminar, or Oscillatory laminar)[a,d]	FSS-duration and frequency[a]	FSS-magnitude[a]	FSS apparatus[a]	Gene expression: Increase, decrease, no change (method w/ reference gene); methods: RT-qPCR, sqPCR, northern hybridization[f]	Gene expression: when it reaches peak and peak's magnitude (fold change; relative gene expression; times or ratio; unclear = ?)[f]	Protein expression: Increase, decrease, no change (method w/reference); methods: ELISA, WB, RIA, EMSA, IF[f]	Protein expression: When it reaches peak and peak's magnitude (times or ratio; unclear = ?)[f]	Remarks
Liao et al. (2017)	RANKL	Tnfsf11	MLO-Y4 (n.g./ n.g. (n.g.), n.g., n.g., n.g., 200000 cells per slide/ 80-90%)	Pulsatile laminar	2h @ 5Hz	0.7±0.3Pa @ 5Hz L-PFF: 4dyn/cm² H-PFF: 16dyn/cm²	Streamer® Shear Stress Device (Flexcell)	Increase (RT-qPCR, GAPDH)	L-PFF: 1.2 (rel.)† H-PFF: 1.04 (rel.)†	Decrease (ELISA)	L-PFF: 0.63 (ratio)† H-PFF: 0.47 (ratio)†	
Liao et al. (2017)	RANKL / OPG	ratio (RANKL/OPG)	MLO-Y4 (n.g./ n.g. (n.g.), n.g., n.g., n.g., 200000 cells per slide/ 80-90%)	Pulsatile laminar	2h @ 5Hz	0.7±0.3Pa @ 5Hz L-PFF: 4dyn/cm² H-PFF: 16dyn/cm²	Streamer® Shear Stress Device (Flexcell)	Decrease (RT-qPCR, GAPDH)	L-PFF: 0.76 (ratio)† H-PFF: 0.56 (ratio)†	Decrease (ELISA)	L-PFF: 0.24 (ratio)† H-PFF: 0.36 (ratio)†	
Liao et al. (2017)	TNF-α	Tnf	MLO-Y4 (n.g./ n.g. (n.g.), n.g., n.g., n.g., 200000 cells per slide/ 80-90%)	Pulsatile laminar	2h @ 5Hz	0.7±0.3Pa @ 5Hz L-PFF: 4dyn/cm² H-PFF: 16dyn/cm²	Streamer® Shear Stress Device (Flexcell)	Decrease (RT-qPCR, GAPDH)	L-PFF: 0.92 (rel.)† H-PFF: 0.93 (rel.)†	Decrease (ELISA)	L-PFF: 0.61 (ratio)† H-PFF: 0.25 (ratio)†	
Deepak et al. (2017)	ALP (alkaline phosphatase)	Alpl	MLO-Y4 (n.g./ n.g. (n.g.), n.g., n.g., n.g., 4x10⁴ cells per slide/ n.g.)	Oscillatory laminar	1h @ 0.5Hz (24h post-PFF incubation)	1Pa	Custom-made	Increase (RT-qPCR, GAPDH)	13.2 (FC)†			approved (Primer-BLAST)
Deepak et al. (2017)	Ca++	Calcium	MLO-Y4 (n.g./ n.g. (n.g.), n.g., n.g., n.g., 4x10⁴ cells per slide/ n.g.)	Oscillatory laminar	1h @ 0.5Hz (24h post-PFF incubation)	1Pa	Custom-made			Fluctuated increase (fluorescence microscopy, Fura-2 AM)		
Deepak et al. (2017)	DMP-1 (dentin matrix protein-1)	Dmp1	MLO-Y4 (n.g./ n.g. (n.g.), n.g., n.g., n.g., 4x10⁴ cells per slide/ n.g.)	Oscillatory laminar	1h @ 0.5Hz (24h post-PFF incubation)	1Pa	Custom-made	Increase (RT-qPCR, GAPDH)	2.5 (FC)†			
Deepak et al. (2017)	NO	Nitric oxide	MLO-Y4 (n.g./ n.g. (n.g.), n.g., n.g., n.g., 4x10⁴ cells per slide/ n.g.)	Oscillatory laminar	1h @ 0.5Hz (24h post-PFF incubation)	1Pa	Custom-made			Increase (coloric assay)	257.1/ 102.3 = 2.5 (ratio-calc)†	BioVision coloric assay
Deepak et al. (2017)	NOS	Nos (unspecific)	MLO-Y4 (n.g./ n.g. (n.g.), n.g., n.g., n.g., 4x10⁴ cells per slide/ n.g.)	Oscillatory laminar	1h @ 0.5Hz (24h post-PFF incubation)	1Pa	Custom-made			Increase (Griess, NO_2^-)	145.1/ 99.5 = 1.5 (ratio-calc)†	
Deepak et al. (2017)	OCN (osteocalcin)	Bglap	MLO-Y4 (n.g./ n.g. (n.g.), n.g., n.g., n.g., 4x10⁴ cells per slide/ n.g.)	Oscillatory laminar	1h @ 0.5Hz (24h post-PFF incubation)	1Pa	Custom-made	Increase (RT-qPCR, GAPDH)	12.5 (FC)†			
Deepak et al. (2017)	OPN (osteopontin)	Spp1	MLO-Y4 (n.g./ n.g. (n.g.), n.g., n.g., n.g., 4x10⁴ cells per slide/ n.g.)	Oscillatory laminar	1h @ 0.5Hz (24h post-PFF incubation)	1Pa	Custom-made	Increase (RT-qPCR, GAPDH)	8.4 (FC)†			
Deepak et al. (2017)	PGE2	PGE2	MLO-Y4 (n.g./ n.g. (n.g.), n.g., n.g., n.g., 4x10⁴ cells per slide/ n.g.)	Oscillatory laminar	1h @ 0.5Hz (24h post-PFF incubation)	1Pa	Custom-made			Increase (ELISA)	826.6/391.1 = 2.1 (ratio-calc)†	
Deepak et al. (2017)	Sost (sclerostin)	Sost	MLO-Y4 (n.g./ n.g. (n.g.), n.g., n.g., n.g., 4x10⁴ cells per slide/ n.g.)	Oscillatory laminar	1h @ 0.5Hz (24h post-PFF incubation)	1Pa	Custom-made	Decrease (RT-qPCR, GAPDH)	0.99 (FC)†			
Seref-Ferlengez et al. (2016)	Ca^{2+}	Calcium	MLO-Y4 (n.g./ n.g. (n.g.), n.g., n.g., n.g., n.g./ n.g.)	Oscillatory Laminar	5min @ 1Hz	(OFSS, τ=±10dyn/cm²)	μ-slide VI[0.4] chamber (ibidi GmbH, Germany)			Fluctuated increase (fluorescence microscopy, Fura-2 AM)		
Cherian et al. (2005)	Cx43	Gja1	MLO-Y4 (n.g./ n.g. (n.g.), n.g., n.g., n.g., 2.0×10³, 7.5×10³, 1.6×10⁴, and 3.8×10⁴ cells per cm²/ n.g.)	Steady laminar	30min, 2h / n.g.	16dyn/cm²	Custom-made			biotinylated Cx43/ Total: increase (WB, Total Cx43)<br><br>biotinylated Cx43 bound: increase (WB, β-actin)	2h FF: 0.75/0.35 = 2.14 (ratio-calc)*<br><br>(biotinylated Cx43 bound): No quantitative information given	
Cherian et al. (2005)	PGE2	PGE2	MLO-Y4 (n.g./ n.g. (n.g.), n.g., n.g., n.g., 2.0×10³, 7.5×10³, 1.6×10⁴, and 3.8×10⁴ cells per cm²/ n.g.)	Steady laminar	30min, 2h / n.g.	16dyn/cm²	Custom-made			Intracellular PGE2: increase (EIA) Extracellular PGE2: increase (EIA)	2h FF: Intracellular PGE2: 9.5pg/10⁴ cells; 9.5/1.5 = 6.3 (ratio-calc)† 2h FF: Extracellular PGE2: 10.8pg/10⁴ cells; 10.8/ 1.8 = 6 (ratio-calc)†	

[a] Entry provided as reported in the given study.
[b] Human genes were confirmed with the HUGO Gene Nomenclature Committee (HGNC; URL: https://www.genenames.org); mouse genes were confirmed with the Mouse Genome Informatics (MGI; URL: https://www.informatics.jax.org/genes.shtml) after checking the specificity of primers with Primer-BLAST.
[c] Sex of donors: "M" – male, "F" – female; Tooth type: "PM" – premolar, "M" – molar; dig. Indicate isolation by cell digestion; Exp. indicate isolation by cell explant; Cell density: given in cells/cm² if not otherwise mentioned.
[d] Flow type deduced from the description of the FSS apparatus given by the authors.
f RT-qPCR (reverse-transcriptase quantitative polymerase chain reaction); sqPCR (semi-quantitative polymerase chain reaction); ELISA (enzyme-linked immuno absorbent assay); WB (western blotting); RIA (radioimmunoassay); EMSA (electromobility shift assay); IF (immunofluorescence)

rel.: indicate relative gene expression. Is entitled to percentages or gene expression ratios normalized to control, and not calculated by ΔΔCT.
FC: indicate fold change. When Author mentions the use of ΔΔCT or the method according to Livak & Schmittken (2004) in calculating FC.
n.g.: not given. For information not given by study-authors.
† Information derived from figures using Engauge Digitizer.
* Indicate manual calculations by measuring the graphs, without using the Engauge Digitizer.
ratio-calc: indicate manual calculation by dividing intervention/control = result (ratio-calc)
ratio: indicate ratios given by study-authors such as normalization to control in case of small molecules data or in case of gene expression ratios, e.g. ratio of RANKL/OPG or Bcl-2/Bax.

Reference	Gene or analyte[a]	Official gene symbol or abbreviation[b]	Cell type (age/ number and sex of donor (health status), tooth type, isolation method, passages used, cell density/confluency)[a,c]	Flow type (Steady laminar, Pulsatile laminar, or Oscillatory laminar)[a,d]	FSS-duration and frequency[a]	FSS-magnitude[a]	FSS apparatus[a]	Gene expression: Increase, decrease, no change (method w/ reference gene); methods: RT-qPCR, sqPCR, northern hybridization[f]	Gene expression: when it reaches peak and peak's magnitude (fold change; relative gene expression; times or ratio; unclear = ?)[f]	Protein expression: Increase, decrease, no change (method w/reference); methods: ELISA, WB, RIA, EMSA, IF[f]	Protein expression: When it reaches peak and peak's magnitude (times or ratio; unclear = ?)[f]	Remarks
Riquelme et al. (2021)	p-AKT / total-Akt	Akt1	MLO-Y4 (n.g./ n.g. (n.g.), n.g., n.g., n.g., n.g./ n.g.)	Steady laminar	10min/n.g.	8dyn/cm2	Custom-made			Increase (WB, β-actin)	2.2 (ratio)†	
Riquelme et al. (2021)	SOST	Sost	MLO-Y4 (n.g./ n.g. (n.g.), n.g., n.g., n.g., n.g./ n.g.)	Steady laminar	10min/n.g.	8dyn/cm2	Custom-made			Decrease (WB, β-actin)	0.6 (ratio)†	
Liu et al. (2015)	COX-2	Ptgs2	MLO-Y4 (n.g./ n.g. (n.g.), n.g., n.g., n.g., n.g./ 80%)	Oscillatory laminar	1h (sampling 24h post FSS) / 1Hz	2Pa	Custom-made	Increase (RT-qPCR, 18S)	24h post FSS: 1.1/0.5 = 2.2 (ratio-calc)†			
Liu et al. (2015)	PGE2	PGE2	MLO-Y4 (n.g./ n.g. (n.g.), n.g., n.g., n.g., n.g./ 80%)	Oscillatory laminar	1h (sampling 24h post FSS) / 1Hz	2Pa	Custom-made			Increase (ELISA)	2437.3pg/ml; 2437.3/1469.1 = 1.7 (ratio-calc)†	
Liu et al. (2015)	VEGF	Vegfa	MLO-Y4 (n.g./ n.g. (n.g.), n.g., n.g., n.g., n.g./ 80%)	Oscillatory laminar	1h (sampling 24h post FSS) / 1Hz	2Pa	Custom-made	Increase (RT-qPCR, 18S)	24h post FSS:0.7/0.4 = 1.75 (ratio-calc)†	Increase (ELISA)	24h post FSS: 2.0/0.9 = 2.2 (ratio-calc)†	
Cherian et al. (2003)	cAMP	cyclic_AMP	MLO-Y4 (n.g./ n.g. (n.g.), n.g., n.g., n.g., n.g./ n.g.)	Steady laminar	2h/n.g. (0h, 0.5h, 2h, 24 h post-FF incubation)	16dyn/cm^2	Custom-made			Increase (EIA)	0.5h post-FF: 21.3pmol/mL; 21.3/ 8.7 = 2.4 (ratio-calc)†	Use of FF conditioned medium
Cherian et al. (2003)	Prostaglandin receptor EP2	Ptger2	MLO-Y4 (n.g./ n.g. (n.g.), n.g., n.g., n.g., n.g./ n.g.)	Steady laminar	2h/n.g. (0h, 0.5h, 2h, 24 h post-FF incubation)	16dyn/cm^2	Custom-made	Decrease then increase (Northern blot, actin)	0h post-FF: 0.8/0.9 = 0.89 (ratio-calc)† 24h post-FF: 1.7/0.8 = 2.1 (ratio-calc)†			
Batra et al. (2014)	p-AKT / AKT	Akt1	MLO-Y4 (n.g./ n.g. (n.g.), n.g., n.g., n.g., n.g./ n.g.)	Steady laminar	0.5h, 2h, 4h, 24h)/n.g.	16dyn/cm^2	Custom-made			Increase (WB, β-actin)	0.5h: 13.0 (ratio)†	
Du et al. (2020)	ARRAY		MLO-Y4 (n.g./ n.g. (n.g.), n.g., n.g., n.g., 2×10^5 cells per slide/ 80–90%)	Pulsatile laminar	2h @ 5Hz	4dyn/cm^2	Streamer STR-4000 (FlexCell)	Microarray analysis to Oebiotech (Shanghai, China); Illumina His Eq. 2000 platform				
Du et al. (2020)	Bad	Bad	MLO-Y4 (n.g./ n.g. (n.g.), n.g., n.g., n.g., 2×10^5 cells per slide/ 80–90%)	Pulsatile laminar	2h @ 5Hz	4dyn/cm^2	Streamer STR-4000 (FlexCell)	Decrease (RT-qPCR, GAPDH)	0.45 (FC)†			
Du et al. (2020)	Bax	Bax	MLO-Y4 (n.g./ n.g. (n.g.), n.g., n.g., n.g., 2×10^5 cells per slide/ 80–90%)	Pulsatile laminar	2h @ 5Hz	4dyn/cm^2	Streamer STR-4000 (FlexCell)	Decrease (RT-qPCR, GAPDH)	0.39 (FC)†			
Du et al. (2020)	Lif	Lif	MLO-Y4 (n.g./ n.g. (n.g.), n.g., n.g., n.g., 2×10^5 cells per slide/ 80–90%)	Pulsatile laminar	2h @ 5Hz	4dyn/cm^2	Streamer STR-4000 (FlexCell)	Increase (RT-qPCR, GAPDH)	1.6 (FC)†			
de Castro et al. (2015)	BAX	Bax	MLO-Y4 (n.g./ n.g. (n.g.), n.g., n.g., n.g., n.g./ n.g.)	Pulsatile laminar	10min @ 8Hz	10dyn/cm^2	Flexcell Streamer shear stress device (Flexcell)			Decrease (WB, β-actin)	0.5 (ratio)†	
de Castro et al. (2015)	Bcl2 (anti-apoptotic protein)	Bcl2	MLO-Y4 (n.g./ n.g. (n.g.), n.g., n.g., n.g., n.g./ n.g.)	Pulsatile laminar	10min @ 8Hz	10dyn/cm^2	Flexcell Streamer shear stress device (Flexcell)			Increase (WB, β-actin)	4.4 (ratio)†	
de Castro et al. (2015)	Bcl2 / BAX	ratio (Bcl2/Bax)	MLO-Y4 (n.g./ n.g. (n.g.), n.g., n.g., n.g., n.g./ n.g.)	Pulsatile laminar	10min @ 8Hz	10dyn/cm^2	Flexcell Streamer shear stress device (Flexcell)			Increase (WB, β-actin)	10.5 (ratio)†	
de Castro et al. (2015)	p-Erk / Erk	Mapk3; Mapk1	MLO-Y4 (n.g./ n.g. (n.g.), n.g., n.g., n.g., n.g./ n.g.)	Pulsatile laminar	10min @ 8Hz	10dyn/cm^2	Flexcell Streamer shear stress device (Flexcell)			Increase (WB, total ERK)	2.4 (ratio)†	
de Castro et al. (2015)	VEGF	Vegfa	MLO-Y4 (n.g./ n.g. (n.g.), n.g., n.g., n.g., n.g./ n.g.)	Pulsatile laminar	10min @ 8Hz	10dyn/cm^2	Flexcell Streamer shear stress device (Flexcell)	Increase (RT-qPCR, 18S)	6h: 3.2 (rel.)†			
de Castro et al. (2015)	VEGFR2	Kdr	MLO-Y4 (n.g./ n.g. (n.g.), n.g., n.g., n.g., n.g./ n.g.)	Pulsatile laminar	10min @ 8Hz	10dyn/cm^2	Flexcell Streamer shear stress device (Flexcell)			Increase (WB, α-tubulin)	2.3 (ratio)†	
de Castro et al. (2015)	β-catenin	Ctnnb1	MLO-Y4 (n.g./ n.g. (n.g.), n.g., n.g., n.g., n.g./ n.g.)	Pulsatile laminar	10min @ 8Hz	10dyn/cm^2	Flexcell Streamer shear stress device (Flexcell)			Increase (WB, β-actin)	2.2 (ratio)†	
Yan et al. (2018)	Cx43 (connexin 43)	Gja1	MLO-Y4 (n.g./ n.g. (n.g.), n.g., n.g., n.g., 1×10^5 cells per mL/ n.g.)	Steady laminar	2h @ n.g. (0h, 3h, 6h post-FSS incubation)	1Pa (10dyn/cm^2)	n.g.	Increase (RT-qPCR, GAPDH)	3h post-FSS: 2.42/1.14 = 2.12 (ratio-calc)†	Increase (WB, β-actin)	3h post-FSS: 2.9 (ratio)†	

[a] Entry provided as reported in the given study.
[b] Human genes were confirmed with the HUGO Gene Nomenclature Committee (HGNC; URL: https://www.genenames.org); mouse genes were confirmed with the Mouse Genome Informatics (MGI; URL: https://www.informatics.jax.org/genes.shtml) after checking the specificity of primers with Primer-BLAST.
[c] Sex of donors: "M" – male, "F" – female; Tooth type: "PM" – premolar, "M" – molar; dig. Indicate isolation by cell digestion; Exp. indicate isolation by cell explant; Cell density: given in cells/cm^2 if not otherwise mentioned.
[d] Flow type deduced from the description of the FSS apparatus given by the authors.
f RT-qPCR (reverse-transcriptase quantitative polymerase chain reaction); sqPCR (semi-quantitative polymerase chain reaction); ELISA (enzyme-linked immuno absorbent assay); WB (western blotting); RIA (radioimmunoassay); EMSA (electromobility shift assay); IF (immunofluorescence)

rel.: indicate relative gene expression. Is entitled to percentages or gene expression ratios normalized to control, and not calculated by ΔΔCT.
FC: indicate fold change. When Author mentions the use of ΔΔCT or the method according to Livak & Schmittken (2004) in calculating FC.
n.g.: not given. For information not given by study-authors.
† Information derived from figures using Engauge Digitizer.
* Indicate manual calculations by measuring the graphs, without using the Engauge Digitizer.
ratio-calc: indicate manual calculation by dividing intervention/control = result (ratio-calc)
ratio: indicate ratios given by study-authors such as normalization to control in case of small molecules data or in case of gene expression ratios, e.g. ratio of RANKL/OPG or Bcl-2/Bax.

Reference	Gene or analyte[a]	Official gene symbol or abbreviation[b]	Cell type (age/ number and sex of donor (health status), tooth type, isolation method, passages used, cell density/confluency)[a,c]	Flow type (Steady laminar, Pulsatile laminar, or Oscillatory laminar)[a,d]	FSS-duration and frequency[a]	FSS-magnitude[a]	FSS apparatus[a]	Gene expression: Increase, decrease, no change (method w/ reference gene); methods: RT-qPCR, sqPCR, northern hybridization[f]	Gene expression: when it reaches peak and peak's magnitude (fold change; relative gene expression; times or ratio; unclear = ?)[f]	Protein expression: Increase, decrease, no change (method w/reference); methods: ELISA, WB, RIA, EMSA, IF[f]	Protein expression: When it reaches peak and peak's magnitude (times or ratio; unclear = ?)[f]	Remarks
Yan et al. (2018)	DKK1	Dkk1	MLO-Y4 (n.g./ n.g. (n.g.), n.g., n.g., n.g., 1×10^5 cells per mL/ n.g.)	Steady laminar	2h @ n.g. (0h, 3h, 6h post-FSS incubation)	1Pa (10dyn/cm^2)	n.g.	Temporary increase followed by decrease then increase (RT-qPCR, GAPDH)	0h post-FSS: 1.19/1.01 = 1.178 (ratio-calc)† 3h post-FSS: 0.625/1.02 = 0.612 (ratio-calc)† 6h post-FSS: 1.99/1.01 (ratio-calc)†	Decrease then increase (WB, β-actin)	3h post-FSS: 0.27 (ratio)† 6h post-FSS: 2.4 (ratio)†	
Yan et al. (2018)	OCN	Bglap	MLO-Y4 (n.g./ n.g. (n.g.), n.g., n.g., n.g., 1×10^5 cells per mL/ n.g.)	Steady laminar	2h @ n.g. (0h, 3h, 6h post-FSS incubation)	1Pa (10dyn/cm^2)	n.g.	Increase (RT-qPCR, GAPDH)	3h post-FSS: 2.08/1.02 = 1.97 (ratio-calc)†	Increase (WB, β-actin)	3h post-FSS: 2.4/1.17 = 2 (ratio-calc)†	
Yan et al. (2018)	OPG	Tnfrsf11b	MLO-Y4 (n.g./ n.g. (n.g.), n.g., n.g., n.g., 1×10^5 cells per mL/ n.g.)	Steady laminar	2h @ n.g. (0h, 3h, 6h post-FSS incubation)	1Pa (10dyn/cm^2)	n.g.	Increase with plateau followed by decrease (RT-qPCR, GAPDH)	0h post-FSS: 2.5 (rel.) 3h post-FSS: 2.80/1.01 = 2.77 (ratio-calc)† 6h post-FSS: 0.666/1.04 = 0.64 (ratio-calc)†	Increase with plateau followed by decrease (WB, β-actin)	0h post-FSS: 1.9 (ratio)† 3h post-FSS: 1.8 (ratio)† 6h post-FSS: 0.7 (ratio)†	
Yan et al. (2018)	RANKL	Tnfsf11	MLO-Y4 (n.g./ n.g. (n.g.), n.g., n.g., n.g., 1×10^5 cells per mL/ n.g.)	Steady laminar	2h @ n.g. (0h, 3h, 6h post-FSS incubation)	1Pa (10dyn/cm^2)	n.g.	Increase then decrease with plateau (RT-qPCR, GAPDH)	0h post-FSS: 1.18 (rel.)† 3h post-FSS: 0.452/ 1.02 = 0.44 (ratio-calc)† 6h post-FSS: 0.51/1.02 = 0.5 (ratio-calc) †	Decrease (WB, β-actin)	3h post-FSS: 0.2 (ratio)†	
Yan et al. (2018)	RANKL / OPG	ratio (RANKL/OPG)	MLO-Y4 (n.g./ n.g. (n.g.), n.g., n.g., n.g., 1×10^5 cells per mL/ n.g.)	Steady laminar	2h @ n.g. (0h, 3h, 6h post-FSS incubation)	1Pa (10dyn/cm^2)	n.g.	Decrease (RT-qPCR, GAPDH)	3h post-FSS: 0.17/0.99 = 0.17 (ratio-calc)†	Decrease (WB, β-actin)	3h post-FSS: 0.12 (ratio)†	
Yan et al. (2018)	SOST	Sost	MLO-Y4 (n.g./ n.g. (n.g.), n.g., n.g., n.g., 1×10^5 cells per mL/ n.g.)	Steady laminar	2h @ n.g. (0h, 3h, 6h post-FSS incubation)	1Pa (10dyn/cm^2)	n.g.	Decrease then increase (RT-qPCR, GAPDH)	3h post-FSS: 0.007/0.999 = 0.007 (ratio-calc)† 6h post-FSS:1.65/1.015 = 1.62 (ratio-calc)†			
Yan et al. (2018)	Wnt3a	Wnt3a	MLO-Y4 (n.g./ n.g. (n.g.), n.g., n.g., n.g., 1×10^5 cells per mL/ n.g.)	Steady laminar	2h @ n.g. (0h, 3h, 6h post-FSS incubation)	1Pa (10dyn/cm^2)	n.g.	Increase (RT-qPCR, GAPDH)	3h post-FSS: 1.82/1.07 = 1.7 (ratio-calc)†	Increase (WB, β-actin)	6h post-FSS: 6.2/1.1 = 5.63 (ratio-calc)†	
Yan et al. (2018)	β-catenin	Ctnnb1	MLO-Y4 (n.g./ n.g. (n.g.), n.g., n.g., n.g., 1×10^5 cells per mL/ n.g.)	Steady laminar	2h @ n.g. (0h, 3h, 6h post-FSS incubation)	1Pa (10dyn/cm^2)	n.g.	Increase (RT-qPCR, GAPDH)	0h post-FSS: 1.50/1.04 = 1.44 (ratio-calc)†	Increase (WB, β-actin)	3h post-FSS: 3.4/1.2 = 2.8 (ratio-calc)†	

References

Alford AI, Jacobs CR, Donahue HJ (2003). Oscillating fluid flow regulates gap junction communication in osteocytic MLO-Y4 cells by an ERK1/2 MAP kinase-dependent mechanism. Bone; 33(1):64-70.

Bakker AD, Silva VC, Krishnan R, Bacabac RG, Blaauboer ME, Lin YC, Marcantonio RA, Cirelli JA, Klein-Nulend J (2009). Tumor necrosis factor alpha and interleukin-1beta modulate calcium and nitric oxide signaling in mechanically stimulated osteocytes. Arthritis Rheum; 60(11):3336-45.

Bakker AD, Zandieh-Doulabi B, Klein-Nulend J (2013). Strontium ranelate affects signaling from mechanically-stimulated osteocytes towards osteoclasts and osteoblasts. Bone; 53(1):112-9.

Bakker AD, Kulkarni RN, Klein-Nulend J, Lems WF (2014). IL-6 alters osteocyte signaling toward osteoblasts but not osteoclasts. J Dent Res; 93(4):394-9.

Batra N, Riquelme MA, Burra S, Kar R, Gu S, Jiang JX (2014). Direct regulation of osteocytic connexin 43 hemichannels through AKT kinase activated by mechanical stimulation. J Biol Chem; 289(15):10582-91.

Chen JC, Chua M, Bellon RB, Jacobs CR (2015). Epigenetic changes during mechanically induced osteogenic lineage commitment. J Biomech Eng; 137(2):020902.

Cheng B, Zhao S, Luo J, Sprague E, Bonewald LF, Jiang JX (2001). Expression of functional gap junctions and regulation by fluid flow in osteocyte-like MLO-Y4 cells. J Bone Miner Res; 16(2):249-59.

Cherian PP, Cheng B, Gu S, Sprague E, Bonewald LF, Jiang JX (2003). Effects of mechanical strain on the function of Gap junctions in osteocytes are mediated through the prostaglandin EP2 receptor. J Biol Chem; 278(44):43146-56.

Cherian PP, Siller-Jackson AJ, Gu S, Wang X, Bonewald LF, Sprague E, Jiang JX (2005). Mechanical strain opens connexin 43 hemichannels in osteocytes: a novel mechanism for the release of prostaglandin. Mol Biol Cell; 16(7):3100-6.

[a] Entry provided as reported in the given study.
[b] Human genes were confirmed with the HUGO Gene Nomenclature Committee (HGNC; URL: https://www.genenames.org); mouse genes were confirmed with the Mouse Genome Informatics (MGI; URL: https://www.informatics.jax.org/genes.shtml) after checking the specificity of primers with Primer-BLAST.
[c] Sex of donors: "M" – male, "F" – female; Tooth type: "PM" – premolar, "M" – molar; dig. Indicate isolation by cell digestion; Exp. indicate isolation by cell explant; Cell density: given in cells/cm^2 if not otherwise mentioned.
[d] Flow type deduced from the description of the FSS apparatus given by the authors.
f RT-qPCR (reverse-transcriptase quantitative polymerase chain reaction); sqPCR (semi-quantitative polymerase chain reaction); ELISA (enzyme-linked immuno absorbent assay); WB (western blotting); RIA (radioimmunoassay); EMSA (electromobility shift assay); IF (immunofluorescence)

rel.: indicate relative gene expression. Is entitled to percentages or gene expression ratios normalized to control, and not calculated by ΔΔCT.
FC: indicate fold change. When Author mentions the use of ΔΔCT or the method according to Livak & Schmittken (2004) in calculating FC.
n.g.: not given. For information not given by study-authors.
† Information derived from figures using Engauge Digitizer.
* Indicate manual calculations by measuring the graphs, without using the Engauge Digitizer.
ratio-calc: indicate manual calculation by dividing intervention/control = result (ratio-calc)
ratio: indicate ratios given by study-authors such as normalization to control in case of small molecules data or in case of gene expression ratios, e.g. ratio of RANKL/OPG or Bcl-2/Bax.

de Castro LF, Maycas M, Bravo B, Esbrit P, Gortazar A (2015). VEGF Receptor 2 (VEGFR2) Activation Is Essential for Osteocyte Survival Induced by Mechanotransduction. J Cell Physiol; 230(2):278-85.
Deepak V, Kayastha P, McNamara LM (2017). Estrogen deficiency attenuates fluid flow-induced [Ca(2+)](i) oscillations and mechanoresponsiveness of MLO-Y4 osteocytes. FASEB J; 31(7):3027-3039.
Du J, Yang J, He Z, Cui J, Yang Y, Xu M, Qu X, Zhao N, Yan M, Li H, Yu Z (2020). Osteoblast and Osteoclast Activity Affect Bone Remodeling Upon Regulation by Mechanical Loading-Induced Leukemia Inhibitory Factor Expression in Osteocytes. Frontiers in Molecular Biosciences; 7:585056.
Fahlgren A, Bratengeier C, Semeins CM, Klein-Nulend J, Bakker AD (2018). Supraphysiological loading induces osteocyte-mediated osteoclastogenesis in a novel in vitro model for bone implant loosening. J Orthop Res; 36(5):1425-1434.
Genetos DC, Kephart CJ, Zhang Y, Yellowley CE, Donahue HJ (2007). Oscillating fluid flow activation of gap junction hemichannels induces ATP release from MLO-Y4 osteocytes. J Cell Physiol; 212(1):207-14.
Geoghegan IP, Hoey DA, McNamara LM (2019). Estrogen deficiency impairs integrin α(v)β(3)-mediated mechanosensation by osteocytes and alters osteoclastogenic paracrine signalling. Sci Rep; 9(1):4654.
González Á, García de Durango C, Alonso V, Bravo B, Rodríguez de Gortázar A, Wells A, Forteza J, Vidal-Vanaclocha F (2017). Distinct Osteomimetic Response of Androgen-Dependent and Independent Human Prostate Cancer Cells to Mechanical Action of Fluid Flow: Prometastatic Implications. Prostate; 77(3):321-333.
Govey PM, Jacobs JM, Tilton SC, Loiselle AE, Zhang Y, Freeman WM, Waters KM, Karin NJ, Donahue HJ (2014). Integrative transcriptomic and proteomic analysis of osteocytic cells exposed to fluid flow reveals novel mechano-sensitive signaling pathways. J Biomech; 47(8):1838-45.
Govey PM, Kawasawa YI, Donahue HJ (2015). Mapping the osteocytic cell response to fluid flow using RNA-Seq. J Biomech; 48(16):4327-32.
Haugh MG, Vaughan TJ, McNamara LM (2015). The role of integrin α(V)β(3) in osteocyte mechanotransduction. J Mech Behav Biomed Mater; 42:67-75.
Huang J, Romero-Suarez S, Lara N, Mo C, Kaja S, Brotto L, Dallas SL, Johnson ML, Jähn K, Bonewald LF, Brotto M (2017). Crosstalk between MLO-Y4 osteocytes and C2C12 muscle cells is mediated by the Wnt/β-catenin pathway. JBMR Plus; 1(2):86-100.
Jing D, Lu XL, Luo E, Sajda P, Leong PL, Guo XE (2013). Spatiotemporal properties of intracellular calcium signaling in osteocytic and osteoblastic cell networks under fluid flow. Bone; 53(2):531-40.
Juffer P, Jaspers RT, Lips P, Bakker AD, Klein-Nulend J (2012). Expression of muscle anabolic and metabolic factors in mechanically loaded MLO-Y4 osteocytes. Am J Physiol Endocrinol Metab; 302(4):E389-95.
Kalogeropoulos M, Varanasi SS, Olstad OK, Sanderson P, Gautvik VT, Reppe S, Francis RM, Gautvik KM, Birch MA, Datta HK (2010). Zic1 transcription factor in bone: neural developmental protein regulates mechanotransduction in osteocytes. FASEB J; 24(8):2893-903.
Kamel MA, Picconi JL, Lara-Castillo N, Johnson ML (2010). Activation of β-catenin signaling in MLO-Y4 osteocytic cells versus 2T3 osteoblastic cells by fluid flow shear stress and PGE2: Implications for the study of mechanosensation in bone. Bone; 47(5):872-81.
Kitase Y, Barragan L, Qing H, Kondoh S, Jiang JX, Johnson ML, Bonewald LF (2010). Mechanical induction of PGE2 in osteocytes blocks glucocorticoid-induced apoptosis through both the β-catenin and PKA pathways. J Bone Miner Res; 25(12):2657-68.
Kitase Y, Lee S, Gluhak-Heinrich J, Johnson ML, Harris SE, Bonewald LF (2014). CCL7 is a protective factor secreted by mechanically loaded osteocytes. J Dent Res; 93(11):1108-15.
Kulkarni RN, Bakker AD, Everts V, Klein-Nulend J (2010). Inhibition of osteoclastogenesis by mechanically loaded osteocytes: involvement of MEPE. Calcif Tissue Int; 87(5):461-8.
Kulkarni RN, Bakker AD, Everts V, Klein-Nulend J (2012a). Mechanical loading prevents the stimulating effect of IL-1β on osteocyte-modulated osteoclastogenesis. Biochem Biophys Res Commun; 420(1):11-6.
Kulkarni RN, Bakker AD, Gruber EV, Chae TD, Veldkamp JB, Klein-Nulend J, Everts V (2012b). MT1-MMP modulates the mechanosensitivity of osteocytes. Biochem Biophys Res Commun; 417(2):824-9.
Li J, Rose E, Frances D, Sun Y, You L (2012). Effect of oscillating fluid flow stimulation on osteocyte mRNA expression. J Biomech; 45(2):247-51.
Li X, Liu C, Li P, Li S, Zhao Z, Chen Y, Huo B, Zhang D (2013). Connexin 43 is a potential regulator in fluid shear stress-induced signal transduction in osteocytes. J Orthop Res; 31(12):1959-65.
Li X, Han L, Nookaew I, Mannen E, Silva MJ, Almeida M, Xiong J (2019). Stimulation of Piezo1 by mechanical signals promotes bone anabolism. Elife; 8.
Liao C, Cheng T, Wang S, Zhang C, Jin L, Yang Y (2017). Shear stress inhibits IL-17A-mediated induction of osteoclastogenesis via osteocyte pathways. Bone; 101:10-20.
Litzenberger JB, Kim JB, Tummala P, Jacobs CR (2010). Beta1 integrins mediate mechanosensitive signaling pathways in osteocytes. Calcif Tissue Int; 86(4):325-32.
Liu C, Zhang X, Wu M, You L (2015). Mechanical loading up-regulates early remodeling signals from osteocytes subjected to physical damage. J Biomech; 48(16):4221-8.
Lu XL, Huo B, Chiang V, Guo XE (2012a). Osteocytic network is more responsive in calcium signaling than osteoblastic network under fluid flow. J Bone Miner Res; 27(3):563-74.
Lu XL, Huo B, Park M, Guo XE (2012b). Calcium response in osteocytic networks under steady and oscillatory fluid flow. Bone; 51(3):466-73.
Maycas M, Ardura JA, de Castro LF, Bravo B, Gortázar AR, Esbrit P (2015). Role of the Parathyroid Hormone Type 1 Receptor (PTH1R) as a Mechanosensor in Osteocyte Survival. J Bone Miner Res; 30(7):1231-44.
Maycas M, Portolés MT, Matesanz MC, Buendía I, Linares J, Feito MJ, Arcos D, Vallet-Regí M, Plotkin LI, Esbrit P, Gortázar AR (2017). High glucose alters the secretome of mechanically stimulated osteocyte-like cells affecting osteoclast precursor recruitment and differentiation. J Cell Physiol; 232(12):3611-3621.
Middleton K, Kondiboyina A, Borrett M, Cui Y, Mei X, You L (2018). Microfluidics approach to investigate the role of dynamic similitude in osteocyte mechanobiology. J Orthop Res; 36(2):663-671.
Rath AL, Bonewald LF, Ling J, Jiang JX, Van Dyke ME, Nicolella DP (2010). Correlation of cell strain in single osteocytes with intracellular calcium, but not intracellular nitric oxide, in response to fluid flow. J Biomech; 43(8):1560-4.
Reilly GC, Haut TR, Yellowley CE, Donahue HJ, Jacobs CR (2003). Fluid flow induced PGE2 release by bone cells is reduced by glycocalyx degradation whereas calcium signals are not. Biorheology; 40(6):591-603.
Ren J, Wang XH, Wang GC, Wu JH (2013). 17β estradiol regulation of connexin 43-based gap junction and mechanosensitivity through classical estrogen receptor pathway in osteocyte-like MLO-Y4 cells. Bone; 53(2):587-96.
Riquelme MA, Gu S, Hua R, Jiang JX (2021). Mechanotransduction via the coordinated actions of integrins, PI3K signaling and Connexin hemichannels. Bone Res; 9(1):8.
Santos A, Bakker AD, Zandieh-Doulabi B, Semeins CM, Klein-Nulend J (2009). Pulsating fluid flow modulates gene expression of proteins involved in Wnt signaling pathways in osteocytes. J Orthop Res; 27(10):1280-7.
Santos A, Bakker AD, Zandieh-Doulabi B, de Blieck-Hogervorst JM, Klein-Nulend J (2010). Early activation of the beta-catenin pathway in osteocytes is mediated by nitric oxide, phosphatidyl inositol-3 kinase/Akt, and focal adhesion kinase. Biochem Biophys Res Commun; 391(1):364-9.
Seref-Ferlengez Z, Maung S, Schaffler MB, Spray DC, Suadicani SO, Thi MM (2016). P2X7R-Panx1 Complex Impairs Bone Mechanosignaling under High Glucose Levels Associated with Type-1 Diabetes. PLoS One; 11(5):e0155107.
Shah KM, Orton P, Mani N, Wilkinson JM, Gartland A (2017). Osteocyte physiology and response to fluid shear stress are impaired following exposure to cobalt and chromium: Implications for bone health following joint replacement. J Orthop Res; 35(8):1716-1723.
Thi MM, Kojima T, Cowin SC, Weinbaum S, Spray DC (2003). Fluid shear stress remodels expression and function of junctional proteins in cultured bone cells. Am J Physiol Cell Physiol; 284(2):C389-403.
Thi MM, Suadicani SO, Spray DC (2010). Fluid flow-induced soluble vascular endothelial growth factor isoforms regulate actin adaptation in osteoblasts. J Biol Chem; 285(40):30931-41.
Wang S, Li S, Hu M, Huo B (2019). Calcium response in bone cells at different osteogenic stages under unidirectional or oscillatory flow. Biomicrofluidics; 13(6):064117.
Xia X, Batra N, Shi Q, Bonewald LF, Sprague E, Jiang JX (2010). Prostaglandin promotion of osteocyte gap junction function through transcriptional regulation of connexin 43 by glycogen synthase kinase 3/beta-catenin signaling. Mol Cell Biol; 30(1):206-19.
Xu H, Zhang J, Wu J, Guan Y, Weng Y, Shang P (2012). Oscillatory fluid flow elicits changes in morphology, cytoskeleton and integrin-associated molecules in MLO-Y4 cells, but not in MC3T3-E1 cells. Biol Res; 45(2):163-9.
Xu H, Guan Y, Wu J, Zhang J, Duan J, An L, Shang P (2014). Polycystin 2 is involved in the nitric oxide production in responding to oscillating fluid shear in MLO-Y4 cells. J Biomech; 47(2):387-91.
Yan Z, Wang P, Wu J, Feng X, Cai J, Zhai M, Li J, Liu X, Jiang M, Luo E, Jing D (2018). Fluid shear stress improves morphology, cytoskeleton architecture, viability, and regulates cytokine expression in a time-dependent manner in MLO-Y4 cells. Cell Biol Int; 42(10):1410-1422.
Zhang JN, Zhao Y, Liu C, Han ES, Yu X, Lidington D, Bolz SS, You L (2015). The role of the sphingosine-1-phosphate signaling pathway in osteocyte mechanotransduction. Bone; 79:71-8.
Zhang K, Barragan-Adjemian C, Ye L, Kotha S, Dallas M, Lu Y, Zhao S, Harris M, Harris SE, Feng JQ, Bonewald LF (2006). E11/gp38 selective expression in osteocytes: regulation by mechanical strain and role in dendrite elongation. Mol Cell Biol; 26(12):4539-52.

[a] Entry provided as reported in the given study.
[b] Human genes were confirmed with the HUGO Gene Nomenclature Committee (HGNC; URL: https://www.genenames.org); mouse genes were confirmed with the Mouse Genome Informatics (MGI; URL: https://www.informatics.jax.org/genes.shtml) after checking the specificity of primers with Primer-BLAST.
[c] Sex of donors: "M" – male, "F" – female; Tooth type: "PM" – premolar, "M" – molar; dig. Indicate isolation by cell digestion; Exp. indicate isolation by cell explant; Cell density: given in cells/cm^2 if not otherwise mentioned.
[d] Flow type deduced from the description of the FSS apparatus given by the authors.
f RT-qPCR (reverse-transcriptase quantitative polymerase chain reaction); sqPCR (semi-quantitative polymerase chain reaction); ELISA (enzyme-linked immuno absorbent assay); WB (western blotting); RIA (radioimmunoassay); EMSA (electromobility shift assay); IF (immunofluorescence)

rel.: indicate relative gene expression. Is entitled to percentages or gene expression ratios normalized to control, and not calculated by ΔΔCT.
FC: indicate fold change. When Author mentions the use of ΔΔCT or the method according to Livak & Schmittken (2004) in calculating FC.
n.g.: not given. For information not given by study-authors.
† Information derived from figures using Engauge Digitizer.
* Indicate manual calculations by measuring the graphs, without using the Engauge Digitizer.
ratio-calc: indicate manual calculation by dividing intervention/control = result (ratio-calc)
ratio: indicate ratios given by study-authors such as normalization to control in case of small molecules data or in case of gene expression ratios, e.g. ratio of RANKL/OPG or Bcl-2/Bax.
