# Supplementary material for: Fluid flow shear stress and tissue remodeling—an orthodontic perspective: evidence synthesis and differential gene expression network analysis
Source: Front Bioeng Biotechnol. 2023 Sep 18;11:1256825. doi: 10.3389/fbioe.2023.1256825 (PMC10545883; doi:10.3389/fbioe.2023.1256825)
Supplement: Supplementary file 3 [file DataSheet4.pdf]

Supplement 4

"Fluid Flow Shear Stress and Tissue Remodeling – an Orthodontic Perspective: Evidence Synthesis and Differential Gene Expression Network analysis"

Summary tables for the most used fluid flow apparatuses, shear stress magnitudes, and time durations

Table of Contents

4.1 Fluid flow chambers used in relation to fluid flow profile 2
  4.1.1 Mouse osteoblasts 2
  4.1.2 Mouse osteocytes 2
  4.1.3 Human MSCs 3
  4.1.4 Human osteoblasts 3
  4.1.5 Human osteocytes 3
  4.1.6 Human PDLCs 3
4.2 Fluid flow profiles used in each cell group 4
4.3 Fluid flow chambers used in each cell group 4
4.4 Most frequently used fluid flow shear stress magnitudes and its duration 5
  4.4.1 Human MSCs 5
  4.4.2 Human Osteoblasts 6
  4.4.3 Human PDLCs 7
  4.4.5 Mouse osteoblasts 8
  4.4.6 Mouse osteocytes 9
References 11

4.1 Fluid flow chambers used in relation to fluid flow profile

4.1.1 Mouse osteoblasts

Chamber	Total	Flow type				Reference
		Steady laminar	Pulsatile laminar	Oscillatory laminar	Unknown	
Custom-made	11	3	6	2	0	Bakker et al. (2001); Bakker et al. (2003a); Bakker et al. (2003b); Castillo et al. (2014); Igwe et al. (2009); Klein-Nulend et al. (1996); Klein-Nulend et al. (1997); Mehrotra et al. (2006); Soejima et al. (2001); Yang et al. (2010); Yang et al. (2015);
Rocking culture system	1	0	0	1	0	Kido et al. (2009)
Cytodyne	4	3	1	0	0	Kapur et al. (2010); Lau et al. (2006); Rangaswami et al. (2012); Thi et al. (2012)
IBIDI	2	1	1	0	0	Bakker et al. (2013a); Fu et al. (2008)
Bioflux system	1	1	0	0	0	Suzuki et al. (2013)
Not given	3	1	1	1	0	Callewaert et al. (2010); Li et al. (2005); Xing et al. (2014)
Total	22	9	9	4	0	
Total number of experiments		22				

4.1.2 Mouse osteocytes

Chamber	Total	Flow type				Reference
		Steady laminar	Pulsatile laminar	Oscillatory laminar	Unknown	
Custom-made	34 (number of flow profiles is 35)	10	10	15	0	Alford et al. (2003); Bakker et al. (2009); Bakker et al. (2013b); Bakker et al. (2014); Batra et al. (2014); Cheng et al. (2001); Cherian et al. (2003); Cherian et al. (2005); Deepak et al. (2017); Fahlgren et al. (2018); Genetos et al. (2007); Geoghegan et al. (2019); Govey et al. (2014); Haugh et al. (2015); Juffer et al. (2012); Kulkarni et al. (2010); Kulkarni et al. (2012a); Kulkarni et al. (2012b); Li et al. (2012); Li et al. (2013); Litzenberger et al. (2010); Liu et al. (2015); Lu et al. (2012a); Lu et al. (2012b); Middleton et al. (2018); Reilly et al. (2003); Riquelme et al. (2021); Santos et al. (2009); Santos et al. (2010); Wang et al. (2019); Xia et al. (2010); Xu et al. (2012); Xu et al. (2014); Zhang et al. (2015)
Rocking culture system	1	0	0	1	0	Chen et al. (2015)
Flexcell® Streamer® (Flexcell Inc.)	8	1	7	0	0	de Castro et al. (2015); Du et al. (2020); González et al. (2017); Kamel et al. (2010); Liao et al. (2017); Maycas et al. (2015); Maycas et al. (2017); Zhang et al. (2006);
IBIDI	2	0	0	2	0	Li et al. (2019); Seref-Ferlengez et al. (2016)
Rotational orbital shaker	1	0	0	1	0	Kalogeropoulos et al. (2010)
Cytodyne	2	1	1	0	0	Thi et al. (2003); Thi et al. (2010)
PeCon parallel plate	1	0	0	1	0	Ren et al. (2013)
Focht Chamber System 2	1	0	1	0	0	Wolf et al. (2013)
Not given	7	3	0	1	3	Govey et al. (2015); Huang et al. (2017); Jing et al. (2013); Kitase et al. (2010); Kitase et al. (2014); Shah et al. (2017); Yan et al. (2018)
Total	57	15	19	21	3	
Total number of experiments		58				

4.1.3 Human MSCs

Chamber	Total	Flow type				Reference
		Steady laminar	Pulsatile laminar	Oscillatory laminar	Unknown	
Custom-made	12	5	2	5	0	Charoenpong et al. (2019); Hoey et al. (2012); Kim et al. (2011); Kraft et al. (2010); Kraft et al. (2011); Kuo et al. (2015); Li et al. (2004); Riddle et al. (2006); Riddle et al. (2007); Sonam et al. (2016); Yourek et al. (2010); Yuan et al. (2012);
Rocking culture system	1	0	0	1	0	Lim et al. (2013)
Flexcell Inc. (Streamer, Streamer Gold, FlexFlo)	4	1	0	2	1	Celil Aydemir et al. (2007); Celil Aydemir et al. (2010); Glossop and Cartmell (2009); Hu et al. (2017)
IBIDI	2	2	0	0	0	Becquart et al. (2016); Lee et al. (2017)
Rotational orbital shaker	1	0	0	1	0	Lim et al. (2014)
Not given	1	0	0	1	0	Salvi et al. (2010)
Total	21	8	2	10	1	
Total number of experiments		21				

4.1.4 Human osteoblasts

Chamber	Total	Flow type				Reference
		Steady laminar	Pulsatile laminar	Oscillatory laminar	Unknown	
Custom-made	10	0	10	0	0	Bakker et al. (2003b); Bakker et al. (2004); Joldersma et al. (2000); Joldersma et al. (2001); Klein-Nulend et al. (1998); Klein-Nulend et al. (2002); McGarry et al. (2005); Santos et al. (2011); Sterck et al. (1998); van der Meijden et al. (2016);
Rocking culture system	1	0	0	1	0	Ehnert et al. (2017)
Cytodyne	2	2	0	0	0	Rangaswami et al. (2009); Rangaswami et al. (2012)
Rotational orbital shaker	1	0	0	1	0	Aisha et al. (2015)
Total	14	2	10	2	0	
Total number of experiments		14				

4.1.5 Human osteocytes

Chamber	Total	Flow type				Reference
		Steady laminar	Pulsatile laminar	Oscillatory laminar	Unknown	
Custom-made	1	0	1	0	0	Pathak et al. (2015)

4.1.6 Human PDLCs

Chamber	Total	Flow type				Reference
		Steady laminar	Pulsatile laminar	Oscillatory laminar	Unknown	
Custom-made	7	5	2	0	0	Maeda et al. (2007); Qi and Zhang (2014); Tang et al. (2014); van der Pauw et al. (2000); Zheng et al. (2012); Zheng et al. (2016); Zheng et al. (2019)

4.2 Fluid flow profiles used in each cell group

Cell type	No. of studies	Steady laminar	Pulsatile laminar	Oscillatory laminar	Unknown	No. of flow profiles used
Mouse osteocytes	57	15	19	21	3	58
Human MSCs	21	8	2	10	1	21
Mouse osteoblasts	22	9	9	4	0	22
Human osteoblasts	14	2	10	2	0	14
Human PDL	7	5	2	0	0	7
Human osteocytes	1	0	1	0	0	1
**Total**	**122**	**39**	**43**	**37**	**4**	**123**

4.3 Fluid flow chambers used in each cell group

Chamber	Cell type						Total	Fluid flow type
	Mouse osteocytes	Human MSCs	Mouse osteoblasts	Human osteoblasts	Human PDL	Human osteocytes		
Custom-made	35	12	11	10	7	1	76	Steady laminar, oscillatory laminar, pulsatile laminar
Rocking culture system	1	1	1	1	0	0	4	Oscillatory laminar
Streamer, Streamer Gold, or FlexFlo (FlexCell Inc.)	8	4	0	0	0	0	12	Steady laminar, oscillatory laminar, pulsatile laminar
IBIDI	2	2	2	0	0	0	6	Steady laminar, oscillatory laminar, pulsatile laminar
Rotational orbital shaker	1	1	0	1	0	0	3	Oscillatory laminar
Cytodyne	2	0	4	2	0	0	8	Steady laminar, pulsatile laminar
PeCon parallel plate (PeCon GmbH)	1	0	0	0	0	0	1	Oscillatory laminar
Focht Chamber System 2 (	1	0	0	0	0	0	1	Pulsatile
Bioflux system	0	0	1	0	0	0	1	Steady laminar
Not Given	7	1	3	0	0	0	11	Laminar, oscillatory laminar, pulsatile laminar
**Total**	**58**	**21**	**22**	**14**	**7**	**1**	**123**	

4.4 Most frequently used fluid flow shear stress magnitudes and its duration

4.4.1 Human MSCs

Most frequently used time durations among different studies in relation to fluid flow profile regardless of shear stress magnitudes used.

Reference	Type of flow	FSS duration (h)
Yourek et al. (2010)	Steady laminar	24
Hu et al. (2017)	Steady laminar	24
Kim et al. (2011)	Steady laminar	24
Hu et al. (2017)	Steady laminar	6
Lee et al. (2017)	Steady laminar	6
Charoenpong et al. (2019)	Steady laminar	6
Yuan et al. (2012)	Steady laminar	0.5
Cui et al. (2014)	Steady laminar	0.5
Becquart et al. (2016)	Steady laminar	0.5
Lim et al. (2013)	Oscillatory laminar	2
Hoey et al. (2012)	Oscillatory laminar	2
Lim et al. (2014)	Oscillatory laminar	2
Li et al. (2004)	Oscillatory laminar	2
Lim et al. (2013)	Oscillatory laminar	1
Kuo et al. (2015)	Oscillatory laminar	1
Lim et al. (2014)	Oscillatory laminar	1
Salvi et al. (2010)	Oscillatory laminar	0.05
Riddle et al. (2006)	Oscillatory laminar	0.05
Riddle et al. (2007)	Oscillatory laminar	0.05
Glossop and Cartmell (2009)	Pulsatile laminar	1
Kraft et al. (2011)	Pulsatile laminar	1
Kraft et al. (2010)	Pulsatile laminar	1

Most used shear stress magnitudes in relation to fluid flow profile and time duration.

FSS (dyn/cm²)	No. of studies	Common duration (h)	Reference
Steady laminar fluid flow			
15	2	6	Lee et al. (2017)
		24	Yourek et al. (2010)
10	2	24	Kim et al. (2011)
		48	Sonam et al. (2016)
2	2	2	Charoenpong et al. (2019); Yuan et al. (2012)
Oscillatory laminar fluid flow			
20	3	0.05	Riddle et al. (2006) ; Riddle et al. (2007); Salvi et al. (2010)
10	4	0.05	Riddle et al. (2007); Salvi et al. (2010)
		2	Hoey et al. (2012) ; Li et al. (2004)
5	2	0.05	Riddle et al. (2007); Salvi et al. (2010)
Pulsatile laminar fluid flow			
6	2	1	Kraft et al. (2010); Kraft et al. (2011)

4.4.2 Human Osteoblasts

Most frequently used time durations among different studies in relation to fluid flow profile regardless of shear stress magnitudes used.

Reference	Type of flow	FSS duration (h)
Bakker et al. (2004)	Pulsatile laminar	1
McGarry et al. (2005)	Pulsatile laminar	1
Sterck et al. (1998)	Pulsatile laminar	1
Bakker et al. (2003b)	Pulsatile laminar	1
Joldersma et al. (2000)	Pulsatile laminar	1
Joldersma et al. (2001)	Pulsatile laminar	1
Klein-Nulend et al. (2002)	Pulsatile laminar	1
Santos et al. (2011)	Pulsatile laminar	1
van der Meijden et al. (2016)	Pulsatile laminar	1
Klein-Nulend et al. (1998)	Pulsatile laminar	1

Most used shear stress magnitudes in relation to fluid flow profile and time duration.

Pulsatile laminar fluid flow			Reference
FSS (dyn/cm^2)	No. of studies	Common duration (h)	
10	2	1	Klein-Nulend et al. (2002) ; Santos et al. (2011)
9	3	1	Bakker et al. (2004) ; Joldersma et al. (2001); McGarry et al. (2005)
7	4	1	Joldersma et al. (2000) ; Klein-Nulend et al. (1998); Sterck et al. (1998) ; van der Meijden et al. (2016) ;
4	3	1	Bakker et al. (2004) ; Klein-Nulend et al. (2002) ; Santos et al. (2011)
3	3	1	Bakker et al. (2004) ; Joldersma et al. (2001); McGarry et al. (2005)

4.4.3 Human PDLCs

Most frequently used time durations among different studies in relation to fluid flow profile *__regardless__* *of shear stress magnitudes used.*

Reference	Type of flow	FSS duration (h)
Zheng et al. (2016)	Steady laminar	4
Zheng et al. (2019)	Steady laminar	4
Zheng et al. (2012)	Steady laminar	4
Zheng et al. (2016)	Steady laminar	2
Zheng et al. (2019)	Steady laminar	2
Tang et al. (2014)	Steady laminar	2
Zheng et al. (2012)	Steady laminar	2

Most used shear stress magnitudes in relation to fluid flow profile and time duration.

Steady laminar fluid flow			Reference
FSS (dyn/cm^2)	No. of studies	Common duration (h)	
12	2	2	Tang et al. (2014) ; Zheng et al. (2012)
9	2	6	Qi and Zhang (2014)
		2, 4, 8, 12	Zheng et al. (2012)
6	3	12	Zheng et al. (2012); Zheng et al. (2016)
		8	Zheng et al. (2012); Zheng et al. (2016)
		4	Zheng et al. (2012); Zheng et al. (2016) ; Zheng et al. (2019)
		2	Zheng et al. (2012); Zheng et al. (2016) ; Zheng et al. (2019)

4.4.5 Mouse osteoblasts

Most frequently used time durations among different studies in relation to fluid flow profile <u>regardless</u> of shear stress magnitudes used.

Reference	Type of flow	FSS duration (h)
Yang et al. (2015)	Steady laminar	1
Fu et al. (2008)	Steady laminar	1
Li et al. (2005)	Steady laminar	1
Mehrotra et al. (2006)	Steady laminar	1
Li et al. (2005)	Steady laminar	0.5
Mehrotra et al. (2006)	Steady laminar	0.5
Kapur et al. (2010)	Steady laminar	0.5
Igwe et al. (2009)	Steady laminar	0.5
Lau et al. (2006)	Steady laminar	0.5
Rangaswami et al. (2012)	Steady laminar	0.16
Mehrotra et al. (2006)	Steady laminar	0.16
Suzuki et al. (2013)	Steady laminar	0.16
Yang et al. (2010)	Oscillatory laminar	1
Castillo et al. (2014)	Oscillatory laminar	1
Kido et al. (2009)	Oscillatory laminar	1
Yang et al. (2010)	Oscillatory laminar	0.5
Castillo et al. (2014)	Oscillatory laminar	0.5
Kido et al. (2009)	Oscillatory laminar	0.5
Bakker et al. (2003b)	Pulsatile laminar	1
Callewaert et al. (2010)	Pulsatile laminar	1
Klein-Nulend et al. (1997)	Pulsatile laminar	1
Thi et al. (2012)	Pulsatile laminar	1
Klein-Nulend et al. (1996)	Pulsatile laminar	1
Bakker et al. (2003a)	Pulsatile laminar	0.5
Callewaert et al. (2010)	Pulsatile laminar	0.5
Bakker et al. (2013a)	Pulsatile laminar	0.5
Soejima et al. (2001)	Pulsatile laminar	0.5

Most used shear stress magnitudes in relation to fluid flow profile and time duration.

FSS (dyn/cm^2)	No. of studies	Common duration (h)	Reference
Steady laminar fluid flow			
20	2	0.5	Kapur et al. (2010) ; Lau et al. (2006)
12	3	1	Fu et al. (2008) ; Li et al. (2005); Yang et al. (2015)
10	2	0.5	Igwe et al. (2009); Mehrotra et al. (2006)
Pulsatile laminar fluid flow			
10	2	0.5	Bakker et al. (2013a)
		1	Thi et al. (2012)
6	3	1	Bakker et al. (2003b) ; Callewaert et al. (2010)
		0.5	Bakker et al. (2003a) ; Callewaert et al. (2010)
		0.083	Bakker et al. (2003a) ; Callewaert et al. (2010)

4.4.6 Mouse osteocytes

Most frequently used time durations among different studies in relation to fluid flow profile <u>regardless</u> of shear stress magnitudes used.

Reference	Type of flow	FSS duration (h)
Du et al. (2020)	Steady laminar	2
Yan et al. (2018)	Steady laminar	2
Thi et al. (2003)	Steady laminar	2
Zhang et al. (2006)	Steady laminar	2
Li et al. (2013)	Steady laminar	2
Middleton et al. (2018)	Steady laminar	2
Cheng et al. (2001)	Steady laminar	2
Cherian et al. (2003)	Steady laminar	2
Batra et al. (2014)	Steady laminar	2
Xia et al. (2010)	Steady laminar	2
Kitase et al. (2010)	Steady laminar	2
Shah et al. (2017)	Steady laminar	0.5
Li et al. (2013)	Steady laminar	0.5
Cherian et al. (2005)	Steady laminar	0.5
Batra et al. (2014)	Steady laminar	0.5
Xia et al. (2010)	Steady laminar	0.5
Zhang et al. (2015)	Oscillatory laminar	2
Govey et al. (2015)	Oscillatory laminar	2
Xu et al. (2012)	Oscillatory laminar	2
Govey et al. (2014)	Oscillatory laminar	2
Litzenberger et al. (2010)	Oscillatory laminar	2
Li et al. (2012)	Oscillatory laminar	2
Xu et al. (2014)	Oscillatory laminar	2
Ren et al. (2013)	Oscillatory laminar	2
Deepak et al. (2017)	Oscillatory laminar	1
Geoghegan et al. (2019)	Oscillatory laminar	1
Kalogeropoulos et al. (2010)	Oscillatory laminar	1
Reilly et al. (2003)	Oscillatory laminar	1
Li et al. (2012)	Oscillatory laminar	1
Haugh et al. (2015)	Oscillatory laminar	1
Liu et al. (2015)	Oscillatory laminar	1
Alford et al. (2003)	Oscillatory laminar	1
Bakker et al. (2014)	Pulsatile laminar	1
Kulkarni et al. (2012b)	Pulsatile laminar	1
Santos et al. (2009)	Pulsatile laminar	1
Fahlgren et al. (2018)	Pulsatile laminar	1
Bakker et al. (2013b)	Pulsatile laminar	1
Kulkarni et al. (2010)	Pulsatile laminar	1
Juffer et al. (2012)	Pulsatile laminar	1
Kulkarni et al. (2012a)	Pulsatile laminar	1
Bakker et al. (2009)	Pulsatile laminar	0.5
González et al. (2017)	Pulsatile laminar	0.5
Santos et al. (2010)	Pulsatile laminar	0.5

Most used shear stress magnitudes in relation to fluid flow profile and time duration.

FSS (dyn/cm^2)	No. of studies	Common duration (h)	Reference
Steady laminar fluid flow			
40	2	0.15	Lu et al. (2012a)
		0.16	Jing et al. (2013)
20	2	0.15	Lu et al. (2012a); Lu et al. (2012b)
16	8	24	Batra et al. (2014); Li et al. (2013)
		4	Batra et al. (2014) ; Li et al. (2013) ; Xia et al. (2010)
		2	Batra et al. (2014) ; Cheng et al. (2001) ; Cherian et al. (2003) ; Kitase et al. (2010); Li et al. (2013) ; Xia et al. (2010) ; Zhang et al. (2006)
		1	Li et al. (2013); Shah et al. (2017)
		0.5	Batra et al. (2014) ; Cherian et al. (2005) ; Li et al. (2013) ; Shah et al. (2017) ; Xia et al. (2010)
10	4	0.16	Jing et al. (2013); Maycas et al. (2017)
		2	Middleton et al. (2018); Yan et al. (2018)
8	2	n.g.	Rath et al. (2010)
		0.16	Riquelme et al. (2021)
5	3	1, 2, 3	Thi et al. (2003)
		0.15	Lu et al. (2012a)
		0.16	Jing et al. (2013)
4	2	2	Du et al. (2020) ; Zhang et al. (2006)
Oscillatory Laminar fluid flow			
20	5	0.15	Lu et al. (2012b); Wang et al. (2019)
		1	Li et al. (2012) ; Liu et al. (2015); Reilly et al. (2003)
10	12	2	Govey et al. (2014) ; Govey et al. (2015) ; Li et al. (2012) ; Litzenberger et al. (2010) ; Xu et al. (2012) ; Xu et al. (2014); Zhang et al. (2015)
		1	Alford et al. (2003); Deepak et al. (2017) ; Geoghegan et al. (2019) ; Haugh et al. (2015) ; Li et al. (2012) ; Reilly et al. (2003)
Pulsatile laminar fluid flow			
16	2	2	Kamel et al. (2010); Liao et al. (2017)
10	7	1	Bakker et al. (2013b); Bakker et al. (2014)
		0.5	Bakker et al. (2009) ; González et al. (2017) ; Santos et al. (2010)
		0.16	de Castro et al. (2015); Maycas et al. (2015)
7	4	1	Kulkarni et al. (2010) ; Kulkarni et al. (2012a); Kulkarni et al. (2012b) ; Santos et al. (2009)
4	6	2	Kamel et al. (2010); Liao et al. (2017)
		1	Bakker et al. (2013b); Bakker et al. (2014)
		0.5	Bakker et al. (2009) ; Santos et al. (2010)

References

Aisha MD, Nor-Ashikin MN, Sharaniza AB, Nawawi H, Froemming GR (2015). Orbital fluid shear stress promotes osteoblast metabolism, proliferation and alkaline phosphates activity in vitro. Exp Cell Res; 337(1):87-93.

Alford AI, Jacobs CR, Donahue HJ (2003). Oscillating fluid flow regulates gap junction communication in osteocytic MLO-Y4 cells by an ERK1/2 MAP kinase-dependent mechanism. Bone; 33(1):64-70.

Bakker A, Klein-Nulend J, Burger E (2004). Shear stress inhibits while disuse promotes osteocyte apoptosis. Biochem Biophys Res Commun; 320(4):1163-8.

Bakker AD, Soejima K, Klein-Nulend J, Burger EH (2001). The production of nitric oxide and prostaglandin E(2) by primary bone cells is shear stress dependent. J Biomech; 34(5):671-7.

Bakker AD, Joldersma M, Klein-Nulend J, Burger EH (2003a). Interactive effects of PTH and mechanical stress on nitric oxide and PGE2 production by primary mouse osteoblastic cells. Am J Physiol Endocrinol Metab; 285(3):E608-13.

Bakker AD, Klein-Nulend J, Burger EH (2003b). Mechanotransduction in bone cells proceeds via activation of COX-2, but not COX-1. Biochem Biophys Res Commun; 305(3):677-83.

Bakker AD, Silva VC, Krishnan R, Bacabac RG, Blaauboer ME, Lin YC, Marcantonio RA, Cirelli JA, Klein-Nulend J (2009). Tumor necrosis factor alpha and interleukin-1beta modulate calcium and nitric oxide signaling in mechanically stimulated osteocytes. Arthritis Rheum; 60(11):3336-45.

Bakker AD, Huesa C, Hughes A, Aspden RM, van't Hof RJ, Klein-Nulend J, Helfrich MH (2013a). Endothelial nitric oxide synthase is not essential for nitric oxide production by osteoblasts subjected to fluid shear stress in vitro. Calcif Tissue Int; 92(3):228-39.

Bakker AD, Zandieh-Doulabi B, Klein-Nulend J (2013b). Strontium ranelate affects signaling from mechanically-stimulated osteocytes towards osteoclasts and osteoblasts. Bone; 53(1):112-9.

Bakker AD, Kulkarni RN, Klein-Nulend J, Lems WF (2014). IL-6 alters osteocyte signaling toward osteoblasts but not osteoclasts. J Dent Res; 93(4):394-9.

Batra N, Riquelme MA, Burra S, Kar R, Gu S, Jiang JX (2014). Direct regulation of osteocytic connexin 43 hemichannels through AKT kinase activated by mechanical stimulation. J Biol Chem; 289(15):10582-91.

Becquart P, Cruel M, Hoc T, Sudre L, Pernelle K, Bizios R, Logeart-Avramoglou D, Petite H, Bensidhoum M (2016). Human mesenchymal stem cell responses to hydrostatic pressure and shear stress. Eur Cell Mater; 31:160-73.

Callewaert F, Bakker A, Schrooten J, Van Meerbeek B, Verhoeven G, Boonen S, Vanderschueren D (2010). Androgen receptor disruption increases the osteogenic response to mechanical loading in male mice. J Bone Miner Res; 25(1):124-31.

Castillo AB, Triplett JW, Pavalko FM, Turner CH (2014). Estrogen receptor-β regulates mechanical signaling in primary osteoblasts. Am J Physiol Endocrinol Metab; 306(8):E937-44.

Celil Aydemir AB, Lee S, Won Kim D, Gardner TR, Prince D, Mok Ahn J, Lee FY (2007). Nuclear factor of activated T cell mediates proinflammatory gene expression in response to mechanotransduction. Ann N Y Acad Sci; 1117:138-42.

Celil Aydemir AB, Minematsu H, Gardner TR, Kim KO, Ahn JM, Lee FY (2010). Nuclear factor of activated T cells mediates fluid shear stress- and tensile strain-induced Cox2 in human and murine bone cells. Bone; 46(1):167-75.

Charoenpong H, Osathanon T, Pavasant P, Limjeerajarus N, Keawprachum B, Limjeerajarus CN, Cheewinthamrongrod V, Palaga T, Lertchirakarn V, Ritprajak P (2019). Mechanical stress induced S100A7 expression in human dental pulp cells to augment osteoclast differentiation. Oral Dis; 25(3):812-821.

Chen JC, Chua M, Bellon RB, Jacobs CR (2015). Epigenetic changes during mechanically induced osteogenic lineage commitment. J Biomech Eng; 137(2):020902.

Cheng B, Zhao S, Luo J, Sprague E, Bonewald LF, Jiang JX (2001). Expression of functional gap junctions and regulation by fluid flow in osteocyte-like MLO-Y4 cells. J Bone Miner Res; 16(2):249-59.

Cherian PP, Cheng B, Gu S, Sprague E, Bonewald LF, Jiang JX (2003). Effects of mechanical strain on the function of Gap junctions in osteocytes are mediated through the prostaglandin EP2 receptor. J Biol Chem; 278(44):43146-56.

Cherian PP, Siller-Jackson AJ, Gu S, Wang X, Bonewald LF, Sprague E, Jiang JX (2005). Mechanical strain opens connexin 43 hemichannels in osteocytes: a novel mechanism for the release of prostaglandin. Mol Biol Cell; 16(7):3100-6.

Cui S, Zhao W, Yu S, Xing G, Zhao F (2014). [Research status of mechanical stimulation of stem cells differentiation in stem cells microenvironment]. Zhongguo Xiu Fu Chong Jian Wai Ke Za Zhi; 28(1):100-4.

de Castro LF, Maycas M, Bravo B, Esbrit P, Gortazar A (2015). VEGF Receptor 2 (VEGFR2) Activation Is Essential for Osteocyte Survival Induced by Mechanotransduction. J Cell Physiol; 230(2):278-85.

Deepak V, Kayastha P, McNamara LM (2017). Estrogen deficiency attenuates fluid flow-induced [Ca(2+)](i) oscillations and mechanoresponsiveness of MLO-Y4 osteocytes. FASEB J; 31(7):3027-3039.

Du J, Yang J, He Z, Cui J, Yang Y, Xu M, Qu X, Zhao N, Yan M, Li H, Yu Z (2020). Osteoblast and Osteoclast Activity Affect Bone Remodeling Upon Regulation by Mechanical Loading-Induced Leukemia Inhibitory Factor Expression in Osteocytes. Frontiers in Molecular Biosciences; 7:585056.

Ehnert S, Sreekumar V, Aspera-Werz RH, Sajadian SO, Wintermeyer E, Sandmann GH, Bahrs C, Hengstler JG, Godoy P, Nussler AK (2017). TGF-β(1) impairs mechanosensation of human osteoblasts via HDAC6-mediated shortening and distortion of primary cilia. J Mol Med (Berl); 95(6):653-663.

Fahlgren A, Bratengeier C, Semeins CM, Klein-Nulend J, Bakker AD (2018). Supraphysiological loading induces osteocyte-mediated osteoclastogenesis in a novel in vitro model for bone implant loosening. J Orthop Res; 36(5):1425-1434.

Fu Q, Wu C, Shen Y, Zheng S, Chen R (2008). Effect of LIMK2 RNAi on reorganization of the actin cytoskeleton in osteoblasts induced by fluid shear stress. J Biomech; 41(15):3225-8.

Genetos DC, Kephart CJ, Zhang Y, Yellowley CE, Donahue HJ (2007). Oscillating fluid flow activation of gap junction hemichannels induces ATP release from MLO-Y4 osteocytes. J Cell Physiol; 212(1):207-14.

Geoghegan IP, Hoey DA, McNamara LM (2019). Estrogen deficiency impairs integrin α(v)β(3)-mediated mechanosensation by osteocytes and alters osteoclastogenic paracrine signalling. Sci Rep; 9(1):4654.

Glossop JR, Cartmell SH (2009). Effect of fluid flow-induced shear stress on human mesenchymal stem cells: differential gene expression of IL1B and MAP3K8 in MAPK signaling. Gene Expression Patterns; 9(5):381-8.

González Á, García de Durango C, Alonso V, Bravo B, Rodríguez de Gortázar A, Wells A, Forteza J, Vidal-Vanaclocha F (2017). Distinct Osteomimetic Response of Androgen-Dependent and Independent Human Prostate Cancer Cells to Mechanical Action of Fluid Flow: Prometastatic Implications. Prostate; 77(3):321-333.

Govey PM, Jacobs JM, Tilton SC, Loiselle AE, Zhang Y, Freeman WM, Waters KM, Karin NJ, Donahue HJ (2014). Integrative transcriptomic and proteomic analysis of osteocytic cells exposed to fluid flow reveals novel mechano-sensitive signaling pathways. J Biomech; 47(8):1838-45.

Govey PM, Kawasawa YI, Donahue HJ (2015). Mapping the osteocytic cell response to fluid flow using RNA-Seq. J Biomech; 48(16):4327-32.

Haugh MG, Vaughan TJ, McNamara LM (2015). The role of integrin α(V)β(3) in osteocyte mechanotransduction. J Mech Behav Biomed Mater; 42:67-75.

Hoey DA, Tormey S, Ramcharan S, O'Brien FJ, Jacobs CR (2012). Primary cilia-mediated mechanotransduction in human mesenchymal stem cells. Stem Cells; 30(11):2561-70.

Hu K, Sun H, Gui B, Sui C (2017). TRPV4 functions in flow shear stress induced early osteogenic differentiation of human bone marrow mesenchymal stem cells. Biomed Pharmacother; 91:841-848.

Huang J, Romero-Suarez S, Lara N, Mo C, Kaja S, Brotto L, Dallas SL, Johnson ML, Jähn K, Bonewald LF, Brotto M (2017). Crosstalk between MLO-Y4 osteocytes and C2C12 muscle cells is mediated by the Wnt/β-catenin pathway. JBMR Plus; 1(2):86-100.

Igwe JC, Jiang X, Paic F, Ma L, Adams DJ, Baldock PA, Pilbeam CC, Kalajzic I (2009). Neuropeptide Y is expressed by osteocytes and can inhibit osteoblastic activity. J Cell Biochem; 108(3):621-30.

Jing D, Lu XL, Luo E, Sajda P, Leong PL, Guo XE (2013). Spatiotemporal properties of intracellular calcium signaling in osteocytic and osteoblastic cell networks under fluid flow. Bone; 53(2):531-40.

Joldersma M, Burger EH, Semeins CM, Klein-Nulend J (2000). Mechanical stress induces COX-2 mRNA expression in bone cells from elderly women. J Biomech; 33(1):53-61.

Joldersma M, Klein-Nulend J, Oleksik AM, Heyligers IC, Burger EH (2001). Estrogen enhances mechanical stress-induced prostaglandin production by bone cells from elderly women. Am J Physiol Endocrinol Metab; 280(3):E436-42.

Juffer P, Jaspers RT, Lips P, Bakker AD, Klein-Nulend J (2012). Expression of muscle anabolic and metabolic factors in mechanically loaded MLO-Y4 osteocytes. Am J Physiol Endocrinol Metab; 302(4):E389-95.

Kalogeropoulos M, Varanasi SS, Olstad OK, Sanderson P, Gautvik VT, Reppe S, Francis RM, Gautvik KM, Birch MA, Datta HK (2010). Zic1 transcription factor in bone: neural developmental protein regulates mechanotransduction in osteocytes. FASEB J; 24(8):2893-903.

Kamel MA, Picconi JL, Lara-Castillo N, Johnson ML (2010). Activation of β-catenin signaling in MLO-Y4 osteocytic cells versus 2T3 osteoblastic cells by fluid flow shear stress and PGE2: Implications for the study of mechanosensation in bone. Bone; 47(5):872-81.

Kapur S, Amoui M, Kesavan C, Wang X, Mohan S, Baylink DJ, Lau KH (2010). Leptin receptor (Lepr) is a negative modulator of bone mechanosensitivity and genetic variations in Lepr may contribute to the differential osteogenic response to mechanical stimulation in the C57BL/6J and C3H/HeJ pair of mouse strains. J Biol Chem; 285(48):37607-18.

Kido S, Kuriwaka-Kido R, Imamura T, Ito Y, Inoue D, Matsumoto T (2009). Mechanical stress induces Interleukin-11 expression to stimulate osteoblast differentiation. Bone; 45(6):1125-32.

Kim DH, Heo SJ, Kim SH, Shin JW, Park SH, Shin JW (2011). Shear stress magnitude is critical in regulating the differentiation of mesenchymal stem cells even with endothelial growth medium. Biotechnol Lett; 33(12):2351-9.

Kitase Y, Barragan L, Qing H, Kondoh S, Jiang JX, Johnson ML, Bonewald LF (2010). Mechanical induction of PGE2 in osteocytes blocks glucocorticoid-induced apoptosis through both the β-catenin and PKA pathways. J Bone Miner Res; 25(12):2657-68.

Kitase Y, Lee S, Gluhak-Heinrich J, Johnson ML, Harris SE, Bonewald LF (2014). CCL7 is a protective factor secreted by mechanically loaded osteocytes. J Dent Res; 93(11):1108-15.

Klein-Nulend J, Semeins CM, Burger EH (1996). Prostaglandin mediated modulation of transforming growth factor-beta metabolism in primary mouse osteoblastic cells in vitro. J Cell Physiol; 168(1):1-7.

Klein-Nulend J, Burger EH, Semeins CM, Raisz LG, Pilbeam CC (1997). Pulsating fluid flow stimulates prostaglandin release and inducible prostaglandin G/H synthase mRNA expression in primary mouse bone cells. J Bone Miner Res; 12(1):45-51.

Klein-Nulend J, Helfrich MH, Sterck JG, MacPherson H, Joldersma M, Ralston SH, Semeins CM, Burger EH (1998). Nitric oxide response to shear stress by human bone cell cultures is endothelial nitric oxide synthase dependent. Biochem Biophys Res Commun; 250(1):108-14.

Klein-Nulend J, Sterck JG, Semeins CM, Lips P, Joldersma M, Baart JA, Burger EH (2002). Donor age and mechanosensitivity of human bone cells. Osteoporos Int; 13(2):137-46.

Kraft DC, Bindslev DA, Melsen B, Abdallah BM, Kassem M, Klein-Nulend J (2010). Mechanosensitivity of dental pulp stem cells is related to their osteogenic maturity. Eur J Oral Sci; 118(1):29-38.

Kraft DC, Bindslev DA, Melsen B, Klein-Nulend J (2011). Human dental pulp cells exhibit bone cell-like responsiveness to fluid shear stress. Cytotherapy; 13(2):214-26.

Kulkarni RN, Bakker AD, Everts V, Klein-Nulend J (2010). Inhibition of osteoclastogenesis by mechanically loaded osteocytes: involvement of MEPE. Calcif Tissue Int; 87(5):461-8.

Kulkarni RN, Bakker AD, Everts V, Klein-Nulend J (2012a). Mechanical loading prevents the stimulating effect of IL-1β on osteocyte-modulated osteoclastogenesis. Biochem Biophys Res Commun; 420(1):11-6.

Kulkarni RN, Bakker AD, Gruber EV, Chae TD, Veldkamp JB, Klein-Nulend J, Everts V (2012b). MT1-MMP modulates the mechanosensitivity of osteocytes. Biochem Biophys Res Commun; 417(2):824-9.

Kuo YC, Chang TH, Hsu WT, Zhou J, Lee HH, Hui-Chun Ho J, Chien S, Lee OK (2015). Oscillatory shear stress mediates directional reorganization of actin cytoskeleton and alters differentiation propensity of mesenchymal stem cells. Stem Cells; 33(2):429-42.

Lau KH, Kapur S, Kesavan C, Baylink DJ (2006). Up-regulation of the Wnt, estrogen receptor, insulin-like growth factor-I, and bone morphogenetic protein pathways in C57BL/6J osteoblasts as opposed to C3H/HeJ osteoblasts in part contributes to the differential anabolic response to fluid shear. J Biol Chem; 281(14):9576-88.

Lee HJ, Diaz MF, Ewere A, Olson SD, Cox CS, Jr., Wenzel PL (2017). Focal adhesion kinase signaling regulates anti-inflammatory function of bone marrow mesenchymal stromal cells induced by biomechanical force. Cell Signal; 38:1-9.

Li J, Liu D, Ke HZ, Duncan RL, Turner CH (2005). The P2X7 nucleotide receptor mediates skeletal mechanotransduction. J Biol Chem; 280(52):42952-9.

Li J, Rose E, Frances D, Sun Y, You L (2012). Effect of oscillating fluid flow stimulation on osteocyte mRNA expression. J Biomech; 45(2):247-51.

Li X, Liu C, Li P, Li S, Zhao Z, Chen Y, Huo B, Zhang D (2013). Connexin 43 is a potential regulator in fluid shear stress-induced signal transduction in osteocytes. J Orthop Res; 31(12):1959-65.
Li X, Han L, Nookaew I, Mannen E, Silva MJ, Almeida M, Xiong J (2019). Stimulation of Piezo1 by mechanical signals promotes bone anabolism. Elife; 8.
Li YJ, Batra NN, You L, Meier SC, Coe IA, Yellowley CE, Jacobs CR (2004). Oscillatory fluid flow affects human marrow stromal cell proliferation and differentiation. J Orthop Res; 22(6):1283-9.
Liao C, Cheng T, Wang S, Zhang C, Jin L, Yang Y (2017). Shear stress inhibits IL-17A-mediated induction of osteoclastogenesis via osteocyte pathways. Bone; 101:10-20.
Lim KT, Kim J, Seonwoo H, Chang JU, Choi H, Hexiu J, Cho WJ, Choung PH, Chung JH (2013). Enhanced osteogenesis of human alveolar bone-derived mesenchymal stem cells for tooth tissue engineering using fluid shear stress in a rocking culture method. Tissue Eng Part C Methods; 19(2):128-45.
Lim KT, Hexiu J, Kim J, Seonwoo H, Choung PH, Chung JH (2014). Synergistic effects of orbital shear stress on in vitro growth and osteogenic differentiation of human alveolar bone-derived mesenchymal stem cells. Biomed Res Int; 2014:316803.
Litzenberger JB, Kim JB, Tummala P, Jacobs CR (2010). Beta1 integrins mediate mechanosensitive signaling pathways in osteocytes. Calcif Tissue Int; 86(4):325-32.
Liu C, Zhang X, Wu M, You L (2015). Mechanical loading up-regulates early remodeling signals from osteocytes subjected to physical damage. J Biomech; 48(16):4221-8.
Lu XL, Huo B, Chiang V, Guo XE (2012a). Osteocytic network is more responsive in calcium signaling than osteoblastic network under fluid flow. J Bone Miner Res; 27(3):563-74.
Lu XL, Huo B, Park M, Guo XE (2012b). Calcium response in osteocytic networks under steady and oscillatory fluid flow. Bone; 51(3):466-73.
Maeda A, Soejima K, Bandow K, Kuroe K, Kakimoto K, Miyawaki S, Okamoto A, Matsuguchi T (2007). Force-induced IL-8 from periodontal ligament cells requires IL-1beta. J Dent Res; 86(7):629-34.
Maycas M, Ardura JA, de Castro LF, Bravo B, Gortázar AR, Esbrit P (2015). Role of the Parathyroid Hormone Type 1 Receptor (PTH1R) as a Mechanosensor in Osteocyte Survival. J Bone Miner Res; 30(7):1231-44.
Maycas M, Portolés MT, Matesanz MC, Buendía I, Linares J, Feito MJ, Arcos D, Vallet-Regí M, Plotkin LI, Esbrit P, Gortázar AR (2017). High glucose alters the secretome of mechanically stimulated osteocyte-like cells affecting osteoclast precursor recruitment and differentiation. J Cell Physiol; 232(12):3611-3621.
McGarry JG, Klein-Nulend J, Mullender MG, Prendergast PJ (2005). A comparison of strain and fluid shear stress in stimulating bone cell responses--a computational and experimental study. FASEB J; 19(3):482-4.
Mehrotra M, Saegusa M, Wadhwa S, Voznesensky O, Peterson D, Pilbeam C (2006). Fluid flow induces Rankl expression in primary murine calvarial osteoblasts. J Cell Biochem; 98(5):1271-83.
Middleton K, Kondiboyina A, Borrett M, Cui Y, Mei X, You L (2018). Microfluidics approach to investigate the role of dynamic similitude in osteocyte mechanobiology. J Orthop Res; 36(2):663-671.
Pathak JL, Bravenboer N, Luyten FP, Verschueren P, Lems WF, Klein-Nulend J, Bakker AD (2015). Mechanical loading reduces inflammation-induced human osteocyte-to-osteoclast communication. Calcif Tissue Int; 97(2):169-78.
Qi L, Zhang Y (2014). The microRNA 132 regulates fluid shear stress-induced differentiation in periodontal ligament cells through mTOR signaling pathway. Cell Physiol Biochem; 33(2):433-45.
Rangaswami H, Marathe N, Zhuang S, Chen Y, Yeh JC, Frangos JA, Boss GR, Pilz RB (2009). Type II cGMP-dependent protein kinase mediates osteoblast mechanotransduction. J Biol Chem; 284(22):14796-808.
Rangaswami H, Schwappacher R, Tran T, Chan GC, Zhuang S, Boss GR, Pilz RB (2012). Protein kinase G and focal adhesion kinase converge on Src/Akt/β-catenin signaling module in osteoblast mechanotransduction. J Biol Chem; 287(25):21509-19.
Rath AL, Bonewald LF, Ling J, Jiang JX, Van Dyke ME, Nicolella DP (2010). Correlation of cell strain in single osteocytes with intracellular calcium, but not intracellular nitric oxide, in response to fluid flow. J Biomech; 43(8):1560-4.
Reilly GC, Haut TR, Yellowley CE, Donahue HJ, Jacobs CR (2003). Fluid flow induced PGE2 release by bone cells is reduced by glycocalyx degradation whereas calcium signals are not. Biorheology; 40(6):591-603.
Ren J, Wang XH, Wang GC, Wu JH (2013). 17β estradiol regulation of connexin 43-based gap junction and mechanosensitivity through classical estrogen receptor pathway in osteocyte-like MLO-Y4 cells. Bone; 53(2):587-96.
Riddle RC, Taylor AF, Genetos DC, Donahue HJ (2006). MAP kinase and calcium signaling mediate fluid flow-induced human mesenchymal stem cell proliferation. Am J Physiol Cell Physiol; 290(3):C776-84.
Riddle RC, Taylor AF, Rogers JR, Donahue HJ (2007). ATP release mediates fluid flow-induced proliferation of human bone marrow stromal cells. J Bone Miner Res; 22(4):589-600.
Riquelme MA, Gu S, Hua R, Jiang JX (2021). Mechanotransduction via the coordinated actions of integrins, PI3K signaling and Connexin hemichannels. Bone Res; 9(1):8.
Salvi JD, Lim JY, Donahue HJ (2010). Increased mechanosensitivity of cells cultured on nanotopographies. J Biomech; 43(15):3058-62.
Santos A, Bakker AD, Zandieh-Doulabi B, Semeins CM, Klein-Nulend J (2009). Pulsating fluid flow modulates gene expression of proteins involved in Wnt signaling pathways in osteocytes. J Orthop Res; 27(10):1280-7.
Santos A, Bakker AD, Zandieh-Doulabi B, de Blieck-Hogervorst JM, Klein-Nulend J (2010). Early activation of the beta-catenin pathway in osteocytes is mediated by nitric oxide, phosphatidyl inositol-3 kinase/Akt, and focal adhesion kinase. Biochem Biophys Res Commun; 391(1):364-9.
Santos A, Bakker AD, Willems HM, Bravenboer N, Bronckers AL, Klein-Nulend J (2011). Mechanical loading stimulates BMP7, but not BMP2, production by osteocytes. Calcif Tissue Int; 89(4):318-26.
Seref-Ferlengez Z, Maung S, Schaffler MB, Spray DC, Suadicani SO, Thi MM (2016). P2X7R-Panx1 Complex Impairs Bone Mechanosignaling under High Glucose Levels Associated with Type-1 Diabetes. PLoS One; 11(5):e0155107.
Shah KM, Orton P, Mani N, Wilkinson JM, Gartland A (2017). Osteocyte physiology and response to fluid shear stress are impaired following exposure to cobalt and chromium: Implications for bone health following joint replacement. J Orthop Res; 35(8):1716-1723.
Soejima K, Klein-Nulend J, Semeins CM, Burger EH (2001). Different responsiveness of cells from adult and neonatal mouse bone to mechanical and biochemical challenge. J Cell Physiol; 186(3):366-70.
Sonam S, Sathe SR, Yim EK, Sheetz MP, Lim CT (2016). Cell contractility arising from topography and shear flow determines human mesenchymal stem cell fate. Scientific Reports; 6:20415.

Sterck JG, Klein-Nulend J, Lips P, Burger EH (1998). Response of normal and osteoporotic human bone cells to mechanical stress in vitro. Am J Physiol; 274(6):E1113-20.
Suzuki T, Notomi T, Miyajima D, Mizoguchi F, Hayata T, Nakamoto T, Hanyu R, Kamolratanakul P, Mizuno A, Suzuki M, Ezura Y, Izumi Y, Noda M (2013). Osteoblastic differentiation enhances expression of TRPV4 that is required for calcium oscillation induced by mechanical force. Bone; 54(1):172-8.
Tang M, Peng Z, Mai Z, Chen L, Mao Q, Chen Z, Chen Q, Liu L, Wang Y, Ai H (2014). Fluid shear stress stimulates osteogenic differentiation of human periodontal ligament cells via the extracellular signal-regulated kinase 1/2 and p38 mitogen-activated protein kinase signaling pathways. J Periodontol; 85(12):1806-13.
Thi MM, Kojima T, Cowin SC, Weinbaum S, Spray DC (2003). Fluid shear stress remodels expression and function of junctional proteins in cultured bone cells. Am J Physiol Cell Physiol; 284(2):C389-403.
Thi MM, Suadicani SO, Spray DC (2010). Fluid flow-induced soluble vascular endothelial growth factor isoforms regulate actin adaptation in osteoblasts. J Biol Chem; 285(40):30931-41.
Thi MM, Islam S, Suadicani SO, Spray DC (2012). Connexin43 and pannexin1 channels in osteoblasts: who is the "hemichannel"? J Membr Biol; 245(7):401-9.
van der Meijden K, Bakker AD, van Essen HW, Heijboer AC, Schulten EA, Lips P, Bravenboer N (2016). Mechanical loading and the synthesis of 1,25(OH)2D in primary human osteoblasts. J Steroid Biochem Mol Biol; 156:32-9.
van der Pauw MT, Klein-Nulend J, van den Bos T, Burger EH, Everts V, Beertsen W (2000). Response of periodontal ligament fibroblasts and gingival fibroblasts to pulsating fluid flow: nitric oxide and prostaglandin E_2 release and expression of tissue non-specific alkaline phosphatase activity. J Periodontal Res; 35(6):335-43.
Wang S, Li S, Hu M, Huo B (2019). Calcium response in bone cells at different osteogenic stages under unidirectional or oscillatory flow. Biomicrofluidics; 13(6):064117.
Wolf M, Lossdörfer S, Craveiro R, Götz W, Jäger A (2013). Regulation of macrophage migration and activity by high-mobility group box 1 protein released from periodontal ligament cells during orthodontically induced periodontal repair: an in vitro and in vivo experimental study. J Orofac Orthop; 74(5):420-34.
Xia X, Batra N, Shi Q, Bonewald LF, Sprague E, Jiang JX (2010). Prostaglandin promotion of osteocyte gap junction function through transcriptional regulation of connexin 43 by glycogen synthase kinase 3/beta-catenin signaling. Mol Cell Biol; 30(1):206-19.
Xing Y, Gu Y, Bresnahan JJ, Paul EM, Donahue HJ, You J (2014). The roles of P2Y2 purinergic receptors in osteoblasts and mechanotransduction. PLoS One; 9(9):e108417.
Xu H, Zhang J, Wu J, Guan Y, Weng Y, Shang P (2012). Oscillatory fluid flow elicits changes in morphology, cytoskeleton and integrin-associated molecules in MLO-Y4 cells, but not in MC3T3-E1 cells. Biol Res; 45(2):163-9.
Xu H, Guan Y, Wu J, Zhang J, Duan J, An L, Shang P (2014). Polycystin 2 is involved in the nitric oxide production in responding to oscillating fluid shear in MLO-Y4 cells. J Biomech; 47(2):387-91.
Yan Z, Wang P, Wu J, Feng X, Cai J, Zhai M, Li J, Liu X, Jiang M, Luo E, Jing D (2018). Fluid shear stress improves morphology, cytoskeleton architecture, viability, and regulates cytokine expression in a time-dependent manner in MLO-Y4 cells. Cell Biol Int; 42(10):1410-1422.
Yang Z, Bidwell JP, Young SR, Gerard-O'Riley R, Wang H, Pavalko FM (2010). Nmp4/CIZ inhibits mechanically induced beta-catenin signaling activity in osteoblasts. J Cell Physiol; 223(2):435-41.
Yang Z, Tan S, Shen Y, Chen R, Wu C, Xu Y, Song Z, Fu Q (2015). Inhibition of FSS-induced actin cytoskeleton reorganization by silencing LIMK2 gene increases the mechanosensitivity of primary osteoblasts. Bone; 74:182-90.
Yourek G, McCormick SM, Mao JJ, Reilly GC (2010). Shear stress induces osteogenic differentiation of human mesenchymal stem cells. Regen Med; 5(5):713-24.
Yuan L, Sakamoto N, Song G, Sato M (2012). Migration of human mesenchymal stem cells under low shear stress mediated by mitogen-activated protein kinase signaling. Stem Cells Dev; 21(13):2520-30.
Zhang JN, Zhao Y, Liu C, Han ES, Yu X, Lidington D, Bolz SS, You L (2015). The role of the sphingosine-1-phosphate signaling pathway in osteocyte mechanotransduction. Bone; 79:71-8.
Zhang K, Barragan-Adjemian C, Ye L, Kotha S, Dallas M, Lu Y, Zhao S, Harris M, Harris SE, Feng JQ, Bonewald LF (2006). E11/gp38 selective expression in osteocytes: regulation by mechanical strain and role in dendrite elongation. Mol Cell Biol; 26(12):4539-52.
Zheng L, Huang Y, Song W, Gong X, Liu M, Jia X, Zhou G, Chen L, Li A, Fan Y (2012). Fluid shear stress regulates metalloproteinase-1 and 2 in human periodontal ligament cells: involvement of extracellular signal-regulated kinase (ERK) and P38 signaling pathways. J Biomech; 45(14):2368-75.
Zheng L, Chen L, Chen Y, Gui J, Li Q, Huang Y, Liu M, Jia X, Song W, Ji J, Gong X, Shi R, Fan Y (2016). The effects of fluid shear stress on proliferation and osteogenesis of human periodontal ligament cells. J Biomech; 49(4):572-9.
Zheng L, Shi Q, Na J, Liu N, Guo Y, Fan Y (2019). Platelet-Derived Growth Factor Receptor-α and β are Involved in Fluid Shear Stress Regulated Cell Migration in Human Periodontal Ligament Cells. Cell Mol Bioeng; 12(1):85-97.
