# Supplementary material for: Fluid flow shear stress and tissue remodeling—an orthodontic perspective: evidence synthesis and differential gene expression network analysis
Source: Front Bioeng Biotechnol. 2023 Sep 18;11:1256825. doi: 10.3389/fbioe.2023.1256825 (PMC10545883; doi:10.3389/fbioe.2023.1256825)
Supplement: Supplementary file 4 [file DataSheet6.pdf]

Supplement 6

"Fluid Flow Shear Stress and Tissue Remodeling – an Orthodontic Perspective: Evidence Synthesis and Differential Gene Expression Network analysis"

Analysis of the commonly investigated gene or metabolite by at least three studies

The articles were organised in this table according to the regulation description. **Increase expression** label includes "increase" + "increase with plateau" + "temporary increase" regulation description reports, while **decrease expression** includes "decrease" + "decrease with plateau" + "Temporary decrease" regulation description reports. Each repeated Article in each row was highlighted in red and counted once. The total percentage was given by dividing the highest number of citations under one of the regulation descriptions by the total number of citations of the given gene/small molecule.

Contents

6.1 Human mesenchymal stem cells 2

6.2 Human osteoblasts 4

6.3 Human PDL cells 5

6.4 Mouse osteoblasts 6

6.5 Mouse osteocytes 7

6.1 Human mesenchymal stem cells

Gene or metabolite	Fluid flow profile	Increase expression		Decrease expression		No change in expression		Other changes in expressions	Number of studies	Total number of studies	Total percentage of studies reporting upregulation
		Gene	Protein or small molecule	Gene	Protein or small molecule	Gene	Protein or small molecule				
ALPL or ALP	Oscillatory laminar	Lim et al. (2013)	Lim et al. (2013), Lim et al. (2014)		Li et al. (2004)				3	6	83.3% (5/6)
	Steady laminar	Sonam et al. (2016), Becquart et al. (2016)	Yourek et al. (2010), Sonam et al. (2016)		Yourek et al. (2010)				3		
	Pulsatile laminar								0		
BMP2	Oscillatory laminar		Lim et al. (2014)					Hoey et al. (2012)	2	3	66.6% (2/3)
	Steady laminar	Yourek et al. (2010)							1		
	Pulsatile laminar								0		
PTGS2	Oscillatory laminar	Celil Aydemir et al. (2010), Celil Aydemir et al. (2007)	Celil Aydemir et al. (2010)					Hoey et al. (2012)	3	7	85.7% (6/7)
	Steady laminar	Becquart et al. (2016), Lee et al. (2017)						Lee et al. (2017)	2		
	Pulsatile laminar	Kraft et al. (2010), Kraft et al. (2011)							2		
BGLAP	Oscillatory laminar	Lim et al. (2013), Li et al. (2004)						Lim et al. (2014)	3	5	60% (3/5)
	Steady laminar	Sonam et al. (2016)	Sonam et al. (2016)					Hu et al. (2017)	2		
	Pulsatile laminar								0		
Calcium	Oscillatory laminar		Li et al. (2004), Riddle et al. (2007), Salvi et al. (2010), Riddle et al. (2006)						4	6	100% (6/6)
	Steady laminar		Lee et al. (2017), Hu et al. (2017)						2		
	Pulsatile laminar								0		
RUNX2	Oscillatory laminar	Kuo et al. (2015), Lim et al. (2013)		Li et al. (2004)				Lim et al. (2013), Hoey et al. (2012), Lim et al. (2014)	5	7	57.1% (4/7)
	Steady laminar	Becquart et al. (2016), Sonam et al. (2016)	Sonam et al. (2016)						2		
	Pulsatile laminar								0		
COL1A1	Oscillatory laminar	Li et al. (2004), Lim et al. (2013)						Lim et al. (2014)	3	3	66.6% (2/3)
	Steady laminar								0		
	Pulsatile laminar								0		

Gene or metabolite	Fluid flow profile	Increase expression		Decrease expression		No change in expression		Other changes in expressions	Number of studies	Total number of studies	Total percentage of studies reporting upregulation
		Gene	Protein or small molecule	Gene	Protein or small molecule	Gene	Protein or small molecule				
Nitric oxide	Oscillatory laminar								0	3	100% (3/3)
	Steady laminar		Becquart et al. (2016)						1		
	Pulsatile laminar		Kraft et al. (2011), Kraft et al. (2010)					Kraft et al. (2011), Kraft et al. (2010)	2		
SPP1	Oscillatory laminar	Li et al. (2004), Lim et al. (2013)						Hoey et al. (2012), Lim et al. (2014)	4	6	66.6% (4/6)
	Steady laminar	Yourek et al. (2010), Sonam et al. (2016)	Sonam et al. (2016)						2		
	Pulsatile laminar								0		
VEGFA	Oscillatory laminar		Lim et al. (2013), Lim et al. (2014)						2	4	100% (4/4)
	Steady laminar	Becquart et al. (2016), Charoenpong et al. (2019)							2		
	Pulsatile laminar								0		
MAPK3; MAPK1	Oscillatory laminar		Riddle et al. (2006)						1	4	100% (4/4)
	Steady laminar		Yuan et al. (2012), Becquart et al. (2016), Lee et al. (2017)		Yuan et al. (2012)				3		
	Pulsatile laminar								0		

6.2 Human osteoblasts

Gene or metabolite	Fluid flow profile	Increase expression		Decrease expression		No change in expression		Other changes in expressions	Number of studies	Total number of studies	Total percentage of studies reporting upregulation
		Gene	Protein or small molecule	Gene	Protein or small molecule	Gene	Protein or small molecule				
Nitric oxide	Oscillatory Laminar								0	5	100% (5/5)
	Steady Laminar								0		
	Pulsatile Laminar		McGarry et al. (2005), Sterck et al. (1998), van der Meijden et al. (2016), Santos et al. (2011), Klein-Nulend et al. (1998)						5		
PGE2	Oscillatory Laminar								0	6	100% (6/6)
	Steady Laminar								0		
	Pulsatile Laminar		Joldersma et al. (2001), Klein-Nulend et al. (2002), McGarry et al. (2005), Sterck et al. (1998), Bakker et al. (2003b) , Joldersma et al. (2000)						6		
PGI2	Oscillatory Laminar								0	3	100% (3/3)
	Steady Laminar								0		
	Pulsatile Laminar		Joldersma et al. (2001), Joldersma et al. (2000), Klein-Nulend et al. (2002)						3		
PTGS1	Oscillatory Laminar								0	3	100% (3/3)
	Steady Laminar								0		
	Pulsatile Laminar	Joldersma et al. (2001), Bakker et al. (2003b), Joldersma et al. (2000)							3		
PTGS2	Oscillatory Laminar								0	3	100% (3/3)
	Steady Laminar								0		
	Pulsatile Laminar	Joldersma et al. (2001), Bakker et al. (2003b), Joldersma et al. (2000)							3		

6.3 Human PDL cells

Gene or metabolite	Fluid flow profile	Increase expression		Decrease expression		No change in expression		Other changes in expressions	Number of studies	Total number of studies	Total percentage of studies reporting upregulation
		Gene	Protein or small molecule	Gene	Protein or small molecule	Gene	Protein or small molecule				
ALPP or ALP	Oscillatory Laminar								0	3	100% (3/3)
	Steady Laminar	Zheng et al. (2016), Qi and Zhang (2014)	Tang et al. (2014), Qi and Zhang (2014)					Tang et al. (2014)	3		
	Pulsatile Laminar								0		

6.4 Mouse osteoblasts

Gene or metabolite	Fluid flow profile	Increase expression		Decrease expression		No change in expression		Other changes in expressions	Number of studies	Total number of studies	Total percentage of studies reporting upregulation
		Gene	Protein or small molecule	Gene	Protein or small molecule	Gene	Protein or small molecule				
Mapk3; Mapk1	Oscillatory Laminar		Xing et al. (2014), Castillo et al. (2014)					Yang et al. (2010)	3	6	83% (5/6)
	Steady Laminar		Mehrotra et al. (2006), Kapur et al. (2010), Lau et al. (2006)						3		
	Pulsatile Laminar								0		
Nitrite oxide	Oscillatory Laminar								0	5	100% (5/5)
	Steady Laminar								0		
	Pulsatile Laminar		Bakker et al. (2003a), Bakker et al. (2001), Soejima et al. (2001), Bakker et al. (2013a); Callewaert et al. (2010)						5		
PGE2	Oscillatory Laminar		Castillo et al. (2014)						1	8	100% (8/8)
	Steady Laminar		Li et al. (2005)						1		
	Pulsatile Laminar		Bakker et al. (2003a), Thi et al. (2012), Bakker et al. (2001), Klein-Nulend et al. (1997), Klein-Nulend et al. (1996), Bakker et al. (2003b)						6		
Ptgs2	Oscillatory Laminar		Castillo et al. (2014)						1	6	100% (6/6)
	Steady Laminar	Mehrotra et al. (2006)	Lau et al. (2006), Yang et al. (2015)						3		
	Pulsatile Laminar	Klein-Nulend et al. (1997), Callewaert et al. (2010)	Klein-Nulend et al. (1997)						2		

6.5 Mouse osteocytes

Gene or metabolite	Fluid flow profile	Increase expression		Decrease expression		No change in expression		Other changes in expressions	Number of studies	Total number of studies	Total percentage of studies reporting upregulation
		Gene	Protein or small molecule	Gene	Protein or small molecule	Gene	Protein or small molecule				
Calcium	Oscillatory Laminar		Deepak et al. (2017), Seref-Ferlengez et al. (2016), Litzenberger et al. (2010), Lu et al. (2012b), Zhang et al. (2015), Reilly et al. (2003)					Wang et al. (2019)	7	14	92.8% (13/14)
	Steady Laminar		Lu et al. (2012b), Lu et al. (2012a), Jing et al. (2013), Middleton et al. (2018)						4		
	Pulsatile Laminar		Rath et al. (2010), Bakker et al. (2009)						2		
	n.g.		Shah et al. (2017)						1		
Runx2	Oscillatory Laminar	Xu et al. (2012)		Chen et al. (2015)					2	3	66.6% (2/3)
	Steady Laminar								0		
	Pulsatile Laminar	González et al. (2017)							1		
Sost	Oscillatory Laminar			Deepak et al. (2017)					1	4	25% (1/4)
	Steady Laminar		Li et al. (2013)	Li et al. (2013)	Li et al. (2013), Riquelme et al. (2021)			Li et al. (2013), Yan et al. (2018)	3		
	Pulsatile Laminar								0		
Tnfsf11	Oscillatory Laminar			Geoghegan et al. (2019), Haugh et al. (2015)					2	11	63.6% (7/11)
	Steady Laminar	Li et al. (2013)	Li et al. (2013)		Yan et al. (2018)			Li et al. (2013), Yan et al. (2018)	2		
	Pulsatile Laminar	Kulkarni et al. (2012a), Bakker et al. (2013b); Kulkarni et al. (2010), Liao et al. (2017)	Fahlgren et al. (2018)	Fahlgren et al. (2018)	Maycas et al. (2017), Fahlgren et al. (2018), Liao et al. (2017)				6		
	n.g.	Shah et al. (2017)							1		
Akt1	Oscillatory Laminar								0	3	100% (3/3)
	Steady Laminar		Xia et al. (2010), Riquelme et al. (2021), Batra et al. (2014)						3		
	Pulsatile Laminar								0		

Gene or metabolite	Fluid flow profile	Increase expression		Decrease expression		No change in expression		Other changes in expressions	Number of studies	Total number of studies	Total percentage of studies reporting upregulation
		Gene	Protein or small molecule	Gene	Protein or small molecule	Gene	Protein or small molecule				
Bglap	Oscillatory Laminar	Chen et al. (2015), Deepak et al. (2017)							2	3	100% (3/3)
	Steady Laminar	Yan et al. (2018)	Yan et al. (2018)						1		
	Pulsatile Laminar								0		
Ctnnb1	Oscillatory Laminar								0	7	85.7% (6/7)
	Steady Laminar	Yan et al. (2018)	Xia et al. (2010) Yan et al. (2018)						2		
	Pulsatile Laminar		Santos et al. (2010), Maycas et al. (2017), Maycas et al. (2015), de Castro et al. (2015)					Santos et al. (2009)	5		
RANKL/OPG	Oscillatory Laminar			Geoghegan et al. (2019), Zhang et al. (2015), Litzenberger et al. (2010), Li et al. (2012)					4	8	12.5% (1/8)
	Steady Laminar			Yan et al. (2018)	Yan et al. (2018)				1		
	Pulsatile Laminar		Fahlgren et al. (2018)	Kulkarni et al. (2010), Liao et al. (2017)	Fahlgren et al. (2018)				3		
Spp1	Oscillatory Laminar	Xu et al. (2012), Chen et al. (2015), Deepak et al. (2017)							3	3	100% (3/3)
	Steady Laminar								0		
	Pulsatile Laminar								0		
Vegfa	Oscillatory Laminar	Liu et al. (2015)	Liu et al. (2015)						1	6	83% (5/6)
	Steady Laminar								0		
	Pulsatile Laminar	González et al. (2017), de Castro et al. (2015), Juffer et al. (2012)	Thi et al. (2010), Juffer et al. (2012)		Maycas et al. (2017)				5		
Gja1	Oscillatory Laminar		Alford et al. (2003), Ren et al. (2013)						2	10	90% (9/10)
	Steady Laminar	Thi et al. (2003), Li et al. (2013), Yan et al. (2018)	Xia et al. (2010), Li et al. (2013), Cherian et al. (2005), Yan et al. (2018)		Li et al. (2013)			Li et al. (2013), Cheng et al. (2001)	6		
	Pulsatile Laminar	Santos et al. (2009)							1		
	n.g.	Shah et al. (2017)							1		
Mapk3; Mapk1	Oscillatory Laminar		Ren et al. (2013)						1	5	100% (5/5)
	Steady Laminar								0		

Gene or metabolite	Fluid flow profile	Increase expression		Decrease expression		No change in expression		Other changes in expressions	Number of studies	Total number of studies	Total percentage of studies reporting upregulation
		Gene	Protein or small molecule	Gene	Protein or small molecule	Gene	Protein or small molecule				
	Pulsatile Laminar		Maycas et al. (2017), Maycas et al. (2015), Liao et al. (2017), de Castro et al. (2015)						4		
PGE2	Oscillatory Laminar		Liu et al. (2015), Zhang et al. (2015), Reilly et al. (2003), Genetos et al. (2007), Litzenberger et al. (2010), Haugh et al. (2015), Xu et al. (2014), Deepak et al. (2017)						8	12	100% (12/12)
	Steady Laminar		Cherian et al. (2005)						1		
	Pulsatile Laminar		Kamel et al. (2010), Fahlgren et al. (2018), Bakker et al. (2013b)		Fahlgren et al. (2018)				3		
Tnfrsf11b	Oscillatory Laminar	Geoghegan et al. (2019), Li et al. (2019)		Haugh et al. (2015)					3	11	81.8% (9/11)
	Steady Laminar	Li et al. (2013),		Li et al. (2013)				Yan et al. (2018)	2		
	Pulsatile Laminar	Kulkarni et al. (2012a), Fahlgren et al. (2018), González et al. (2017), Bakker et al. (2013b), Kulkarni et al. (2010), Liao et al. (2017)	Fahlgren et al. (2018), Liao et al. (2017)	Fahlgren et al. (2018)	Fahlgren et al. (2018)				6		
Nitric oxide	Oscillatory Laminar		Xu et al. (2014), Deepak et al. (2017)						2	11	100% (11/11)
	Steady Laminar								0		
	Pulsatile Laminar		Santos et al. (2010), Bakker et al. (2014), Kulkarni et al. (2012b), Rath et al. (2010), Bakker et al. (2009), Juffer et al. (2012), Santos et al. (2009), Fahlgren et al. (2018), Bakker et al. (2013b)						9		

Gene or metabolite	Fluid flow profile	Increase expression		Decrease expression		No change in expression		Other changes in expressions	Number of studies	Total number of studies	Total percentage of studies reporting upregulation
		Gene	Protein or small molecule	Gene	Protein or small molecule	Gene	Protein or small molecule				
Pdpn	Oscillatory Laminar	Xu et al. (2012)							1	3	100% (3/3)
	Steady Laminar	Zhang et al. (2006)							1		
	Pulsatile Laminar								0		
	n.g.	Shah et al. (2017)							1		
Ptgs2	Oscillatory Laminar	Geoghegan et al. (2019), Zhang et al. (2015), Xu et al. (2012), Litzenberger et al. (2010), Li et al. (2012), Haugh et al. (2015), Xu et al. (2014), Li et al. (2019), Liu et al. (2015)							9	13	100% (13/13)
	Steady Laminar								0		
	Pulsatile Laminar	Bakker et al. (2013b), Fahlgren et al. (2018), Kamel et al. (2010), Bakker et al. (2014)		Fahlgren et al. (2018)		Fahlgren et al. (2018)			4		
Wnt3a	Oscillatory Laminar								0	3	66.7% (2/3)
	Steady Laminar	Yan et al. (2018)	Yan et al. (2018)						1		
	Pulsatile Laminar							Santos et al. (2009)	1		
	n.g.	Huang et al. (2017)		Huang et al. (2017)					1		

References

Alford AI, Jacobs CR, Donahue HJ (2003). Oscillating fluid flow regulates gap junction communication in osteocytic MLO-Y4 cells by an ERK1/2 MAP kinase-dependent mechanism. Bone; 33(1):64-70.

Bakker AD, Soejima K, Klein-Nulend J, Burger EH (2001). The production of nitric oxide and prostaglandin E(2) by primary bone cells is shear stress dependent. J Biomech; 34(5):671-7.

Bakker AD, Joldersma M, Klein-Nulend J, Burger EH (2003a). Interactive effects of PTH and mechanical stress on nitric oxide and PGE2 production by primary mouse osteoblastic cells. Am J Physiol Endocrinol Metab; 285(3):E608-13.

Bakker AD, Klein-Nulend J, Burger EH (2003b). Mechanotransduction in bone cells proceeds via activation of COX-2, but not COX-1. Biochem Biophys Res Commun; 305(3):677-83.

Bakker AD, Silva VC, Krishnan R, Bacabac RG, Blaauboer ME, Lin YC, Marcantonio RA, Cirelli JA, Klein-Nulend J (2009). Tumor necrosis factor alpha and interleukin-1beta modulate calcium and nitric oxide signaling in mechanically stimulated osteocytes. Arthritis Rheum; 60(11):3336-45.

Bakker AD, Huesa C, Hughes A, Aspden RM, van't Hof RJ, Klein-Nulend J, Helfrich MH (2013a). Endothelial nitric oxide synthase is not essential for nitric oxide production by osteoblasts subjected to fluid shear stress in vitro. Calcif Tissue Int; 92(3):228-39.

Bakker AD, Zandieh-Doulabi B, Klein-Nulend J (2013b). Strontium ranelate affects signaling from mechanically-stimulated osteocytes towards osteoclasts and osteoblasts. Bone; 53(1):112-9.

Bakker AD, Kulkarni RN, Klein-Nulend J, Lems WF (2014). IL-6 alters osteocyte signaling toward osteoblasts but not osteoclasts. J Dent Res; 93(4):394-9.

Batra N, Riquelme MA, Burra S, Kar R, Gu S, Jiang JX (2014). Direct regulation of osteocytic connexin 43 hemichannels through AKT kinase activated by mechanical stimulation. J Biol Chem; 289(15):10582-91.

Becquart P, Cruel M, Hoc T, Sudre L, Pernelle K, Bizios R, Logeart-Avramoglou D, Petite H, Bensidhoum M (2016). Human mesenchymal stem cell responses to hydrostatic pressure and shear stress. Eur Cell Mater; 31:160-73.

Callewaert F, Bakker A, Schrooten J, Van Meerbeek B, Verhoeven G, Boonen S, Vanderschueren D (2010). Androgen receptor disruption increases the osteogenic response to mechanical loading in male mice. J Bone Miner Res; 25(1):124-31.

Castillo AB, Triplett JW, Pavalko FM, Turner CH (2014). Estrogen receptor-β regulates mechanical signaling in primary osteoblasts. Am J Physiol Endocrinol Metab; 306(8):E937-44.

Celil Aydemir AB, Lee S, Won Kim D, Gardner TR, Prince D, Mok Ahn J, Lee FY (2007). Nuclear factor of activated T cell mediates proinflammatory gene expression in response to mechanotransduction. Ann N Y Acad Sci; 1117:138-42.

Celil Aydemir AB, Minematsu H, Gardner TR, Kim KO, Ahn JM, Lee FY (2010). Nuclear factor of activated T cells mediates fluid shear stress- and tensile strain-induced Cox2 in human and murine bone cells. Bone; 46(1):167-75.

Charoenpong H, Osathanon T, Pavasant P, Limjeerajarus N, Keawprachum B, Limjeerajarus CN, Cheewinthamrongrod V, Palaga T, Lertchirakarn V, Ritprajak P (2019). Mechanical stress induced S100A7 expression in human dental pulp cells to augment osteoclast differentiation. Oral Dis; 25(3):812-821.

Chen JC, Chua M, Bellon RB, Jacobs CR (2015). Epigenetic changes during mechanically induced osteogenic lineage commitment. J Biomech Eng; 137(2):020902.

Cheng B, Zhao S, Luo J, Sprague E, Bonewald LF, Jiang JX (2001). Expression of functional gap junctions and regulation by fluid flow in osteocyte-like MLO-Y4 cells. J Bone Miner Res; 16(2):249-59.

Cherian PP, Siller-Jackson AJ, Gu S, Wang X, Bonewald LF, Sprague E, Jiang JX (2005). Mechanical strain opens connexin 43 hemichannels in osteocytes: a novel mechanism for the release of prostaglandin. Mol Biol Cell; 16(7):3100-6.

de Castro LF, Maycas M, Bravo B, Esbrit P, Gortazar A (2015). VEGF Receptor 2 (VEGFR2) Activation Is Essential for Osteocyte Survival Induced by Mechanotransduction. J Cell Physiol; 230(2):278-85.

Deepak V, Kayastha P, McNamara LM (2017). Estrogen deficiency attenuates fluid flow-induced [Ca(2+)](i) oscillations and mechanoresponsiveness of MLO-Y4 osteocytes. FASEB J; 31(7):3027-3039.

Fahlgren A, Bratengeier C, Semeins CM, Klein-Nulend J, Bakker AD (2018). Supraphysiological loading induces osteocyte-mediated osteoclastogenesis in a novel in vitro model for bone implant loosening. J Orthop Res; 36(5):1425-1434.

Genetos DC, Kephart CJ, Zhang Y, Yellowley CE, Donahue HJ (2007). Oscillating fluid flow activation of gap junction hemichannels induces ATP release from MLO-Y4 osteocytes. J Cell Physiol; 212(1):207-14.

Geoghegan IP, Hoey DA, McNamara LM (2019). Estrogen deficiency impairs integrin α(v)β(3)-mediated mechanosensation by osteocytes and alters osteoclastogenic paracrine signalling. Sci Rep; 9(1):4654.

González Á, García de Durango C, Alonso V, Bravo B, Rodríguez de Gortázar A, Wells A, Forteza J, Vidal-Vanaclocha F (2017). Distinct Osteomimetic Response of Androgen-Dependent and Independent Human Prostate Cancer Cells to Mechanical Action of Fluid Flow: Prometastatic Implications. Prostate; 77(3):321-333.

Haugh MG, Vaughan TJ, McNamara LM (2015). The role of integrin α(V)β(3) in osteocyte mechanotransduction. J Mech Behav Biomed Mater; 42:67-75.

Hoey DA, Tormey S, Ramcharan S, O'Brien FJ, Jacobs CR (2012). Primary cilia-mediated mechanotransduction in human mesenchymal stem cells. Stem Cells; 30(11):2561-70.

Hu K, Sun H, Gui B, Sui C (2017). TRPV4 functions in flow shear stress induced early osteogenic differentiation of human bone marrow mesenchymal stem cells. Biomed Pharmacother; 91:841-848.

Huang J, Romero-Suarez S, Lara N, Mo C, Kaja S, Brotto L, Dallas SL, Johnson ML, Jähn K, Bonewald LF, Brotto M (2017). Crosstalk between MLO-Y4 osteocytes and C2C12 muscle cells is mediated by the Wnt/β-catenin pathway. JBMR Plus; 1(2):86-100.

Jing D, Lu XL, Luo E, Sajda P, Leong PL, Guo XE (2013). Spatiotemporal properties of intracellular calcium signaling in osteocytic and osteoblastic cell networks under fluid flow. Bone; 53(2):531-40.

Joldersma M, Burger EH, Semeins CM, Klein-Nulend J (2000). Mechanical stress induces COX-2 mRNA expression in bone cells from elderly women. J Biomech; 33(1):53-61.

Joldersma M, Klein-Nulend J, Oleksik AM, Heyligers IC, Burger EH (2001). Estrogen enhances mechanical stress-induced prostaglandin production by bone cells from elderly women. Am J Physiol Endocrinol Metab; 280(3):E436-42.

Juffer P, Jaspers RT, Lips P, Bakker AD, Klein-Nulend J (2012). Expression of muscle anabolic and metabolic factors in mechanically loaded MLO-Y4 osteocytes. Am J Physiol Endocrinol Metab; 302(4):E389-95.

Kamel MA, Picconi JL, Lara-Castillo N, Johnson ML (2010). Activation of β-catenin signaling in MLO-Y4 osteocytic cells versus 2T3 osteoblastic cells by fluid flow shear stress and PGE2: Implications for the study of mechanosensation in bone. Bone; 47(5):872-81.

Kapur S, Amoui M, Kesavan C, Wang X, Mohan S, Baylink DJ, Lau KH (2010). Leptin receptor (Lepr) is a negative modulator of bone mechanosensitivity and genetic variations in Lepr may contribute to the differential osteogenic response to mechanical stimulation in the C57BL/6J and C3H/HeJ pair of mouse strains. J Biol Chem; 285(48):37607-18.

Klein-Nulend J, Semeins CM, Burger EH (1996). Prostaglandin mediated modulation of transforming growth factor-beta metabolism in primary mouse osteoblastic cells in vitro. J Cell Physiol; 168(1):1-7.

Klein-Nulend J, Burger EH, Semeins CM, Raisz LG, Pilbeam CC (1997). Pulsating fluid flow stimulates prostaglandin release and inducible prostaglandin G/H synthase mRNA expression in primary mouse bone cells. J Bone Miner Res; 12(1):45-51.

Klein-Nulend J, Helfrich MH, Sterck JG, MacPherson H, Joldersma M, Ralston SH, Semeins CM, Burger EH (1998). Nitric oxide response to shear stress by human bone cell cultures is

endothelial nitric oxide synthase dependent. Biochem Biophys Res Commun; 250(1):108-14.
Klein-Nulend J, Sterck JG, Semeins CM, Lips P, Joldersma M, Baart JA, Burger EH (2002). Donor age and mechanosensitivity of human bone cells. Osteoporos Int; 13(2):137-46.
Kraft DC, Bindslev DA, Melsen B, Abdallah BM, Kassem M, Klein-Nulend J (2010). Mechanosensitivity of dental pulp stem cells is related to their osteogenic maturity. Eur J Oral Sci; 118(1):29-38.
Kraft DC, Bindslev DA, Melsen B, Klein-Nulend J (2011). Human dental pulp cells exhibit bone cell-like responsiveness to fluid shear stress. Cytotherapy; 13(2):214-26.
Kulkarni RN, Bakker AD, Everts V, Klein-Nulend J (2010). Inhibition of osteoclastogenesis by mechanically loaded osteocytes: involvement of MEPE. Calcif Tissue Int; 87(5):461-8.
Kulkarni RN, Bakker AD, Everts V, Klein-Nulend J (2012a). Mechanical loading prevents the stimulating effect of IL-1β on osteocyte-modulated osteoclastogenesis. Biochem Biophys Res Commun; 420(1):11-6.
Kulkarni RN, Bakker AD, Gruber EV, Chae TD, Veldkamp JB, Klein-Nulend J, Everts V (2012b). MT1-MMP modulates the mechanosensitivity of osteocytes. Biochem Biophys Res Commun; 417(2):824-9.
Kuo YC, Chang TH, Hsu WT, Zhou J, Lee HH, Hui-Chun Ho J, Chien S, Lee OK (2015). Oscillatory shear stress mediates directional reorganization of actin cytoskeleton and alters differentiation propensity of mesenchymal stem cells. Stem Cells; 33(2):429-42.
Lau KH, Kapur S, Kesavan C, Baylink DJ (2006). Up-regulation of the Wnt, estrogen receptor, insulin-like growth factor-I, and bone morphogenetic protein pathways in C57BL/6J osteoblasts as opposed to C3H/HeJ osteoblasts in part contributes to the differential anabolic response to fluid shear. J Biol Chem; 281(14):9576-88.
Lee HJ, Diaz MF, Ewere A, Olson SD, Cox CS, Jr., Wenzel PL (2017). Focal adhesion kinase signaling regulates anti-inflammatory function of bone marrow mesenchymal stromal cells induced by biomechanical force. Cell Signal; 38:1-9.
Li J, Liu D, Ke HZ, Duncan RL, Turner CH (2005). The P2X7 nucleotide receptor mediates skeletal mechanotransduction. J Biol Chem; 280(52):42952-9.
Li J, Rose E, Frances D, Sun Y, You L (2012). Effect of oscillating fluid flow stimulation on osteocyte mRNA expression. J Biomech; 45(2):247-51.
Li X, Liu C, Li P, Li S, Zhao Z, Chen Y, Huo B, Zhang D (2013). Connexin 43 is a potential regulator in fluid shear stress-induced signal transduction in osteocytes. J Orthop Res; 31(12):1959-65.
Li X, Han L, Nookaew I, Mannen E, Silva MJ, Almeida M, Xiong J (2019). Stimulation of Piezo1 by mechanical signals promotes bone anabolism. Elife; 8.
Li YJ, Batra NN, You L, Meier SC, Coe IA, Yellowley CE, Jacobs CR (2004). Oscillatory fluid flow affects human marrow stromal cell proliferation and differentiation. J Orthop Res; 22(6):1283-9.
Liao C, Cheng T, Wang S, Zhang C, Jin L, Yang Y (2017). Shear stress inhibits IL-17A-mediated induction of osteoclastogenesis via osteocyte pathways. Bone; 101:10-20.
Lim KT, Kim J, Seonwoo H, Chang JU, Choi H, Hexiu J, Cho WJ, Choung PH, Chung JH (2013). Enhanced osteogenesis of human alveolar bone-derived mesenchymal stem cells for tooth tissue engineering using fluid shear stress in a rocking culture method. Tissue Eng Part C Methods; 19(2):128-45.
Lim KT, Hexiu J, Kim J, Seonwoo H, Choung PH, Chung JH (2014). Synergistic effects of orbital shear stress on in vitro growth and osteogenic differentiation of human alveolar bone-derived mesenchymal stem cells. Biomed Res Int; 2014:316803.
Litzenberger JB, Kim JB, Tummala P, Jacobs CR (2010). Beta1 integrins mediate mechanosensitive signaling pathways in osteocytes. Calcif Tissue Int; 86(4):325-32.
Liu C, Zhang X, Wu M, You L (2015). Mechanical loading up-regulates early remodeling signals from osteocytes subjected to physical damage. J Biomech; 48(16):4221-8.
Lu XL, Huo B, Chiang V, Guo XE (2012a). Osteocytic network is more responsive in calcium signaling than osteoblastic network under fluid flow. J Bone Miner Res; 27(3):563-74.
Lu XL, Huo B, Park M, Guo XE (2012b). Calcium response in osteocytic networks under steady and oscillatory fluid flow. Bone; 51(3):466-73.
Maycas M, Ardura JA, de Castro LF, Bravo B, Gortázar AR, Esbrit P (2015). Role of the Parathyroid Hormone Type 1 Receptor (PTH1R) as a Mechanosensor in Osteocyte Survival. J Bone Miner Res; 30(7):1231-44.
Maycas M, Portolés MT, Matesanz MC, Buendía I, Linares J, Feito MJ, Arcos D, Vallet-Regí M, Plotkin LI, Esbrit P, Gortázar AR (2017). High glucose alters the secretome of mechanically stimulated osteocyte-like cells affecting osteoclast precursor recruitment and differentiation. J Cell Physiol; 232(12):3611-3621.
McGarry JG, Klein-Nulend J, Mullender MG, Prendergast PJ (2005). A comparison of strain and fluid shear stress in stimulating bone cell responses--a computational and experimental study. FASEB J; 19(3):482-4.
Mehrotra M, Saegusa M, Wadhwa S, Voznesensky O, Peterson D, Pilbeam C (2006). Fluid flow induces Rankl expression in primary murine calvarial osteoblasts. J Cell Biochem; 98(5):1271-83.
Middleton K, Kondiboyina A, Borrett M, Cui Y, Mei X, You L (2018). Microfluidics approach to investigate the role of dynamic similitude in osteocyte mechanobiology. J Orthop Res; 36(2):663-671.
Qi L, Zhang Y (2014). The microRNA 132 regulates fluid shear stress-induced differentiation in periodontal ligament cells through mTOR signaling pathway. Cell Physiol Biochem; 33(2):433-45.
Rath AL, Bonewald LF, Ling J, Jiang JX, Van Dyke ME, Nicolella DP (2010). Correlation of cell strain in single osteocytes with intracellular calcium, but not intracellular nitric oxide, in response to fluid flow. J Biomech; 43(8):1560-4.
Reilly GC, Haut TR, Yellowley CE, Donahue HJ, Jacobs CR (2003). Fluid flow induced PGE2 release by bone cells is reduced by glycocalyx degradation whereas calcium signals are not. Biorheology; 40(6):591-603.
Ren J, Wang XH, Wang GC, Wu JH (2013). 17β estradiol regulation of connexin 43-based gap junction and mechanosensitivity through classical estrogen receptor pathway in osteocyte-like MLO-Y4 cells. Bone; 53(2):587-96.
Riddle RC, Taylor AF, Genetos DC, Donahue HJ (2006). MAP kinase and calcium signaling mediate fluid flow-induced human mesenchymal stem cell proliferation. Am J Physiol Cell Physiol; 290(3):C776-84.
Riddle RC, Taylor AF, Rogers JR, Donahue HJ (2007). ATP release mediates fluid flow-induced proliferation of human bone marrow stromal cells. J Bone Miner Res; 22(4):589-600.
Riquelme MA, Gu S, Hua R, Jiang JX (2021). Mechanotransduction via the coordinated actions of integrins, PI3K signaling and Connexin hemichannels. Bone Res; 9(1):8.
Salvi JD, Lim JY, Donahue HJ (2010). Increased mechanosensitivity of cells cultured on nanotopographies. J Biomech; 43(15):3058-62.
Santos A, Bakker AD, Zandieh-Doulabi B, Semeins CM, Klein-Nulend J (2009). Pulsating fluid flow modulates gene expression of proteins involved in Wnt signaling pathways in osteocytes. J Orthop Res; 27(10):1280-7.
Santos A, Bakker AD, Zandieh-Doulabi B, de Blieck-Hogervorst JM, Klein-Nulend J (2010). Early activation of the beta-catenin pathway in osteocytes is mediated by nitric oxide, phosphatidyl inositol-3 kinase/Akt, and focal adhesion kinase. Biochem Biophys Res Commun; 391(1):364-9.
Santos A, Bakker AD, Willems HM, Bravenboer N, Bronckers AL, Klein-Nulend J (2011). Mechanical loading stimulates BMP7, but not BMP2, production by osteocytes. Calcif Tissue Int; 89(4):318-26.
Seref-Ferlengez Z, Maung S, Schaffler MB, Spray DC, Suadicani SO, Thi MM (2016). P2X7R-Panx1 Complex Impairs Bone Mechanosignaling under High Glucose Levels Associated with Type-1 Diabetes. PLoS One; 11(5):e0155107.
Shah KM, Orton P, Mani N, Wilkinson JM, Gartland A (2017). Osteocyte physiology and response to fluid shear stress are impaired following exposure to cobalt and chromium: Implications for bone health following joint replacement. J Orthop Res; 35(8):1716-1723.
Soejima K, Klein-Nulend J, Semeins CM, Burger EH (2001). Different responsiveness of cells from adult and neonatal mouse bone

to mechanical and biochemical challenge. J Cell Physiol; 186(3):366-70.
Sonam S, Sathe SR, Yim EK, Sheetz MP, Lim CT (2016). Cell contractility arising from topography and shear flow determines human mesenchymal stem cell fate. Scientific Reports; 6:20415.
Sterck JG, Klein-Nulend J, Lips P, Burger EH (1998). Response of normal and osteoporotic human bone cells to mechanical stress in vitro. Am J Physiol; 274(6):E1113-20.
Tang M, Peng Z, Mai Z, Chen L, Mao Q, Chen Z, Chen Q, Liu L, Wang Y, Ai H (2014). Fluid shear stress stimulates osteogenic differentiation of human periodontal ligament cells via the extracellular signal-regulated kinase 1/2 and p38 mitogen-activated protein kinase signaling pathways. J Periodontol; 85(12):1806-13.
Thi MM, Kojima T, Cowin SC, Weinbaum S, Spray DC (2003). Fluid shear stress remodels expression and function of junctional proteins in cultured bone cells. Am J Physiol Cell Physiol; 284(2):C389-403.
Thi MM, Suadicani SO, Spray DC (2010). Fluid flow-induced soluble vascular endothelial growth factor isoforms regulate actin adaptation in osteoblasts. J Biol Chem; 285(40):30931-41.
Thi MM, Islam S, Suadicani SO, Spray DC (2012). Connexin43 and pannexin1 channels in osteoblasts: who is the "hemichannel"? J Membr Biol; 245(7):401-9.
van der Meijden K, Bakker AD, van Essen HW, Heijboer AC, Schulten EA, Lips P, Bravenboer N (2016). Mechanical loading and the synthesis of 1,25(OH)2D in primary human osteoblasts. J Steroid Biochem Mol Biol; 156:32-9.
Wang S, Li S, Hu M, Huo B (2019). Calcium response in bone cells at different osteogenic stages under unidirectional or oscillatory flow. Biomicrofluidics; 13(6):064117.
Xia X, Batra N, Shi Q, Bonewald LF, Sprague E, Jiang JX (2010). Prostaglandin promotion of osteocyte gap junction function through transcriptional regulation of connexin 43 by glycogen synthase kinase 3/beta-catenin signaling. Mol Cell Biol; 30(1):206-19.
Xing Y, Gu Y, Bresnahan JJ, Paul EM, Donahue HJ, You J (2014). The roles of P2Y2 purinergic receptors in osteoblasts and mechanotransduction. PLoS One; 9(9):e108417.
Xu H, Zhang J, Wu J, Guan Y, Weng Y, Shang P (2012). Oscillatory fluid flow elicits changes in morphology, cytoskeleton and integrin-associated molecules in MLO-Y4 cells, but not in MC3T3-E1 cells. Biol Res; 45(2):163-9.
Xu H, Guan Y, Wu J, Zhang J, Duan J, An L, Shang P (2014). Polycystin 2 is involved in the nitric oxide production in responding to oscillating fluid shear in MLO-Y4 cells. J Biomech; 47(2):387-91.
Yan Z, Wang P, Wu J, Feng X, Cai J, Zhai M, Li J, Liu X, Jiang M, Luo E, Jing D (2018). Fluid shear stress improves morphology, cytoskeleton architecture, viability, and regulates cytokine expression in a time-dependent manner in MLO-Y4 cells. Cell Biol Int; 42(10):1410-1422.
Yang Z, Bidwell JP, Young SR, Gerard-O'Riley R, Wang H, Pavalko FM (2010). Nmp4/CIZ inhibits mechanically induced beta-catenin signaling activity in osteoblasts. J Cell Physiol; 223(2):435-41.
Yang Z, Tan S, Shen Y, Chen R, Wu C, Xu Y, Song Z, Fu Q (2015). Inhibition of FSS-induced actin cytoskeleton reorganization by silencing LIMK2 gene increases the mechanosensitivity of primary osteoblasts. Bone; 74:182-90.
Yourek G, McCormick SM, Mao JJ, Reilly GC (2010). Shear stress induces osteogenic differentiation of human mesenchymal stem cells. Regen Med; 5(5):713-24.
Yuan L, Sakamoto N, Song G, Sato M (2012). Migration of human mesenchymal stem cells under low shear stress mediated by mitogen-activated protein kinase signaling. Stem Cells Dev; 21(13):2520-30.
Zhang JN, Zhao Y, Liu C, Han ES, Yu X, Lidington D, Bolz SS, You L (2015). The role of the sphingosine-1-phosphate signaling pathway in osteocyte mechanotransduction. Bone; 79:71-8.
Zhang K, Barragan-Adjemian C, Ye L, Kotha S, Dallas M, Lu Y, Zhao S, Harris M, Harris SE, Feng JQ, Bonewald LF (2006). E11/gp38 selective expression in osteocytes: regulation by mechanical strain and role in dendrite elongation. Mol Cell Biol; 26(12):4539-52.
Zheng L, Chen L, Chen Y, Gui J, Li Q, Huang Y, Liu M, Jia X, Song W, Ji J, Gong X, Shi R, Fan Y (2016). The effects of fluid shear stress on proliferation and osteogenesis of human periodontal ligament cells. J Biomech; 49(4):572-9.
