# Supplementary material for: Fluid flow shear stress and tissue remodeling—an orthodontic perspective: evidence synthesis and differential gene expression network analysis
Source: Front Bioeng Biotechnol. 2023 Sep 18;11:1256825. doi: 10.3389/fbioe.2023.1256825 (PMC10545883; doi:10.3389/fbioe.2023.1256825)
Supplement: Supplementary file 5 [file DataSheet3.pdf]

Supplement 3

"Fluid Flow Shear Stress and Tissue Remodeling – an Orthodontic Perspective: Evidence Synthesis and Differential Gene Expression Network analysis"

Risk of bias analysis

Table of contents

REVISED ROB EXTRACTION SHEETS 2

3.1.1 REPORTING RISK OF BIAS........ 2S
3.1.2 METHODOLOGICAL RISK OF BIAS 3

HUMAN MESENCHYMAL STEM CELLS 4

3.2 HUMAN MESENCHYMAL STEM CELLS – REPORTING RISK OF BIAS 4
3.3 HUMAN MESENCHYMAL STEM CELLS – METHODOLOGICAL RISK OF BIAS 5
REFERENCES 6

HUMAN OSTEOBLASTS 7

3.4 HUMAN OSTEOBLASTS – REPORTING RISK OF BIAS 7
3.5 HUMAN OSTEOBLASTS – METHODOLOGICAL RISK OF BIAS 8
REFERENCES 8

HUMAN PERIODONTAL LIGAMENT CELLS 10

3.6 HUMAN PERIODONTAL LIGAMENT CELLS – REPORTING RISK OF BIAS 10
3.7 HUMAN PERIODONTAL LIGAMENT CELLS – METHODOLOGICAL RISK OF BIAS 11
REFERENCES 11

MOUSE OSTEOBLASTS 12

3.8 MOUSE OSTEOBLASTS – REPORTING RISK OF BIAS 12
3.9 MOUSE OSTEOBLASTS – METHODOLOGICAL RISK OF BIAS........ 13
REFERENCES 14

MOUSE OSTEOCYTES 15

3.10 MOUSE OSTEOCYTES – REPORTING RISK OF BIAS 15
3.11 MOUSE OSTEOCYTES – METHODOLOGICAL RISK OF BIAS 17
REFERENCES 19

Revised RoB extraction sheets

3.1.1 Reporting risk of bias

Reporting quality of *in vitro* studies

**Reference**		
**#1 Description of scientific background**		
**#2 Description objective**		
**#3 Justification for model**	☐<br>☐<br>☐<br>☐<br>☐	Cell selection<br>Force selection<br>Force parameter: magnitude<br>Force parameter: duration<br>Force parameter: frequency
**#4 Study design description**		
**#5 Defined experimental outcomes**		
**#6 Ethical statement**		
**#7 Cell maintenance condition**	☐<br><br>☐<br>☐<br>☐<br>☐<br>☐	Source of cells (primary cells and cell lines): information on donor (e.g. sex, age), company or cell culture collection, or similar<br>Cell isolation method (primary cells)<br>Cell culture conditions (both)<br>Passage number used in experiments (primary cells)<br>Cell seeding density used in experiments (both)<br>Cell confluency level (both)
**#8 Description of measurement precision and variability**		
**#9 Statistical analysis**		
**#10 Results description**		

Levels:

"+"	Low risk of bias (LoB)
"-"	High risk of bias (HoB)
"?"	Incomplete/unclear risk of bias
n. a.	Not applicable

3.1.2 Methodological risk of bias

Methodological quality of *in vitro* studies

Reference

**#1* Selection bias / Baseline characteristics similarity/appropriate control group selection**	
**#2 Selection bias / Allocation concealment**	
**#3 Selection bias / Randomization**	
**#4 Performance bias / Blinding of researchers**	
**#5 Detection bias / Blinding of outcome assessors**	
**#6 Attrition bias / Complete outcome data**	
**#7 Reporting bias / Selective outcome data**	
**#8 Confounding bias / Account for confounding variables**	
**#9 Appropriate statistical methods / Sample size determination**	
**#10 Appropriate statistical methods / Statistical analysis**	
**#11* Appropriate/controlled exposure (incl. characterization)**	
**#12* Optimal time window used**	☐ Force type<br>☐ Force duration<br>☐ Force magnitude<br>☐ Force frequency (if applicable)<br>☐ Controls appropriate (*refer to #1 in this RoB assessment*)
**#13 Statement conflict of interest/funding source**	
**#14* Test substance/treatment details**	
**#15 Test organism/system**	☐ Source of cells (primary cells and cell lines): information on donor (e.g. sex, age), company or cell culture collection, or similar<br>☐ Cell isolation method (primary cells)<br>☐ Cell culture conditions (both)<br>☐ Passage number used in experiments (primary cells)<br>☐ Cell seeding density used in experiments (both)<br>☐ Cell confluency level (both)

Levels:

“+” Low risk of bias (LoB)
“-“ High risk of bias (HoB)
“?” Incomplete/unclear risk of bias
n. a. Not applicable

Human mesenchymal stem cells

3.2 Human mesenchymal stem cells – Reporting risk of bias

Reference	Description of scientific background	Description objective	Justification for model	Study design description	Defined experimental outcomes	Ethical statement	Cell maintenance condition	Description of measurement precision and variability	Statistical analysis	Results description
Yourek et al. (2010)	+	+	?	+	+	+	?	?	?	+
Kraft et al. (2011)	+	+	?	+	?	+	?	?	?	?
Sonam et al. (2016)	+	+	?	+	+	-	?	?	?	+
Hu et al. (2017)	+	+	?	+	+	+	?	?	?	+
Yuan et al. (2012)	+	+	?	+	+	-	?	?	?	+
Kim et al. (2011)	+	+	?	+	+	-	?	?	?	+
Kraft et al. (2010)	+	+	?	+	?	+	?	?	?	?
Glossop and Cartmell (2009)	+	+	?	+	?	-	?	?	?	?
Lim et al. (2013)	+	+	?	+	+	+	?	?	?	+
Salvi et al. (2010)	+	+	+	?	+	-	?	?	?	+
Celil Aydemir et al. (2010)	+	+	?	+	+	-	?	?	?	+
Celil Aydemir et al. (2007)	+	+	?	+	+	-	?	?	?	+
Hoey et al. (2012)	+	+	?	+	?	-	?	?	?	?
Kuo et al. (2015)	+	+	?	+	+	-	?	?	?	+
Riddle et al. (2006)	+	+	?	+	?	-	?	?	?	?
Lim et al. (2014)	+	+	?	+	+	-	?	?	?	+
Becquart et al. (2016)	+	+	?	+	?	+	?	?	?	?
Lee et al. (2017)	+	+	?	+	+	-	?	+	+	+
Riddle et al. (2007)	+	+	?	+	?	-	?	?	?	?
Li et al. (2004)	+	+	+	+	+	-	?	?	?	+
Charoenpong et al. (2019)	+	+	?	+	+	+	?	?	?	+
Summary										
Low risk of bias "+"	21 (100%)	21 (100%)	2 (10%)	20 (95%)	14 (67%)	7 (33%)	0 (0%)	1 (5%)	1 (5%)	14 (67%)
Incomplete/unclear risk of bias "?"	0 (0%)	0 (0%)	19 (90%)	1 (5%)	7 (33%)	0 (0%)	21 (100%)	20 (95%)	20 (95%)	7 (33%)
High risk of bias "-"	0 (0%)	0 (0%)	0 (0%)	0 (0%)	0 (0%)	14 (67%)	0 (0%)	0 (0%)	0 (0%)	0 (0%)
Not applicable "n. a."	0 (0%)	0 (0%)	0 (0%)	0 (0%)	0 (0%)	0 (0%)	0 (0%)	0 (0%)	0 (0%)	0 (0%)
Sum	21	21	21	21	21	21	21	21	21	21

3.3 Human mesenchymal stem cells – Methodological risk of bias

Reference	Selection bias			Performance bias	Detection bias	Attrition bias	Reporting bias	Confounding bias	Appropriate statistical methods		Appropriate/ controlled exposure (incl. characterization)	Optimal time window used	Statement conflict of interest/funding source	Test substance/treatment details	Test organism/system
	Baseline characteristics similarity / appropriate control group selection	Allocation concealment	Randomization	Blinding of researchers	Blinding of outcome assessors	Complete outcome data	Selective outcome data	Account for confounding variables	Sample size determination	Statistical analysis					
Yourek et al. (2010)	?	?	n.a.	n.a.	n.a.	+	+	?	-	-	?	?	?	?	?
Kraft et al. (2011)	?	?	n.a.	n.a.	n.a.	-	?	?	-	-	?	?	+	?	?
Sonam et al. (2016)	?	?	n.a.	n.a.	n.a.	+	+	?	-	-	?	?	-	?	?
Hu et al. (2017)	?	?	n.a.	n.a.	n.a.	+	+	?	-	-	?	?	+	?	?
Yuan et al. (2012)	?	?	n.a.	n.a.	n.a.	+	+	?	-	-	?	-	+	?	?
Kim et al. (2011)	+	?	n.a.	n.a.	n.a.	+	+	?	-	-	?	?	?	?	?
Kraft et al. (2010)	?	?	n.a.	n.a.	n.a.	-	?	?	-	-	?	?	?	?	?
Glossop and Cartmell (2009)	+	?	n.a.	n.a.	n.a.	?	?	?	-	-	?	?	?	?	?
Lim et al. (2013)	-	?	n.a.	n.a.	n.a.	+	+	?	-	-	?	-	?	?	?
Salvi et al. (2010)	?	?	n.a.	n.a.	n.a.	+	+	?	-	-	?	?	+	?	?
Celil Aydemir et al. (2010)	?	?	n.a.	n.a.	n.a.	+	+	?	-	-	?	?	?	?	?
Celil Aydemir et al. (2007)	?	?	n.a.	n.a.	n.a.	+	+	?	-	-	?	?	-	?	?
Hoey et al. (2012)	?	?	n.a.	n.a.	n.a.	-	?	?	-	-	?	?	+	?	?
Kuo et al. (2015)	?	?	n.a.	n.a.	n.a.	+	+	?	-	-	?	?	+	?	?
Riddle et al. (2006)	?	?	n.a.	n.a.	n.a.	-	?	?	-	-	?	?	?	?	?
Lim et al. (2014)	?	?	n.a.	n.a.	n.a.	+	+	?	-	-	?	?	+	?	?
Becquart et al. (2016)	?	?	n.a.	n.a.	n.a.	-	?	?	-	-	?	?	+	?	?
Lee et al. (2017)	+	?	n.a.	n.a.	n.a.	+	+	?	-	?	?	?	+	?	?
Riddle et al. (2007)	?	?	n.a.	n.a.	n.a.	-	?	?	-	-	?	?	?	?	?
Li et al. (2004)	?	?	n.a.	n.a.	n.a.	+	+	?	-	-	?	?	?	?	?
Charoenpong et al. (2019)	?	?	n.a.	n.a.	n.a.	+	+	?	-	-	?	?	+	?	?
Summary															
Low risk of bias "+"	3 (14%)	0 (0%)	0 (0%)	0 (0%)	0 (0%)	14 (67%)	14 (67%)	0 (0%)	0 (0%)	0 (0%)	0 (0%)	0 (0%)	10 (48%)	0 (0%)	0 (0%)
Incomplete/unclear risk of bias "?"	17 (81%)	21 (100%)	0 (0%)	0 (0%)	0 (0%)	1 (5%)	7 (33%)	21 (100%)	0 (0%)	1 (5%)	21 (100%	19 (90%)	9 (43%)	21 (100%)	21 (100%)
High risk of bias "-"	1 (5%)	0 (0%)	0 (0%)	0 (0%)	0 (0%)	6 (29%)	0 (0%)	0 (0%)	21 (100%)	20 (95%)	0 (0%)	2 (10%)	2 (10%)	0 (0%)	0 (0%)
Not applicable "n. a."	0 (0%)	0 (0%)	21 (100%)	21 (100%)	21 (100%)	0 (0%)	0 (0%)	0 (0%)	0 (0%)	0 (0%)	0 (0%)	0 (0%)	0 (0%)	0 (0%)	0 (0%)
Sum	21	21	21	21	21	21	21	21	21	21	21	21	21	21	21

References

Becquart P, Cruel M, Hoc T, Sudre L, Pernelle K, Bizios R, Logeart-Avramoglou D, Petite H, Bensidhoum M (2016). Human mesenchymal stem cell responses to hydrostatic pressure and shear stress. Eur Cell Mater; 31:160-73.

Celil Aydemir AB, Lee S, Won Kim D, Gardner TR, Prince D, Mok Ahn J, Lee FY (2007). Nuclear factor of activated T cell mediates proinflammatory gene expression in response to mechanotransduction. Ann N Y Acad Sci; 1117:138-42.

Celil Aydemir AB, Minematsu H, Gardner TR, Kim KO, Ahn JM, Lee FY (2010). Nuclear factor of activated T cells mediates fluid shear stress- and tensile strain-induced Cox2 in human and murine bone cells. Bone; 46(1):167-75.

Charoenpong H, Osathanon T, Pavasant P, Limjeerajarus N, Keawprachum B, Limjeerajarus CN, Cheewinthamrongrod V, Palaga T, Lertchirakarn V, Ritprajak P (2019). Mechanical stress induced S100A7 expression in human dental pulp cells to augment osteoclast differentiation. Oral Dis; 25(3):812-821.

Glossop JR, Cartmell SH (2009). Effect of fluid flow-induced shear stress on human mesenchymal stem cells: differential gene expression of IL1B and MAP3K8 in MAPK signaling. Gene Expression Patterns; 9(5):381-8.

Hoey DA, Tormey S, Ramcharan S, O'Brien FJ, Jacobs CR (2012). Primary cilia-mediated mechanotransduction in human mesenchymal stem cells. Stem Cells; 30(11):2561-70.

Hu K, Sun H, Gui B, Sui C (2017). TRPV4 functions in flow shear stress induced early osteogenic differentiation of human bone marrow mesenchymal stem cells. Biomed Pharmacother; 91:841-848.

Kim DH, Heo SJ, Kim SH, Shin JW, Park SH, Shin JW (2011). Shear stress magnitude is critical in regulating the differentiation of mesenchymal stem cells even with endothelial growth medium. Biotechnol Lett; 33(12):2351-9.

Kraft DC, Bindslev DA, Melsen B, Abdallah BM, Kassem M, Klein-Nulend J (2010). Mechanosensitivity of dental pulp stem cells is related to their osteogenic maturity. Eur J Oral Sci; 118(1):29-38.

Kraft DC, Bindslev DA, Melsen B, Klein-Nulend J (2011). Human dental pulp cells exhibit bone cell-like responsiveness to fluid shear stress. Cytotherapy; 13(2):214-26.

Kuo YC, Chang TH, Hsu WT, Zhou J, Lee HH, Hui-Chun Ho J, Chien S, Lee OK (2015). Oscillatory shear stress mediates directional reorganization of actin cytoskeleton and alters differentiation propensity of mesenchymal stem cells. Stem Cells; 33(2):429-42.

Lee HJ, Diaz MF, Ewere A, Olson SD, Cox CS, Jr., Wenzel PL (2017). Focal adhesion kinase signaling regulates anti-inflammatory function of bone marrow mesenchymal stromal cells induced by biomechanical force. Cell Signal; 38:1-9.

Li YJ, Batra NN, You L, Meier SC, Coe IA, Yellowley CE, Jacobs CR (2004). Oscillatory fluid flow affects human marrow stromal cell proliferation and differentiation. J Orthop Res; 22(6):1283-9.

Lim KT, Kim J, Seonwoo H, Chang JU, Choi H, Hexiu J, Cho WJ, Choung PH, Chung JH (2013). Enhanced osteogenesis of human alveolar bone-derived mesenchymal stem cells for tooth tissue engineering using fluid shear stress in a rocking culture method. Tissue Eng Part C Methods; 19(2):128-45.

Lim KT, Hexiu J, Kim J, Seonwoo H, Choung PH, Chung JH (2014). Synergistic effects of orbital shear stress on in vitro growth and osteogenic differentiation of human alveolar bone-derived mesenchymal stem cells. Biomed Res Int; 2014:316803.

Riddle RC, Taylor AF, Genetos DC, Donahue HJ (2006). MAP kinase and calcium signaling mediate fluid flow-induced human mesenchymal stem cell proliferation. Am J Physiol Cell Physiol; 290(3):C776-84.

Riddle RC, Taylor AF, Rogers JR, Donahue HJ (2007). ATP release mediates fluid flow-induced proliferation of human bone marrow stromal cells. J Bone Miner Res; 22(4):589-600.

Salvi JD, Lim JY, Donahue HJ (2010). Increased mechanosensitivity of cells cultured on nanotopographies. J Biomech; 43(15):3058-62.

Sonam S, Sathe SR, Yim EK, Sheetz MP, Lim CT (2016). Cell contractility arising from topography and shear flow determines human mesenchymal stem cell fate. Scientific Reports; 6:20415.

Yourek G, McCormick SM, Mao JJ, Reilly GC (2010). Shear stress induces osteogenic differentiation of human mesenchymal stem cells. Regen Med; 5(5):713-24.

Yuan L, Sakamoto N, Song G, Sato M (2012). Migration of human mesenchymal stem cells under low shear stress mediated by mitogen-activated protein kinase signaling. Stem Cells Dev; 21(13):2520-30.

Human osteoblasts

3.4 Human osteoblasts – Reporting risk of bias

Reference	Description of scientific background	Description objective	Justification for model	Study design description	Defined experimental outcomes	Ethical statement	Cell maintenance condition	Description of measurement precision and variability	Statistical analysis	Results description
Bakker et al. (2004)	+	+	+	+	+	+	?	+	+	+
McGarry et al. (2005)	+	+	?	+	?	-	?	-	-	+
Sterck et al. (1998)	+	+	?	+	+	+	?	+	+	+
Rangaswami et al. (2009)	+	+	+	+	+	-	?	?	?	+
Rangaswami et al. (2012)	+	-	?	+	+	-	?	?	?	+
Aisha et al. (2015)	+	+	+	+	+	-	?	?	?	+
Bakker et al. (2003)	+	+	?	+	?	+	?	?	?	?
Joldersma et al. (2000)	+	+	?	+	+	+	?	?	?	+
Ehnert et al. (2017)	+	+	?	+	+	+	?	?	?	+
Joldersma et al. (2001)	+	+	?	+	?	+	?	?	?	?
Klein-Nulend et al. (2002)	+	+	?	+	+	+	?	+	+	+
Santos et al. (2011)	+	+	?	+	+	+	?	?	?	+
van der Meijden et al. (2016)	+	+	?	+	+	+	?	?	?	+
Klein-Nulend et al. (1998)	+	+	?	+	+	+	?	+	+	+
Summary										
Low risk of bias "+"	14 (100%)	13 (93%)	3 (21%)	14 (100%	11 (79%)	10 (71%)	0 (0%)	4 (29%)	4 (29%)	12 (86%)
Incomplete/unclear risk of bias "?"	0 (0%)	0 (0%)	11 (79%)	0 (0%)	3 (21%)	0 (0%)	14 (100%)	9 (64%)	9 (64%)	2 (14%)
High risk of bias "-"	0 (0%)	1 (7%)	0 (0%)	0 (0%)	0 (0%)	4 (29%)	0 (0%)	1 (7%)	1 (7%)	0 (0%)
Not applicable "n. a."	0 (0%)	0 (0%)	0 (0%)	0 (0%)	0 (0%)	0 (0%)	0 (0%)	0 (0%)	0 (0%)	0 (0%)
Sum	14	14	14	14	14	14	14	14	14	14

3.5 Human osteoblasts – Methodological risk of bias

Reference	Selection bias			Perfor-mance bias	Detection bias	Attrition bias	Reporting bias	Confounding bias	Appropriate statistical methods		Appropriate/ controlled exposure (incl. characterization)	Optimal time window used	Statement conflict of interest/funding source	Test substance/treatment details	Test organism/system
	Baseline characteristics similarity / appropriate control group selection	Allocation concealment	Randomization	Blinding of researchers	Blinding of outcome assessors	Complete outcome data	Selective outcome data	Account for confounding variables	Sample size determination	Statistical analysis					
Bakker et al. (2004)	?	?	n.a.	n.a.	n.a.	+	+	?	-	?	?	+	-	?	?
McGarry et al. (2005)	-	?	n.a.	n.a.	n.a.	+	+	?	-	-	?	?	?	?	?
Sterck et al. (1998)	?	?	n.a.	n.a.	n.a.	+	+	?	-	?	?	?	-	?	?
Rangaswami et al. (2009)	-	?	n.a.	n.a.	n.a.	+	+	?	-	-	?	+	-	?	?
Rangaswami et al. (2012)	+	?	n.a.	n.a.	n.a.	+	+	?	-	-	?	?	-	?	?
Aisha et al. (2015)	+	?	n.a.	n.a.	n.a.	+	+	?	-	-	?	?	-	?	?
Bakker et al. (2003)	?	?	n.a.	n.a.	n.a.	-	-	?	-	-	?	?	?	?	?
Joldersma et al. (2000)	?	?	n.a.	n.a.	n.a.	+	+	?	-	-	?	?	?	?	?
Ehnert et al. (2017)	?	?	n.a.	n.a.	n.a.	+	+	?	-	-	?	?	?	?	?
Joldersma et al. (2001)	?	?	n.a.	n.a.	n.a.	-	?	?	-	-	?	?	?	?	?
Klein-Nulend et al. (2002)	+	?	n.a.	n.a.	n.a.	+	+	?	-	?	?	-	?	?	?
Santos et al. (2011)	+	?	n.a.	n.a.	n.a.	+	+	?	-	-	+	?	?	?	?
van der Meijden et al. (2016)	?	?	n.a.	n.a.	n.a.	+	+	?	+	+	?	?	-	?	?
Klein-Nulend et al. (1998)	?	?	n.a.	n.a.	n.a.	+	+	?	-	?	?	?	?	?	?
Summary															
Low risk of bias "+"	4 (29%)	0 (0%)	0 (0%)	0 (0%)	0 (0%)	12 (86%)	12 (86%)	0 (0%)	1 (7%)	1 (7%)	1 (7%)	2 (14%)	0 (0%)	0 (0%)	0 (0%)
Incomplete/unclear risk of bias "?"	8 (57%)	14 (100%)	0 (0%)	0 (0%)	0 (0%)	0 (0%)	1 (7%)	14 (100%)	0 (0%)	4 (29%)	13 (93%)	11 (79%)	8 (57%)	14 (100%)	14 (100%)
High risk of bias "-"	2 (14%)	0 (0%)	0 (0%)	0 (0%)	0 (0%)	2 (14%)	1 (7%)	0 (0%)	13 (93%)	9 (64%)	0 (0%)	1 (7%)	6 (43%)	0 (0%)	0 (0%)
Not applicable "n. a."	0 (0%)	0 (0%)	14 (100%	14 (100%)	14 (100%)	0 (0%)	0 (0%)	0 (0%)	0 (0%)	0 (0%)	0 (0%)	0 (0%)	0 (0%)	0 (0%)	0 (0%)
Sum	14	14	14	14	14	14	14	14	14	14	14	14	14	14	14

References

Aisha MD, Nor-Ashikin MN, Sharaniza AB, Nawawi H, Froemming GR (2015). Orbital fluid shear stress promotes osteoblast metabolism, proliferation and alkaline phosphates activity in vitro. Exp Cell Res; 337(1):87-93.

Bakker A, Klein-Nulend J, Burger E (2004). Shear stress inhibits while disuse promotes osteocyte apoptosis. Biochem Biophys Res Commun; 320(4):1163-8.

Bakker AD, Klein-Nulend J, Burger EH (2003). Mechanotransduction in bone cells proceeds via activation of COX-2, but not COX-1. Biochem Biophys Res Commun; 305(3):677-83.

Ehnert S, Sreekumar V, Aspera-Werz RH, Sajadian SO, Wintermeyer E, Sandmann GH, Bahrs C, Hengstler JG, Godoy P, Nussler AK (2017). TGF-β(1) impairs mechanosensation of human osteoblasts via HDAC6-mediated shortening and distortion of primary cilia. J Mol Med (Berl); 95(6):653-663.

Joldersma M, Burger EH, Semeins CM, Klein-Nulend J (2000). Mechanical stress induces COX-2 mRNA expression in bone cells from elderly women. J Biomech; 33(1):53-61.
Joldersma M, Klein-Nulend J, Oleksik AM, Heyligers IC, Burger EH (2001). Estrogen enhances mechanical stress-induced prostaglandin production by bone cells from elderly women. Am J Physiol Endocrinol Metab; 280(3):E436-42.
Klein-Nulend J, Helfrich MH, Sterck JG, MacPherson H, Joldersma M, Ralston SH, Semeins CM, Burger EH (1998). Nitric oxide response to shear stress by human bone cell cultures is endothelial nitric oxide synthase dependent. Biochem Biophys Res Commun; 250(1):108-14.
Klein-Nulend J, Sterck JG, Semeins CM, Lips P, Joldersma M, Baart JA, Burger EH (2002). Donor age and mechanosensitivity of human bone cells. Osteoporos Int; 13(2):137-46.
McGarry JG, Klein-Nulend J, Mullender MG, Prendergast PJ (2005). A comparison of strain and fluid shear stress in stimulating bone cell responses--a computational and experimental study. FASEB J; 19(3):482-4.
Rangaswami H, Marathe N, Zhuang S, Chen Y, Yeh JC, Frangos JA, Boss GR, Pilz RB (2009). Type II cGMP-dependent protein kinase mediates osteoblast mechanotransduction. J Biol Chem; 284(22):14796-808.
Rangaswami H, Schwappacher R, Tran T, Chan GC, Zhuang S, Boss GR, Pilz RB (2012). Protein kinase G and focal adhesion kinase converge on Src/Akt/β-catenin signaling module in osteoblast mechanotransduction. J Biol Chem; 287(25):21509-19.
Santos A, Bakker AD, Willems HM, Bravenboer N, Bronckers AL, Klein-Nulend J (2011). Mechanical loading stimulates BMP7, but not BMP2, production by osteocytes. Calcif Tissue Int; 89(4):318-26.
Sterck JG, Klein-Nulend J, Lips P, Burger EH (1998). Response of normal and osteoporotic human bone cells to mechanical stress in vitro. Am J Physiol; 274(6):E1113-20.
van der Meijden K, Bakker AD, van Essen HW, Heijboer AC, Schulten EA, Lips P, Bravenboer N (2016). Mechanical loading and the synthesis of 1,25(OH)2D in primary human osteoblasts. J Steroid Biochem Mol Biol; 156:32-9.

Human periodontal ligament cells

3.6 Human periodontal ligament cells – Reporting risk of bias

Reference	Description of scientific background	Description objective	Justification for model	Study design description	Defined experimental outcomes	Ethical statement	Cell maintenance condition	Description of measurement precision and variability	Statistical analysis	Results description
Zheng et al. (2016)	+	+	?	+	+	+	?	?	?	+
Maeda et al. (2007)	+	+	?	+	?	+	?	?	?	?
Zheng et al. (2019)	+	+	?	+	+	+	?	?	?	+
Qi and Zhang (2014)	+	+	?	+	+	+	?	?	?	+
Tang et al. (2014)	+	+	?	+	+	-	?	?	?	+
van der Pauw et al. (2000)	+	+	?	+	+	-	?	?	?	+
Zheng et al. (2012)	+	+	?	+	+	-	?	?	?	+
Summary										
Low risk of bias "+"	7 (100%)	7 (100%	0 (0%)	7 (100%)	6 (86%)	4 (57%)	0 (0%)	0 (0%)	0 (0%)	6 (86%)
Incomplete/unclear risk of bias "?"	0 (0%)	0 (0%)	7 (100%)	0 (0%)	1 (14%)	0 (0%)	7 (100%)	7 (100%)	7 (100%)	1 (14%)
High risk of bias "-"	0 (0%)	0 (0%)	0 (0%)	0 (0%)	0 (0%)	3 (43%)	0 (0%)	0 (0%)	0 (0%)	0 (0%)
Not applicable "n. a."	0 (0%)	0 (0%)	0 (0%)	0 (0%)	0 (0%)	0 (0%)	0 (0%)	0 (0%)	0 (0%)	0 (0%)
Sum	7	7	7	7	7	7	7	7	7	7

3.7 Human periodontal ligament cells – Methodological risk of bias

Reference	Selection bias			Perfor-mance bias	Detection bias	Attrition bias	Reporting bias	Confounding bias	Appropriate statistical methods		Appropriate/ controlled exposure (incl. characterization)	Optimal time window used	Statement conflict of interest/funding source	Test substance/treatment details	Test organism/system
	Baseline characteristics similarity / appropriate control group selection	Allocation concealment	Randomization	Blinding of researchers	Blinding of outcome assessors	Complete outcome data	Selective outcome data	Account for confounding variables	Sample size determination	Statistical analysis					
Zheng et al. (2016)	?	?	n.a.	n.a.	n.a.	+	+	?	-	-	?	?	+	?	?
Maeda et al. (2007)	?	?	n.g.	n.a.	n.a.	?	?	?	-	-	?	+	?	?	?
Zheng et al. (2019)	?	?	n.a.	n.a.	n.a.	+	+	?	-	-	?	?	+	+	?
Qi and Zhang (2014)	?	?	n.a.	n.a.	n.a.	+	+	?	-	-	?	?	?	?	?
Tang et al. (2014)	+	?	n.a.	n.a.	n.a.	+	+	?	-	-	?	?	?	?	?
van der Pauw et al. (2000)	?	?	n.a.	n.a.	n.a.	+	+	?	-	-	?	?	-	?	?
Zheng et al. (2012)	?	?	n.a.	n.a.	n.a.	+	+	?	-	-	?	?	+	?	?
Summary															
Low risk of bias "+"	1 (14%)	0 (0%)	0 (0%)	0 (0%)	0 (0%)	6 (86%)	6 (86%)	0 (0%)	0 (0%)	0 (0%)	0 (0%)	1 (14%)	3 (43%)	1 (14%)	0 (0%)
Incomplete/unclear risk of bias "?"	6 (86%)	7 (100%)	0 (0%)	0 (0%)	0 (0%)	1 (14%)	1 (14%)	7 (100%)	0 (0%)	0 (0%)	7 (100%)	6 (86%)	3 (43%)	6 (86%)	7 (100%)
High risk of bias "-"	0 (0%)	0 (0%)	0 (0%)	0 (0%)	0 (0%)	0 (0%)	0 (0%)	0 (0%)	7 (100%)	7 (100%)	0 (0%)	0 (0%)	1 (14%)	0 (0%)	0 (0%)
Not applicable "n. a."	0 (0%)	0 (0%)	7 (100%)	7 (100%)	7 (100%)	0 (0%)	0 (0%)	0 (0%)	0 (0%)	0 (0%)	0 (0%)	0 (0%)	0 (0%)	0 (0%)	0 (0%)
Sum	7	7	7	7	7	7	7	7	7	7	7	7	7	7	7

References

Maeda A, Soejima K, Bandow K, Kuroe K, Kakimoto K, Miyawaki S, Okamoto A, Matsuguchi T (2007). Force-induced IL-8 from periodontal ligament cells requires IL-1beta. J Dent Res; 86(7):629-34.

Qi L, Zhang Y (2014). The microRNA 132 regulates fluid shear stress-induced differentiation in periodontal ligament cells through mTOR signaling pathway. Cell Physiol Biochem; 33(2):433-45.

Tang M, Peng Z, Mai Z, Chen L, Mao Q, Chen Z, Chen Q, Liu L, Wang Y, Ai H (2014). Fluid shear stress stimulates osteogenic differentiation of human periodontal ligament cells via the extracellular signal-regulated kinase 1/2 and p38 mitogen-activated protein kinase signaling pathways. J Periodontol; 85(12):1806-13.

van der Pauw MT, Klein-Nulend J, van den Bos T, Burger EH, Everts V, Beertsen W (2000). Response of periodontal ligament fibroblasts and gingival fibroblasts to pulsating fluid flow: nitric oxide and prostaglandin E_2 release and expression of tissue non-specific alkaline phosphatase activity. J Periodontal Res; 35(6):335-43.

Zheng L, Huang Y, Song W, Gong X, Liu M, Jia X, Zhou G, Chen L, Li A, Fan Y (2012). Fluid shear stress regulates metalloproteinase-1 and 2 in human periodontal ligament cells: involvement of extracellular signal-regulated kinase (ERK) and P38 signaling pathways. J Biomech; 45(14):2368-75.

Zheng L, Chen L, Chen Y, Gui J, Li Q, Huang Y, Liu M, Jia X, Song W, Ji J, Gong X, Shi R, Fan Y (2016). The effects of fluid shear stress on proliferation and osteogenesis of human periodontal ligament cells. J Biomech; 49(4):572-9.

Zheng L, Shi Q, Na J, Liu N, Guo Y, Fan Y (2019). Platelet-Derived Growth Factor Receptor-α and β are Involved in Fluid Shear Stress Regulated Cell Migration in Human Periodontal Ligament Cells. Cell Mol Bioeng; 12(1):85-97.

Mouse osteoblasts

3.8 Mouse osteoblasts – Reporting risk of bias

Reference	Description of scientific background	Description objective	Justification for model	Study design description	Defined experimental outcomes	Ethical statement	Cell maintenance condition	Description of measurement precision and variability	Statistical analysis	Results description
Yang et al. (2015)	+	+	?	+	+	+	?	?	?	+
Yang et al. (2010)	+	+	?	+	?	-	?	?	?	?
Fu et al. (2008)	+	+	?	+	+	-	?	?	?	+
Li et al. (2005)	+	+	?	?	+	-	?	?	?	+
Bakker et al. (2003a)	+	+	?	+	?	-	?	?	?	?
Rangaswami et al. (2012)	+	+	?	+	?	-	?	?	?	?
Mehrotra et al. (2006)	+	+	?	+	?	-	?	?	?	?
Bakker et al. (2003b)	+	+	?	+	?	+	?	?	?	?
Kapur et al. (2010)	+	+	?	+	?	-	?	?	?	?
Xing et al. (2014)	+	+	?	?	+	+	?	?	?	+
Castillo et al. (2014)	+	+	?	+	?	-	?	?	?	?
Kido et al. (2009)	+	?	?	+	?	-	?	?	?	?
Callewaert et al. (2010)	+	+	?	?	?	+	?	?	?	?
Igwe et al. (2009)	+	+	?	+	?	-	?	?	?	?
Lau et al. (2006)	+	+	?	+	+	-	?	?	?	+
Suzuki et al. (2013)	+	+	?	+	+	-	?	?	?	+
Bakker et al. (2013)	+	+	?	+	?	-	?	?	?	?
Thi et al. (2012)	+	+	?	+	+	-	?	?	?	+
Bakker et al. (2001)	+	+	?	+	?	-	?	?	?	?
Soejima et al. (2001)	?	-	?	+	?	-	?	?	?	?
Klein-Nulend et al. (1997)	+	+	?	+	?	-	?	?	?	?
Klein-Nulend et al. (1996)	+	+	?	+	?	-	?	?	?	?
Summary										
Low risk of bias "+"	21 (95%)	20 (91%)	0 (0%)	19 (86%)	7 (32%)	4 (18%)	0 (0%)	0 (0%)	0 (0%)	7 (32%)
Incomplete/unclear risk of bias "?"	1 (5%)	1 (5%)	22 (100%)	3 (14%)	15 (68%)	0 (0%)	22 (100%)	22 (100%)	22 (100%)	15 (68%)
High risk of bias "-"	0 (0%)	1 (5%)	0 (0%)	0 (0%)	0 (0%)	18 (82%)	0 (0%)	0 (0%)	0 (0%)	0 (0%)
Not applicable "n. a."	0 (0%)	0 (0%)	0 (0%)	0 (0%)	0 (0%)	0 (0%)	0 (0%)	0 (0%)	0 (0%)	0 (0%)
Sum	22	22	22	22	22	22	22	22	22	22

3.9 Mouse osteoblasts – Methodological risk of bias

Reference	Selection bias: Baseline characteristics similarity / appropriate control [illegible]	Selection bias: Allocation concealment	Selection bias: Randomization	Performance bias: Blinding of researchers	Detection bias: Blinding of outcome assessors	Attrition bias: Complete outcome data	Reporting bias: Selective outcome data	Confounding bias: Account for confounding variables	Appropriate statistical methods: Sample size determination	Appropriate statistical methods: Statistical analysis	Appropriate/ controlled exposure (incl. characterization)	Optimal time window used	Statement conflict of interest/funding source	Test substance/treatment details	Test organism/system
Yang et al. (2015)	?	?	n.a.	n.a.	n.a.	+	+	?	-	-	?	?	+	?	?
Yang et al. (2010)	?	?	n.a.	n.a.	n.a.	?	-	?	-	-	?	?	?	?	?
Fu et al. (2008)	?	?	n.a.	n.a.	n.a.	+	+	?	-	-	?	?	?	?	?
Li et al. (2005)	?	?	n.a.	n.a.	n.a.	+	+	?	-	-	?	?	-	?	?
Bakker et al. (2003a)	?	?	n.a.	n.a.	n.a.	?	-	?	-	-	?	?	?	?	?
Rangaswami et al. (2012)	?	?	n.a.	n.a.	n.a.	?	-	?	-	-	?	?	-	?	?
Mehrotra et al. (2006)	-	?	n.a.	n.a.	n.a.	?	-	?	-	-	?	?	?	?	?
Bakker et al. (2003b)	?	?	n.a.	n.a.	n.a.	?	-	?	-	-	?	?	?	?	?
Kapur et al. (2010)	?	?	n.a.	n.a.	n.a.	?	-	?	-	-	?	?	-	?	?
Xing et al. (2014)	-	?	n.a.	n.a.	n.a.	+	+	?	-	-	?	?	-	?	?
Castillo et al. (2014)	?	?	n.a.	n.a.	n.a.	?	-	?	-	-	?	?	-	?	?
Kido et al. (2009)	?	?	n.a.	n.a.	n.a.	?	-	?	-	-	?	?	+	?	?
Callewaert et al. (2010)	?	?	n.a.	n.a.	n.a.	?	-	?	-	?	?	?	+	?	?
Igwe et al. (2009)	?	?	n.a.	n.a.	n.a.	?	-	?	-	-	?	?	?	?	?
Lau et al. (2006)	?	?	n.a.	n.a.	n.a.	+	+	?	-	-	?	?	-	?	?
Suzuki et al. (2013)	?	?	n.a.	n.a.	n.a.	+	+	?	-	-	?	?	?	?	?
Bakker et al. (2013)	?	?	n.a.	n.a.	n.a.	?	-	?	-	-	?	?	?	?	?
Thi et al. (2012)	?	?	n.a.	n.a.	n.a.	+	+	?	-	-	?	?	?	?	?
Bakker et al. (2001)	?	?	n.a.	n.a.	n.a.	?	-	?	-	-	?	?	?	?	?
Soejima et al. (2001)	?	?	n.a.	n.a.	n.a.	?	-	?	-	-	?	?	-	?	?
Klein-Nulend et al. (1997)	?	?	n.a.	n.a.	n.a.	?	-	?	-	-	?	?	?	?	?
Klein-Nulend et al. (1996)	?	?	n.a.	n.a.	n.a.	?	-	?	-	-	?	?	?	?	?
Summary															
Low risk of bias "+"	0 (0%)	0 (0%)	0 (0%)	0 (0%)	0 (0%)	7 (32%)	7 (32%)	0 (0%)	0 (0%)	0 (0%)	0 (0%)	0 (0%)	3 (14%)	0 (0%)	0 (0%)
Incomplete/unclear risk of bias "?"	20 (91%)	22 (100%)	0 (0%)	0 (0%)	0 (0%)	15 (68%)	0 (0%)	22 (100%)	0 (0%)	1 (5%)	22 (100%)	22 (100%)	12 (55%)	22 (100%)	22 (100%)

	Selection bias			Perfor-mance bias	Detection bias	Attrition bias	Reporting bias	Confounding bias	Appropriate statistical methods						
Reference	Baseline characteristics similarity / appropriate control	Allocation concealment	Randomization	Blinding of researchers	Blinding of outcome assessors	Complete outcome data	Selective outcome data	Account for confounding variables	Sample size determination	Statistical analysis	Appropriate/ controlled exposure (incl. characterization)	Optimal time window used	Statement conflict of interest/funding source	Test substance/treatment details	Test organism/system
High risk of bias "-"	2 (9%)	0 (0%)	0 (0%)	0 (0%)	0 (0%)	0 (0%)	15 (68%)	0 (0%)	22 (100%)	21 (95%)	0 (0%)	0 (0%)	7 (32%)	0 (0%)	0 (0%)
Not applicable "n. a."	0 (0%)	0 (0%)	22 (100 %)	22 (100%)	22 (100%)	0 (0%)	0 (0%)	0 (0%)	0 (0%)	0 (0%)	0 (0%)	0 (0%)	0 (0%)	0 (0%)	0 (0%)
Sum	22	22	22	22	22	22	22	22	22	22	22	22	22	22	22

References

Bakker AD, Soejima K, Klein-Nulend J, Burger EH (2001). The production of nitric oxide and prostaglandin E(2) by primary bone cells is shear stress dependent. J Biomech; 34(5):671-7.

Bakker AD, Joldersma M, Klein-Nulend J, Burger EH (2003a). Interactive effects of PTH and mechanical stress on nitric oxide and PGE2 production by primary mouse osteoblastic cells. Am J Physiol Endocrinol Metab; 285(3):E608-13.

Bakker AD, Klein-Nulend J, Burger EH (2003b). Mechanotransduction in bone cells proceeds via activation of COX-2, but not COX-1. Biochem Biophys Res Commun; 305(3):677-83.

Bakker AD, Huesa C, Hughes A, Aspden RM, van't Hof RJ, Klein-Nulend J, Helfrich MH (2013). Endothelial nitric oxide synthase is not essential for nitric oxide production by osteoblasts subjected to fluid shear stress in vitro. Calcif Tissue Int; 92(3):228-39.

Callewaert F, Bakker A, Schrooten J, Van Meerbeek B, Verhoeven G, Boonen S, Vanderschueren D (2010). Androgen receptor disruption increases the osteogenic response to mechanical loading in male mice. J Bone Miner Res; 25(1):124-31.

Castillo AB, Triplett JW, Pavalko FM, Turner CH (2014). Estrogen receptor-β regulates mechanical signaling in primary osteoblasts. Am J Physiol Endocrinol Metab; 306(8):E937-44.

Fu Q, Wu C, Shen Y, Zheng S, Chen R (2008). Effect of LIMK2 RNAi on reorganization of the actin cytoskeleton in osteoblasts induced by fluid shear stress. J Biomech; 41(15):3225-8.

Igwe JC, Jiang X, Paic F, Ma L, Adams DJ, Baldock PA, Pilbeam CC, Kalajzic I (2009). Neuropeptide Y is expressed by osteocytes and can inhibit osteoblastic activity. J Cell Biochem; 108(3):621-30.

Kapur S, Amoui M, Kesavan C, Wang X, Mohan S, Baylink DJ, Lau KH (2010). Leptin receptor (Lepr) is a negative modulator of bone mechanosensitivity and genetic variations in Lepr may contribute to the differential osteogenic response to mechanical stimulation in the C57BL/6J and C3H/HeJ pair of mouse strains. J Biol Chem; 285(48):37607-18.

Kido S, Kuriwaka-Kido R, Imamura T, Ito Y, Inoue D, Matsumoto T (2009). Mechanical stress induces Interleukin-11 expression to stimulate osteoblast differentiation. Bone; 45(6):1125-32.

Klein-Nulend J, Semeins CM, Burger EH (1996). Prostaglandin mediated modulation of transforming growth factor-beta metabolism in primary mouse osteoblastic cells in vitro. J Cell Physiol; 168(1):1-7.

Klein-Nulend J, Burger EH, Semeins CM, Raisz LG, Pilbeam CC (1997). Pulsating fluid flow stimulates prostaglandin release and inducible prostaglandin G/H synthase mRNA expression in primary mouse bone cells. J Bone Miner Res; 12(1):45-51.

Lau KH, Kapur S, Kesavan C, Baylink DJ (2006). Up-regulation of the Wnt, estrogen receptor, insulin-like growth factor-I, and bone morphogenetic protein pathways in C57BL/6J osteoblasts as opposed to C3H/HeJ osteoblasts in part contributes to the differential anabolic response to fluid shear. J Biol Chem; 281(14):9576-88.

Li J, Liu D, Ke HZ, Duncan RL, Turner CH (2005). The P2X7 nucleotide receptor mediates skeletal mechanotransduction. J Biol Chem; 280(52):42952-9.

Mehrotra M, Saegusa M, Wadhwa S, Voznesensky O, Peterson D, Pilbeam C (2006). Fluid flow induces Rankl expression in primary murine calvarial osteoblasts. J Cell Biochem; 98(5):1271-83.

Rangaswami H, Schwappacher R, Tran T, Chan GC, Zhuang S, Boss GR, Pilz RB (2012). Protein kinase G and focal adhesion kinase converge on Src/Akt/β-catenin signaling module in osteoblast mechanotransduction. J Biol Chem; 287(25):21509-19.

Soejima K, Klein-Nulend J, Semeins CM, Burger EH (2001). Different responsiveness of cells from adult and neonatal mouse bone to mechanical and biochemical challenge. J Cell Physiol; 186(3):366-70.

Suzuki T, Notomi T, Miyajima D, Mizoguchi F, Hayata T, Nakamoto T, Hanyu R, Kamolratanakul P, Mizuno A, Suzuki M, Ezura Y, Izumi Y, Noda M (2013). Osteoblastic differentiation enhances expression of TRPV4 that is required for calcium oscillation induced by mechanical force. Bone; 54(1):172-8.

Thi MM, Islam S, Suadicani SO, Spray DC (2012). Connexin43 and pannexin1 channels in osteoblasts: who is the "hemichannel"? J Membr Biol; 245(7):401-9.

Xing Y, Gu Y, Bresnahan JJ, Paul EM, Donahue HJ, You J (2014). The roles of P2Y2 purinergic receptors in osteoblasts and mechanotransduction. PLoS One; 9(9):e108417.

Yang Z, Bidwell JP, Young SR, Gerard-O'Riley R, Wang H, Pavalko FM (2010). Nmp4/CIZ inhibits mechanically induced beta-catenin signaling activity in osteoblasts. J Cell Physiol; 223(2):435-41.

Yang Z, Tan S, Shen Y, Chen R, Wu C, Xu Y, Song Z, Fu Q (2015). Inhibition of FSS-induced actin cytoskeleton reorganization by silencing LIMK2 gene increases the mechanosensitivity of primary osteoblasts. Bone; 74:182-90.

Mouse osteocytes

3.10 Mouse osteocytes – Reporting risk of bias

Reference	Description of scientific background	Description objective	Justification for model	Study design description	Defined experimental outcomes	Ethical statement	Cell maintenance condition	Description of measurement precision and variability	Statistical analysis	Results description
Wang et al. (2019)	+	+	?	+	+	-	-	?	?	+
Liao et al. (2017)	+	+	+	+	+	-	?	?	?	+
Deepak et al. (2017)	+	+	?	+	+	-	?	?	?	+
Du et al. (2020)	+	+	?	+	+	+	?	?	?	+
Yan et al. (2018)	+	+	?	?	+	-	?	+	+	+
Geoghegan et al. (2019)	+	+	+	+	+	-	?	?	?	+
Lu et al. (2012b)	+	+	?	+	+	-	?	?	?	?
Zhang et al. (2015)	+	?	?	+	+	-	?	?	?	+
Govey et al. (2015)	+	-	?	?	+	-	?	-	-	+
Kalogeropoulos et al. (2010)	+	-	?	+	?	+	?	?	?	?
Bakker et al. (2014)	+	+	+	+	?	-	?	?	?	?
Thi et al. (2010)	+	-	?	+	+	-	?	?	?	+
Maycas et al. (2017)	+	+	?	+	+	-	?	?	?	+
Shah et al. (2017)	+	+	?	?	?	-	?	+	+	+
Thi et al. (2003)	+	+	?	+	?	-	?	?	?	+
Reilly et al. (2003)	+	+	?	+	+	-	?	+	+	+
Lu et al. (2012a)	+	+	?	+	?	-	?	?	?	+
Xu et al. (2012)	+	+	?	+	+	-	?	?	?	+
Jing et al. (2013)	+	+	?	?	+	-	?	?	?	+
Govey et al. (2014)	+	+	+	+	+	-	?	?	?	+
Kulkarni et al. (2012b)	+	+	?	+	?	-	?	+	+	?
Genetos et al. (2007)	+	+	+	+	?	-	?	?	?	?
Litzenberger et al. (2010)	+	+	?	+	?	-	?	?	?	?
Li et al. (2012)	+	+	+	+	+	-	?	?	?	+
Kamel et al. (2010)	+	+	?	+	?	-	?	?	?	?
Rath et al. (2010)	+	+	?	?	+	-	?	?	?	+
Zhang et al. (2006)	+	+	?	+	?	-	?	?	?	?
Bakker et al. (2009)	+	+	?	+	+	-	?	?	?	+
Li et al. (2013)	+	+	+	+	+	-	?	?	?	+
Middleton et al. (2018)	+	+	?	+	+	-	?	?	?	+
Cheng et al. (2001)	+	+	?	+	?	-	?	?	?	?

Reference	Description of scientific background	Description objective	Justification for model	Study design description	Defined experimental outcomes	Ethical statement	Cell maintenance condition	Description of measurement precision and variability	Statistical analysis	Results description
Maycas et al. (2015)	+	+	+	+	?	-	-	?	?	?
Haugh et al. (2015)	+	+	?	+	+	-	?	?	?	+
Santos et al. (2009)	+	+	?	+	?	-	?	?	?	+
Xu et al. (2014)	+	+	?	+	+	-	?	?	?	+
Fahlgren et al. (2018)	+	+	+	+	+	?	?	?	?	+
Chen et al. (2015)	+	+	+	+	+	-	?	+	+	+
Kitase et al. (2014)	+	+	?	-	+	-	-	?	?	+
Ren et al. (2013)	+	+	?	+	+	-	?	?	?	+
González et al. (2017)	+	+	+	+	+	-	?	?	?	+
Bakker et al. (2013)	+	+	?	+	?	-	?	+	+	?
Kulkarni et al. (2010)	+	+	+	+	?	-	?	?	?	?
Seref-Ferlengez et al. (2016)	+	+	?	+	?	-	?	?	?	?
Cherian et al. (2005)	+	-	?	+	+	-	?	?	?	+
Riquelme et al. (2021)	+	+	+	+	+	-	?	?	?	+
Liu et al. (2015)	+	+	?	+	?	-	?	?	?	?
Cherian et al. (2003)	+	-	?	+	?	-	?	?	?	?
Batra et al. (2014)	+	?	?	+	+	-	?	?	?	+
de Castro et al. (2015)	+	+	?	+	+	-	?	?	?	+
Santos et al. (2010)	+	+	?	+	?	-	?	?	?	?
Xia et al. (2010)	+	+	?	+	?	-	?	?	?	?
Huang et al. (2017)	+	+	?	?	+	-	?	+	+	+
Kulkarni et al. (2012a)	+	+	?	+	?	-	?	?	?	?
Juffer et al. (2012)	+	+	?	+	?	-	?	?	?	?
Kitase et al. (2010)	+	+	?	?	?	-	?	?	?	?
Alford et al. (2003)	+	-	?	+	?	-	?	?	?	?
Li et al. (2019)	+	?	+	?	+	+	?	+	+	+
Summary										
Low risk of bias "+"	57 (100%)	48 (84%)	14 (25%)	48 (84%)	32 (56%)	3 (5%)	0 (0%)	8 (14%)	8 (14%)	36 (63%)
Incomplete/unclear risk of bias "?"	0 (0%)	3 (5%)	43 (75%)	8 (14%)	25 (44%)	1 (2%)	54 (95%)	48 (84%)	48 (84%)	21 (37%)
High risk of bias "-"	0 (0%)	6 (11%)	0 (0%)	1 (2%)	0 (0%)	53 (93%)	3 (5%)	1 (2%)	1 (2%)	0 (0%)
Not applicable "n. a."	0 (0%)	0 (0%)	0 (0%)	0 (0%)	0 (0%)	0 (0%)	0 (0%)	0 (0%)	0 (0%)	0 (0%)
Sum	57	57	57	57	57	57	57	57	57	57

3.11 Mouse osteocytes – Methodological risk of bias

Reference	Selection bias			Perfor-mance bias	Detection bias	Attrition bias	Reporting bias	Confounding bias	Appropriate statistical methods		Appropriate/ controlled exposure (incl. characterization)	Optimal time window used	Statement conflict of interest/funding source	Test substance/treatment details	Test organism/system
	Baseline characteristics similarity / appropriate control group selection	Allocation concealment	Randomization	Blinding of researchers	Blinding of outcome assessors	Complete outcome data	Selective outcome data	Account for confounding variables	Sample size determination	Statistical analysis					
Wang et al. (2019)	?	?	n.a.	n.a.	n.a.	+	+	?	-	-	?	?	+	?	-
Liao et al. (2017)	?	?	n.a.	n.a.	n.a.	+	+	?	-	-	?	?	?	?	?
Deepak et al. (2017)	?	?	n.a	n.a.	n.a.	+	+	?	-	-	?	?	+	?	?
Du et al. (2020)	?	?	n.a.	n.a.	n.a.	+	+	?	-	-	?	?	?	?	?
Yan et al. (2018)	?	?	n.a.	n.a.	n.a.	+	+	?	-	?	?	?	+	?	?
Geoghegan et al. (2019)	?	?	n.a.	n.a.	n.a.	+	+	?	-	-	?	?	+	?	?
Lu et al. (2012b)	?	?	n.a.	n.a.	n.a.	+	?	?	-	-	?	?	?	?	?
Zhang et al. (2015)	?	?	n.a.	n.a.	n.a.	+	+	?	-	-	?	?	?	?	?
Govey et al. (2015)	?	?	n.a.	n.a.	n.a.	+	+	?	-	-	?	?	+	?	?
Kalogeropoulos et al. (2010)	-	?	n.a.	n.a.	n.a.	?	-	?	-	-	?	?	?	?	?
Bakker et al. (2014)	?	?	n.a.	n.a.	n.a.	?	-	?	-	?	?	?	-	?	?
Thi et al. (2010)	?	?	n.a.	n.a.	n.a.	+	+	?	-	-	?	?	-	?	?
Maycas et al. (2017)	?	?	n.a.	n.a.	n.a.	+	+	?	-	-	?	?	?	?	?
Shah et al. (2017)	?	?	n.a.	n.a.	n.a.	?	-	?	+	?	?	?	?	?	?
Thi et al. (2003)	?	?	n.a.	n.a.	n.a.	?	-	?	-	-	?	?	?	?	?
Reilly et al. (2003)	?	?	n.a.	n.a.	n.a.	+	+	?	-	-	?	?	?	?	?
Lu et al. (2012a)	?	?	n.a.	n.a.	n.a.	?	-	?	-	-	?	?	+	?	?
Xu et al. (2012)	?	?	n.a.	n.a.	n.a.	+	+	?	-	-	?	?	?	?	?
Jing et al. (2013)	?	?	n.a.	n.a.	n.a.	+	+	?	-	-	?	?	?	?	?
Govey et al. (2014)	?	?	n.a.	n.a.	n.a.	+	+	?	-	-	?	?	+	?	?
Kulkarni et al. (2012b)	?	?	n.a.	n.a.	n.a.	?	-	?	-	?	?	?	?	?	?
Genetos et al. (2007)	?	?	n.a.	n.a.	n.a.	?	-	?	-	-	?	?	?	?	?
Litzenberger et al. (2010)	?	?	n.a.	n.a.	n.a.	?	-	?	-	-	?	?	?	?	?
Li et al. (2012)	?	?	n.a.	n.a.	n.a.	+	+	?	-	-	?	?	+	?	?
Kamel et al. (2010)	?	?	n.a.	n.a.	n.a.	?	-	?	-	-	?	?	?	?	?
Rath et al. (2010)	?	?	n.a.	n.a.	n.a.	+	+	?	-	-	?	?	+	?	?
Zhang et al. (2006)	?	?	n.a.	n.a.	n.a.	?	-	?	-	-	?	?	?	?	?
Bakker et al. (2009)	?	?	n.a.	n.a.	n.a.	+	+	?	-	-	?	?	-	?	?

Reference	Selection bias			Performance bias	Detection bias	Attrition bias	Reporting bias	Confounding bias	Appropriate statistical methods						
	Baseline characteristics similarity / appropriate control group selection	Allocation concealment	Randomization	Blinding of researchers	Blinding of outcome assessors	Complete outcome data	Selective outcome data	Account for confounding variables	Sample size determination	Statistical analysis	Appropriate/ controlled exposure (incl. characterization)	Optimal time window used	Statement conflict of interest/funding source	Test substance/treatment details	Test organism/system
Li et al. (2013)	?	?	n.a.	n.a.	n.a.	+	+	?	-	-	?	?	?	?	?
Middleton et al. (2018)	?	?	n.a.	n.a.	n.a.	+	+	?	-	-	?	?	?	?	?
Cheng et al. (2001)	?	?	n.a.	n.a.	n.a.	?	-	?	-	-	?	?	?	?	?
Maycas et al. (2015)	-	?	n.a.	n.a.	n.a.	?	-	?	-	-	-	?	+	?	-
Haugh et al. (2015)	?	?	n.a.	n.a.	n.a.	+	+	?	-	-	?	?	?	?	?
Santos et al. (2009)	?	?	n.a.	n.a.	n.a.	?	-	?	-	-	?	?	?	?	?
Xu et al. (2014)	?	?	n.a.	n.a.	n.a.	+	+	?	-	-	?	?	+	?	?
Fahlgren et al. (2018)	?	?	n.a.	n.a.	n.a.	+	+	?	-	-	?	?	?	?	?
Chen et al. (2015)	?	?	n.a.	n.a.	n.a.	+	+	?	-	?	?	?	?	?	?
Kitase et al. (2014)	?	?	n.a.	n.a.	n.a.	+	+	?	-	-	?	?	+	?	-
Ren et al. (2013)	?	?	n.a.	n.a.	n.a.	+	+	?	-	-	?	?	?	?	?
González et al. (2017)	?	?	n.a.	n.a.	n.a.	+	+	?	-	-	?	?	-	?	?
Bakker et al. (2013)	?	?	n.a.	n.a.	n.a.	?	-	?	-	-	?	?	?	?	?
Kulkarni et al. (2010)	?	?	n.a.	n.a.	n.a.	?	-	?	-	-	?	?	?	?	?
Seref-Ferlengez et al. (2016)	?	?	n.a.	n.a.	n.a.	?	-	?	-	-	?	?	-	?	?
Cherian et al. (2005)	?	?	n.a.	n.a.	n.a.	+	+	?	-	-	?	?	?	?	?
Riquelme et al. (2021)	?	?	n.a.	n.a.	n.a.	+	+	?	-	-	?	?	+	?	?
Liu et al. (2015)	?	?	n.a.	n.a.	n.a.	?	-	?	-	-	?	?	+	?	?
Cherian et al. (2003)	?	?	n.a.	n.a.	n.a.	?	-	?	-	-	?	?	-	?	?
Batra et al. (2014)	?	?	n.a.	n.a.	n.a.	+	+	?	-	-	?	?	-	?	?
de Castro et al. (2015)	?	?	n.a.	n.a.	n.a.	+	+	?	-	-	?	?	?	+	?
Santos et al. (2010)	?	?	n.a.	n.a.	n.a.	?	-	?	-	-	?	?	?	?	?
Xia et al. (2010)	?	?	n.a.	n.a.	n.a.	?	-	?	-	-	?	?	?	?	?
Huang et al. (2017)	?	?	n.a.	n.a.	n.a.	+	+	?	-	-	?	-	+	+	?
Kulkarni et al. (2012a)	?	?	n.a.	n.a.	n.a.	?	-	?	-	-	?	?	?	?	?
Juffer et al. (2012)	?	?	n.a.	n.a.	n.a.	?	-	?	-	-	?	?	+	?	?
Kitase et al. (2010)	?	?	n.a.	n.a.	n.a.	?	-	?	-	-	?	?	+	?	?
Alford et al. (2003)	?	?	n.a.	n.a.	n.a.	?	-	?	-	-	?	?	-	?	?
Li et al. (2019)	?	?	n.a.	n.a.	n.a.	+	?	?	-	?	?	?	+	?	?
Summary															

Reference	Selection bias			Performance bias	Detection bias	Attrition bias	Reporting bias	Confounding bias	Appropriate statistical methods		Appropriate/ controlled exposure (incl. characterization)	Optimal time window used	Statement conflict of interest/funding source	Test substance/treatment details	Test organism/system
	Baseline characteristics similarity / appropriate control group selection	Allocation concealment	Randomization	Blinding of researchers	Blinding of outcome assessors	Complete outcome data	Selective outcome data	Account for confounding variables	Sample size determination	Statistical analysis					
Low risk of bias "+"	0 (0%)	0 (0%)	0 (0%)	0 (0%)	0 (0%)	33 (58%)	31 (54%)	0 (0%)	1 (2%)	0 (0%)	0 (0%)	0 (0%)	18 (32%)	2 (4%)	0 (0%)
Incomplete/unclear risk of bias "?"	55 (96%)	57 (100%)	0 (0%)	0 (0%)	0 (0%)	24 (42%)	2 (4%)	57 (100%)	0 (0%)	6 (11%)	56 (98%)	56 (98%)	31 (54%)	55 (96%)	54 (95%)
High risk of bias "-"	2 (4%)	0 (0%)	0 (0%)	0 (0%)	0 (0%)	0 (0%)	24 (42%)	0 (0%)	56 (98%)	51 (89%)	1 (2%)	1 (2%)	8 (14%)	0 (0%)	3 (5%)
Not applicable "n. a."	0 (0%)	0 (0%)	57 (100%)	57 (100%)	57 (100%)	0 (0%)	0 (0%)	0 (0%)	0 (0%)	0 (0%)	0 (0%)	0 (0%)	0 (0%)	0 (0%)	0 (0%)
Sum	57	57	57	57	57	57	57	57	57	57	57	57	57	57	57

References

Alford AI, Jacobs CR, Donahue HJ (2003). Oscillating fluid flow regulates gap junction communication in osteocytic MLO-Y4 cells by an ERK1/2 MAP kinase-dependent mechanism. Bone; 33(1):64-70.

Bakker AD, Silva VC, Krishnan R, Bacabac RG, Blaauboer ME, Lin YC, Marcantonio RA, Cirelli JA, Klein-Nulend J (2009). Tumor necrosis factor alpha and interleukin-1beta modulate calcium and nitric oxide signaling in mechanically stimulated osteocytes. Arthritis Rheum; 60(11):3336-45.

Bakker AD, Zandieh-Doulabi B, Klein-Nulend J (2013). Strontium ranelate affects signaling from mechanically-stimulated osteocytes towards osteoclasts and osteoblasts. Bone; 53(1):112-9.

Bakker AD, Kulkarni RN, Klein-Nulend J, Lems WF (2014). IL-6 alters osteocyte signaling toward osteoblasts but not osteoclasts. J Dent Res; 93(4):394-9.

Batra N, Riquelme MA, Burra S, Kar R, Gu S, Jiang JX (2014). Direct regulation of osteocytic connexin 43 hemichannels through AKT kinase activated by mechanical stimulation. J Biol Chem; 289(15):10582-91.

Chen JC, Chua M, Bellon RB, Jacobs CR (2015). Epigenetic changes during mechanically induced osteogenic lineage commitment. J Biomech Eng; 137(2):020902.

Cheng B, Zhao S, Luo J, Sprague E, Bonewald LF, Jiang JX (2001). Expression of functional gap junctions and regulation by fluid flow in osteocyte-like MLO-Y4 cells. J Bone Miner Res; 16(2):249-59.

Cherian PP, Cheng B, Gu S, Sprague E, Bonewald LF, Jiang JX (2003). Effects of mechanical strain on the function of Gap junctions in osteocytes are mediated through the prostaglandin EP2 receptor. J Biol Chem; 278(44):43146-56.

Cherian PP, Siller-Jackson AJ, Gu S, Wang X, Bonewald LF, Sprague E, Jiang JX (2005). Mechanical strain opens connexin 43 hemichannels in osteocytes: a novel mechanism for the release of prostaglandin. Mol Biol Cell; 16(7):3100-6.

de Castro LF, Maycas M, Bravo B, Esbrit P, Gortazar A (2015). VEGF Receptor 2 (VEGFR2) Activation Is Essential for Osteocyte Survival Induced by Mechanotransduction. J Cell Physiol; 230(2):278-85.

Deepak V, Kayastha P, McNamara LM (2017). Estrogen deficiency attenuates fluid flow-induced [Ca(2+)](i) oscillations and mechanoresponsiveness of MLO-Y4 osteocytes. FASEB J; 31(7):3027-3039.

Du J, Yang J, He Z, Cui J, Yang Y, Xu M, Qu X, Zhao N, Yan M, Li H, Yu Z (2020). Osteoblast and Osteoclast Activity Affect Bone Remodeling Upon Regulation by Mechanical Loading-Induced Leukemia Inhibitory Factor Expression in Osteocytes. Frontiers in Molecular Biosciences; 7:585056.

Fahlgren A, Bratengeier C, Semeins CM, Klein-Nulend J, Bakker AD (2018). Supraphysiological loading induces osteocyte-mediated osteoclastogenesis in a novel in vitro model for bone implant loosening. J Orthop Res; 36(5):1425-1434.

Genetos DC, Kephart CJ, Zhang Y, Yellowley CE, Donahue HJ (2007). Oscillating fluid flow activation of gap junction hemichannels induces ATP release from MLO-Y4 osteocytes. J Cell Physiol; 212(1):207-14.

Geoghegan IP, Hoey DA, McNamara LM (2019). Estrogen deficiency impairs integrin α(v)β(3)-mediated mechanosensation by osteocytes and alters osteoclastogenic paracrine signalling. Sci Rep; 9(1):4654.

González Á, García de Durango C, Alonso V, Bravo B, Rodríguez de Gortázar A, Wells A, Forteza J, Vidal-Vanaclocha F (2017). Distinct Osteomimetic Response of Androgen-Dependent and

Independent Human Prostate Cancer Cells to Mechanical Action of Fluid Flow: Prometastatic Implications. Prostate; 77(3):321-333.
Govey PM, Jacobs JM, Tilton SC, Loiselle AE, Zhang Y, Freeman WM, Waters KM, Karin NJ, Donahue HJ (2014). Integrative transcriptomic and proteomic analysis of osteocytic cells exposed to fluid flow reveals novel mechano-sensitive signaling pathways. J Biomech; 47(8):1838-45.
Govey PM, Kawasawa YI, Donahue HJ (2015). Mapping the osteocytic cell response to fluid flow using RNA-Seq. J Biomech; 48(16):4327-32.
Haugh MG, Vaughan TJ, McNamara LM (2015). The role of integrin α(V)β(3) in osteocyte mechanotransduction. J Mech Behav Biomed Mater; 42:67-75.
Huang J, Romero-Suarez S, Lara N, Mo C, Kaja S, Brotto L, Dallas SL, Johnson ML, Jähn K, Bonewald LF, Brotto M (2017). Crosstalk between MLO-Y4 osteocytes and C2C12 muscle cells is mediated by the Wnt/β-catenin pathway. JBMR Plus; 1(2):86-100.
Jing D, Lu XL, Luo E, Sajda P, Leong PL, Guo XE (2013). Spatiotemporal properties of intracellular calcium signaling in osteocytic and osteoblastic cell networks under fluid flow. Bone; 53(2):531-40.
Juffer P, Jaspers RT, Lips P, Bakker AD, Klein-Nulend J (2012). Expression of muscle anabolic and metabolic factors in mechanically loaded MLO-Y4 osteocytes. Am J Physiol Endocrinol Metab; 302(4):E389-95.
Kalogeropoulos M, Varanasi SS, Olstad OK, Sanderson P, Gautvik VT, Reppe S, Francis RM, Gautvik KM, Birch MA, Datta HK (2010). Zic1 transcription factor in bone: neural developmental protein regulates mechanotransduction in osteocytes. FASEB J; 24(8):2893-903.
Kamel MA, Picconi JL, Lara-Castillo N, Johnson ML (2010). Activation of β-catenin signaling in MLO-Y4 osteocytic cells versus 2T3 osteoblastic cells by fluid flow shear stress and PGE2: Implications for the study of mechanosensation in bone. Bone; 47(5):872-81.
Kitase Y, Barragan L, Qing H, Kondoh S, Jiang JX, Johnson ML, Bonewald LF (2010). Mechanical induction of PGE2 in osteocytes blocks glucocorticoid-induced apoptosis through both the β-catenin and PKA pathways. J Bone Miner Res; 25(12):2657-68.
Kitase Y, Lee S, Gluhak-Heinrich J, Johnson ML, Harris SE, Bonewald LF (2014). CCL7 is a protective factor secreted by mechanically loaded osteocytes. J Dent Res; 93(11):1108-15.
Kulkarni RN, Bakker AD, Everts V, Klein-Nulend J (2010). Inhibition of osteoclastogenesis by mechanically loaded osteocytes: involvement of MEPE. Calcif Tissue Int; 87(5):461-8.
Kulkarni RN, Bakker AD, Everts V, Klein-Nulend J (2012a). Mechanical loading prevents the stimulating effect of IL-1β on osteocyte-modulated osteoclastogenesis. Biochem Biophys Res Commun; 420(1):11-6.
Kulkarni RN, Bakker AD, Gruber EV, Chae TD, Veldkamp JB, Klein-Nulend J, Everts V (2012b). MT1-MMP modulates the mechanosensitivity of osteocytes. Biochem Biophys Res Commun; 417(2):824-9.
Li J, Rose E, Frances D, Sun Y, You L (2012). Effect of oscillating fluid flow stimulation on osteocyte mRNA expression. J Biomech; 45(2):247-51.
Li X, Liu C, Li P, Li S, Zhao Z, Chen Y, Huo B, Zhang D (2013). Connexin 43 is a potential regulator in fluid shear stress-induced signal transduction in osteocytes. J Orthop Res; 31(12):1959-65.
Li X, Han L, Nookaew I, Mannen E, Silva MJ, Almeida M, Xiong J (2019). Stimulation of Piezo1 by mechanical signals promotes bone anabolism. Elife; 8.
Liao C, Cheng T, Wang S, Zhang C, Jin L, Yang Y (2017). Shear stress inhibits IL-17A-mediated induction of osteoclastogenesis via osteocyte pathways. Bone; 101:10-20.
Litzenberger JB, Kim JB, Tummala P, Jacobs CR (2010). Beta1 integrins mediate mechanosensitive signaling pathways in osteocytes. Calcif Tissue Int; 86(4):325-32.
Liu C, Zhang X, Wu M, You L (2015). Mechanical loading up-regulates early remodeling signals from osteocytes subjected to physical damage. J Biomech; 48(16):4221-8.
Lu XL, Huo B, Chiang V, Guo XE (2012a). Osteocytic network is more responsive in calcium signaling than osteoblastic network under fluid flow. J Bone Miner Res; 27(3):563-74.
Lu XL, Huo B, Park M, Guo XE (2012b). Calcium response in osteocytic networks under steady and oscillatory fluid flow. Bone; 51(3):466-73.
Maycas M, Ardura JA, de Castro LF, Bravo B, Gortázar AR, Esbrit P (2015). Role of the Parathyroid Hormone Type 1 Receptor (PTH1R) as a Mechanosensor in Osteocyte Survival. J Bone Miner Res; 30(7):1231-44.
Maycas M, Portolés MT, Matesanz MC, Buendía I, Linares J, Feito MJ, Arcos D, Vallet-Regí M, Plotkin LI, Esbrit P, Gortázar AR (2017). High glucose alters the secretome of mechanically stimulated osteocyte-like cells affecting osteoclast precursor recruitment and differentiation. J Cell Physiol; 232(12):3611-3621.
Middleton K, Kondiboyina A, Borrett M, Cui Y, Mei X, You L (2018). Microfluidics approach to investigate the role of dynamic similitude in osteocyte mechanobiology. J Orthop Res; 36(2):663-671.
Rath AL, Bonewald LF, Ling J, Jiang JX, Van Dyke ME, Nicolella DP (2010). Correlation of cell strain in single osteocytes with intracellular calcium, but not intracellular nitric oxide, in response to fluid flow. J Biomech; 43(8):1560-4.
Reilly GC, Haut TR, Yellowley CE, Donahue HJ, Jacobs CR (2003). Fluid flow induced PGE2 release by bone cells is reduced by glycocalyx degradation whereas calcium signals are not. Biorheology; 40(6):591-603.
Ren J, Wang XH, Wang GC, Wu JH (2013). 17β estradiol regulation of connexin 43-based gap junction and mechanosensitivity through classical estrogen receptor pathway in osteocyte-like MLO-Y4 cells. Bone; 53(2):587-96.
Riquelme MA, Gu S, Hua R, Jiang JX (2021). Mechanotransduction via the coordinated actions of integrins, PI3K signaling and Connexin hemichannels. Bone Res; 9(1):8.
Santos A, Bakker AD, Zandieh-Doulabi B, Semeins CM, Klein-Nulend J (2009). Pulsating fluid flow modulates gene expression of proteins involved in Wnt signaling pathways in osteocytes. J Orthop Res; 27(10):1280-7.
Santos A, Bakker AD, Zandieh-Doulabi B, de Blieck-Hogervorst JM, Klein-Nulend J (2010). Early activation of the beta-catenin pathway in osteocytes is mediated by nitric oxide, phosphatidyl inositol-3 kinase/Akt, and focal adhesion kinase. Biochem Biophys Res Commun; 391(1):364-9.
Seref-Ferlengez Z, Maung S, Schaffler MB, Spray DC, Suadicani SO, Thi MM (2016). P2X7R-Panx1 Complex Impairs Bone Mechanosignaling under High Glucose Levels Associated with Type-1 Diabetes. PLoS One; 11(5):e0155107.
Shah KM, Orton P, Mani N, Wilkinson JM, Gartland A (2017). Osteocyte physiology and response to fluid shear stress are impaired following exposure to cobalt and chromium: Implications for bone health following joint replacement. J Orthop Res; 35(8):1716-1723.
Thi MM, Kojima T, Cowin SC, Weinbaum S, Spray DC (2003). Fluid shear stress remodels expression and function of junctional proteins in cultured bone cells. Am J Physiol Cell Physiol; 284(2):C389-403.
Thi MM, Suadicani SO, Spray DC (2010). Fluid flow-induced soluble vascular endothelial growth factor isoforms regulate actin adaptation in osteoblasts. J Biol Chem; 285(40):30931-41.
Wang S, Li S, Hu M, Huo B (2019). Calcium response in bone cells at different osteogenic stages under unidirectional or oscillatory flow. Biomicrofluidics; 13(6):064117.
Xia X, Batra N, Shi Q, Bonewald LF, Sprague E, Jiang JX (2010). Prostaglandin promotion of osteocyte gap junction function through transcriptional regulation of connexin 43 by glycogen synthase kinase 3/beta-catenin signaling. Mol Cell Biol; 30(1):206-19.
Xu H, Zhang J, Wu J, Guan Y, Weng Y, Shang P (2012). Oscillatory fluid flow elicits changes in morphology, cytoskeleton and integrin-associated molecules in MLO-Y4 cells, but not in MC3T3-E1 cells. Biol Res; 45(2):163-9.
Xu H, Guan Y, Wu J, Zhang J, Duan J, An L, Shang P (2014). Polycystin 2 is involved in the nitric oxide production in responding to oscillating fluid shear in MLO-Y4 cells. J Biomech; 47(2):387-91.
Yan Z, Wang P, Wu J, Feng X, Cai J, Zhai M, Li J, Liu X, Jiang M, Luo E, Jing D (2018). Fluid shear stress improves morphology, cytoskeleton architecture, viability, and regulates cytokine expression in a time-dependent manner in MLO-Y4 cells. Cell Biol Int; 42(10):1410-1422.
Zhang JN, Zhao Y, Liu C, Han ES, Yu X, Lidington D, Bolz SS, You L (2015). The role of the sphingosine-1-phosphate signaling pathway in osteocyte mechanotransduction. Bone; 79:71-8.

Zhang K, Barragan-Adjemian C, Ye L, Kotha S, Dallas M, Lu Y, Zhao S, Harris M, Harris SE, Feng JQ, Bonewald LF (2006). E11/gp38 selective expression in osteocytes: regulation by mechanical strain and role in dendrite elongation. Mol Cell Biol; 26(12):4539-52.
