# Supplementary material for: Fluid flow shear stress and tissue remodeling—an orthodontic perspective: evidence synthesis and differential gene expression network analysis
Source: Front Bioeng Biotechnol. 2023 Sep 18;11:1256825. doi: 10.3389/fbioe.2023.1256825 (PMC10545883; doi:10.3389/fbioe.2023.1256825)
Supplement: Supplementary file 6 [file DataSheet1.pdf]

Supplement 1

"Fluid Flow Shear Stress and Tissue Remodeling – an Orthodontic Perspective: Evidence Synthesis and Differential Gene Expression Network analysis"

Search formula & Exclusion criteria

Search formula

Field	Mechanical stimulation	Cells	Setting
orthodont* OR<br>tooth movement OR<br>ortho* OR<br>Dent* OR<br>Oral OR<br>Mechanotransduction OR<br>bone remodeling OR<br>Stomatology OR<br>mechanosens* OR<br>calcium signaling OR<br>osteogenesis OR<br>orthodontic tooth movement OR<br>osteoblastogenesis OR<br>Periodont*	Extracellular Fluid flow OR<br>interstitial fluid flow OR<br>"Shear Strength"[MeSH] OR<br>"Stress, Mechanical"[MeSH] OR<br>fluid flow OR<br>pulsatile flow OR<br>flow chamber OR<br>shear stress OR<br>FSS OR<br>fluid shear stress OR<br>shear strength OR<br>laminar shear stress OR<br>pulsatile fluid flow	PDL OR<br>hPDLCs OR<br>hPDLFs OR<br>hPDLs OR<br>bone cells OR<br>osteo* OR<br>bone OR<br>periodontal ligament OR<br>fibroblast* OR<br>MLO-Y4 OR<br>human osteoblast-like cells OR<br>human fetal osteoblast-like cells OR<br>mesenchymal stem cells OR<br>primary human osteoblasts OR<br>progenitor cell* OR<br>stem cell* OR<br>human PDL-cells OR<br>human PDL-fibroblasts OR<br>human PDLFs OR<br>human PDLs	cell* OR<br>model OR<br>in vitro

Search: ("bone remodeling"[tw] OR "calcium signaling"[tw] OR "orthodontic tooth movement"[tw] OR "tooth movement"[tw] OR dent* OR mechanosens*[tw] OR mechanotransduction[tw] OR oral OR ortho* OR orthodont* OR osteoblastogenesis OR osteogenesis[tw] OR periodont* OR stomatology) AND ("Extracellular Fluid flow"[tw] OR "flow chamber"[tw] OR "fluid flow"[tw] OR "fluid shear stress"[tw] OR "interstitial fluid flow"[tw] OR "laminar shear stress"[tw] OR "pulsatile flow"[tw] OR "pulsatile fluid flow"[tw] OR "Shear Strength"[MeSH] OR "shear stress"[tw] OR "Stress, Mechanical"[MeSH] OR FSS[tw]) AND ("bone cells"[tw] OR "human fetal osteoblast-like cells"[tw] OR "human osteoblast-like cells"[tw] OR "human PDL-cells"[tw] OR "human PDL-fibroblasts"[tw] OR "human PDLFs"[tw] OR "human PDLs"[tw] OR "mesenchymal stem cells"[tw] OR "MLO-Y4"[tw] OR "periodontal ligament"[tw] OR "primary human osteoblasts"[tw] OR "progenitor cell*"[tw] OR "stem cell*"[tw] OR bone[tw] OR fibroblast*[tw] OR hPDLCs[tw] OR hPDLFs[tw] OR hPDLs[tw] OR osteo*[tw] OR PDL[tw]) AND ("in vitro"[tw] OR cell*[tw] OR model[tw])
Filters: from 1000/1/1 - 2021/7/19 Sort by: Publication Date

Exclusion criteria

Reason for exclusion

After full text reading, the articles were filtered based on eligibility criteria. Articles not fulfilling these criteria were identified and excluded. Along with assigning the reason for exclusion, the citation and number of excluded articles under each criterion was documented and quantitively reported in PRISMA 2020 workflow.

Total number of excluded studies: 98

Exclusion Criteria	Reference
Review article/Short communication/protocol study (5 articles)	Riddle et al. (2008b); Riddle et al. (2008a); Sarkar et al. (2011); Rosser and Bonewald (2012); Wolfe et al. (2016)
Primary cells independent of origin, not fulfilling the inclusion criteria (34 articles)	Klein-Nulend et al. (2003); Burger and Klein-Nulend (1998); Ishihara et al. (2013); Vezeridis et al. (2006); Li et al. (2018); Xing et al. (2014); Young et al. (2009); Nauman et al. (2001); Reich et al. (1990); Liu et al. (2010b); Allen et al. (2000); Tan et al. (2010); Birmingham et al. (2015); Reich and Frangos (1993); Reich et al. (1997); Robling et al. (2016); Matsugaki et al. (2020); Lu et al. (2016); Kreke and Goldstein (2004); Sharp et al. (2009); Zhong et al. (2013); Birmingham et al. (2016); Coughlin et al. (2016); Kreke et al. (2008); Kreke et al. (2005); Ramaswamy et al. (2010); Bao et al. (2018); Young et al. (2011); Young et al. (2010); Qiu et al. (2012); Liu et al. (2015); Zhou et al. (2020); McBride et al. (2008); Lei et al. (2020)
In vivo, in silico, or *ex vivo* models (2 articles)	Xiao et al. (2011); Qiu et al. (2014)
3D cell culture models (6 articles)	Martinez et al. (2013); Liu et al. (2012); Liu et al. (2011); Kim and Ma (2012); Grayson et al. (2010); Xue and Cartmell (2020)
No FSS-related reporting on gene or protein expression (19 articles)	Glossop and Cartmell (2010); Park et al. (2010); Stavenschi et al. (2018); Etter et al. (2018); Mei et al. (2019); Jekir and Donahue (2009); Mulcahy et al. (2011); Cabahug-Zuckerman et al. (2018); Villaseñor et al. (2019); Verbruggen et al. (2021); Riehl et al. (2015); Yu et al. (2018); Cheung et al. (2011); Jiang and Cherian (2003); Imai et al. (2009); Papanicolaou et al. (2009); Chen et al. (2019); Lim et al. (2016); Bratengeier et al. (2020)
Unclear/unrelated *in vitro* fluid-flow model design or experiment (15 articles)	Elliot et al. (2004); Jackson et al. (2008); Bravo et al. (2017); El Haj et al. (2015); Kokkinos et al. (2009); Liu et al. (2010a); Wang et al. (2019); Bivi et al. (2013); Fan et al. (2020); Qiu and Li (2017); Thi et al. (2013); Ma et al. (2018); Wang et al. (2018); Hum et al. (2014); Kim et al. (2014)
Article not published in English (12 articles)	Liu et al. (2008); Li et al. (2019); Cui et al. (2012); Kamioka and Yamashiro (2008); Hakeda et al. (2000); Shen et al. (2012); Zhang et al. (2009); Li et al. (2010); Xiang et al. (2012); Wu et al. (2004); Zhang et al. (2006); Chen et al. (2005)
Unhealthy/sick patient or controls (3 articles)	Bakker et al. (2006); Kaneko et al. (2014); Bakker et al. (2005)
Co-culture studies unless monoculture data is provided (2 articles)	Middleton et al. (2017); Taylor et al. (2007)

References

Allen FD, Hung CT, Pollack SR, Brighton CT (2000). Serum modulates the intracellular calcium response of primary cultured bone cells to shear flow. J Biomech; 33(12):1585-91.

Bakker AD, Klein-Nulend J, Tanck E, Albers GH, Lips P, Burger EH (2005). Additive effects of estrogen and mechanical stress on nitric oxide and prostaglandin E2 production by bone cells from osteoporotic donors. Osteoporos Int; 16(8):983-9.

Bakker AD, Klein-Nulend J, Tanck E, Heyligers IC, Albers GH, Lips P, Burger EH (2006). Different responsiveness to mechanical stress of bone cells from osteoporotic versus osteoarthritic donors. Osteoporos Int; 17(6):827-33.

Bao X, Li Z, Liu H, Feng K, Yin F, Li H, Qin J (2018). Stimulation of chondrocytes and chondroinduced mesenchymal stem cells by osteoinduced mesenchymal stem cells under a fluid flow stimulus on an integrated microfluidic device. Mol Med Rep; 17(2):2277-2288.

Birmingham E, Kreipke TC, Dolan EB, Coughlin TR, Owens P, McNamara LM, Niebur GL, McHugh PE (2015). Mechanical stimulation of bone marrow in situ induces bone formation in trabecular explants. Ann Biomed Eng; 43(4):1036-50.

Birmingham E, Niebur GL, McNamara LM, McHugh PE (2016). An Experimental and Computational Investigation of Bone Formation in Mechanically Loaded Trabecular Bone Explants. Ann Biomed Eng; 44(4):1191-203.

Bivi N, Pacheco-Costa R, Brun LR, Murphy TR, Farlow NR, Robling AG, Bellido T, Plotkin LI (2013). Absence of Cx43 selectively from osteocytes enhances responsiveness to mechanical force in mice. J Orthop Res; 31(7):1075-81.

Bratengeier C, Liszka A, Hoffman J, Bakker AD, Fahlgren A (2020). High shear stress amplitude in combination with prolonged stimulus duration determine induction of osteoclast formation by hematopoietic progenitor cells. FASEB J; 34(3):3755-3772.

Bravo B, García de Durango C, González Á, Gortázar AR, Santos X, Forteza-Vila J, Vidal-Vanaclocha F (2017). Opposite Effects of Mechanical Action of Fluid Flow on Proangiogenic Factor Secretion From Human Adipose-Derived Stem Cells With and Without Oxidative Stress. J Cell Physiol; 232(8):2158-2167.

Burger EH, Klein-Nulend J (1998). Microgravity and bone cell mechanosensitivity. Bone; 22(5 Suppl):127s-130s.

Cabahug-Zuckerman P, Stout RF, Jr., Majeska RJ, Thi MM, Spray DC, Weinbaum S, Schaffler MB (2018). Potential role for a specialized β(3) integrin-based structure on osteocyte processes in bone mechanosensation. J Orthop Res; 36(2):642-652.

Chen M, Liang X, Wang W, Wang H, Song H, Sun H, Zhu B (2005). [A biomechanical system that can apply fluid shear stress to osteoclast-like cells in vitro]. Sheng Wu Yi Xue Gong Cheng Xue Za Zhi; 22(2):288-92.

Chen WT, Hsu WT, Yen MH, Changou CA, Han CL, Chen YJ, Cheng JY, Chang TH, Lee OK, Ho JH (2019). Alteration of mesenchymal stem cells polarity by laminar shear stimulation promoting β-catenin nuclear localization. Biomaterials; 190-191:1-10.

Cheung WY, Liu C, Tonelli-Zasarsky RM, Simmons CA, You L (2011). Osteocyte apoptosis is mechanically regulated and induces angiogenesis in vitro. J Orthop Res; 29(4):523-30.

Coughlin TR, Schiavi J, Alyssa Varsanik M, Voisin M, Birmingham E, Haugh MG, McNamara LM, Niebur GL (2016). Primary cilia expression in bone marrow in response to mechanical stimulation in explant bioreactor culture. Eur Cell Mater; 32:111-22.

Cui L, Li XT, Zhang D (2012). Effect of fluid flow-induced shear stress on osteoclast formation induced by osteocyte. Zhongguo Yi Xue Ke Xue Yuan Xue Bao; 34(3):207-11.

El Haj AJ, Glossop JR, Sura HS, Lees MR, Hu B, Wolbank S, van Griensven M, Redl H, Dobson J (2015). An in vitro model of mesenchymal stem cell targeting using magnetic particle labelling. J Tissue Eng Regen Med; 9(6):724-33.

Elliot KJ, Millward-Sadler SJ, Wright MO, Robb JE, Wallace WH, Salter DM (2004). Effects of methotrexate on human bone cell responses to mechanical stimulation. Rheumatology (Oxford); 43(10):1226-31.

Etter JN, Karasinski M, Ware J, Oldinski RA (2018). Dual-crosslinked homogeneous alginate microspheres for mesenchymal stem cell encapsulation. J Mater Sci Mater Med; 29(9):143.

Fan Y, Jalali A, Chen A, Zhao X, Liu S, Teli M, Guo Y, Li F, Li J, Siegel A, Yang L, Liu J, Na S, Agarwal M, Robling AG, Nakshatri H, Li BY, Yokota H (2020). Skeletal loading regulates breast cancer-associated osteolysis in a loading intensity-dependent fashion. Bone Res; 8:9.

Glossop JR, Cartmell SH (2010). Tensile strain and magnetic particle force application do not induce MAP3K8 and IL-1B differential gene expression in a similar manner to fluid shear stress in human mesenchymal stem cells. J Tissue Eng Regen Med; 4(7):577-9.

Grayson WL, Bhumiratana S, Grace Chao PH, Hung CT, Vunjak-Novakovic G (2010). Spatial regulation of human mesenchymal stem cell differentiation in engineered osteochondral constructs: effects of pre-differentiation, soluble factors and medium perfusion. Osteoarthritis Cartilage; 18(5):714-23.

Hakeda Y, Arakawa T, Ogasawara A, Kumegawa M (2000). [Recent progress in studies on osteocytes--osteocytes and mechanical stress]. Kaibogaku Zasshi; 75(5):451-6.

Hum JM, Day RN, Bidwell JP, Wang Y, Pavalko FM (2014). Mechanical loading in osteocytes induces formation of a Src/Pyk2/MBD2 complex that suppresses anabolic gene expression. PLoS One; 9(5):e97942.

Imai S, Heino TJ, Hienola A, Kurata K, Büki K, Matsusue Y, Väänänen HK, Rauvala H (2009). Osteocyte-derived HB-GAM (pleiotrophin) is associated with bone formation and mechanical loading. Bone; 44(5):785-94.

Ishihara Y, Sugawara Y, Kamioka H, Kawanabe N, Hayano S, Balam TA, Naruse K, Yamashiro T (2013). Ex vivo real-time observation of Ca(2+) signaling in living bone in response to shear stress applied on the bone surface. Bone; 53(1):204-15.

Jackson WM, Jaasma MJ, Baik AD, Keaveny TM (2008). Over-expression of alpha-actinin with a GFP fusion protein is sufficient to increase whole-cell stiffness in human osteoblasts. Ann Biomed Eng; 36(10):1605-14.

Jekir MG, Donahue HJ (2009). Gap junctions and osteoblast-like cell gene expression in response to fluid flow. J Biomech Eng; 131(1):011005.

Jiang JX, Cherian PP (2003). Hemichannels formed by connexin 43 play an important role in the release of prostaglandin E(2) by osteocytes in response to mechanical strain. Cell Commun Adhes; 10(4-6):259-64.

Kamioka H, Yamashiro T (2008). [Osteocytes and mechanical stress]. Clin Calcium; 18(9):1287-93.

Kaneko K, Ito M, Naoe Y, Lacy-Hulbert A, Ikeda K (2014). Integrin αv in the mechanical response of osteoblast lineage cells. Biochem Biophys Res Commun; 447(2):352-7.

Kim J, Ma T (2012). Bioreactor strategy in bone tissue engineering: pre-culture and osteogenic differentiation under two flow configurations. Tissue Eng Part A; 18(21-22):2354-64.

Kim KM, Choi YJ, Hwang JH, Kim AR, Cho HJ, Hwang ES, Park JY, Lee SH, Hong JH (2014). Shear stress induced by an interstitial level of slow flow increases the osteogenic differentiation of mesenchymal stem cells through TAZ activation. PLoS One; 9(3):e92427.

Klein-Nulend J, Bacabac RG, Veldhuijzen JP, Van Loon JJ (2003). Microgravity and bone cell mechanosensitivity. Adv Space Res; 32(8):1551-9.

Kokkinos PA, Zarkadis IK, Panidis TT, Deligianni DD (2009). Estimation of hydrodynamic shear stresses developed on human osteoblasts cultured on Ti-6Al-4V and strained by four point bending. Effects of mechanical loading to specific gene expression. J Mater Sci Mater Med; 20(3):655-65.

Kreke MR, Goldstein AS (2004). Hydrodynamic shear stimulates osteocalcin expression but not proliferation of bone marrow stromal cells. Tissue Eng; 10(5-6):780-8.

Kreke MR, Huckle WR, Goldstein AS (2005). Fluid flow stimulates expression of osteopontin and bone sialoprotein by bone marrow stromal cells in a temporally dependent manner. Bone; 36(6):1047-55.

Kreke MR, Sharp LA, Lee YW, Goldstein AS (2008). Effect of intermittent shear stress on mechanotransductive signaling and osteoblastic differentiation of bone marrow stromal cells. Tissue Eng Part A; 14(4):529-37.

Lei X, Liu B, Wu H, Wu X, Wang XL, Song Y, Zhang SS, Li JQ, Bi L, Pei GX (2020). The effect of fluid shear stress on fibroblasts and stem cells on plane and groove topographies. Cell Adh Migr; 14(1):12-23.

Li Q, Yang L, Lu Y (2019). [Optimization of the theoretical model for growth rate of mesenchymal stem cells on three-dimensional scaffold under fluid shear stress]. Sheng Wu Yi Xue Gong Cheng Xue Za Zhi; 36(5):795-802.

Li WH, Yang XY, Xian SQ (2010). [Observation on osteoblasts responded to fluid induced shear cultured on different surfaces with scanning electron microscope]. Hua Xi Kou Qiang Yi Xue Za Zhi; 28(6):649-52.

Li Y, Yuan J, Wang Q, Sun L, Sha Y, Li Y, Wang L, Wang Z, Ma Y, Cao H (2018). The collective influence of 1, 25-dihydroxyvitamin D(3) with physiological fluid shear stress on osteoblasts. Steroids; 129:9-16.

Lim KT, Jin H, Seonwoo H, Kim HB, Kim J, Kim JW, Renji C, Choung PH, Chung JH (2016). Physical Stimulation-Based Osteogenesis: Effect of Secretion In Vitro on Fluid Dynamic Shear Stress of Human Alveolar Bone-Derived Mesenchymal Stem Cells. IEEE Trans Nanobioscience; 15(8):881-890.

Liu C, Zhao Y, Cheung WY, Gandhi R, Wang L, You L (2010a). Effects of cyclic hydraulic pressure on osteocytes. Bone; 46(5):1449-56.

Liu JS, Ma JF, Wang Q, Zhang DF, Liu CT, Liang X (2008). [Effects of fluid shear stress on apoptosis of osteoblasts and expression of bcl-2,Bax and caspase-3]. Shanghai Kou Qiang Yi Xue; 17(5):501-4.

Liu L, Shao L, Li B, Zong C, Li J, Zheng Q, Tong X, Gao C, Wang J (2011). Extracellular signal-regulated kinase1/2 activated by fluid shear stress promotes osteogenic differentiation of human bone marrow-derived mesenchymal stem cells through novel signaling pathways. Int J Biochem Cell Biol; 43(11):1591-601.

Liu L, Yu B, Chen J, Tang Z, Zong C, Shen D, Zheng Q, Tong X, Gao C, Wang J (2012). Different effects of intermittent and continuous fluid shear stresses on osteogenic differentiation of human mesenchymal stem cells. Biomech Model Mechanobiol; 11(3-4):391-401.

Liu X, Zhang X, Lee I (2010b). A quantitative study on morphological responses of osteoblastic cells to fluid shear stress. Acta Biochim Biophys Sin (Shanghai); 42(3):195-201.

Liu YS, Liu YA, Huang CJ, Yen MH, Tseng CT, Chien S, Lee OK (2015). Mechanosensitive TRPM7 mediates shear stress and modulates osteogenic differentiation of mesenchymal stromal cells through Osterix pathway. Sci Rep; 5:16522.

Lu J, Fan Y, Gong X, Zhou X, Yi C, Zhang Y, Pan J (2016). The Lineage Specification of Mesenchymal Stem Cells Is Directed by the Rate of Fluid Shear Stress. J Cell Physiol; 231(8):1752-60.

Ma YV, Lam C, Dalmia S, Gao P, Young J, Middleton K, Liu C, Xu H, You L (2018). Mechanical regulation of breast cancer migration and apoptosis via direct and indirect osteocyte signaling. J Cell Biochem; 119(7):5665-5675.

Martinez C, Rath S, Van Gulden S, Pelaez D, Alfonso A, Fernandez N, Kos L, Cheung H, Ramaswamy S (2013). Periodontal ligament cells cultured under steady-flow environments demonstrate potential for use in heart valve tissue engineering. Tissue Eng Part A; 19(3-4):458-66.

Matsugaki A, Matsuzaka T, Murakami A, Wang P, Nakano T (2020). 3D Printing of Anisotropic Bone-Mimetic Structure with Controlled Fluid Flow Stimuli for Osteocytes: Flow Orientation Determines the Elongation of Dendrites. Int J Bioprint; 6(4):293.

McBride SH, Falls T, Knothe Tate ML (2008). Modulation of stem cell shape and fate B: mechanical modulation of cell shape and gene expression. Tissue Eng Part A; 14(9):1573-80.

Mei X, Middleton K, Shim D, Wan Q, Xu L, Ma YV, Devadas D, Walji N, Wang L, Young EWK, You L (2019). Microfluidic platform for studying osteocyte mechanoregulation of breast cancer bone metastasis. Integr Biol (Camb); 11(4):119-129.

Middleton K, Al-Dujaili S, Mei X, Günther A, You L (2017). Microfluidic co-culture platform for investigating osteocyte-osteoclast signalling during fluid shear stress mechanostimulation. J Biomech; 59:35-42.

Mulcahy LE, Taylor D, Lee TC, Duffy GP (2011). RANKL and OPG activity is regulated by injury size in networks of osteocyte-like cells. Bone; 48(2):182-8.

Nauman EA, Satcher RL, Keaveny TM, Halloran BP, Bikle DD (2001). Osteoblasts respond to pulsatile fluid flow with short-term increases in PGE(2) but no change in mineralization. J Appl Physiol (1985); 90(5):1849-54.

Papanicolaou SE, Phipps RJ, Fyhrie DP, Genetos DC (2009). Modulation of sclerostin expression by mechanical loading and bone morphogenetic proteins in osteogenic cells. Biorheology; 46(5):389-99.

Park JY, Yoo SJ, Patel L, Lee SH, Lee SH (2010). Cell morphological response to low shear stress in a two-dimensional culture microsystem with magnitudes comparable to interstitial shear stress. Biorheology; 47(3-4):165-78.

Qiu J, Baik AD, Lu XL, Hillman EM, Zhuang Z, Dong C, Guo XE (2014). A noninvasive approach to determine viscoelastic properties of an individual adherent cell under fluid flow. J Biomech; 47(6):1537-41.

Qiu J, Li FF (2017). Mechanical behavior of an individual adherent MLO-Y4 osteocyte under shear flow. Biomech Model Mechanobiol; 16(1):63-74.

Qiu N, Xiao Z, Cao L, Buechel MM, David V, Roan E, Quarles LD (2012). Disruption of Kif3a in osteoblasts results in defective bone formation and osteopenia. J Cell Sci; 125(Pt 8):1945-57.

Ramaswamy S, Gottlieb D, Engelmayr GC, Jr., Aikawa E, Schmidt DE, Gaitan-Leon DM, Sales VL, Mayer JE, Jr., Sacks MS (2010). The role of organ level conditioning on the promotion of engineered heart valve tissue development in-vitro using mesenchymal stem cells. Biomaterials; 31(6):1114-25.
Reich KM, Gay CV, Frangos JA (1990). Fluid shear stress as a mediator of osteoblast cyclic adenosine monophosphate production. J Cell Physiol; 143(1):100-4.
Reich KM, Frangos JA (1993). Protein kinase C mediates flow-induced prostaglandin E2 production in osteoblasts. Calcif Tissue Int; 52(1):62-6.
Reich KM, McAllister TN, Gudi S, Frangos JA (1997). Activation of G proteins mediates flow-induced prostaglandin E2 production in osteoblasts. Endocrinology; 138(3):1014-8.
Riddle RC, Hippe KR, Donahue HJ (2008a). Chemotransport contributes to the effect of oscillatory fluid flow on human bone marrow stromal cell proliferation. J Orthop Res; 26(7):918-24.
Riddle RC, Taylor AF, Donahue HJ (2008b). Fluid flow assays. Methods Mol Biol; 455:335-45.
Riehl BD, Lee JS, Ha L, Lim JY (2015). Fluid-flow-induced mesenchymal stem cell migration: role of focal adhesion kinase and RhoA kinase sensors. J R Soc Interface; 12(104):20141351.
Robling AG, Kang KS, Bullock WA, Foster WH, Murugesh D, Loots GG, Genetos DC (2016). Sost, independent of the non-coding enhancer ECR5, is required for bone mechanoadaptation. Bone; 92:180-188.
Rosser J, Bonewald LF (2012). Studying osteocyte function using the cell lines MLO-Y4 and MLO-A5. Methods Mol Biol; 816:67-81.
Sarkar D, Zhao W, Gupta A, Loh WL, Karnik R, Karp JM (2011). Cell surface engineering of mesenchymal stem cells. Methods Mol Biol; 698:505-23.
Sharp LA, Lee YW, Goldstein AS (2009). Effect of low-frequency pulsatile flow on expression of osteoblastic genes by bone marrow stromal cells. Ann Biomed Eng; 37(3):445-53.
Shen Y, Ouyang K, Wu Y, Xu Y (2012). [Feasibility of quantified fluid shear stress loading on osteoblasts through rocking system]. Sheng Wu Yi Xue Gong Cheng Xue Za Zhi; 29(5):889-93.
Stavenschi E, Corrigan MA, Johnson GP, Riffault M, Hoey DA (2018). Physiological cyclic hydrostatic pressure induces osteogenic lineage commitment of human bone marrow stem cells: a systematic study. Stem Cell Res Ther; 9(1):276.
Tan L, Meyer T, Pfau B, Hofmann T, Tan TW, Jones D (2010). Rapid vinculin exchange dynamics at focal adhesions in primary osteoblasts following shear flow stimulation. J Musculoskelet Neuronal Interact; 10(1):92-9.
Taylor AF, Saunders MM, Shingle DL, Cimbala JM, Zhou Z, Donahue HJ (2007). Mechanically stimulated osteocytes regulate osteoblastic activity via gap junctions. Am J Physiol Cell Physiol; 292(1):C545-52.
Thi MM, Suadicani SO, Schaffler MB, Weinbaum S, Spray DC (2013). Mechanosensory responses of osteocytes to physiological forces occur along processes and not cell body and require αVβ3 integrin. Proc Natl Acad Sci U S A; 110(52):21012-7.
Verbruggen SW, Thompson CL, Duffy MP, Lunetto S, Nolan J, Pearce OMT, Jacobs CR, Knight MM (2021). Mechanical Stimulation Modulates Osteocyte Regulation of Cancer Cell Phenotype. Cancers (Basel); 13(12).
Vezeridis PS, Semeins CM, Chen Q, Klein-Nulend J (2006). Osteocytes subjected to pulsating fluid flow regulate osteoblast proliferation and differentiation. Biochem Biophys Res Commun; 348(3):1082-8.
Villaseñor A, Aedo-Martín D, Obeso D, Erjavec I, Rodríguez-Coira J, Buendía I, Ardura JA, Barbas C, Gortazar AR (2019). Metabolomics reveals citric acid secretion in mechanically-stimulated osteocytes is inhibited by high glucose. Sci Rep; 9(1):2295.
Wang W, Sarazin BA, Kornilowicz G, Lynch ME (2018). Mechanically-Loaded Breast Cancer Cells Modify Osteocyte Mechanosensitivity by Secreting Factors That Increase Osteocyte Dendrite Formation and Downstream Resorption. Front Endocrinol (Lausanne); 9:352.
Wang Z, Ishihara Y, Ishikawa T, Hoshijima M, Odagaki N, Ei Hsu Hlaing E, Kamioka H (2019). Screening of key candidate genes and pathways for osteocytes involved in the differential response to different types of mechanical stimulation using a bioinformatics analysis. J Bone Miner Metab; 37(4):614-626.
Wolfe RP, Guidry JB, Messina SL, Ahsan T (2016). Applying Shear Stress to Pluripotent Stem Cells. Methods Mol Biol; 1341:377-89.
Wu J, Chen H, Cao H, Zhou J, Zhang L, Sung KL (2004). [Alterations in expression of F-actin and DNA of fluid shear stress treated-mesenchymal stem cells affected by titanium particles loading]. Sheng Wu Yi Xue Gong Cheng Xue Za Zhi; 21(1):1-7.
Xiang YH, Shao MF, Song Y, Yang Z, Chen XD, Fu Q (2012). [Effect of cytoskeleton reorganization inhibition on the activation of extracellular signal-regulated kinase in osteoblasts by fluid shear stress]. Zhonghua Kou Qiang Yi Xue Za Zhi; 47(11):680-3.
Xiao Z, Dallas M, Qiu N, Nicolella D, Cao L, Johnson M, Bonewald L, Quarles LD (2011). Conditional deletion of Pkd1 in osteocytes disrupts skeletal mechanosensing in mice. FASEB J; 25(7):2418-32.
Xing J, Li Y, Lin M, Wang J, Wu J, Ma Y, Wang Y, Yang L, Luo Y (2014). Surface chemistry modulates osteoblasts sensitivity to low fluid shear stress. J Biomed Mater Res A; 102(11):4151-60.
Xue R, Cartmell S (2020). A simple in vitro biomimetic perfusion system for mechanotransduction study. Sci Technol Adv Mater; 21(1):635-640.
Young SR, Gerard-O'Riley R, Kim JB, Pavalko FM (2009). Focal adhesion kinase is important for fluid shear stress-induced mechanotransduction in osteoblasts. J Bone Miner Res; 24(3):411-24.
Young SR, Gerard-O'Riley R, Harrington M, Pavalko FM (2010). Activation of NF-kappaB by fluid shear stress, but not TNF-alpha, requires focal adhesion kinase in osteoblasts. Bone; 47(1):74-82.
Young SR, Hum JM, Rodenberg E, Turner CH, Pavalko FM (2011). Non-overlapping functions for Pyk2 and FAK in osteoblasts during fluid shear stress-induced mechanotransduction. PLoS One; 6(1):e16026.
Yu K, Sellman DP, Bahraini A, Hagan ML, Elsherbini A, Vanpelt KT, Marshall PL, Hamrick MW, McNeil A, McNeil PL, McGee-Lawrence ME (2018). Mechanical loading disrupts osteocyte plasma membranes which initiates mechanosensation events in bone. J Orthop Res; 36(2):653-662.
Zhang Y, Chen H, Huang H, Xu X, Tang X, Yin G, Wu J (2006). [Mechanical environment of rotating bioreactor and its effect on cell growth]. Sheng Wu Yi Xue Gong Cheng Xue Za Zhi; 23(2):400-4.
Zhang YP, Qin F, Wu CJ, Li YR, Chen R, Shao MF, Fu Q (2009). [Effects of LIMK2 RNA interference on the mechanosensitivity of c-fos gene in osteoblast]. Zhonghua Yi Xue Za Zhi; 89(44):3143-6.

Zhong W, Tian K, Zheng X, Li L, Zhang W, Wang S, Qin J (2013). Mesenchymal stem cell and chondrocyte fates in a multishear microdevice are regulated by Yes-associated protein. Stem Cells Dev; 22(14):2083-93.
Zhou T, Gao B, Fan Y, Liu Y, Feng S, Cong Q, Zhang X, Zhou Y, Yadav PS, Lin J, Wu N, Zhao L, Huang D, Zhou S, Su P, Yang Y (2020). Piezo1/2 mediate mechanotransduction essential for bone formation through concerted activation of NFAT-YAP1-ß-catenin. Elife; 9.
