# Supplementary material for: Fluid flow shear stress and tissue remodeling—an orthodontic perspective: evidence synthesis and differential gene expression network analysis
Source: Front Bioeng Biotechnol. 2023 Sep 18;11:1256825. doi: 10.3389/fbioe.2023.1256825 (PMC10545883; doi:10.3389/fbioe.2023.1256825)
Supplement: Supplementary file 7 [file DataSheet5.pdf]

Supplement 5

"Fluid Flow Shear Stress and Tissue Remodeling – an Orthodontic Perspective: Evidence Synthesis and Differential Gene Expression Network analysis"

Commonly investigated genes and metabolites mentioned by at least three studies

The commonly investigated genes and small molecules data mentioned by at least three studies were extracted from supplement 3. Data was then organised based on fluid flow profile (Oscillatory Laminar, Steady Laminar, Pulsatile Laminar). Then the regulation description of each gene was highlighted based on the regulation description as follows:

Increase
Decrease
No change
Increase with plateau
Decrease with plateau
Others

Contents

5.1 Human mesenchymal stem cells 2

5.2 Human osteoblasts 9

5.3 Human PDL cells 11

5.4 Mouse osteoblasts 12

5.5 Mouse osteocytes 14

5.1 Human mesenchymal stem cells

Official gene symbol or metabolite abbreviation	Reference	Cell type (age/ number and sex of donor (health status), tooth type, isolation method, passages used, cell density/confluency)	Flow type (steady laminar, pulsatile laminar, or oscillatory laminar)	FSS-duration and frequency (Hz)	FSS magnitude	FSS apparatus	Gene expression: increase, decrease, no change (method w/ reference gene); methods: (RT-qPCR, sqPCR)	Gene expression: when it reaches peak and peak's magnitude (fold change; relative gene expression; times or ratio; unclear = ?)	Protein expression: increase, decrease, no change (method w/reference); methods: ELISA, WB, RIA, EMSA, IF	Protein expression: when it reaches peak and peak's magnitude (times or ratio; unclear = ?)	Remarks including fluorescent microscopy assay
ALP/ALPL	Li et al. (2004)	MSCs (BioWhittaker) (n.g./ n.g. (healthy), n.g., Ficoll paque, P2-8, n.g./n.g.)	Oscillatory laminar	2h FSS (3d post FSS incubation) /1Hz	10dyn/cm²	Custom-made			Decrease (colorimetric assay)	3d post-FSS: 0.02µg/mL; 0.02/0.03 = 0.66 (ratio-calc)†	ALP acitivity with p-nitrophenol substrate; unspecific!
	Lim et al. (2014)	hABMSCs (n.g./ n.g. (n.g.), n.g., n.g., n.g., 10⁴ cells per cm²/n.g.)	Oscillatory laminar	10d @ 10, 30, 60, 120, 180min/d / n.g.	0.86–1.51dyn/cm²	Rotational orbital shaker			Increase with plateau (activity)	30min/d: 6.1ng/h/protein; 6.1/4.7 = 1.3 (ratio-calc)†<br>60min/d: 5.5ng/h/protein; 5.5/4.7 = 1.17 (ratio-calc)†<br>120min/d: 5.6ng/h/protein; 5.6/4.7 = 1.19 (ratio-calc)†	ALP mentioned in footnote of Table 1; ALP activity shown in figure but determination not specified in methods!
	Lim et al. (2013)	hABMSCs (n.g./ n.g. (n.g.), n.g., n.g., P3-5, n.g./n.g.)	Oscillatory laminar	5, 10, 30, 60, and 120min/d for 2w or 3w / n.g.	0.01-0.0205dyn/cm² (max. 0.00205Pa)	Rocking culture system (Vision Ltd.)	2w: increase with plateau (5-30min/d) then maximum increase at 60min (sqPCR, GAPDH)<br>3w: increase (sqPCR, GAPDH)	5min/d for 2w: 2.5 (rel.)†<br>10min/d for 2w: 2.5 (rel.)†<br>30min/d for 2w: 2.4 (rel.)†<br>60min/d for 2w: 5.9 (rel.)†<br>60min/d for 3w: 2.6 (rel.)†	2w: increase (colorimetric assay)	30min/d for 2w: 7.2ng/h/protein; 7.2/5.3 = 1.4 (ratio-calc)†	Genbank: BC090861
	Yourek et al. (2010)	Isolated bone marrow hMSCs (20-26y/7M and 1F (Healthy), n.g., Ficoll paque, P3-5, 7.6–8.6×10³ cells per cm²/n.g.) ("hMSC")	Steady laminar	24h: immediately after FSS and 3d, 7d, 8d, 11d post-FSS/n.g.	9dyn/cm²	Custom-made			cellular ALP @ 7d post-FSS: increase (activity)<br>cellular ALP @ 8d post-FSS: decrease (activity)<br>cellular ALP @ 11d post-FSS: decrease (activity)	hMSCs cellular ALP @ 7d: 14.5mU/ng; 14.5/14.2 = 1.02 (ratio-calc)†	
	Sonam et al. (2016)	hMSC (Lonza) (n.g./ n.g. (n.g.), n.g., n.g., P1-5, 1000 cells per cm² /n.g.)	Steady laminar	48h/n.g.	1Pa	Custom-made (planar topography)	Increase (RT-qPCR; GAPDH)	48h: 3.5 (rel.)†	Increase (IF)	48h: 2.7 (ratio)†	
	Becquart et al. (2016)	Human MSCs (n.g./ 1 and 6 n.g. (n.g.), n.g., Ficoll paque, P2, cell n.g./n.g.)	Steady laminar	30min (sampling 0h, 1h, 6h post-FSS) / 2.8Hz	0.7Pa	µ-Slide w/ Ibidi pump system (Ibidi, DE)	Increase (RT-qPCR, 18S)	1h post-FSS: 2.1 (FC)†			
BMP2	Hoey et al. (2012)	MSCs harvested from human bone marrow (Lonza, Walkersville, MD, USA, http://www.lonza. com) (n.g./ n.g. (n.g.), n.g., n.g., P4, n.g./n.g.)	Oscillatory laminar	2h (sampling 30min, 2h, 24h, 48h post-FSS)/n.g.	1.0Pa (28ml/min)	Custom-made	Increase followed by decrease then baseline (RT-qPCR; GAPDH)	2h post-FSS: 4.6/0.9 = 5.1 (ratio-calc)†<br>24h post-FSS: 0.2/0.3= 0.67 (ratio-calc)†<br>48h post-FSS: 0.35/0.35 = 1 (ratio-calc)†			
	Lim et al. (2014)	hABMSCs (n.g./ n.g. (n.g.), n.g., n.g., n.g., 10⁴ cells per cm²/n.g.)	Oscillatory laminar	10d @ 10, 30, 60, 120, 180min/d / n.g.	0.86–1.51dyn/cm²	Rotational orbital shaker			Increase (ELISA)	120min/d: 2718.5pg/mL; 2718.5/1688.5 = 1.61 (ratio-calc)†	

Official gene symbol or metabolite abbreviation	Reference	Cell type (age/ number and sex of donor (health status), tooth type, isolation method, passages used, cell density/confluency)	Flow type (steady laminar, pulsatile laminar, or oscillatory laminar)	FSS-duration and frequency (Hz)	FSS magnitude	FSS apparatus	Gene expression: increase, decrease, no change (method w/ reference gene); methods: (RT-qPCR, sqPCR)	Gene expression: when it reaches peak and peak's magnitude (fold change; relative gene expression; times or ratio; unclear = ?)	Protein expression: increase, decrease, no change (method w/reference); methods: ELISA, WB, RIA, EMSA, IF	Protein expression: when it reaches peak and peak's magnitude (times or ratio; unclear = ?)	Remarks including fluorescent microscopy assay
	Yourek et al. (2010)	Isolated bone marrow hMSCs (20-26y/7M and 1F (Healthy), n.g., Ficoll paque, P3-5, 7.6–8.6×10^3 cells per cm^2/n.g.) ("hMSC")	Steady laminar	24h (after 3d or 7d pre-culture in basal medium)/n.g.	4dyn/cm^2, 15dyn/cm^2, 22dyn/cm^2	Custom-made	4dyn/cm^2: increase (RT-qPCR, GAPDH)<br>15dyn/cm^2: increase (RT-qPCR, GAPDH)<br>22dyn/cm^2: increase (RT-qPCR, GAPDH)	4dyn/cm^2 @ 7d pre-FSS: 5.3 (FC)†<br>15dyn/cm^2 @ 7d pre-FSS: 7.0 (FC)†<br>22dyn/cm^2 @ 7d pre-FSS: 7.2 (FC)†			
PTGS2	Celil Aydemir et al. (2010)	Human mesenchymal stem cells (hMSC) (Cambrex Inc., Walkersville, MD) (n.g./ n.g. (n.g.), n.g., n.g., n.g., n.g./n.g.)	Oscillatory laminar	15min/1Hz	16dyn/cm^2 (1.6Pa)	Flexcell Inc.	Increase (RT-qPCR, GAPDH)	15min: 2.8493 (rel.)†	Increase (WB, GAPDH)	15min: 145/79 = 1.84 (ratio-calc)†	(See (Celil Aydemir et al. (2007), similar system)
	Celil Aydemir et al. (2007)	hMSCs were purchased from Cambrex (East Rutherford, NJ, USA) (n.g./ n.g. (n.g.), n.g., n.g., n.g., n.g./n.g.)	Oscillatory laminar	15min/1Hz	5–10dyn/cm^2	Flexcell Inc.	Increase (RT-qPCR, GAPDH)	15min: 2.3 (rel.)			
	Hoey et al. (2012)	MSCs harvested from human bone marrow (Lonza, Walkersville, MD, USA, http://www.lonza. com) (n.g./ n.g. (n.g.), n.g., n.g., P4, n.g./n.g.)	Oscillatory laminar	2h (sampling 30min, 2h, 24h, 48h post-FSS)/n.g.	1.0Pa (28ml/min)	Custom-made	Increase followed by decrease then baseline (RT-qPCR; GAPDH)	30min post-FSS: 1.7/0.17 = 10 (ratio-calc)†<br>24h post-FSS: 1 (ratio-calc)†<br>48h post-FSS: 1 (ratio-calc)†			
	Becquart et al. (2016)	Human MSCs (n.g./ 1 and 6 n.g. (n.g.), n.g., Ficoll paque, P2, cell n.g./n.g.)	Steady laminar	30min (sampling 0h, 1h, 6h post-FSS) / 2.8Hz	0.7Pa	µ-Slide w/ Ibidi pump system (Ibidi, DE)	Increase (RT-qPCR, 18S)	1h post-FSS: 56.7 (FC)†			
	Lee et al. (2017)	Bone marrow MSCs (n.g./ n.g. (n.g.), n.g., Ficoll paque, P1, 3×10^6 cells per ml / 80%)	Steady laminar	5min, 6h / n.g.	15dyn/cm^2	µ-slide $VI^{0.4}$ (Ibidi, DE) w/ fibronectin coating; 12-roller peristaltic pump (REGLO analog MS4/12, Ismatec)			Decrease then increase (WB, β-actin)	5min: 0.9 (ratio)†<br>6h: 2.4 (ratio)†	
	Lee et al. (2017)	Bone marrow MSCs (n.g./ n.g. (n.g.), n.g., Ficoll paque, P1, 3×10^6 cells per ml / 80%)	Steady laminar	3h, 6h / n.g.	15dyn/cm^2	µ-slide $VI^{0.4}$ (Ibidi, DE) w/ fibronectin coating; 12-roller peristaltic pump (REGLO analog MS4/12, Ismatec)	Increase (RT-qPCR, GAPDH)	6h: 5.3 (FC)†			
	Kraft et al. (2010)	human dental pulp-derived mesenchymal stem cells (PDSCs) (20y / 1M (healthy), M, dig., P2-11 and P4-23, 4×10^5 cells/n.g.) "mature"	Pulsating laminar	1h FSS (0min, 5min, 10min, 15min, 30min, 60min, 3h post-FSS incubation)/5Hz	0.6Pa, a pulse amplitude of 0.3Pa and a peak shear stress rate of 8.4Pa/s.	Custom-made	Increase (RT-qPCR, GUS)	3h post-FSS: 2.96 (FC)†			"mature" phenotype
	Kraft et al. (2010)	human dental pulp-derived mesenchymal stem cells (PDSCs) (21y / 1F (healthy), M, dig., P2-11 and P4-23, 4×10^5 cells/n.g.) "immature"	Pulsating laminar	1h FSS (0min, 5min, 10min, 15min, 30min, 60min, 3h post-FSS incubation)/5Hz	0.6Pa, a pulse amplitude of 0.3Pa and a peak shear stress rate of 8.4Pa/s.	Custom-made	Increase (RT-qPCR, GUS)	1h post-FSS: 4.9 (FC)†			"immature" phenotype

Official gene symbol or metabolite abbreviation	Reference	Cell type (age/ number and sex of donor (health status), tooth type, isolation method, passages used, cell density/confluency)	Flow type (steady laminar, pulsatile laminar, or oscillatory laminar)	FSS-duration and frequency (Hz)	FSS magnitude	FSS apparatus	Gene expression: increase, decrease, no change (method w/ reference gene); methods: (RT-qPCR, sqPCR)	Gene expression: when it reaches peak and peak's magnitude (fold change; relative gene expression; times or ratio; unclear = ?)	Protein expression: increase, decrease, no change (method w/reference); methods: ELISA, WB, RIA, EMSA, IF	Protein expression: when it reaches peak and peak's magnitude (times or ratio; unclear = ?)	Remarks including fluorescent microscopy assay
	Kraft et al. (2011)	Human Dental Pulp Cells (21y/ 1M (healthy), M, dig., P5, cell $4×10^5$ cells/n.g.)	Pulsatile laminar	1h FSS (0h, 1h, 3h post-FSS incubation)/5Hz	0.6Pa, a pulse amplitude of 0.3Pa and a peak shear stress rate of 8.4Pa/s	Custom-made	Post-FSS incubation: increase (RT-qPCR, GUS)	3h post-FSS: 5.0141 (FC)†			
BGLAP	Lim et al. (2013)	hABMSCs (n.g./ n.g. (n.g.), n.g., n.g., P3-5, n.g./n.g.)	Oscillatory laminar	5, 10, 30, 60, and 120min/d for 2w or 3w /n.g.	0.01-0.0205dyn/cm² (max. 0.00205Pa)	Rocking culture system (Vision Ltd.)	2w: increase (sqPCR, GAPDH)<br>3w: increase (sqPCR, GAPDH)	10min/d for 2w: 4.9 (rel.)†<br>60min/d for 3w: 1.7 (rel.)†			
	Li et al. (2004)	MSCs (BioWhittaker) (n.g./ n.g. (healthy), n.g., Ficoll paque, P2-8, n.g./n.g.)	Oscillatory laminar	2h FSS (24h post-FSS incubation)/1Hz	10dyn/cm²	Custom-made	Increase (RT-qPCR, GAPDH)	24h post-FSS: 1.4 (rel.)†			verified; Primer-BLAST (primer) and BLAST (probe)
	Lim et al. (2014)	hABMSCs (n.g./ n.g. (n.g.), n.g., n.g., n.g., 10^4 cells per cm²/n.g.)	Oscillatory laminar	10d @ 10, 30, 60, 120, 180min/d / n.g.	0.86–1.51dyn/cm²	Rotational orbital shaker	Temporary decrease then increase (sqPCR, GAPDH)	10min/d: 1.0 (rel.)†<br>30min/d: 1.9 (rel.)†			
	Hu et al. (2017)	Bone marrow cells, posterior iliac crests (MSCs) (n.g./ n.g. (healthy), n.g., n.g., P3-5, n.g./n.g.)	Steady laminar	1h, 3h, 6h, 12h, 24h, 72h/n.g.	12dyn/cm²	FlexFlo™ chamber (FlexCell Int.)	Increase, temporary decrease then increase (RT-qPCR; GAPDH)	1h: 1.2/1 = 1.2 (ratio-calc)†<br>3h: 1.1/1.2 = 0.91 (ratio-calc)†<br>6h: 1.3/1.2 = 1.1 (ratio-calc)†			
	Sonam et al. (2016)	hMSC (Lonza) (n.g./ n.g. (n.g.), n.g., n.g., P1-5, 1000 cells per cm² /n.g.)	Steady laminar	48h/n.g.	1Pa	Custom-made (planar topography)	Increase (RT-qPCR; GAPDH)	48h: 3.1 (rel.)†	Increase (IF)	48h: 2.6 (ratio)†	
Calcium (Ca^{2+})	Li et al. (2004)	MSCs (BioWhittaker) (n.g./ n.g. (healthy), n.g., Ficoll paque, P2-8, n.g./n.g.)	Oscillatory laminar	2h FSS (24h post-FSS incubation)/1Hz	10dyn/cm²	Custom-made			Fluctuated increase (fura-2 microscopy)		
	Riddle et al. (2007)	Human BMSCs (Cambrex Biosciences) (18y/ 1M (n.g.), n.g., n.g., P3-8, 130,000 cells or 80,000 cells/n.g.)	Oscillatory laminar	180s/1Hz	5dyn/cm²,<br>10dyn/cm²,<br>20dyn/cm²	Custom-made			Fluctuated increase (Fura-2 microscopy)		
	Salvi et al. (2010)	Human mesenchymal stem cells (hMSCs, Cambrex PT-2501) (n.g./ n.g.(n.g.), n.g., n.g., n.g., $4×10^3$ cells per cm²/n.g.)	Oscillatory laminar	180s/1Hz	5dyn/cm²,<br>10dyn/cm²,<br>20dyn/cm²	n.g.			Fluctuated increase (Fluorescence microscopy, Fura Red-AM dye)		
	Riddle et al. (2006)	hMSCs (Cambrex Biosciences) (18y/ 1M (n.g.), n.g., n.g., n.g., n.g./n.g.)	Oscillatory laminar	180s/1Hz	20dyn/cm²	Custom-made			Fluctuated increase (fura-2 microscopy)		
	Lee et al. (2017)	Bone marrow MSCs (n.g./ n.g. (n.g.), n.g., Ficoll paque, P1, $3×10^6$ cells per ml / 80%)	Steady laminar	70s/n.g.	15dyn/cm²	μ-slide $VI^{0.4}$ (Ibidi, DE) w/ fibronectin coating; 12-roller peristaltic pump (REGLO analog MS4/12, Ismatec)			Fluctuated increase (Fluo-4 AM microscopy)		

Official gene symbol or metabolite abbreviation	Reference	Cell type (age/ number and sex of donor (health status), tooth type, isolation method, passages used, cell density/confluency)	Flow type (steady laminar, pulsatile laminar, or oscillatory laminar)	FSS-duration and frequency (Hz)	FSS magnitude	FSS apparatus	Gene expression: increase, decrease, no change (method w/ reference gene); methods: (RT-qPCR, sqPCR)	Gene expression: when it reaches peak and peak's magnitude (fold change; relative gene expression; times or ratio; unclear = ?)	Protein expression: increase, decrease, no change (method w/reference); methods: ELISA, WB, RIA, EMSA, IF	Protein expression: when it reaches peak and peak's magnitude (times or ratio; unclear = ?)	Remarks including fluorescent microscopy assay
	Hu et al. (2017)	Bone marrow cells, posterior iliac crests (MSCs) (n.g./ n.g. (healthy), n.g., n.g., P3-5, n.g./n.g.)	Steady laminar	120s FSS/n.g.	12dyn/cm^2	FlexFlo™ chamber (FlexCell Int.)			Fluctuated increase (fura-2 microscopy)		
RUNX2	Li et al. (2004)	MSCs (BioWhittaker) (n.g./ n.g. (healthy), n.g., Ficoll paque, P2-8, n.g./n.g.)	Oscillatory laminar	2h FSS (24h post-FSS)/1Hz	10dyn/cm^2	Custom-made	Decrease (RT-qPCR, GAPDH)	24h post-FSS: 0.9 (rel.)†			
	Lim et al. (2013)	hABMSCs (n.g./ n.g. (n.g.), n.g., n.g., P3-5, n.g./n.g.)	Oscillatory laminar	5, 10, 30, 60, and 120min/d for 2w or 3w / n.g.	0.01-0.0205dyn/cm^2 (max. 0.00205Pa)	Rocking culture system (Vision Ltd.)	2w: increase followed by plateau (sqPCR, GAPDH)<br>3w: increase then Decrease (sqPCR, GAPDH)	5min/d for 2w: 2.7 (rel.)†<br>10min/d for 2w: 3 (rel.)†<br>30min/d for 2w: 3.1 (rel.)†<br>5min/d for 3w: 1.3 (rel.)†<br>120min/d for 3w: 0.7 (rel.)†			
	Hoey et al. (2012)	MSCs harvested from human bone marrow (Lonza, Walkersville, MD, USA, http://www.lonza. com) (n.g./ n.g. (n.g.), n.g., n.g., P4, n.g./n.g.)	Oscillatory laminar	2h (sampling 30min, 2h, 24h, 48h post-FSS)/n.g.	1.0Pa (28ml/min)	Custom-made	Decrease then increase with plateau (RT-qPCR; GAPDH)	2h post-FSS: 0.3/0.4 = 0.75 (ratio--calc)†<br>24h post-FSS: 0.8/0.7 = 1.14 (ratio-calc)†			
	Kuo et al. (2015)	human bone marrow-derived MSCs (Lonza) (21y/1F (n.g.), n.g., n.g., P4-5, 6,000 cells per cm/n.g.)	Oscillatory laminar	1h 0.5h,1h, 2h, 4h, 24h /1Hz (60/min)	0.5d±4dyn/cm^2	Custom-made	Increase (RT-qPCR; GAPDH)	2h: 3 (rel.)†			
	Lim et al. (2014)	hABMSCs (n.g./ n.g. (n.g.), n.g., n.g., n.g., 10^4 cells per cm^2/n.g.)	Oscillatory laminar	10d @ 10, 30, 60, 120, 180min/d / n.g.	0.86–1.51dyn/cm^2	Rotational orbital shaker	Decrease with plateau then increase (sqPCR, GAPDH)	30min/d: 0.6 (rel.)†<br>180min/d: 1.3 (rel.)†			
	Becquart et al. (2016)	Human MSCs (n.g./ 1 and 6 n.g. (n.g.), n.g., Ficoll paque, P2, cell n.g./n.g.)	Steady laminar	30min (sampling 0h, 1h, 6h post-FSS) / 2.8Hz	0.7Pa	µ-Slide w/ Ibidi pump system (Ibidi, DE)	Increase (RT-qPCR, 18S)	0h post-FSS: 1.9 (FC)†			
	Sonam et al. (2016)	hMSC (Lonza) (n.g./ n.g. (n.g.), n.g., n.g., P1-5, 1000 cells per cm^2 /n.g.)	Steady laminar	48h/n.g.	1Pa	Custom-made (planar topography)	Increase (RT-qPCR; GAPDH)	48h: 2.9 (rel.)†	Increase (IF)	48h: 3.1 (ratio)†	
COL1A1	Li et al. (2004)	MSCs (BioWhittaker) (n.g./ n.g. (healthy), n.g., Ficoll paque, P2-8, n.g./n.g.)	Oscillatory laminar	2h FSS (24h post-FSS incubation)/1Hz	10dyn/cm^2	Custom-made	Increase (RT-qPCR, GAPDH)	24h post-FSS: 1.1 (rel.)†			
	Lim et al. (2013)	hABMSCs (n.g./ n.g. (n.g.), n.g., n.g., P3-5, n.g./n.g.)	Oscillatory laminar	5, 10, 30, 60, and 120min/d for 2w or 3w / n.g.	0.01-0.0205dyn/cm^2 (max. 0.00205Pa)	Rocking culture system (Vision Ltd.)	2w: increase followed by plateau (sqPCR, GAPDH)<br>3w: increase followed by plateau (sqPCR, GAPDH)	30min/d for 2w: 1.55 (rel.) †<br>120min/d for 2w: 1.6 (rel.)†<br>30min/d for 3w: 1.21 (rel.) †<br>120min/d for 3w: 1.3 (rel.)†			
	Lim et al. (2014)	hABMSCs (n.g./ n.g. (n.g.), n.g., n.g., n.g., 10^4 cells per cm^2/n.g.)	Oscillatory laminar	10d @ 10, 30, 60, 120, 180min/d / n.g.	0.86–1.51dyn/cm^2	Rotational orbital shaker	Decrease then increase (sqPCR, GAPDH)	10min/d: 0.6 (rel.)†<br>180min/d: 1.3 (rel.)†			
Nitric oxide	Becquart et al. (2016)	Human MSCs (n.g./ 1 and 6 n.g. (n.g.), n.g., Ficoll paque, P2, cell n.g./n.g.)	Steady laminar	30min (sampling 0h, 1h, 6h post-FSS) / 2.8Hz	0.1Pa, 0.7Pa, 2.1Pa, 4.2Pa	µ-Slide w/ Ibidi pump system (Ibidi, DE)			Increase (Griess, NO_2^- + colorimetric assay)	0.7Pa @ 0h post-FSS: 24.5µM; 24.5/1.6 = 15.31 (ratio-calc)†	

Official gene symbol or metabolite abbreviation	Reference	Cell type (age/ number and sex of donor (health status), tooth type, isolation method, passages used, cell density/confluency)	Flow type (steady laminar, pulsatile laminar, or oscillatory laminar)	FSS-duration and frequency (Hz)	FSS magnitude	FSS apparatus	Gene expression: increase, decrease, no change (method w/ reference gene); methods: (RT-qPCR, sqPCR)	Gene expression: when it reaches peak and peak's magnitude (fold change; relative gene expression; times or ratio; unclear = ?)	Protein expression: increase, decrease, no change (method w/reference); methods: ELISA, WB, RIA, EMSA, IF	Protein expression: when it reaches peak and peak's magnitude (times or ratio; unclear = ?)	Remarks including fluorescent microscopy assay
	Kraft et al. (2011)	Human Dental Pulp Cells (21y/ 1M (healthy), M, dig., P5, cell 4×10^5 cells/n.g.)	Pulsatile laminar	1h FSS (1h, 3h post-FSS incubation)/5Hz	0.6Pa, a pulse amplitude of 0.3Pa and a peak shear stress rate of 8.4Pa/s	Custom-made			Decrease then increase (Griess, NO_2^-)	1h post-FSS: 0.78195 (ratio)†<br>3h post-FSS: 1.53383 (ratio)†	
	Kraft et al. (2011)	Human Dental Pulp Cells (21y/ 1M (healthy), M, dig., P5, cell 4×10^5 cells/n.g.)	Pulsatile laminar	5min, 10min, 15min, 30min, 60min FSS/5Hz	0.6Pa, a pulse amplitude of 0.3Pa and a peak shear stress rate of 8.4Pa/s	Custom-made			Increase (Griess, NO_2^-)	60min FSS: 3.7313 (ratio)†	
	Kraft et al. (2010)	human dental pulp-derived mesenchymal stem cells (PDSCs) (20y / 1M (healthy), M, dig., P2-11 and P4-23, 4×10^5 cells/n.g.) "mature"	Pulsating laminar	1h FSS (0min, 5min, 10min, 15min, 30min, 60min, 3h post-FSS incubation)/5Hz	0.6Pa, a pulse amplitude of 0.3Pa and a peak shear stress rate of 8.4Pa/s.	Custom-made			0h post-FSS: increase (Griess, NO_2^-)<br>Post-FSS: decrease then increase (Griess, NO_2^-)	0h post-FSS: 17.1nmol; 17.1/4.5 = 3.8 (ratio-calc)†<br>1h post-FSS: 1.8nmol; 1.8/2.3 = 0.78 (ratio-calc)†<br>3h post-FSS: 5.4nmol; 5.4/3.5 = 1.54 (ratio-calc)†	"mature" phenotype;
	Kraft et al. (2010)	human dental pulp-derived mesenchymal stem cells (PDSCs) (21y / 1F (healthy), M, dig., P2-11 and P4-23, 4×10^5 cells/n.g.) "immature"	Pulsating laminar	1h FSS (0min, 5min, 10min, 15min, 30min, 60min, 3h post-FSS incubation)/5Hz	0.6Pa, a pulse amplitude of 0.3Pa and a peak shear stress rate of 8.4Pa/s.	Custom-made			0h post-FSS: increase (Griess, NO_2^-)<br>Post-FSS: decrease then increase (Griess, NO_2^-)	0h post-FSS: 12.2nmol; 12.2/2.3 = 5.31 (ratio-calc)†<br>1h post-FSS: 1.8nmol; 1.8/2.4 = 0.75 (ratio-calc)†<br>3h post-FSS: 2.9nmol; 2.9/2.5 = 1.16 (ratio-calc)†	"immature" phenotype
SPP1	Li et al. (2004)	MSCs (BioWhittaker) (n.g./ n.g. (healthy), n.g., Ficoll paque, P2-8, n.g./n.g.)	Oscillatory laminar	2h FSS (24h post-FSS incubation)/1Hz	10dyn/cm^2	Custom-made	Increase (RT-qPCR, GAPDH)	24h post-FSS: 1.6 (rel.)†			
	Lim et al. (2013)	hABMSCs (n.g./ n.g. (n.g.), n.g., n.g., P3-5, n.g./n.g.)	Oscillatory laminar	5, 10, 30, 60, and 120min/d for 2w or 3w/n.g.	0.01-0.0205dyn/cm^2 (max. 0.00205Pa)	Rocking culture system (Vision Ltd.)	2w: increase (sqPCR, GAPDH)<br>3w: increase followed by plateau (sqPCR, GAPDH)	30min/d for 2w: 6.0 (rel.)†<br>30min/d for 3w: 1.26 (rel.)†<br>600min/d for 3w: 1.22 (rel.)†<br>120min/d for 3w: 1.2 (rel.)†			
	Hoey et al. (2012)	MSCs harvested from human bone marrow (Lonza, Walkersville, MD, USA, http://www.lonza. com) (n.g./ n.g. (n.g.), n.g., n.g., P4, n.g./n.g.)	Oscillatory laminar	2h (sampling 30min, 2h, 24h, 48h post-FSS)/n.g.	1.0Pa (28ml/min)	Custom-made	Increase then baseline (RT-qPCR, GAPDH)	30min post-FSS: 0.4/0.3 = 1.3 (ratio-calc)†<br>24h post-FSS: 1 (ratio-calc)†			
	Lim et al. (2014)	hABMSCs (n.g./ n.g. (n.g.), n.g., n.g., n.g., 10^4 cells per cm^2/n.g.)	Oscillatory laminar	10d @ 10, 30, 60, 120, 180min/d / n.g.	0.86–1.51dyn/cm^2	Rotational orbital shaker	Increase, decrease then increase (sqPCR, GAPDH)	30min/d: 2.3 (rel.)†<br>60min/d: 0.8 (rel.)†<br>180min/d: 1.9 (rel.)†			
	Yourek et al. (2010)	Isolated bone marrow hMSCs (20-26y/7M and 1F (Healthy), n.g., Ficoll paque, P3-5, 7.6–8.6×10^3 cells per cm^2/n.g.) ("hMSC")	Steady laminar	24h (after 3d or 7d pre-culture in basal medium)/n.g.	4dyn/cm^2, 15dyn/cm^2, 22dyn/cm^2	Custom-made	4dyn/cm^2: increase (RT-qPCR, GAPDH)<br>15dyn/cm^2: increase (RT-qPCR, GAPDH)<br>22dyn/cm^2: increase (RT-qPCR, GAPDH)	4dyn/cm^2 @ 7d pre-FSS: 8.3 (FC)†<br>15dyn/cm^2 @ 7d pre-FSS: 9.6(FC)†<br>22dyn/cm^2 @ 7d pre-FSS: 9.5(FC)†			

Official gene symbol or metabolite abbreviation	Reference	Cell type (age/ number and sex of donor (health status), tooth type, isolation method, passages used, cell density/confluency)	Flow type (steady laminar, pulsatile laminar, or oscillatory laminar)	FSS-duration and frequency (Hz)	FSS magnitude	FSS apparatus	Gene expression: increase, decrease, no change (method w/ reference gene); methods: (RT-qPCR, sqPCR)	Gene expression: when it reaches peak and peak's magnitude (fold change; relative gene expression; times or ratio; unclear = ?)	Protein expression: increase, decrease, no change (method w/reference); methods: ELISA, WB, RIA, EMSA, IF	Protein expression: when it reaches peak and peak's magnitude (times or ratio; unclear = ?)	Remarks including fluorescent microscopy assay
	Sonam et al. (2016)	hMSC (Lonza) (n.g./ n.g. (n.g.), n.g., n.g., P1-5, 1000 cells per cm^2 /n.g.)	Steady laminar	48h/n.g.	1Pa	Custom-made (planar topography)	Increase (RT-qPCR; GAPDH)	48h: 3.4 (rel.)†	Increase (IF)	48h: 2.4 (ratio)†	
VEGFA	Lim et al. (2013)	hABMSCs (n.g./ n.g. (n.g.), n.g., n.g., P3-5, n.g./n.g.)	Oscillatory laminar	5, 10, 30, 60, and 120min/d / n.g.	0.01-0.0205dyn/cm^2 (max. 0.00205Pa)	Rocking culture system (Vision Ltd.)			Increase (antibody array)	Time not mentioned	p.142/c
	Lim et al. (2014)	hABMSCs (n.g./ n.g. (n.g.), n.g., n.g., n.g., 10^4 cells per cm^2/n.g.)	Oscillatory laminar	10d @ 10, 30, 60, 120, 180min/d / n.g.	0.86–1.51dyn/cm^2	Rotational orbital shaker			Increase (ELISA)	60min/d: 4193.2pg/mL; 4193.2/2537.2 = 1.65 (ratio-calc)†	
	Lim et al. (2013)	hABMSCs (n.g./ n.g. (n.g.), n.g., n.g., P3-5, n.g./n.g.)	Oscillatory laminar	5, 10, 30, 60, and 120min/d / n.g.	0.01-0.0205dyn/cm^2 (max. 0.00205Pa)	Rocking culture system (Vision Ltd.)			Increase with plateau (ELISA)	5min/d for 24h: 2845.3 pg/mL; 2845.3/2380.6 = 1.2 (ratio-calc)†<br>10min/d for 24h: 2689 pg/mL; 2689/2380.6 =1.12 (ratio-calc)†<br>30min/d for 24h: 2845.3 pg/mL; 2845.3/2380.6 = 1.2 (ratio-calc)†<br>60min/d for 24h: 2700 pg/mL; 2700/2380.6 = 1.13 (ratio-calc) †	
	Becquart et al. (2016)	Human MSCs (n.g./ 1 and 6 n.g. (n.g.), n.g., Ficoll paque, P2, cell n.g./n.g.)	Steady laminar	30min (sampling 0h, 1h, 6h post-FSS) / 2.8Hz	0.7Pa	µ-Slide w/ Ibidi pump system (Ibidi, DE)	Increase (RT-qPCR, 18S)	1h post-FSS: 14 (FC)†			
	Charoenpong et al. (2019)	Human Pulpal Stem Cells (HDPCs) (n.g./ n.g. (n.g.), M, Ficoll paque, P3-6, n.g./n.g.)	Steady laminar	2h, 6h, 16h/n.g.	0.1Pa, 0.2Pa	Custom-made	Increase (RT-qPCR, GAPDH)	0.2 Pa @ 2h: 1.5 (rel.)†			
MAPK3; MAPK1	Riddle et al. (2006)	hMSCs (Cambrex Biosciences) (18y/ 1M (n.g.), n.g., n.g., n.g., n.g./n.g.)	Oscillatory laminar	15-120min/1Hz	20dyn/cm^2	Custom-made			1min, 5min, 15min, 30min, 60min: increase (WB, total ERK)	No quantitative information given.	
	Yuan et al. (2012)	hMSCs (Lonza) (n.g./ n.g. (n.g.), n.g., n.g., P4-8, n.g./n.g.)	Steady laminar	10min, 30min, 60min, 120min/n.g.	2Pa	Custom-made			Temporary decrease (WB, β-actin)	10min: 0.7/1.1 = 0.64 (ratio-calc)*<br>30min: 0.75/1.1 = 0.68 (ratio-calc)*	
	Yuan et al. (2012)	hMSCs (Lonza) (n.g./ n.g. (n.g.), n.g., n.g., P4-8, n.g./n.g.)	Steady laminar	10min, 30min, 60min, 120min/n.g.	0.2Pa	Custom-made			Temporary decrease (WB, β-actin)	0.2Pa @ 30min: 1.1/1.35 = 0.81 (ratio-calc)*	
	Yuan et al. (2012)	hMSCs (Lonza) (n.g./ n.g. (n.g.), n.g., n.g., P4-8, n.g./n.g.)	Steady laminar	10min, 30min, 60min, 120min/n.g.	0.2Pa	Custom-made			Increase (WB, β-actin)	0.2Pa @ 60min: 1.3/0.2 = 6.5 (ratio-calc)*	
	Becquart et al. (2016)	Human MSCs (n.g./ 1 and 6 n.g. (n.g.), n.g., Ficoll paque, P2, cell n.g./n.g.)	Steady laminar	30min (sampling 0h, 1h, 6h post-FSS) / 2.8Hz	0.7Pa	µ-Slide w/ Ibidi pump system (Ibidi, DE)			Increase (WB, β-tubulin)	0.7Pa @ 0h post-FSS: 9.5 (ratio)†	

Official gene symbol or metabolite abbreviation	Reference	Cell type (age/ number and sex of donor (health status), tooth type, isolation method, passages used, cell density/confluency)	Flow type (steady laminar, pulsatile laminar, or oscillatory laminar)	FSS-duration and frequency (Hz)	FSS magnitude	FSS apparatus	Gene expression: increase, decrease, no change (method w/ reference gene); methods: (RT-qPCR, sqPCR)	Gene expression: when it reaches peak and peak's magnitude (fold change; relative gene expression; times or ratio; unclear = ?)	Protein expression: increase, decrease, no change (method w/reference); methods: ELISA, WB, RIA, EMSA, IF	Protein expression: when it reaches peak and peak's magnitude (times or ratio; unclear = ?)	Remarks including fluorescent microscopy assay
	Lee et al. (2017)	Bone marrow MSCs (n.g./ n.g. (n.g.), n.g., Ficoll paque, P1, 3×10^6 cells per ml / 80%)	Steady laminar	5min, 1h, 3h / n.g.	15dyn/cm^2	µ-slide VI$^{0.4}$ (Ibidi, DE) w/ fibronectin coating; 12-roller peristaltic pump (REGLO analog MS4/12, Ismatec)			p-ERK @ 5min: increase (WB, ERK)	p-ERK/ERK @ 5 min: 3.4 (ratio)†	

5.2 Human osteoblasts

Official gene symbol or metabolite abbreviation	Reference	Cell Type (age and sex)/number and sex of donor (health status), tooth type, isolation method, passages used, cell density / confluency)	Flow type (steady laminar, pulsatile laminar, or oscillatory laminar)	FSS duration and frequency	FSS magnitude	FSS apparatus	Gene expression: Increase, decrease, no change (method w/ reference gene); methods: RT-qPCR, sqPCR	Gene expression: when it reaches peak and peak's magnitude (fold change; relative gene expression; times or ratio; unclear = ?)	Protein expression: Increase, decrease, no change (method w/ reference); methods: ELISA, WB, RIA, EMSA, IF	Protein expression: When it reaches peak and peak's magnitude (times or ratio; unclear = ?)	Remarks including fluorescent microscopy assay
Nitric oxide	McGarry et al. (2005)	Human bone fragments ((14–16y males and 11y female)/2M (n.g) + 1F (n.g), n.g., exp., P (2+), 5×10^5 cells per cm^2 / n.g.)	Pulsatile laminar	1h @ 5Hz	0.6±0.3Pa	Custom-made			Increase (Griess, NO2-)	1h: 7.1 (ratio)	
	Sterck et al. (1998)	Human transiliac bone biopsies ((7–77y males and females)/10M (Healthy)+7F(Healthy), n.g., dig., n.g., 25×10^3 cells per cm^2/n.g.)	Pulsatile laminar	1h (sampling 0h, 24h post-PFF) @ 5Hz	SS of 0.7±0.03Pa, peak stress rate of 12.2Pa/s	Custom-made			Increase (Griess, NO_2^-)	0h post-PFF: 2.3 (ratio)<br>24h post-PFF: 1.01 (ratio-calc)	
	van der Meijden et al. (2016)	Human trabecular bone (maxilla/mandible) samples ((35.1± 5.7y males and females)/2M(healthy), 9F(Healthy), n.g., exp., n.g., 5×10^5 cells per cm^2/ n.g.)	Pulsatile laminar	1h (sampling 0h, 3h post-PFF) / 5Hz	0.7Pa	Custom-made			Increase (Griess, NO_2^-)	0h post-PFF: 34.3nmol; 34.3/2.3 = 14.9 (ratio-calc)†	
	Santos et al. (2011)	Human trabecular bone samples ((18-84y males and females)/2M(n.g.) and 7F(n.g.), n.g, dig., P2, 5×10^3 cells per cm^2/ n.g.)	Pulsatile laminar	5min / n.g.	0.7±0.3Pa	Custom-made			Increase (Griess, NO_2^-)	16.8 nmol; 16.8/5.1 = 3.2 (ratio-calc)†	
	Klein-Nulend et al. (1998)	Human Transiliac bone biopsies ((7-90y, n.g.)/18, n.g. (healthy), n.g., dig., n.g., 5×10^5 cells per cm^2/ n.g.)	Pulsatile laminar	0min, 4min, 8min, 15min, 30min, 60min @ 5Hz	0.7±0.03Pa (12.2Pa/s)	Custom-made			Increase (Griess, NO_2^-)	30min: 423.8nmol/mg protein; 423.8/63.8 = 6.6 (ratio-calc)†	
	Klein-Nulend et al. (1998)	Human Transiliac bone biopsies ((7-90y, n.g.)/18, n.g. (healthy), n.g., dig., n.g., 5×10^5 cells per cm^2/ n.g.)	Pulsatile laminar	1h (sampling 0h, 1h, 24h post-PFF) @ 5Hz	0.7±0.03Pa (12.2Pa/s)	Custom-made			Increase (Griess, NO_2^-)	0h post-PFF: 734.8nmol/mg protein; 734.8/323.1 = 2.3 (ratio-calc)†<br>1h post-PFF: 70.9nmol/mg protein; 70.9/62.4 = 1.14 (ratio-calc)†<br>24h post-PFF: 358.6nmol/mg protein; 358.6/319.9 = 1.12 (ratio-calc)†	
PGE2	Joldersma et al. (2001)	Transiliac bone biopsies ((56-75y females)/7F(Healthy), n.g., dig., n.g., 25×10^3 cells per cm^2/n.g.)	Pulsatile laminar (roller pump)	1h @ 5Hz	0.6±0.3Pa, peak stress rate 8.4Pa/s	Custom-made			Mean increase (EIA)	1h: 1504.1pg/µgDNA; 1504.1/491.7 = 3 (ratio-calc)†	
	Klein-Nulend et al. (2002)	Iliac crest bone biopsies ((7-85y males and females)/22M(healthy) and 17F(healthy), n.g., dig., P2, 5×10^5 cells per cm^2/n.g.)	Pulsating laminar	1h (sampling 0h, 1h, 24h post-PFF) @ 5Hz	Mean SS 0.7±0.3Pa, peak stress rate of 9.5Pa/s	Custom-made			Increase (EIA)	0h post-PFF: 178.4ng/mg protein; 178.4/70.9 = 2.5 (ratio-calc)†<br>1h post-PFF: 24.2/16.4 = 1.4 (ratio-calc)<br>24h post-PFF: 106.8/54.4 =1.9 (ratio-calc)	

Official gene symbol or metabolite abbreviation	Reference	Cell Type (age and sex)/number and sex of donor (health status), tooth type, isolation method, passages used, cell density / confluency)	Flow type (steady laminar, pulsatile laminar, or oscillatory laminar)	FSS duration and frequency	FSS magnitude	FSS apparatus	Gene expression: Increase, decrease, no change (method w/ reference gene); methods: RT-qPCR, sqPCR	Gene expression: when it reaches peak and peak's magnitude (fold change; relative gene expression; times or ratio; unclear = ?)	Protein expression: Increase, decrease, no change (method w/ reference); methods: ELISA, WB, RIA, EMSA, IF	Protein expression: When it reaches peak and peak's magnitude (times or ratio; unclear = ?)	Remarks including fluorescent microscopy assay
	McGarry et al. (2005)	Human bone fragments ((14–16y males and 11y female)/2M (n.g) + 1F (n,g), n.g., exp., P (2+), 5×10^5 cells per cm^2 / n.g.)	Pulsatile laminar	1h @ 5Hz	0.6±0.3Pa	Custom-made			Increase (ELISA)	1h: 3.3 (ratio)	
	Sterck et al. (1998)	Human transiliac bone biopsies ((7–77y males and females)/10M (Healthy)+7F(Healthy), n.g., dig., n.g., 25×10^3 cells per cm^2/n.g.)	Pulsatile laminar	1h (sampling 0h, 24h post-PFF) @ 5Hz	SS of 0.7±0.03Pa, peak stress rate of 12.2Pa/s	Custom-made			Increase (ELISA)	0h post-PFF: 1.8 (ratio)<br>24h post-PFF: 1.9 (ratio)	
	Bakker et al. (2003b)	Primary human bone cells ((54–84y females)/ 9F (healthy), n.g., dig., n.g., 5×10^5 cells per slide /n.g.)	Pulsatile laminar	1h (sampling 0h, 24h post-PFF) @ 5Hz	0.6±0.3Pa mean SS	Custom-made			Increase (ELISA)	0h post-PFF: 2.3 ng/5×10^5 cells; 2.3/1.2 = 2.76 (ratio-calc)†<br>24h post-PFF: 0.96 ng/5×10^5 cells; 0.96/0.6 = 1.6 (ratio-calc)†	
	Joldersma et al. (2000)	Human Transiliac bone biopsies ((56-80y females)/9F(Healthy), n.g., dig., n.g., 25×10^3 cells per cm^2/n.g.)	Pulsatile laminar	1h @ 5Hz	FSS of 0.7±0.02Pa, peak stress rate of 12.2Pa/s	Custom-made			Mean increase (EIA)	3897.4 pg/µgDNA; 3897.4/1107.7 = 3.52 (ratio-calc)†	Data of 9 individuals also reported.
PGI2	Joldersma et al. (2001)	Transiliac bone biopsies ((56-75y females)/7F(Healthy), n.g., dig., n.g., 25×10^3 cells per cm^2/ n.g.)	Pulsatile laminar (roller pump)	1h @ 5Hz	0.6±0.3Pa, peak stress rate 8.4Pa/s	Custom-made			Mean increase (EIA)	1h: 320.2pg/µgDNA; 320.2/155.3 = 2 (ratio-calc)†	
	Klein-Nulend et al. (2002)	Iliac crest bone biopsies ((7-85y males and females)/22M(healthy) and 17F(healthy), n.g., dig., P2, 5×10^5 cells per cm^2/ n.g.)	Pulsating laminar	1h (sampling 0h, 1h, 24h post-PFF) @ 5Hz	Mean SS 0.7±0.3Pa, peak stress rate of 9.5Pa/s	Custom-made			Increase (EIA)	0h post-PFF: 10.6ng/mg protein; 10.6/6.5 1.6 (ratio-calc)†<br>1h post-PFF: 0.8/0.6 = 1.3 (ratio-calc)<br>24h post-PFF: 2.9/1.7 = 1.7 (ratio-calc)	
	Joldersma et al. (2000)	Human transiliac bone biopsies ((56-80y females)/9F(Healthy), n.g., dig., n.g., 25×10^3 cells per cm^2/ n.g.)	Pulsatile laminar	1h @ 5Hz	FSS of 0.7±0.02Pa, peak stress rate of 12.2Pa/s	Custom-made			Mean increase (EIA)	1110.2pg/µgDNA; 1110.2/302 = 3.68 (ratio-calc)†	Data of 9 individuals also reported.
PTGS1	Joldersma et al. (2001)	Transiliac bone biopsies ((56-75y females)/7F(Healthy), n.g., dig., n.g., 25×10^3 cells per cm^2/n.g.)	Pulsatile laminar (roller pump)	1h @ 5Hz	FSS: 0.6±0.3Pa, peak stress rate is 8.4Pa/s	Custom-made	Increase (sqPCR, GAPDH)	1h: 2.9 (rel.)†			
	Bakker et al. (2003b)	Primary human bone cells ((54–84y females)/ 9F (healthy), n.g., dig., n.g., 5×10^5 cells per slide /n.g.)	Pulsatile laminar	1h (sampling 0h, 24h post-PFF) @ 5Hz	0.6±0.3Pa mean SS	Custom-made	Mean increase (sqPCR, GAPDH)	0h post-PFF: 1.3 (rel.)†			
	Joldersma et al. (2000)	Human Transiliac bone biopsies ((56-80y females)/9F(Healthy), n.g., dig., n.g., 25×10^3 cells per cm^2/n.g.)	Pulsatile laminar	1h @ 5Hz	FSS of 0.7±0.02Pa, peak stress rate of 12.2Pa/s	Custom-made	Increase (sqPCR, GAPDH)	1.3 (rel.)†			Data of 9 individuals also reported.

Official gene symbol or metabolite abbreviation	Reference	Cell Type (age and sex)/number and sex of donor (health status), tooth type, isolation method, passages used, cell density / confluency)	Flow type (steady laminar, pulsatile laminar, or oscillatory laminar)	FSS duration and frequency	FSS magnitude	FSS apparatus	Gene expression: Increase, decrease, no change (method w/ reference gene); methods: RT-qPCR, sqPCR	Gene expression: when it reaches peak and peak's magnitude (fold change; relative gene expression; times or ratio; unclear = ?)	Protein expression: Increase, decrease, no change (method w/ reference); methods: ELISA, WB, RIA, EMSA, IF	Protein expression: When it reaches peak and peak's magnitude (times or ratio; unclear = ?)	Remarks including fluorescent microscopy assay
PTGS2	Joldersma et al. (2001)	Transiliac bone biopsies ((56-75y females)/7F(Healthy), n.g., dig., n.g., 25×10³ cells per cm²/n.g.)	Pulsatile laminar (roller pump)	1h @ 5Hz	0.6±0.3Pa, peak stress rate 8.4Pa/s	Custom-made	Increase (sqPCR, GAPDH)	1h: 1.3 (rel.)†			
	Bakker et al. (2003b)	Primary human bone cells ((54–84y females)/ 9F (healthy), n.g., dig., n.g., 5×10⁵ cells per slide /n.g.)	Pulsatile laminar	1h (sampling 0h, 24h post-PFF) @ 5Hz	0.6±0.3Pa mean SS	Custom-made	Mean increase (RT-qPCR, PBGD)	0h post-PFF: 3.8 (rel.)†<br>24h post-PFF: 4.97 (rel.)†			
	Joldersma et al. (2000)	Human Transiliac bone biopsies ((56-80y females)/9F(Healthy), n.g., dig., n.g., 25×10³ cells per cm²/n.g.)	Pulsatile laminar	1h @ 5Hz	FSS of 0.7±0.02Pa, peak stress rate of 12.2Pa/s	Custom-made	Increase (sqPCR, GAPDH)	2.9 (rel.)†			Data of 9 individuals also reported.

5.3 Human PDL cells

Official gene symbol or metabolite abbreviation	Reference	Cell Type (age/sex of donor, health status, tooth type, isolation method, passages used, cell density/confluency)	Flow type (steady laminar, pulsatile laminar, or oscillatory laminar)	FSS duration and frequency	FSS magnitude	FSS apparatus	Gene expression: Increase, decrease, no change (method w/ reference gene); Methods: (RT-qPCR, sqPCR)	Gene expression: when it reaches peak and peak's magnitude (fold change; relative gene expression; times or ratio; unclear = ?)	Protein expression: Increase, decrease, no change (method w/reference); Methods: ELISA, WB, RIA, EMSA, IF	Protein expression: When it reaches peak and peak's magnitude (times or ratio; unclear = ?)	Remarks including fluorescent microscopy assay
ALP/ALPP	Tang et al. (2014)	hPDLCs (11–28y/3M+3F, Healthy, PM, exp., P3–6, 1×10⁵ cells per cm²/ 70-80% confluency)	Steady laminar	2h (sampling 0h, 6h, 12h, 24h, 48h post-FSS) / n.g.	12dyn/cm²	Custom-made	Temporary decrease followed by increase then decrease (RT-qPCR, GAPDH)	6h post-FSS: 0.8 (FC)†<br>12h post-FSS: 1.6 (FC)†<br>48h post-FSS: 0.95 (FC)†	Increase (ELISA)	24h post-FSS: 0.030U/mg; 0.030/0.019 = 1.6 (ratio-calc)†	ALP activity with p-nitrophenol substrate.
	Zheng et al. (2016)	hPDLCs (12–28y/n.g., Healthy, M, dig., P3–6, n.g./ 90% confluency)	Steady laminar	2h, 4h, 8h, 12h/n.g.	6dyn/cm²	Custom-made	Increase (sqPCR, GAPDH)	8h: 1.6/0.56 = 2.9 (ratio-calc)†			Verified by Primer-BLAST.
	Qi and Zhang (2014)	hPDLCs (<25y/n.g., n.g., n.g., exp., n.g., n.g./70-80% confluency, immortalized) "I-PDL"	Steady laminar	6h/n.g.	3dyn/cm², 6dyn/cm², 9dyn/cm², 12dyn/cm², 15dyn/cm²	Custom-made	Increase (RT-qPCR, GAPDH)	3dyn: 1.7 (FC)†<br>6dyn: 2.3 (FC)†<br>9dyn: 3.4 (FC)†<br>12dyn: 4 (FC)†<br>15dyn: 5.3 (FC)†	15dyn: increase (WB, β-actin)	No quantitative information given.	Verified by Primer-BLAST

5.4 Mouse osteoblasts

Official gene symbol or metabolite abbreviation	Reference	Cell Type (age/number and sex of donor (health status), tooth type, isolation method, passages used, cell density/confluency)	Flow type (steady laminar, pulsatile laminar, or oscillatory laminar)	FSS duration and frequency	FSS magnitude	FSS apparatus	Gene expression: Increase, decrease, no change (method w/ reference gene); methods: RT-qPCR, sqPCR	Gene expression: when it reaches peak and peak's magnitude (fold change; relative gene expression; times or ratio; unclear = ?)	Protein expression: Increase, decrease, no change (method w/reference); methods: ELISA, WB, RIA, EMSA, IF	Protein expression: When it reaches peak and peak's magnitude (times or ratio; unclear = ?)	Remarks including fluorescent microscopy assay
Mapk3; Mapk1	Yang et al. (2010)	Calvarial osteoblasts (day 3–5 after birth/ 10 to 12 (C57BL/6J), n.g., dig., P2-4, 2×10^5/n.g.)	Oscillatory laminar	5min, 15min, 30min, 60min / 1Hz	12dyn/cm^2	Custom-made			Increase then decrease (WB, total-Erk1/2)	15min: 0.81/0.4 = 2.03 (ratio-calc)* 5min: 0.95/0.4 = 2.37 (ratio-calc)*	
	Xing et al. (2014)	Primary osteoblastic cells (6 to 12 weeks/ n.g. (n.g.), n.g., dig., n.g., n.g./n.g.)	Oscillatory laminar	5min, 15min @1Hz	10dyn/cm^2	n.g.			5min: increase (WB, total ERK)	No quantitative information given.	
	Castillo et al. (2014)	Primary mouse calvaria cells (3- to 5-day-old/ n.g. (WT), n.g., dig., P4-6, 2,500 per cm^2/80%)	Oscillatory laminar	30min @1Hz	15dyn/cm^2	Custom-made			Increase (WB, total ERK)	30 min: 4.5/1.1 = 4 (ratio-calc)†	BERKO: β-estrogen receptor knockout
	Mehrotra et al. (2006)	Primary osteoblastic calvariae cells (n.g./ n.g. (n.g.), n.g., dig., n.g., n.g./n.g.)	Steady laminar	0min, 5min, 15min, 30min, 60 / n.g.	10dyn/cm^2	Custom-made			5min FSS: increase (WB, ERK1/2)	No quantitative information is given	
	Kapur et al. (2010)	Primary calvariae osteoblasts (8,12 and 10-week-old / n.g. (C57BL/6), n.g. dig., P3-6, 50000 cells/~80%)	Steady laminar	30min/n.g.	20dyn/cm^2	Cytodyne flow chamber			30min: increase (WB, ERK1/2)	300/100= 3 (ratio-calc)*	Figure2B
	Lau et al. (2006)	Primary mouse calvarial osteoblasts (8-week-old / n.g. (C57BL/6), n.g., dig., P 3–6, 50,000 cells /~80%)	Steady laminar	30min/n.g.	20dyn/cm^2	Cytodyne flow chamber			Increase (WB, ERK1/2)	320/100 = 3.2 (ratio-calc)*	
Nitric oxide	Bakker et al. (2013a)	Mouse long-bone osteoblasts (8 and 28 weeks / n.g./M (C57BL/6J), n.g., dig., P3, 3×10^5 cells per glass slide/n.g.)	Pulsatile laminar	0, 5, 10, 15, 20, 25, 30min / 5Hz	0.7±0.3Pa	Custom-made			Increase with plateau (Griess, NO_2^-)	5min: 12.69nmole/3×10^5; 12.6/3.5 = 3.6 30min: 14.9nmole/3×10^5; 14.9/5.2 = 2.9 (ratio-calc)†	
	Bakker et al. (2003a)	Primary mouse bone cells (n.g./ n.g. (Swiss albino mice), n.g., dig., n.g., 15–30×10^4 / n.g.)	Pulsating laminar	5min, 30 min / 5Hz	0.6Pa (w/ 0.3Pa pulse amplitude, 8.4Pa/s peak stress rate)	Custom-made			Increase (Griess, NO_2^-)	5min: 110.4nmol/mg; 110.4/61.5 = 1.8 (ratio-calc)†	
	Bakker et al. (2013a)	Mouse calvaria-derived osteoblasts (n.g./ n.g.(C57BL/6J), n.g., dig., P3, 5×10^4 cells per mL /n.g.)	Pulsatile laminar	30min / 5Hz	0.7±0.3Pa	µ-Slide IV (Ibidi), collagen coated			Increase (DAR4 M-AM)	1.1/0.2 = 5.5 (production rate)	Huesa, Helfrich, Aspden (2010) Parallel-plate fluid flow systems for bone cell stimulation. J Biomech 43:1182–1189
	Bakker et al. (2001)	Primary Mouse long bone cells (n.g./ n.g. (pregnant Swiss albino mice), n.g., dig., n.g., 5×10^5 cells per glass slide/n.g.)	Pulsatile laminar	15min @ 5Hz	Mean SS: 0.64Pa Peak SS: 8.40Pa	Custom-made			Increase (Griess, NO_2^-)	15min @ 5Hz(0.64Pa): 13.0nM/5×10^5 cells; 13.0/0.4 = 32.5 (ratio-calc)†	
	Soejima et al. (2001)	Primary mouse long bone cells (adult/ n.g. (Swiss albino mice), n.g., dig., n.g., 5×10^5 cells per slide/n.g.)	Pulsatile laminar	30min @ 5Hz	FSS of 0.6±0.3Pa, estimated peak stress rate of 8.5Pa/s	Custom-made			Adult mouse long bone cells: increase (Griess, NO_2^-)	30min: 10.7nM/10^5 cells; 10.7/2.6 = 4.11 (ratio-calc)†	
	Soejima et al. (2001)	Primary mouse calvarial cells (adult/ n.g. (Swiss albino mice), n.g., dig., n.g., 5×10^5 cells per slide /n.g)	Pulsatile laminar	30min @ 5Hz	FSS of 0.6±0.3Pa, estimated peak stress rate of 8.5Pa/s	Custom-made			Adult mouse calavarial cells: increase followed by plateau (Griess, NO_2^-)	5min: 6nM/10^5 cells; 6/1 = 6 (ratio-calc)* 20-30 min: 4.8nM/10^5 cells; 4.8/1.5 = 3.2 (ratio-calc)†	
	Soejima et al. (2001)	Primary neonatal mouse calvarial cells (3-4days/ n.g., n.g., dig., n.g., 5×10^5 cells per slide /n.g)	Pulsatile laminar	30min @ 5Hz	FSS of 0.6±0.3Pa, estimated peak stress rate of 8.5Pa/s	Custom-made			Neonatal mouse calavarial cells: increase (Griess, NO_2^-)	15min: 19.4nM/10^5 cells; 19.4/9.9 = 1.95 (ratio-calc)†	
	Callewaert et al. (2010)	Primary mouse bone cells (n.g./ n.g. (WT), n.g., dig., n.g., 2 x10^5 cells per slide/n.g.)	Pulsating laminar	10min @ 5Hz	Mean SS of 0.6Pa, Pulse amplitude of 0.3Pa	n.g.			Increase (Griess, NO_2^-)	10 min: 0.77pmol/ng DNA; 0.77/0.26 = 2.96 (ratio-calc)†	No reference for chamber used given.
PGE2	Castillo et al. (2014)	Primary mouse calvaria cells (3- to 5-day-old/ n.g. (WT), n.g., dig., P4-6, 2,500 per cm^2/80%)	Oscillatory laminar	4h (sampling 1h post FSS) @1Hz	15dyn/cm^2	Custom-made			Increase (ELISA)	1h post FSS: 24.7ng/mg; 24.7/7.1 = 3.5 (ratio-calc)†	
	Li et al. (2005)	Calvarial osteoblasts (3–5-day-old/ n.g. (C57BL/6), n.g., dig., n.g., 2,000 cells per cm^2 / 90%)	Steady laminar	30min, 15–60min / n.g.	12dyn/cm^2	n.g.			Increase (ELISA)	60min: 37.4ng/mg; 37.4/5.1 = 7.3 (ratio-calc)†	

Official gene symbol or metabolite abbreviation	Reference	Cell Type (age/number and sex of donor (health status), tooth type, isolation method, passages used, cell density/confluency)	Flow type (steady laminar, pulsatile laminar, or oscillatory laminar)	FSS duration and frequency	FSS magnitude	FSS apparatus	Gene expression: Increase, decrease, no change (method w/ reference gene); methods: RT-qPCR, sqPCR	Gene expression: when it reaches peak and peak's magnitude (fold change; relative gene expression; times or ratio; unclear = ?)	Protein expression: Increase, decrease, no change (method w/reference); methods: ELISA, WB, RIA, EMSA, IF	Protein expression: When it reaches peak and peak's magnitude (times or ratio; unclear = ?)	Remarks including fluorescent microscopy assay
	Bakker et al. (2003a)	Primary mouse bone cells (n.g./ n.g. (Swiss albino mice), n.g., dig., n.g., 15–30×10^4 / n.g.)	Pulsating laminar	5min, 30 min / 5Hz	0.6Pa (w/ 0.3Pa pulse amplitude, 8.4Pa/s peak stress rate)	Custom-made			Increase (ELISA)	30min: 52.6ng/mg; 52.6/30.1 = 1.74 (ratio-calc)†	
	Thi et al. (2012)	Primary mouse osteoblasts (n.g./ n.g. (embryonic (E19–20) wild type), n.g., dig., n.g., 2×10^4 cells per cm^2/n.g.)	Pulsatile laminar	1h / 1Hz	10dyn/cm^2	Cytodyne (La Jolla, CA)			Increase (ELISA)	1h: 1.7pg/ug; 1.7/ 0.7 = 2.4 (ratio-calc)†	
	Bakker et al. (2001)	Primary Mouse long bone cells (n.g./ n.g. (pregnant Swiss albino mice), n.g., dig., n.g., 5×10^5 cells per glass slide/n.g.)	Pulsatile laminar	15min @ 9Hz	Mean SS:1.20 pa Peak SS: 20.90Pa	Custom-made			Increase (ELISA)	15min @ 9Hz(1.20Pa): 8.7ng/5×10^5cells; 8.7/3.1 = 2.8 (ratio-calc)†	
	Klein-Nulend et al. (1997)	Primary mouse calvarial cells (3- to 4-day-old/ n.g. (n.g.), n.g., dig., n.g., 5×10^5 cells per slide/n.g.)	Pulsatile laminar	1h (sampling 1h post FSS) @ 5Hz	FSS: 0.7±0.03Pa, Peak stress rate: 12Pa/s	Custom-made			Increase (ELISA)	1h post FSS: 553 pg; 553/111.8 = 4.9 (ratio-calc)†	
	Klein-Nulend et al. (1996)	Primary mouse calvarial bone cells (3-4 day old / n.g. (n.g.), n.g., dig., n.g., 25×10^3 cells per well /n.g.)	Pulsatile laminar	1h (sampling 1h post FSS) / 5Hz	FSS: 0.5±0.02Pa, and the peak stress rate 0.4Pa/s	Custom-made			Increase (ELISA)	1h post FSS: 30.4nM; 30.4/10.8 = 2.8 (ratio-calc)†	
	Bakker et al. (2003b)	Mouse long bone cells (n.g./ n.g. (adult Swiss albino mice), n.g., dig., n.g., 5×10^5 cells per slide/n.g.)	Pulsatile laminar	1h (sampling 0h, 24h post FSS) / 5Hz	Mean SS of 0.6Pa, pulse amplitude of 0.3Pa	Custom-made			Increase (EIA)	0h post FSS: 8.96ng/5×10^5 cells; 8.96/5.9 = 1.5 (ratio-calc)† 24h post FSS: 3.8ng/5×10^5 cells; 3.8/1.9 = 2 (ratio-calc)†	
Ptgs2	Castillo et al. (2014)	Primary mouse calvaria cells (3- to 5-day-old/ n.g. (WT), n.g., dig., P4-6, 2,500 per cm^2/80%)	Oscillatory laminar	4h (sampling 1h post FSS) @1Hz	15dyn/cm^2	Custom-made			Increase (WB, vinculin)	1h post FSS: 1.2/0.53 = 2.3 (ratio-calc)†	
	Lau et al. (2006)	Primary mouse calvarial osteoblasts (8-week-old / n.g. (C57BL/6), n.g., dig., P 3–6, 50,000 cells /~80%)	Steady laminar	30min/n.g.	20dyn/cm^2	Cytodyne flow chamber			increase (WB, actin)	350/100 = 3.5 (ratio-calc)*	
	Yang et al. (2015)	Primary mouse osteoblasts (2-3 day/ n.g. (BALB/c neonatal mice), n.g., dig., n.g., 1.5×10^5 per slide/n.g.)	Steady laminar	60min, 90min / n.g.	≈12dyn/cm^2	Custom-made			Increase (WB, GAPDH)	90min: 0.9/0.8 = 1.12 (ratio-calc)†	
	Mehrotra et al. (2006)	Primary osteoblastic calvariae cells (n.g./ n.g. (n.g.), n.g., dig., n.g., n.g./n.g.)	Steady laminar	1h (sampling 0h, 4h post FSS) / n.g.	10dyn/cm^2	Custom-made	4h post FSS: increase (Northern blot, GAPDH)	No quantitative information given.			
	Klein-Nulend et al. (1997)	Primary mouse calvarial cells (3- to 4-day-old/ n.g. (n.g.), n.g., dig., n.g., 5×10^5 cells per slide/n.g.)	Pulsatile laminar	1h (sampling 0h, 1h post FSS) @ 5Hz	FSS: 0.7±0.03Pa, Peak stress rate: 12Pa/s	Custom-made	0h post FSS: Increase (Northern blot, GAPDH) 1h post FSS: Increase (Northern blot, GAPDH)	0h post FSS: 700/100 = 7 (ratio-calc)* 1h post FSS: 1450/150 = 9.7 (ratio-calc)*	Increase (ELISA)	1h post FSS: 31.8 (nM PEG_2); 31.8/ 13.9 = 2.3 (ratio-calc)†	
	Callewaert et al. (2010)	Primary mouse bone cells (n.g./ n.g. (WT), n.g., dig., n.g., 2 x10^5 cells per slide/n.g.)	Pulsating laminar	1h @ 5Hz	Mean SS of 0.6Pa, Pulse amplitude of 0.3Pa	n.g.	Increase (RT-qPCR, HPRT)	1h: 3.14±1.72/0.42±0.17 = 7.5 (ratio-calc)			No reference for chamber used given.

5.5 Mouse osteocytes

Official gene symbol or metabolite abbreviation	Reference	Cell type (age/ number and sex of donor (health status), tooth type, isolation method, passages used, cell density/confluency)	Flow type (Steady laminar, Pulsatile laminar, or Oscillatory laminar)	FSS duration and frequency	FSS magnitude	FSS apparatus	Gene expression: Increase, decrease, no change (method w/ reference gene); methods: RT-qPCR, sqPCR, northern hybridization	Gene expression: when it reaches peak and peak's magnitude (fold change; relative gene expression; times or ratio; unclear = ?)	Protein expression: Increase, decrease, no change (method w/reference); methods: ELISA, WB, RIA, EMSA, IF	Protein expression: When it reaches peak and peak's magnitude (times or ratio; unclear = ?)	Remarks including fluorescent microscopy assay
Calcium (Ca^{2+})	Wang et al. (2019)	MLO-Y4 cells (n.g./ n.g. (n.g.), n.g., n.g., n.g., n.g./n.g.)	Oscillatory laminar	9min (sampling at 60s, 120s, 240s, 360s, 480s, 600s during FSS) @ 0.25Hz	2Pa	Custom-made			Fluctuated increase and decrease (fluorescence microscopy, Suramin)	High density:<br>Increase @ 119s: 1.77 ([Ca^{2+}] fold of baseline ratio)*<br>Decrease @ 238s: 0.95 ([Ca^{2+}] fold of baseline ratio)*	
	Deepak et al. (2017)	Murine MLO-Y4 osteocyte-like cells (n.g./ n.g. (n.g.), n.g., n.g., n.g., 4x10^4 cells per slide/n.g.)	Oscillatory laminar	1h @ 0.5Hz (24h post-PFF incubation)	1Pa	Custom-made			Fluctuated increase (fluorescence microscopy, Fura-2 AM)		
	Seref-Ferlengez et al. (2016)	Osteocytic MLO-Y4 (n.g./ n.g. (n.g.), n.g., n.g., n.g., n.g./n.g.)	Oscillatory Laminar	5min @ 1Hz	(OFSS, τ=±10dyn/cm²)	μ-slide VI$^{0.4}$ chamber (ibidi GmbH, Germany)			Fluctuated increase (fluorescence microscopy, Fura-2 AM)		
	Litzenberger et al. (2010)	MLO-Y4 parental cells (n.g./ n.g. (n.g.), n.g., n.g., n.g., n.g./n.g.)	Oscillatory laminar	2h @1Hz	1Pa	Custom-made			Increase (Florescence microscopy Fura-2 AM)	Increase: 68.95 (precent of cells exhibiting Ca^{2+} flux)†	MLO-Y4 "parental" cells was extracted as mentioned by Author
	Lu et al. (2012b)	Osteocyte-like MLO-Y4 cells (n.g./ n.g. (n.g.), n.g., n.g., n.g., n.g./70-80%)	Oscillatory laminar	9min (sampling at 60s, 120s, 240s, 360s, 480s, 600s during FSS) @ 1Hz	20dyn/cm²	Custom-made			Fluctuated increase (Fura-2 AM microscopy)	oscillatory laminar (87s): 2.65 ([Ca^{2+}] intensity of baseline ratio)†; Note: comparison between steady and oscillatory fluid flow	
	Wang et al. (2019)	MLO-Y4 cells (n.g./ n.g. (n.g.), n.g., n.g., n.g., n.g./n.g.)	Oscillatory laminar	9min (sampling at 60s, 120s, 240s, 360s, 480s, 600s during FSS) @ 0.25Hz	2Pa	Custom-made			Fluctuated increase and decrease (fluorescence microscopy, Suramin)	Low density:<br>Increase @ 70s: 1.65 ([Ca^{2+}] fold of baseline ratio)*<br>Decrease @ 355s: 0.89 ([Ca^{2+}] fold of baseline ratio) *	
	Shah et al. (2017)	MLO-Y4 cells (n.g./ n.g. (n.g.), n.g., n.g., n.g., 15×10^3 or 25×10^3 cells per slide/n.g.)	n.g.	20s, 30min, 24h/n.g.	16dyn/cm²	n.g.			Increase (intracellular calcium Fluorescence microscopy, Fluo-4AM)		
	Zhang et al. (2015)	Murine long bone osteocyte Y4 (MLO-Y4) cells (n.g./ n.g. (n.g.), n.g., n.g., n.g., 100,000 cells/slide and 150,000 cells per slide/70-80%)	Oscillatory laminar	3min-2h @ 1Hz	1Pa	Custom-made			Fluctuated increase (Fluorescence microscopy, Fura-2 AM)		
	Reilly et al. (2003)	MLO-Y4 osteocytic cell line (n.g./ n.g. (n.g.), n.g., n.g., n.g., 200000 cell per slide/80%)	Oscillatory laminar	1h/n.g.	2Pa, 1Pa	Custom-made			Fluctuated increase (fluorescence microscopy, Fura-2 AM)		
	Lu et al. (2012b)	Osteocyte-like MLO-Y4 cells (n.g./ n.g. (n.g.), n.g., n.g., n.g., n.g./70-80%)	Steady laminar	9min (sampling at 60s 120s, 240s, 360s, 480s, 600s during FSS) @ 1Hz	20dyn/cm²	Custom-made			Fluctuated increase (Fura-2 AM microscopy)	steady; laminar (80s): 4.9 ([Ca^{2+}] intensity of baseline ratio))†	
	Lu et al. (2012a)	Osteocyte-like MLO-Y4 cells (n.g./ n.g. (n.g.), n.g., n.g., n.g., 1.0×10^4 cell per cm² slide area/n.g.)	Steady laminar (magnetic gear pump)	9 min /n.g.	0.5Pa, 1Pa, 2Pa, 4Pa	Custom-made			Fluctuated increase (fluorescence microscopy, Fura-2 AM)		
	Jing et al. (2013)	Osteocyte-like MLO-Y4 cells (n.g./ n.g. (n.g.), n.g., n.g., n.g., n.g./n.g.)	Steady laminar (magnetic gear pump)	10min period/n.g.	0.5Pa, 1Pa, 2Pa, 4Pa	n.g.			Fluctuated increase (fluorescence microscopy, Fura-2 AM)		

Official gene symbol or metabolite abbreviation	Reference	Cell type (age/ number and sex of donor (health status), tooth type, isolation method, passages used, cell density/confluency)	Flow type (Steady laminar, Pulsatile laminar, or Oscillatory laminar)	FSS duration and frequency	FSS magnitude	FSS apparatus	Gene expression: Increase, decrease, no change (method w/ reference gene); methods: RT-qPCR, sqPCR, northern hybridization	Gene expression: when it reaches peak and peak's magnitude (fold change; relative gene expression; times or ratio; unclear = ?)	Protein expression: Increase, decrease, no change (method w/reference); methods: ELISA, WB, RIA, EMSA, IF	Protein expression: When it reaches peak and peak's magnitude (times or ratio; unclear = ?)	Remarks including fluorescent microscopy assay
	Middleton et al. (2018)	Osteocyte-like MLO-Y4 cells (n.g./ n.g. (n.g.), n.g., n.g., P40, 1×10^6 cells per mL / 80%)	Steady laminar	2h/n.g.	1Pa	Custom-made, Steady fluid flow microfluidics syringe			Increase (Fluorescence microscopy, Fura-2 AM)		
	Rath et al. (2010)	Osteocyte-like MLO-Y4 cells (n.g./ n.g. (n.g.), n.g., n.g., n.g., n.g./70–80%)	Pulsatile laminar	n.g./n.g.	2dyn/cm^2, 8dyn/cm^2, 16dyn/cm^2	Parallel plate, live-cell micro-observation chamber (Focht Chamber System 2, Bioptechs Inc., Butler, PA, USA)			Fluorescence microscopy (Fluo-4 AM): increase with increasing shear stress.		
	Bakker et al. (2009)	MLO-Y4 osteocytes (n.g./ n.g. (n.g.), n.g., n.g., P30-35, 2×10^4 cells per cm^2/n.g.)	Pulsatile laminar	30min @ 5Hz	0.7±0.3Pa	Custom-made			Fluorescence microscopy (Fluo-4/AM): Fluctuated increase		
	Xu et al. (2012)	MLO-Y4 cell line (n.g./ n.g. (n.g.), n.g., n.g., n.g., 2.2×10^6 per slide/n.g.)	Oscillatory laminar	2h @ 1Hz	1Pa (10dyn/cm^2)	Custom-made	Increase (RT-qPCR, 18S)	2h: 6.9(FC)†			
Runx2	Chen et al. (2015)	MLO-Y4 osteocyte-like murine cells (n.g./ n.g. (n.g.), n.g., n.g., n.g., 4000 cells per cm^2/80%)	Oscillatory laminar	24h @ 0.5Hz	Amplitude of 1.5cm	Rocking platform	Decrease (RT-qPCR, GAPDH)	0.04/0.19 = 0.21 (ratio-calc)†			
	González et al. (2017)	Mouse osteocytes (MLO-Y4 cell line) (n.g./ n.g. (n.g.), n.g., n.g., n.g., n.g. n.g./n.g.)	Pulsatile laminar	30min/n.g.	10dyn/cm^2 (8.5cm/s)	FlexCell Streamer	Increase (RT-qPCR, 18S)	9.3/4 = 2.3 (ratio-calc)†			
	Deepak et al. (2017)	Murine MLO-Y4 osteocyte-like cells (n.g./ n.g. (n.g.), n.g., n.g., n.g., 4×10^4 cells per slide/n.g.)	Oscillatory laminar	1h @ 0.5Hz (24h post-PFF incubation)	1Pa	Custom-made	Decrease (RT-qPCR, GAPDH)	0.99 (FC)†			
Sost	Li et al. (2013)	MLO-Y4 cells (n.g./ n.g. (n.g.), n.g., n.g., n.g., n.g./75–85%)	Steady laminar (magnetic gear pump)	0h, 0.5h, 1h, 2h, 4h, 8h, 12h, 24h / n.g.	16dyn/cm^2, 30dyn/cm^2	Custom-made parallel-plate flow chamber	16dyn/cm^2: decrease with plateau (RT-qPCR, GAPDH)<br>30dyn/cm^2: increase then baseline (RT-qPCR, GAPDH)	16dyn/cm^2 @ 2h: 0.5 (rel.)†<br>16dyn/cm^2 @ 8h: 0.5 (rel.)†<br>16dyn/cm^2 @ 24h: 0.1 (rel.)†<br>30dyn/cm^2 @ 2h: 1.8 (rel.)†<br>30dyn/cm^2 @ 24h: 1 (rel.)†	16dyn/cm^2: 24h: decrease (WB, GAPDH)<br>30dyn/cm^2: increase (WB, GAPDH)	16dyn/cm^2 @ 24h:0.27/0.55 = 0.49 (ratio-calc)*<br>30dyn/cm^2 @24h: 0.44/0.34 = 1.3 (ratio-calc)†	16dyn/cm^2 (physiological levels), 30dyn/cm^2 (high levels)
	Riquelme et al. (2021)	MLO-Y4 osteocytic cells (n.g./ n.g. (n.g.), n.g., n.g., n.g., n.g./n.g.)	Steady laminar	10min/n.g.	8dyn/cm2	Custom-made			Decrease (WB, β-actin)	0.6 (ratio)†	
	Yan et al. (2018)	Osteocyte-like MLO-Y4 cell line (n.g./ n.g. (n.g.), n.g., n.g., n.g., 1×10^5 cells per mL/n.g.)	Steady laminar	2h @ n.g. (0h, 3h, 6h post-FSS incubation)	1Pa (10dyn/cm^2)	n.g.	Decrease then increase (RT-qPCR, GAPDH)	3h post-FSS: 0.007/0.999 = 0.007 (ratio-calc)†<br>6h post-FSS:1.65/1.015 = 1.62 (ratio-calc)†			
	Geoghegan et al. (2019)	MLO-Y4 mouse osteocyte-like cells (n.g./ n.g. (n.g.), n.g., n.g., n.g., 200,000 cells per slide/n.g.)	Oscillatory laminar	1h @ 0.5Hz	1Pa	Custom-made	Decrease (RT-qPCR, Rpl13a)	0.88 (rel.)†			
Tnfsf11	Haugh et al. (2015)	MLO-Y4 mouse osteocytes (n.g./ n.g. (n.g.), n.g., n.g., n.g., 200000 cells per slide/n.g.)	Oscillatory laminar	1h @ 1Hz	1Pa	Custom-made	Decrease (RT-qPCR, GAPDH)	0.8 (FC)†			
	Li et al. (2013)	MLO-Y4 cells (n.g./ n.g. (n.g.), n.g., n.g., n.g., n.g./75–85%)	Steady laminar (magnetic gear pump)	0h, 0.5h, 1h, 2h, 4h, 8h, 12h, 24h / n.g.	16dyn/cm^2, 30dyn/cm^2	Custom-made parallel-plate flow chamber	16dyn/cm^2: increase (RT-qPCR, GAPDH)<br>30dyn/cm^2: increase (RT-qPCR, GAPDH)	16dyn/cm^2 @ 0.5h: 7.2 (rel.)†<br>30dyn/cm^2 @ 24h: 6.8 (rel.)†	16dyn/cm^2: Increase then decrease (WB, GAPDH)<br>30dyn/cm^2: increase (WB, GAPDH)	16dyn/cm^2 @ 2h: 0.6/0.36 = 1.7 (ratio-calc)*<br>24h: 0.25/0.36 = 0.7 (ratio-calc)*<br>30dyn/cm^2 @24h: 0.91/0.46 = 1.97 (ratio-calc)†	16dyn/cm^2 (physiological levels), 30dyn/cm^2 (high levels)

Official gene symbol or metabolite abbreviation	Reference	Cell type (age/ number and sex of donor (health status), tooth type, isolation method, passages used, cell density/confluency)	Flow type (Steady laminar, Pulsatile laminar, or Oscillatory laminar)	FSS duration and frequency	FSS magnitude	FSS apparatus	Gene expression: Increase, decrease, no change (method w/ reference gene); methods: RT-qPCR, sqPCR, northern hybridization	Gene expression: when it reaches peak and peak's magnitude (fold change; relative gene expression; times or ratio; unclear = ?)	Protein expression: Increase, decrease, no change (method w/reference); methods: ELISA, WB, RIA, EMSA, IF	Protein expression: When it reaches peak and peak's magnitude (times or ratio; unclear = ?)	Remarks including fluorescent microscopy assay
	Yan et al. (2018)	Osteocyte-like MLO-Y4 cell line (n.g./ n.g. (n.g.), n.g., n.g., n.g., 1×10^5 cells per mL/n.g.)	Steady laminar	2h @ n.g. (0h, 3h, 6h post-FSS incubation)	1Pa (10dyn/cm^2)	n.g.	Increase then decrease with plateau (RT-qPCR, GAPDH)	0h post-FSS: 1.18 (rel.)† 3h post-FSS: 0.452/ 1.02 = 0.44 (ratio-calc)† 6h post-FSS: 0.51/1.02 = 0.5 (ratio-calc) †	Decrease (WB, β-actin)	3h post-FSS: 0.2 (ratio)†	
	Shah et al. (2017)	MLO-Y4 cells (n.g./ n.g. (n.g.), n.g., n.g., n.g., 15×10^3 or 25×10^3 cells per slide/n.g.)	n.g.	20s, 30min, 24h/n.g.	16dyn/cm^2	n.g.	30min: increase (RT-qPCR, GAPDH) 24h: increase (RT-qPCR, GAPDH)	30min: 6.6 (FC)† 24h: 2.3 (FC)†			
	Maycas et al. (2017)	Osteocytic MLO-Y4 cell line (n.g./ n.g. (n.g.), n.g., n.g., n.g., 2×10^4 cells per cm^2/n.g.)	Pulsatile laminar	10min (sampling 18h post FSS) / 8Hz	10dyn/cm^2	Flexcell® Streamer®			Decrease (ELISA)	6819.1pg/mL; 6819.1/8793 = 0.77 (ratio-calc)†	
	Kulkarni et al. (2012a)	MLO-Y4 osteocytes (n.g./ n.g. (n.g.), n.g., n.g., P30-31, 2×10^4 cells per cm^2/n.g.)	Pulsatile laminar	1h @ 5Hz	Mean FSS: 0.7Pa, pulse amplitude of 0.3Pa, peak SS: 8.4Pa/s	Custom-made	Increase (RT-qPCR, GAPDH)	33.2/10 = 3.3 (ratio-calc)†			
	Fahlgren et al. (2018)	MLO-Y4 osteocytes (n.g./ n.g. (n.g.), n.g., n.g., P31–33, 1.3×10^3 cells/cm^2 per slide/n.g.)	Pulsatile Laminar (SP: square wave; PL: sinusoidal wave)	60min / (1Hz, 5Hz)	2.9±2.9Pa @ 1Hz square wave (SP); 0.7±0.7Pa @ 5Hz, sinusoidal wave (PL); Unloaded	Custom-made	SP/PL: decrease SP/unloading decrease PL/unloading: decrease (RT-qPCR, S18)	SP/PL: 0.75/ 0.99 = 0.75 (ratio-calc)† SP/unloading: 0.75/ 1.08 = 0.69 (ratio-calc)† PL/unloading: 0.99/1.08 = 0.92 (ratio-calc)†	SP/PL: increase SP/unloading decrease PL/unloading: decrease (ELISA)	SP/PL: 1.05pg/mL; 1.05/0.99 = 1.1 (ratio-clac)† SP/unloading: 1.05pg/mL; 1.05/1.05 = 1 (ratio-calc)† PL/unloading: 0.99pg/mL; 0.99/1.05 = 0.94 (ratio-calc)†	SP (supraphysiological loading), PL (physiological loading) SP/PL: increase SP/unloading increase
	Bakker et al. (2013b)	MLO-Y4 osteocytes (n.g./ n.g. (n.g.), n.g., n.g., P30-38, 2×10^4 cells per cm^2/n.g.)	Pulsatile laminar	60min @ 5Hz	0.7±0.3Pa	Custom-made	Increase (RT-qPCR, GAPDH)	1.93 (rel.)†			
	Kulkarni et al. (2010)	MLO-Y4 osteocytes (n.g./ n.g. (n.g.), n.g., n.g., P30-31, 1×10^3 cells per cm^2/n.g.)	Pulsatile laminar	1h (sampling 0h, 1h, 6h post FSS) / 5Hz	Mean shear stress: 0.7Pa, pulse amplitude of 0.3Pa, peak SS of 8.4Pa/s	Custom-made	Increase (RT-qPCR, GAPDH)	0h post FSS: 38.9/20.8 = 1.9 (ratio-calc)†			
	Liao et al. (2017)	MLO-Y4 osteocytes (n.g./ n.g. (n.g.), n.g., n.g., n.g., 200000 cells per slide/80-90%)	Pulsatile laminar	2h @ 5Hz	0.7±0.3Pa @ 5Hz L-PFF: 4dyn/cm^2 H-PFF: 16dyn/cm^2	Streamer® Shear Stress Device (Flexcell)	Increase (RT-qPCR, GAPDH)	L-PFF: 1.2 (rel.)† H-PFF: 1.04 (rel.)†	Decrease (ELISA)	L-PFF: 0.63 (ratio)† H-PFF: 0.47 (ratio)†	
	Xia et al. (2010)	MLO-Y4 cells (n.g./ n.g. (n.g.), n.g., n.g., n.g., n.g./n.g.)	Steady laminar	0h, 0.5h, 2h, 8h, 16h / n.g.	16dyn/cm^2	Custom-made			Increase (WB, AKT)	0.5h: 16.9 (ratio)†	
Akt1	Xia et al. (2010)	MLO-Y4 cells (n.g./ n.g. (n.g.), n.g., n.g., n.g., n.g./n.g.)	Steady laminar	0h, 0.5h, 2h, 8h, 16h / n.g.	16dyn/cm^2	Custom-made			Increase (WB, AKT)	0.5h: 15.4 (ratio)†	
	Riquelme et al. (2021)	MLO-Y4 osteocytic cells (n.g./ n.g. (n.g.), n.g., n.g., n.g., n.g./n.g.)	Steady laminar	10min/n.g.	8dyn/cm2	Custom-made			Increase (WB, β-actin)	2.2 (ratio)†	
	Batra et al. (2014)	MLO-Y4 osteocytic cells (n.g./ n.g. (n.g.), n.g., n.g., n.g., n.g./n.g.)	Steady laminar	0.5h, 2h, 4h, 24h)/n.g.	16dyn/cm^2	Custom-made			Increase (WB, β-actin)	0.5h: 13.0 (ratio)†	
	Chen et al. (2015)	MLO-Y4 osteocyte-like murine cells (n.g./ n.g. (n.g.), n.g., n.g., n.g., 4000 cells per cm^2/80%)	Oscillatory laminar	24h @ 0.5Hz	Amplitude of 1.5cm	Rocking platform	Increase (RT-qPCR, GAPDH)	0.8/0.03 = 26.6 (ratio-calc)†			
Bglap	Deepak et al. (2017)	Murine MLO-Y4 osteocyte-like cells (n.g./ n.g. (n.g.), n.g., n.g., n.g., 4x10^4 cells per slide/n.g.)	Oscillatory laminar	1h @ 0.5Hz (24h post-PFF incubation)	1Pa	Custom-made	Increase (RT-qPCR, GAPDH)	12.5 (FC)†			
	Yan et al. (2018)	Osteocyte-like MLO-Y4 cell line (n.g./ n.g. (n.g.), n.g., n.g., n.g., 1×10^5 cells per mL/n.g.)	Steady laminar	2h @ n.g. (0h, 3h, 6h post-FSS incubation)	1Pa (10dyn/cm^2)	n.g.	Increase (RT-qPCR, GAPDH)	3h post-FSS: 2.08/1.02 = 1.97 (ratio-calc)†	Increase (WB, β-actin)	3h post-FSS: 2.4/1.17 = 2 (ratio-calc)†	
	Xia et al. (2010)	MLO-Y4 cells (n.g./ n.g. (n.g.), n.g., n.g., n.g., n.g./n.g.)	Steady laminar	0h, 0.5h, 2h, 8h, 16h / n.g.	16dyn/cm^2	Custom-made			Increase (WB, β-actin)	0.5h: 2.3 (ratio)†	
Ctnnb1	Xia et al. (2010)	MLO-Y4 cells (n.g./ n.g. (n.g.), n.g., n.g., n.g., n.g./n.g.)	Steady laminar	0h, 0.5h, 2h, 8h, 16h / n.g.	16dyn/cm^2	Custom-made			Increase (WB, β-actin)	0.5h: 2.8 (ratio)†	

Official gene symbol or metabolite abbreviation	Reference	Cell type (age/ number and sex of donor (health status), tooth type, isolation method, passages used, cell density/confluency)	Flow type (Steady laminar, Pulsatile laminar, or Oscillatory laminar)	FSS duration and frequency	FSS magnitude	FSS apparatus	Gene expression: Increase, decrease, no change (method w/ reference gene); methods: RT-qPCR, sqPCR, northern hybridization	Gene expression: when it reaches peak and peak's magnitude (fold change; relative gene expression; times or ratio; unclear = ?)	Protein expression: Increase, decrease, no change (method w/reference); methods: ELISA, WB, RIA, EMSA, IF	Protein expression: When it reaches peak and peak's magnitude (times or ratio; unclear = ?)	Remarks including fluorescent microscopy assay
	Yan et al. (2018)	Osteocyte-like MLO-Y4 cell line (n.g./ n.g. (n.g.), n.g., n.g., n.g., 1×10^5 cells per mL/n.g.)	Steady laminar	2h @ n.g. (0h, 3h, 6h post-FSS incubation)	1Pa (10dyn/cm^2)	n.g.	Increase (RT-qPCR, GAPDH)	0h post-FSS: 1.50/1.04 = 1.44 (ratio-calc)†	Increase (WB, β-actin)	3h post-FSS: 3.4/1.2 = 2.8 (ratio-calc)†	
	Santos et al. (2010)	MLO-Y4 osteocytes (n.g./ n.g. (n.g.), n.g., n.g., n.g., 2.75×10^5 cells per slide/n.g.)	Pulsatile laminar	30min @ 5Hz	0.7±0.3Pa	Custom-made			Increase (ELISA)	1194.8pg/mL; 1194.8/712.8 = 1.7 (ratio-calc)†	
	Maycas et al. (2017)	Osteocytic MLO-Y4 cell line (n.g./ n.g. (n.g.), n.g., n.g., n.g., 2×10^4 cells per cm^2/n.g.)	Pulsatile laminar	10min (sampling 18h post FSS) / 8Hz	10dyn/cm^2	Flexcell® Streamer®			Increase (WB, Lamin B1)	1.8 (ratio)†	
	Maycas et al. (2017)	Osteocytic MLO-Y4 cell line (n.g./ n.g. (n.g.), n.g., n.g., n.g., 2×10^4 cells per cm^2/n.g.)	Pulsatile laminar	10min (sampling 18h post FSS) / 8Hz	10dyn/cm^2	Flexcell® Streamer®			Increase (WB, α-tubulin)	4.7 (ratio)†	
	Maycas et al. (2015)	MLO-Y4 cells (n.g./ n.g. (n.g.), n.g., n.g., n.g., n.g./n.g.)	Pulsatile laminar	10min @ 8Hz	10dyn/cm^2	Pulsatile laminar (Flexcell Streamer)			Nuclear β-catenin: increase (WB, lamin B1)<br>Total β-catenin: increase (WB, α-tubulin)	Nuclear β-catenin: 1.8 (ratio)†<br>Total β-catenin: 4.6 (ratio)†	
	Santos et al. (2009)	MLO-Y4 osteocytes (n.g./ n.g. (n.g.), n.g., n.g., n.g., 2×10^5 cells per slide/n.g.)	Pulsatile laminar	1h (1h, 3h post-PFF incubation) / 5Hz	mean FSS: 0.7Pa, pulse amplitude of 0.3Pa	Custom-made	Temporary decrease then increase (RT-qPCR, GAPDH)	0.5h post-PFF: 0.9 (rel.)†<br>1h post-PFF: 1.3 (rel.)†			
	de Castro et al. (2015)	MLO-Y4 cell line (n.g./ n.g. (n.g.), n.g., n.g., n.g., n.g./n.g.)	Pulsatile laminar	10min @ 8Hz	10dyn/cm^2	Flexcell Streamer shear stress device (Flexcell)			Increase (WB, β-actin)	2.2 (ratio)†	
	Geoghegan et al. (2019)	MLO-Y4 mouse osteocyte-like cells (n.g./ n.g. (n.g.), n.g., n.g., n.g., 200,000 cells per slide/n.g.)	Oscillatory laminar	1h @ 0.5Hz	1Pa	Custom-made	Decrease (RT-qPCR, Rpl13a)	0.50 (ratio)†			
RANKL/OPG	Zhang et al. (2015)	Murine long bone osteocyte Y4 (MLO-Y4) cells (n.g./ n.g. (n.g.), n.g., n.g., n.g., 100,000 cells/slide and 150,000 cells per slide/70-80%)	Oscillatory laminar	3min-2h @ 1Hz	1Pa	Custom-made	Decrease (RT-qPCR, 18S)	2h: 0.69/1.3 = 0.5 (ratio-calc)†			
	Litzenberger et al. (2010)	MLO-Y4 parental cells (n.g./ n.g. (n.g.), n.g., n.g., n.g., n.g./n.g.)	Oscillatory laminar	2h @1Hz	1Pa	Custom-made	Decrease (RT-qPCR, 18S)	0.59 (ratio)†			
	Li et al. (2012)	MLO-Y4 osteocyte-like cells (n.g./ n.g. (n.g.), n.g., n.g., n.g., n.g./70%)	Oscillatory laminar	1h, 2h, 4h @ 0.5Hz, 1Hz, 2Hz, 5Hz	0.5Pa, 1Pa, 2Pa, 5Pa	Custom-made	Duration-related: decrease (RT-qPCR, 18S)	2Hz, 0.5Pa@2h: 0.42 (ratio)†<br>2Hz, 1.0Pa@1h: 0.85 (ratio)†<br>2Hz, 2.0Pa@2h: 0.46 (ratio)†<br>2Hz, 5.0Pa@1h: 0.44 (ratio)†<br>0.5Hz, 0.5Pa@2h: 1.1 (ratio)†<br>0.5Hz, 1.0Pa@2h: 0.7 (ratio)†<br>0.5Hz, 2.0Pa@2h: 0.3 (ratio)†<br>0.5Hz, 5.0Pa@4h: 0.58 (ratio)†			
	Yan et al. (2018)	Osteocyte-like MLO-Y4 cell line (n.g./ n.g. (n.g.), n.g., n.g., n.g., 1×10^5 cells per mL/n.g.)	Steady laminar	2h @ n.g. (0h, 3h, 6h post-FSS incubation)	1Pa (10dyn/cm^2)	n.g.	Decrease (RT-qPCR, GAPDH)	3h post-FSS: 0.17/0.99 = 0.17 (ratio-calc)†	Decrease (WB, β-actin)	3h post-FSS: 0.12 (ratio)†	
	Fahlgren et al. (2018)	MLO-Y4 osteocytes (n.g./ n.g. (n.g.), n.g., n.g., P31–33, 1.3×10^3 cells/cm^2 per slide/n.g.)	Pulsatile Laminar (SP: square wave; PL: sinusoidal wave)	60min / (1Hz, 5Hz)	2.9±2.9Pa @ 1Hz square wave (SP); 0.7±0.7Pa @ 5Hz, sinusoidal wave (PL); Unloaded	Custom-made			SP/PL: decrease (ELISA)<br>SP/unloading: decrease (ELISA)<br>PL/unloading: increase (ELISA)	SP/PL: 0.15/1.0 = 0.15 (ratio-calc)†<br>SP/unloading: 0.15/0.54 = 0.3 (ratio-calc)†<br>PL/unloading: 1.0/0.54 = 1.85 (ratio-calc)†	SP (supraphysiological loading), PL (physiological loading)

Official gene symbol or metabolite abbreviation	Reference	Cell type (age/ number and sex of donor (health status), tooth type, isolation method, passages used, cell density/confluency)	Flow type (Steady laminar, Pulsatile laminar, or Oscillatory laminar)	FSS duration and frequency	FSS magnitude	FSS apparatus	Gene expression: Increase, decrease, no change (method w/ reference gene); methods: RT-qPCR, sqPCR, northern hybridization	Gene expression: when it reaches peak and peak's magnitude (fold change; relative gene expression; times or ratio; unclear = ?)	Protein expression: Increase, decrease, no change (method w/reference); methods: ELISA, WB, RIA, EMSA, IF	Protein expression: When it reaches peak and peak's magnitude (times or ratio; unclear = ?)	Remarks including fluorescent microscopy assay
Spp1	Kulkarni et al. (2010)	MLO-Y4 osteocytes (n.g./ n.g. (n.g.), n.g., n.g., P30-31, 1×10³ cells per cm²/n.g.)	Pulsatile laminar	1h (sampling 0h, 1h, 6h post FSS) / 5Hz	Mean shear stress: 0.7Pa, pulse amplitude of 0.3Pa, peak SS of 8.4Pa/s	Custom-made	Decrease (RT-qPCR, GAPDH)	0h post FSS: 1.1/1.4 = 0.8 (ratio-calc)†			
	Liao et al. (2017)	MLO-Y4 osteocytes (n.g./ n.g. (n.g.), n.g., n.g., n.g., 200000 cells per slide/80-90%)	Pulsatile laminar	2h @ 5Hz	0.7±0.3Pa @ 5Hz L-PFF: 4dyn/cm² H-PFF: 16dyn/cm²	Streamer® Shear Stress Device (Flexcell)	Decrease (RT-qPCR, GAPDH)	L-PFF: 0.76 (ratio)† H-PFF: 0.56 (ratio)†	Decrease (ELISA)	L-PFF: 0.24 (ratio)† H-PFF: 0.36 (ratio)†	
	Xu et al. (2012)	MLO-Y4 cell line (n.g./ n.g. (n.g.), n.g., n.g., n.g., 2.2×10⁶ per slide/n.g.)	Oscillatory laminar	2h @ 1Hz	1Pa (10dyn/cm²)	Custom-made	Increase (RT-qPCR, 18S)	2h: 2.65 (FC)†			
	Chen et al. (2015)	MLO-Y4 osteocyte-like murine cells (n.g./ n.g. (n.g.), n.g., n.g., n.g., 4000 cells per cm²/80%)	Oscillatory laminar	24h @ 0.5Hz	Amplitude of 1.5cm	Rocking platform	Increase (RT-qPCR, GAPDH)	6.3/2.3 = 2.7(ratio-calc)†			
	Deepak et al. (2017)	Murine MLO-Y4 osteocyte-like cells (n.g./ n.g. (n.g.), n.g., n.g., n.g., 4x10⁴ cells per slide/n.g.)	Oscillatory laminar	1h @ 0.5Hz (24h post-PFF incubation)	1Pa	Custom-made	Increase (RT-qPCR, GAPDH)	8.4 (FC)†			
	Liu et al. (2015)	1-MLO-Y4 osteocyte-like cells (n.g./ n.g. (n.g.), n.g., n.g., n.g., n.g./80%)	Oscillatory laminar	1h (sampling 24h post FSS) / 1Hz	2Pa	Custom-made	Increase (RT-qPCR, 18S)	24h post FSS:0.7/0.4 = 1.75 (ratio-calc)†	Increase (ELISA)	24h post FSS: 2.0/0.9 = 2.2 (ratio-calc)†	
Vegfa	Thi et al. (2010)	Osteocytic MLO-Y4 cells (n.g./ n.g. (n.g.), n.g., n.g, n.g., n.g./n.g.)	Pulsatile laminar	5h @ 1Hz	5dyn/cm²	Cytodyne parallel plate flow chamber			Increase (ELISA); Increase (WB, GAPDH)	ELISA: 20.4pg/µg protein; 20.4/1.1 = 18.5 (ratio/calc)† WB: no quantitative information given	
	Maycas et al. (2017)	Osteocytic MLO-Y4 cell line (n.g./ n.g. (n.g.), n.g., n.g., n.g., 2×10⁴ cells per cm²/n.g.)	Pulsatile laminar	10min (sampling 18h post FSS) / 8Hz	10dyn/cm²	Flexcell® Streamer®			Decrease (ELISA)	635.6pg/mL; 635.6/3156.8 = 0.2 (ratio-calc)†	
	Juffer et al. (2012)	MLO-Y4 osteocytes (n.g./ n.g. (n.g.), n.g., n.g., n.g., 3x10⁵ cells per glass slide /n.g.)	Pulsatile laminar	1h (sampling 1h, 3h, 6h, 24h post FSS) / 5Hz	22Pa/s; 44Pa/s	Custom-made	22Pa/s: increase followed by plateau (RT-qPCR, GAPDH)	1h post FSS: 2.4 (rel.)† 6-24h post FSS: ≈1.6 (rel.)	Increase (ELISA)	24h post FSS: 1.6ng; 1.6/0.8 = 2 (ratio-calc)†	22Pa/s ("low shear stress"); 44Pa/s ("high shear stress")
	González et al. (2017)	Mouse osteocytes (MLO-Y4 cell line) (n.g./ n.g. (n.g.), n.g., n.g., n.g., n.g. n.g./n.g.)	Pulsatile laminar	30min/n.g.	10dyn/cm² (8.5cm/s)	FlexCell Streamer	Increase (RT-qPCR, 18S)	2.96/1.4 = 2.1 (ratio-calc)†			
	de Castro et al. (2015)	MLO-Y4 cell line (n.g./ n.g. (n.g.), n.g., n.g., n.g., n.g./n.g.)	Pulsatile laminar	10min @ 8Hz	10dyn/cm²	Flexcell Streamer shear stress device (Flexcell)	Increase (RT-qPCR, 18S)	6h: 3.2 (rel.)†			
	Alford et al. (2003)	Cells of the osteocytic line MLOY-4 (n.g./ n.g. (n.g.), n.g., n.g., n.g., 250000 cells per slide/n.g.)	Oscillatory laminar	1h @ 5Hz	±10dyn/cm²	Custom-made			Increase (WB, n.g.)	2.4 (ratio)†	
Gja1	Ren et al. (2013)	Osteocyte-like MLO-Y4 cells (n.g./ n.g. (n.g.), n.g., n.g., n.g., 8×10⁴ cells/n.g.)	Oscillatory laminar	2h @ 1Hz (2h post-FF incubation)	12dyn	Parallel plate flow chamber (Pecon, Germany)			Increase (WB, actin)	1.2 (ratio)†	
	Xia et al. (2010)	MLO-Y4 cells (n.g./ n.g. (n.g.), n.g., n.g., n.g., n.g./n.g.)	Steady laminar	0h, 0.5h, 2h, 8h, 16h / n.g.	16dyn/cm²	Custom-made			Increase (WB, β-actin)	16h: 2.6 (ratio)†	
	Shah et al. (2017)	MLO-Y4 cells (n.g./ n.g. (n.g.), n.g., n.g., n.g., 15×10³ or 25×10³ cells per slide/n.g.)	n.g.	20s, 30min, 24h/n.g.	16dyn/cm²	n.g.	30min: increase (RT-qPCR, GAPDH) 24h: increase (RT-qPCR, GAPDH)	30min: 1.8 (FC)† 24h: 2.2 (FC)†			
	Thi et al. (2003)	MLO-Y4 cells (n.g./ n.g. (n.g.), n.g., n.g., n.g., n.g./n.g.)	Steady laminar	1h, 2h, or 3 h / n.g.	5dyn/cm², 20dyn/cm²	Parallel-plate flow chamber (Cytodyne, La Jolla, CA)	5dyn/cm² @ 3h: increase (Northern blot/18S) 20dyn/cm² @ 1h: increase (Northern, 18S)	5dyn/cm² @ 3h: 1.5 (rel.)* 20dyn/cm² @ 1h: 1.1 (rel.)*			

Official gene symbol or metabolite abbreviation	Reference	Cell type (age/ number and sex of donor (health status), tooth type, isolation method, passages used, cell density/confluency)	Flow type (Steady laminar, Pulsatile laminar, or Oscillatory laminar)	FSS duration and frequency	FSS magnitude	FSS apparatus	Gene expression: Increase, decrease, no change (method w/ reference gene); methods: RT-qPCR, sqPCR, northern hybridization	Gene expression: when it reaches peak and peak's magnitude (fold change; relative gene expression; times or ratio; unclear = ?)	Protein expression: Increase, decrease, no change (method w/reference); methods: ELISA, WB, RIA, EMSA, IF	Protein expression: When it reaches peak and peak's magnitude (times or ratio; unclear = ?)	Remarks including fluorescent microscopy assay
	Li et al. (2013)	MLO-Y4 cells (n.g./ n.g. (n.g.), n.g., n.g., n.g., n.g./75–85%)	Steady laminar (magnetic gear pump)	0h, 0.5h, 1h, 2h, 4h, 8h, 12h, 24h / n.g.	16dyn/cm^2, 30dyn/cm^2	Custom-made parallel-plate flow chamber	16dyn/cm^2: increase (RT-qPCR, GAPDH) 30dyn/cm^2: decrease then increase (RT-qPCR, GAPDH)	16dyn/cm^2 @ 24h: 5.8 (rel.)† 30dyn/cm^2 @ 2h: 0.22 (rel.)† 30dyn/cm^2 @ 24h: 1.4 (rel.)†	16dyn/cm^2: Total and membrane Cxc43: increase (WB, GAPDH) 30dyn/cm^2: Total and membrane Cxc43: decrease (WB, GAPDH)	16dyn/cm^2: Total 24h:0.69/0.37 = 1.9 (ratio-calc)* 16dyn/cm^2: Membrane 24h:0.6/0.24 = 2.5 (ratio-calc)* 30dyn/cm^2: Total 24h:0.32/0.6 = 0.53 (ratio-calc)† 16dyn/cm^2: Membrane 24h:0.30/0.46 = 0.65 (ratio-calc)†	16dyn/cm^2 (physiological levels), 30dyn/cm^2 (high levels)<br><br>Membrane and Total Cx43 were extracted as per Author
	Cheng et al. (2001)	MLO-Y4 cells (n.g./ n.g. (n.g.), n.g., n.g., n.g., n.g./75–85%)	Steady laminar	2h (Sampling 0h, 0.5h, 2h, 4h, and 24h post FSS) / 5Hz	16dyn/cm^2	Custom-made			Increase then decrease (WB, n.g.)	4h post FSS: 44.6/29.7 = 1.5 (ratio-calc)† 24h post FSS: 6.5/16.5 = 0.4 (ratio-calc)†	
	Cherian et al. (2005)	MLO-Y4 cells (n.g./ n.g. (n.g.), n.g., n.g., n.g., 2.0×10^3, 7.5×10^3, 1.6×10^4, and 3.8×10^4 cells per cm^2/n.g.)	Steady laminar	30min, 2h / n.g.	16dyn/cm^2	Custom-made			biotinylated Cx43/ Total: increase (WB, Total Cx43)<br><br>biotinylated Cx43 bound: increase (WB, β-actin)	2h FF: 0.75/0.35 = 2.14 (ratio-calc)*<br><br>(biotinylated Cx43 bound): No quantitative information given	
	Yan et al. (2018)	Osteocyte-like MLO-Y4 cell line (n.g./ n.g. (n.g.), n.g., n.g., n.g., 1×10^5 cells per mL/n.g.)	Steady laminar	2h @ n.g. (0h, 3h, 6h post-FSS incubation)	1Pa (10dyn/cm^2)	n.g.	Increase (RT-qPCR, GAPDH)	3h post-FSS: 2.42/1.14 = 2.12 (ratio-calc)†	Increase (WB, β-actin)	3h post-FSS: 2.9 (ratio)†	
	Santos et al. (2009)	MLO-Y4 osteocytes (n.g./ n.g. (n.g.), n.g., n.g., n.g., 2×10^5 cells per slide/n.g.)	Pulsatile laminar	1h (1h, 3h post-PFF incubation) / 5Hz	mean FSS: 0.7Pa, pulse amplitude of 0.3Pa	Custom-made	Increase (RT-qPCR, GAPDH)	1h post-PFF: 1.5 (rel.)†			
	Ren et al. (2013)	Osteocyte-like MLO-Y4 cells (n.g./ n.g. (n.g.), n.g., n.g., n.g., 8×10^4 cells/n.g.)	Oscillatory laminar	2h @ 1Hz (2h post-FF incubation)	12dyn	Parallel plate flow chamber (Pecon, Germany)			Increase (WB, total ERK 1/2)	1.6 (ratio)†	
Mapk3; Mapk1	Maycas et al. (2017)	Osteocytic MLO-Y4 cell line (n.g./ n.g. (n.g.), n.g., n.g., n.g., 2×10^4 cells per cm^2/n.g.)	Pulsatile laminar	10min (sampling 18h post FSS) / 8Hz	10dyn/cm^2	Flexcell® Streamer®			Increase (WB, Lamin B1)	1.7 (ratio)†	
	Maycas et al. (2017)	Osteocytic MLO-Y4 cell line (n.g./ n.g. (n.g.), n.g., n.g., n.g., 2×10^4 cells per cm^2/n.g.)	Pulsatile laminar	10min (sampling 18h post FSS) / 8Hz	10dyn/cm^2	Flexcell® Streamer®			Increase (WB, ERK)	2.2 (ratio)†	
	Maycas et al. (2015)	MLO-Y4 cells (n.g./ n.g. (n.g.), n.g., n.g., n.g., n.g./n.g.)	Pulsatile laminar	10min @ 8Hz	10dyn/cm^2	Pulsatile laminar (Flexcell Streamer)			Increase (WB, ERK)	2.3 (ratio)†	
	Liao et al. (2017)	MLO-Y4 osteocytes (n.g./ n.g. (n.g.), n.g., n.g., n.g., 200000 cells per slide/80-90%)	Pulsatile laminar	2h @ 5Hz	0.7±0.3Pa @ 5Hz L-PFF: 4dyn/cm^2 H-PFF: 16dyn/cm^2	Streamer® Shear Stress Device (Flexcell)			Increase (WB, GAPDH)	No quantitative information given.	
	de Castro et al. (2015)	MLO-Y4 cell line (n.g./ n.g. (n.g.), n.g., n.g., n.g., n.g./n.g.)	Pulsatile laminar	10min @ 8Hz	10dyn/cm^2	Flexcell Streamer shear stress device (Flexcell)			Increase (WB, total ERK)	2.4 (ratio)†	
	Liu et al. (2015)	1-MLO-Y4 osteocyte-like cells (n.g./ n.g. (n.g.), n.g., n.g., n.g., n.g./80%)	Oscillatory laminar	1h (sampling 24h post FSS) / 1Hz	2Pa	Custom-made			Increase (ELISA)	2437.3pg/ml; 2437.3/1469.1 = 1.7 (ratio-calc)†	
PGE2	Zhang et al. (2015)	Murine long bone osteocyte Y4 (MLO-Y4) cells (n.g./ n.g. (n.g.), n.g., n.g., n.g., 100,000 cells/slide and 150,000 cells per slide/70-80%)	Oscillatory laminar	3min-2h @ 1Hz	1Pa	Custom-made			Increase (ELISA)†	2h: 4.2 (ratio)†	Note: interesting figure combining release and mRNA expression
	Reilly et al. (2003)	MLO-Y4 osteocytic cell line (n.g./ n.g. (n.g.), n.g., n.g., n.g., 200000 cell per slide/80%)	Oscillatory laminar	1h/n.g.	2Pa, 1Pa	Custom-made			Increase (ELISA)	4.4 (PGE2/μg DNA); 4.4 (ratio)†	

Official gene symbol or metabolite abbreviation	Reference	Cell type (age/ number and sex of donor (health status), tooth type, isolation method, passages used, cell density/confluency)	Flow type (Steady laminar, Pulsatile laminar, or Oscillatory laminar)	FSS duration and frequency	FSS magnitude	FSS apparatus	Gene expression: Increase, decrease, no change (method w/ reference gene); methods: RT-qPCR, sqPCR, northern hybridization	Gene expression: when it reaches peak and peak's magnitude (fold change; relative gene expression; times or ratio; unclear = ?)	Protein expression: Increase, decrease, no change (method w/reference); methods: ELISA, WB, RIA, EMSA, IF	Protein expression: When it reaches peak and peak's magnitude (times or ratio; unclear = ?)	Remarks including fluorescent microscopy assay
	Genetos et al. (2007)	Osteocytic MLO-Y4 cells (n.g./ n.g. (n.g.), n.g., n.g., n.g., 900 cells per cm^2 /n.g.)	Oscillatory laminar	30min @1Hz	20dyn/cm2	Custom-made			Increase (ELISA)	2.2/ 0.93 = 2.4 (ratio-calc)†	
	Litzenberger et al. (2010)	MLO-Y4 parental cells (n.g./ n.g. (n.g.), n.g., n.g., n.g., n.g./n.g.)	Oscillatory laminar	2h @1Hz	1Pa	Custom-made			Increase (ELISA)	1.8 (ratio)†	
	Haugh et al. (2015)	MLO-Y4 mouse osteocytes (n.g./ n.g. (n.g.), n.g., n.g., n.g., 200000 cells per slide/n.g.)	Oscillatory laminar	1h @ 1Hz	1Pa	Custom-made			Increase (EIA)	10.2pg/ngDNA; 10.2/3.9 = 2.6 (ratio-calc)†	
	Xu et al. (2014)	MLO-Y4 cell line (n.g./ n.g. (n.g.), n.g., n.g., n.g., 2.2×10^6 per slide/n.g.)	Oscillatory laminar	2h @ 1Hz	1Pa (10dyn/cm^2)	Custom-made			Increase (EIA)	178.62µg/mL;178.62/30.2 = 5.9 (ratio-calc)†	
	Deepak et al. (2017)	Murine MLO-Y4 osteocyte-like cells (n.g./ n.g. (n.g.), n.g., n.g., n.g., 4×10^4 cells per slide/n.g.)	Oscillatory laminar	1h @ 0.5Hz (24h post-PFF incubation)	1Pa	Custom-made			Increase (ELISA)	826.6/391.1 = 2.1 (ratio-calc)†	
	Cherian et al. (2005)	MLO-Y4 cells (n.g./ n.g. (n.g.), n.g., n.g., n.g., 2.0×10^3, 7.5×10^3, 1.6×10^4, and 3.8×10^4 cells per cm^2/n.g.)	Steady laminar	30min, 2h / n.g.	16dyn/cm^2	Custom-made			Intracellular PGE2: increase (EIA) Extracellular PGE2: increase (EIA)	2h FF: Intracellular PGE2: 9.5pg/10^4 cells; 9.5/1.5 = 6.3 (ratio-calc)† 2h FF: Extracellular PGE2: 10.8pg/10^4 cells; 10.8/ 1.8 = 6 (ratio-calc)†	
	Fahlgren et al. (2018)	MLO-Y4 osteocytes (n.g./ n.g. (n.g.), n.g., n.g., P31–33, 1.3×10^3 cells/cm^2 per slide/n.g.)	Pulsatile Laminar (SP: square wave; PL: sinusoidal wave)	60min / (1Hz, 5Hz)	2.9±2.9Pa @ 1Hz square wave (SP); 0.7±0.7Pa @ 5Hz, sinusoidal wave (PL); Unloaded	Custom-made			SP/PL: decrease SP/unloading: increase PL/unloading: increase (ELISA)	SP/PL: 0.88/0.99 = 0.88 (ratio-calc)† SP/unloading: 0.88/0.78 = 1.1 (ratio-calc)† PL/unloading: 0.99/0.78 = 1.3 (ratio-calc)†	SP (supraphysiological loading), PL (physiological loading)
	Bakker et al. (2013b)	MLO-Y4 osteocytes (n.g./ n.g. (n.g.), n.g., n.g., P30-38, 2×10^4 cells per cm^2/n.g.)	Pulsatile laminar	60min (sampling 0min, 10 min, 20min, 30min, 40min, 50min, 60min) / 5Hz	0.7±0.3Pa	Custom-made			Increase with plateau (ELISA)	5min: 512.8ng/3×10^5 cells; 512.8/356 = 1.4 (ratio-calc)† 60min: 520.8ng/3×10^5 cells; 520.8/428 = 1.2 (ratio-calc)†	
	Kamel et al. (2010)	MLO-Y4 cells (n.g./ n.g. (n.g.), n.g., n.g., P27, 5×10^5 cells per slide/70%)	Pulsatile laminar	2h @ 0.5Hz	(2, 4, 8, 16, 24 ±0.6) dyn/cm2	Flexcell® Streamer® Shear Stress Device			Increase (ELISA)	24dyn/cm^2 @120min: 1704.9pg/mL †	
	Geoghegan et al. (2019)	MLO-Y4 mouse osteocyte-like cells (n.g./ n.g. (n.g.), n.g., n.g., n.g., 200,000 cells per slide/n.g.)	Oscillatory laminar	1h @ 0.5Hz	1Pa	Custom-made	Increase (RT-qPCR, Rpl13a)	1.6 (rel.)†			
Tnfrsf11b	Haugh et al. (2015)	MLO-Y4 mouse osteocytes (n.g./ n.g. (n.g.), n.g., n.g., n.g., 200000 cells per slide/n.g.)	Oscillatory laminar	1h @ 1Hz	1Pa	Custom-made	Decrease (RT-qPCR, GAPDH)	0.9 (FC)†			
	Li et al. (2019)	Cells of the osteocytic line MLOY-4 (n.g./ n.g. (n.g.), n.g., n.g., n.g., n.g./n.g.)	Oscillatory laminar	2h @ 1Hz	15 dyn/cm^2	ibidi pump system (ibidi, Germany)	Increase (RT-qPCR, ribosomal protein S2)	0.3/0.12 = 2.5 (ratio-calc)*			
	Yan et al. (2018)	Osteocyte-like MLO-Y4 cell line (n.g./ n.g. (n.g.), n.g., n.g., n.g., 1×10^5 cells per mL/n.g.)	Steady laminar	2h @ n.g. (0h, 3h, 6h post-FSS incubation)	1Pa (10dyn/cm^2)	n.g.	Increase with plateau followed by decrease (RT-qPCR, GAPDH)	0h post-FSS: 2.5 (rel.) 3h post-FSS: 2.80/1.01 = 2.77 (ratio-calc)† 6h post-FSS: 0.666/1.04 = 0.64 (ratio-calc)†	Increase with plateau followed by decrease (WB, β-actin)	0h post-FSS: 1.9 (ratio)† 3h post-FSS: 1.8 (ratio)† 6h post-FSS: 0.7 (ratio)†	
	Li et al. (2013)	MLO-Y4 cells (n.g./ n.g. (n.g.), n.g., n.g., n.g., n.g./75–85%)	Steady laminar (magnetic gear pump)	0h, 0.5h, 1h, 2h, 4h, 8h, 12h, 24h / n.g.	16dyn/cm^2, 30dyn/cm^2	Custom-made parallel-plate flow chamber	16dyn/cm^2: increase (RT-qPCR, GAPDH) 30dyn/cm^2: decrease with plateau (RT-qPCR, GAPDH)	16dyn/cm^2 @ 24h: 3.6 (rel.)† 30dyn/cm^2 @ 2h: 0.4 (rel.)† 30dyn/cm^2 @ 8h: 0.5 (rel.)† 30dyn/cm^2 @ 24h: 0.4 (rel.)†			16dyn/cm^2 (physiological levels), 30dyn/cm^2 (high levels)
	Kulkarni et al. (2012a)	MLO-Y4 osteocytes (n.g./ n.g. (n.g.), n.g., n.g., P30-31, 2×10^4 cells per cm^2/n.g.)	Pulsatile laminar	1h @ 5Hz	Mean FSS: 0.7Pa, pulse amplitude of 0.3Pa, peak SS: 8.4Pa/s	Custom-made	Increase (RT-qPCR, GAPDH)	26.1/18 = 1.45 (ratio-calc)†			

Official gene symbol or metabolite abbreviation	Reference	Cell type (age/ number and sex of donor (health status), tooth type, isolation method, passages used, cell density/confluency)	Flow type (Steady laminar, Pulsatile laminar, or Oscillatory laminar)	FSS duration and frequency	FSS magnitude	FSS apparatus	Gene expression: Increase, decrease, no change (method w/ reference gene); methods: RT-qPCR, sqPCR, northern hybridization	Gene expression: when it reaches peak and peak's magnitude (fold change; relative gene expression; times or ratio; unclear = ?)	Protein expression: Increase, decrease, no change (method w/reference); methods: ELISA, WB, RIA, EMSA, IF	Protein expression: When it reaches peak and peak's magnitude (times or ratio; unclear = ?)	Remarks including fluorescent microscopy assay
	Fahlgren et al. (2018)	MLO-Y4 osteocytes (n.g./ n.g. (n.g.), n.g., n.g., P31–33, 1.3×10^3 cells/cm^2 per slide/n.g.)	Pulsatile Laminar (SP: square wave; PL: sinusoidal wave)	60min / (1Hz, 5Hz)	2.9±2.9Pa @ 1Hz square wave (SP); 0.7±0.7Pa @ 5Hz, sinusoidal wave (PL); Unloaded	Custom-made	SP/PL: decrease SP/unloading increase PL/unloading: increase (RT-qPCR, S18)	SP/PL: 0.89/1.00 = 0.89 (ratio-calc)† SP/unloading: 0.89/0.8 = 1.1 (ratio-calc)† PL/unloading: 1.00/0.8 = 1.25 (ratio-calc)†	ELISA: SP/PL: increase SP/unloading increase PL/unloading: decrease	SP/PL: 7.128pg/mL; 7.128/0.957 = 7.4 (ratio-calc)† SP/unloading: 7.1pg/mL; 7.128/1.915 = 3.7 (ratio-calcL)† PL/unloading: 0.957pg/mL; 0.957/1.915 = 0.5 (ratio-calc)†	SP (supraphysiological loading), PL (physiological loading)
	González et al. (2017)	Mouse osteocytes (MLO-Y4 cell line) (n.g./ n.g. (n.g.), n.g., n.g., n.g., n.g. n.g./n.g.)	Pulsatile laminar	30min/n.g.	10dyn/cm^2 (8.5cm/s)	FlexCell Streamer	Increase (RT-qPCR, 18S)	11.5/3.3 = 3.5 (ratio-calc)†			
	Bakker et al. (2013b)	MLO-Y4 osteocytes (n.g./ n.g. (n.g.), n.g., n.g., P30-38, 2×10^4 cells per cm^2/n.g.)	Pulsatile laminar	60min @ 5Hz	0.7±0.3Pa	Custom-made	Increase (RT-qPCR, GAPDH)	1.99 (rel.)†			
	Kulkarni et al. (2010)	MLO-Y4 osteocytes (n.g./ n.g. (n.g.), n.g., n.g., P30-31, 1×10^3 cells per cm^2/n.g.)	Pulsatile laminar	1h (sampling 0h, 1h, 6h post FSS) / 5Hz	Mean shear stress: 0.7Pa, pulse amplitude of 0.3Pa, peak SS of 8.4Pa/s	Custom-made	Increase (RT-qPCR, GAPDH)	0h post FSS: 44.97/20.7 = 2.2 (ratio-calc)†			
	Liao et al. (2017)	MLO-Y4 osteocytes (n.g./ n.g. (n.g.), n.g., n.g., n.g., 200000 cells per slide/80-90%)	Pulsatile laminar	2h @ 5Hz	0.7±0.3Pa @ 5Hz L-PFF: 4dyn/cm^2 H-PFF: 16dyn/cm^2	Streamer® Shear Stress Device (Flexcell)	Increase (RT-qPCR, GAPDH)	L-PFF: 1.6 (rel.)† H-PFF: 1.4 (rel.)†	Increase (ELISA)	L-PFF: 4 (ratio)† H-PFF: 3.0 (ratio)†	
	Xu et al. (2014)	MLO-Y4 cell line (n.g./ n.g. (n.g.), n.g., n.g., n.g., 2.2×10^6 per slide/n.g.)	Oscillatory laminar	2h @ 1Hz	1Pa (10dyn/cm^2)	Custom-made			Increase (Griess, NO_2^-)	11.8μM;11.8/4.9 = 2.4 (ratio-calc)†	
Nitric oxide	Deepak et al. (2017)	Murine MLO-Y4 osteocyte-like cells (n.g./ n.g. (n.g.), n.g., n.g., n.g., 4x10^4 cells per slide/n.g.)	Oscillatory laminar	1h @ 0.5Hz (24h post-PFF incubation)	1Pa	Custom-made			Increase (coloric assay)	257.1/ 102.3 = 2.5 (ratio-calc)†	BioVision coloric assay
	Santos et al. (2010)	MLO-Y4 osteocytes (n.g./ n.g. (n.g.), n.g., n.g., n.g., 2.75×10^5 cells per slide/n.g.)	Pulsatile laminar	0min, 5min, 10min, 15min@ 5Hz	0.7±0.3Pa	Custom-made			Increase (Griess, NO_2^-)	Problem in control in the figure	Contradicting reporting between figure (2a) and text.
	Bakker et al. (2014)	MLO-Y4 osteocytes (n.g./ n.g. (n.g.), n.g., n.g., n.g., n.g./n.g.)	Pulsatile laminar	60min (sampling 5min and 60min during FSS) / 5Hz	0.7±0.3Pa	Custom-made			Increase (Griess, NO_2^-)	5min: 1.8-fold increase 60min: 4.5-fold increase	
	Kulkarni et al. (2012b)	MLO-Y4 osteocytes (n.g./ n.g. (n.g.), n.g., n.g., n.g., 2×10^5/n.g.)	Pulsatile laminar	1h @ 5Hz, (samples taken at 5min, 15min, 60min during FSS)	0.7Pa (pulse amplitude of 0.3Pa, peak stress rate of 8.4Pa/s)	Custom-made			Increase (Griess, NO_2-)	5min: 3.65 (ratio)†	
	Rath et al. (2010)	Osteocyte-like MLO-Y4 cells (n.g./ n.g. (n.g.), n.g., n.g., n.g., n.g./70–80%)	Pulsatile laminar	n.g./n.g.	2dyn/cm^2, 8dyn/cm^2, 16dyn/cm^2	Parallel plate, live-cell micro-observation chamber (Focht Chamber System 2, Bioptechs Inc., Butler, PA, USA)			DAR-4M fluorescence: increase		
	Bakker et al. (2009)	MLO-Y4 osteocytes (n.g./ n.g. (n.g.), n.g., n.g., P30-35, 2×10^4 cells per cm^2/n.g.)	Pulsatile laminar	30min @ 5Hz	0.7±0.3Pa	Custom-made			Increase (Griess, NO_2^-)	30 min: 16 nmol/μg DNA; 16/2.8 = 5.7 (ratio-calc)†	
	Juffer et al. (2012)	MLO-Y4 osteocytes (n.g./ n.g. (n.g.), n.g., n.g., n.g., 3x10^5 cells per glass slide /n.g.)	Pulsatile laminar	15min / 5Hz	22Pa/s; 44Pa/s	Custom-made			Increase (Griess, NO_2^-)	15min: 8.6nmole; 8.6/2.3 = 3.7 (ratio-calc)†	22Pa/s ("low shear stress"); 44Pa/s ("high shear stress")
	Santos et al. (2009)	MLO-Y4 osteocytes (n.g./ n.g. (n.g.), n.g., n.g., n.g., 2×10^5 cells per slide/n.g.)	Pulsatile laminar	1h / 5Hz	mean FSS: 0.7Pa, pulse amplitude of 0.3Pa	Custom-made			Increase (Griess, NO_2^-)	178.6nmol; 178.6/30.2 = 5.9 (ratio-calc)†	
	Fahlgren et al. (2018)	MLO-Y4 osteocytes (n.g./ n.g. (n.g.), n.g., n.g., P31–33, 1.3×10^3 cells/cm^2 per slide/n.g.)	Pulsatile Laminar (SP: square wave; PL: sinusoidal wave)	60min / (1Hz, 5Hz)	2.9±2.9Pa @ 1Hz square wave (SP); 0.7±0.7Pa @ 5Hz, sinusoidal wave (PL); Unloaded	Custom-made			Increase (Griess, NO_2^-)	5 min post-FF: SP/PL: 2.7-fold SP/unloading: 33-fold 15min post-FF: SP/PL: 2.9-fold SP/unloading: 43-fold 60 min post-FF: SP/PL: 2.3-fold SP/unloading: 58-fold	SP (supraphysiological loading), PL (physiological loading)

Official gene symbol or metabolite abbreviation	Reference	Cell type (age/ number and sex of donor (health status), tooth type, isolation method, passages used, cell density/confluency)	Flow type (Steady laminar, Pulsatile laminar, or Oscillatory laminar)	FSS duration and frequency	FSS magnitude	FSS apparatus	Gene expression: Increase, decrease, no change (method w/ reference gene); methods: RT-qPCR, sqPCR, northern hybridization	Gene expression: when it reaches peak and peak's magnitude (fold change; relative gene expression; times or ratio; unclear = ?)	Protein expression: Increase, decrease, no change (method w/reference); methods: ELISA, WB, RIA, EMSA, IF	Protein expression: When it reaches peak and peak's magnitude (times or ratio; unclear = ?)	Remarks including fluorescent microscopy assay
	Bakker et al. (2013b)	MLO-Y4 osteocytes (n.g./ n.g. (n.g.), n.g., n.g., P30-38, $2×10^4$ cells per cm^2/n.g.)	Pulsatile laminar	60min @ 5Hz	0.7±0.3Pa	Custom-made			Increase (Griess, NO_2^-)	17.5nmol/$3×10^5$ cells; 17.5/6.1 = 2.9 (ratio-calc)†	
	Shah et al. (2017)	MLO-Y4 cells (n.g./ n.g. (n.g.), n.g., n.g., n.g., $15×10^3$ or $25×10^3$ cells per slide/n.g.)	n.g.	20s, 30min, 24h/n.g.	16dyn/cm^2	n.g.	30min: increase (RT-qPCR, GAPDH)<br>24h: increase (RT-qPCR, GAPDH)	30min: 4.5 (FC)†<br>24h: 3.7 (FC)†			
Pdpn	Xu et al. (2012)	MLO-Y4 cell line (n.g./ n.g. (n.g.), n.g., n.g., n.g., $2.2×10^6$ per slide/n.g.)	Oscillatory laminar	2h @ 1Hz	1Pa (10dyn/cm^2)	Custom-made	Increase (RT-qPCR, 18S)	2h: 7.6 (FC)†			Primer-BLAST approved
	Zhang et al. (2006)	MLO-Y4 osteocyte-like cell Lines (n.g./ n.g. (n.g.), n.g., n.g., n.g., $4×10^5$/n.g.)	Steady laminar	2h (2h and 24h post FSS incubation) / n.g.	4dyn/cm^2, 16dyn/cm^2	Streamer Gold chamber (Flexcell International Corp., Hillsborough, NC)	Increase (Northern blot, GAPDH)	4dyn/cm^2 @ 2h post FSS: 4.3463/2.7408 = 1.6 (ratio-calc)†<br>16dyn/cm^2 @ 2h post FSS: 5.0/2.87 = 1.7 (ratio-calc)†			
	Geoghegan et al. (2019)	MLO-Y4 mouse osteocyte-like cells (n.g./ n.g. (n.g.), n.g., n.g., n.g., 200,000 cells per slide/n.g.)	Oscillatory laminar	1h @ 0.5Hz	1Pa	Custom-made	Increase (RT-qPCR, Rpl13a)	2.7 (rel.)†			
Ptgs2	Zhang et al. (2015)	Murine long bone osteocyte Y4 (MLO-Y4) cells (n.g./ n.g. (n.g.), n.g., n.g., n.g., 100,000 cells/slide and 150,000 cells per slide/70-80%)	Oscillatory laminar	3min-2h @ 1Hz	1Pa	Custom-made	Increase (RT-qPCR, 18S)	2h: 3.9 (rel.)†			
	Xu et al. (2012)	MLO-Y4 cell line (n.g./ n.g. (n.g.), n.g., n.g., n.g., $2.2×10^6$ per slide/n.g.)	Oscillatory laminar	2h @ 1Hz	1Pa (10dyn/cm^2)	Custom-made	Increase (RT-qPCR, 18S)	2h: 4.05 (FC)†			
	Litzenberger et al. (2010)	MLO-Y4 parental cells (n.g./ n.g. (n.g.), n.g., n.g., n.g., n.g./n.g.)	Oscillatory laminar	2h @1Hz	1Pa	Custom-made	Increase (RT-qPCR, 18S)	6.1 (rel.)†			
	Li et al. (2012)	MLO-Y4 osteocyte-like cells (n.g./ n.g. (n.g.), n.g., n.g., n.g., n.g./70%)	Oscillatory laminar	1h, 2h, 4h @ 0.5Hz, 1Hz, 2Hz, 5Hz	0.5Pa, 1Pa, 2Pa, 5Pa	Custom-made	Duration-related: increase (RT-qPCR, 18S)	2Hz, 0.5Pa@2h: 1.287(rel.)†<br>2Hz, 1.0Pa@2h: 1.5 (rel.)†<br>2Hz, 2.0Pa@2h: 2 (rel.)†<br>2Hz, 5.0Pa@2h: 2.9 (rel.)†<br>0.5Hz, 0.5Pa@4h: 2.7 (rel.)†<br>0.5Hz, 1.0Pa@4h: 2.6 (rel.)†<br>0.5Hz, 2.0Pa@4h: 3.9 (rel.)†<br>0.5Hz, 5.0Pa@4h: 4.4 (rel.)†			
	Haugh et al. (2015)	MLO-Y4 mouse osteocytes (n.g./ n.g. (n.g.), n.g., n.g., n.g., 200000 cells per slide/n.g.)	Oscillatory laminar	1h @ 1Hz	1Pa	Custom-made	Increase (RT-qPCR, GAPDH)	6.22 (FC)†			
	Xu et al. (2014)	MLO-Y4 cell line (n.g./ n.g. (n.g.), n.g., n.g., n.g., $2.2×10^6$ per slide/n.g.)	Oscillatory laminar	2h @ 1Hz	1Pa (10dyn/cm^2)	Custom-made	Increase (RT-qPCR, 18S)	2h: 1.74(FC)†			
	Li et al. (2019)	Cells of the osteocytic line MLOY-4 (n.g./ n.g. (n.g.), n.g., n.g., n.g., n.g./n.g.)	Oscillatory laminar	2h @ 1Hz	15 dyn/cm^2	ibidi pump system (ibidi, Germany)	Increase (RT-qPCR, ribosomal protein S2)	15/2 = 7.5 (ratio-calc)*			
	Liu et al. (2015)	1-MLO-Y4 osteocyte-like cells (n.g./ n.g. (n.g.), n.g., n.g., n.g., n.g./80%)	Oscillatory laminar	1h (sampling 24h post FSS) / 1Hz	2Pa	Custom-made	Increase (RT-qPCR, 18S)	24h post FSS: 1.1/0.5 = 2.2 (ratio-calc)†			
	Bakker et al. (2013b)	MLO-Y4 osteocytes (n.g./ n.g. (n.g.), n.g., n.g., P30-38, $2×10^4$ cells per cm^2/n.g.)	Pulsatile laminar	60min @ 5Hz	0.7±0.3Pa	Custom-made	Increase (RT-qPCR, GAPDH)	2.49 (rel.)†			

Official gene symbol or metabolite abbreviation	Reference	Cell type (age/ number and sex of donor (health status), tooth type, isolation method, passages used, cell density/confluency)	Flow type (Steady laminar, Pulsatile laminar, or Oscillatory laminar)	FSS duration and frequency	FSS magnitude	FSS apparatus	Gene expression: Increase, decrease, no change (method w/ reference gene); methods: RT-qPCR, sqPCR, northern hybridization	Gene expression: when it reaches peak and peak's magnitude (fold change; relative gene expression; times or ratio; unclear = ?)	Protein expression: Increase, decrease, no change (method w/reference); methods: ELISA, WB, RIA, EMSA, IF	Protein expression: When it reaches peak and peak's magnitude (times or ratio; unclear = ?)	Remarks including fluorescent microscopy assay
	Fahlgren et al. (2018)	MLO-Y4 osteocytes (n.g./ n.g. (n.g.), n.g., n.g., P31–33, 1.3×10³ cells/cm² per slide/n.g.)	Pulsatile Laminar (SP: square wave; PL: sinusoidal wave)	60min / (1Hz, 5Hz)	2.9±2.9Pa @ 1Hz square wave (SP); 0.7±0.7Pa @ 5Hz, sinusoidal wave (PL); Unloaded	Custom-made	SP/PL: decrease<br>SP/unloading: no change<br>PL/unloading: increase<br>(RT-qPCR, S18)	SP/PL: 0.81/0.99 = 0.81 (ratio-calc)<br>SP/unloading: 0.81/0.81 = 1 (ratio-calc)†<br>PL/unloading: 0.99/0.81 = 1.2 (ratio-calc)†			SP (supraphysiological loading), PL (physiological loading)
	Kamel et al. (2010)	MLO-Y4 cells (n.g./ n.g. (n.g.), n.g., n.g., P27, 5×10⁵ cells per slide/70%)	Pulsatile laminar	2h @ 0.5Hz	(2, 4, 8, 16, 24 ±0.6) dyn/cm2	Flexcell® Streamer® Shear Stress Device	Increase (RT-qPCR, GAPDH)	16dyn/cm²: 5.4 (FC)†			
	Bakker et al. (2014)	MLO-Y4 osteocytes (n.g./ n.g. (n.g.), n.g., n.g., n.g., n.g./n.g.)	Pulsatile laminar	60min / 5Hz	0.7±0.3Pa	Custom-made	Increase (RT-qPCR, GAPDH)	0.1/0.04 = 2.5 (ratio-calc)†			
	Yan et al. (2018)	Osteocyte-like MLO-Y4 cell line (n.g./ n.g. (n.g.), n.g., n.g., n.g., 1×10⁵ cells per mL/n.g.)	Steady laminar	2h @ n.g. (0h, 3h, 6h post-FSS incubation)	1Pa (10dyn/cm²)	n.g.	Increase (RT-qPCR, GAPDH)	3h post-FSS: 1.82/1.07 = 1.7 (ratio-calc)†	Increase (WB, β-actin)	6h post-FSS: 6.2/1.1 = 5.63 (ratio-calc)†	
Wnt3a	Huang et al. (2017)	Primary mouse osteocyte (5 months/ n.g./M (C57BL/6 mice), n.g., n.g., n.g., n.g./n.g.)	n.g.	2h (sampling at 0h and 24h post FSS) / n.g.	2dyn/cm²	n.g.	0h post FSS: increase<br>24h post FSS: decrease<br>(RT-qPCR, GAPDH)	0h post FSS: 2 (rel.)†<br>24h post FSS: 0.92 (rel.)†			
	Santos et al. (2009)	MLO-Y4 osteocytes (n.g./ n.g. (n.g.), n.g., n.g., n.g., 2×10⁵ cells per slide/n.g.)	Pulsatile laminar	1h (1h, 3h post-PFF incubation) / 5Hz	mean FSS: 0.7Pa, pulse amplitude of 0.3Pa	Custom-made	Decrease then increase (RT-qPCR, GAPDH)	0.5h post-PFF: 0.5 (rel.)†<br>1h post-PFF: 1.7 (rel.)†			

References

Alford AI, Jacobs CR, Donahue HJ (2003). Oscillating fluid flow regulates gap junction communication in osteocytic MLO-Y4 cells by an ERK1/2 MAP kinase-dependent mechanism. Bone; 33(1):64-70.

Bakker AD, Soejima K, Klein-Nulend J, Burger EH (2001). The production of nitric oxide and prostaglandin E(2) by primary bone cells is shear stress dependent. J Biomech; 34(5):671-7.

Bakker AD, Joldersma M, Klein-Nulend J, Burger EH (2003a). Interactive effects of PTH and mechanical stress on nitric oxide and PGE2 production by primary mouse osteoblastic cells. Am J Physiol Endocrinol Metab; 285(3):E608-13.

Bakker AD, Klein-Nulend J, Burger EH (2003b). Mechanotransduction in bone cells proceeds via activation of COX-2, but not COX-1. Biochem Biophys Res Commun; 305(3):677-83.

Bakker AD, Silva VC, Krishnan R, Bacabac RG, Blaauboer ME, Lin YC, Marcantonio RA, Cirelli JA, Klein-Nulend J (2009). Tumor necrosis factor alpha and interleukin-1beta modulate calcium and nitric oxide signaling in mechanically stimulated osteocytes. Arthritis Rheum; 60(11):3336-45.

Bakker AD, Huesa C, Hughes A, Aspden RM, van't Hof RJ, Klein-Nulend J, Helfrich MH (2013a). Endothelial nitric oxide synthase is not essential for nitric oxide production by osteoblasts subjected to fluid shear stress in vitro. Calcif Tissue Int; 92(3):228-39.

Bakker AD, Zandieh-Doulabi B, Klein-Nulend J (2013b). Strontium ranelate affects signaling from mechanically-stimulated osteocytes towards osteoclasts and osteoblasts. Bone; 53(1):112-9.

Bakker AD, Kulkarni RN, Klein-Nulend J, Lems WF (2014). IL-6 alters osteocyte signaling toward osteoblasts but not osteoclasts. J Dent Res; 93(4):394-9.

Batra N, Riquelme MA, Burra S, Kar R, Gu S, Jiang JX (2014). Direct regulation of osteocytic connexin 43 hemichannels through AKT kinase activated by mechanical stimulation. J Biol Chem; 289(15):10582-91.

Becquart P, Cruel M, Hoc T, Sudre L, Pernelle K, Bizios R, Logeart-Avramoglou D, Petite H, Bensidhoum M (2016). Human mesenchymal stem cell responses to hydrostatic pressure and shear stress. Eur Cell Mater; 31:160-73.

Callewaert F, Bakker A, Schrooten J, Van Meerbeek B, Verhoeven G, Boonen S, Vanderschueren D (2010). Androgen receptor disruption increases the osteogenic response to mechanical loading in male mice. J Bone Miner Res; 25(1):124-31.

Castillo AB, Triplett JW, Pavalko FM, Turner CH (2014). Estrogen receptor-β regulates mechanical signaling in primary osteoblasts. Am J Physiol Endocrinol Metab; 306(8):E937-44.

Celil Aydemir AB, Lee S, Won Kim D, Gardner TR, Prince D, Mok Ahn J, Lee FY (2007). Nuclear factor of activated T cell mediates proinflammatory gene expression in response to mechanotransduction. Ann N Y Acad Sci; 1117:138-42.

Celil Aydemir AB, Minematsu H, Gardner TR, Kim KO, Ahn JM, Lee FY (2010). Nuclear factor of activated T cells mediates fluid shear stress- and tensile strain-induced Cox2 in human and murine bone cells. Bone; 46(1):167-75.

Charoenpong H, Osathanon T, Pavasant P, Limjeerajarus N, Keawprachum B, Limjeerajarus CN, Cheewinthamrongrod V, Palaga T, Lertchirakarn V, Ritprajak P (2019). Mechanical stress induced S100A7 expression in human dental pulp cells to augment osteoclast differentiation. Oral Dis; 25(3):812-821.

Chen JC, Chua M, Bellon RB, Jacobs CR (2015). Epigenetic changes during mechanically induced osteogenic lineage commitment. J Biomech Eng; 137(2):020902.

Cheng B, Zhao S, Luo J, Sprague E, Bonewald LF, Jiang JX (2001). Expression of functional gap junctions and regulation by fluid flow in osteocyte-like MLO-Y4 cells. J Bone Miner Res; 16(2):249-59.

Cherian PP, Siller-Jackson AJ, Gu S, Wang X, Bonewald LF, Sprague E, Jiang JX (2005). Mechanical strain opens connexin 43 hemichannels in osteocytes: a novel mechanism for the release of prostaglandin. Mol Biol Cell; 16(7):3100-6.
de Castro LF, Maycas M, Bravo B, Esbrit P, Gortazar A (2015). VEGF Receptor 2 (VEGFR2) Activation Is Essential for Osteocyte Survival Induced by Mechanotransduction. J Cell Physiol; 230(2):278-85.
Deepak V, Kayastha P, McNamara LM (2017). Estrogen deficiency attenuates fluid flow-induced [Ca(2+)](i) oscillations and mechanoresponsiveness of MLO-Y4 osteocytes. FASEB J; 31(7):3027-3039.
Fahlgren A, Bratengeier C, Semeins CM, Klein-Nulend J, Bakker AD (2018). Supraphysiological loading induces osteocyte-mediated osteoclastogenesis in a novel in vitro model for bone implant loosening. J Orthop Res; 36(5):1425-1434.
Genetos DC, Kephart CJ, Zhang Y, Yellowley CE, Donahue HJ (2007). Oscillating fluid flow activation of gap junction hemichannels induces ATP release from MLO-Y4 osteocytes. J Cell Physiol; 212(1):207-14.
Geoghegan IP, Hoey DA, McNamara LM (2019). Estrogen deficiency impairs integrin α(v)β(3)-mediated mechanosensation by osteocytes and alters osteoclastogenic paracrine signalling. Sci Rep; 9(1):4654.
González Á, García de Durango C, Alonso V, Bravo B, Rodríguez de Gortázar A, Wells A, Forteza J, Vidal-Vanaclocha F (2017). Distinct Osteomimetic Response of Androgen-Dependent and Independent Human Prostate Cancer Cells to Mechanical Action of Fluid Flow: Prometastatic Implications. Prostate; 77(3):321-333.
Haugh MG, Vaughan TJ, McNamara LM (2015). The role of integrin α(V)β(3) in osteocyte mechanotransduction. J Mech Behav Biomed Mater; 42:67-75.
Hoey DA, Tormey S, Ramcharan S, O'Brien FJ, Jacobs CR (2012). Primary cilia-mediated mechanotransduction in human mesenchymal stem cells. Stem Cells; 30(11):2561-70.
Hu K, Sun H, Gui B, Sui C (2017). TRPV4 functions in flow shear stress induced early osteogenic differentiation of human bone marrow mesenchymal stem cells. Biomed Pharmacother; 91:841-848.
Huang J, Romero-Suarez S, Lara N, Mo C, Kaja S, Brotto L, Dallas SL, Johnson ML, Jähn K, Bonewald LF, Brotto M (2017). Crosstalk between MLO-Y4 osteocytes and C2C12 muscle cells is mediated by the Wnt/β-catenin pathway. JBMR Plus; 1(2):86-100.
Jing D, Lu XL, Luo E, Sajda P, Leong PL, Guo XE (2013). Spatiotemporal properties of intracellular calcium signaling in osteocytic and osteoblastic cell networks under fluid flow. Bone; 53(2):531-40.
Joldersma M, Burger EH, Semeins CM, Klein-Nulend J (2000). Mechanical stress induces COX-2 mRNA expression in bone cells from elderly women. J Biomech; 33(1):53-61.
Joldersma M, Klein-Nulend J, Oleksik AM, Heyligers IC, Burger EH (2001). Estrogen enhances mechanical stress-induced prostaglandin production by bone cells from elderly women. Am J Physiol Endocrinol Metab; 280(3):E436-42.
Juffer P, Jaspers RT, Lips P, Bakker AD, Klein-Nulend J (2012). Expression of muscle anabolic and metabolic factors in mechanically loaded MLO-Y4 osteocytes. Am J Physiol Endocrinol Metab; 302(4):E389-95.
Kamel MA, Picconi JL, Lara-Castillo N, Johnson ML (2010). Activation of β-catenin signaling in MLO-Y4 osteocytic cells versus 2T3 osteoblastic cells by fluid flow shear stress and PGE2: Implications for the study of mechanosensation in bone. Bone; 47(5):872-81.
Kapur S, Amoui M, Kesavan C, Wang X, Mohan S, Baylink DJ, Lau KH (2010). Leptin receptor (Lepr) is a negative modulator of bone mechanosensitivity and genetic variations in Lepr may contribute to the differential osteogenic response to mechanical stimulation in the C57BL/6J and C3H/HeJ pair of mouse strains. J Biol Chem; 285(48):37607-18.
Klein-Nulend J, Semeins CM, Burger EH (1996). Prostaglandin mediated modulation of transforming growth factor-beta metabolism in primary mouse osteoblastic cells in vitro. J Cell Physiol; 168(1):1-7.
Klein-Nulend J, Burger EH, Semeins CM, Raisz LG, Pilbeam CC (1997). Pulsating fluid flow stimulates prostaglandin release and inducible prostaglandin G/H synthase mRNA expression in primary mouse bone cells. J Bone Miner Res; 12(1):45-51.
Klein-Nulend J, Helfrich MH, Sterck JG, MacPherson H, Joldersma M, Ralston SH, Semeins CM, Burger EH (1998). Nitric oxide response to shear stress by human bone cell cultures is endothelial nitric oxide synthase dependent. Biochem Biophys Res Commun; 250(1):108-14.
Klein-Nulend J, Sterck JG, Semeins CM, Lips P, Joldersma M, Baart JA, Burger EH (2002). Donor age and mechanosensitivity of human bone cells. Osteoporos Int; 13(2):137-46.
Kraft DC, Bindslev DA, Melsen B, Abdallah BM, Kassem M, Klein-Nulend J (2010). Mechanosensitivity of dental pulp stem cells is related to their osteogenic maturity. Eur J Oral Sci; 118(1):29-38.
Kraft DC, Bindslev DA, Melsen B, Klein-Nulend J (2011). Human dental pulp cells exhibit bone cell-like responsiveness to fluid shear stress. Cytotherapy; 13(2):214-26.
Kulkarni RN, Bakker AD, Everts V, Klein-Nulend J (2010). Inhibition of osteoclastogenesis by mechanically loaded osteocytes: involvement of MEPE. Calcif Tissue Int; 87(5):461-8.
Kulkarni RN, Bakker AD, Everts V, Klein-Nulend J (2012a). Mechanical loading prevents the stimulating effect of IL-1β on osteocyte-modulated osteoclastogenesis. Biochem Biophys Res Commun; 420(1):11-6.
Kulkarni RN, Bakker AD, Gruber EV, Chae TD, Veldkamp JB, Klein-Nulend J, Everts V (2012b). MT1-MMP modulates the mechanosensitivity of osteocytes. Biochem Biophys Res Commun; 417(2):824-9.
Kuo YC, Chang TH, Hsu WT, Zhou J, Lee HH, Hui-Chun Ho J, Chien S, Lee OK (2015). Oscillatory shear stress mediates directional reorganization of actin cytoskeleton and alters differentiation propensity of mesenchymal stem cells. Stem Cells; 33(2):429-42.
Lau KH, Kapur S, Kesavan C, Baylink DJ (2006). Up-regulation of the Wnt, estrogen receptor, insulin-like growth factor-I, and bone morphogenetic protein pathways in C57BL/6J osteoblasts as opposed to C3H/HeJ osteoblasts in part contributes to the differential anabolic response to fluid shear. J Biol Chem; 281(14):9576-88.
Lee HJ, Diaz MF, Ewere A, Olson SD, Cox CS, Jr., Wenzel PL (2017). Focal adhesion kinase signaling regulates anti-inflammatory function of bone marrow mesenchymal stromal cells induced by biomechanical force. Cell Signal; 38:1-9.
Li J, Liu D, Ke HZ, Duncan RL, Turner CH (2005). The P2X7 nucleotide receptor mediates skeletal mechanotransduction. J Biol Chem; 280(52):42952-9.
Li J, Rose E, Frances D, Sun Y, You L (2012). Effect of oscillating fluid flow stimulation on osteocyte mRNA expression. J Biomech; 45(2):247-51.
Li X, Liu C, Li P, Li S, Zhao Z, Chen Y, Huo B, Zhang D (2013). Connexin 43 is a potential regulator in fluid shear stress-induced signal transduction in osteocytes. J Orthop Res; 31(12):1959-65.
Li X, Han L, Nookaew I, Mannen E, Silva MJ, Almeida M, Xiong J (2019). Stimulation of Piezo1 by mechanical signals promotes bone anabolism. Elife; 8.
Li YJ, Batra NN, You L, Meier SC, Coe IA, Yellowley CE, Jacobs CR (2004). Oscillatory fluid flow affects human marrow stromal cell proliferation and differentiation. J Orthop Res; 22(6):1283-9.
Liao C, Cheng T, Wang S, Zhang C, Jin L, Yang Y (2017). Shear stress inhibits IL-17A-mediated induction of osteoclastogenesis via osteocyte pathways. Bone; 101:10-20.
Lim KT, Kim J, Seonwoo H, Chang JU, Choi H, Hexiu J, Cho WJ, Choung PH, Chung JH (2013). Enhanced osteogenesis of human alveolar bone-derived mesenchymal stem cells for tooth tissue engineering using fluid shear stress in a rocking culture method. Tissue Eng Part C Methods; 19(2):128-45.
Lim KT, Hexiu J, Kim J, Seonwoo H, Choung PH, Chung JH (2014). Synergistic effects of orbital shear stress on in vitro growth and osteogenic differentiation of human alveolar bone-derived mesenchymal stem cells. Biomed Res Int; 2014:316803.
Litzenberger JB, Kim JB, Tummala P, Jacobs CR (2010). Beta1 integrins mediate mechanosensitive signaling pathways in osteocytes. Calcif Tissue Int; 86(4):325-32.
Liu C, Zhang X, Wu M, You L (2015). Mechanical loading up-regulates early remodeling signals from osteocytes subjected to physical damage. J Biomech; 48(16):4221-8.
Lu XL, Huo B, Chiang V, Guo XE (2012a). Osteocytic network is more responsive in calcium signaling than osteoblastic network under fluid flow. J Bone Miner Res; 27(3):563-74.
Lu XL, Huo B, Park M, Guo XE (2012b). Calcium response in osteocytic networks under steady and oscillatory fluid flow. Bone; 51(3):466-73.
Maycas M, Ardura JA, de Castro LF, Bravo B, Gortázar AR, Esbrit P (2015). Role of the Parathyroid Hormone Type 1 Receptor (PTH1R) as a Mechanosensor in Osteocyte Survival. J Bone Miner Res; 30(7):1231-44.
Maycas M, Portolés MT, Matesanz MC, Buendía I, Linares J, Feito MJ, Arcos D, Vallet-Regí M, Plotkin LI, Esbrit P, Gortázar AR (2017). High glucose alters the secretome of mechanically stimulated osteocyte-like cells affecting osteoclast precursor recruitment and differentiation. J Cell Physiol; 232(12):3611-3621.
McGarry JG, Klein-Nulend J, Mullender MG, Prendergast PJ (2005). A comparison of strain and fluid shear stress in stimulating bone cell responses--a computational and experimental study. FASEB J; 19(3):482-4.

Mehrotra M, Saegusa M, Wadhwa S, Voznesensky O, Peterson D, Pilbeam C (2006). Fluid flow induces Rankl expression in primary murine calvarial osteoblasts. J Cell Biochem; 98(5):1271-83.

Middleton K, Kondiboyina A, Borrett M, Cui Y, Mei X, You L (2018). Microfluidics approach to investigate the role of dynamic similitude in osteocyte mechanobiology. J Orthop Res; 36(2):663-671.

Qi L, Zhang Y (2014). The microRNA 132 regulates fluid shear stress-induced differentiation in periodontal ligament cells through mTOR signaling pathway. Cell Physiol Biochem; 33(2):433-45.

Rath AL, Bonewald LF, Ling J, Jiang JX, Van Dyke ME, Nicolella DP (2010). Correlation of cell strain in single osteocytes with intracellular calcium, but not intracellular nitric oxide, in response to fluid flow. J Biomech; 43(8):1560-4.

Reilly GC, Haut TR, Yellowley CE, Donahue HJ, Jacobs CR (2003). Fluid flow induced PGE2 release by bone cells is reduced by glycocalyx degradation whereas calcium signals are not. Biorheology; 40(6):591-603.

Ren J, Wang XH, Wang GC, Wu JH (2013). 17β estradiol regulation of connexin 43-based gap junction and mechanosensitivity through classical estrogen receptor pathway in osteocyte-like MLO-Y4 cells. Bone; 53(2):587-96.

Riddle RC, Taylor AF, Genetos DC, Donahue HJ (2006). MAP kinase and calcium signaling mediate fluid flow-induced human mesenchymal stem cell proliferation. Am J Physiol Cell Physiol; 290(3):C776-84.

Riddle RC, Taylor AF, Rogers JR, Donahue HJ (2007). ATP release mediates fluid flow-induced proliferation of human bone marrow stromal cells. J Bone Miner Res; 22(4):589-600.

Riquelme MA, Gu S, Hua R, Jiang JX (2021). Mechanotransduction via the coordinated actions of integrins, PI3K signaling and Connexin hemichannels. Bone Res; 9(1):8.

Salvi JD, Lim JY, Donahue HJ (2010). Increased mechanosensitivity of cells cultured on nanotopographies. J Biomech; 43(15):3058-62.

Santos A, Bakker AD, Zandieh-Doulabi B, Semeins CM, Klein-Nulend J (2009). Pulsating fluid flow modulates gene expression of proteins involved in Wnt signaling pathways in osteocytes. J Orthop Res; 27(10):1280-7.

Santos A, Bakker AD, Zandieh-Doulabi B, de Blieck-Hogervorst JM, Klein-Nulend J (2010). Early activation of the beta-catenin pathway in osteocytes is mediated by nitric oxide, phosphatidyl inositol-3 kinase/Akt, and focal adhesion kinase. Biochem Biophys Res Commun; 391(1):364-9.

Santos A, Bakker AD, Willems HM, Bravenboer N, Bronckers AL, Klein-Nulend J (2011). Mechanical loading stimulates BMP7, but not BMP2, production by osteocytes. Calcif Tissue Int; 89(4):318-26.

Seref-Ferlengez Z, Maung S, Schaffler MB, Spray DC, Suadicani SO, Thi MM (2016). P2X7R-Panx1 Complex Impairs Bone Mechanosignaling under High Glucose Levels Associated with Type-1 Diabetes. PLoS One; 11(5):e0155107.

Shah KM, Orton P, Mani N, Wilkinson JM, Gartland A (2017). Osteocyte physiology and response to fluid shear stress are impaired following exposure to cobalt and chromium: Implications for bone health following joint replacement. J Orthop Res; 35(8):1716-1723.

Soejima K, Klein-Nulend J, Semeins CM, Burger EH (2001). Different responsiveness of cells from adult and neonatal mouse bone to mechanical and biochemical challenge. J Cell Physiol; 186(3):366-70.

Sonam S, Sathe SR, Yim EK, Sheetz MP, Lim CT (2016). Cell contractility arising from topography and shear flow determines human mesenchymal stem cell fate. Scientific Reports; 6:20415.

Sterck JG, Klein-Nulend J, Lips P, Burger EH (1998). Response of normal and osteoporotic human bone cells to mechanical stress in vitro. Am J Physiol; 274(6):E1113-20.

Tang M, Peng Z, Mai Z, Chen L, Mao Q, Chen Z, Chen Q, Liu L, Wang Y, Ai H (2014). Fluid shear stress stimulates osteogenic differentiation of human periodontal ligament cells via the extracellular signal-regulated kinase 1/2 and p38 mitogen-activated protein kinase signaling pathways. J Periodontol; 85(12):1806-13.

Thi MM, Kojima T, Cowin SC, Weinbaum S, Spray DC (2003). Fluid shear stress remodels expression and function of junctional proteins in cultured bone cells. Am J Physiol Cell Physiol; 284(2):C389-403.

Thi MM, Suadicani SO, Spray DC (2010). Fluid flow-induced soluble vascular endothelial growth factor isoforms regulate actin adaptation in osteoblasts. J Biol Chem; 285(40):30931-41.

Thi MM, Islam S, Suadicani SO, Spray DC (2012). Connexin43 and pannexin1 channels in osteoblasts: who is the "hemichannel"? J Membr Biol; 245(7):401-9.

van der Meijden K, Bakker AD, van Essen HW, Heijboer AC, Schulten EA, Lips P, Bravenboer N (2016). Mechanical loading and the synthesis of 1,25(OH)2D in primary human osteoblasts. J Steroid Biochem Mol Biol; 156:32-9.

Wang S, Li S, Hu M, Huo B (2019). Calcium response in bone cells at different osteogenic stages under unidirectional or oscillatory flow. Biomicrofluidics; 13(6):064117.

Xia X, Batra N, Shi Q, Bonewald LF, Sprague E, Jiang JX (2010). Prostaglandin promotion of osteocyte gap junction function through transcriptional regulation of connexin 43 by glycogen synthase kinase 3/beta-catenin signaling. Mol Cell Biol; 30(1):206-19.

Xing Y, Gu Y, Bresnahan JJ, Paul EM, Donahue HJ, You J (2014). The roles of P2Y2 purinergic receptors in osteoblasts and mechanotransduction. PLoS One; 9(9):e108417.

Xu H, Zhang J, Wu J, Guan Y, Weng Y, Shang P (2012). Oscillatory fluid flow elicits changes in morphology, cytoskeleton and integrin-associated molecules in MLO-Y4 cells, but not in MC3T3-E1 cells. Biol Res; 45(2):163-9.

Xu H, Guan Y, Wu J, Zhang J, Duan J, An L, Shang P (2014). Polycystin 2 is involved in the nitric oxide production in responding to oscillating fluid shear in MLO-Y4 cells. J Biomech; 47(2):387-91.

Yan Z, Wang P, Wu J, Feng X, Cai J, Zhai M, Li J, Liu X, Jiang M, Luo E, Jing D (2018). Fluid shear stress improves morphology, cytoskeleton architecture, viability, and regulates cytokine expression in a time-dependent manner in MLO-Y4 cells. Cell Biol Int; 42(10):1410-1422.

Yang Z, Bidwell JP, Young SR, Gerard-O'Riley R, Wang H, Pavalko FM (2010). Nmp4/CIZ inhibits mechanically induced beta-catenin signaling activity in osteoblasts. J Cell Physiol; 223(2):435-41.

Yang Z, Tan S, Shen Y, Chen R, Wu C, Xu Y, Song Z, Fu Q (2015). Inhibition of FSS-induced actin cytoskeleton reorganization by silencing LIMK2 gene increases the mechanosensitivity of primary osteoblasts. Bone; 74:182-90.

Yourek G, McCormick SM, Mao JJ, Reilly GC (2010). Shear stress induces osteogenic differentiation of human mesenchymal stem cells. Regen Med; 5(5):713-24.

Yuan L, Sakamoto N, Song G, Sato M (2012). Migration of human mesenchymal stem cells under low shear stress mediated by mitogen-activated protein kinase signaling. Stem Cells Dev; 21(13):2520-30.

Zhang JN, Zhao Y, Liu C, Han ES, Yu X, Lidington D, Bolz SS, You L (2015). The role of the sphingosine-1-phosphate signaling pathway in osteocyte mechanotransduction. Bone; 79:71-8.

Zhang K, Barragan-Adjemian C, Ye L, Kotha S, Dallas M, Lu Y, Zhao S, Harris M, Harris SE, Feng JQ, Bonewald LF (2006). E11/gp38 selective expression in osteocytes: regulation by mechanical strain and role in dendrite elongation. Mol Cell Biol; 26(12):4539-52.

Zheng L, Chen L, Chen Y, Gui J, Li Q, Huang Y, Liu M, Jia X, Song W, Ji J, Gong X, Shi R, Fan Y (2016). The effects of fluid shear stress on proliferation and osteogenesis of human periodontal ligament cells. J Biomech; 49(4):572-9.
