# Supplementary material for: Fluid flow shear stress and tissue remodeling—an orthodontic perspective: evidence synthesis and differential gene expression network analysis
Source: Front Bioeng Biotechnol. 2023 Sep 18;11:1256825. doi: 10.3389/fbioe.2023.1256825 (PMC10545883; doi:10.3389/fbioe.2023.1256825)
Supplement: Supplementary file 8 [file DataSheet8.pdf]

Supplement 8

"Fluid Flow Shear Stress and Tissue Remodeling – an Orthodontic Perspective: Evidence Synthesis and Differential Gene Expression Network analysis"

Pre-ranked Gene Set Enrichment Analysis (Pre-ranked GSEA)

Table of contents

8 Pre-ranked Gene Set Enrichment Analysis 2

8.1 General information 2

8.2 Description of the differential gene expression analysis workflows 3
8.2.1 Affymetrix microarrays (E-MEXP-1923, GSE42874) 3
8.2.2 Illumina BeadArrays (GSE82269) 3
8.2.3 RNA-seq data (SRP060567/GSE70667, SRP212008) 3

8.3 References 5

8.4 Human bone marrow mesenchymal stem cells (MSCs) 6
8.4.1 Summary 6
8.4.2 GSE82269 and E-MEXP-1923: easyVizR comparison of the pre-ranked easyGSEA results 7

8.5 Mouse MLO-Y4 osteocyte-like cell line 10
8.5.1 Summary 10
8.5.2 GSE42874, GSE70667 and SRP212008: easyVizR comparison of the pre-ranked easyGSEA results 11

8 Pre-ranked Gene Set Enrichment Analysis

8.1 General information

During data extraction, five different human and mouse high-throughput gene expression studies with raw data available in public repositories were identified (**Table 8.1**). In 2 studies, fluid shear stress (FSS) was applied to human bone marrow-derived mesenchymal stem cells (hsMSC) (Diaz et al. 2017; Glossop and Cartmell 2009). In the remaining 3 studies, FSS was applied to the mouse osteocytic-cell line MLO-Y4 (Govey et al. 2014; Govey et al. 2015; Li et al. 2019). The minimum requirement for selection was, that the experimental condition (FSS) and the corresponding control condition were represented each by at least 2 samples (microarrays or RNA-seq reactions).

The raw data was downloaded from the specific repositories and analyzed separately with workflows specific to the platform used (microarray, RNA-seq). Pre-ranked gene lists were exported, and gene set enrichment analysis (GSEA) was applied using easyGSEA (Cheng et al. 2021). Gene set definitions from KEGG (Du et al. 2014), GeneOntology/Biological Process (Thomas 2017), and WikiPathways (Martens et al. 2021; Pico et al. 2008) were selected to enhance comparability with the over-representation analysis of the species-specific gene lists.

For each cell type (human MSCs, mouse MLO-Y4), common features were identified separately. Afterwards, the easyVizR website was used to compare the cell-type-specific results from easyGSEA (Cheng et al. 2021). In both comparisons, general pathways related to cancer and/or infectious diseases like "*Chagas disease*" (hsa05142) or "*Pertussis*" (hsa05133) were removed, and their accession numbers were reported.

***Table 8.1**. Summary of the high-throughput gene expression studies analyzed with pre-ranked GSEA.*

Study Id	Reference	Platform	Type	Cell type	FSS parameters	Re-analyzed samples
E-MEXP-1923 (AE)	Glossop and Cartmell (2009)	Affymetrix GeneChip Human Genome U133 Plus 2.0 [HG-U133_Plus_2]	Microarray (probe)	Human bone marrow MSCs from 1 female donor (23 yrs old; commercial source)	FSS: 1 dyn/cm^2 for 1 h and 2 h post-FSS; Control: sham treated	2 samples FSS, 2 samples control
GSE82269 (GEO)	Diaz et al. (2017)	Illumina HumanHT-12 V4.0 Expression BeadChip (GPL10558)	Microarray (BeadArray)	Human bone marrow MSCs from 3 donors (1 female, 20yrs; 2 male, 22 and 25 yrs; commercial source)	FSS: 15 dyn/cm^2 for 6 h; Control: sham treated	3 samples FSS, 3 samples control (batch correction: donor)
GSE42874 (GEO)	Govey et al. (2014)	Affymetrix Mouse Genome 430A 2.0 Array (GPL8321)	Microarray (probe)	Mouse MLO-Y4 osteocyte-like cell line	FSS: sinusoidally oscillating FF w/ peak shear stress of 10 dyn/cm^2 @ 1 Hz for 2 h and post-FSS incubation for 0/2/8/24 h; Control: paired sham	3 samples FSS (2h FSS and 2h post-FSS), 3 samples control
GSE70667 (recount3: SRP060567)	Govey et al. (2015)	Illumina HiSeq 2500 (Mus musculus) (GPL17021)	RNA-seq	Mouse MLO-Y4 osteocyte-like cell line	FSS: sinusoidally oscillating FF w/ peak shear stress of 10 dyn/cm^2 @ 1 Hz for 2 h and post-FSS incubation for 2 h; Control: paired sham	3 samples FSS, 3 samples control
SRP212008 (recount3)	Li et al. (2019)	Illumina NextSeq 550	RNA-seq	Mouse MLO-Y4 osteocyte-like cell line	FSS: 15 dyn/cm^2 oscillatory FSS @ 1 Hz for 2 h; Controls: sham treated	3 samples FSS, 3 samples control

GEO, Gene Expression Omnibus (https://www.ncbi.nlm.nih.gov/geo/; AE, ArrayExpress (https://www.ebi.ac.uk/biostudies/arrayexpress/); recount3 study explorer (https://jhubiostatistics.shinyapps.io/recount3-study-explorer/)

8.2 Description of the differential gene expression analysis workflows

Differential gene expression analysis and the generation of the pre-ranked gene lists from each study were done using R (version 4.1.1.r80981 patched) (R Core Team 2021) running on an Apple MacBook Pro with 3.1 GHz Intel core i7, 16GB RAM, and macOS 10.14.6. RStudio IDE (version 2021.09.2.382) (RStudio Team 2022) was used for scripting. Depending on the study data, different R packages were used including those from Bioconductor version 3.14 (Huber et al. 2015). Raw data from each study were individually processed as described below.

8.2.1 Affymetrix microarrays (E-MEXP-1923, GSE42874)

CEL-files representing the raw probe-level expression data from the studies using Affymetrix microarrays were downloaded from their repositories and stored locally. Both datasets were separately analyzed. The *affy* package version 1.72.0 (Gautier et al. 2004) was used for parsing the raw data and robust multichip average (RMA) normalization. The array quality was assessed using the *arrayQualityMetrics* package version 3.50.0 (Kauffmann et al. 2009). Arrays not passing the quality check in at least 2 different tests were removed from analysis (GSE42874). Manual filtering was applied after normalization by removing the lower 10% expressed probes (Klaus and Reisenauer 2016). Differential expression analysis was evaluated with the *limma* package version 3.50.3 (Ritchie et al. 2015). First, a linear model with the experimental conditions was fitted for each gene of a dataset, and afterwards the eBayes function of the empirical Bayes method was applied. Multiple testing correction was applied using the Benjamini and Hochberg false discovery rate method implemented in the topfit function from *limma*. The ranking of the genes was calculated as logFC × -log10(*P*-value). The pre-ranked gene lists were exported for each study separately containing the gene symbols and the calculated gene ranks.

8.2.2 Illumina BeadArrays (GSE82269)

IDAT-files representing the raw data of this study were downloaded from GEO and stored locally. The *beadarray* package version 2.44.0 (Dunning et al. 2007) was used for parsing the raw data and first quality checks. The array quality was assessed using the *arrayQualityMetrics* package version 3.50.0 (Kauffmann et al. 2009). For normalization of expression intensities neqc normalization (Shi et al. 2010) was applied using *beadarray*'s *normaliseIllumina* function (Dunning et al. 2007). Probes with quality scores assigned as "bad", "no match" or "not available" were removed. Differential expression analysis was evaluated with the *limma* package version 3.50.3 (Ritchie et al. 2015). First, a linear model with the experimental conditions was fitted for each gene of a dataset. "Donor" was included as a blocking factor to account for potential batch effects. Afterwards, the eBayes function of the empirical Bayes method was applied. Multiple testing correction was applied using the Benjamini and Hochberg false discovery rate method implemented in the topfit function from *limma*. The ranking of the genes was calculated as logFC × -log10(*P*-value). The pre-ranked gene lists were exported for each study separately containing the gene symbols and the calculated gene ranks.

8.2.3 RNA-seq data (SRP060567/GSE70667, SRP212008)

Gene count, metadata, and gene annotations for both studies were downloaded from recount3 (Wilks et al. 2021) and locally stored in a directory tree imitation the recount3 directory structure (see below). The *recount3* package version 1.4.0 (Collado-Torres 2023) was used to import the gene count data as RangedSummarizedExperiment class data and for initial data processing. The *DESeq2* package version 1.34.0 (Love et al. 2014) was then used for normalization and differential expression analysis. The *RNAseqQC* package version 0.1.4 (Ziebell 2022) was applied for quality control. After normalization, not expressed genes (i.e., genes with 0 counts) in all samples were removed and genes with a minimum count of ≥ 10 in at least 3 samples were retained. Lfcshrinkage was applied with the "apeglm" algorithm (Zhu et al. 2019) as implemented with the *lfcShrink* function of the *DESeq2* package (Love et al. 2014). Protein-coding genes were retained. ENSEMBL gene ids were converted to gene symbols. Gene symbols, that were not unambiguously assigned (probe id, ENSEMBL id) the entry with the highest log2FoldChange was retained. The ranking of the genes was then done according to the shrinked log2FoldChange. The pre-ranked gene lists were exported for each study separately containing the gene symbols and the calculated gene ranks.

```
recode3
|____mouse
| |____annotations
| | |____gene_sums
| | | |____mouse.gene_sums.M023.gtf.gz
| | |____exon_sums
| |   |____mouse.exon_sums.M023.gtf.gz
| |____data_sources
|   |____sra
|     |____gene_sums
|     | |____67
|     | | |____SRP060567
|     | |   |____sra.gene_sums.SRP060567.M023.gz
|     | |____08
|     |   |____SRP212008
|     |     |____sra.gene_sums.SRP212008.M023.gz
|     |____junctions
|     | |____67
|     | | |____SRP060567
|     | |   |____sra.junctions.SRP060567.ALL.ID.gz
|     | |   |____sra.junctions.SRP060567.ALL.RR.gz
|     | |   |____sra.junctions.SRP060567.ALL.MM.gz
|     | |____08
|     |   |____SRP212008
|     |     |____sra.junctions.SRP212008.ALL.RR.gz
|     |     |____sra.junctions.SRP212008.ALL.MM.gz
|     |     |____sra.junctions.SRP212008.ALL.ID.gz
|     |____exon_sums
|     | |____67
|     | | |____SRP060567
|     | |   |____sra.exon_sums.SRP060567.M023.gz
|     | |____08
|     |   |____SRP212008
|     |     |____sra.exon_sums.SRP212008.M023.gz
|     |____metadata
|       |____67
|       | |____SRP060567
|       |   |____sra.recount_seq_qc.SRP060567.MD.gz
|       |   |____sra.recount_qc.SRP060567.MD.gz
|       |   |____sra.recount_pred.SRP060567.MD.gz
|       |   |____sra.sra.SRP060567.MD.gz
|       |   |____sra.recount_project.SRP060567.MD.gz
|       |____08
|         |____SRP212008
|           |____sra.sra.SRP212008.MD.gz
|           |____sra.recount_project.SRP212008.MD.gz
|           |____sra.recount_seq_qc.SRP212008.MD.gz
|           |____sra.recount_qc.SRP212008.MD.gz
|           |____sra.recount_pred.SRP212008.MD.gz
|____recount3_selection_SRP060567.csv
|____recount3_SRP212008.R
```

8.3 References

Cheng X, Yan J, Liu Y, Wang J, Taubert S (2021). eVITTA: a web-based visualization and inference toolbox for transcriptome analysis. Nucleic Acids Res; 49(W1):W207-W215.

Collado-Torres L (2023). Explore and download data from the recount3 project, R package version 1.4.0; doi:10.18129/B9.bioc.recount3;URL: https://www.bioconductor.org/packages/recount3.

Diaz MF, Vaidya AB, Evans SM, Lee HJ, Aertker BM, Alexander AJ, Price KM, Ozuna JA, Liao GP, Aroom KR, Xue H, Gu L, Omichi R, Bedi S, Olson SD, Cox CS, Jr., Wenzel PL (2017). Biomechanical Forces Promote Immune Regulatory Function of Bone Marrow Mesenchymal Stromal Cells. Stem Cells; 35(5):1259-1272.

Du J, Yuan Z, Ma Z, Song J, Xie X, Chen Y (2014). KEGG-PATH: Kyoto encyclopedia of genes and genomes-based pathway analysis using a path analysis model. Mol Biosyst; 10(9):2441-7.

Dunning MJ, Smith ML, Ritchie ME, Tavare S (2007). beadarray: R classes and methods for Illumina bead-based data. Bioinformatics; 23(16):2183-4.

Gautier L, Cope L, Bolstad BM, Irizarry RA (2004). affy--analysis of Affymetrix GeneChip data at the probe level. Bioinformatics; 20(3):307-15.

Glossop JR, Cartmell SH (2009). Effect of fluid flow-induced shear stress on human mesenchymal stem cells: differential gene expression of IL1B and MAP3K8 in MAPK signaling. Gene Expression Patterns; 9(5):381-8.

Govey PM, Jacobs JM, Tilton SC, Loiselle AE, Zhang Y, Freeman WM, Waters KM, Karin NJ, Donahue HJ (2014). Integrative transcriptomic and proteomic analysis of osteocytic cells exposed to fluid flow reveals novel mechano-sensitive signaling pathways. J Biomech; 47(8):1838-45.

Govey PM, Kawasawa YI, Donahue HJ (2015). Mapping the osteocytic cell response to fluid flow using RNA-Seq. J Biomech; 48(16):4327-32.

Huber W, Carey VJ, Gentleman R, Anders S, Carlson M, Carvalho BS, Bravo HC, Davis S, Gatto L, Girke T, Gottardo R, Hahne F, Hansen KD, Irizarry RA, Lawrence M, Love MI, MacDonald J, Obenchain V, Oles AK, Pages H, Reyes A, Shannon P, Smyth GK, Tenenbaum D, Waldron L, Morgan M (2015). Orchestrating high-throughput genomic analysis with Bioconductor. Nat Methods; 12(2):115-21.

Kauffmann A, Gentleman R, Huber W (2009). arrayQualityMetrics--a bioconductor package for quality assessment of microarray data. Bioinformatics; 25(3):415-6.

Klaus B, Reisenauer S (2016). An end to end workflow for differential gene expression using Affymetrix microarrays. F1000Res; 5:1384.

Li X, Han L, Nookaew I, Mannen E, Silva MJ, Almeida M, Xiong J (2019). Stimulation of Piezo1 by mechanical signals promotes bone anabolism. Elife; 8:e49631.

Love MI, Huber W, Anders S (2014). Moderated estimation of fold change and dispersion for RNA-seq data with DESeq2. Genome Biology; 15(12):550.

Martens M, Ammar A, Riutta A, Waagmeester A, Slenter DN, Hanspers K, R AM, Digles D, Lopes EN, Ehrhart F, Dupuis LJ, Winckers LA, Coort SL, Willighagen EL, Evelo CT, Pico AR, Kutmon M (2021). WikiPathways: connecting communities. Nucleic Acids Res; 49(D1):D613-D621.

Pico AR, Kelder T, van Iersel MP, Hanspers K, Conklin BR, Evelo C (2008). WikiPathways: pathway editing for the people. PLoS Biol; 6(7):e184.

R Core Team (2021). R: A Language and Environment for Statistical Computing, Vienna, Austria: R Foundation for Statistical Computing;URL: https://www.R-project.org/.

Ritchie ME, Phipson B, Wu D, Hu Y, Law CW, Shi W, Smyth GK (2015). limma powers differential expression analyses for RNA-sequencing and microarray studies. Nucleic Acids Res; 43(7):e47.

RStudio Team (2022). RStudio: Integrated Development Environment for R, 2021.9.2.382; Boston, MA: RStudio, PBC;URL: https://www.rstudio.com/.

Shi W, Oshlack A, Smyth GK (2010). Optimizing the noise versus bias trade-off for Illumina whole genome expression BeadChips. Nucleic Acids Res; 38(22):e204.

Thomas PD (2017). The Gene Ontology and the Meaning of Biological Function. Methods Mol Biol; 1446:15-24.

Wilks C, Zheng SC, Chen FY, Charles R, Solomon B, Ling JP, Imada EL, Zhang D, Joseph L, Leek JT, Jaffe AE, Nellore A, Collado-Torres L, Hansen KD, Langmead B (2021). recount3: summaries and queries for large-scale RNA-seq expression and splicing. Genome Biology; 22(1):323.

Zhu A, Ibrahim JG, Love MI (2019). Heavy-tailed prior distributions for sequence count data: removing the noise and preserving large differences. Bioinformatics; 35(12):2084-2092.

Ziebell F (2022). RNAseqQC: Quality Control for RNA-Seq Data, R package version 0.1.4;URL: https://CRAN.R-project.org/package=RNAseqQC.

8.4 Human bone marrow mesenchymal stem cells (MSCs)

8.4.1 Summary

	**E-MEXP-1923**	**GSE82269**
**Sample information**		
Reference	Glossop and Cartmell (2009)	Diaz et al. (2017)
Download[1]	ArrayExpress; E-MEXP-1923	Gene Expression Omnibus (GEO); GSE82269
Platform	Affymetrix GeneChip Human Genome U133 Plus 2.0 [HG-U133_Plus_2]	Illumina HumanHT-12 V4.0 Expression BeadChip< (GPL10558; HumanHT-12_V4_0_R2_15002873_B.txt)
Type	Microarray	Microarray/BeadArray
Cell type	Human bone marrow MSCs from 1 female donor (23 years old; commercial source)	Human bone marrow MSCs (3 donors: 1 female, 20 years old; 2 male, 22 and 25 years old; commercial source)
Samples	total N = 11 CEL-files; included were 2 arrays for FSS (see below) and 2 arrays representing the corresponding controls	total N = 6 array IDAT-files; FSS (3 arrays), control (3 arrays; from each donor one for FSS and one as control)
FSS application of selected samples	Streamer Fluid Shear Bioreactor (FlexCell Int.); 72h adhesion; FSS with 1dyn/cm^2 for 1h and 2h post-FSS (2 arrays), control without FSS but otherwise treated identically (2 arrays)	ibidi VI$^{0.4}$ channel slides; 18h adhesion; FSS with 15dyn/cm^2 for 6h (FSS) or without FSS but otherwise treated identically (control)
**easyGSEA**[2]	Pre-ranked gene set enrichment analysis using default settings; DB: KEGG, WikiPathways, GO/Biological Process; gene set size filters: min=15, max=500; results in 3130 of 13461 gene sets	
File:	hsMSC_E-MEXP-1923_AE_eset_SYMBOLS_sorted.rnk	hsMSC_GSE82269_geo_w_beadarray_SYMBOLS-bead_sorted.rnk
Number of genes considered:	20446 / 20446	19220 / 19220
Summary report:	10 (down) 319 (up) gene sets are sig. enriched at P_{adj} < 0.25<br>0 (down) 84 (up) gene sets are sig. enriched at P_{adj} < 0.05	31 (down) 198 (up) gene sets are sig. enriched at P_{adj} < 0.25
**easyVizR**[3]	Default filter settings: *P*<0.05; FDR < 1.1; |ES| > -0.1 => Filtered only according to *P* value!	
Filtered list:	456 out of 2687 total	347 out of 2687 total
Intersection:	**Overlap of 162 (25%) gene sets in both studies with *P* < 0.05**	

[1] https://www.ebi.ac.uk/biostudies/arrayexpress/studies/E-MEXP-1923; https://www.ncbi.nlm.nih.gov/geo/query/acc.cgi?acc=GSE82269
[2] URL: https://tau.cmmt.ubc.ca/eVITTA/easyGSEA/ (2023-04-25)
[3] URL: https://tau.cmmt.ubc.ca/eVITTA/easyVizR/ (2023-04-25)

8.4.2 GSE82269 and E-MEXP-1923: easyVizR comparison of the pre-ranked easyGSEA results

Sorted according to descending ES of E-MEXP-1923 GSEA results; at least one of the GSEA results showed FDR ≤ 0.1 for a given gene set (green).

ES = enrichment score; FDR = false discovery rate; NES = normalized enrichment score; Size = size statistics reported by fGSEA

Gene set	Gene set Id	E-MEXP-1923						GSE82269					
		ES	*P*-value	FDR	NES	Size	Leading edge	ES	*P*-value	FDR	NES	Size	Leading edge
WP OVERVIEW OF NANOPARTICLE EFFECTS	WP3287	0.9820	0.0020	0.0375	1.6081	19	PTGS2; IL6; CXCL8; PIK3CD; TNF	0.9603	0.0015	0.0642	1.6238	18	PTGS2; HMOX1; CXCL8; IL6; PIK3CD
WP IL1 AND MEGAKARYOCYTES IN OBESITY	WP2865	0.9765	0.0020	0.0375	1.6301	22	HBEGF; ICAM1; CCL2; IL1B; NFKB1; NLRP3	0.8878	0.0030	0.0793	1.5569	23	NFKB1; HBEGF; CCL2; TIMP2
WP CELLS AND MOLECULES INVOLVED IN LOCAL ACUTE INFLAMMATORY RESPONSE	WP4493	0.9718	0.0040	0.0532	1.5793	17	ICAM1; IL6; CXCL8; IL1A; TNF; VCAM1	0.8684	0.0250	0.1782	1.4532	16	CXCL8; IL6; ITGA4; IL1A
WP CANCER IMMUNOTHERAPY BY PD-1 BLOCKADE	WP4585	0.9628	0.0040	0.0532	1.6023	23	CD274; NFKB1; NFATC2; NFATC1; JUN	0.8428	0.0210	0.1782	1.4667	22	NFKB1; NFATC1; NFATC3; STAT3
WP RESISTIN AS A REGULATOR OF INFLAMMATION	WP4481	0.9619	0.0019	0.0375	1.6612	32	IL6; CXCL8; NFKB1; NFKBIA; PIK3CD; TNF; RETN; RELA	0.7895	0.0364	0.2125	1.4401	30	NFKB1; CXCL8; IL6; PIK3CD
WP LTF DANGER SIGNAL RESPONSE PATHWAY	WP4478	0.9612	0.0059	0.0614	1.5740	19	IL6; CXCL8; IL1B; NFKB1; IL1A; TNF; TRAF6	0.9427	0.0015	0.0642	1.5939	18	NFKB1; CXCL8; IL6; TRAF6
WP HYPERTROPHY MODEL	WP516	0.9596	0.0060	0.0614	1.5724	18	HBEGF; NR4A3; IL1A; ATF3; VEGFA; ANKRD1; DUSP14	0.8894	0.0123	0.1400	1.5038	18	HBEGF; IFRD1; EIF4E; ATF3; CCN1; DUSP14
WP PHOTODYNAMIC THERAPY-INDUCED NF-KB SURVIVAL SIGNALING	WP3617	0.9590	0.0019	0.0375	1.6673	33	PTGS2; ICAM1; IL6; CXCL2; CXCL8; IL1B; NFKB1; IL1A; BCL2A1; TNF; REL; BIRC3; VEGFA; RELB; MMP3; MMP1; NFKB2; TRAF6	0.9186	0.0029	0.0793	1.6936	32	PTGS2; NFKB1; CXCL8; IL6; TRAF6; BCL2A1; CCND1
WP EICOSANOID SYNTHESIS	WP167	0.9505	0.0060	0.0614	1.5817	23	PTGS2; PTGS1	0.8235	0.0315	0.2082	1.4333	22	PTGS2; PLA2G4A; PNPLA8; ALOX5
KEGG IL-17 SIGNALING PATHWAY	HSA04657	0.9495	0.0016	0.0134	1.7876	93	PTGS2; FOSB; CXCL3; IL6; CCL20; CCL2; CXCL2; CXCL8; CXCL1; IL1B; TNFAIP3; NFKB1; CCL7; NFKBIA; TNF; CXCL10; MMP3; JUN; MMP1; TRAF3IP2; FOSL1; TAB3; TRAF6; TAB2; TRAF3	0.7834	0.0027	0.0763	1.6347	86	PTGS2; NFKB1; CCL20; FOSB; CXCL8; IL6; CEBPB; TRAF6; CASP3; MAPK6; TRAF3
WP T-CELL RECEPTOR AND CO-STIMULATORY SIGNALING	WP2583	0.9494	0.0039	0.0532	1.6149	28	NFKB1; NFKBIA; NFATC2; DYRK2; RASGRP1	0.7965	0.0363	0.2125	1.4510	29	NFKB1; DYRK2; DYRK1A; RASA1; PDK1; CSNK1A1
KEGG C-TYPE LECTIN RECEPTOR SIGNALING PATHWAY	HSA04625	0.9416	0.0016	0.0134	1.7958	99	PTGS2; EGR2; EGR3; IL6; IL1B; PLK3; NFKB1; NFKBIA; NFATC2; IRF1; PIK3CD; TNF; NFATC1; NLRP3; RELB; MALT1; PPP3CC; JUN; BCL10; NFKB2	0.7238	0.0026	0.0763	1.5454	98	PTGS2; EGR2; NFKB1; IL6; NFATC1; PIK3CD; MRAS; PLK3; EGR3; NRAS
KEGG TNF SIGNALING PATHWAY	HSA04668	0.9406	0.0015	0.0134	1.8129	113	PTGS2; LIF; CXCL3; ICAM1; IL6; CCL20; MAP3K8; CCL2; CXCL2; JUNB; CXCL1; IL1B; TNFAIP3; NFKB1; NFKBIA; IRF1; PIK3CD; TNF; JAG1; MAP2K3; BIRC3; CXCL10; TRAF1; EDN1; MMP3; JUN	0.7847	0.0013	0.0655	1.6802	106	PTGS2; NFKB1; CCL20; LIF; IL6; EDN1; JUNB; VEGFC; MAP2K3; PIK3CD; CEBPB; CREB3L2; CASP3; JAG1; PGAM5; FAS; TRAF3
WP GLUCOCORTICOID RECEPTOR PATHWAY	WP2880	0.9382	0.0016	0.0375	1.7452	69	PTGS2; IL11; CCL20; ANGPTL4; CCL2; SLC19A2; TNFAIP3; PPP1R14C; BHLHE40; BIRC3; RGS2; PLK2; ANKRD1; GADD45B; ENC1; CPEB4; ACKR3; SERTAD2; JUN; AMIGO2; ARL5B; NFKB2; LRRC8A	0.7420	0.0177	0.1747	1.4908	65	PTGS2; CCL20; GADD45B; LRRC8A; PPP1R14C; NAV3; ARL5B; ENC1; ANGPTL4; SLC26A2; RGS2; TSC22D3; ABHD2
WP EBV LMP1 SIGNALING	WP262	0.9379	0.0159	0.0946	1.5608	23	CCL20; CXCL8; NFKB1; NFKBIA; TNF; TRAF1; NFKB2; TRAF6	0.8758	0.0060	0.1073	1.5243	22	NFKB1; CCL20; CXCL8; TRAF6; MAP3K3; MAP3K7; HSP90AA1
WP VITAMIN B12 METABOLISM	WP1533	0.9368	0.0036	0.0532	1.6511	49	ICAM1; IL6; CCL2; IL1B; NFKB1; TNF; SERPINE1; NFKB2	0.8543	0.0028	0.0793	1.6482	45	NFKB1; IL6; MTRR; SERPINE1; CCL2
WP FIBRIN COMPLEMENT RECEPTOR 3 SIGNALING PATHWAY	WP4136	0.9363	0.0037	0.0532	1.6358	40	CXCL3; IL6; CCL2; IRAK2; NFKB1; TNF; CXCL10; RASSF5; TICAM1; TRAF6	0.8213	0.0128	0.1400	1.5281	34	NFKB1; IL6; TRAF6; FGA; CCL2; SRC; IKBKB
WP TNF RELATED WEAK INDUCER OF APOPTOSIS (TWEAK) SIGNALING PATHWAY	WP2036	0.9309	0.0055	0.0614	1.6156	42	IL6; CCL2; NFKB1; NFKBIA; TNF; BIRC3; TNFRSF12A; TRAF1; RELB; JUN; NFKB2; TRAF3	0.8105	0.0125	0.1400	1.5499	40	NFKB1; IL6; TNFRSF12A; CASP3; TRAF3; MAP3K7; NFKBIB; CCL2; IKBKB; CCL5; MAPK8
WP SELENIUM MICRONUTRIENT NETWORK	WP15	0.9305	0.0016	0.0375	1.7317	84	PTGS2; ICAM1; IL6; CCL2; IL1B; NFKB1; TNF; SERPINE1; NFKB2	0.7942	0.0026	0.0793	1.6422	80	PTGS2; NFKB1; TXNRD1; IL6; SELENOI; SERPINE1; SELENOK; PNPO; SELENOS; SELENOT; RFK; FLAD1; ALOX5; FGA; CCL2
KEGG NF-KAPPA B SIGNALING PATHWAY	HSA04064	0.9298	0.0016	0.0134	1.7733	99	PTGS2; CXCL3; ICAM1; CXCL2; CXCL8; CXCL1; IL1B; TNFAIP3; NFKB1; NFKBIA; BCL2A1; TNF; BIRC3; GADD45A; TRAF1; RELB; MALT1; GADD45B; PLAU; TICAM1; BCL10; NFKB2; TAB3; TRAF6; TAB2; TRAF3; CFLAR; CARD10; EDA; VCAM1; TRAF2	0.7488	0.0052	0.1090	1.5753	89	PTGS2; NFKB1; CXCL8; GADD45B; GADD45G; TRAF6; BCL2A1; PLAU; TRAF3; GADD45A; MAP3K7
WP OLIGODENDROCYTE SPECIFICATION AND DIFFERENTIATION. LEADING TO MYELIN COMPONENTS FOR CNS	WP4304	0.9254	0.0138	0.0903	1.5566	27	LIF; CXCL2; CXCL1; IL1B; BMP2; TNF; FGF2; GLI2	0.9357	0.0015	0.0642	1.7068	30	LIF; BMP2; FGF2; NKX2-6
KEGG OVARIAN STEROIDOGENESIS	HSA04913	0.9148	0.0018	0.0134	1.6329	47	PTGS2; BMP6	0.7647	0.0228	0.1968	1.4743	46	PTGS2; PLA2G4A; CYP1B1; ALOX5; LHB; PRKACB
WP MELATONIN METABOLISM AND EFFECTS	WP3298	0.9123	0.0112	0.0816	1.5851	37	FOXO1; NFKB1; PER2; EDN1; MIR3142HG; CRY1; TRAF6	0.8243	0.0112	0.1400	1.5630	38	NFKB1; EDN1; CRY1; FOXO1; TRAF6; CYP1B1; SIRT1; ECE1; ARNTL
WP FOLATE METABOLISM	WP176	0.9123	0.0017	0.0375	1.6758	66	ICAM1; IL6; CCL2; IL1B; NFKB1; TNF; SERPINE1; NFKB2	0.6968	0.0355	0.2125	1.3896	62	NFKB1; IL6; MTRR; SERPINE1; RFK; MTHFD2; FLAD1; FGA; CCL2

Gene set	Gene set Id	E-MEXP-1923						GSE82269					
		ES	*P*-value	FDR	NES	Size	Leading edge	ES	*P*-value	FDR	NES	Size	Leading edge
WP SPINAL CORD INJURY	WP2431	0.9119	0.0015	0.0375	1.7443	115	PTGS2; ICAM1; IL6; CCL2; CXCL2; CXCL8; CXCL1; IL1B; EGR1; IL1A; NR4A1; RHOB; TNF; GADD45A; CXCL10; FOXO3; NOX4; GDNF; CHST11	0.7374	0.0037	0.0884	1.5819	114	PTGS2; NOX4; CXCL8; IL6; RHOB; EGR1; CCND1; FOXO3; CASP3; MYC; GADD45A; NR4A1; EFNB2
WP IL-18 SIGNALING PATHWAY	WP4754	0.9114	0.0013	0.0375	1.8706	263	PTGS2; CXCL3; ICAM1; IL6; CCL20; CCL2; ZC3H12A; CXCL2; IER3; CXCL8; IL1B; TNFAIP3; BMP2; NFKB1; NFKBIA; TNFAIP2; CD83; NR4A1; NFKBIE; IRF1; TNIP3; TNF; MEF2A; SDC4; NFKBIZ; REL; ATF3; BIRC3; TRAF1; VEGFA; RGS16; MMP3; JUN; CLDN1; HSPB8; MMP1; CCL3; NFKB2; SPP1	0.6373	0.0045	0.0921	1.4554	252	PTGS2; HMOX1; NFKB1; CCL20; CXCL8; BMP2; IL6; HSPB8; SLC4A7; CEBPB; TRAF6; ARFGAP1; ATF3; CASP3; IER3; BPGM; PRCC; TNFRSF11B; TMEM165; RUNX2; FAS; RXRB; GPAT4; ABCF1; NR4A1; LMNB2; MAP3K7; RAE1; TOMM40; CLDN1; CD83; FBXW7; IL18BP; NCF1; CCL2
KEGG ARACHIDONIC ACID METABOLISM	HSA00590	0.9103	0.0017	0.0134	1.6488	57	PTGS2; CYP2C9	0.7566	0.0169	0.1623	1.4743	51	PTGS2; PLA2G4A; ALOX5
WP BLADDER CANCER	WP2828	0.9063	0.0129	0.0870	1.5833	40	HBEGF; CXCL8; VEGFA; MMP1; EGFR; CDH1; DAPK3; SRC	0.7544	0.0338	0.2125	1.4368	39	HBEGF; CXCL8; CDKN1A; CCND1; MYC; NRAS; TYMP
WP CORTICOTROPIN-RELEASING HORMONE SIGNALING PATHWAY	WP2355	0.8986	0.0016	0.0375	1.6780	90	FOSB; CXCL8; JUNB; NFKB1; NR4A2; NFKBIA; NR4A1; ERN1; FOSL1	0.6773	0.0337	0.2125	1.4007	84	NFKB1; FOSB; CXCL8; JUNB; CASP3; GNAQ; TFAP2A; NR4A1; ECE1; HSP90AA1; FOSL1; PTK2; NR4A2; BCL2
WP CYTOSOLIC DNA-SENSING PATHWAY	WP4655	0.8940	0.0033	0.0532	1.6473	67	IL6; IL1B; NFKB1; NFKBIA; CXCL10; CGAS	0.7320	0.0231	0.1782	1.4520	59	NFKB1; IL6; IFNA8; POLR1C; POLR3D; NFKBIB; POLR3E
KEGG CYTOSOLIC DNA-SENSING PATHWAY	HSA04623	0.8931	0.0017	0.0134	1.6395	67	IL6; IL1B; NFKB1; NFKBIA; CXCL10; NLRP3; CGAS	0.7577	0.0083	0.1384	1.5061	59	NFKB1; IL6; CASP3; IFNA8; POLR1C; POLR3D; NFKBIB; POLR3E
WP STRUCTURAL PATHWAY OF INTERLEUKIN 1 (IL-1)	WP2637	0.8879	0.0126	0.0863	1.5649	49	MAP3K8; IRAK2; NFKB1; NFKBIA; IL1A; MAP2K3; IL1RAP; TAB3; TRAF6; TAB2	0.7686	0.0194	0.1782	1.4845	47	NFKB1; MAP2K3; TRAF6; EIF4E; MYC; MAP3K3; MAP3K7; NFKBIB; TOLLIP; IL1A; IKBKB; MAPK8; SAFB
KEGG REGULATION OF LIPOLYSIS IN ADIPOCYTES	HSA04923	0.8834	0.0121	0.0620	1.5939	56	PTGS2; PIK3CD; IRS2	0.7334	0.0266	0.1968	1.4530	57	PTGS2; PIK3CD; NPR1; IRS2; PRKACB; PTGER3; ADCY2; ABHD5
WP TOLL-LIKE RECEPTOR SIGNALING PATHWAY	WP75	0.8822	0.0016	0.0375	1.6725	98	IL6; MAP3K8; CXCL8; IL1B; NFKB1; NFKBIA; PIK3CD; TNF; MAP2K3; CXCL10; JUN; TICAM1; CCL3; SPP1; TAB3; TRAF6; TAB2; TRAF3	0.7073	0.0128	0.1400	1.4832	93	NFKB1; CXCL8; IL6; MAP2K3; PIK3CD; TRAF6; IFNA8; TRAF3; IFNAR1; MAP3K7; NFKBIB; TOLLIP
KEGG TOLL-LIKE RECEPTOR SIGNALING PATHWAY	HSA04620	0.8807	0.0016	0.0134	1.6643	95	IL6; MAP3K8; CXCL8; IL1B; NFKB1; NFKBIA; PIK3CD; TNF; MAP2K3; CXCL10; JUN; TICAM1; CCL3; SPP1; TRAF6; TAB2; TRAF3	0.7052	0.0117	0.1547	1.4895	92	NFKB1; CXCL8; IL6; MAP2K3; PIK3CD; TRAF6; IFNA8; TRAF3; IFNAR1; MAP3K7; TOLLIP
KEGG CYTOKINE-CYTOKINE RECEPTOR INTERACTION	HSA04060	0.8769	0.0013	0.0134	1.8113	274	LIF; IL11; CXCL3; IL6; CCL20; TSLP; CCL2; CXCL2; CXCL8; CXCL1; IL1B; BMP2; CLCF1; CCL7; IL1A; TNF; TNFSF9; CXCL10; TNFRSF12A; INHBA; ACKR3; RELT; CCL3; BMPR2; NGF; BMP6; IL1RAP; AMH; IL5RA; IL7R; TNFRSF10B; IFNGR2; TGFBR1; ACVR2A	0.5892	0.0139	0.1597	1.3513	268	CLCF1; CCL20; LIF; CXCL8; BMP2; IL6; TNFRSF12A; NGF; BMPR2; IFNA8; TNFRSF11B; FAS; XCR1; INHBA; IFNAR1; IL5RA; BMP3; CXCL14; PPBP; GDF11; CCL2; TNFRSF10B; IL15RA; GDF9; CXCR5; CSF1R; GDF15; IL5; IL1A; CXCR3; IL24; ACVR2A; CCL5; IFNE; ACKR4; IL17C
WP NUCLEAR RECEPTORS META-PATHWAY	WP2882	0.8678	0.0013	0.0375	1.7976	300	PTGS2; HBEGF; IL11; CCL20; ANGPTL4; CCL2; SLC19A2; JUNB; IL1B; FOXO1; TNFAIP3; SERPINB2; PPP1R14C; EGR1; MAFF; BHLHE40; TNF; BIRC3; RGS2; SLC7A5; EPHA2; PLK2; ACOX1; ANKRD1; GADD45B; SLC2A1; ENC1; CPEB4; SLC39A14; ACKR3; SERTAD2; JUN; PPARD; NRG1; AMIGO2; VDR; ARL5B; NFKB2; LRRC8A; EGFR; THBD; IRS2; SLC2A6; MAFG; SERPINA1	0.6842	0.0011	0.0642	1.5751	294	PTGS2; HMOX1; GCLM; SERPINB2; HSPA1A; MAFG; TXNRD1; CCL20; HBEGF; GCLC; JUNB; SRXN1; GADD45B; SLC7A11; FOXO1; LRRC8A; PPP1R14C; NAV3; GPAM; EGR1; ACOX1; ABCC5; ARL5B; ENC1; SLC7A5; MAFF; ANGPTL4; VDR; CCND1; SLC26A2; CYP1B1; DNAJB1; SLC39A14; MYC; PPARD; RGS2; TSC22D3; GCC1; IGFBP1; ABHD2; NQO1; SLC6A17
WP BRAIN-DERIVED NEUROTROPHIC FACTOR (BDNF) SIGNALING PATHWAY	WP2380	0.8557	0.0014	0.0375	1.6638	144	EGR2; BMP2; EGR1; NFKB1; NFKBIA; MEF2A; FOXO3; STAT5A; CDKL5; SIRPA; JUN; NGF; SPP1; TRAF6; IRS2; FRS2; MAP3K2; SRC; NCF2; MAPK8	0.6796	0.0133	0.1424	1.4855	137	EGR2; NFKB1; BMP2; EGR1; SIRPA; TRAF6; RAP1A; NGF; EIF4E; PPP2CA; FOXO3; CASP3; EIF2S2; GRIA3; PDPK1; MAPT; CDK5R1; DOCK3; IRS2; DOK5; NCF1; STAT3; SHC1; SQSTM1; SRC; CSNK2A1; MEF2A; FRS2; IKBKB
KEGG FLUID SHEAR STRESS AND ATHEROSCLEROSIS	HSA05418	0.8474	0.0015	0.0134	1.6367	135	ICAM1; CCL2; IL1B; NFKB1; IL1A; PDGFA; PIK3CD; TNF; MEF2A; SDC4; VEGFA; EDN1; JUN; DUSP1; BMPR2; THBD; KDR; ACVR2A; VCAM1; SRC; NCF2; MAPK8	0.6340	0.0244	0.1968	1.3840	132	HMOX1; NFKB1; EDN1; PIK3CD; BMPR2; NQO1; MAP3K7; HSP90AA1; NCF1; CCL2; PTK2; KLF2; BCL2; SDC1; GSTM2; SQSTM1; SRC; SELE; IL1A; MEF2A; GSTO2; IKBKB; ACVR2A; ACTB; MAPK8; AKT3
WP VEGFA-VEGFR2 SIGNALING PATHWAY	WP3888	0.8432	0.0012	0.0375	1.7895	425	PTGS2; HBEGF; EGR3; ICAM1; CCL2; CXCL8; NR4A3; FOXO1; DUSP5; BMP2; EGR1; HLX; NFKB1; NR4A2; NFKBIA; NFATC2; NR4A1; ETS1; F3; PLAUR; JAG1; MAP2K3; NFATC1; FOXO3; SHB; SRF; ERN1; VEGFA; CSRP2; NOX4; NRP2; EPHA2; RCAN1; CCN2; ACKR3; FMNL3; PLAU; JUN; SHROOM2; PNP; C15ORF39; TRAF3IP2; ABL1; TEAD4; IER5; SLC7A1; NRARP; KDR; FRS2; CSRP1; ARPC5L; KANK1	0.6419	0.0011	0.0642	1.5011	417	PTGS2; HLX; DNAJB9; NFKB1; HSPA1A; DUSP5; HBEGF; NOX4; CXCL8; BMP2; DNAJA1; TMEM170A; SDF2L1; GRB10; NFATC1; RBM39; PNP; FOXO1; LUC7L; MAP2K3; EGR1; DNAJB4; HERPUD1; SLC25A25; RAP1A; CHAC1; PLA2G4A; SYNJ1; CSRP2; ELOA; EIF4E; TEAD4; PPP2CA; GIGYF2; PRRC2C; STAM; CCN1; CCND1; FOXO3;

Gene set	Gene set Id	E-MEXP-1923						GSE82269					
		ES	*P*-value	FDR	NES	Size	Leading edge	ES	*P*-value	FDR	NES	Size	Leading edge
													PTPN1; CBL; JAG1; STIP1; ARPC5L; HDAC5; CRK; EGR3; SPIRE1; PLAUR; PLAU; PDPK1; CCN2; SSR3; RHOJ; FAS
KEGG CELLULAR SENESCENCE	HSA04218	0.8380	0.0014	0.0134	1.6499	154	IL6; CXCL8; FOXO1; NFKB1; NFATC2; IL1A; ETS1; PIK3CD; MAP2K3; NFATC1; GADD45A; FOXO3; SERPINE1; RASSF5; GADD45B; PPP3CC; CDKN2B; CDK6; TRAF3IP2; HIPK3	0.6081	0.0402	0.2432	1.3398	150	CDKN2B; NFKB1; CXCL8; IL6; GADD45B; NFATC1; FOXO1; MAP2K3; GADD45G; PIK3CD; MRAS; CDKN1A; HIPK2; SERPINE1; CCND1; FOXO3; TSC1; MYBL2; MYC; NRAS; CDK6; E2F3; HIPK3; NFATC3; GADD45A
KEGG LIPID AND ATHEROSCLEROSIS	HSA05417	0.8368	0.0014	0.0134	1.6706	204	CXCL3; ICAM1; IL6; CCL2; CXCL2; CXCL8; CXCL1; IL1B; NFKB1; NFKBIA; NFATC2; PIK3CD; TNF; MAP2K3; NFATC1; ERN1; NLRP3; MMP3; PPP3CC; JUN; TICAM1; MMP1; CCL3; TRAF6; TAB2; TRAF3; TNFRSF10B; POU2F2; SOD2; VCAM1; TRAF2	0.6619	0.0024	0.0763	1.4880	197	NFKB1; HSPA1A; HSPA1B; CXCL8; IL6; HSPA5; NFATC1; MAP2K3; PIK3CD; HSPA4; TRAF6; RAP1A; XBP1; CASP3; IFNA8; HSPA8; PDPK1; NRAS; FAS; TRAF3; APAF1; RXRB; NFATC3; MAP3K7; EIF2AK3; HSP90AA1; LYN; POU2F3; NCF1; CCL2; HSPD1; TNFRSF10B; PTK2; BCL2; STAT3
WP GASTRIN SIGNALING PATHWAY	WP4659	0.8357	0.0076	0.0703	1.5983	112	PTGS2; CXCL8; FOXO1; SERPINB2; BMP2; EGR1; NFKB1; NFKBIA; RHOB; JAG1; BIRC3; FOXO3; VEGFA; SERPINE1; JUN; CLDN1	0.7246	0.0062	0.1073	1.5567	111	PTGS2; SERPINB2; NFKB1; CXCL8; BMP2; RHOB; FOXO1; EGR1; CDKN1A; SERPINE1; CCND1; FOXO3; CASP3; LAMTOR3; JAG1; CRK; MYC; GNAQ; CHGA
WP B CELL RECEPTOR SIGNALING PATHWAY	WP23	0.8240	0.0124	0.0863	1.5558	96	FOXO1; NFKB1; NFKBIA; NFATC2; ETS1; REL; BCL6; MALT1; JUN; BCL10	0.6602	0.0420	0.2356	1.3841	92	NFKB1; FOXO1; MAX; CBL; CRK; MYC; PDPK1; PIP5K1A; E2F3; NFATC3; MAP3K7; GAB1; LYN; BTK; SHC1
KEGG SEROTONERGIC SYNAPSE	HSA04726	0.8134	0.0092	0.0491	1.5603	103	PTGS2; DUSP1	0.6605	0.0251	0.1968	1.4217	108	PTGS2; PLA2G4A; CASP3; NRAS; GNAQ; HTR1E; CYP4X1; GNG7; ALOX5; CACNA1B; PRKACB
WP NRF2 PATHWAY	WP2884	0.8133	0.0090	0.0703	1.5695	134	HBEGF; EGR1; MAFF; EPHA2; SLC2A1; SLC39A14; PPARD; NRG1; SLC2A6; MAFG; SERPINA1; ADH7	0.6990	0.0036	0.0884	1.5242	132	HMOX1; GCLM; HSPA1A; MAFG; TXNRD1; HBEGF; GCLC; SRXN1; SLC7A11; EGR1; ABCC5; MAFF; DNAJB1; SLC39A14; PPARD; NQO1; SLC6A17; UGT1A9; SLC5A6
WP MAPK SIGNALING PATHWAY	WP382	0.7606	0.0078	0.0703	1.5592	242	MAP3K8; DUSP6; IL1B; FGF1; NFKB1; FGF18; IL1A; NR4A1; PDGFA; TNF; FGF2; MAP2K3; NFATC1; GADD45A; SRF; DUSP2; RELB; PPP3CC; JUN; MAP3K4; DUSP1; NGF; NFKB2; DUSP7; TRAF6; RASGRP2; EGFR; TAB2; MAP3K2; TGFBR1; CACNG2; TRAF2; FLNC; FGF8; DUSP16; MAPK8; RAPGEF2	0.6111	0.0192	0.1782	1.3905	232	NFKB1; HSPA1A; MAP3K4; HSPA1B; FGF1; FGF2; NLK; NFATC1; PTPRR; MAP2K3; MRAS; ELK4; TRAF6; RAP1A; MAX; PLA2G4A; NGF; CASP3; RASA1; HSPA8; LAMTOR3; DUSP2; CRK; MYC; PPM1A; FLNC; PPM1B; MAPT; NRAS; FAS; RAPGEF2; FGF5; NFATC3; GADD45A; NR4A1; DUSP16; MAP3K7; SOS2; CACNA1B; DUSP3; TAOK2
KEGG MAPK SIGNALING PATHWAY	HSA04010	0.7443	0.0038	0.0241	1.5514	291	MAP3K8; DUSP6; IL1B; DUSP5; EREG; FGF1; NFKB1; FGF18; IL1A; NR4A1; PDGFA; AREG; TNF; FGF2; MAP2K3; NFATC1; GADD45A; SRF; DUSP2; VEGFA; EPHA2; RELB; GADD45B; PPP3CC; JUN; MAP3K4; DUSP1; NGF; EFNA1; IL1RAP; NFKB2; DUSP7; TRAF6; RASGRP2; EGFR; TAB2; KDR; MAP3K2; TGFBR1; CACNG2; TRAF2; FLNC; FGF8; DUSP16; MAPK8	0.6178	0.0023	0.0763	1.4197	274	NFKB1; HSPA1A; MAP3K4; DUSP5; HSPA1B; FGF1; FGF2; GADD45B; NLK; NFATC1; VEGFC; PTPRR; MAP2K3; GADD45G; MRAS; ELK4; TRAF6; RAP1A; MAX; PLA2G4A; NGF; CASP3; RASA1; HSPA8; LAMTOR3; DUSP2; CRK; MYC; PPM1A; FLNC; PPM1B; MAPT; NRAS; FAS; RAPGEF2; FGF5; NFATC3; GADD45A; NR4A1; DUSP16; MAP3K3; MAP3K7

Gene sets removed: HSA05030, HSA04936, HSA05133, HSA05134, HSA05135, HSA05140, HSA05146, HSA05161, HSA05162, HSA05163, HSA05164, HSA05165, HSA05167, HSA05203, HSA05204, HSA05206, HSA05222, WP1544, WP2446, WP3617, WP3624, WP3646, WP4329, WP4396, WP4585, WP4658, WP4666, WP4884, WP4961, WP4969, WP706

8.5 Mouse MLO-Y4 osteocyte-like cell line

8.5.1 Summary

	GSE42874	SRP060567 / GSE70667	SRP212008
**Sample information**			
Reference	Govey et al. (2014)	Govey et al. (2015)<br>(includes comparison with GSE42874)	Li et al. (2019)
Download[1]	Gene Expression Omnibus (GEO); GSE42874	recount3; SRP060567<br>(published as GSE70667 on GEO)	recount3; SRP212008<br>(published as BioProject PRJNA551282)
Platform	Affymetrix Mouse Genome 430A 2.0 Array<br>(GPL8321; Mouse430A_2)	Illumina HiSeq 2500 (Mus musculus) (GPL17021)	Illumina NextSeq 550
Type:	Microarray	RNA-seq	RNA-seq
Cell type	Mouse MLO-Y4 osteocyte-like cells	Mouse MLO-Y4 osteocyte-like cells	Mouse MLO-Y4 osteocyte-like cells
Samples	total n=24; 3x FSS (2 h FSS + 2 h post-FSS);<br>control: 3x (0 h FSS + 2 h post-FSS)	total n=6; 3x FSS + 3x Control	total n=6; 3x FSS + 3x Control
FSS application of selected samples	pre-cultivation on collagen I-coated glass slides (2 d); FSS: sinusoidally oscillating FF w/ peak shear stress of 10 dyn/cm^2 @ 1 Hz for 2 h and post-FSS incubation for 0/2/8/24 h; control: paired sham	pre-cultivation on collagen I-coated glass slides (2 d); FSS: sinusoidally oscillating FF w/ peak shear stress of 10 dyn/cm^2 @ 1 Hz for 2 h and post-FSS incubation for 2 h; control: paired sham	ibidi Pump System; FSS: 15 dyn/cm^2 oscillatory FSS @ 1 Hz for 2 h; untreated samples as controls
**easyGSEA**[2]	Pre-ranked gene set enrichment analysis using default settings; DB: KEGG, WikiPathways, GO/Biological Process; gene set size filters: min=15, max=500; results in 3168 from 13304 gene sets		
File:	mmMLOY4_GSE42874_AE_topfit.rnk	mmMLOY4_GSE70667_recount3_SRP060567_DESeq2_shrinked_sorted_log2FC.rnk	mmMLOY4_SRP212008_recount3_DESeq2_shrinked_sorted_log2FC.rnk
Number of genes considered:	6887 / 6891	14450 / 14450	15133 / 15133
Summary report:	61 (down) 139 (up) gene sets are sig. enriched at P_{adj} < 0.25	39 (down) 49 (up) gene sets are sig. enriched at P_{adj}< 0.25<br>7 (down) 8 (up) gene sets are sig. enriched at P_{adj} < 0.05	155 (down) 271 (up) gene sets are sig. enriched at P_{adj} < 0.25<br>20 (down) 32 (up) gene sets are sig. enriched at P_{adj}< 0.05
**easyVizR**[3]	Default filter settings, i.e., P<0.05; FDR < 1.1; |ES| > -0.1. Filtered only according to *p* value!		
Filtered list:	234 out of 2173 total	228 out of 2173 total	431 out of 2173 total
Intersection:	**Overlap of 26 (4%) gene sets between all three studies**		

[1] URL: https://www.ncbi.nlm.nih.gov/geo/query/acc.cgi?acc=GSE42874; recount3 study explorer (https://jhubiostatistics.shinyapps.io/recount3-study-explorer/)
[2] URL: https://tau.cmmt.ubc.ca/eVITTA/easyGSEA/ (2023-04-25)
[3] URL: https://tau.cmmt.ubc.ca/eVITTA/easyVizR/ (2023-04-25)

8.5.2 GSE42874, GSE70667 and SRP212008: easyVizR comparison of the pre-ranked easyGSEA results

Sorted according to descending ES of GSE42874 GSEA results; at least one of the GSEA results showed FDR ≤ 0.1 for a given gene set (green).

ES = enrichment score; FDR = false discovery rate; NES = normalized enrichment score; Size = size statistics reported by fGSEA

Gene set	Gene set Id	GSE42874						GSE70667						SRP212008					
		ES	*P* value	FDR	NES	Size	Leading Edge	ES	*P* value	FDR	NES	Size	Leading Edge	ES	*P* value	FDR	NES	Size	Leading Edge
BP EXTRACELLULAR MATRIX ORGANIZATION	GO:0030198	0.3789	0.0327	0.2373	1.4751	50	NFKB2; APBB2; MMP13; DNAJB6; ADAMTSL4; COL5A3; COL16A1; COL8A1; TGFBI; BCL3; COL1A1; OLFML2B; COL3A1; TNF; COL4A1; FN1; B4GALT1; COL4A2; HSPG2; HSD17B12; SOX9; COL5A1; MMP14; SMARCA4	-0.4944	0.0223	0.4392	-1.4597	129	ERO1A; COL1A2; TFIP11; MMP2; PDGFRA; LGALS3; ADAMTSL4; ERCC2; COL3A1; FBLN5; SMARCA4; COL16A1; FBLN1; COL1A1; SOX9; MMP11; VHL; COL4A2; COL5A2; COL5A3; ADAMTS5; FN1; ADAMTS14; POMT1; OLFML2B; TNXB; ATXN1L; COL18A1; MMP14; FOXF2; COL4A1; MMP28	0.4123	0.0047	0.0771	1.5917	146	ADAMTS16; VTN; DMP1; MMP13; TNF; MMP10; ERO1A; COL5A3; RXFP1; ADAMTS6; CRISPLD2; COL22A1; MMP3; PXDN; FOXF1; SOX9; TNFRSF11B; CCN1; COL7A1; COL17A1; NID1; FBLN1; APBB2; AGT; FOXF2; FN1; COL4A1; TNXB; COL27A1; SMOC2; MATN4; EMILIN1; ERCC2
BP POSITIVE REGULATION OF PROTEIN LOCALIZATION TO PLASMA MEMBRANE	GO:1903078	0.4664	0.0273	0.2144	1.5772	27	EPHA2; RANGRF; EZR; RHOG; LRP1; AKT1; ACTR3; SQSTM1; TNF; MYO5A	0.6817	0.0424	0.4747	1.7110	48	AKAP5; RAB11A; DLG1; KIF5B	0.4416	0.0433	0.2542	1.4310	49	GRIP1; RAMP3; TNF; EZR; SQSTM1; STAC3; EGFR; RANGRF; WNK3; AKAP5; STX3; ZDHHC5; DLG1; TREM2; ACTR3; LRP1; ITGB1; EPHA2; PRKCI
WP SPINAL CORD INJURY	WP2432	0.4034	0.0182	0.1884	1.5803	48	CCND1; CXCL1; IL1R1; CCL2; CXCL2; CXCL10; ANXA1; RHOB; IL6; PRKCA; FKBP1A; NTN1; BDNF; TNF; COL4A1; CD47	0.7091	0.0036	0.0909	1.8420	81	CXCL2; CXCL1; CCL2; MAPK1; CCNG1	0.4980	0.0024	0.0327	1.7933	88	CXCL1; CXCL2; SLIT1; RHOB; GDNF; TNF; CCL2; GADD45A; CSPG4; CHST11; ZFP36; EFNB2; FOS; XYLT1; MYC; BTG2; SOX9; EGR1; KLK8; COL4A1; TGFB1; BDNF; IL6
KEGG COMPLEMENT AND COAGULATION CASCADES	MMU04610	0.5471	0.0287	0.1791	1.6203	16	PLAU; PLAUR; SERPINE1; PLAT; F2R; THBD; C1S1; MASP1	0.7249	0.0073	0.0974	1.8636	60	KLKB1; CR1L; CD46	0.7200	0.0023	0.0312	2.4086	58	SERPINB2; VTN; CLU; PROCR; SERPINE1; F3; THBD; ITGB2; C3; PLAUR; C4B; C1S1; C1S2; BDKRB2; CFB; BDKRB1; PLAT; KNG2; PLAU; C1RB; PROC; C1RA; F13A1; KNG1; VWF; C4A
BP NEGATIVE REGULATION OF SIGNAL TRANSDUCTION	GO:0009968	0.5543	0.0102	0.1357	1.7309	19	SOCS3; RGS3; MFHAS1; PMEPA1; SOCS5; GSK3B; PAK1IP1; RGS17; CAV1	-0.7232	0.0043	0.1779	-1.7895	45	ARRB1; SOCS1; GSK3A; RGS3; SEC14L1	0.5891	0.0044	0.0751	1.8884	47	RGS16; SOCS3; SOCS1; PMEPA1; SOCS2; SOCS5; MFHAS1; SOCS7; DDIT4; UBASH3B; RGS3; BRAP; RGS17; SOCS6; SOCS4; ACVR1; RGS5
WP CYTOPLASMIC RIBOSOMAL PROTEINS	WP163	-0.4534	0.0014	0.1174	-1.7321	70	RPL37; RPL28; RPL35A; RPL30; RPL10A; RPS7; RPS19; RPS15; RPS12; RPL13; FAU; RPL38; RPS3; RPLP0; RPL23A; RPS4X; RPL29; RPL34; RPL27; RPL17; RPS11; RPL13A; RPL26; RPL35; RPL39; RPS23; RPS18; RPL12; RPL9; RPS10; RPS3A1; RPS20; RPL18; RPL19; RPL24; RPL15; RPL7; RPS24; RPLP1	0.6199	0.0345	0.2887	1.6209	83	RPL39; GM7429; RPL38; RPS15A; RPL23A; RPL37RT; RPLP2; RPL36; RPS25; RPS8; RPL34-PS1; RPL7; RPL37; RPS6KA3; RPS11; RPL30; RPS4X; RPS27A; RPL24; RPS29; RPL22; RPL32L; RPS3A1; RPS19	0.5027	0.0025	0.0327	1.7865	83	RPL28; FAU; RPL37RT; RPS17; RPL36; RPL13; RPL29; RPS27; RPL32L; RPS15A; RPS16; RPLP2; RPL30; RPL21; RPL12; RPL38; RPS21; RPL35A; RPL9; GM7429; RPL24; RPS13; RPLP1; RPL17; RPS6; RPL32; RPL7A; RPS10; RPL34; RPS23; RPS15; RPS28; RPL7; RPS3; RPL37A; RPS11; RPL27; RPL10A; RPL39; RPS7; RPL8; RPL31; RPS24; RPL18; RPS29; RPL37; RPS5; RPL22; RPL35; RPL13A; RPL23A; RPS12; RPS3A1; RPL18A; RPLP0
BP POSITIVE REGULATION OF PROTEIN KINASE B SIGNALING	GO:0051897	0.4459	0.0080	0.1168	1.7627	52	CX3CL1; MFHAS1; IGF1R; PHB; HBEGF; IL6; PDGFA; ILK; THBS1; FGFR1; ENG; GPX1; TNFSF11; TNF; ADAM8	-0.5303	0.0329	0.4500	-1.5007	96	THBS1; GCNT2; RARA; ENG; HBEGF; GPX1; FGFR1; VEGFB; MAZ; AXL; HAX1; FERMT2	0.5694	0.0023	0.0604	2.0722	99	OSM; HBEGF; SPRY2; FGF2; F3; VEGFA; RAMP3; TNF; PTPRJ; THBS1; RET; CASS4; PIK3CB; PDGFA; GDF15; PHLPP1; ADAM8; HPSE; FGFR1; SEMA5A; MFHAS1; FERMT2; PIK3CD; ARFGEF1; FAM110C; EGFR; TGFB1; OSBPL8; TNFSF11; IL6; BAG4
BP POSITIVE REGULATION OF CELL MIGRATION	GO:0030335	0.3650	0.0065	0.1091	1.6646	117	CX3CL1; S1PR1; EPHA2; PLAU; IGF1R; HBEGF; CCL7; SOD2; SEMA4B; ETS1; CSF1; CXCL10; TNFAIP6; NOTCH1; CCL5; PDGFA; PRKCA; COL1A1; ILK; PDCD10; THBS1; ACTN4; CREB3; HSPA5; PIK3R1; ACTG1; ATM; ITGAV; TWIST2; PDPN; F2R; FN1; ELP5	-0.4871	0.0094	0.2949	-1.5339	206	THBS1; GCNT2; SEMA3B; ALDOA; STAT3; GPNMB; ITGA5; MMP2; DAPK3; HBEGF; NUMB; BCAR1; GRN; PDGFRA; FAM89B; MYO1C; DAB2; FURIN; EPHA2; ACTN4; GNAI2; NOTCH1; TWIST1; COL1A1; MAZ; RIPOR1; SEMA6B; ACTG1; AKT2; FERMT2; TRIP6; FAM83H; TJP1; PDGFRB; STX4A	0.4116	0.0026	0.0604	1.6546	220	PLET1; CSF2; HBEGF; LRRC15; SPRY2; CCR1; VEGFA; ITGB3; SPHK1; TNFAIP6; ITGA5; PDPN; SEMA6D; CDH13; LAMC2; THBS1; NOTCH1; MMP3; RET; LEF1; SEMA3E; CASS4; SEMA7A; FOXF1; ZFP703; SEMA4G; CCN1; PDGFA; MDK; HPSE; SEMA4B; CSF1; CEMIP; SEMA5A; PDGFC; CPEB1; CCL7; HSPA5; FERMT2; PIK3CD; ETS1; FN1; FAM110C; EGFR; PTP4A1; PLAU; TGFB1; CCL5; RUFY3; CBLL1; ITGAV; PTK2B; FURIN; DAAM2; ACVR1
BP G PROTEIN-COUPLED RECEPTOR SIGNALING PATHWAY	GO:0007186	0.3994	0.0058	0.1061	1.6241	62	CX3CL1; S1PR1; RGS3; CCL7; CCL2; PTGER4; CXCL10; GNG12; TGM2; CCL5; ACKR3; GNB4; GNB2; PTGER3; CCL9; GNA11; RGS17; GDE1; F2R; GNAI3	-0.4419	0.0097	0.2965	-1.4271	272	ARRB1; APLN; MRGPRF; ADRA1D; FZD7; BCAR1; GNB2; ITPR3; PLCB3; FZD2; GNAI2; RGS3; ARHGEF1	0.4021	0.0027	0.0605	1.6924	315	AREG; GPR68; GPR84; P2RY2; GRK3; ADRA1B; RGS16; CCR1; GPR34; ACKR2; TBXA2R; RAMP3; GLP2R; PTGIR; APOE; CCL2; HTR1D; GPRC5A; ADGRA1; RHO; ADGRG1; FZD5; RXFP1; TAS1R3; TAFA5; PTGDR2; TGM2; ADGRG5; ADORA2A; BDKRB2; HCAR2; HTR2A; FZD9; HTR1B; XCR1; QRFPR; GPR35; GPR153; HTR2B; PLPPR4; PROKR1; P2RY6; GNGT2; BDKRB1; GNB4; AKAP13; GALR2; ADGRB2; S1PR3
BP RRNA PROCESSING	GO:0006364	0.4314	0.0033	0.0820	1.9641	121	BOP1; NOP10; RRP15; TFB2M; NHP2; EMG1; RRP12; WDR12; FBL; UTP4; RPF2; MRTO4; RCL1; DIMT1; RRP8; RPF1; UTP18; RRP7A; LYAR; NOP14; PWP1; DCAF13; DICER1; UTP6; GAR1; BRIX1; MPHOSPH10; DIS3; RRP9; WDR75; DDX56; TBL3; IMP4; NAF1; PA2G4; NOP2; UTP20; BYSL; DDX21; DDX10; ESF1; PES1; FTSJ3; EXOSC1; DDX49; MRM3; EBNA1BP2; TSR2; DDX27; RPP30; PPAN; EXOSC10; RRP1B; WDR36	0.5393	0.0362	0.4648	1.5475	149	RPS27RT; UTP18; RPF2; RPP30; DIMT1; BRIX1; KRR1; MPHOSPH10; NOL8; DIS3; WDR12; RPS25; WDR75; PWP1; RPL7; TENT4B; DDX10; DDX21; NAF1; RPP40; ESF1; RRP15; RPF1; EXOSC9; NOL11; ERI1; C1D; MTREX; DKC1; EXOSC1; MPHOSPH6; DCAF13; UTP20; RPS19; UTP15; WDR36; NOP10; UTP6; UTP4; NOP14; UTP23; TFB1M; POP4; WDR43; UTP14A	0.5962	0.0023	0.0604	2.3125	150	RRP1B; RRP9; RRP12; UTP15; NOP2; DIMT1; CHD7; ISG20; PDCD11; WDR55; NAF1; RCL1; DDX10; UTP4; LYAR; UTP14B; GAR1; PPAN; WDR43; TENT4B; NOL8; IMP4; NOL9; UTP20; EBNA1BP2; MRTO4; DDX21; UTP18; NOL6; MPHOSPH6; NOP14; MPHOSPH10; RRP15; ESF1; PWP1; FTSJ3; LAS1L; SBDS; DDX27; NSUN5; EXOSC6; BRIX1; DKC1; WDR74; TFB2M; UTP6; UTP25; DDX54; RPS17; DDX51; RRP8; RPS27; KRR1; NHP2; RPF2; POP7; WDR12; DIS3; UTP14A; DDX56; RPS16; IMP3; FBL; RPS27RT; BYSL; WDR75; DDX49; RRP36; NAT10; NVL; PES1; RPP40; EXOSC3; EXOSC2; DDX17; TSR2; PA2G4; DICER1; RPL35A; WDR36; DCAF13; UTP23; REXO4;

Gene set	Gene set Id	GSE42874						GSE70667						SRP212008					
		ES	*P* value	FDR	NES	Size	Leading Edge	ES	*P* value	FDR	NES	Size	Leading Edge	ES	*P* value	FDR	NES	Size	Leading Edge
																			ERI1; EXOSC1; EMG1; EXOSC10; BOP1; NPM3; MRM3; TSR3; RPUSD4; DDX47; RPS6; TBL3; EXOSC5; RPF1; FCF1
BP RIBOSOME BIOGENESIS	GO:0042254	0.5827	0.0032	0.0820	2.5240	88	SNU13; RRN3; RRS1; BOP1; NOP10; EIF6; NHP2; EMG1; GLUL; WDR12; UTP4; RPF2; NIP7; MRTO4; RIOX1; RCL1; NOC4L; RPF1; DDX3X; RSL24D1; MYBBP1A; NOP14; PWP1; DCAF13; SURF6; GAR1; BRIX1; MPHOSPH10; GTPBP4; TSR1; WDR75; AATF; DDX56; RIOK3; IMP4; NAF1; NOP58; NOP2; PAK1IP1; GRWD1; RIOK2; BYSL; TMA16; RIOK1	0.6043	0.0147	0.3442	1.6800	108	XPO1; RIOK3; RPF2; BRIX1; KRR1; DDX3X; MPHOSPH10; MTERF3; GTPBP4; AATF; GTPBP10; NOP58; PRKDC; WDR12; WDR75; PWP1; RBIS; NAF1; RIOK2; TMA16; RPF1; NOL11; CUL4B; DKC1; RRN3; FASTKD2; SDAD1; DCAF13; MALSU1; UTP15; SPATA5; XRCC5; NOP10; RIOX2; UTP4; NOP14; UTP23; TSR1; WDR43; UTP14A; FCF1; RPL7A; PAK1IP1; DDX31; ZNHIT6; SNU13; NSA2; NHP2	0.6750	0.0023	0.0604	2.4795	108	GLUL; ZFP593; RIOX1; TMA16; RRS1; UTP15; NOP2; SURF6; DDX31; NIP7; RIOK2; NAF1; TSR1; RCL1; NOP56; UTP4; SDAD1; UTP14B; GAR1; URB2; WDR43; SPATA5; IMP4; EIF6; ZFP622; DHX37; GTPBP4; RIOX2; NOC4L; EBNA1BP2; MRTO4; RRN3; NOP14; MPHOSPH10; GNL2; MYBBP1A; SNU13; PWP1; FTSJ3; ZNHIT6; SBDS; DDX27; GRWD1; NOP58; AATF; BRIX1; MTERF3; DKC1; FASTKD2; DDX51; KRR1; NHP2; RPF2; WDR12; C1QBP; 9930104L06RIK; UTP14A; ISG20L2; DDX56; IMP3; RIOK1; BYSL; SDE2; RSL24D1; WDR75; RRP36; NVL; PES1
BP RIBOSOMAL SMALL SUBUNIT BIOGENESIS	GO:0042274	0.4484	0.0028	0.0820	1.8231	59	SNU13; EMG1; PWP2; FBL; UTP4; RCL1; DIMT1; UTP18; PNO1; HEATR1; UTP6; SURF6; MPHOSPH10; RRP9; DNTTIP2; WDR75; AATF; TBL3; IMP4; NOP58; UTP20; LTV1; RIOK2	0.6270	0.0403	0.4672	1.6716	71	RPS27RT; RPS15A; UTP18; PNO1; DIMT1; KRR1; MPHOSPH10; AATF; NOP58; RPS25; WDR75; NPM1; RPS8; DNTTIP2; RIOK2; RPS11; WDR3; LTV1; UTP20; RPS3A1; RPS19; UTP15; WDR36; UTP6; UTP4; WDR43; FCF1; HEATR1; NOL7; BMS1; SNU13	0.6919	0.0024	0.0604	2.3702	71	METTL17; RRP9; PWP2; UTP15; SURF6; DIMT1; RIOK2; RCL1; WDR3; HEATR1; NOP56; UTP4; WDR43; DNTTIP2; IMP4; DHX37; UTP20; UTP18; LTV1; NOL6; MPHOSPH10; NOM1; SNU13; ERCC2; NOP58; AATF; NOB1; WDR46; BMS1; RPS19BP1; UTP6; RPS17; RPS27; PNO1; KRR1; RPS15A; RPS16; IMP3; RIOK1; FBL; RPS27RT; WDR75; NPM1; RRP36; NAT10
BP INFLAMMATORY RESPONSE	GO:0006954	0.4980	0.0033	0.0820	2.2821	125	CX3CL1; IL1RL1; CXCL5; EPHA2; MFHAS1; NINJ1; PTGS2; VCAM1; NFKB1; NFKB2; CXCL1; CCL7; IL1R1; CCL2; MAPKAPK2; CD44; MYD88; TNFRSF1B; TLR2; CSF1; CXCL2; RELA; PTGER4; RELB; CXCL10; MAP2K3; ANXA1; CASP7; IL6; DDX3X; MAP3K7; CCL5; THBS1; PTGER3; CCL9; AKT1; NFKBIB; NFKBIZ; NFE2L2; NDST1; TNF; CD47; ITGAV; CUL3; ADAM8	0.6497	0.0017	0.1232	1.9593	263	CXCL5; KLKB1; CXCL2; CXCL1; NLRP3; NOD2; CCL2; TLR2; VCAM1; REL; ACER3; DDX3X; TMED2; MAPK8; PLAA; HMGB1; CUL3; NFE2L2; PTGFR; NFKB1; NFKB2; TMEM258; DHX9	0.3702	0.0026	0.0604	1.5298	275	S100A8; CXCL1; CXCL2; PTGS2; TSPAN18; TNFAIP3; CCR1; IL1RL1; CRHBP; ACKR2; SPHK1; TBXA2R; TNF; IL1RN; KDM6B; PTGIR; C3; CCL2; NLRP3; THBS1; CXCL5; CSPG4; GGT5; IL27; SEMA7A; TNIP2; IL17RA; BMPR1B; ZC3H12A; ADAM8; HPSE; NFKBID; CXCL3; BDKRB1; CSF1; S1PR3; TNFRSF4; CYBA; CCL7; MFHAS1; MACIR; PIK3CD; HAVCR2; LY96; TGFB1; CD44; BMP6; CCL5; KRT16; NFKB1; PTGER4; MAP2K3; ECM1; KNG1; TNFRSF1B; IL6; HNRNPA0; IL1RL2; MAPKAPK2; ITGAV; VWF; NDST1; CXCR6; SCYL3; CRLF2
BP POSITIVE REGULATION OF INFLAMMATORY RESPONSE	GO:0050729	0.4864	0.0027	0.0820	1.8571	46	IL1RL1; NINJ1; CCN4; ETS1; TLR2; IL33; PTGER4; SERPINE1; TGM2; CLOCK; PRKCA; FGFR1; NFKBIZ; TNF; CD47; ADAM8; NMI; NFKBIA; LGALS1	0.6885	0.0056	0.2085	1.8410	76	NLRP3; TLR2; CEBPB; JAK2; CLOCK; HSPD1; CCN4	0.4724	0.0072	0.0968	1.6496	81	OSM; S100A8; ITGA2; IL33; SERPINE1; IL1RL1; NFKBIA; TNF; CCN4; NLRP3; TGM2; IL17RA; MDK; CAMK2N1; ADAM8; FGFR1
BP IMMUNE RESPONSE	GO:0006955	0.4710	0.0029	0.0820	1.9211	61	CX3CL1; SLPI; CXCL5; IGF1R; CXCL1; CCL7; PLSCR1; CCL2; MYD88; TNFRSF1B; TLR2; CXCL2; CXCL10; IL6; CCL5; ACKR3; H2-Q4; CCL9; TNFSF11; TNF; IRAK1BP1	0.7440	0.0018	0.1232	2.1701	167	CXCL5; CXCL2; CXCL1; CCL2; FAS; TLR2; JAK2	0.3268	0.0448	0.2542	1.2856	177	OSM; CSF2; LIF; CXCL1; CXCL2; VTN; CD1D2; CD1D1; CCR1; ACKR2; TNF; IL1RN; CCL2; CXCL5; CLEC4E; MILL2; H2-AB1; XCR1; CBLB; PRG4; CXCL3; SLPI; ENPP1; H2-T23; CCL7; CTSL; BMP6; CCL5; IL12RB1; TNFSF11; IL12RB2; TNFRSF1B; PLSCR1; MARCHF1; GM7030; IL6; TRAF6
BP DEFENSE RESPONSE	GO:0006952	0.6068	0.0028	0.0820	2.0954	29	CXCL5; MFHAS1; CXCL1; IFNB1; TLR2; CXCL2; RELA; CXCL10	0.8273	0.0019	0.1232	2.1292	57	CXCL5; CXCL2; CXCL1; NOD2; TLR2	-0.5215	0.0036	0.0751	-1.6692	53	CXCL10; TLR3; IRGM2; STAT2; TGTP2; STAT1; TGTP1; GM12185; TAP1; TLR1; IRGM1; NOD1; IFI47; IGTP; STAT5A; IIGP1; STAT3
BP CHEMOTAXIS	GO:0006935	0.6232	0.0028	0.0820	2.2577	36	CX3CL1; S1PR1; CXCL5; NINJ1; CXCL1; CCL7; CCL2; CXCL2; CXCL10; CCL5; ACKR3; CREB3; CCL9	0.8775	0.0019	0.1232	2.3519	79	CXCL5; CXCL2; CXCL1; CCL2; HMGB1	0.3895	0.0413	0.2519	1.3718	86	S100A8; CXCL1; CXCL2; NCKAP1L; CCR1; ACKR2; CCL2; CXCL5; PTGDR2; XCR1; CCN1; CXCL3; CCL7; PIK3CD; CCL5; TYMP
BP NEUTROPHIL CHEMOTAXIS	GO:0030593	0.6575	0.0026	0.0820	2.0983	21	CX3CL1; CXCL5; CXCL1; CCL7; CCL2; CXCL2; CXCL10; CCL5; PRKCA; CCL9	0.9233	0.0019	0.1232	2.2876	44	CXCL5; CXCL2; CXCL1; CCL2; CXCL10	0.5299	0.0044	0.0751	1.6987	47	S100A8; CXCL1; CXCL2; NCKAP1L; ITGB2; CCL2; CXCL5; LBP; BSG; CXCL3; CCL7
KEGG NF-KAPPA B SIGNALING PATHWAY	MMU04064	0.5399	0.0028	0.0747	2.0971	50	PLAU; PTGS2; VCAM1; NFKB1; CSNK2A2; NFKB2; CXCL1; IL1R1; TRAF3; GADD45B; MYD88; CXCL2; RELA; RELB; BCL10; MAP3K7; TNFSF11; ATM; CHUK; TNF; TRAF2; NFKBIA; GADD45G	0.7029	0.0037	0.0663	1.8977	82	CXCL2; CXCL1; VCAM1; ERC1; BIRC2; CHUK; NFKB1; NFKB2	0.4305	0.0047	0.0418	1.5334	87	CXCL1; CXCL2; PTGS2; TNFAIP3; NFKBIA; TNF; GADD45A; TRAF5; LBP; CXCL3; GADD45B; PLAU; LY96; GADD45G; CSNK2A2; NFKB1; TAB3; TNFSF11; TRAF6; BCL10; RELB; TIRAP; ERC1; UBE2I; CARD10
KEGG TNF SIGNALING PATHWAY	MMU04668	0.4806	0.0031	0.0747	2.0370	74	CX3CL1; SOCS3; CXCL5; PTGS2; VCAM1; NFKB1; CXCL1; TRAF3; CCL2; IFNB1; TNFRSF1B; CSF1; JUNB; CXCL2; RELA; CXCL10; MAP2K3; CASP7; IL6; MAP3K7; BCL3; CCL5; CREB3; PIK3R1; AKT1; CHUK; TNF; TRAF2; NFKBIA; MAP2K4	0.7708	0.0018	0.0494	2.1690	108	CXCL5; CXCL2; CXCL1; NOD2; CCL2; FAS; VCAM1; CEBPB; PIK3R1; MAP3K5; MAPK8; MAPK1; CREB1; BIRC2; CHUK; NFKB1; DNM1L	0.4322	0.0025	0.0312	1.5919	110	CSF2; LIF; CXCL1; CXCL2; PTGS2; TNFAIP3; SOCS3; NFKBIA; TNF; CCL2; CXCL5; TRAF5; JAG1; MMP3; FOS; CREB3L2; PIK3CB; JUNB; CXCL3; CSF1; JUN; PIK3CD; CCL5; NFKB1; TAB3; ATF4; MAP2K3; TNFRSF1B; IL6; PGAM5; BAG4
KEGG CYTOKINE-CYTOKINE RECEPTOR INTERACTION	MMU04060	0.7083	0.0027	0.0747	2.8197	52	CX3CL1; IL15RA; INHBA; IL1RL1; CXCL5; CLCF1; TNFRSF12A; CXCL1; CCL7; IL1R1; CCL2; IFNB1; TNFRSF1B; CSF1; CXCL2; IL33; CXCL10; IFNGR2; IL6ST; IL6; CCL5; ACKR3; IL13RA1; IL4RA; CCL9; TNFSF11; TNF	0.7838	0.0017	0.0494	2.2852	172	CXCL5; CXCL2; CXCL1; CCL2; FAS; BMPR2; INHBA	0.4661	0.0026	0.0312	1.8616	190	OSM; NGF; CSF2; LIF; IL11; CXCL1; IL21R; CXCL2; IL33; CLCF1; INHBA; IL13RA2; TNFRSF9; BMP8A; CCR1; IL1RL1; TNF; IL1RN; CCL2; TNFRSF8; CXCL5; IL27; TNFRSF12A; XCR1; IL17RA; TNFRSF11B; GDF15; BMPR1B; IL3RA; IL6RA; CXCL3; RELT; CSF1; AMH; TNFRSF4; CCL7; ACVR2A; GDF5; TGFB1; GDF9; BMP6; CCL5; IL12RB1; TNFSF11; IL12RB2; IL4RA; TNFRSF1B; IL6; IL1RL2; CXCR6; ACVR1; CRLF2; BMPR2
KEGG IL-17 SIGNALING PATHWAY	MMU04657	0.4537	0.0028	0.0747	1.7624	50	CXCL5; PTGS2; NFKB1; CXCL1; CCL7; TRAF3; MMP13; CCL2; CXCL2; RELA; CXCL10; SRSF1; IL6; MAP3K7; FOSL1; CHUK; GSK3B; TNF; TRAF2; NFKBIA	0.8136	0.0018	0.0494	2.1633	73	CXCL5; CXCL2; CXCL1; CCL2; CEBPB; ELAVL1; HSP90AA1; MAPK8; MAPK1; CHUK; SRSF1; NFKB1	0.6273	0.0023	0.0312	2.1961	77	S100A8; CSF2; CXCL1; FOSB; CXCL2; PTGS2; TNFAIP3; MMP13; MUC5AC; NFKBIA; TNF; CCL2; CXCL5; TRAF5; MMP3; FOS; IL17RA; MAPK6; LCN2; FOSL1; CXCL3; JUN; CCL7; NFKB1; TAB3; ELAVL1; IL6; TRAF6

BP, GeneOntology/Biological Process; KEGG, Kyoto Encyclopedia of Genes and Genomes; WP, WikiPathways

Removed gene sets: MMU05164, MMU05146, GO:0050830, WP3632
